# Dirhodium(II)/Phosphine Catalyst with Chiral Environment at Bridging Site and Its Application in Enantioselective Atropisomer Synthesis

**DOI:** 10.1021/acscentsci.2c01207

**Published:** 2023-03-20

**Authors:** Lei Shi, Xiaoping Xue, Biqiong Hong, Qigang Li, Zhenhua Gu

**Affiliations:** † Hefei National Research Center for Physical Sciences at the Microscale and Department of Chemistry, University of Science and Technology of China, 96 Jinzhai Road, Hefei, Anhui 230026, P. R. China; ‡ College of Materials and Chemical Engineering, Minjiang University, Fuzhou, Fujian 350108, China

## Abstract

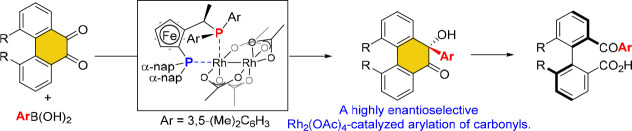

A dirhodium(II)/phosphine catalyst with a chiral environment at the bridging site was developed for the asymmetric arylation of phenanthrene-9,10-diones with arylboronic acids. In contrast to the classic chiral bridging carboxylic acid (or derivatives) ligand strategy of bimetallic dirhodium(II) catalysis, in this reaction, tuning both axial and bridging ligands realized the first Rh_2_(OAc)_4_/phosphine-catalyzed highly enantioselective carbonyl addition reaction. The kinetic analysis reveals that dirhodium(II) and arylboronic acid follow the first-order kinetics, while phenanthrene-9,10-dione is zeroth-order. These data supported the proposed catalytic cycle, where the key intermediate in the rate-determining step involved the dirhodium(II) complex and arylboronic acid. Finally, axially chiral biaryls were prepared based on a newly developed oxidative ring-opening reaction of α-hydroxyl ketones with a base and molecular oxygen, which featured a central-to-axial chirality transfer radical β-scission step.

## Abstract 2

Reactivity and stereoselectivity of dirhodium(II) catalyst were tuned via both axial and bridging position coordination, realizing a Rh_2_(OAc)_4_-catalyzed highly enantioselective arylation of diketones.

## Introduction

**Figure 1:**
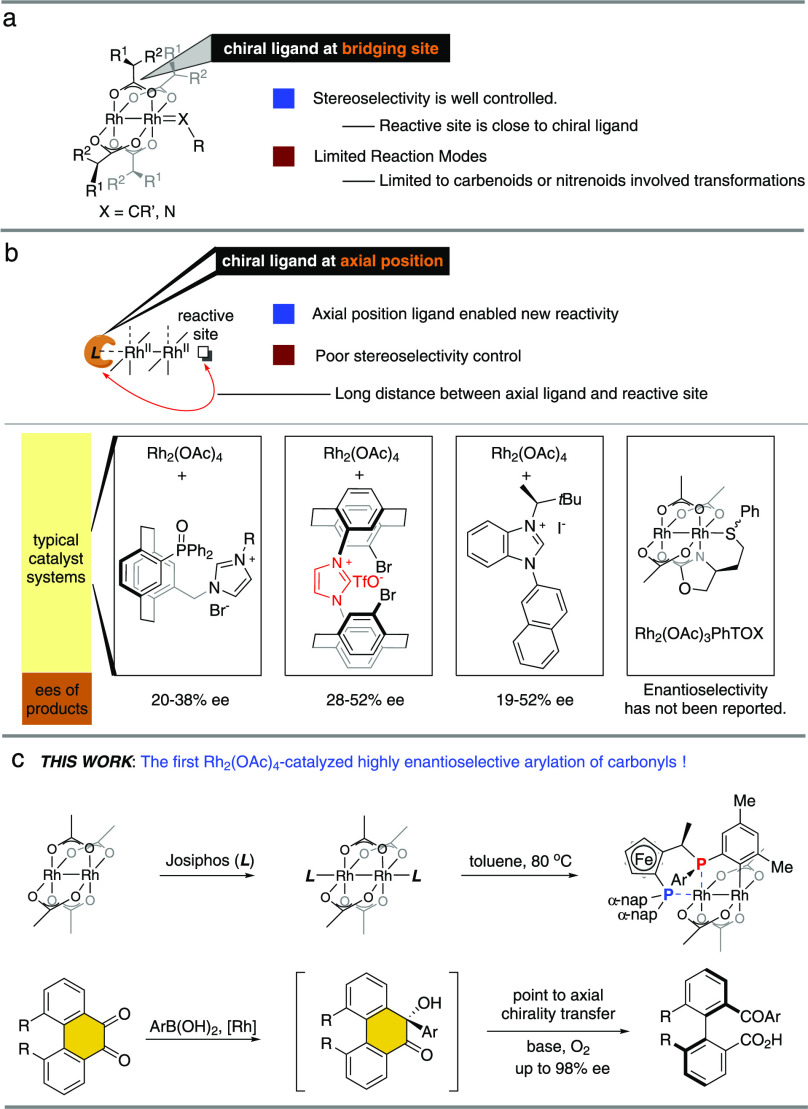
(a) Dirhodium(II) with chiral bridging ligand. (b) Dirhodium(II) with chiral ligand at axial position. (c) This work: [Rh(II)–Rh(II)]/Josiphos-catalyzed high enantioselective carbonyl addition.

Dirhodium(II) complexes, such as Rh_2_(OAc)_4_ or its carboxylic analogues, display unique reactivities, which rely on the unique bimetallic structure, Rh(II)–Rh(II), and each has octahedral molecular geometry. The chiral bridging ligands, which connect two rhodium atoms in the coordination complex, well tune the properties of the complex to realize stereocontrol. However, these reactions mainly locate on the carbenoid or nitrenoid involved transformations (Figure 1a).^1−7^ Besides the bridging ligands, it was also found that fine-tuning of axial ligand(s) of dirhodium(II) complexes can explore new reactivities. In 2001, Fürstner and Krause first proved that IMes, the axial ligand of the dirhodium(II) complex, enabled the dirhodium(II) complexes to show new catalytic activity: the arylations of aldehydes with boronic acids.^8^ In the next two decades, the strategy of adjusting the axial ligands was proven to be a valuable way to tune the reactivities of dirhodium(II) complexes, which boosted the applications of these complexes in organic synthesis.^9−16^

Despite the great successes in asymmetric C–H functionalization and cyclopropanation of carbenoids by using bimetallic dirhodium(II) catalysts with chiral bridging ligands, the asymmetric synthesis by employing achiral bridging ligands and a chiral axial ligand is very challenging. In Bolm, Ma, and Li’s pioneering works, there was no example of excellent stereoinduction catalyzed by dirhodium(II) complex with chiral axial ligand as the only chiral source.^17−20^ Over the last 20 years, the enantiomeric excesses of all the reported arylation reactions of carbonyls with a combination of achiral dirhodium(II) and chiral axial ligand only ranged from ∼20–52%. These observations were possibly due to the chiral environment being far from the reactive center: the chiral ligand is at one axial position of dirhodium(II) complexes, and the reactive center is at the opposite axial site (Figure 1b). In contrast to the well-established Rh(I)-catalyzed addition of arylmetal reagents to aldehydes, there are limited examples of Rh-catalyzed highly enantioselective carbonyl addition reactions, which directly give optically active alcohols, including sterically bulky tertiary alcohols.^21−28^ These reactions still suffer from the narrow substrate scope, particularly the lack of methods for high stereoselective addition to ketones. Herein we report the development of a dirhodium(II)/Josiphos catalyst, which bears an axial phosphine ligand to tune the reactivity and bridging ligand to create a chiral environment (Figure 1c). The new catalyst has been applied in asymmetric arylation of diketones. Moreover, these obtained optically active α-hydroxyl ketones could be efficiently converted to axially chiral biaryls by treatment with a base and molecular oxygen.

## Results and Discussion

### Reaction Conditions Optimization

4,5-Dimethylphenanthrene-9,10-dione **1a** and phenylboronic acid **2a** have been selected as model substrates, and the initial trials focused on Rh_2_(OAc)_4_ as the catalyst with various chiral phosphine ligands (Table 1). Notably, without the assistance of phosphine ligand the reaction is very sluggish, and only a trace amount of product was observed (entry 1). A survey of diverse chiral phosphines indicated that these ligands not only induce stereochemistry but also dramatically improve the reactivity, which is beneficial for catalytic asymmetric reactions by minimizing the background reaction. The Josiphos (**L1**–**L6**) resulted promising stereoselectivity, where **L6** gave the best outcomes regarding both the yield and ee value (entries 2–7). Furthermore, the reaction is fairly robust and can be scaled up to 1.0 mmol without deteriorating either the yield or stereoselectivity (entry 8). With K_3_PO_4_, or even in the absence of a base, the reaction still worked; however, the yields of **3a** decreased and the same enantiomeric excesses were obtained (entries 9 and 10). For comparison, several rhodium(I) catalysts were tested, all trials gave reduced yield or stereoselectivity, showing the superiority of Rh_2_(OAc)_4_ in this asymmetric arylation reaction (entries 11–13).

**Table 1:**
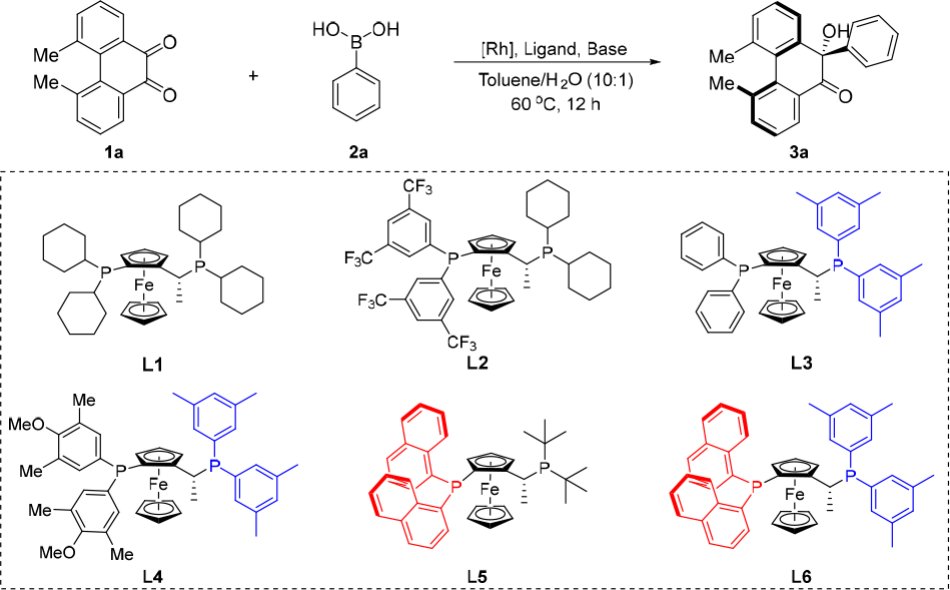
Optimization of Reaction Conditions^a^

entry	ligand	[Rh]	base	yield/ee of **3a**/%
1	**/**	Rh_2_(OAc)_4_	K_2_CO_3_	trace/–
2	**L1**	Rh_2_(OAc)_4_	K_2_CO_3_	85/63
3	**L2**	Rh_2_(OAc)_4_	K_2_CO_3_	95/46
4	**L3**	Rh_2_(OAc)_4_	K_2_CO_3_	73/72
5	**L4**	Rh_2_(OAc)_4_	K_2_CO_3_	65/72
6	**L5**	Rh_2_(OAc)_4_	K_2_CO_3_	24/30
7	**L6**	Rh_2_(OAc)_4_	K_2_CO_3_	96/96
8^b^	**L6**	Rh_2_(OAc)_4_	K_2_CO_3_	99/96
9	**L6**	Rh_2_(OAc)_4_	K_3_PO_4_	86/96
10^b^	**L6**	Rh_2_(OAc)_4_	/	30/97
11	**L6**	[Rh(C_2_H_4_)_2_Cl]_2_	K_3_PO_4_	56/85
12	**L6**	[Rh(COD)_2_BF_4_]_2_	K_3_PO_4_	92/67
13	**L6**	[Rh(CO)_2_Cl]_2_	K_3_PO_4_	23/33

a Conditions: **1a** (0.10 mmol), **2a** (0.30 mmol), Rh (2.5 mol %), and ligand (5.0 mol %) in toluene (2.0 mL) and H_2_O (0.2 mL) at 60 °C for 12 h.

b The scale: **1a** (1.0 mmol), **2a** (2.5 mmol) in toluene (10 mL) and H_2_O (1.0 mL).

### Reaction Scope and Selectivity

**Scheme 1:**
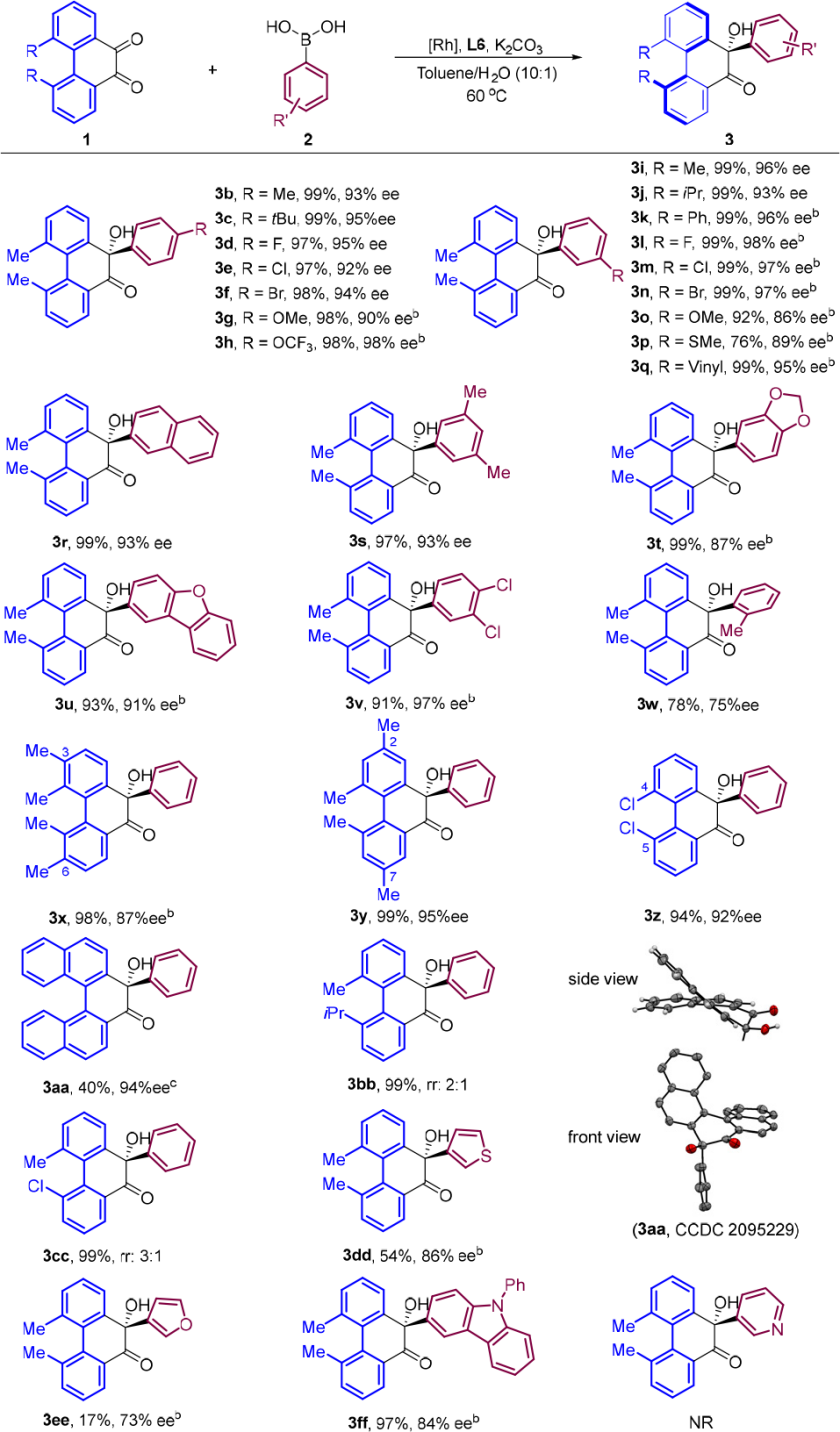
Substrate Scope of Arylation Reaction conditions: **1** (0.40 mmol) and **2** (1.0 mmol), Rh_2_(OAc)_4_ (2.5 mol %), **L6** (5.0 mol %) and K_2_CO_3_ (0.40 mmol) in 3.3 mL of solvent (3.0 mL of toluene and 0.3 mL of H_2_O) at 60 °C for 12 h. **2** (1.2 mmol), K_2_HPO_4_·3H_2_O (0.40 mmol), 80 °C. **2** (1.2 mmol), KH_2_PO_4_ (1.2 mmol), 80 °C.

With the validity of our concept established, the dirhodium(II)/Josiphos-catalyzed asymmetric arylation was applied to different substituted phenanthrene-9,10-diones and boronic acids (Scheme 1). The substituent effect at the *para* and *meta* positions of phenylboronic acids was investigated. It can be alkyl, halogen atom, methoxy, trifluoromethoxy, as well as vinyl group (**3b**–**3q**). The reactions with phenylboronic acids bearing multisubstituents also proceed uneventfully (**3r**–**3v**). The stereoselectivity slightly decreased when the arylboronic acids contain electron-donating group, i.e., methoxy, methylthio, and [1,3]-dioxol (**3g**, **3o**, **3p**, and **3t**). Both the reactivity and selectivity are sensitive to *ortho* substituted phenylboronic acids: compound **3w** was obtained in 78% yield with 75% ee. Introduction of dimethyl groups to 3,6-positions of phenanthrene-9,10-diones slightly decreased the stereoinduction (**3x**), while the reactions of 2,4,5,7-tetramethyl or 4,5-dichloro phenanthrene-9,10-diones worked as efficiently as the model compound (**3y** and **3z**). The binaphthyl analogue resulted in a decreased yield, while the enantiomeric excess still reached 94% [(*S*)-**3aa** (CCDC 2095229)]. The arylation of nonsymmetric phenanthrene-9,10-diones by replacing one methyl group with either an isopropyl or chloro group gave poor regioselectivity (**3bb** and **3cc**). For heteroaromatic boronic acids, the thiophene and carbazole derivatives can be obtained in moderate to excellent yields, with the ee values still maintaining at high levels (**3dd** and **3ff**). However, 3-furanylboronic acid resulted in a dramatic decrease of reactivity, and the enantioselectivity also drops significantly (**3ee**). 3-Pyridinylboronic acid is not a compatible substrate, which is possibly due to the coordinative ability of the pyridine unit (**3gg**).

**Scheme 2:**
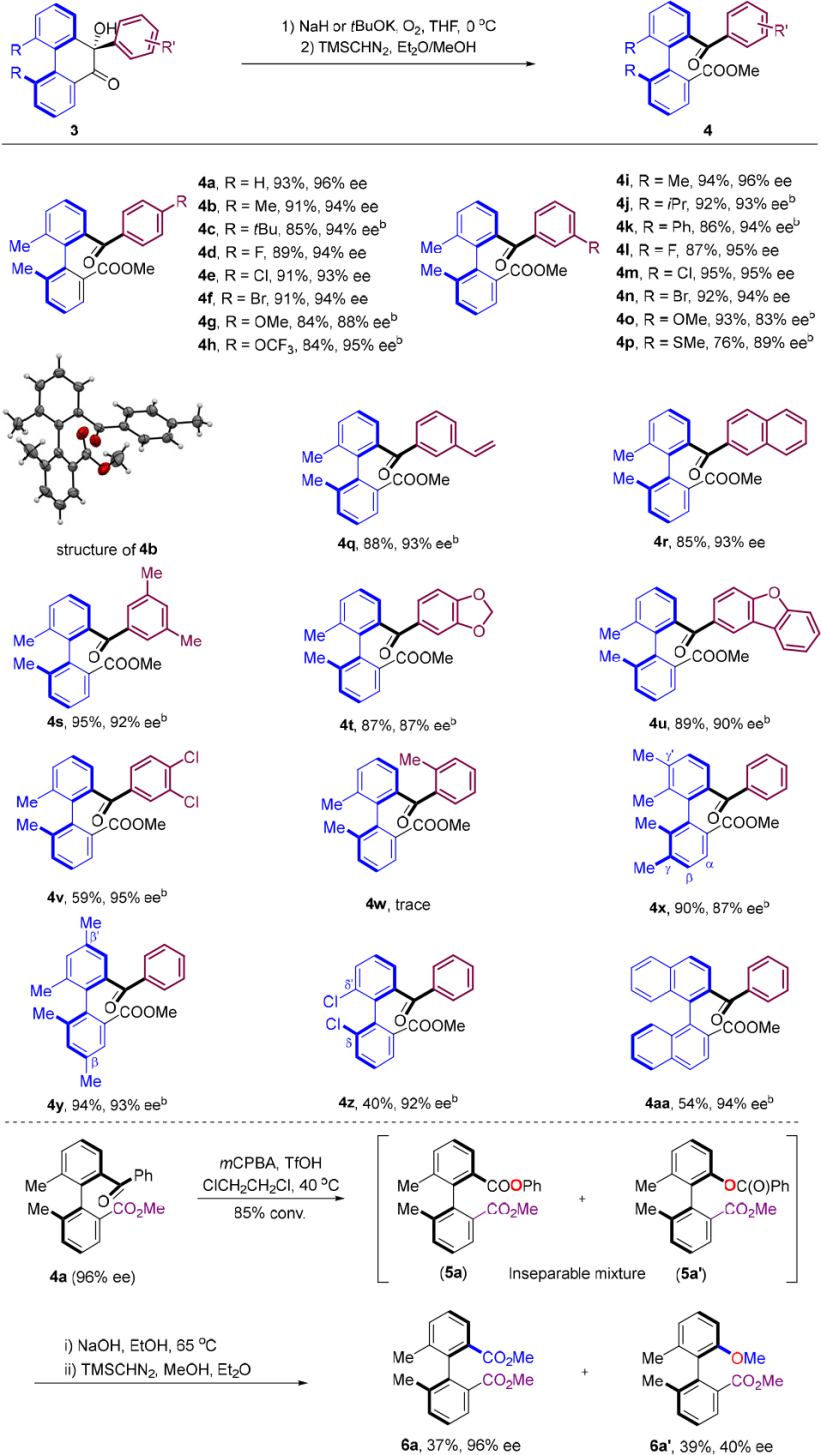
Substrate Scope of Oxidative Ring-Opening Reaction conditions: (1) **3** (0.20 mmol) and NaH (0.80 mmol) in 3.0 mL THF under 1 atm of O_2_ at 0 °C for 3 h. (2) TMSCHN_2_ (1.0 mmol), Et_2_O (1 mL), and MeOH (1 mL) were used under N_2_ atmosphere at rt for 1 h. *t*BuOK (0.80 mmol) was used. Reaction conditions:(1) **4a** (0.20 mmol), *m*CPBA (2.0 mmol) and TfOH (0.2 mmol) in 3.0 mL DCE under N_2_ atmosphere at 40 °C for 48 h. (2) NaOH (2.0 mmol) and EtOH (2 mL) were used under N_2_ atmosphere at 65 °C for 10 h. (3) TMSCHN_2_ (1.0 mmol), Et_2_O (1 mL), and MeOH (1 mL) were used under N_2_ atmosphere at rt for 3 h.

Atropisomerism is one of the key elements of chirality, and biaryl atropisomers are widespread in natural products and pharmaceuticals.^29−37^ Herein, we anticipate preparing axially chiral biaryls via ring-opening reaction^38−46^ from these optically active α-hydroxyl ketones **3**. However, the challenge is to develop an efficient method that can break the C–C bond at mild conditions, and at the same time the central chirality of tertiary alcohol can be efficiently transferred to the axial chirality.^47−56^ It is well-established that α-hydroperoxy ketones readily undergo C–C bond breaking reaction via the degradation of hydroperoxide to the oxygen-centered radical, followed by β-scission to form acyl radial. After investigation of a coupling of metal oxidants, we were surprised to find the oxidative ring-opening reaction proceeded efficiently to give carboxylic acid under 1 atm of O_2_ upon the treatment of excess NaH or KO*t*Bu at 0 °C. In order to make the purification and enantiomeric excess analysis easy, the carboxylic acids were esterified to the methyl esters. Notably, this reaction condition is fairly mild and it proceeded with 97% to full transfer of chirality. The *para* and *meta*-substituents at the phenyl ring had a marginal effect on this ring-opening reaction (**4a**–**4o**), except the substrate bearing methylthio group (**4p**) (Scheme 2). The absolute configuration of **4b** was determined by X-ray crystallographic analysis (CCDC 2095230). Although the reaction might proceed through a radical pathway (see below for a detailed discussion), the aryl-halogen bond as well as the vinyl group were well tolerated (**4d**–**4f**, **4l**–**4n**, and **4q**). This reaction could be applied to substrates bearing big aryls (**4r**–**4v**); however, the *o*-methylphenyl derivative proceeded in a quite low efficacy (**4w**). Introducing γ,γ′- or β,β′-dimethyl did not alter the virtue of this base-promoted chirality transfer reaction (**4x** and **4y**), while dichlorobiphenyl or binaphthyl atropisomers were obtained in decreased yields (**4z** and **4aa**). Baeyer–Villiger oxidation of **4a** with *m*CPBA and triflic acid gave inseparable regioisomeric esters **5a** and **5a′**. Subsequently, hydrolysis, followed by treatment with TMSCHN_2_ gave bicarboxylic ester **6a** in 37% overall yield, along with 39% yield of methyl ether **6a′** in a decreased ee value.

### Mechanistic Studies

**Figure 2:**
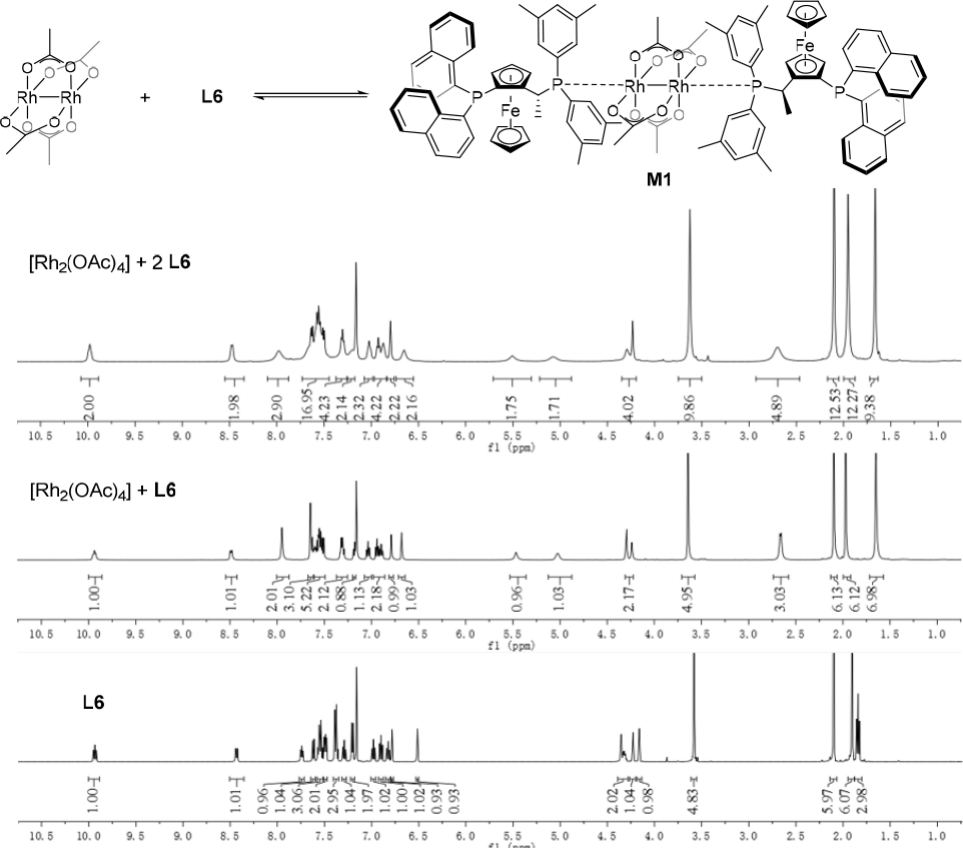
Complexation analyzed by ^1^H NMR.

**Figure 3:**
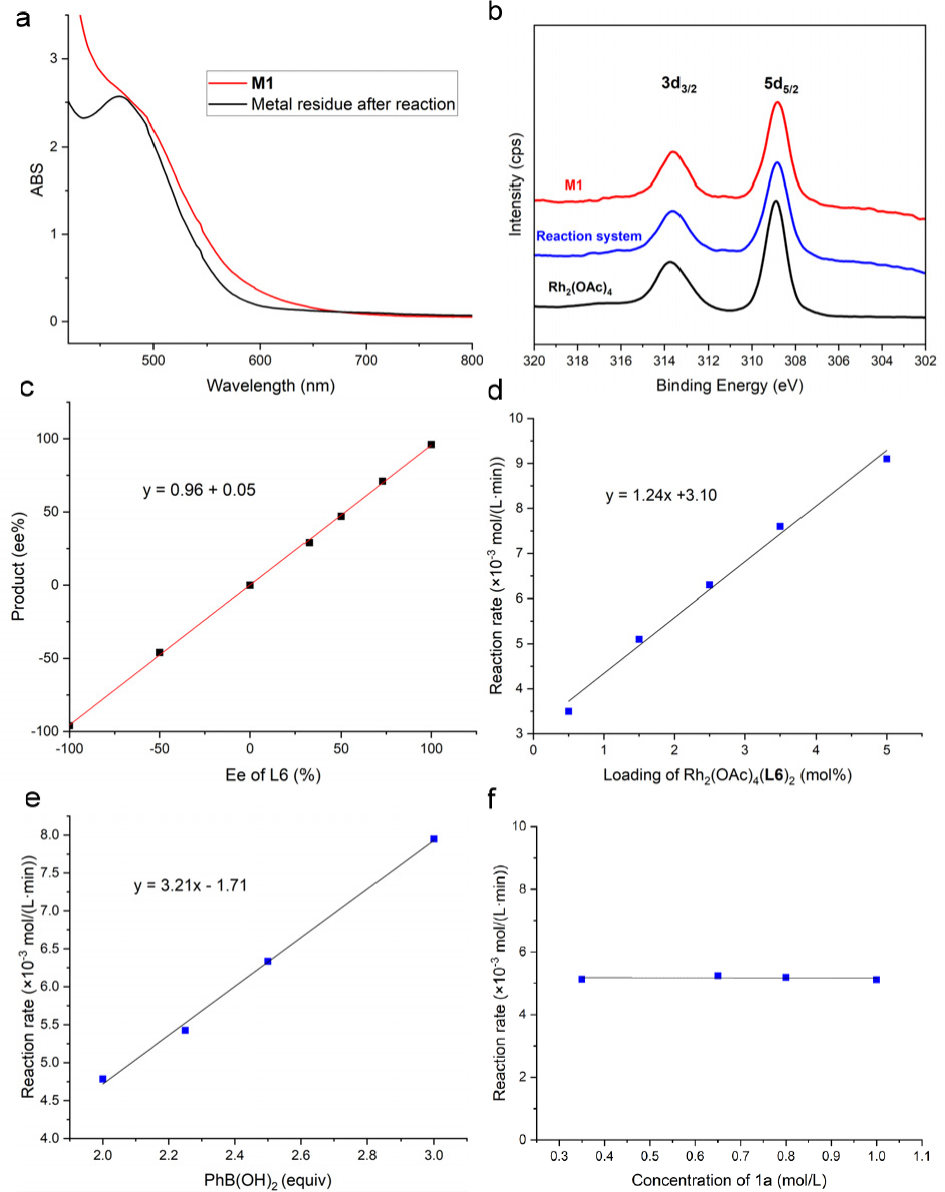
Mechanistic aspects of dirhodium-catalyzed asymmetric arylation. (a) UV–vis absorption spectra in CH_2_Cl_2_ (2 × 10^–3^ M). (b) XPS of Rh_2_(OAc)_4_, **M1**, and reaction mixture. (c) The ee values of 3a versus ee values of **L6**. (d) The reaction order of Rh(OAc)_4_(**L6**)_2_. (e) The reaction order of PhB(OH)_2_. (f) The reaction order of diketone **1a**.

After establishing the two-step sequence for axially chiral biaryl synthesis, we became interested in demonstrating the possible mechanism of this reaction. The coordination of phosphine to Rh_2_(OAc)_4_ was reversible, which was monitored by ^1^H and ^31^P NMR upon gradually increasing the loading of **L6**. Notably, with 1:1 ratio of Rh_2_(OAc)_4_ and **L6**, the reaction seemed to form a mixture of Rh_2_(OAc)_4_(**L6**) and Rh_2_(OAc)_4_(**L6**)_2_, along with a small amount of insoluble Rh_2_(OAc)_4_ (Figure 2). A pair of doublet of doublets signals in ^31^P NMR were observed by the analysis of the metal residue after the reaction. Furthermore, replacing phenyboronic acid with 4-fluorophenyboronic acid would change the chemical shift of these two doublet of doublets peaks (Figure S11), which were assigned to the dirhodium(II) complex **M4** (vide infra). The UV–vis spectra of Rh_2_(OAc)_4_(**L6**)_2_ has a shoulder absorption at ∼500 nm, which is ascribed to the Rh–Rh π* to Rh–Rh σ* HOMO–LUMO transition.^57^ The metal residue after the reaction also displayed an absorption at ∼500 nm. These observations confirm that the dirhodium(II) complex did not dissociate to the monomer (Figure 3a). The oxidation state of the rhodium catalyst was still +2 based on the X-ray photoelectron spectroscopy (XPS) analysis of the reaction mixture (Figure 3b). It was found that the ee value of **3a** was perfectly linear with the optical purity of **L6**, which possible indicated the catalytically active Rh complex binding with only one molecule of **L6** (Figure 3c). Kinetic analyses were performed to learn the orders in the dirhodium(II) catalyst and each reagent. All the reactions displayed a short induction time (see the Supporting Information for details), and Rh_2_(OAc)_4_(**L6**)_2_ and phenylboronic acid **2a** displayed first-order kinetics versus the reaction rate (Figure 3d,e). It was interesting to observe that **1a** showed a good zeroth-order kinetics, indicating **1a** did not involve in the rate-determining step (Figure 3f). These kinetic behaviors are different from the observations in Gois’s catalytic system,^9^ where a concerted three-component interaction model of dirhodium(II)–(phenylboronic acid)–(aldehyde) was proposed.

**Figure 4:**
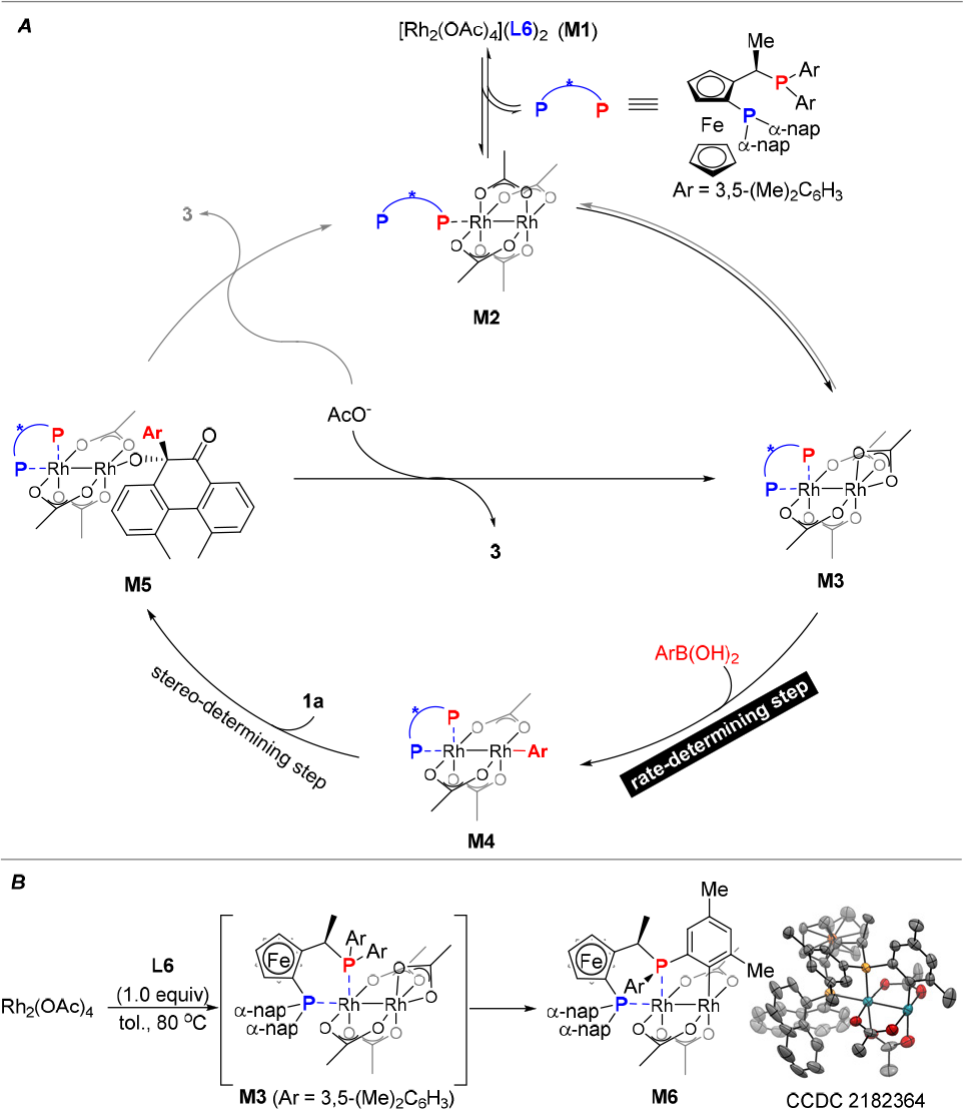
Possible rationale of dirhodium(II)-catalyzed arylation.

On the basis of the above studies and previous reports by other groups,^58^ we tentatively proposed a pathway for the Rh_2_(OAc)_4_(**L6**)_2_ catalyzed arylation reaction (Figure 4A). The coordination of **L6** with dirhodium(II) species is a kinetic process, where complex **M1** releases one molecule of **L6** to form **M2**. The second arm of the phosphine ligand in **M2** coordinated to the Rh atom to push one of the bridging acetates to fully bind to the other Rh atom (**M3**), which was the possible process causing a short period of induction time in kinetic studies (Figure S9). This coordination mode of acetate with dirhodium(II) complexes has been observed by Gois and Li.^9,59^ Luckily, an independent reaction of Rh_2_(OAc)_4_ (1.0 equiv) and **L6** (1.0 equiv) in the absence of aryl boronic acid and diketone in toluene at 80 °C gave C–H metallic rhodium complex **M6** (Figure 4B), which is collateral evidence of the above coordination mode. The formation of dirhodium-aryl species, i.e., **M4**, via transmetalation of **M3** with arylboronic acid had been previously studied by Doyle and co-workers.^12^ Transmetalation is considered as the rate-determining step, which is consistent with the kinetic behaviors observed in Figure 3d–f. Subsequently, stereoselective carbonyl addition delivered the oxyrhodium **M5**. Finally, releasing the alkoxyl group with the assistance of the acetate anion would regenerate either **M2** or **M3** rhodium catalyst to complete the catalytic cycle.

**Figure 5:**
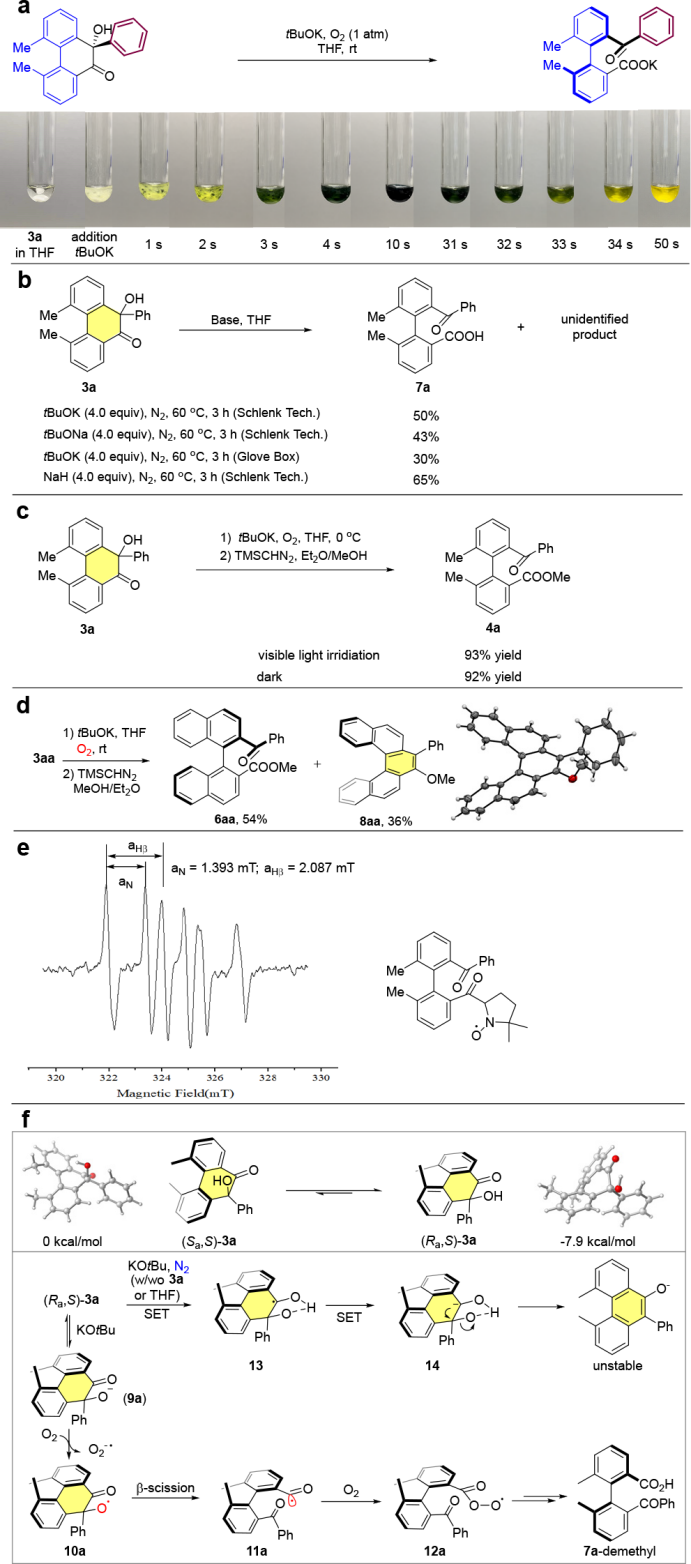
Mechanistic aspects of oxidative ring-opening.

It was observed that the colorless solution of **3a** in THF quickly changed to deep blue upon the addition of *t*BuOK over several seconds. The deep blue color would last for around 30 s, and the color then turned to light yellow, which indicates the end point of the ring-opening reaction (Figure 5a). To elucidate the possible rationale of the base-promoted oxidative ring-opening reaction, a couple of control experiments were conducted (Figure 5b). A standard Schlenk technique with N_2_ atmosphere, *t*BuOK, or NaH (4.0 equiv) still promoted the ring-opening reaction to deliver **7a** in decent yield at 60 °C, along with 7–15% of unknown compound (based on the mass isolated). *t*BuONa resulted in a slightly reduced yield. Even in a glovebox, compound **7a** was still formed, albeit in a relatively lower yield. These results indicated that a trace amount of O_2_ still could promote this reaction upon heating. The oxidative ring-opening reaction is not affected by light: excellent yields can be obtained either under visible light irridiation or under dark conditions (Figure 5c). The oxidative ring-opening of **3aa** followed by methylation gave **6aa** in 54% yield, along with **8aa** (CCDC 2095231) in 36% yield (Figure 5d). The addition of TEMPO dramatically suppressed the reaction, and the corresponding TEMPO adduct was confirmed by HRMS (see the Supporting Information). Moreover, a prominent EPR signal was observed by a spin trapping experiment with DMPO, whose hyperfine splitting (hfs) constants (*a*_N_ = 1.393 mT; *a*_Hβ_ = 2.087 mT) well match the classic acyl-DMPO radicals (Figure 5e).

After the analysis of the configuration and conformation of crystal structures of **3aa** and **4b**, a stereochemical model for the transfer of chirality was proposed (Figure 5f) . It is calculated that (*S*_a_,*S*)-**3a** was thermodynamically 7.9 kcal/mol less stable than (*R*_a_,*S*)-**3a**,^60^ whose anion species **9a** is readily oxidized by O_2_ to deliver **10a**. β-Scission of oxygen-centered radical **10a** gave a more stable acyl radical **11a**,^61−64^ which reacted with oxygen, followed by peroxide radical degradation, to produce the axially chiral carboxylic acid. Under nitrogen atmosphere, reduction of (*R*_a_,*S*)-**3a** via a single electron transfer (SET) process afforded **13**.^65−67^ The second SET reduction, followed by elimination of hydroxide anion, gave the side product phenol.

## Conclusions

We have developed a dirhodium(II)-catalyzed asymmetric arylation of 4,5-disubstituted phenanthrene-9,10-diones, which demonstrated the first chiral phosphine ligand controlled dirhodium(II)-catalyzed highly enantioselective carbonyl addition reaction. Kinetic analysis revealed that the rate-determining step involved dirhodium catalyst and arylboronic acid, while the phenanthrene-9,10-dione is zeroth-order for this reaction. Moreover, axially chiral biaryls could be efficiently prepared through a base-promoted oxidative ring-opening reaction of optically active α-hydroxyl ketones. The process conceived a radical type β-scission reaction, which had a high degree of central-to-axial chirality transfer. A notable feature of this reaction is that the radical was initiated under very mild conditions without adding extra metal oxidant.

## Supporting Information Available

Experimental procedures, characterization of products, and spectroscopic data (PDF)
Crystallographic data for **3aa** (CIF)
Crystallographic data for **4b** (CIF)
Crystallographic data for **8aa** (CIF)
Crystallographic data for **M6** (CIF)
Crystallographic data for **S26** (CIF)

## Supplementary Material

oc2c01207_si_001.pdf

oc2c01207_si_002.cif

oc2c01207_si_003.cif

oc2c01207_si_004.cif

oc2c01207_si_005.cif

oc2c01207_si_006.cif

## References

[ref1] Davies H. M. L.; Hutcheson D. K. Enantioselective Synthesis of Vinylcyclopropanes by Rhodium(II) Catalyzed Decomposition of Vinyldiazomethanes in the Presence of Alkenes. Tetrahedron Lett. 1993, 34, 7243–7246. 10.1016/S0040-4039(00)79298-7.

[ref2] Doyle M. P.; Forbes D. C. Recent Advances in Asymmetric Catalytic Metal Carbene Transformations. Chem. Rev. 1998, 98, 911–936. 10.1021/cr940066a. 11848918

[ref3] Breslow R.; Gellman S. H. Intramolecular Nitrene Carbon-Hydrogen Insertions Mediated by Transition-Metal Complexes as Nitrogen Analogs of Cytochrome P-450 Reactions. J. Am. Chem. Soc. 1983, 105, 6728–6729. 10.1021/ja00360a039.

[ref4] Liao K.; Pickel T. C.; Boyarskikh V.; Bacsa J.; Musaev D. G.; Davies H. M. L. Site-selective and stereoselective functionalization of non-activated tertiary C–H bonds. Nature 2017, 551, 609–613. 10.1038/nature24641. 29156454

[ref5] Roizen J. L.; Harvey M. E.; Du Bois J. Metal-Catalyzed Nitrogen-Atom Transfer Methods for the Oxidation of Aliphatic C–H Bonds. Acc. Chem. Res. 2012, 45, 911–922. 10.1021/ar200318q. 22546004 PMC5483381

[ref6] Müller P.; Fruit C. Enantioselective Catalytic Aziridinations and Asymmetric Nitrene Insertions into CH Bonds. Chem. Rev. 2003, 103, 2905–2920. 10.1021/cr020043t. 12914485

[ref7] Zhang M.; Tang W. Synthesis of Functionalized Cyclohexenone Core of Welwitindolinones via Rhodium-Catalyzed [5 + 1] Cycloaddition. Org. Lett. 2012, 14, 3756–3759. 10.1021/ol301614v. 22783971 PMC3480994

[ref8] Fürstner A.; Krause H. Practical Method for the Rhodium-Catalyzed Addition of Aryl- and Alkenylboronic Acids to Aldehydes. Adv. Synth. Catal. 2001, 343, 343–350. 10.1002/1615-4169(20010430)343:4<343::AID-ADSC343>3.0.CO;2-Z.

[ref9] Trindade A. F.; Coelho J. A. S.; Afonso C. A. M.; Veiros L. F.; Gois P. M. P. Fine Tuning of Dirhodium(II) Complexes: Exploring the Axial Modification. ACS Catal. 2012, 2, 370–383. 10.1021/cs200597a.

[ref10] Snyder J. P.; Padwa A.; Stengel T.; et al. A Stable Dirhodium Tetracarboxylate Carbenoid: Crystal Structure, Bonding Analysis, and Catalysis. J. Am. Chem. Soc. 2001, 123, 11318–11319. 10.1021/ja016928o. 11697986

[ref11] Gois P. M. P.; Trindade A. F.; Veiros L. F.; André V.; Duarte M. T.; Afonso C. A. M.; Caddick S.; Cloke F. G. N. Tuning the Reactivity of Dirhodium(II) Complexes with Axial N-Heterocyclic Carbene Ligands: The Arylation of Aldehydes. Angew. Chem., Int. Ed. 2007, 46, 5750–5753. 10.1002/anie.200700924. 17591740

[ref12] Doyle M. P.; Shabashov D.; Zhou L.; Zavalij P. Y.; Welch C.; Pirzada Z. Does an Axial Propeller Shape on a Dirhodium(III,III) Core Affect Equatorial Ligand Chirality?. Organometallics 2011, 30, 3619–3627. 10.1021/om2003078.

[ref13] Tan J.; Kuang Y.; Wang Y.; Huang Q.; Zhu J.; Wang Y. Axial Tri-tert-butylphosphane Coordination to Rh_2_(OAc)_4_: Synthesis, Structure, and Catalytic Studies. Organometallics 2016, 35, 3139–3147. 10.1021/acs.organomet.6b00477.

[ref14] Collins L. R.; van Gastel M.; Neese F.; Fürstner A. Enhanced Electrophilicity of Heterobimetallic Bi–Rh Paddlewheel Carbene Complexes: A Combined Experimental, Spectroscopic, and Computational Study. J. Am. Chem. Soc. 2018, 140, 13042–13055. 10.1021/jacs.8b08384. 30217113

[ref15] Ma Z.; Wang Y. Dirhodium(II)/P(t-Bu)_3_ Catalyzed Tandem Reaction of α,β-Unsaturated Aldehydes with Arylboronic Acids. Org. Biomol. Chem. 2018, 16, 7470–7476. 10.1039/C8OB01997E. 30270369

[ref16] Cressy D.; Zavala C.; Abshire A.; Sheffield W.; Darko A. Tuning Rh(II)-Catalysed Cyclopropanation with Tethered Thioether Ligands. Dalton Trans. 2020, 49, 15779–15787. 10.1039/D0DT03019H. 33146649

[ref17] Focken T.; Rudolph J.; Bolm C. Planar Chiral Imidazolium Salts in the Asymmetric Rhodium-Catalyzed 1,2-Addition of Arylboronic Acids to Aldehydes. Synthesis 2005, 2005 (3), 429–436. 10.1055/s-2005-861800.

[ref18] Ma Q.; Ma Y.; Liu X.; Duan W.; Qu B.; Song C. Planar Chiral Imidazolium Salts Based on [2.2]Paracyclophane in the Asymmetric Rhodium-Catalyzed 1,2-Addition of Arylboronic Acids to Aldehydes. Tetrahedron: Asymmetry. 2010, 21, 292–298. 10.1016/j.tetasy.2010.01.025.

[ref19] Duan W.; Ma Y.; Qu B.; Zhao L.; Chen J.; Song C. Synthesis of New Alkoxy/Sulfonate-Substituted Carbene Precursors Derived from [2.2]Paracyclophane and Their Application in the Asymmetric Arylation of Aldehydes. Tetrahedron: Asymmetry. 2012, 23, 1369–1375. 10.1016/j.tetasy.2012.09.001.

[ref20] He W.-P.; Zhou B.-H.; Zhou Y.-L.; Li X.-R.; Fan L.-M.; Shou H.-W.; Li J. Synthesis of New Benzimidazolium Salts and Their Application in the Asymmetric Arylation of Aldehydes. Tetrahedron Lett. 2016, 57, 3152–3155. 10.1016/j.tetlet.2016.06.023.

[ref21] Toullec P. Y.; Jagt R. B. C.; de Vries J. G.; Feringa B. L.; Minnaard A. J. Rhodium-Catalyzed Addition of Arylboronic Acids to Isatins: An Entry to Diversity in 3-Aryl-3-Hydroxyoxindoles. Org. Lett. 2006, 8, 2715–2718. 10.1021/ol0608101. 16774239

[ref22] Shintani R.; Inoue M.; Hayashi T. Rhodium-Catalyzed Asymmetric Addition of Aryl- and Alkenylboronic Acids to Isatins. Angew. Chem., Int. Ed. 2006, 45, 3353–3356. 10.1002/anie.200600392. 16596682

[ref23] Duan H.-F.; Xie J.-H.; Qiao X.-C.; Wang L.-X.; Zhou Q.-L. Enantioselective Rhodium-Catalyzed Addition of Arylboronic Acids to α-Ketoesters. Angew. Chem., Int. Ed. 2008, 47, 4351–4353. 10.1002/anie.200800423. 18442151

[ref24] Facchetti S.; Cavallini I.; Funaioli T.; Marchetti F.; Iuliano A. Tropos Ligands in Asymmetric Rhodium(I)-Catalyzed Addition of Arylboronic Acids to Enones: How a Tunable Coordination Gives Different Reaction Products. Organometallics. 2009, 28, 4150–4158. 10.1021/om900306s.

[ref25] Cai F.; Pu X.; Qi X.; Lynch V.; Radha A.; Ready J. M. Chiral Allene-Containing Phosphines in Asymmetric Catalysis. J. Am. Chem. Soc. 2011, 133, 18066–18069. 10.1021/ja207748r. 21972824 PMC3216402

[ref26] Feng X.; Nie Y.; Yang J.; Du H. Rh(I)-Catalyzed Asymmetric 1,2-Addition to α-Diketones with Chiral Sulfur–Alkene Hybrid Ligands. Org. Lett. 2012, 14, 624–627. 10.1021/ol203238j. 22233270

[ref27] Zhu T.-S.; Jin S.-S.; Xu M.-H. Rhodium-Catalyzed, Highly Enantioselective 1,2-Addition of Aryl Boronic Acids to α-Ketoesters and α-Diketones Using Simple, Chiral Sulfur–Olefin Ligands. Angew. Chem., Int. Ed. 2012, 51, 780–783. 10.1002/anie.201106972. 22134900

[ref28] Zhang Z.-F.; Zhu D.-X.; Chen W.-W.; Xu B.; Xu M.-H. Enantioselective Synthesis of gem-Diaryl Benzofuran-3(2H)-ones via One-Pot Asymmetric Rhodium/Palladium Relay Catalysis. Org. Lett. 2017, 19, 2726–2729. 10.1021/acs.orglett.7b01070. 28485148

[ref29] Wencel-Delord J.; Panossian A.; Leroux F. R.; Colobert F. Recent advances and new concepts for the synthesis of axially stereoenriched biaryls. Chem. Soc. Rev. 2015, 44, 3418–3430. 10.1039/C5CS00012B. 25904287

[ref30] Kumarasamy E.; Raghunathan R.; Sibi M. P.; Sivaguru J. Nonbiaryl and Heterobiaryl Atropisomers: Molecular Templates with Promise for Atropselective Chemical Transformations. Chem. Rev. 2015, 115, 11239–11300. 10.1021/acs.chemrev.5b00136. 26414162

[ref31] Link A.; Sparr C. Stereoselective arene formation. Chem. Soc. Rev. 2018, 47, 3804–3815. 10.1039/C7CS00875A. 29565066

[ref32] Bao X.; Rodriguez J.; Bonne D. Enantioselective Synthesis of Atropisomers with Multiple Stereogenic Axes. Angew. Chem., Int. Ed. 2020, 59, 12623–12634. 10.1002/anie.202002518. 32202361

[ref33] Carmona J. A.; Rodríguez-Franco C.; Fernández R.; Hornillos V.; Lassaletta J. M. Atroposelective transformation of axially chiral (hetero)biaryls. From desymmetrization to modern resolution strategies. Chem. Soc. Rev. 2021, 50, 2968–2983. 10.1039/D0CS00870B. 33491680

[ref34] Liu C.-X.; Zhang W.-W.; Yin S.-Y.; Gu Q.; You S.-L. Synthesis of Atropisomers by Transition-Metal-Catalyzed Asymmetric C-H Functionalization Reactions. J. Am. Chem. Soc. 2021, 143, 14025–14040. 10.1021/jacs.1c07635. 34432467

[ref35] Cheng J. K.; Xiang S.-H.; Li S.; Ye L.; Tan B. Recent Advances in Catalytic Asymmetric Construction of Atropisomers. Chem. Rev. 2021, 121, 4805–4902. 10.1021/acs.chemrev.0c01306. 33775097

[ref36] Liu Z.-S.; Xie P.-P.; Hua Y.; Wu C.; Ma Y.; Chen J.; Cheng H.-G.; Hong X.; Zhou Q. An Axial to Axial Chirality Transfer Strategy for Atroposelective Construction of C-N Axial Chirality. Chem. 2021, 7, 1917–1932. 10.1016/j.chempr.2021.04.005.

[ref37] Liao G.; Zhou T.; Yao Q.-J.; Shi B.-F. Recent advances in the synthesis of axially chiral biaryls via transition metal-catalysed asymmetric C–H functionalization. Chem. Commun. 2019, 55, 8514–8523. 10.1039/C9CC03967H. 31276136

[ref38] Bringmann G.; Reuscher H. Atropdiastereoselective Ring Opening of Bridged, “Axial-Prostereogenic” Biaryls: Directed Synthesis of (+)-Ancistrocladisine. Angew. Chem., Int. Ed. Engl. 1989, 28, 1672–1673. 10.1002/anie.198916721.

[ref39] Bringmann G.; Menche D. Stereoselective Total Synthesis of Axially Chiral Natural Products via Biaryl Lactones. Acc. Chem. Res. 2001, 34, 615–624. 10.1021/ar000106z. 11513568

[ref40] Shimada T.; Cho Y. H.; Hayashi T. Nickel-catalyzed Asymmetric Grignard Cross-Coupling of Dinaphthothiophene Giving Axially Chiral 1,1’-Binaphthyls. J. Am. Chem. Soc. 2002, 124, 13396–13397. 10.1021/ja0282588. 12418887

[ref41] Yu C.; Huang H.; Li X.; Zhang Y.; Wang W. Dynamic Kinetic Resolution of Biaryl Lactones via a Chiral Bifunctional Amine Thiourea-Catalyzed Highly Atropo-Enantioselective Transesterification. J. Am. Chem. Soc. 2016, 138, 6956–6959. 10.1021/jacs.6b03609. 27218264

[ref42] Zhao K.; Duan L.; Xu S.; Jiang J.; Fu Y.; Gu Z. Enhanced Reactivity by Torsional Strain of Cyclic Diaryliodonium in Cu-Catalyzed Enantioselective Ring-Opening Reaction. Chem. 2018, 4, 599–612. 10.1016/j.chempr.2018.01.017.

[ref43] Chen G.-Q.; Lin B.-J.; Huang J.-M.; Zhao L.-Y.; Chen Q.-S.; Jia S.-P.; Yin Q.; Zhang X. Design and Synthesis of Chiral Oxa-Spirocyclic Ligands for Ir-Catalyzed Direct Asymmetric Reduction of Bringmann’s Lactones with Molecular H_2_. J. Am. Chem. Soc. 2018, 140, 8064–8068. 10.1021/jacs.8b03642. 29920089

[ref44] Deng R.; Xi J.; Li Q.; Gu Z. Enantioselective Carbon-Carbon Bond Cleavage for Biaryl Atropisomers Synthesis. Chem. 2019, 5, 1834–1846. 10.1016/j.chempr.2019.04.008.

[ref45] Wang G.; Shi Q.; Hu W.; Chen T.; Guo Y.; Hu Z.; Gong M.; Guo J.; Fu Z.; Huang W.; et al. Organocatalytic Asymmetric N-Sulfonyl Amide C-N Bond Activation to Access Axially Chiral Biaryl Amino Acids. Nat. Commun. 2020, 11, 946 10.1038/s41467-020-14799-8. 32075976 PMC7031291

[ref46] Feng J.; Bi X.; Xue X.; Li N.; Shi L.; Gu Z. Catalytic Asymmetric C–Si Bond Activation via Torsional Strain-Promoted Rh-Catalyzed Aryl Narasaka Acylation. Nat. Commun. 2020, 11, 4449 10.1038/s41467-020-18273-3. 32895390 PMC7477585

[ref47] Guo F.; Konkol L. C.; Thomson R. J. Enantioselective Synthesis of Biphenols from 1,4-Diketones by Traceless Central-to-Axial Chirality Exchange. J. Am. Chem. Soc. 2011, 133, 18–20. 10.1021/ja108717r. 21141997 PMC3073418

[ref48] He X.-L.; Zhao H.-R.; Song X.; Jiang B.; Du W.; Chen Y.-C. Asymmetric Barton-Zard Reaction to Access 3-Pyrrole-Containing Axially Chiral Skeletons. ACS Catal. 2019, 9, 4374–4381. 10.1021/acscatal.9b00767.

[ref49] Quinonero O.; Jean M.; Vanthuyne N.; Roussel C.; Bonne D.; Constantieux T.; Bressy C.; Bugaut X.; Rodriguez J. Combining Organocatalysis with Central-to-Axial Chirality Conversion: Atroposelective Hantzsch-Type Synthesis of 4-Arylpyridines. Angew. Chem., Int. Ed. 2016, 55, 1401–1405. 10.1002/anie.201509967. 26662927

[ref50] Wang Y.-B.; Zheng S.-C.; Hu Y.-M.; Tan B. Bro̷nsted Acid-Catalysed Enantioselective Construction of Axially Chiral Arylquina-zolinones. Nat. Commun. 2017, 8, 15489 10.1038/ncomms15489. 28524863 PMC5454535

[ref51] Zhu S.; Chen Y.-H.; Wang Y.-B.; Yu P.; Li S.-Y.; Xiang S.-H.; Wang J.-Q.; Xiao J.; Tan B. Organocatalytic Atroposelective Construction of Axially Chiral Arylquinones. Nat. Commun. 2019, 10, 4268 10.1038/s41467-019-12269-4. 31537811 PMC6753127

[ref52] Raut V. S.; Jean M.; Vanthuyne N.; Roussel C.; Constantieux T.; Bressy C.; Bugaut X.; Bonne D.; Rodriguez J. Enantioselective Syntheses of Furan Atropisomers by an Oxidative Central-to-Axial Chirality Conversion Strategy. J. Am. Chem. Soc. 2017, 139, 2140–2143. 10.1021/jacs.6b11079. 28106391

[ref53] Bisag G. D.; Pecorari D.; Mazzanti A.; Bernardi L.; Fochi M.; Bencivenni G.; Bertuzzi G.; Corti V. Central-to-Axial Chirality Conversion Approach Designed on Organocatalytic Enantioselective Povarov Cycloadditions: First Access to Configurationally Stable Indol-Quinoline Atropisomers. Chem.—Eur. J. 2019, 25, 15694–15701. 10.1002/chem.201904213. 31556176

[ref54] Xu Y.; Yan G.; Ren Z.; Dong G. A Unified Strategy for Diverse Functionalization at Alcohol β-Position. Nat. Chem. 2015, 7, 829–834. 10.1038/nchem.2326. 26391083

[ref55] Thompson S. J.; Thach D. Q.; Dong G. Cyclic Ether Synthesis via Palladium-catalyzed Alcohol-mediated Dehydrogenative Annulation at Unactivated Terminal Positions. J. Am. Chem. Soc. 2015, 137, 11586–11589. 10.1021/jacs.5b07384. 26322370

[ref56] Hu P.; Kong L.; Wang F.; Zhu X.; Li X. Twofold C–H Activation-Based Enantio- and Diastereoselective C–H Arylation Using Diarylacetylenes as Rare Arylating Reagents. Angew. Chem., Int. Ed. 2021, 60, 20424–20429. 10.1002/anie.202106871. 34145966

[ref57] Warzecha E.; Berto T. C.; Wilkinson C. C.; Berry J. F. Rhodium Rainbow: A Colorful Laboratory Experiment Highlighting Ligand Field Effects of Dirhodium Tetraacetate. J. Chem. Educ. 2019, 96, 571–576. 10.1021/acs.jchemed.6b00648.

[ref58] Hong B.; Shi L.; Li L.; Zhan S.; Gu Z. Paddlewheel dirhodium(II) complexes with N-heterocyclic carbene or phosphine ligand: New reactivity and selectivity. Green Synth. Catal. 2022, 3, 137–149. 10.1016/j.gresc.2022.03.001.

[ref59] Fu L.; Li S.; Cai Z.; Ding Y.; Guo X.-Q.; Zhou L.-P.; Yuan D.; Sun Q.-F.; Li G. Ligand-enabled site-selectivity in a versatile rhodium(ii)-catalysed aryl C-H carboxylation with CO_2_. Nat. Catal. 2018, 1, 469–478. 10.1038/s41929-018-0080-y.

[ref60] Liu Y.; Tse Y.-L.; Kwong F. Y.; Yeung Y.-Y. Accessing Axially Chiral Biaryls via Organocatalytic Enantioselective Dynamic-Kinetic Resolution-Semipinacol Rearrangement. ACS Catal. 2017, 7, 4435–4440. 10.1021/acscatal.7b01056.

[ref61] Bentrude W. G.; Darnall K. R. A Free-Radical Acylation. J. Am. Chem. Soc. 1968, 90, 3588–3589. 10.1021/ja01015a066.

[ref62] Sawaki Y.; Ogata Y. Photolysis of α-Hydroperoxy Ketones. J. Am. Chem. Soc. 1976, 98, 7324–7327. 10.1021/ja00439a036.

[ref63] Tsang A. S.-K.; Kapat A.; Schoenebeck F. Factors That Control C–C Cleavage versus C–H Bond Hydroxylation in Copper-Catalyzed Oxidations of Ketones with O_2_. J. Am. Chem. Soc. 2016, 138, 518–526. 10.1021/jacs.5b08347. 26675262

[ref64] Jia K.; Pan Y.; Chen Y. Selective Carbonyl-C(sp3) Bond Cleavage to Construct Ynamides, Ynoates, and Ynones by Photoredox Catalysis. Angew. Chem., Int. Ed. 2017, 56, 2478–2481. 10.1002/anie.201611897. 28121070

[ref65] Studer A.; Curran D. P. The Electron is a Catalyst. Nat. Chem. 2014, 6, 765–773. 10.1038/nchem.2031. 25143210

[ref66] Dewanji A.; Mück-Lichtenfeld C.; Studer A. Radical Hydrodeiodination of Aryl, Alkenyl, Alkynyl, and Alkyl Iodides with an Alcoholate as Organic Chain Reductant through Electron Catalysis. Angew. Chem., Int. Ed. 2016, 55, 6749–6752. 10.1002/anie.201601930. 27101530

[ref67] Ding T.-H.; Qu J.-P.; Kang Y.-B. Visible-Light-Induced, Base-Promoted Transition-Metal-Free Dehalogenation of Aryl Fluorides, Chlorides, Bromides, and Iodides. Org. Lett. 2020, 22, 3084–308. 10.1021/acs.orglett.0c00827. 32227906

