# Supplementary material for: Dirhodium(II)/Phosphine Catalyst with Chiral Environment at Bridging Site and Its Application in Enantioselective Atropisomer Synthesis
Source: ACS Cent Sci. 2023 Mar 20;9(4):748–55. doi: 10.1021/acscentsci.2c01207 (PMC10141619; doi:10.1021/acscentsci.2c01207)
Supplement: Supplementary file 1 — oc2c01207_si_001.pdf [file oc2c01207_si_001.pdf]

Supporting Information

Dirhodium(II)/Phosphine Catalyst with Chiral Environment at Bridging Site and Its Application in Enantioselective Atropisomer Synthesis

Lei Shi,[1] Xiaoping Xue,[1] Biqiong Hong,[2] Qigang Li[1] and Zhenhua Gu*[1,2]

1. Hefei National Research Center for Physical Sciences at the Microscale and Department of Chemistry, University of Science and Technology of China, 96 Jinzhai Road, Hefei, Anhui 230026, P. R. China

2. College of Materials and Chemical Engineering, Minjiang University, Fuzhou, Fujian, 350108, China

* email: zhgu@ustc.edu.cn

General Information

Nuclear magnetic resonances were recorded on Bruker-400 MHz and Bruker-500 MHz instruments. Reference values for residual solvents were taken as δ = 0.00 ppm (TMS), δ = 7.26 ppm ($CDCl_3$), for ^1H NMR; δ = 77.00 ppm ($CDCl_3$) for ^{13}C NMR. High resolution mass spectral analysis (HRMS) was performed on Waters XEVO G2 Q-TOF.

All reactions were performed under an inert atmosphere of dry nitrogen in flame-dried glassware, unless otherwise stated. Toluene was distilled over calcium hydride under an atmosphere of nitrogen. Tetrahydrofuran and diethyl ether were distilled over sodium in the presence of benzophenone under an atmosphere of nitrogen.

Our initial trials focused on Rh(I)-catalyzed enantioselective arylation. We screened various Rh(I) metal complexes, as well as a vast series of chiral ligands, however, however, no satisfied results were achieved. Typical results were listed in Scheme S1 by using $[Rh(C_2H_4)Cl]_2$ as the catalyst.

Scheme S1. Ligand screening in Rh(I)-catalyzed asymmetric arylation

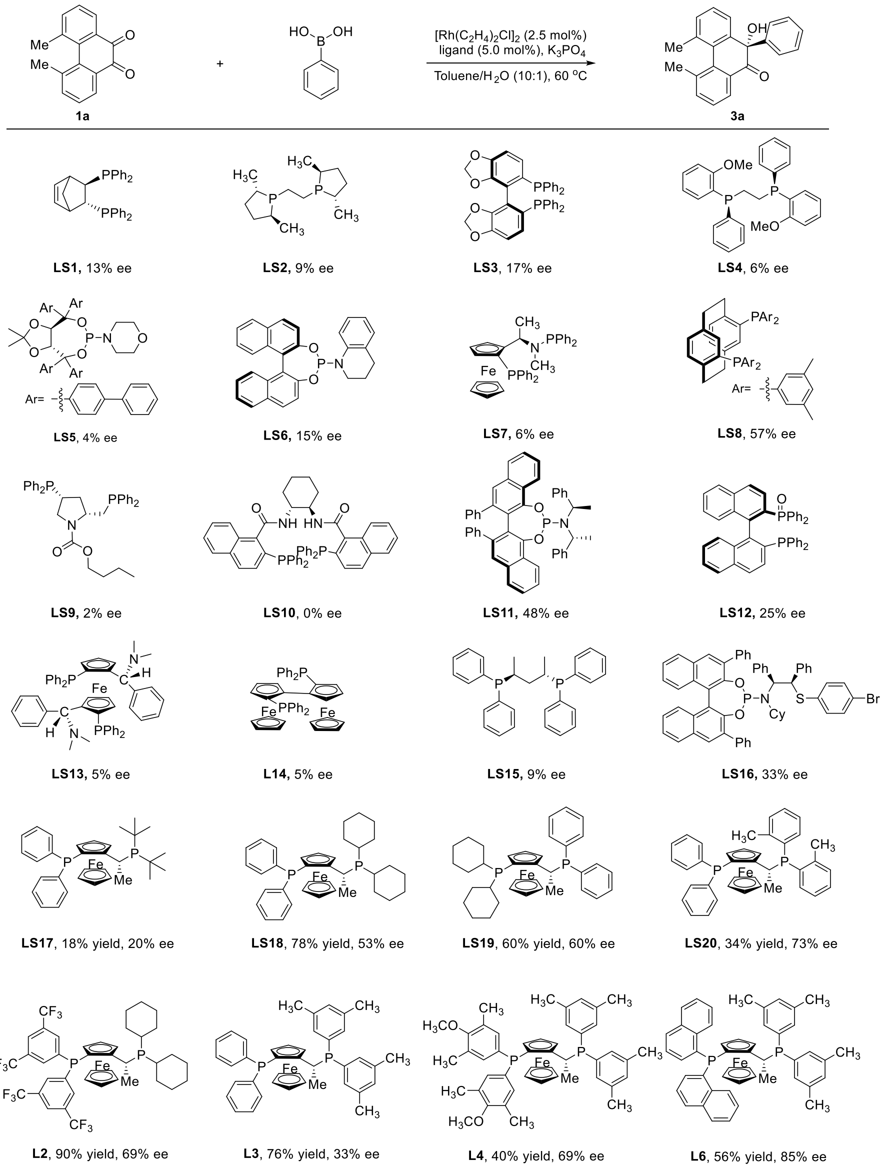

Experimental Procedure and Characteristic Data

Typical Procedure for synthesis of dialdehyde[1,2] (Typical Procedure A)

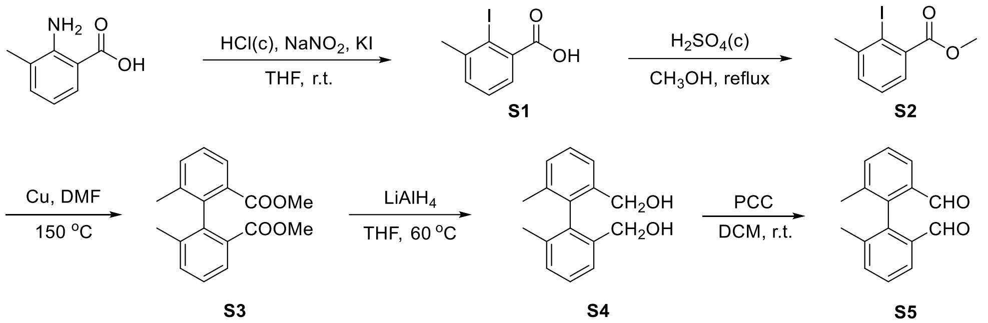

To a mixture of 2-amino-3-methylbenzoic acid (15.12 g, 0.10 mol, 1.0 equiv) and 4 M HCl (250 mL, 1.00 mol, 10.0 equiv) in THF (100 mL) was added an aqueous solution of $NaNO_2$ (1 M, 120 mL, 0.12 mol, 1.2 equiv) dropwise at 0 °C and stirred for 20 min. Aqueous KI (2 M, 125 mL, 0.25 mol, 2.5 equiv) was added dropwise at 0 °C and stirred for 10 min at 0 °C and 5 h at room temperature. The mixture was extracted with ethyl acetate (250 mL×4), and the combined organic layer was washed with H_2O, saturated $NaHSO_3$ solution and brine. The organic phase was dried over anhydrous Na_2SO_4, filtrated and concentrated to afford crude **S1**, which was used in next step without further purification.

A mixture of **S1** and methanol (100 mL) was added concentrated H_2SO_4 (10 mL), then refluxed at 85 °C for 4h. The completion of the reaction was monitored by TLC. The reaction mixture was cooled to rt and the solvent was evaporated. The residue was poured into a separating funnel with cold water and extracted with EtOAc for three times. The combined organic layer was washed with $NaHCO_3$ (aq.) and brine, dried over anhydrous Na_2SO_4, filtered and concentrated to afford crude **S2**, which was used in next step without further purification.

To a solution of **S2** in *N,N*-dimethylformamide (40 mL) was added active copper (12.00 g, 0.19 mol) and the mixture was stirred at 150 °C overnight. After being cooled to room temperature, solid was filtered off and washed with ethyl acetate. The filtrate was thoroughly washed sequentially with water, 1M HCl, $NaHCO_3$ (aq.), brine and dried over anhydrous Na_2SO_4. After filtration the solvent was concentrated in vacuum and the residue was purified by flash column chromatography (PE/EtOAc 90:10) on silica gel to afford the desired product **S3** (13.28 g, 89% for three steps) as a colorless oil.[2] **^{1}H NMR** (500 MHz, $CDCl_3$) δ 7.85 (d, *J* = 7.7 Hz, 2H), 7.43 (d, *J* = 7.5 Hz, 2H), 7.36 – 7.30 (m, 2H), 3.58 (s, 6H), 1.91 (s, 6H).

To a mixture of $LiAlH_4$ (3.91 g, 103 mmol, 2.3 equiv) in THF (60 mL) was added a solution of **S3** (13.28 g, 44.5 mmol) in THF (60 mL) very carefully at 0 °C. After the addition, the reaction was allowed to warm to 60 °C and stirred for 3 h. The mixture was cooled to 0 °C and aqueous potassium sodium tartrate was added with a vigorous stirring for 20 min. The mixture was diluted with water and extracted with EtOAc for three times. The combined organic layer was washed with brine, dried over anhydrous Na_2SO_4, filtered and concentrated to afford crude **S4**, which was directly used in next step.

To a mixture of PCC (CrO_3·Pydidine·HCl) (28.02 g, 130 mmol) in dry CH_2Cl_2 (100 mL) was added a solution of **S4** in dry CH_2Cl_2 (100 mL). The mixture was stirred vigorously for 4 h at room temperature. The resulting solution was passed through a plug of silica gel. The plug was washed

with CH_2Cl_2 and the filtrate was concentrated in vacuum and purified by flash column chromatography (PE/EtOAc 95:5) on silica gel to afford the desired product **S5** (9.23 g, 87% for two steps) as a white solid.[2] **^{1}H NMR** (400 MHz, $CDCl_3$) δ 9.60 (s, 2H), 7.91 (d, *J* = 6.8 Hz, 2H), 7.67 – 7.55 (m, 2H), 7.54 – 7.45 (m, 2H), 1.97 (s, 6H).

Compound **S10** was prepared following the **Typical Procedure A**

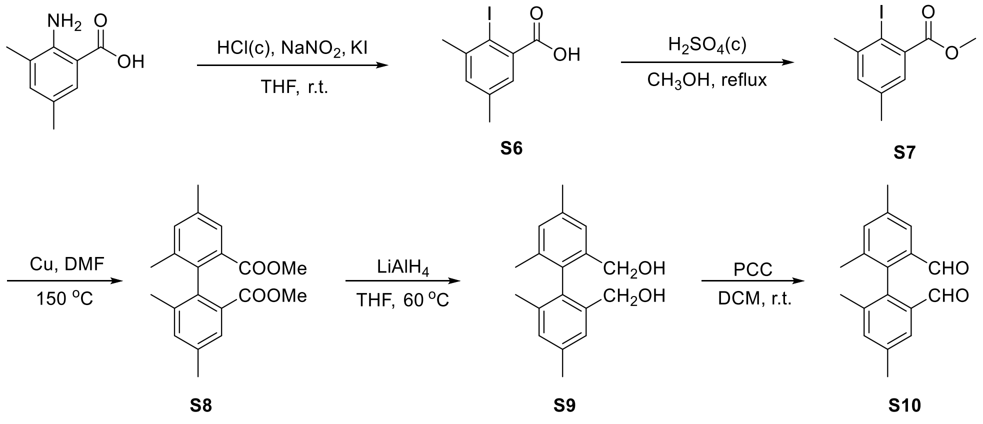

The reaction of 2-amino-3,5-dimethylbenzoic acid (9.91 g, 60.0 mmol, 1.0 equiv), 4 M HCl (150 mL, 0.60 mol, 10 equiv), aqueous $NaNO_2$ (1 M, 72 mL, 72.0 mmol, 1.2 equiv) and aqueous KI (2 M, 75 mL, 0.15 mol, 2.5 equiv) afforded crude product **S6**, which was used in next step without further purification.

The reaction of crude **S6**, MeOH (60 mL) and concentrated H_2SO_4 (6 mL) afforded crude product **S7**, which was used in next step without further purification.

The reaction of crude **S7** and active copper (7.24 g, 114 mmol) afforded the desired product **S8** (8.68 g, 89% for three steps) as a white solid.[3] **^{1}H NMR** (500 MHz, $CDCl_3$) δ 7.66 (s, 2H), 7.25 (s, 2H), 3.59 (s, 6H), 2.39 (s, 6H), 1.87 (s, 6H).

The reaction of **S8** (8.68 g, 26.6 mmol) and $LiAlH_4$ (2.32 g, 61.2 mmol, 2.3 equiv) afforded crude product **S9**, which was directly used in next step.

The reaction of **S9** and PCC (17.20 g, 79.8 mmol) afforded the desired product **S10** (6.34 g, 89% for two steps) as a white solid.[4] **^{1}H NMR** (500 MHz, $CDCl_3$) δ 9.56 (s, 2H), 7.69 (s, 2H), 7.38 (s, 2H), 2.43 (s, 6H), 1.92 (s, 6H).

Compound **S15** was prepared following the **Typical Procedure A**

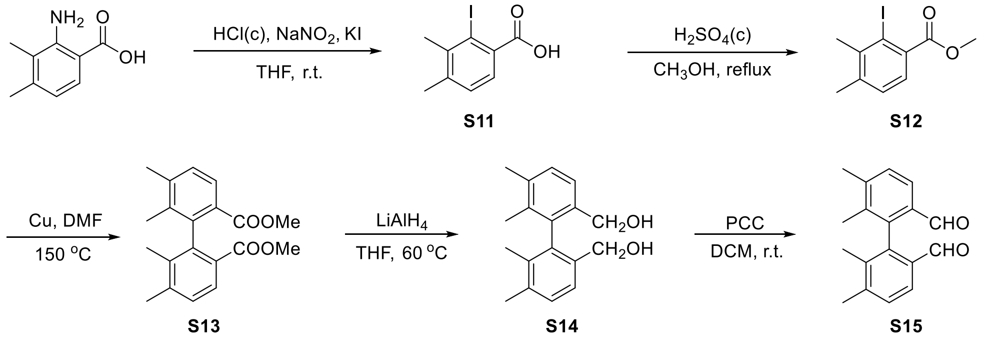

The reaction of 2-amino-3,4-dimethylbenzoic acid (9.91 g, 60.0 mmol, 1.0 equiv), 4 M HCl (150

mL, 0.60 mol, 10 equiv), aqueous $NaNO_2$ (1 M, 72 mL, 72.0 mmol, 1.2 equiv) and aqueous KI (2 M, 75 mL, 0.15 mol, 2.5 equiv) afforded crude product **S11**, which was used in next step without further purification.

The reaction of crude **S11**, MeOH (60 mL) and concentrated H_2SO_4 (6 mL) afforded crude product **S12**, which was used in next step without further purification.

The reaction of crude **S12** and active copper (7.24 g, 114 mmol) afforded the desired product **S13** (8.51 g, 87% for three steps) as a white solid.[3] **^{1}H NMR** (500 MHz, $CDCl_3$) δ 7.77 (d, *J* = 8.0 Hz, 2H), 7.21 (d, *J* = 8.0 Hz, 2H), 3.56 (s, 6H), 2.36 (s, 6H), 1.79 (s, 6H).

The reaction of **S13** (8.51 g, 26.1 mmol) and $LiAlH_4$ (2.28 g, 60.0 mmol, 2.3 equiv) afforded crude product **S14**, which was directly used in next step.

The reaction of **S14** and PCC (16.90 g, 78.3 mmol) afforded the desired product **S15** (5.84 g, 84% for two steps) as a white solid. **^{1}H NMR** (500 MHz, $CDCl_3$) δ 7.49 – 7.43 (m, 2H), 7.82 (d, *J* = 8.0 Hz, 2H), 7.37 (d, *J* = 8.0 Hz, 2H), 2.42 (s, 6H), 1.87 (s, 6H). **^{13}C NMR** (126 MHz, $CDCl_3$) δ 191.4, 144.4, 140.5, 136.1, 133.0, 130.1, 125.7, 21.2, 16.1. HRMS (ESI) calcd for $C_{18}H_{19}O_2$ $[M+H]^+$ 267.1385, found 267.1387.

Compound **S20** was prepared following the **Typical Procedure A**

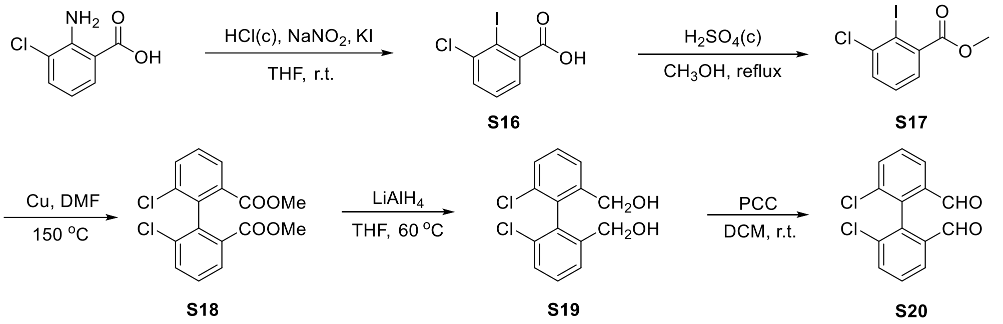

The reaction of 2-amino-3-chlorobenzoic acid (10.29 g, 60.0 mmol, 1.0 equiv), 4 M HCl (150 mL, 0.60 mol, 10 equiv), aqueous $NaNO_2$ (1 M, 72 mL, 72.0 mmol, 1.2 equiv) and aqueous KI (2 M, 75 mL, 0.15 mol, 2.5 equiv) afforded crude product **S16**, which was used in next step without further purification.

The reaction of crude **S16**, MeOH (60 mL) and concentrated H_2SO_4 (6 mL) afforded crude product **S17**, which was used in next step without further purification.

The reaction of crude **S17** and active copper (7.24 g, 114 mmol) afforded the desired product **S18**[5] (6.82 g, 67% for three steps) as a white solid. **^{1}H NMR** (500 MHz, $CDCl_3$) δ 8.03 (d, *J* = 7.9 Hz, 2H), 7.67 (d, *J* = 8.0 Hz, 2H), 7.47 – 7.39 (m, 2H), 3.65 (s, 6H).

The reaction of **S18** (6.82 g, 20.1 mmol) and $LiAlH_4$ (1.75 g, 46.2 mmol, 2.3 equiv) afforded crude product **S19**, which was used directly used in next step.

The reaction of **S19** and PCC (13.00 g, 60.3 mmol) afforded the desired product **S20** (3.91 g, 70% for two steps) as a white solid. **^{1}H NMR** (500 MHz, CDCl3) δ 9.64 (s, 2H), 7.97 (d, *J* = 7.5 Hz, 2H), 7.77 (d, *J* = 8.0 Hz, 2H), 7.65 – 7.55 (m, 2H). **^{13}C NMR** (126 MHz, $CDCl_3$) δ 189.5, 136.4, 136.0, 135.1, 134.8, 130.3, 128.1. HRMS (ESI) calcd for $C_{14}H_8Cl_2O_2Na$ $[M+Na]^+$ 300.9799, found 300.9804.

Typical Procedure for synthesis of diiodides[6] (Typical Procedure B)

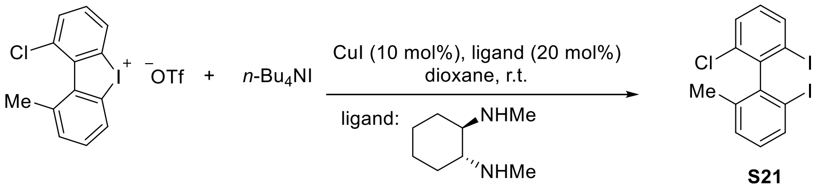

S21

A mixture of 1-chloro-9-methyl cyclic diaryliodonium[7] (5.38 g, 11.3 mmol, 1.0 equiv), n-Bu$_4$NI (8.35 g, 22.6 mmol, 2.0 equiv), CuI (215 mg, 1.13 mmol, 10 mol%), *trans*-N,N'-dimethylcyclohexane-1,2-diamine (321 mg, 2.26 mmol, 20 mol%) in dioxane (50 mL) was stirred at room temperature for 24 hours. After complete consumption of starting material, the mixture was filtered through Celite and the filtrate was concentrated in vacuum. The residue was purified by flash column chromatography (PE/EtOAc = 95:5) on silica gel to afford the desired product **S21** (4.68 g, 91%) as a white solid.[8] **^{1}H NMR** (500 MHz, CDCl$_3$) δ 7.89 (d, J = 8.0 Hz, 1H), 7.81 (d, J = 8.0 Hz, 1H), 7.51 (d, J = 8.0 Hz, 1H), 7.29 (d, J = 7.5 Hz, 1H), 7.08 – 7.00 (m, 2H), 2.06 (s, 3H).

Compound **S22** was prepared following the **Typical Procedure B**

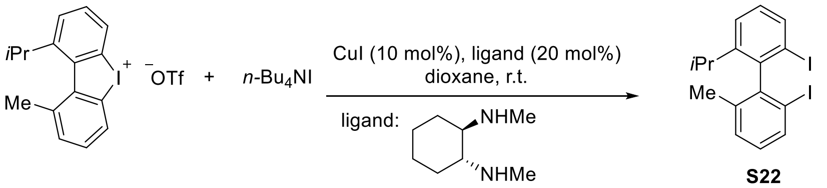

S22

The reaction of 1-isopropyl-9-methyl cyclic diaryliodonium (6.59 g, 13.6 mmol, 1.0 equiv) and n-Bu$_4$NI (10.05 g, 27.2 mmol, 2.0 equiv) afforded the desired product **S22** (5.62 g, 90%) as a white solid.[9] **^{1}H NMR** (500 MHz, CDCl$_3$) δ 7.81 (d, J = 7.5 Hz, 2H), 7.40 (d, J = 8.0 Hz, 1H), 7.28 (d, J = 7.5 Hz, 1H), 7.15 – 7.08 (m, 1H), 7.04 – 6.97 (m, 1H), 2.48 (hept, J = 7.0 Hz, 1H), 2.05 (s, 3H), 1.23 (d, J = 7.0 Hz, 3H), 1.08 (d, J = 7.0 Hz, 3H).

Typical Procedure for synthesis of dialdehyde[10] (Typical Procedure C)

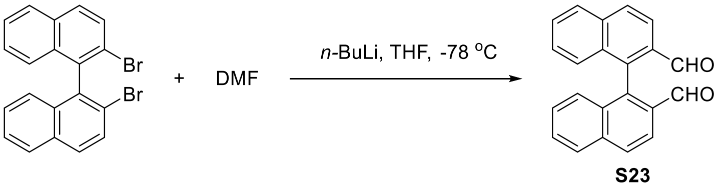

S23

Under nitrogen atmosphere, to a mixture of 2,2'-dibromo-1,1'-binaphthalene (3.71 g, 9.0 mmol, 1.0 equiv) in THF (150 mL) was added n-BuLi (2.4 M in hexanes, 15.0 mL, 36.0 mmol, 4.0 equiv) dropwise at -78 °C. After addition, the reaction mixture was stirred at -78 °C for 1.5 h, followed by the addition of dry DMF (4.2 mL, 54.0 mmol, 6.0 equiv). The stirring was continued for 0.5 h,then the cooling bath was removed and stirring was continued at room temperature for 12 h. Water (30 mL) was added and the product was extracted with EtOAc for three times. The combined organic layer was washed with brine and dried over anhydrous Na$_2$SO$_4$. After filtration the solvent was concentrated in vacuum and the residue was purified by flash column chromatography (PE/EtOAc = 10:1) on silica gel to afford the desired product **S23** (1.95 g, 63%) as a yellow-white solid. **^{1}H**

NMR (500 MHz, $CDCl_3$) δ 9.65 (s, 1H), 8.21 (d, J = 8.5 Hz, 1H), 8.12 (d, J = 8.5 Hz, 1H), 8.00 (d, J = 8.0 Hz, 1H), 7.64 – 7.57 (m, 1H), 7.38 – 7.30 (m, 1H), 7.28 – 7.22 (m, 1H). **^{13}C NMR** (126 MHz, $CDCl_3$) δ 190.9, 139.5, 135.8, 133.3, 133.1, 129.6, 129.3, 128.4, 127.8, 127.1, 122.2. HRMS (ESI) calcd for $C_{22}H_{14}O_2Na$ $[M+Na]^+$ 333.0891, found 333.0891.

Compound **S24** was prepared following the **Typical Procedure C**

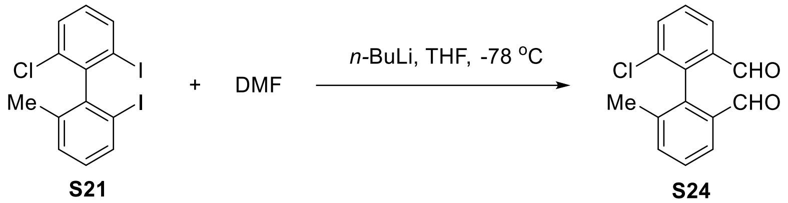

The reaction of **S21** (4.67 g, 10.26 mmol, 1.0 equiv), *n*-BuLi (2.4 M in hexanes, 12.8 mL, 30.8 mmol, 3.0 equiv) and DMF (4.8 mL, 61.6 mmol, 6.0 equiv) afforded **S24** (1.96 g, 74%) as a white solid. **^{1}H NMR** (500 MHz, $CDCl_3$) δ 9.68 (s, 1H), 9.56 (s, 1H), 7.98 (d, J = 7.5 Hz, 1H), 7.90 (d, J = 7.5 Hz, 1H), 7.78 (d, J = 8.0 Hz, 1H), 7.60 (d, J = 7.5 Hz, 1H), 7.58 – 7.53 (m, 2H), 2.02 (s, 3H). **^{13}C NMR** (126 MHz, $CDCl_3$) δ 190.9, 190.1, 139.5, 138.0, 136.7, 135.9, 135.8, 134.9, 134.8, 134.5, 129.7, 129.2, 127.7, 126.9, 19.6. HRMS (ESI) calcd for $C_{15}H_{12}ClO_2$ $[M+H]^+$ 259.0526, found 259.0511.

Compound **S25** was prepared following the **Typical Procedure C**

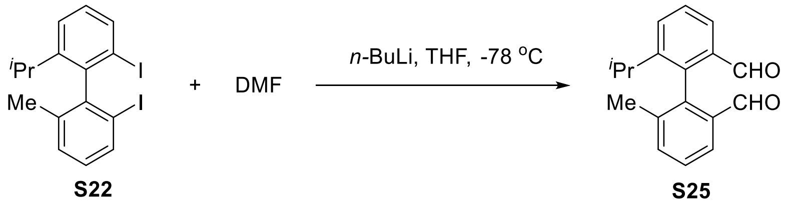

The reaction of **S22** (2.87 g, 6.16 mmol, 1.0 equiv), *n*-BuLi (2.4 M in hexanes, 7.7 mL, 18.5 mmol, 3.0 equiv) and DMF (2.9 mL, 37.0 mmol, 6.0 equiv) afforded **S25** (1.27 g, 78%) as a white solid. **^{1}H NMR** (500 MHz, $CDCl_3$) δ 9.61 (s, 1H), 9.55 (s, 1H), 7.91 (d, J = 7.5 Hz, 2H), 7.71 (d, J = 8.0 Hz, 1H), 7.62 – 7.55 (m, 2H), 7.54 – 7.48 (m, 1H), 2.48 (p, J = 7.0 Hz, 1H), 2.00 (s, 3H), 1.14 (d, J = 7.0 Hz, 3H), 1.03 (d, J = 7.0 Hz, 3H). **^{13}C NMR** (126 MHz, $CDCl_3$) δ 191.5, 191.3, 148.2, 139.6, 138.1, 138.0, 135.7, 135.0, 134.2, 131.9, 129.1, 128.5, 126.4, 126.0, 29.6, 23.8, 23.7, 19.9. HRMS (ESI) calcd for $C_{18}H_{19}O_2$ $[M+H]^+$ 267.1385, found 267.1367.

Typical Procedure for synthesis of diketone[11] (Typical Procedure D)

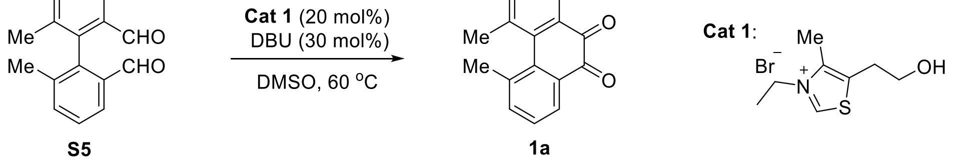

To a stirring mixture of **S5** (2.38 g, 10.0 mmol, 1.0 equiv) and **Cat 1** (514 mg, 2.0 mmol, 20 mol%) in DMSO (100 mL) was added 1,8-diazabicyclo[5.4.0]undec-7-ene (DBU) (0.45 mL, 3.0 mmol, 30 mol%) slowly at 60 °C. The mixture was stirred at this temperature for 12 h and then

under air atmosphere for 1 h. The reaction mixture was then poured into H_2O (500 mL), extracted with CH_2Cl_2 for three times. The combined organic layer was washed with brine and dried over anhydrous Na_2SO_4. After filtration the solvent was concentrated in vacuum and purified by flash column chromatography (PE/EtOAc = 10:1) on silica gel to afford **1a** (991.5 mg, 42%) as a yellow solid. **^{1}H NMR** (500 MHz, $CDCl_3$) δ 7.86 (d, *J* = 7.4 Hz, 2H), 7.56 (d, *J* = 7.3 Hz, 2H), 7.43 – 7.33 (m, 2H), 2.32 (s, 6H). **^{13}C NMR** (126 MHz, $CDCl_3$) δ 184.9, 138.0, 137.34, 137.29, 130.9, 128.3, 126.8, 20.7. HRMS (ESI) calcd for $C_{16}H_{13}O_2$ $[M+H]^+$ 237.0916, found 237.0910.

Compound **1b** was prepared following the **Typical Procedure D**

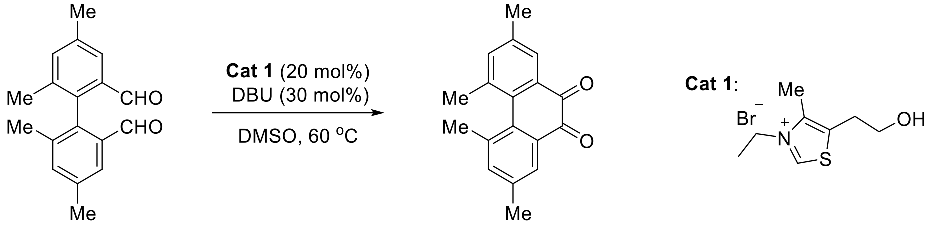

S10 **1b**

The reaction of **S10** (799.0 mg, 3.0 mmol, 1.0 equiv) and **Cat 1** (154.3 mg, 0.60 mmol 20 mol%) and DBU (0.13 mL, 0.90 mmol, 30 mol%) afforded **1b** (432.0 mg, 55%) as a yellow solid. **^{1}H NMR** (400 MHz, $CDCl_3$) δ 7.71 – 7.65 (m, 2H), 7.38 – 7.33 (m, 2H), 2.38 (s, 6H), 2.27 (s, 6H). **^{13}C NMR** (101 MHz, $CDCl_3$) δ 185.2, 138.8, 138.1, 137.0, 135.1, 130.8, 127.4, 20.73, 20.66. HRMS (ESI) calcd for $C_{18}H_{16}O_2Na$ $[M+Na]^+$ 287.1048, found 287.1048.

Compound **1c** was prepared following the **Typical Procedure D**

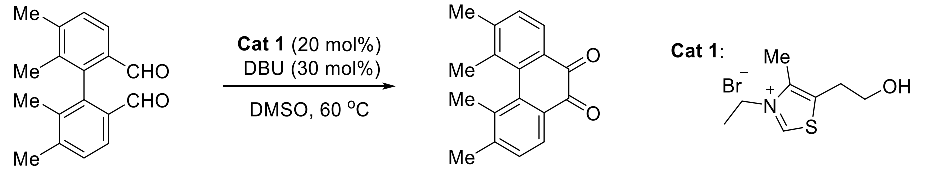

S15 **1c**

The reaction of **S15** (532.7 mg, 2.0 mmol, 1.0 equiv) and **Cat 1** (102.9 mg, 0.40 mmol 20 mol%) and DBU (90 μL, 0.60 mmol, 30 mol%) afforded **1c** (282.5 mg, 54%) as a yellow solid. **^{1}H NMR** (500 MHz, $CDCl_3$) δ 7.81 (d, *J* = 7.5 Hz, 2H), 7.27 (d, *J* = 7.5 Hz, 2H), 2.41 (s, 6H), 2.16 (s, 6H). **^{13}C NMR** (126 MHz, $CDCl_3$) δ 184.6, 146.2, 138.1, 136.3, 129.9, 129.0, 126.7, 21.5, 18.6. HRMS (ESI) calcd for $C_{18}H_{17}O_2$ $[M+H]^+$ 265.1229, found 265.1226.

Compound **1d** was prepared following the **Typical Procedure D**

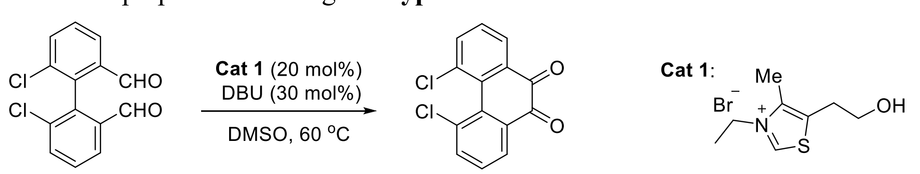

S20 **1d**

The reaction of **S20** (837.3 mg, 3.0 mmol, 1.0 equiv) and **Cat 1** (154.3 mg, 0.60 mmol 20 mol%) and DBU (0.13 mL, 0.90 mmol, 30 mol%) afforded **1d** (191.2 mg, 23%) as a yellow solid. **^{1}H NMR** (500 MHz, $CDCl_3$) δ 7.95 (d, *J* = 7.5 Hz, 2H), 7.76 (d, *J* = 8.0 Hz, 2H), 7.53 – 7.43 (m, 2H). **^{13}C NMR** (126 MHz, $CDCl_3$) δ 182.4, 137.5, 134.39, 134.36, 132.2, 130.3, 127.4. HRMS (ESI) calcd

for $C_{14}H_6Cl_2O_2Na$ [M+Na]$^+$ 298.9643, found 298.9632.

Compound **1e** was prepared following the **Typical Procedure D**

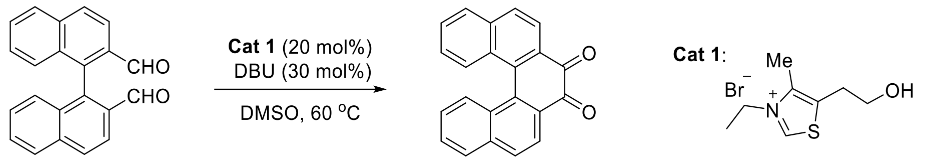

The reaction of **S23** (931.1 mg, 3.0 mmol, 1.0 equiv) and **Cat 1** (154.3 mg, 0.60 mmol 20 mol%) and DBU (0.13 mL, 0.90 mmol, 30 mol%) afforded **1e** (407.0 mg, 44%) as a deep red solid. ^{1}**H NMR** (500 MHz, $CDCl_3$) δ 8.13 (d, J = 8.0 Hz, 2H), 7.97 (d, J = 8.5 Hz, 2H), 7.92 (d, J = 8.5 Hz, 2H), 7.66 – 7.59 (m, 2H), 7.39 (d, J = 9.0 Hz, 2H), 7.34 – 7.28 (m, 2H). ^{13}**C NMR** (126 MHz, $CDCl_3$) δ 183.2, 137.52, 137.48, 130.4, 130.0, 129.3, 128.8, 128.6, 128.1, 126.2, 123.3. HRMS (ESI) calcd for $C_{22}H_{13}O_2$ [M+H]$^+$ 309.0916, found 309.0906.

Compound **1f** was prepared following the **Typical Procedure D**

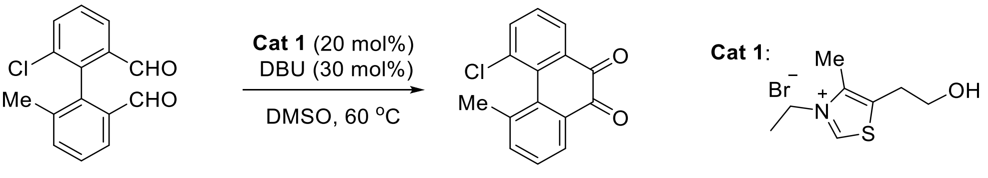

The reaction of **S24** (776.1 mg, 3.0 mmol, 1.0 equiv) and **Cat 1** (154.3 mg, 0.60 mmol 20 mol%) and DBU (0.13 mL, 0.90 mmol, 30 mol%) afforded **1f** (216.5 mg, 28%) as a yellow solid. ^{1}**H NMR** (500 MHz, $CDCl_3$) δ 7.93 (d, J = 7.5 Hz, 1H), 7.86 (d, J = 7.5 Hz, 1H), 7.72 (d, J = 8.0 Hz, 1H), 7.59 (d, J = 7.5 Hz, 1H), 7.46 – 7.39 (m, 2H), 2.46 (s, 3H). ^{13}**C NMR** (126 MHz, $CDCl_3$) δ 183.7, 183.6, 138.5, 138.4, 137.1, 136.5, 134.9, 133.5, 132.4, 130.7, 129.6, 129.1, 127.5, 126.7, 21.7. HRMS (ESI) calcd for $C_{15}H_{10}ClO_2$ [M+H]$^+$ 257.0369, found 257.0368.

Compound **1g** was prepared following the **Typical Procedure D**

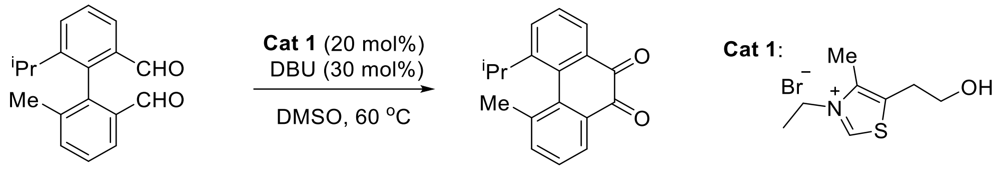

The reaction of **S25** (612.6 mg, 2.3 mmol, 1.0 equiv) and **Cat 1** (118.3 mg, 0.46 mmol 20 mol%) and DBU (0.10 mL, 0.69 mmol, 30 mol%) afforded the desired product **1g** (371.9 mg, 31%) as a yellow solid. ^{1}**H NMR** (500 MHz, $CDCl_3$) δ 7.90 – 7.81 (m, 2H), 7.70 (d, J = 7.5 Hz, 1H), 7.54 (d, J = 7.5 Hz, 1H), 7.47 – 7.42 (m, 1H), 7.39 – 7.34 (m, 1H), 3.15 (p, J = 6.5 Hz, 1H), 2.36 (s, 3H), 1.51 (d, J = 6.5 Hz, 3H), 0.83 (d, J = 6.5 Hz, 3H). ^{13}**C NMR** (126 MHz, $CDCl_3$) δ 185.3, 185.1, 148.8, 137.8, 137.4, 137.2, 135.6, 132.7, 131.0, 130.8, 129.3, 128.2, 127.0, 126.8, 30.4, 27.5, 21.1, 20.8. HRMS (ESI) calcd for $C_{18}H_{17}O_2$ [M+H]$^+$ 265.1229, found 265.1216.

Typical Procedure for Rh-catalyzed addition of arylboronic acids to diketones (Typical Procedure E)

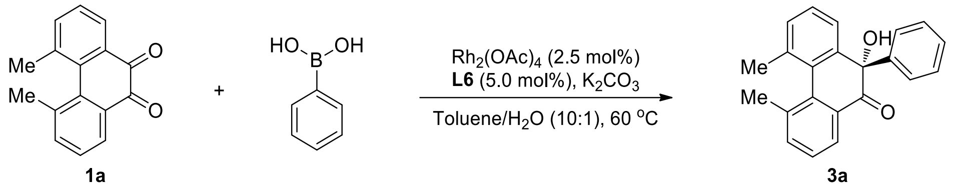

1a **3a**

Under nitrogen atmosphere, to a Schlenk tube was sequentially added **1a** (94.5 mg, 0.40 mmol, 1.0 equiv), phenylboronic acid (122.0 mg, 1.0 mmol, 2.5 equiv), $Rh_2(OAc)_4$ (4.4 mg, 2.5 mol%), **L6** (14.8 mg, 5.0 mol%) and K_2CO_3 (55.3 mg, 0.40 mmol, 1.0 equiv) in toluene (3 mL) and deionized water (0.3 mL) was heated at 60 °C for 12 h. After being cooled to room temperature, the solvent was removed and the residue was purified by flash chromatography on silica gel (PE/EtOAc = 100:10) to afford **3a** (124.8 mg, 99%, 96% ee). $[\alpha]_D^{20}$ – 195 (c 1.00, $CHCl_3$). HPLC conditions: Chiralcel AD-H, isopropanol/hexane = 10:90, flow: 1.0 mL/min, λ = 254 nm. **^{1}H NMR** (400 MHz, $CDCl_3$) δ 7.76 – 7.71 (m, 1H), 7.46 – 7.38 (m, 2H), 7.38 – 7.34 (m, 2H), 7.19 – 7.14 (m, 1H), 7.12 – 7.06 (m, 5H), 4.77 (s, 1H), 2.31 (s, 3H), 2.27 (s, 3H). **^{13}C NMR** (101 MHz, $CDCl_3$) δ 201.5, 141.3, 137.0, 136.4, 135.98, 135.95, 135.3, 132.2, 131.2, 130.8, 128.3, 128.1, 127.9, 127.5, 126.4, 124.12, 124.07, 81.2, 20.6, 20.3. HRMS (ESI) calcd for $C_{22}H_{18}O_2Na$ $[M+Na]^+$ 337.1204, found 337.1210.

Compound **3b** was prepared following the **Typical Procedure E**

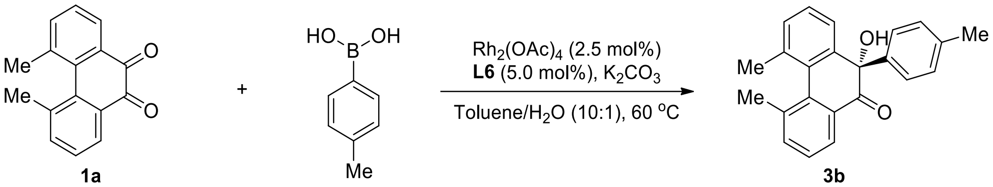

1a **3b**

The reaction of **1a** (94.5 mg, 0.40 mmol, 1.0 equiv), 4-tolylboronic acid (136.0 mg, 1.0 mmol, 2.5 equiv), $Rh_2(OAc)_4$ (4.4 mg, 2.5 mol%), **L6** (14.8 mg, 5.0 mol%) and K_2CO_3 (55.3 mg, 0.40 mmol, 1.0 equiv) in toluene (3 mL) and deionized water (0.3 mL) at 60 °C for 12 h afforded **3b** (130.2 mg, 99%, 93% ee). $[\alpha]_D^{20}$ – 203 (c 1.00, $CHCl_3$). HPLC conditions: Chiralcel AD-H, isopropanol/hexane = 10:90, flow: 1.0 mL/min, λ = 254 nm. **^{1}H NMR** (500 MHz, $CDCl_3$) δ 7.72 (d, *J* = 7.5 Hz, 1H), 7.45 (d, *J* = 7.5 Hz, 1H), 7.42 – 7.38 (m, 1H), 7.38 – 7.33 (m, 2H), 7.19 – 7.14 (m, 1H), 6.96 – 6.95 (m, 2H), 6.90 – 6.88 (m, 2H), 4.72 (s, 1H), 2.32 (s, 3H), 2.27 (s, 3H), 2.17 (s, 3H). **^{13}C NMR** (101 MHz, $CDCl_3$) δ 201.6, 138.4, 137.6, 137.1, 136.3, 136.2, 135.9, 135.3, 132.2, 131.1, 130.8, 128.8, 128.3, 127.5, 126.3, 124.1, 124.0, 81.0, 20.9, 20.6, 20.3. HRMS (ESI) calcd for $C_{23}H_{20}O_2Na$ $[M+Na]^+$ 351.1361, found 351.1360.

Compound **3c** was prepared following the **Typical Procedure E**

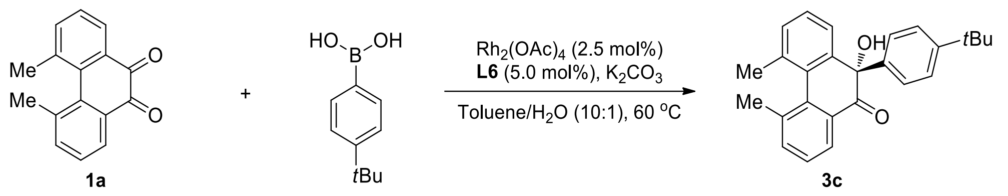

The reaction of **1a** (94.5 mg, 0.40 mmol, 1.0 equiv), 4-*tert*-butylbenzeneboronic acid (178.0 mg, 1.0 mmol, 2.5 equiv), $Rh_2(OAc)_4$ (4.4 mg, 2.5 mol%), **L6** (14.8 mg, 5.0 mol%) and K_2CO_3 (55.3 mg, 0.40 mmol, 1.0 equiv) in toluene (3 mL) and deionized water (0.3 mL) at 60 °C for 12 h afforded **3c** (147.6 mg, 99%, 95% ee). $[\alpha]_D^{20}$ – 128 (c 1.60, $CHCl_3$). HPLC conditions: Chiralcel ID, isopropanol/hexane = 7:93, flow: 1.0 mL/min, λ = 254 nm. **^{1}H NMR** (500 MHz, $CDCl_3$) δ 7.73 (d, *J* = 7.5 Hz, 1H), 7.49 (d, *J* = 7.5 Hz, 1H), 7.41 – 7.36 (m, 2H), 7.36 – 7.33 (m, 1H), 7.19 – 7.15 (m, 1H), 7.14 – 7.10 (m, 2H), 7.05 – 7.00 (m, 2H), 4.80 (s, 1H), 2.35 (s, 3H), 2.28 (s, 3H), 1.19 (s, 9H). **^{13}C NMR** (126 MHz, $CDCl_3$) δ 201.5, 150.6, 138.3, 137.1, 136.5, 136.4, 135.9, 135.2, 132.0, 131.1, 130.6, 128.2, 127.4, 126.0, 125.1, 124.2, 124.0, 80.8, 34.3, 31.1, 20.6, 20.3. HRMS (ESI) calcd for $C_{26}H_{26}O_2Na$ $[M+Na]^+$ 393.1830, found 393.1833.

Compound **3d** was prepared following the **Typical Procedure E**

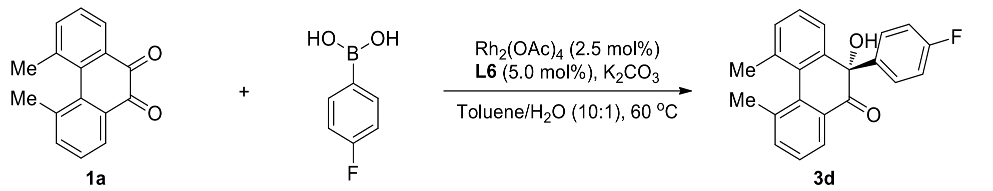

The reaction of **1a** (94.5 mg, 0.40 mmol, 1.0 equiv), 4-fluorobenzeneboronic acid (139.9 mg, 1.0 mmol, 2.5 equiv), $Rh_2(OAc)_4$ (4.4 mg, 2.5 mol%), **L6** (14.8 mg, 5.0 mol%) and K_2CO_3 (55.3 mg, 0.40 mmol, 1.0 equiv) in toluene (3 mL) and deionized water (0.3 mL) at 60 °C for 12 h afforded **3d** (129.1 mg, 97%, 95% ee). $[\alpha]_D^{20}$ – 195 (c 1.00, $CHCl_3$). HPLC conditions: Chiralcel AD-H, isopropanol/hexane = 10:90, flow: 1.0 mL/min, λ = 254 nm. **^{1}H NMR** (400 MHz, $CDCl_3$) δ 7.72 (dd, *J* = 7.6, 1.2 Hz, 1H), 7.45 – 7.42 (m, 1H), 7.41 – 7.39 (m, 1H), 7.39 – 7.35 (m, 2H), 7.22 – 7.15 (m, 1H), 7.08 – 7.02 (m, 2H), 6.81 – 6.72 (m, 2H), 4.73 (s, 1H), 2.31 (s, 3H), 2.27 (s, 3H). **^{13}C NMR** (101 MHz, $CDCl_3$) δ 201.3, 162.2 (d, *J* = 248 Hz), 137.3 (d, *J* = 3.1 Hz), 136.9, 136.5, 136.0, 135.8, 135.5, 132.1, 131.4, 130.6, 128.43, 128.37, 128.3, 127.7, 124.1 (d, *J* = 16.2 Hz), 115.0 (d, *J* = 21.7 Hz), 80.6, 20.6, 20.3. **^{19}F NMR** (376 MHz, $CDCl_3$) δ -114.1. HRMS (ESI) calcd for $C_{22}H_{17}FO_2Na$ $[M+Na]^+$ 355.1110, found 355.1109.

Compound **3e** was prepared following the **Typical Procedure E**

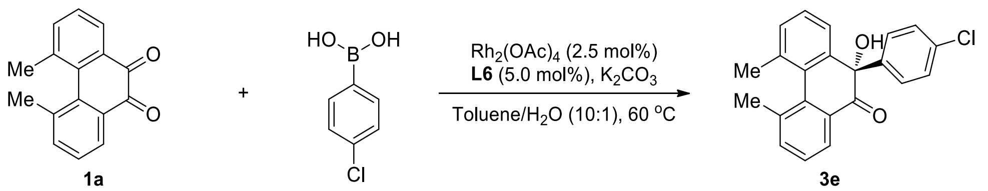

The reaction of **1a** (94.5 mg, 0.40 mmol, 1.0 equiv), 4-chlorophenylboronic acid (156.4 mg, 1.0 mmol, 2.5 equiv), $Rh_2(OAc)_4$ (4.4 mg, 2.5 mol%), **L6** (14.8 mg, 5.0 mol%) and K_2CO_3 (55.3 mg, 0.40 mmol, 1.0 equiv) in toluene (3 mL) and deionized water (0.3 mL) at 60 °C for 12 h afforded **3e** (135.4 mg, 97%, 92% ee). $[\alpha]_D^{20}$ – 182 (c 1.00, $CHCl_3$). HPLC conditions: Chiralcel AD-H,

isopropanol/hexane = 10:90, flow: 1.0 mL/min, λ = 254 nm. **^{1}H NMR** (500 MHz, $CDCl_3$) δ 7.73 – 7.69 (m, 1H), 7.43 (d, *J* = 7.0 Hz, 1H), 7.42 – 7.38 (m, 2H), 7.38 – 7.35 (m, 1H), 7.22 – 7.16 (m, 1H), 7.08 – 7.04 (m, 2H), 7.03 – 6.99 (m, 2H), 4.75 (s, 1H), 2.32 (s, 3H), 2.27 (s, 3H). **^{13}C NMR** (101 MHz, $CDCl_3$) δ 201.1, 140.0, 136.9, 136.6, 136.1, 135.53, 135.51, 133.8, 132.1, 131.5, 130.6, 128.5, 128.3, 127.9, 127.7, 124.2, 124.0, 80.6, 20.6, 20.3. HRMS (ESI) calcd for $C_{22}H_{17}ClO_2Na$ $[M+Na]^+$ 371.0815, found 371.0815.

Compound **3f** was prepared following the **Typical Procedure E**

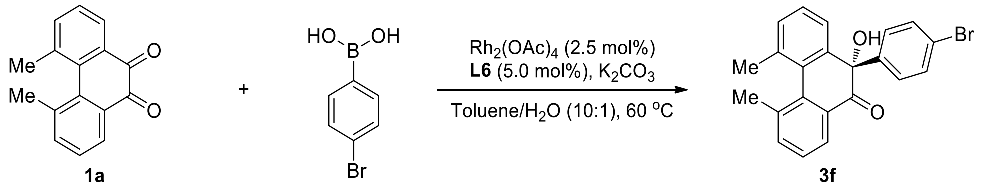

The reaction of **1a** (94.5 mg, 0.40 mmol, 1.0 equiv), 4-bromophenylboronic acid (200.8 mg, 1.0 mmol, 2.5 equiv), $Rh_2(OAc)_4$ (4.4 mg, 2.5 mol%), **L6** (14.8 mg, 5.0 mol%) and K_2CO_3 (55.3 mg, 0.40 mmol, 1.0 equiv) in toluene (3 mL) and deionized water (0.3 mL) at 60 °C for 12 h afforded **3f** (153.6 mg, 98%, 94% ee). $[\alpha]_D^{20}$ – 168 (c 1.00, $CHCl_3$). HPLC conditions: Chiralcel AD-H, isopropanol/hexane = 10:90, flow: 1.0 mL/min, λ = 254 nm. **^{1}H NMR** (400 MHz, $CDCl_3$) δ 7.70 (dd, *J* = 7.2, 1.6 Hz, 1H), 7.46 – 7.42 (m, 1H), 7.42 – 7.38 (m, 2H), 7.38 – 7.34 (m, 1H), 7.24 – 7.21 (m, 1H), 7.21 – 7.20 (m, 1H), 7.20 – 7.17 (m, 1H), 6.98 – 6.92 (m, 2H), 4.75 (s, 1H), 2.32 (s, 3H), 2.27 (s, 3H). **^{13}C NMR** (101 MHz, $CDCl_3$) δ 201.0, 140.6, 136.9, 136.7, 136.1, 135.51, 135.47, 132.1, 131.5, 131.3, 130.5, 128.5, 128.2, 127.7, 124.2, 124.0, 122.1, 80.6, 20.6, 20.3. HRMS (ESI) calcd for $C_{22}H_{17}BrO_2Na$ $[M+Na]^+$ 415.0310, found 415.0313.

Compound **3g** was prepared following the **Typical Procedure E**

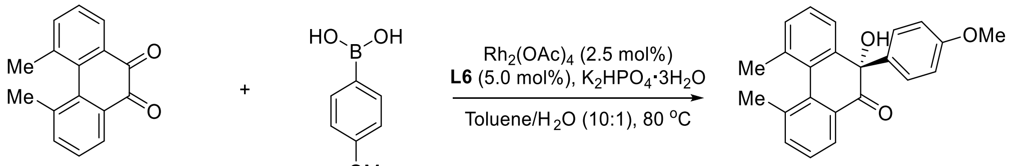

The reaction of **1a** (94.5 mg, 0.40 mmol, 1.0 equiv), 4-methoxyphenylboronic acid (182.4 mg, 1.2 mmol, 3.0 equiv), $Rh_2(OAc)_4$ (4.4 mg, 2.5 mol%), **L6** (14.8 mg, 5.0 mol%) and $K_2HPO_4 \cdot 3H_2O$ (91.3 mg, 0.40 mmol, 1.0 equiv) in toluene (3 mL) and deionized water (0.3 mL) at 80 °C for 12 h afforded **3g** (134.6 mg, 98%, 90% ee). $[\alpha]_D^{20}$ – 151 (c 1.00, $CHCl_3$). HPLC conditions: Chiralcel AD-H, isopropanol/hexane = 20:80, flow: 1.0 mL/min, λ = 254 nm. **^{1}H NMR** (500 MHz, $CDCl_3$) δ 7.73 (d, *J* = 7.5 Hz, 1H), 7.45 (d, *J* = 7.5 Hz, 1H), 7.42 – 7.38 (m, 1H), 7.38 – 7.34 (m, 2H), 7.21 – 7.14 (m, 1H), 7.03 – 6.97 (m, 2H), 6.64 – 6.59 (m, 2H), 4.71 (s, 1H), 3.66 (s, 3H), 2.31 (s, 3H), 2.27 (s, 3H). **^{13}C NMR** (126 MHz, $CDCl_3$) δ 201.6, 159.0, 136.9, 136.3, 136.2, 135.9, 135.3, 133.5, 132.1, 131.1, 130.7, 128.3, 127.7, 127.5, 124.04, 123.96, 113.4, 80.7, 55.1, 20.6, 20.3. HRMS (ESI) calcd for $C_{23}H_{20}O_3Na$ $[M+Na]^+$ 367.1310, found 367.1312.

Compound **3h** was prepared following the **Typical Procedure E**

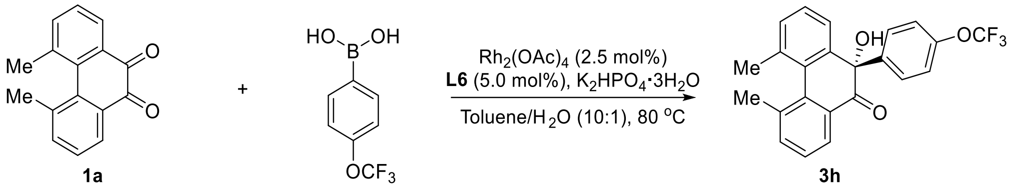

The reaction of **1a** (94.5 mg, 0.40 mmol, 1.0 equiv), 4-trifluoromethoxyphenylboronic acid (247.1 mg, 1.2 mmol, 3.0 equiv), $Rh_2(OAc)_4$ (4.4 mg, 2.5 mol%), **L6** (14.8 mg, 5.0 mol%) and $K_2HPO_4·3H_2O$ (91.3 mg, 0.40 mmol, 1.0 equiv) in toluene (3 mL) and deionized water (0.3 mL) at 80 °C for 12 h afforded **3h** (156.3 mg, 98%, 98% ee). $[\alpha]_D^{20}$ – 147 (c 1.45, $CHCl_3$). HPLC conditions: Chiralcel AD-H, isopropanol/hexane = 10:90, flow: 1.0 mL/min, λ = 254 nm. **^{1}H NMR** (500 MHz, $CDCl_3$) δ 7.72 (d, *J* = 7.0 Hz, 1H), 7.45 (d, *J* = 7.0 Hz, 1H), 7.43 – 7.39 (m, 2H), 7.39 – 7.35 (m, 1H), 7.23 – 7.16 (m, 1H), 7.16 – 7.08 (m, 2H), 6.99 – 6.89 (m, 2H), 4.81 (s, 1H), 2.33 (s, 3H), 2.27 (s, 3H). **^{13}C NMR** (126 MHz, $CDCl_3$) δ 201.0, 148.7, 140.0, 136.9, 136.7, 136.1, 135.6, 135.5, 132.1, 131.5, 130.5, 128.5, 128.0, 127.7, 124.3, 124.0, 120.4, 120.2 (q, *J* = 258 Hz), 80.5, 20.6, 20.3. **^{19}F NMR** (471 MHz, $CDCl_3$) δ -57.8. HRMS (ESI) calcd for $C_{23}H_{17}F_3O_3Na$ $[M+Na]^+$ 421.1027, found 421.1028.

Compound **3i** was prepared following the **Typical Procedure E**

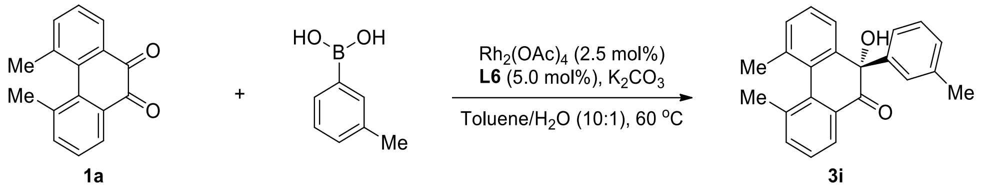

The reaction of **1a** (94.5 mg, 0.40 mmol, 1.0 equiv), 3-tolylboronic acid (136.0 mg, 1.0 mmol, 2.5 equiv), $Rh_2(OAc)_4$ (4.4 mg, 2.5 mol%), **L6** (14.8 mg, 5.0 mol%) and K_2CO_3 (55.3 mg, 0.40 mmol, 1.0 equiv) in toluene (3 mL) and deionized water (0.3 mL) at 60 °C for 12 h afforded **3i** (128.5 mg, 99%, 96% ee). $[\alpha]_D^{20}$ – 199 (c 1.25, $CHCl_3$). HPLC conditions: Chiralcel AD-H, isopropanol/hexane = 10:90, flow: 1.0 mL/min, λ = 254 nm. **^{1}H NMR** (400 MHz, $CDCl_3$) δ 7.72 (dd, *J* = 7.2, 1.2 Hz, 1H), 7.45 – 7.42 (m, 1H), 7.42 – 7.38 (m, 1H), 7.38 – 7.33 (m, 2H), 7.19 – 7.13 (m, 1H), 6.99 – 6.94 (m, 1H), 6.92 – 6.87 (m, 2H), 6.87 – 6.82 (m, 1H), 4.75 (s, 1H), 2.32 (s, 3H), 2.27 (s, 3H), 2.15 (s, 3H). **^{13}C NMR** (101 MHz, $CDCl_3$) δ 201.5, 141.2, 137.8, 137.1, 136.3, 136.1, 135.9, 135.3, 132.3, 131.2, 130.7, 128.7, 128.3, 127.9, 127.5, 127.1, 124.1, 124.0, 123.6, 81.2, 21.3, 20.6, 20.3. HRMS (ESI) calcd for $C_{23}H_{20}O_2Na$ $[M+Na]^+$ 351.1361, found 351.1367.

Compound **3j** was prepared following the **Typical Procedure E**

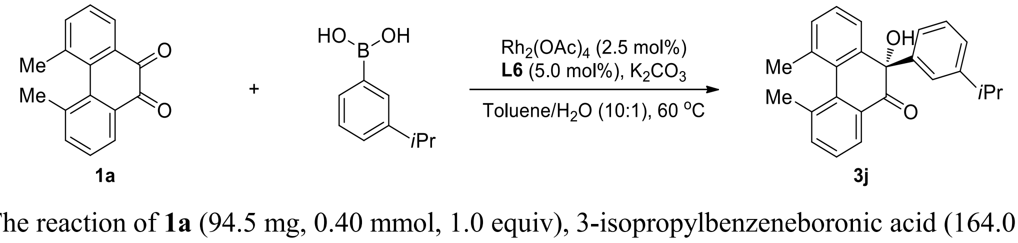

The reaction of **1a** (94.5 mg, 0.40 mmol, 1.0 equiv), 3-isopropylbenzeneboronic acid (164.0 mg, 1.0 mmol, 2.5 equiv), $Rh_2(OAc)_4$ (4.4 mg, 2.5 mol%), **L6** (14.8 mg, 5.0 mol%) and K_2CO_3 (55.3 mg, 0.40 mmol, 1.0 equiv) in toluene (3 mL) and deionized water (0.3 mL) at 60 °C for 12 h afforded **3j** (140.7 mg, 99%, 93% ee). $[\alpha]_D^{20}$ – 156 (c 1.45, $CHCl_3$). HPLC conditions: Chiralcel AD-H,

isopropanol/hexane = 10:90, flow: 1.0 mL/min, λ = 254 nm. **^{1}H NMR** (500 MHz, $CDCl_3$) δ 7.75 (d, J = 7.5 Hz, 1H), 7.44 (d, J = 7.5 Hz, 1H), 7.43 – 7.39 (m, 1H), 7.39 – 7.34 (m, 2H), 7.19 – 7.13 (m, 1H), 7.07 – 7.00 (m, 1H), 6.98 – 6.93 (m 2H), 6.88 (s, 1H), 4.78 (s, 1H), 2.69 (hept, J = 7.0 Hz, 1H), 2.32 (s, 3H), 2.28 (s, 3H), 1.07 – 1.01 (m, 6H). **^{13}C NMR** (126 MHz, $CDCl_3$) δ 201.6, 148.6, 141.1, 137.1, 136.3, 136.2, 135.8, 135.2, 132.2, 131.1, 130.8, 128.3, 128.0, 127.5, 125.9, 124.6, 124.1, 124.0, 123.9, 81.2, 33.9, 23.9, 23.6, 20.5, 20.3. HRMS (ESI) calcd for $C_{25}H_{24}O_2Na$ $[M+Na]^+$ 379.1674, found 379.1672.

Compound **3k** was prepared following the **Typical Procedure E**

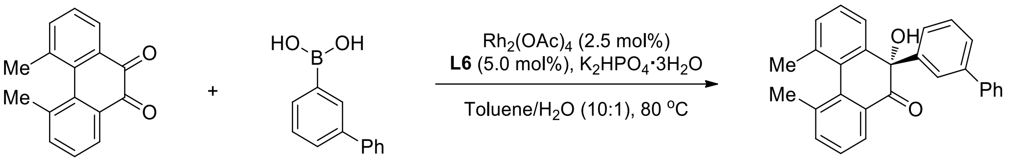

1a **3k**

The reaction of **1a** (94.5 mg, 0.40 mmol, 1.0 equiv), biphenyl-3-boronic acid (237.6 mg, 1.2 mmol, 3.0 equiv), $Rh_2(OAc)_4$ (4.4 mg, 2.5 mol%), **L6** (14.8 mg, 5.0 mol%) and $K_2HPO_4·3H_2O$ (91.3 mg, 0.40 mmol, 1.0 equiv) in toluene (3 mL) and deionized water (0.3 mL) at 80 °C for 12 h afforded **3k** (153.2 mg, 99%, 96% ee). $[\alpha]_D^{20}$ – 164 (c 1.71, $CHCl_3$). HPLC conditions: Chiralcel AD-H, isopropanol/hexane = 10:90, flow: 1.0 mL/min, λ = 254 nm. **^{1}H NMR** (500 MHz, $CDCl_3$) δ 7.78 (d, J = 7.0 Hz, 1H), 7.49 (d, J = 7.0 Hz, 1H), 7.44 – 7.40 (m, 2H), 7.39 – 7.37 (m, 2H), 7.36 – 7.35 (m, 2H), 7.35 – 7.34 (m, 1H), 7.34 – 7.32 (m, 1H), 7.32 – 7.28 (m, 2H), 7.22 – 7.18 (m, 2H), 7.17 – 7.13 (m, 1H), 4.88 (s, 1H), 2.37 (s, 3H), 2.30 (s, 3H). **^{13}C NMR** (126 MHz, $CDCl_3$) δ 201.4, 141.7, 141.0, 140.7, 137.2, 136.5, 136.1, 135.9, 135.3, 132.1, 131.3, 130.7, 128.6, 128.5, 128.4, 127.6, 127.2, 127.0, 126.7, 125.4, 125.3, 124.3, 124.1, 81.1, 20.6, 20.4. HRMS (ESI) calcd for $C_{28}H_{22}O_2Na$ $[M+Na]^+$ 413.1517, found 413.1520.

Compound **3l** was prepared following the **Typical Procedure E**

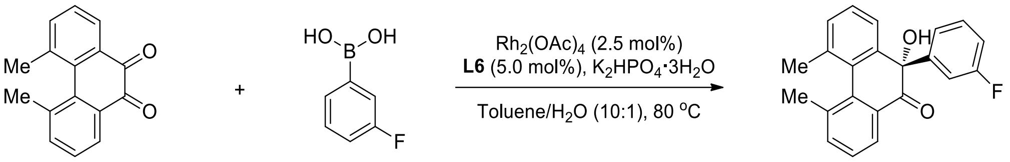

1a **3l**

The reaction of **1a** (94.5 mg, 0.40 mmol, 1.0 equiv), 3-fluorophenylboronic acid (167.9 mg, 1.2 mmol, 3.0 equiv), $Rh_2(OAc)_4$ (4.4 mg, 2.5 mol%), **L6** (14.8 mg, 5.0 mol%) and $K_2HPO_4·3H_2O$ (91.3 mg, 0.40 mmol, 1.0 equiv) in toluene (3 mL) and deionized water (0.3 mL) at 80 °C for 12 h afforded **3l** (131.4 mg, 99%, 98% ee). $[\alpha]_D^{20}$ – 168 (c 1.25, $CHCl_3$). HPLC conditions: Chiralcel AD-H, isopropanol/hexane = 10:90, flow: 1.0 mL/min, λ = 254 nm. **^{1}H NMR** (500 MHz, $CDCl_3$) δ 7.72 (d, J = 7.0 Hz, 1H), 7.45 (d, J = 7.0 Hz, 1H), 7.43 – 7.40 (m, 1H), 7.40 – 7.34 (m, 2H), 7.23 – 7.15 (m, 1H), 7.10 – 7.02 (m, 1H), 6.88 (d, J = 7.5 Hz, 1H), 6.84 – 6.74 (m, 2H), 4.81 (s, 1H), 2.33 (s, 3H), 2.27 (s, 3H). **^{13}C NMR** (126 MHz, $CDCl_3$) δ 200.9, 162.5 (d, J = 247 Hz), 144.0 (d, J = 6.7 Hz), 137.0, 136.6, 136.1, 135.5, 135.4, 132.1, 131.5, 130.5, 129.5 (d, J = 8.2 Hz), 128.5, 127.6, 124.2, 124.0, 122.2 (d, J = 2.8 Hz), 114.9 (d, J = 21.3 Hz), 113.7 (d, J = 23.1 Hz), 80.6 (d, J = 1.5

Hz), 20.5, 20.3. **^{19}F NMR** (471 MHz, $CDCl_3$) δ -112.7. HRMS (ESI) calcd for $C_{22}H_{17}FO_2Na$ $[M+Na]^+$ 355.1110, found 355.1110.

Compound **3m** was prepared following the **Typical Procedure E**

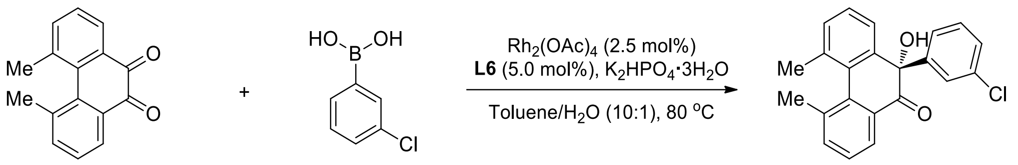

1a **3m**

The reaction of **1a** (94.5 mg, 0.40 mmol, 1.0 equiv), 3-chlorophenylboronic acid (187.6 mg, 1.2 mmol, 3.0 equiv), $Rh_2(OAc)_4$ (4.4 mg, 2.5 mol%), **L6** (14.8 mg, 5.0 mol%) and $K_2HPO_4 \cdot 3H_2O$ (91.3 mg, 0.40 mmol, 1.0 equiv) in toluene (3 mL) and deionized water (0.3 mL) at 80 °C for 12 h afforded **3m** (138.2 mg, 99%, 97% ee). $[\alpha]_D^{20}$ – 213 (c 0.85, $CHCl_3$). HPLC conditions: Chiralcel AD-H, isopropanol/hexane = 10:90, flow: 1.0 mL/min, λ = 254 nm. **^{1}H NMR** (500 MHz, $CDCl_3$) δ 7.73 – 7.68 (m, 1H), 7.43 (d, *J* = 8.0 Hz, 1H), 7.42 – 7.39 (m, 2H), 7.39 – 7.36 (m, 1H), 7.22 – 7.17 (m, 1H), 7.08 – 7.05 (m, 2H), 7.05 – 7.01 (m, 1H), 7.00 – 6.96 (m, 1H), 4.80 (s, 1H), 2.34 (s, 3H), 2.27 (s, 3H). **^{13}C NMR** (126 MHz, $CDCl_3$) δ 200.8, 143.3, 137.0, 136.7, 136.2, 135.5, 135.2, 134.1, 132.2, 131.5, 130.4, 129.3, 128.5, 128.1, 127.7, 126.8, 124.7, 124.2, 124.0, 80.7, 20.5, 20.2. HRMS (ESI) calcd for $C_{22}H_{17}ClO_2Na$ $[M+Na]^+$ 371.0815, found 371.0813.

Compound **3n** was prepared following the **Typical Procedure E**

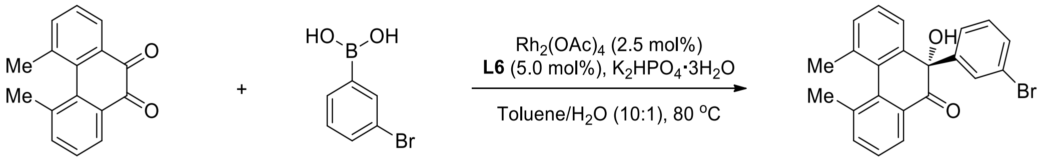

1a **3n**

The reaction of **1a** (94.5 mg, 0.40 mmol, 1.0 equiv), 3-bromophenylboronic acid (241.0 mg, 1.2 mmol, 3.0 equiv), $Rh_2(OAc)_4$ (4.4 mg, 2.5 mol%), **L6** (14.8 mg, 5.0 mol%) and $K_2HPO_4 \cdot 3H_2O$ (91.3 mg, 0.40 mmol, 1.0 equiv) in toluene (3 mL) and deionized water (0.3 mL) at 80 °C for 12 h afforded **3n** (156.1 mg, 99%, 97% ee). $[\alpha]_D^{20}$ – 213 (c 1.10, $CHCl_3$). HPLC conditions: Chiralcel AD-H, isopropanol/hexane = 13:87, flow: 1.0 mL/min, λ = 254 nm. **^{1}H NMR** (500 MHz, $CDCl_3$) δ 7.74 – 7.68 (m, 1H), 7.45 – 7.40 (m, 2H), 7.40 – 7.35 (m, 2H), 7.23 – 7.20 (m, 2H), 7.20 – 7.16 (m, 1H), 7.06 – 7.01 (m, 1H), 6.99 – 6.94 (m, 1H), 4.80 (s, 1H), 2.35 (s, 3H), 2.28 (s, 3H). **^{13}C NMR** (126 MHz, $CDCl_3$) δ 200.8, 143.6, 137.1, 136.7, 136.3, 135.5, 135.3, 132.3, 131.6, 131.0, 130.4, 129.8, 129.7, 128.6, 127.8, 125.2, 124.3, 124.1, 122.3, 80.7, 20.6, 20.3. HRMS (ESI) calcd for $C_{22}H_{17}BrO_2Na$ $[M+Na]^+$ 415.0310, found 415.0307.

Compound **3o** was prepared following the **Typical Procedure E**

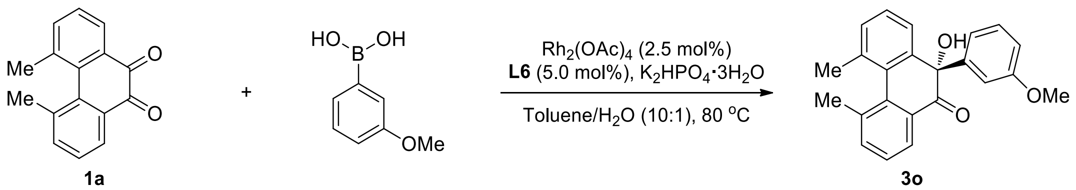

1a **3o**

The reaction of **1a** (94.5 mg, 0.40 mmol, 1.0 equiv), 3-methoxyphenylboronic acid (182.4 mg, 1.2 mmol, 3.0 equiv), $Rh_2(OAc)_4$ (4.4 mg, 2.5 mol%), **L6** (14.8 mg, 5.0 mol%) and $K_2HPO_4{\cdot}3H_2O$ (91.3 mg, 0.40 mmol, 1.0 equiv) in toluene (3 mL) and deionized water (0.3 mL) at 80 °C for 12 h afforded **3o** (127.0 mg, 92%, 86% ee). $[\alpha]_D^{20}$ – 167 (c 0.94, $CHCl_3$). HPLC conditions: Chiralcel AD-H, isopropanol/hexane = 15:85, flow: 1.0 mL/min, λ = 254 nm. **^{1}H NMR** (500 MHz, $CDCl_3$) δ 7.72 (d, *J* = 7.5 Hz, 1H), 7.45 (d, *J* = 7.0 Hz, 1H), 7.41 – 7.38 (m, 1H), 7.38 – 7.33 (m, 2H), 7.21 – 7.15 (m, 1H), 7.04 – 6.98 (m, 1H), 6.68 – 6.65 (m, 1H), 6.65 – 6.61 (m, 2H), 4.76 (s, 1H), 3.61 (s, 3H), 2.32 (s, 3H), 2.27 (s, 3H). **^{13}C NMR** (126 MHz, $CDCl_3$) δ 201.4, 159.3, 142.9, 137.1, 136.4, 135.9, 135.3, 132.2, 131.2, 130.7, 129.1, 128.3, 127.5, 124.2, 124.1, 119.0, 113.6, 112.1, 81.0, 55.0, 20.6, 20.3. HRMS (ESI) calcd for $C_{23}H_{20}O_3Na$ $[M+Na]^+$ 367.1310, found 367.1302.

Compound **3p** was prepared following the **Typical Procedure E**

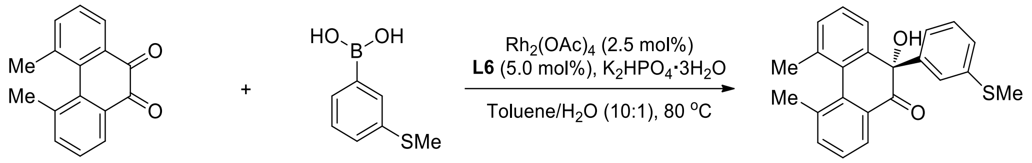

The reaction of **1a** (94.5 mg, 0.40 mmol, 1.0 equiv), 3-methylthiophenylboronic acid (201.6 mg, 1.2 mmol, 3.0 equiv), $Rh_2(OAc)_4$ (4.4 mg, 2.5 mol%), **L6** (14.8 mg, 5.0 mol%) and $K_2HPO_4{\cdot}3H_2O$ (91.3 mg, 0.40 mmol, 1.0 equiv) in toluene (3 mL) and deionized water (0.3 mL) at 80 °C for 12 h afforded **3p** (109.3 mg, 76%, 89% ee). $[\alpha]_D^{20}$ – 189 (c 1.00, $CHCl_3$). HPLC conditions: Chiralcel AD-H, isopropanol/hexane = 15:85, flow: 1.0 mL/min, λ = 254 nm. **^{1}H NMR** (500 MHz, $CDCl_3$) δ 7.71 (d, *J* = 7.0 Hz, 1H), 7.44 (d, *J* = 7.5 Hz, 1H), 7.42 – 7.39 (m, 1H), 7.39 – 7.34 (m, 2H), 7.21 – 7.15 (m, 1H), 7.04 – 7.00 (m, 1H), 7.00 – 6.95 (m, 2H), 6.89 – 6.85 (m, 1H), 4.77 (s, 1H), 2.33 (s, 3H), 2.29 (s, 3H), 2.27 (s, 3H). **^{13}C NMR** (126 MHz, $CDCl_3$) δ 201.2, 142.0, 138.4, 137.1, 136.5, 136.0, 135.8, 135.3, 132.2, 131.3, 130.6, 128.5, 128.4, 127.6, 126.1, 124.7, 124.2, 124.1, 123.3, 80.9, 20.6, 20.3, 15.7. HRMS (ESI) calcd for $C_{23}H_{20}O_2SNa$ $[M+Na]^+$ 383.1082, found 383.1073.

Compound **3q** was prepared following the **Typical Procedure E**

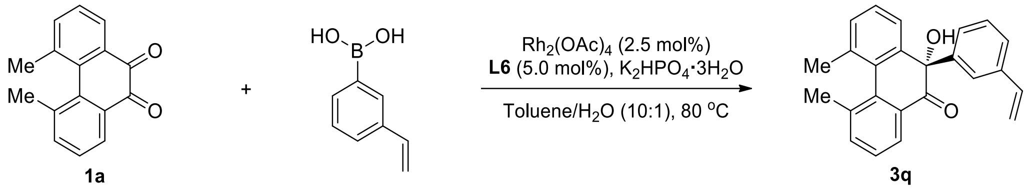

The reaction of **1a** (94.5 mg, 0.40 mmol, 1.0 equiv), 3-vinylphenylboronic acid (177.6 mg, 1.2 mmol, 3.0 equiv), $Rh_2(OAc)_4$ (4.4 mg, 2.5 mol%), **L6** (14.8 mg, 5.0 mol%) and $K_2HPO_4{\cdot}3H_2O$ (91.3 mg, 0.40 mmol, 1.0 equiv) in toluene (3 mL) and deionized water (0.3 mL) at 80 °C for 12 h afforded **3q** (135.2 mg, 99%, 95% ee). $[\alpha]_D^{20}$ – 212 (c 1.15, $CHCl_3$). HPLC conditions: Chiralcel AD-H, isopropanol/hexane = 10:90, flow: 1.0 mL/min, λ = 254 nm. **^{1}H NMR** (400 MHz, $CDCl_3$) δ 7.78 – 7.72 (m, 1H), 7.47 – 7.43 (m, 1H), 7.43 – 7.39 (m, 1H), 7.39 – 7.34 (m, 2H), 7.20 – 7.16 (m, 1H), 7.16 – 7.12 (m, 2H), 7.09 – 7.04 (m, 1H), 7.03 – 6.99 (m, 1H), 6.51 (dd, *J* = 17.6, 10.8, Hz, 1H), 5.13 (d, *J* = 17.6 Hz, 1H), 5.13 (d, *J* = 10.8 Hz, 1H), 4.82 (s, 1H), 2.34 (s, 3H), 2.28 (s, 3H). **^{13}C NMR** (101 MHz, $CDCl_3$) δ 201.3, 141.5, 137.4, 137.1, 136.4, 136.0, 135.9, 135.3, 132.2, 131.3, 130.6, 128.4, 128.2, 127.5, 125.9, 125.6, 124.5, 124.2, 124.0, 114.0, 81.0, 20.5, 20.3. HRMS (ESI)

calcd for $C_{24}H_{20}O_2Na$ [M+Na]$^+$ 363.1321, found 363.1359.

Compound **3r** was prepared following the **Typical Procedure E**

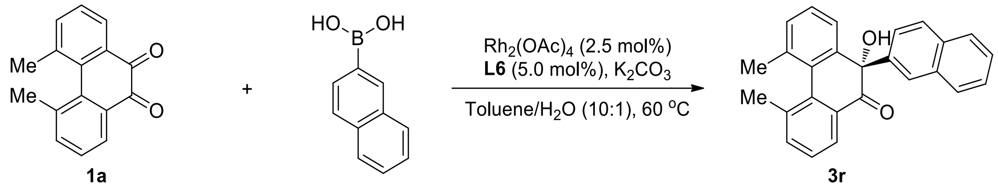

The reaction of **1a** (94.5 mg, 0.40 mmol, 1.0 equiv), 2-naphthaleneboronic acid (172.0 mg, 1.0 mmol, 2.5 equiv), $Rh_2(OAc)_4$ (4.4 mg, 2.5 mol%), **L6** (14.8 mg, 5.0 mol%) and K_2CO_3 (55.3 mg, 0.40 mmol, 1.0 equiv) in toluene (3 mL) and deionized water (0.3 mL) at 60 °C for 12 h afforded **3r** (145.0 mg, 99%, 93% ee). $[\alpha]_D^{20}$ – 271 (c 1.00, $CHCl_3$). HPLC conditions: Chiralcel AD-H, isopropanol/hexane = 10:90, flow: 1.0 mL/min, λ = 254 nm. **^{1}H NMR** (400 MHz, $CDCl_3$) δ 7.82 (d, *J* = 7.2 Hz, 1H), 7.72 – 7.66 (m, 1H), 7.62 (d, *J* = 8.4 Hz, 1H), 7.60 – 7.56 (m, 1H), 7.52 – 7.49 (m, 1H), 7.49 – 7.46 (m, 1H), 7.45 – 7.40 (m, 2H), 7.40 – 7.33 (m, 2H), 7.32 – 7.26 (m, 2H), 7.14 – 7.07 (m, 1H), 4.92 (d, *J* = 1.2 Hz, 1H), 2.34 (s, 3H), 2.31 (s, 3H). **^{13}C NMR** (101 MHz, $CDCl_3$) δ 201.4, 138.8, 137.0, 136.5, 135.94, 135.90, 135.4, 132.8, 132.7, 132.4, 131.4, 130.6, 128.4, 128.1, 128.0, 127.5, 127.3, 126.2, 125.94, 125.87, 124.2, 124.08, 124.07, 81.3, 20.6, 20.2. HRMS (ESI) calcd for $C_{26}H_{20}O_2Na$ [M+Na]$^+$ 387.1361, found 387.1356.

Compound **3s** was prepared following the **Typical Procedure E**

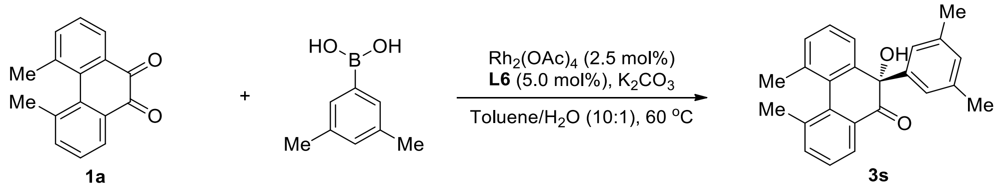

The reaction of **1a** (94.5 mg, 0.40 mmol, 1.0 equiv), 3,5-dimethylphenylboronic acid (150.0 mg, 1.0 mmol, 2.5 equiv), $Rh_2(OAc)_4$ (4.4 mg, 2.5 mol%), **L6** (14.8 mg, 5.0 mol%) and K_2CO_3 (55.3 mg, 0.40 mmol, 1.0 equiv) in toluene (3 mL) and deionized water (0.3 mL) at 60 °C for 12 h afforded **3s** (133.3 mg, 97%, 93% ee). $[\alpha]_D^{20}$ – 203 (c 1.85, $CHCl_3$). HPLC conditions: Chiralcel ID, isopropanol/hexane = 10:90, flow: 1.0 mL/min, λ = 254 nm. **^{1}H NMR** (500 MHz, $CDCl_3$) δ 7.73 (d, *J* = 7.0 Hz, 1H), 7.47 – 7.43 (m, 1H), 7.43 – 7.39 (m, 1H), 7.39 – 7.35 (m, 2H), 7.19 – 7.14 (m, 1H), 6.73 – 6.71 (m, 1H), 6.71 – 6.68 (m, 2H), 4.77 (s, 1H), 2.34 (s, 3H), 2.28 (s, 3H), 2.11 (s, 6H). **^{13}C NMR** (126 MHz, $CDCl_3$) δ 201.5, 141.0, 137.5, 137.2, 136.20, 136.16, 135.8, 135.1, 132.3, 131.1, 130.6, 129.6, 128.3, 127.4, 124.3, 124.1, 124.0, 81.1, 21.1, 20.5, 20.2. HRMS (ESI) calcd for $C_{24}H_{22}O_2Na$ [M+Na]$^+$ 365.1517, found 365.1518.

Compound **3t** was prepared following the **Typical Procedure E**

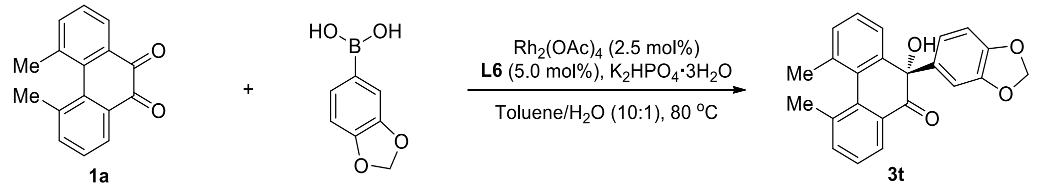

The reaction of **1a** (94.5 mg, 0.40 mmol, 1.0 equiv), 3,4-methylenedioxyphenylboronic acid

(199.1 mg, 1.2 mmol, 3.0 equiv), $Rh_2(OAc)_4$ (4.4 mg, 2.5 mol%), **L6** (14.8 mg, 5.0 mol%) and $K_2HPO_4{\cdot}3H_2O$ (91.3 mg, 0.40 mmol, 1.0 equiv) in toluene (3 mL) and deionized water (0.3 mL) at 80 °C for 12 h afforded **3t** (144.8 mg, 99%, 87% ee). $[\alpha]_D^{20}$ – 117 (c 1.30, $CHCl_3$). HPLC conditions: Chiralcel AD-H, isopropanol/hexane = 10:90, flow: 1.0 mL/min, λ = 254 nm. **^{1}H NMR** (500 MHz, $CDCl_3$) δ 7.72 (d, *J* = 7.5 Hz, 1H), 7.47 (d, *J* = 7.0 Hz, 1H), 7.42 – 7.37 (m, 2H), 7.37 – 7.33 (m, 1H), 7.22 – 7.17 (m, 1H), 6.61 – 6.56 (m, 1H), 6.55 – 6.46 (m, 2H), 5.81 (d, *J* = 4.5 Hz, 2H), 4.71 (s, 1H), 2.31 (s, 3H), 2.26 (s, 3H). **^{13}C NMR** (126 MHz, $CDCl_3$) δ 201.4, 147.5, 147.2, 136.8, 136.4, 135.94, 135.91, 135.44, 135.35, 132.1, 131.3, 130.6, 128.3, 127.5, 124.1, 124.0, 120.4, 107.7, 107.1, 101.0, 80.7, 20.5, 20.3. HRMS (ESI) calcd for $C_{23}H_{18}O_4Na$ $[M+Na]^+$ 381.1103, found 381.1102.

Compound **3u** was prepared following the **Typical Procedure E**

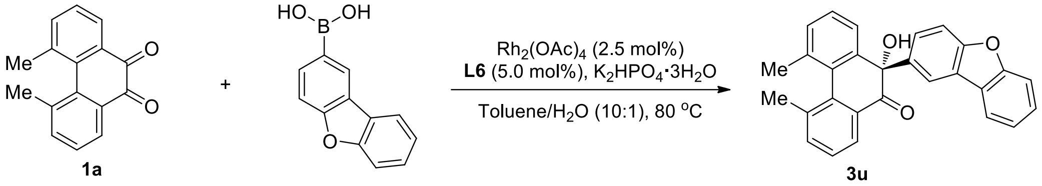

The reaction of **1a** (94.5 mg, 0.40 mmol, 1.0 equiv), dibenzo[*b,d*]furan-2-ylboronic acid (254.4 mg, 1.2 mmol, 3.0 equiv), $Rh_2(OAc)_4$ (4.4 mg, 2.5 mol%), **L6** (14.8 mg, 5.0 mol%) and $K_2HPO_4{\cdot}3H_2O$ (91.3 mg, 0.40 mmol, 1.0 equiv) in toluene (3 mL) and deionized water (0.3 mL) at 80 °C for 12 h afforded **3u** (150.1 mg, 93%, 91% ee). $[\alpha]_D^{20}$ – 213 (c 1.00, $CHCl_3$). HPLC conditions: Chiralcel AD-H, isopropanol/hexane = 20:80, flow: 1.0 mL/min, λ = 254 nm. **^{1}H NMR** (500 MHz, $CDCl_3$) δ 7.82 – 7.77 (m, 1H), 7.73 (d, *J* = 7.5 Hz, 1H), 7.65 (d, *J* = 2.0 Hz, 1H), 7.45 – 7.41 (m, 2H), 7.41 – 7.39 (m, 1H), 7.39 – 7.37 (m, 1H), 7.37 – 7.33 (m, 1H), 7.29 – 7.26 (m, 1H), 7.25 – 7.23 (m, 1H), 7.23 – 7.21 (m, 1H), 7.20 – 7.17 (m, 1H), 7.10 – 7.06 (m, 1H), 4.86 (s, 1H), 2.32 (s, 3H), 2.27 (s, 3H). **^{13}C NMR** (126 MHz, $CDCl_3$) δ 201.5, 156.4, 155.6, 137.0, 136.4, 136.2, 136.1, 135.9, 135.4, 132.3, 131.4, 130.6, 128.5, 127.6, 127.2, 125.8, 124.2, 124.1, 124.0, 123.9, 122.7, 120.5, 119.0, 111.6, 111.2, 81.2, 20.6, 20.2. HRMS (ESI) calcd for $C_{28}H_{20}O_3Na$ $[M+Na]^+$ 427.1310, found 427.1313.

Compound **3v** was prepared following the **Typical Procedure E**

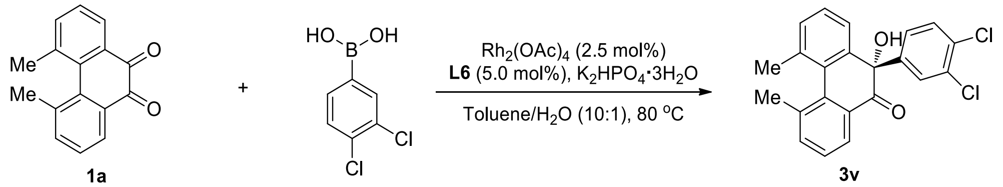

The reaction of **1a** (94.5 mg, 0.40 mmol, 1.0 equiv), 3,4-dichlorophenylboronic acid (229.0 mg, 1.2 mmol, 3.0 equiv), $Rh_2(OAc)_4$ (4.4 mg, 2.5 mol%), **L6** (14.8 mg, 5.0 mol%) and $K_2HPO_4{\cdot}3H_2O$ (91.3 mg, 0.40 mmol, 1.0 equiv) in toluene (3 mL) and deionized water (0.3 mL) at 80 °C for 12 h afforded **3v** (139.7 mg, 91%, 97% ee). $[\alpha]_D^{20}$ – 172 (c 1.30, $CHCl_3$). HPLC conditions: Chiralcel AD-H, isopropanol/hexane = 10:90, flow: 1.0 mL/min, λ = 254 nm. **^{1}H NMR** (500 MHz, $CDCl_3$) δ 7.69 (dd, *J* = 7.0, 1.5 Hz, 1H), 7.45 – 7.42 (m, 2H), 7.42 – 7.36 (m, 2H), 7.24 – 7.19 (m, 1H), 7.18 – 7.13 (m, 2H), 6.94 (dd, *J* = 8.5, 2.0 Hz, 1H), 4.80 (s, 1H), 2.35 (s, 3H), 2.27 (s, 3H). **^{13}C NMR**

(126 MHz, $CDCl_3$) δ 200.4, 141.6, 136.9, 136.2, 135.6, 134.9, 132.3, 132.1, 132.0, 131.7, 130.2, 130.0, 128.7, 128.6, 127.9, 125.9, 124.3, 123.9, 80.2, 20.5, 20.2. HRMS (ESI) calcd for $C_{22}H_{16}Cl_2O_2Na$ $[M+Na]^+$ 405.0425, found 405.0424.

Compound **3w** was prepared following the **Typical Procedure E**

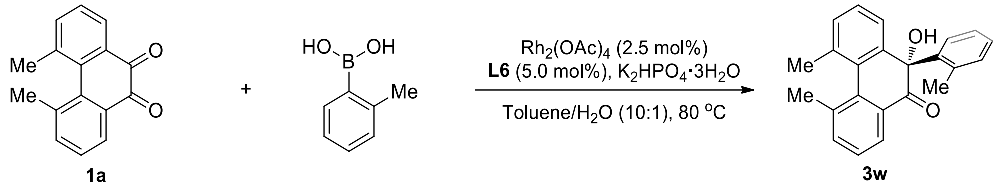

The reaction of **1a** (94.5 mg, 0.40 mmol, 1.0 equiv), 2-tolylboronic acid (475.9 mg, 1.4 mmol, 3.5 equiv), $Rh_2(OAc)_4$ (4.4 mg, 2.5 mol%), **L6** (14.8 mg, 5.0 mol%) and K_2CO_3 (55.3 mg, 0.40 mmol, 1.0 equiv) in toluene (3 mL) and deionized water (0.3 mL) at 90 °C for 18 h afforded **3w** (102.4 mg, 78%, 75% ee). $[\alpha]_D^{20}$ – 74.4 (c 1.00, $CHCl_3$). HPLC conditions: Chiralcel IC, isopropanol/hexane = 10:90, flow: 1.0 mL/min, λ = 254 nm. **^{1}H NMR** (400 MHz, $CDCl_3$) δ 7.78 – 7.73 (m, 1H), 7.44 – 7.38 (m, 1H), 7.37 – 7.34 (m, 1H), 7.32 (d, *J* = 7.2 Hz, 1H), 7.30 – 7.26 (m, 1H), 7.15 – 7.09 (m, 1H), 7.01 – 6.96 (m, 1H), 6.96 – 6.92 (d, *J* = 6.1 Hz, 1H), 6.90 – 6.85 (m, 1H), 6.84 – 6.78 (m, 1H), 4.38 (s, 1H), 2.27 (s, 3H), 2.24 (s, 3H), 2.24 (s, 3H). **^{13}C NMR** (101 MHz, $CDCl_3$) δ 203.2, 138.4, 138.1, 136.6, 136.1, 135.7, 135.6, 135.2, 133.2, 132.2, 132.0, 131.3, 128.4, 128.2, 128.0, 127.5, 125.2, 124.2, 122.2, 83.4, 20.3, 20.1, 20.0. HRMS (ESI) calcd for $C_{23}H_{20}O_2Na$ $[M+Na]^+$ 351.1361, found 351.1364.

Compound **3x** was prepared following the **Typical Procedure E**

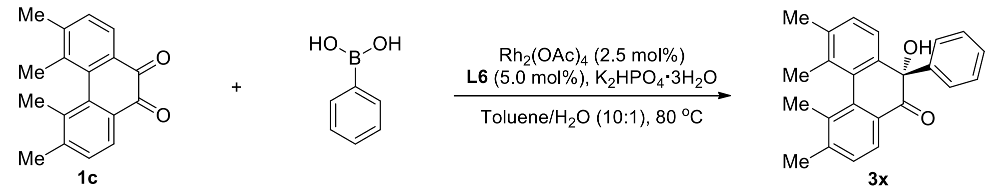

The reaction of **1c** (105.7 mg, 0.40 mmol, 1.0 equiv), phenylboronic acid (146.3 mg, 1.2 mmol, 3.0 equiv), $Rh_2(OAc)_4$ (4.4 mg, 2.5 mol%), **L6** (14.8 mg, 5.0 mol%) and $K_2HPO_4{\cdot}3H_2O$ (91.3 mg, 0.40 mmol, 1.0 equiv) in toluene (3 mL) and deionized water (0.3 mL) at 80 °C for 12 h afforded **3x** (133.7 mg, 98%, 87% ee). $[\alpha]_D^{20}$ – 194 (c 1.25, $CHCl_3$). HPLC conditions: Chiralcel AD-H, isopropanol/hexane = 10:90, flow: 1.0 mL/min, λ = 254 nm. **^{1}H NMR** (500 MHz, $CDCl_3$) δ 7.66 (d, *J* = 8.0 Hz, 1H), 7.39 (d, *J* = 7.5 Hz, 1H), 7.31 (d, *J* = 7.5 Hz, 1H), 7.12 – 7.07 (m, 5H), 7.05 (d, *J* = 7.5 Hz, 1H), 4.81 (s, 1H), 2.39 (s, 3H), 2.29 (s, 3H), 2.18 (s, 3H), 2.11 (s, 3H). **^{13}C NMR** (126 MHz, $CDCl_3$) δ 201.3, 144.2, 141.7, 137.8, 137.6, 135.0, 134.2, 133.4, 132.7, 129.7, 129.0, 128.7, 128.0, 127.7, 126.4, 124.0, 123.9, 80.9, 21.1, 20.8, 18.3, 18.1. HRMS (ESI) calcd for $C_{24}H_{22}O_2Na$ $[M+Na]^+$ 365.1517, found 365.1512.

Compound **3y** was prepared following the **Typical Procedure E**

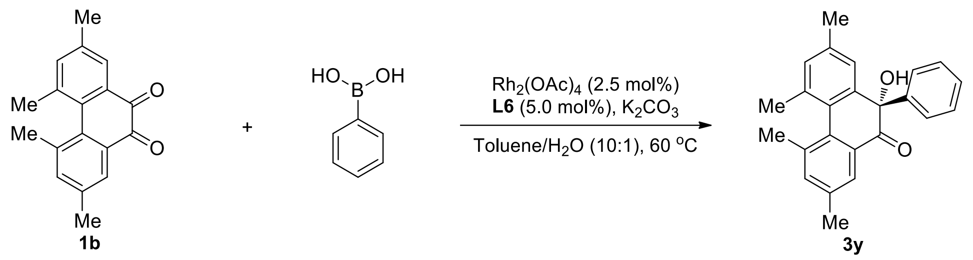

The reaction of **1b** (105.7 mg, 0.40 mmol, 1.0 equiv), phenylboronic acid (122.0 mg, 1.0 mmol, 2.5 equiv), $Rh_2(OAc)_4$ (4.4 mg, 2.5 mol%), **L6** (14.8 mg, 5.0 mol%) and K_2CO_3 (55.3 mg, 0.40 mmol, 1.0 equiv) in toluene (3 mL) and deionized water (0.3 mL) at 60 °C for 12 h afforded **3y** (135.9 mg, 99%, 95% ee). $[\alpha]_D^{20}$ – 174 (c 1.15, $CHCl_3$). HPLC conditions: Chiralcel AD-H, isopropanol/hexane = 10:90, flow: 1.0 mL/min, λ = 254 nm. **^{1}H NMR** (400 MHz, $CDCl_3$) δ 7.54 (s, 1H), 7.26 (s, 1H), 7.17 (s, 2H), 7.14 – 7.08 (m, 5H), 4.83 (s, 1H), 2.41 (s, 3H), 2.28 (s, 3H), 2.25 (s, 3H), 2.24 (s, 3H). **^{13}C NMR** (101 MHz, $CDCl_3$) δ 201.7, 141.6, 137.9, 137.1, 137.0, 136.0, 135.6, 135.1, 134.7, 131.8, 130.5, 129.7, 128.0, 127.8, 126.4, 124.8, 124.7, 81.1, 21.2, 20.7, 20.5, 20.3. HRMS (ESI) calcd for $C_{24}H_{22}O_2Na$ $[M+Na]^+$ 365.1517, found 365.1517.

Compound **3z** was prepared following the **Typical Procedure E**

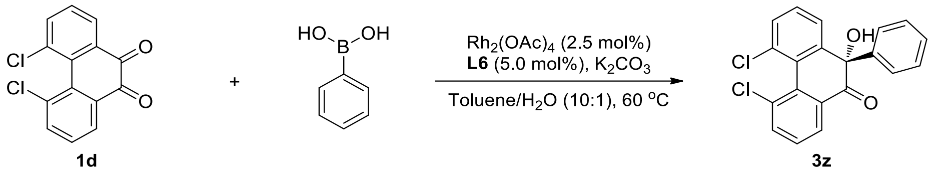

The reaction of **1d** (110.8 mg, 0.40 mmol, 1.0 equiv), phenylboronic acid (146.3 mg, 1.2 mmol, 2.5 equiv), $Rh_2(OAc)_4$ (4.4 mg, 2.5 mol%), **L6** (14.8 mg, 5.0 mol%) and K_2CO_3 (55.3 mg, 0.40 mmol, 1.0 equiv) in toluene (3 mL) and deionized water (0.3 mL) at 60 °C for 18 h afforded **3z** (133.8 mg, 94%, 92% ee). $[\alpha]_D^{20}$ – 275 (c 1.00, $CHCl_3$). HPLC conditions: Chiralcel AD-H, isopropanol/hexane = 10:90, flow: 1.0 mL/min, λ = 254 nm. **^{1}H NMR** (500 MHz, $CDCl_3$) δ 7.84 (d, *J* = 7.5 Hz, 1H), 7.56 (d, *J* = 8.0 Hz, 1H), 7.53 (d, *J* = 8.0 Hz, 1H), 7.50 (d, *J* = 7.5 Hz, 1H),7.49 – 7.44 (m, 1H), 7.25 – 7.19 (m, 1H), 7.18 – 7.14 (m, 1H), 7.14 – 7.12 (m, 2H), 7.11 – 7.05 (m, 2H), 4.75 (s, 1H). **^{13}C NMR** (126 MHz, $CDCl_3$) δ 199.3, 139.9, 138.1, 135.8, 134.3, 133.2, 132.7, 131.9, 130.9, 130.3, 129.7, 129.6, 128.5, 128.4, 126.4, 125.1, 124.8, 81.2. HRMS (ESI) calcd for $C_{20}H_{12}Cl_2O_2Na$ $[M+Na]^+$ 377.0112, found 377.0108.

Compound **3aa** was prepared following the **Typical Procedure E**

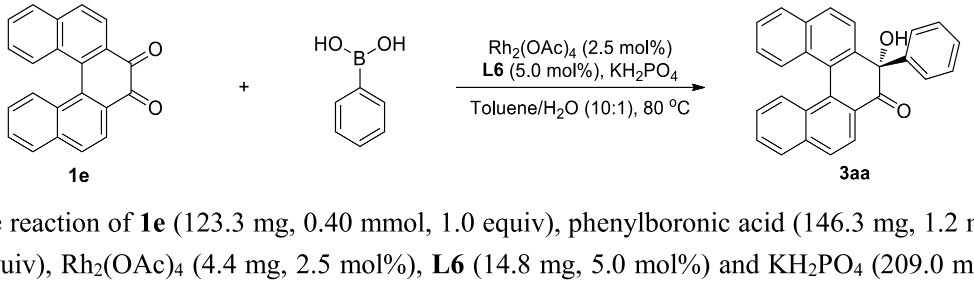

The reaction of **1e** (123.3 mg, 0.40 mmol, 1.0 equiv), phenylboronic acid (146.3 mg, 1.2 mmol, 3.0 equiv), $Rh_2(OAc)_4$ (4.4 mg, 2.5 mol%), **L6** (14.8 mg, 5.0 mol%) and KH_2PO_4 (209.0 mg, 1.2 mmol, 3.0 equiv) in toluene (3 mL) and deionized water (0.3 mL) at 80 °C for 12 h afforded **3aa** (61.9 mg, 40%, 94% ee). $[\alpha]_D^{20}$ – 379 (c 0.77, $CHCl_3$). HPLC conditions: Chiralcel AD-H,

isopropanol/hexane = 10:90, flow: 1.0 mL/min, λ = 254 nm. **^{1}H NMR** (500 MHz, $CDCl_3$) δ 8.10 – 8.03 (m, 2H), 7.95 (d, J = 8.0 Hz, 1H), 7.84 (d, J = 8.5 Hz, 1H), 7.82 – 7.77 (m, 2H), 7.73 (d, J = 8.5 Hz, 1H), 7.60 – 7.54 (m, 1H), 7.53 – 7.47 (m, 1H), 7.42 (d, J = 8.5 Hz, 1H), 7.38 – 7.31 (m, 1H), 7.30 – 7.25 (m, 1H), 7.25 – 7.21 (m, 2H), 7.06 – 7.02 (m, 2H), 7.02 – 6.99 (m, 1H), 4.91 (s, 1H). **^{13}C NMR** (126 MHz, $CDCl_3$) δ 201.9, 140.6, 137.0, 136.8, 135.3, 134.1, 130.1, 129.84, 129.77, 129.1, 128.6, 128.5, 128.4, 128.3, 128.1, 127.4, 127.2, 126.2, 126.0, 125.9, 125.6, 124.1, 122.1, 81.3. HRMS (ESI) calcd for $C_{28}H_{18}O_2Na$ $[M+Na]^+$ 409.1204, found 409.1212.

Compound **3bb** was prepared following the **Typical Procedure E**

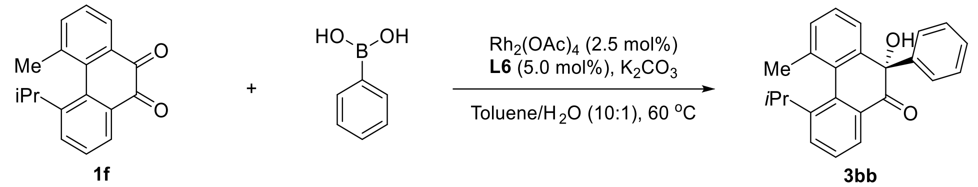

The reaction of **1f** (105.7 mg, 0.40 mmol, 1.0 equiv), phenylboronic acid (122.0 mg, 1.0 mmol, 2.5 equiv), $Rh_2(OAc)_4$ (4.4 mg, 2.5 mol%), **L6** (14.8 mg, 5.0 mol%) and K_2CO_3 (55.3 mg, 0.40 mmol, 1.0 equiv) in toluene (3 mL) and deionized water (0.3 mL) at 60 °C for 12 h afforded **3bb** (135.5 mg, 99%, rr = 2:1). **^{1}H NMR** (500 MHz, $CDCl_3$) δ 7.81 (d, J = 7.5 Hz, 2H), 7.78 – 7.72 (m, 1H), 7.53 – 7.46 (m, 6H), 7.45 – 7.38 (m, 3H), 7.36 – 7.31 (m, 3H), 7.29 – 7.23 (m, 3H), 7.15 – 7.05 (m, 15H), 4.77 (s, 1H), 4.72 (s, 2H), 3.17 (p, J = 6.5 Hz, 2H), 3.10 (p, J = 6.5 Hz, 1H), 2.36 (s, 3H), 2.29 (s, 6H), 1.53 (d, J = 6.5 Hz, 3H), 1.47 (d, J = 6.5 Hz, 6H), 0.77 (d, J = 6.5 Hz, 3H), 0.66 (d, J = 6.5 Hz, 6H). HRMS (ESI) calcd for $C_{24}H_{22}O_2Na$ $[M+Na]^+$ 365.1512, found 365.1503.

Compound **3cc** was prepared following the **Typical Procedure E**

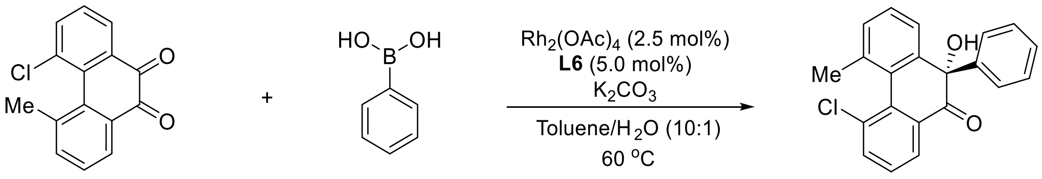

The reaction of **1g** (102.7 mg, 0.40 mmol, 1.0 equiv), Phenylboronic acid (122.0 mg, 1.0 mmol, 2.5 equiv), $Rh_2(OAc)_4$ (4.4 mg, 2.5 mol%), **L6** (14.8 mg, 5.0 mol%) and K_2CO_3 (55.3 mg, 0.40 mmol, 1.0 equiv) in toluene (3 mL) and deionized water (0.3 mL) at 60 °C for 12 h afforded **3cc** (133.1 mg, 99%, rr = 3:1). **^{1}H NMR** (500 MHz, $CDCl_3$) δ 7.86 (d, J = 7.5 Hz, 3H), 7.75 (d, J = 7.5 Hz, 1H), 7.54 (d, J = 8.0 Hz, 3H), 7.52 – 7.49 (m, 2H), 7.49 – 7.45 (m, 4H), 7.45 – 7.40 (m, 4H), 7.40 – 7.36 (m, 3H), 7.23 – 7.18 (m, 3H), 7.18 – 7.15 (m, 1H), 7.15 – 7.10 (m, 14H), 7.10 – 7.05 (m, 6H), 4.85 (s, 3H), 4.72 (s, 1H), 2.46 (s, 9H), 2.43 (s, 3H). HRMS (ESI) calcd for $C_{21}H_{15}ClO_2Na$ $[M+Na]^+$ 357.0653, found 357.0653.

Compound **3dd** was prepared following the **Typical Procedure E**

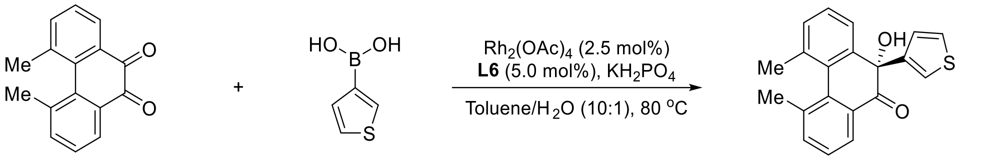

1a **3dd**

The reaction of **1a** (94.5 mg, 0.40 mmol, 1.0 equiv), 3-Thiopheneboronic acid (153.5 mg, 1.2 mmol, 3.0 equiv), $Rh_2(OAc)_4$ (4.4 mg, 2.5 mol%), **L6** (14.8 mg, 5.0 mol%) and $K_2HPO_4 \cdot 3H_2O$ (91.3 mg, 0.40 mmol, 1.0 equiv) in toluene (3 mL) and deionized water (0.3 mL) at 80 °C for 12 h afforded **3dd** (69.3 mg, 54%, 86% ee). $[\alpha]_D^{20}$ – 188 (c 1.24, $CHCl_3$). HPLC conditions: Chiralcel AD-H, isopropanol/hexane = 10:90, flow: 1.0 mL/min, λ = 254 nm. ^{1}**H NMR** (500 MHz, $CDCl_3$) δ 7.73 (dd, *J* = 7.5, 1.5 Hz, 1H), 7.52 (dd, *J* = 7.5, 1.5 Hz, 1H), 7.44 (dd, *J* = 7.5, 1.5 Hz, 1H), 7.40 – 7.36 (m, 1H), 7.33 (dd, *J* = 8.0, 1.5 Hz, 1H), 7.25 – 7.21 (m, 1H), 7.03 (dd, *J* = 5.0, 3.0 Hz, 1H), 6.80 (dd, *J* = 3.0, 1.5 Hz, 1H), 6.73 (dd, *J* = 5.0, 1.5 Hz, 1H), 4.70 (s, 1H), 2.33 (s, 3H), 2.27 (s, 3H). ^{13}**C NMR** (151 MHz, $CDCl_3$) δ 200.7, 143.3, 137.2, 136.8, 136.6, 136.0, 135.5, 131.3, 131.2, 130.4, 128.3, 127.6, 125.9, 125.7, 124.3, 123.3, 122.9, 78.5, 20.6, 20.4. HRMS (ESI) calcd for $C_{20}H_{16}SO_2Na$ $[M+Na]^+$ 343.0763, found 343.0766.

Compound **3ee** was prepared following the **Typical Procedure E**

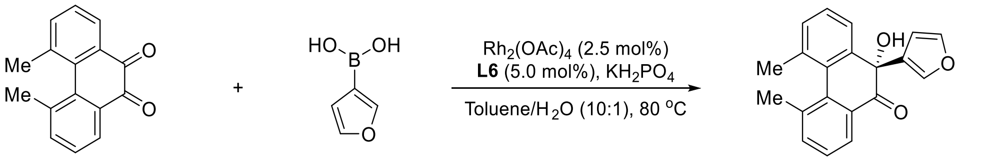

1a **3ee**

The reaction of **1a** (94.5 mg, 0.40 mmol, 1.0 equiv), 3-Thiopheneboronic acid (134.3 mg, 1.2 mmol, 3.0 equiv), $Rh_2(OAc)_4$ (4.4 mg, 2.5 mol%), **L6** (14.8 mg, 5.0 mol%) and $K_2HPO_4 \cdot 3H_2O$ (91.3 mg, 0.40 mmol, 1.0 equiv) in toluene (3 mL) and deionized water (0.3 mL) at 80 °C for 12 h afforded **3ee** (20.6 mg, 17%, 73% ee). $[\alpha]_D^{20}$ – 115 (c 1.00, $CHCl_3$). HPLC conditions: Chiralcel AD-H, isopropanol/hexane = 10:90, flow: 1.0 mL/min, λ = 254 nm. ^{1}**H NMR** (500 MHz, $CDCl_3$) δ 7.70 (dd, *J* = 7.5, 1.5 Hz, 1H), 7.56 (dd, *J* = 7.5, 1.5 Hz, 1H), 7.49 (dd, *J* = 7.5, 1.5 Hz, 1H), 7.38 – 7.33 (m, 1H), 7.32 (dd, *J* = 8.0, 1.5 Hz, 1H), 7.30 – 7.27 (m, 1H), 7.12 – 7.10 (m, 1H), 6.88 – 6.85 (m, 1H), 6.01 (dd, *J* = 2.0, 1.0 Hz, 1H), 4.52 (s, 1H), 2.34 (s, 3H), 2.26 (s, 3H). ^{13}**C NMR** (151 MHz, $CDCl_3$) δ 200.8, 143.0, 140.3, 137.3, 136.8, 136.6, 136.1, 135.6, 131.2, 130.9, 130.3, 128.7, 128.3, 127.6, 124.4, 123.0, 108.7, 75.8, 20.6, 20.4. HRMS (ESI) calcd for $C_{20}H_{17}O_3$ $[M+H]^+$ 305.1172, found 305.1176.

Compound **3ff** was prepared following the **Typical Procedure E**

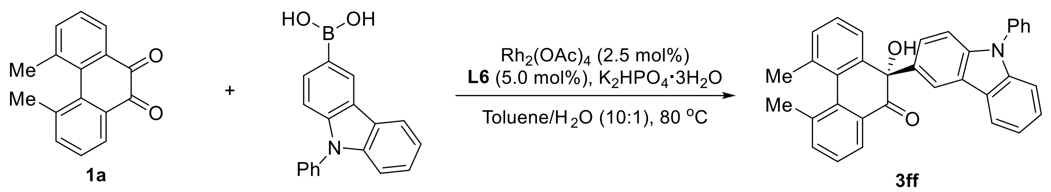

The reaction of **1a** (94.5 mg, 0.40 mmol, 1.0 equiv), 9-Phenyl-9H-carbazol-3-ylboronic acid (344.5 mg, 1.2 mmol, 3.0 equiv), $Rh_2(OAc)_4$ (4.4 mg, 2.5 mol%), **L6** (14.8 mg, 5.0 mol%) and $K_2HPO_4·3H_2O$ (91.3 mg, 0.40 mmol, 1.0 equiv) in toluene (3 mL) and deionized water (0.3 mL) at 80 °C for 12 h afforded **3ff** (186.1 mg, 97%, 84% ee). $[\alpha]_D^{20}$ – 139 (c 1.00, $CHCl_3$). HPLC conditions: Chiralcel AD-H, isopropanol/hexane = 10:90, flow: 1.0 mL/min, λ = 254 nm. **^{1}H NMR** (500 MHz, $CDCl_3$) δ 7.95 – 7.91 (m, 1H), 7.85 (dd, *J* = 7.5, 1.5 Hz, 1H), 7.83 (d, *J* = 2.0 Hz, 1H), 7.57 – 7.51 (m, 2H), 7.49 – 7.46 (m, 1H), 7.46 – 7.44 (m, 2H), 7.44 – 7.42 (m, 2H), 7.42 – 7.40 (m, 1H), 7.36 – 7.31 (m, 2H), 7.30 (dd, *J* = 8.0, 1.5 Hz, 1H), 7.23 – 7.19 (m, 2H), 7.17 – 7.13 (m, 1H), 7.13 – 7.09 (m, 1H), 4.90 (s, 1H), 2.37 (s, 3H), 2.33 (s, 3H). **^{13}C NMR** (126 MHz, $CDCl_3$) δ 201.8, 141.0, 140.3, 137.3, 137.1, 136.6, 136.2, 135.9, 135.3, 133.0, 132.3, 131.2, 130.7, 129.8, 128.4, 127.45, 127.42, 126.8, 125.9, 124.6, 124.2, 124.1, 123.1, 123.0, 120.1, 119.9, 118.6, 109.7, 109.5, 81.4, 20.6, 20.3. HRMS (ESI) calcd for $C_{34}H_{25}NO_2Na$ $[M+Na]^+$ 502.1778, found 502.1770.

Typical Procedure for based-promoted oxidative ring-opening reaction (Typical Procedure F)

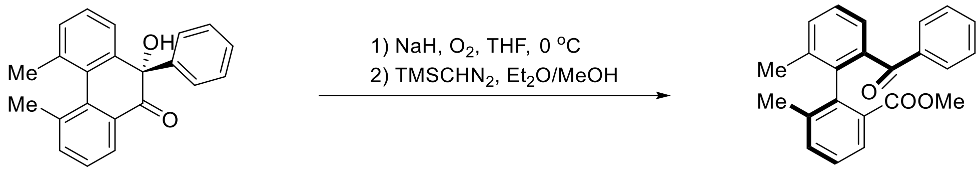

Under oxygen atmosphere, a solution of **3a** (62.8 mg, 0.20 mmol, 1.0 equiv) in THF (4 mL) was added NaH (60% in mineral oil, 32.0 mg, 0.80 mmol, 4.0 equiv) at 0 °C, then the mixture was stirred at the same temperature for 3 h. The reaction was restored to room temperature, 1M HCl (1 mL) was added and stirred for 10 min. The mixture was diluted with H_2O and extracted with EtOAc for three times. The combined organic layer was washed with brine, dried over anhydrous Na_2SO_4, filtered and concentrated to afford crude acid, which was used in next step without further purification.

Under nitrogen atmosphere, to a mixture of the above crude product in anhydrous Et_2O (1 mL) and MeOH (1 mL) was added $TMSCHN_2$ (2.0 M in hexane, 0.50 mL, 1.0 mmol) slowly at room temperature and stirred for 2 h. The solvent was removed and the residue was purified by flash chromatography on silica gel (PE/EtOAc = 13:1) to afford **4a** (63.7 mg, 93%, 96% ee). $[\alpha]_D^{20}$ – 15.1 (c 1.00, $CHCl_3$). HPLC conditions: Chiralcel AD-H, isopropanol/hexane = 3:97, flow: 1.0 mL/min, λ = 254 nm. **^{1}H NMR** (500 MHz, $CDCl_3$) δ 7.78 – 7.74 (m, 1H), 7.72 – 7.67 (m, 2H), 7.54 – 7.49 (m, 1H), 7.49 – 7.46 (m, 1H), 7.43 – 7.40 (m, 1H), 7.40 – 7.37 (m, 2H), 7.37 – 7.35 (m, 1H), 7.33 – 7.30 (m, 1H), 7.28 (d, *J* = 7.5 Hz, 1H), 3.61 (s, 3H), 2.07 (s, 3H), 2.06 (s, 3H). **^{13}C NMR** (126 MHz, $CDCl_3$) δ 197.3, 167.1, 139.7, 139.4, 138.1, 137.8, 137.2, 137.1, 133.5, 132.5, 131.7, 130.2, 129.4, 127.9, 127.7, 127.2, 126.5, 126.1, 51.7, 20.3, 19.9. HRMS (ESI) calcd for $C_{23}H_{20}O_3Na$ $[M+Na]^+$ 367.1310, found 367.1314.

Compound **4b** was prepared following the **Typical Procedure F**

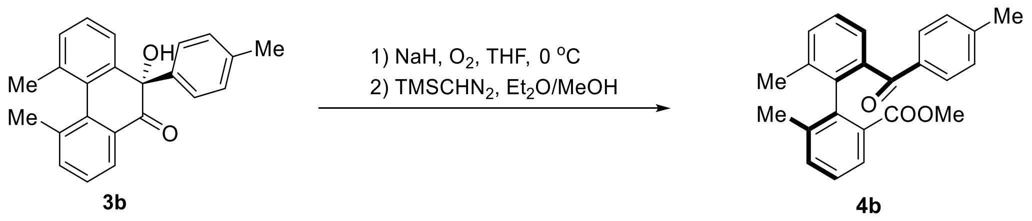

The reaction of **3b** (65.7 mg, 0.20 mmol, 1.0 equiv) and NaH (60% in mineral oil, 32.0 mg, 0.80 mmol, 4.0 equiv) in THF (4 mL) at 0 °C for 3 h afforded crude acid, which was used in next step without further purification.

The reaction of the above crude product and $TMSCHN_2$ (2.0 M in hexane, 0.50 mL, 1.0 mmol) in anhydrous Et_2O (1 mL) and MeOH (1 mL) at room temperature afforded **4b** (65.2 mg, 91%, 94% ee). $[\alpha]_D^{20}$ + 1.65 (c 1.00, $CHCl_3$). HPLC conditions: Chiralcel AD-H, isopropanol/hexane = 2:98, flow: 1.0 mL/min, λ = 254 nm. **^{1}H NMR** (500 MHz, $CDCl_3$) δ 7.76 (d, *J* = 7.5 Hz, 1H), 7.65 – 7.58 (m, 2H), 7.47 (d, *J* = 7.5 Hz, 1H), 7.41 (d, *J* = 7.5 Hz, 1H), 7.38 – 7.33 (m, 1H), 7.31 – 7.27 (m, 2H), 7.22 – 7.16 (m, 2H), 3.61 (s, 3H), 2.41 (s, 3H), 2.08 (s, 3H), 2.06 (s, 3H). **^{13}C NMR** (126 MHz, $CDCl_3$) δ 196.9, 167.2, 143.4, 139.7, 139.3, 138.1, 137.4, 137.1, 135.1, 133.5, 131.5, 130.4, 129.5, 128.6, 127.6, 127.1, 126.3, 126.0, 51.7, 21.6, 20.3, 19.9. HRMS (ESI) calcd for $C_{24}H_{22}O_3Na$ $[M+Na]^+$ 381.1467, found 381.1476.

Compound **4c** was prepared following the **Typical Procedure F**

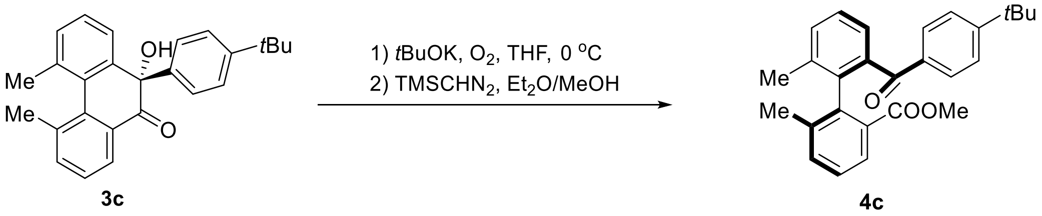

The reaction of **3c** (74.1 mg, 0.20 mmol, 1.0 equiv) and *t*BuOK (89.8 mg, 0.80 mmol, 4.0 equiv) in THF (4 mL) at 0 °C for 3 h afforded crude acid, which was used in next step without further purification.

The reaction of the above crude product and $TMSCHN_2$ (2.0 M in hexane, 0.50 mL, 1.0 mmol) in anhydrous Et_2O (1 mL) and MeOH (1 mL) at room temperature afforded **4c** (68.1 mg, 85%, 94% ee). $[\alpha]_D^{20}$ + 3.73 (c 1.25, $CHCl_3$). HPLC conditions: Chiralcel ID, isopropanol/hexane = 5:95, flow: 1.0 mL/min, λ = 254 nm. **^{1}H NMR** (500 MHz, $CDCl_3$) δ 7.77 (d, *J* = 7.8 Hz, 1H), 7.68 – 7.64 (m, 2H), 7.48 (d, *J* = 7.2 Hz, 1H), 7.43 – 7.41 (m, 2H), 7.41 – 7.39 (m, 1H), 7.39 – 7.35 (m, 1H), 7.35 – 7.32 (m, 1H), 7.28 (d, *J* = 7.5 Hz, 1H), 3.62 (s, 3H), 2.09 (s, 3H), 2.07 (s, 3H), 1.36 (s, 9H). **^{13}C NMR** (126 MHz, $CDCl_3$) δ 196.9, 167.1, 156.1, 139.8, 139.3, 138.0, 137.3, 137.0, 135.1, 133.5, 131.5, 130.1, 129.5, 127.9, 127.1, 126.4, 126.0, 124.8, 51.6, 35.0, 31.0, 20.3, 19.9. HRMS (ESI) calcd for $C_{27}H_{28}O_3Na$ $[M+Na]^+$ 423.1936, found 423.1938.

Compound **4d** was prepared following the **Typical Procedure F**

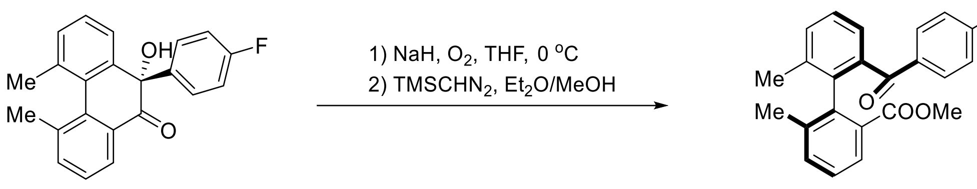

3d **4d**

The reaction of **3d** (66.5 mg, 0.20 mmol, 1.0 equiv) and NaH (60% in mineral oil, 32 mg, 0.80 mmol, 4.0 equiv) in THF (4 mL) at 0 °C for 3 h afforded crude acid, which was used in next step without further purification.

The reaction of the above crude product and $TMSCHN_2$ (2.0 M in hexane, 0.50 mL, 1.0 mmol) in anhydrous Et_2O (1 mL) and MeOH (1 mL) at room temperature afforded **4d** (64.4 mg, 89%, 94% ee). $[\alpha]_D^{20}$ – 1.49 (c 1.05, $CHCl_3$). HPLC conditions: Chiralcel AD-H, isopropanol/hexane = 3:97, flow: 1.0 mL/min, λ = 254 nm. **^{1}H NMR** (500 MHz, $CDCl_3$) δ 7.75 – 7.72 (m, 1H), 7.72 – 7.66 (m, 2H), 7.45 (d, *J* = 7.5 Hz, 1H), 7.39 (d, *J* = 7.5 Hz, 1H), 7.36 – 7.31 (m, 1H), 7.27 – 7.22 (m, 2H), 7.04 – 6.98 (m, 2H), 3.58 (s, 3H), 2.03 (s, 3H), 2.01 (s, 3H). **^{13}C NMR** (126 MHz, $CDCl_3$) δ 195.9, 167.1, 165.4 (d, *J* = 255 Hz) 139.6, 139.3, 138.2, 137.2, 137.1, 134.1 (d, *J* = 2.8 Hz), 133.6, 132.8 (d, *J* = 9.3 Hz), 131.8, 129.3, 127.7, 127.3, 126.2, 115.0 (d, *J* = 21.8 Hz), 51.7, 20.2, 19.9. **^{19}F NMR** (471 MHz, $CDCl_3$) δ -105.77. HRMS (ESI) calcd for $C_{23}H_{19}FO_3Na$ $[M+Na]^+$ 385.1216, found 385.1217.

Compound **4e** was prepared following the **Typical Procedure F**

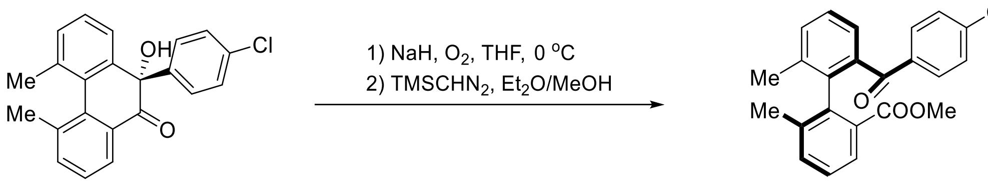

3e **4e**

The reaction of **3e** (69.8 mg, 0.20 mmol, 1.0 equiv) and NaH (60% in mineral oil, 32 mg, 0.80 mmol, 4.0 equiv) in THF (4 mL) at 0 °C for 3 h afforded crude acid, which was used without further purification.

The reaction of the above crude product and $TMSCHN_2$ (2.0 M in hexane, 0.50 mL, 1.0 mmol) in anhydrous Et_2O (1 mL) and MeOH (1 mL) at room temperature afforded **4e** (69.0 mg, 91%, 93% ee). $[\alpha]_D^{20}$ + 17.1 (c 1.00, $CHCl_3$). HPLC conditions: Chiralcel AD-H, isopropanol/hexane = 3:97, flow: 1.0 mL/min, λ = 254 nm. **^{1}H NMR** (500 MHz, $CDCl_3$) δ 7.74 (d, *J* = 7.5 Hz, 1H), 7.64 – 7.58 (m, 2H), 7.45 (d, *J* = 7.5 Hz, 1H), 7.39 (d, *J* = 7.5 Hz, 1H), 7.36 – 7.33 (m, 1H), 7.33 – 7.32 (m, 1H), 7.32 – 7.30 (m, 1H), 7.28 – 7.23 (m, 2H), 3.59 (s, 3H), 2.03 (s, 3H), 2.01 (s, 3H). **^{13}C NMR** (126 MHz, $CDCl_3$) δ 196.2, 167.1, 139.6, 139.4, 139.0, 138.2, 137.2, 136.9, 136.1, 133.6, 132.0, 131.6, 129.2, 128.2, 127.7, 127.3, 126.3, 126.2, 51.7, 20.2, 19.9. HRMS (ESI) calcd for $C_{23}H_{19}ClO_3Na$ $[M+Na]^+$ 401.0920, found 401.0920.

Compound **4f** was prepared following the **Typical Procedure F**

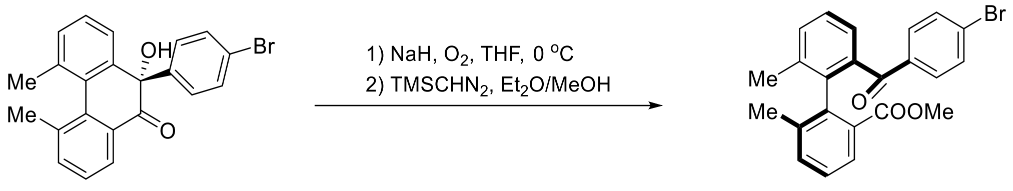

The reaction of **3f** (78.7 mg, 0.20 mmol, 1.0 equiv) and NaH (60% in mineral oil, 32 mg, 0.80 mmol, 4.0 equiv) in THF (4 mL) at 0 °C for 3 h afforded crude acid, which was used in next step without further purification.

The reaction of the above crude product and $TMSCHN_2$ (2.0 M in hexane, 0.50 mL, 1.0 mmol) in anhydrous Et_2O (1 mL) and MeOH (1 mL) at room temperature afforded **4e** (77.1 mg, 91%, 94% ee). $[\alpha]_D^{20}$ + 22.2 (c 1.46, $CHCl_3$). HPLC conditions: Chiralcel AD-H, isopropanol/hexane = 3:97, flow: 1.0 mL/min, λ = 254 nm. **^{1}H NMR** (500 MHz, $CDCl_3$) δ 7.74 (d, J = 7.8 Hz, 1H), 7.55 – 7.51 (m, 2H), 7.50 – 7.47 (m, 2H), 7.45 (d, J = 7.5 Hz, 1H), 7.39 (d, J = 7.5 Hz, 1H), 7.36 – 7.30 (m, 1H), 7.28 – 7.23 (m, 2H), 3.58 (s, 3H), 2.02 (s, 3H), 2.01 (s, 3H).**^{13}C NMR** (126 MHz, $CDCl_3$) δ 196.3, 167.0, 139.6, 139.4, 138.2, 137.2, 136.8, 136.5, 133.6, 132.0, 131.6, 131.2, 129.2, 127.7, 127.3, 126.3, 126.2, 51.7, 20.2, 19.8. HRMS (ESI) calcd for $C_{23}H_{19}BrO_3Na$ $[M+Na]^+$ 445.0415, found 445.0416.

Compound **4g** was prepared following the **Typical Procedure F**

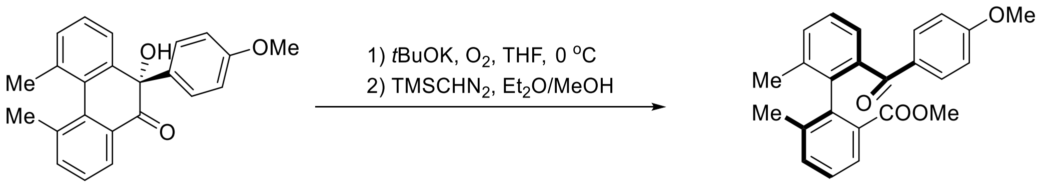

The reaction of **3g** (68.9 mg, 0.20 mmol, 1.0 equiv) and *t*BuOK (89.8 mg, 0.80 mmol, 4.0 equiv) in THF (4 mL) at 0 °C for 3 h afforded crude acid, which was used in next step without further purification.

The reaction of the above crude product and $TMSCHN_2$ (2.0 M in hexane, 0.50 mL, 1.0 mmol) in anhydrous Et_2O (1 mL) and MeOH (1 mL) at room temperature afforded **4g** (62.8 mg, 84%, 87% ee). $[\alpha]_D^{20}$ + 6.02 (c 1.00, $CHCl_3$). HPLC conditions: Chiralcel AD-H, isopropanol/hexane = 3:97, flow: 1.0 mL/min, λ = 254 nm. **^{1}H NMR** (400 MHz, $CDCl_3$) δ 7.77 (d, J = 7.6 Hz, 1H), 7.74 – 7.68 (m, 2H), 7.47 (d, J = 7.6 Hz, 1H), 7.42 (d, J = 7.6 Hz, 1H), 7.39 – 7.34 (m, 1H), 7.32 – 7.26 (m, 2H), 6.92 – 6.82 (m, 2H), 3.86 (s, 3H), 3.62 (s, 3H), 2.09 (s, 3H), 2.07 (s, 3H). **^{13}C NMR** (101 MHz, $CDCl_3$) δ 195.9, 167.1, 163.1, 139.7, 139.1, 138.2, 137.5, 137.0, 133.5, 132.5, 131.2, 130.5, 129.4, 127.6, 127.1, 126.0, 113.1, 55.3, 51.6, 20.2, 19.8. HRMS (ESI) calcd for $C_{24}H_{22}O_4Na$ $[M+Na]^+$ 397.1416, found 397.1413.

Compound **4h** was prepared following the **Typical Procedure F**

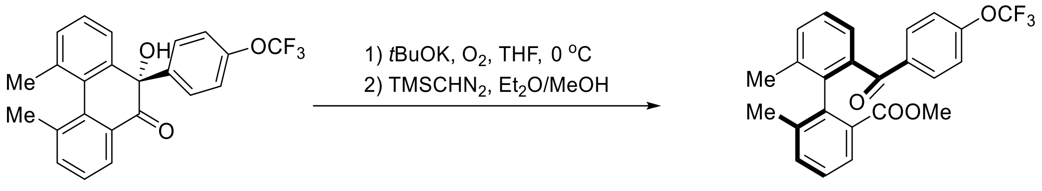

The reaction of **3h** (79.7 mg, 0.20 mmol, 1.0 equiv) and *t*BuOK (89.8 mg, 0.80 mmol, 4.0 equiv) in THF (4 mL) at 0 °C for 3 h afforded crude acid, which was used in next step without further purification.

The reaction of the above crude product and $TMSCHN_2$ (2.0 M in hexane, 0.50 mL, 1.0 mmol) in anhydrous Et_2O (1 mL) and MeOH (1 mL) at room temperature afforded **4h** (71.7 mg, 84%, 95% ee). $[\alpha]_D^{20}$ + 2.43 (c 1.35, $CHCl_3$). HPLC conditions: Chiralcel AD-H, isopropanol/hexane = 1:99, flow: 1.0 mL/min, λ = 254 nm. **^{1}H NMR** (500 MHz, $CDCl_3$) δ 7.78 – 7.75 (m, 1H), 7.75 – 7.71 (m, 2H), 7.49 (d, *J* = 7.5 Hz, 1H), 7.42 (d, *J* = 7.5 Hz, 1H), 7.40 – 7.35 (m, 1H), 7.32 – 7.27 (m, 2H), 7.19 (d, *J* = 8.4 Hz, 2H), 3.61 (s, 3H), 2.06 (s, 3H), 2.04 (s, 3H). **^{13}C NMR** (126 MHz, $CDCl_3$) δ 195.9, 167.0, 152.1, 139.6, 139.4, 138.2, 137.2, 136.8, 136.1, 133.6, 132.1, 132.0, 129.2, 127.7, 127.3, 126.3, 126.2, 120.2 (q, *J* = 259 Hz), 119.8, 51.7, 20.2, 19.8. **^{19}F NMR** (471 MHz, $CDCl_3$) δ -57.6. HRMS (ESI) calcd for $C_{24}H_{19}F_3O_4Na$ $[M+Na]^+$ 451.1133, found 451.1134.

Compound **4i** was prepared following the **Typical Procedure F**

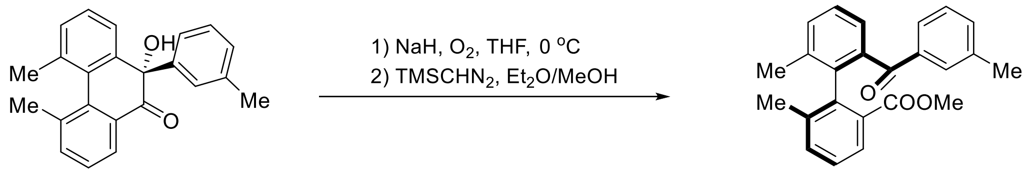

The reaction of **3i** (65.7 mg, 0.20 mmol, 1.0 equiv) and NaH (60% in mineral oil, 32 mg, 0.80 mmol, 4.0 equiv) in THF (4 mL) at 0 °C for 3 h afforded crude acid, which was used in next step without further purification.

The reaction of the above crude product and $TMSCHN_2$ (2.0 M in hexane, 0.50 mL, 1.0 mmol) in anhydrous Et_2O (1 mL) and MeOH (1 mL) at room temperature afforded **4i** (67.4 mg, 94%, 96% ee). $[\alpha]_D^{20}$ – 5.10 (c 1.45, $CHCl_3$). HPLC conditions: Chiralcel IC, isopropanol/hexane = 3:97, flow: 1.0 mL/min, λ = 254 nm. **^{1}H NMR** (500 MHz, $CDCl_3$) δ 7.73 (d, *J* = 7.5 Hz, 1H), 7.49 (s, 1H), 7.46 – 7.40 (m, 2H), 7.37 (d, *J* = 7.5 Hz, 1H), 7.35 – 7.31 (m, 1H), 7.30 – 7.25 (m, 2H), 7.25 – 7.19 (m, 2H), 3.58 (s, 3H), 2.31 (s, 3H), 2.03 (s, 3H), 2.02 (s, 3H). **^{13}C NMR** (126 MHz, $CDCl_3$) δ 197.5, 167.1, 139.7, 139.4, 138.1, 137.8, 137.7, 137.3, 137.0, 133.5, 133.3, 131.6, 130.4, 129.4, 127.70, 127.65, 127.6, 127.1, 126.5, 126.0, 51.6, 21.2, 20.2, 19.9. HRMS (ESI) calcd for $C_{24}H_{22}O_3Na$ $[M+Na]^+$ 381.1467, found 381.1468.

Compound **4j** was prepared following the **Typical Procedure F**

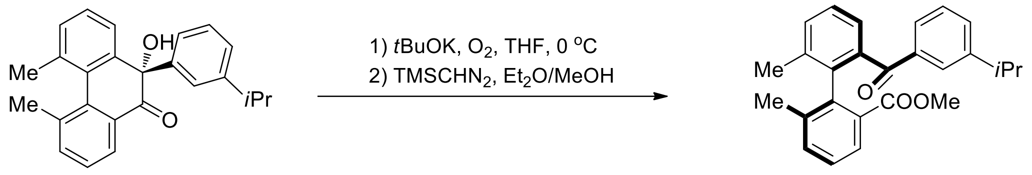

3j **4j**

The reaction of **3j** (71.3 mg, 0.20 mmol, 1.0 equiv) and *t*BuOK (89.8 mg, 0.80 mmol, 4.0 equiv) in THF (4 mL) at 0 °C for 3 h afforded crude acid, which was used in next step without further purification.

The reaction of the above crude product and $TMSCHN_2$ (2.0 M in hexane, 0.50 mL, 1.0 mmol) in anhydrous Et_2O (1 mL) and MeOH (1 mL) at room temperature afforded **4j** (71.5 mg, 92%, 93% ee). $[\alpha]_D^{20}$ – 15.3 (c 1.50, $CHCl_3$). HPLC conditions: Chiralcel IC, isopropanol/hexane = 1:99, flow: 1.0 mL/min, λ = 254 nm. **^{1}H NMR** (500 MHz, $CDCl_3$) δ 7.79 (d, *J* = 8.0 Hz, 1H), 7.62 (s, 1H), 7.50 – 7.46 (m, 2H), 7.41 (d, *J* = 7.5 Hz, 1H), 7.40 – 7.37 (m, 1H), 7.37 – 7.34 (m, 1H), 7.34 – 7.31 (m, 1H), 7.31 – 7.27 (m, 2H), 3.63 (s, 3H), 2.92 (hept, *J* = 6.8 Hz, 1H), 2.07 (s, 3H), 2.05 (s, 3H), 1.25 (s, 3H), 1.23 (s, 3H). **^{13}C NMR** (126 MHz, $CDCl_3$) δ 197.6, 167.1, 148.7, 139.9, 139.6, 138.0, 137.9, 137.3, 136.9, 133.5, 131.7, 130.8, 129.4, 128.2, 127.9, 127.7, 127.6, 127.1, 126.7, 126.0, 51.7, 33.9, 23.8, 23.7, 20.2, 19.9. HRMS (ESI) calcd for $C_{26}H_{26}O_3Na$ $[M+Na]^+$ 409.1780, found 409.1782.

Compound **4k** was prepared following the **Typical Procedure F**

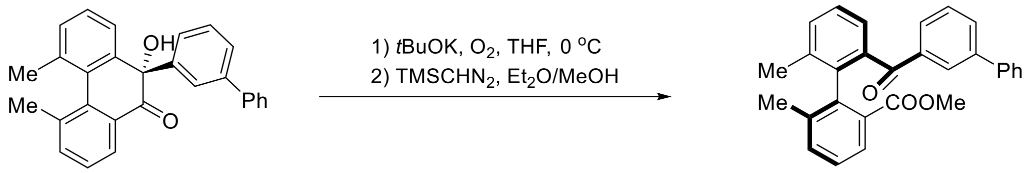

3k **4k**

The reaction of **3k** (78.1 mg, 0.20 mmol, 1.0 equiv) and *t*BuOK (89.8 mg, 0.80 mmol, 4.0 equiv) in THF (4 mL) at 0 °C for 3 h afforded crude acid, which was used in next step without further purification.

The reaction of the above crude product and $TMSCHN_2$ (2.0 M in hexane, 0.50 mL, 1.0 mmol) in anhydrous Et_2O (1 mL) and MeOH (1 mL) at room temperature afforded **4k** (72.6 mg, 86%, 94% ee). $[\alpha]_D^{20}$ + 28.2 (c 1.40, $CHCl_3$). HPLC conditions: Chiralcel IC, isopropanol/hexane = 6:94, flow: 1.0 mL/min, λ = 254 nm. **^{1}H NMR** (500 MHz, $CDCl_3$) δ 7.92 (s, 1H), 7.76 – 7.68 (m, 2H), 7.62 (d, *J* = 7.5 Hz, 1H), 7.57 – 7.51 (m, 2H), 7.48 – 7.43 (m, 1H), 7.42 – 7.39 (m, 2H), 7.39 – 7.36 (m, 2H), 7.35 – 7.33 (m, 2H), 7.32 – 7.29 (m, 1H), 7.25 – 7.21 (m, 1H), 3.50 (s, 3H), 2.05 (s, 3H), 2.02 (s, 3H). **^{13}C NMR** (126 MHz, $CDCl_3$) δ 197.4, 167.0, 140.8, 140.0, 139.8, 139.5, 138.4, 138.2, 137.3, 137.0, 133.6, 131.8, 131.0, 129.3, 128.9, 128.8, 128.7, 128.3, 127.7, 127.6, 127.2, 127.0, 126.5, 126.1, 51.6, 20.3, 19.9. HRMS (ESI) calcd for $C_{29}H_{24}O_3Na$ $[M+Na]^+$ 443.1623, found 443.1627.

Compound **4l** was prepared following the **Typical Procedure F**

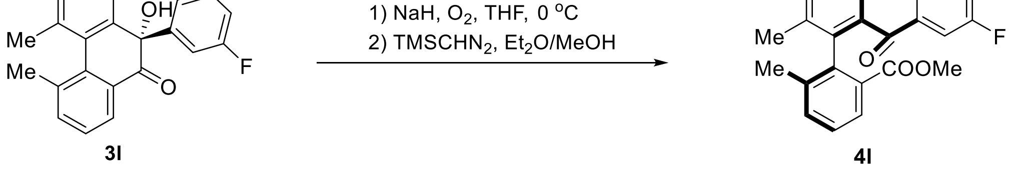

3l **4l**

The reaction of **3l** (66.5 mg, 0.20 mmol, 1.0 equiv) and NaH (60% in mineral oil, 32 mg, 0.80

mmol, 4.0 equiv) in THF (4 mL) at 0 °C for 3 h afforded crude acid, which was used in next step without further purification.

The reaction of the above crude product and $TMSCHN_2$ (2.0 M in hexane, 0.50 mL, 1.0 mmol) in anhydrous Et_2O (1 mL) and MeOH (1 mL) at room temperature afforded **4l** (63.1 mg, 87%, 95% ee). $[\alpha]_D^{20}$ – 1.65 (c 1.40, $CHCl_3$). HPLC conditions: Chiralcel ID, isopropanol/hexane = 2:98, flow: 1.0 mL/min, λ = 254 nm. **^{1}H NMR** (500 MHz, $CDCl_3$) δ 7.73 (d, *J* = 7.5 Hz, 1H), 7.46 (d, *J* = 7.5 Hz, 1H), 7.44 – 7.41 (m, 1H), 7.36 – 7.33 (m, 1H), 7.36 – 7.32 (m, 2H), 7.32 – 7.29 (m, 1H), 7.29 – 7.22 (m, 2H), 7.19 – 7.14 (m, 1H), 3.59 (s, 3H), 2.03 (s, 3H), 2.02 (s, 3H). **^{13}C NMR** (126 MHz, $CDCl_3$) δ 196.1 (d, *J* = 2.1 Hz), 167.0, 162.2 (d, *J* = 248 Hz), 139.9 (d, *J* = 6.2 Hz), 139.5, 139.4, 138.1, 137.2, 136.8, 133.6, 132.0, 129.5 (d, *J* = 7.7 Hz), 129.3, 127.7, 127.3, 126.3, 126.2, 125.8 (d, *J* = 2.9 Hz), 119.5 (d, *J* = 21.5 Hz), 116.6 (d, *J* = 22.3 Hz), 116.5, 51.7, 20.2, 19.8. **^{19}F NMR** (471 MHz, $CDCl_3$) δ -112.4. HRMS (ESI) calcd for $C_{23}H_{19}FO_3Na$ $[M+Na]^+$ 385.1216, found 385.1219.

Compound **4m** was prepared following the **Typical Procedure F**

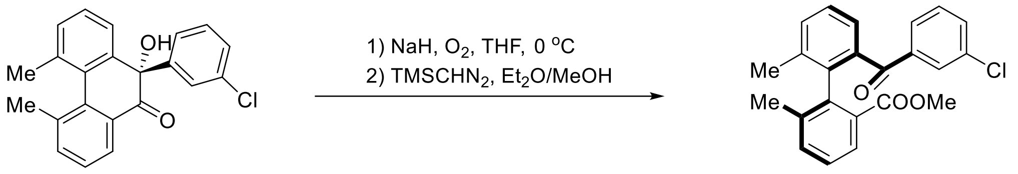

3m **4m**

The reaction of **3m** (69.8 mg, 0.20 mmol, 1.0 equiv) and NaH (60% in mineral oil, 32 mg, 0.80 mmol, 4.0 equiv) in THF (4 mL) at 0 °C for 3 h afforded crude acid, which was used in next step without further purification.

The reaction of the above crude product and $TMSCHN_2$ (2.0 M in hexane, 0.50 mL, 1.0 mmol) in anhydrous Et_2O (1 mL) and MeOH (1 mL) at room temperature afforded **4m** (71.7 mg, 95%, 95% ee). $[\alpha]_D^{20}$ + 11.4 (c 1.55, $CHCl_3$). HPLC conditions: Chiralcel ID, isopropanol/hexane = 0.5:99.5, flow: 1.0 mL/min, λ = 254 nm. **^{1}H NMR** (500 MHz, $CDCl_3$) δ 7.72 (d, *J* = 8.0 Hz, 1H), 7.62 – 7.58 (m, 1H), 7.53 – 7.49 (m, 1H), 7.47 – 7.44 (m, 1H), 7.44 – 7.41 (m, 1H), 7.37 (d, *J* = 7.5 Hz, 1H), 7.36 – 7.31 (m, 1H), 7.28 – 7.25 (m, 2H), 7.24 – 7.23 (m, 1H), 3.59 (s, 3H), 2.02 (s, 3H), 2.00 (s, 3H). **^{13}C NMR** (126 MHz, $CDCl_3$) δ 196.1, 167.1, 139.48, 139.45, 139.4, 138.2, 137.2, 136.7, 134.1, 133.6, 132.4, 132.1, 123.0, 129.2, 128.0, 127.8, 127.3, 126.33, 126.29, 51.8, 20.2, 19.8. HRMS (ESI) calcd for $C_{23}H_{19}ClO_3Na$ $[M+Na]^+$ 401.0920, found 401.0919.

Compound **4n** was prepared following the **Typical Procedure F**

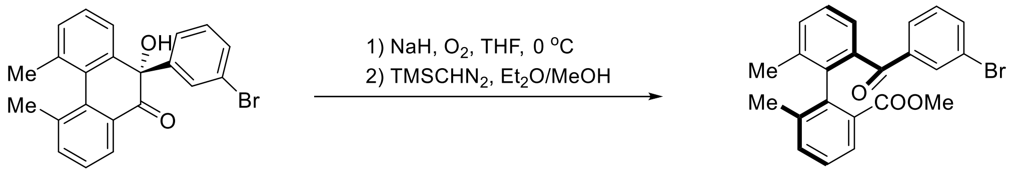

3n **4n**

The reaction of **3n** (78.7 mg, 0.20 mmol, 1.0 equiv) and NaH (60% in mineral oil, 32 mg, 0.80 mmol, 4.0 equiv) in THF (4 mL) at 0 °C for 3 h afforded crude acid, which was used in next step without further purification.

The reaction of the above crude product and $TMSCHN_2$ (2.0 M in hexane, 0.50 mL, 1.0 mmol) in anhydrous Et_2O (1 mL) and MeOH (1 mL) at room temperature afforded **4n** (78.3 mg, 92%, 94% ee). $[\alpha]_D^{20}$ + 15.5 (c 1.56, $CHCl_3$). HPLC conditions: Chiralcel OD, isopropanol/hexane = 1:99,

flow: 1.0 mL/min, λ = 254 nm. **^{1}H NMR** (500 MHz, $CDCl_3$) δ 7.81 – 7.78 (m, 1H), 7.76 (d, *J* = 7.5 Hz, 1H), 7.63 – 7.58 (m, 2H), 7.49 (d, *J* = 7.5 Hz, 1H), 7.41 (d, *J* = 7.5 Hz, 1H), 7.40 – 7.36 (m, 1H), 7.31 – 7.29 (m, 1H), 7.29 – 7.27 (m, 1H), 7.26 – 7.22 (m, 1H), 3.63 (s, 3H), 2.06 (s, 3H), 2.05 (s, 3H). **^{13}C NMR** (126 MHz, $CDCl_3$) δ 196.0, 167.0, 139.7, 139.48, 139.47, 138.2, 137.2, 136.7, 135.3, 133.6, 132.8, 132.1, 129.5, 129.3, 128.5, 127.8, 127.3, 126.34, 126.29, 122.2, 51.8, 20.2, 19.8. HRMS (ESI) calcd for $C_{23}H_{20}BrO_3$ $[M+H]^+$ 423.0596, found 423.0604.

Compound **4o** was prepared following the **Typical Procedure F**

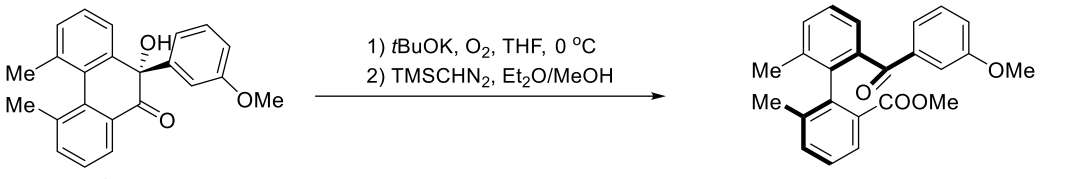

3o **4o**

The reaction of **3o** (68.9 mg, 0.20 mmol, 1.0 equiv) and *t*BuOK (89.8mg, 0.80 mmol, 4.0 equiv) in THF (4 mL) at 0 °C for 3 h afforded crude acid, which was used in next step without further purification.

The reaction of the above crude product and $TMSCHN_2$ (2.0 M in hexane, 0.50 mL, 1.0 mmol) in anhydrous Et_2O (1 mL) and MeOH (1 mL) at room temperature afforded **4o** (69.9 mg, 93%, 83% ee). $[\alpha]_D^{20}$ – 3.95 (c 1.30, $CHCl_3$). HPLC conditions: Chiralcel ID, isopropanol/hexane = 8:92, flow: 1.0 mL/min, λ = 254 nm. **^{1}H NMR** (500 MHz, $CDCl_3$) δ 7.74 (d, *J* = 7.5 Hz, 1H), 7.44 (d, *J* = 7.0 Hz, 1H), 7.38 (d, *J* = 7.5 Hz, 1H), 7.35 – 7.30 (m, 1H), 7.29 – 7.26 (m, 1H), 7.26 – 7.23 (m, 2H), 7.23 – 7.22 (m, 1H), 7.22 – 7.18 (m, 1H), 7.03 (ddd, *J* = 8.0, 2.5, 1.0 Hz, 1H), 3.76 (s, 3H), 3.58 (s, 3H), 2.04 (s, 3H), 2.02 (s, 3H). **^{13}C NMR** (126 MHz, $CDCl_3$) δ 197.1, 167.1, 159.3, 139.7, 139.4, 139.1, 138.1, 137.2, 137.1, 133.5, 131.7, 129.4, 128.7, 127.7, 127.1, 126.4, 126.0, 123.3, 119.4, 113.7, 55.2, 51.7, 20.2, 19.6. HRMS (ESI) calcd for $C_{24}H_{22}O_4Na$ $[M+Na]^+$ 397.1416, found 397.1418.

Compound **4p** was prepared following the **Typical Procedure F**

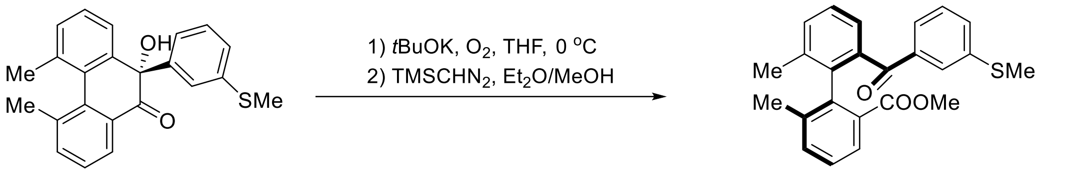

3p **4p**

The reaction of **3p** (72.1 mg, 0.20 mmol, 1.0 equiv) and *t*BuOK (89.8mg, 0.80 mmol, 4.0 equiv) in THF (4 mL) at 0 °C for 3 h afforded crude acid, which was used in next step without further purification.

The reaction of the above crude product and $TMSCHN_2$ (2.0 M in hexane, 0.50 mL, 1.0 mmol) in anhydrous Et_2O (1 mL) and MeOH (1 mL) at room temperature afforded **4p** (59.4 mg, 76%, 89% ee). $[\alpha]_D^{20}$ + 4.04 (c 1.00, $CHCl_3$). HPLC conditions: Chiralcel AD-H, isopropanol/hexane = 1:99, flow: 1.0 mL/min, λ = 254 nm. **^{1}H NMR** (500 MHz, $CDCl_3$) δ 7.74 (d, *J* = 8.0 Hz, 1H), 7.54 (s, 1H), 7.45 (d, *J* = 7.5 Hz, 1H), 7.40 – 7.36 (m, 2H), 7.36 – 7.30 (m, 2H), 7.28 – 7.26 (m, 1H), 7.26 – 7.21 (m, 2H), 3.59 (s, 3H), 2.43 (s, 3H), 2.04 (s, 3H), 2.01 (s, 3H). **^{13}C NMR** (126 MHz, $CDCl_3$) δ 196.9, 167.1, 139.7, 139.5, 139.0, 138.4, 138.1, 137.1, 133.5, 131.9, 130.3, 129.3, 128.1, 127.7, 127.2, 127.1, 126.9, 126.5, 126.1, 51.7, 20.2, 19.8, 15.4. HRMS (ESI) calcd for $C_{24}H_{22}O_3SNa$

[M+Na]+ 413.1187, found 413.1187.

Compound **4q** was prepared following the **Typical Procedure F**

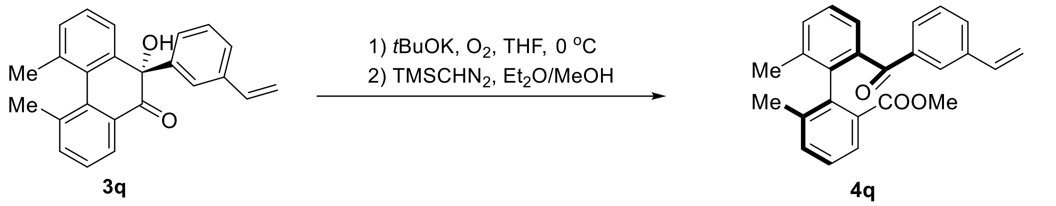

The reaction of **3q** (68.1 mg, 0.20 mmol, 1.0 equiv) and *t*BuOK (89.8mg, 0.80 mmol, 4.0 equiv) in THF (4 mL) at 0 °C for 3 h afforded crude acid, which was used in next step without further purification.

The reaction of the above crude product and $TMSCHN_2$ (2.0 M in hexane, 0.50 mL, 1.0 mmol) in anhydrous Et_2O (1 mL) and MeOH (1 mL) at room temperature afforded **4q** (65.1 mg, 88%, 93% ee). $[\alpha]_D^{20}$ + 11.1 (c 1.20, $CHCl_3$). HPLC conditions: Chiralcel IC, isopropanol/hexane = 2:98, flow: 1.0 mL/min, λ = 254 nm. **^{1}H NMR** (500 MHz, $CDCl_3$) δ 7.77 – 7.69 (m, 2H), 7.56 – 7.50 (m, 2H), 7.46 (d, *J* = 7.5 Hz, 1H), 7.39 (d, *J* = 7.5 Hz, 1H), 7.37 – 7.33 (m, 1H), 7.32 – 7.27 (m, 2H), 7.26 – 7.22 (m, 1H), 6.67 (dd, *J* = 17.5, 11.0 Hz, 1H), 5.74 (d, *J* = 17.5 Hz, 1H), 5.26 (d, *J* = 11.0 Hz, 1H), 3.58 (s, 3H), 2.05 (s, 3H), 2.03 (s, 3H). **^{13}C NMR** (126 MHz, $CDCl_3$) δ 197.3, 167.0, 139.7, 139.4, 138.15, 138.08, 137.3, 137.2, 137.1, 135.9, 133.5, 131.8, 130.1, 129.5, 129.3, 128.0, 127.8, 127.7, 127.2, 126.4, 126.1, 115.0, 51.7, 20.2, 19.8. HRMS (ESI) calcd for $C_{25}H_{22}O_3Na$ [M+Na]+ 393.1467, found 393.1471.

Compound **4r** was prepared following the **Typical Procedure F**

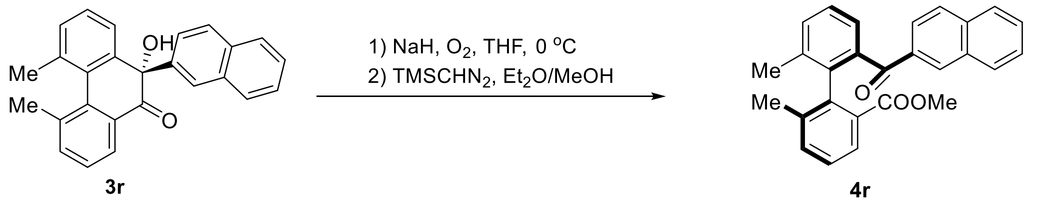

The reaction of **3r** (72.9 mg, 0.20 mmol, 1.0 equiv) and NaH (60% in mineral oil, 32.0 mg, 0.80 mmol, 4.0 equiv) in THF (4 mL) at 0 °C for 3 h afforded crude acid, which was used in next step without further purification.

The reaction of the above crude product and $TMSCHN_2$ (2.0 M in hexane, 0.50 mL, 1.0 mmol) in anhydrous Et_2O (1 mL) and MeOH (1 mL) at room temperature afforded **4r** (67.1 mg, 85%, 93% ee). $[\alpha]_D^{20}$ + 84.7 (c 1.50, $CHCl_3$). HPLC conditions: Chiralcel AD-H, isopropanol/hexane = 3:97, flow: 1.0 mL/min, λ = 254 nm. **^{1}H NMR** (500 MHz, $CDCl_3$) δ 8.16 (s, 1H), 7.88 – 7.82 (m, 2H), 7.82 – 7.78 (m, 2H), 7.70 (d, *J* = 7.5 Hz, 1H), 7.60 – 7.54 (m, 1H), 7.53 – 7.48 (m, 2H), 7.41 – 7.37 (m, 2H), 7.37 – 7.34 (m, 1H), 7.25 – 7.21 (m, 1H), 3.52 (s, 3H), 2.10 (s, 3H), 2.07 (s, 3H). **^{13}C NMR** (126 MHz, $CDCl_3$) δ 197.4, 167.1, 139.7, 139.3, 138.2, 137.5, 137.1, 135.3, 135.0, 133.6, 132.7, 132.0, 131.6, 129.4, 129.2, 128.3, 127.9, 127.69, 127.68, 127.2, 126.5, 126.4, 126.2, 125.2, 51.6, 20.3, 19.9. HRMS (ESI) calcd for $C_{27}H_{22}O_3Na$ [M+Na]+ 417.1467, found 417.1469.

Compound **4s** was prepared following the **Typical Procedure F**

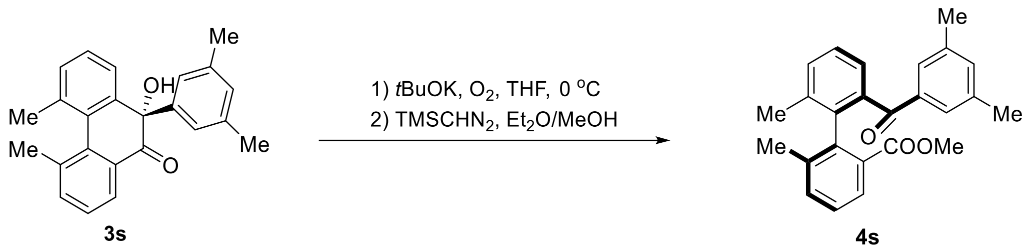

The reaction of **3s** (68.5 mg, 0.20 mmol, 1.0 equiv) and *t*BuOK (89.8 mg, 0.80 mmol, 4.0 equiv) in THF (4 mL) at 0 °C for 3 h afforded crude acid, which was used in next step without further purification.

The reaction of the above crude product and $TMSCHN_2$ (2.0 M in hexane, 0.50 mL, 1.0 mmol) in anhydrous Et_2O (1 mL) and MeOH (1 mL) at room temperature afforded **4s** (70.5 mg, 95%, 92% ee). $[\alpha]_D^{20}$ – 1.41 (c 1.00, $CHCl_3$). HPLC conditions: Chiralcel IC, isopropanol/hexane = 3:97, flow: 1.0 mL/min, λ = 254 nm. **^{1}H NMR** (500 MHz, $CDCl_3$) δ 7.78 (d, *J* = 8.0 Hz, 1H), 7.49 (d, *J* = 7.5 Hz, 1H), 7.41 (d, *J* = 7.5 Hz, 1H), 7.40 – 7.35 (m, 1H), 7.33 – 7.30 (m, 3H), 7.30 – 7.27 (m, 1H), 7.16 (s, 1H), 3.63 (s, 3H), 2.32 (s, 6H), 2.09 (s, 3H), 2.07 (s, 3H). **^{13}C NMR** (126 MHz, $CDCl_3$) δ 197.6, 167.1, 139.8, 139.4, 138.1, 137.8, 137.5, 137.4, 136.9, 134.2, 133.5, 131.5, 129.4, 127.9, 127.6, 127.1, 126.4, 126.0, 51.6, 21.1, 20.2, 19.8. HRMS (ESI) calcd for $C_{25}H_{24}O_3Na$ $[M+Na]^+$ 395.1623, found 395.1621.

Compound **4t** was prepared following the **Typical Procedure F**

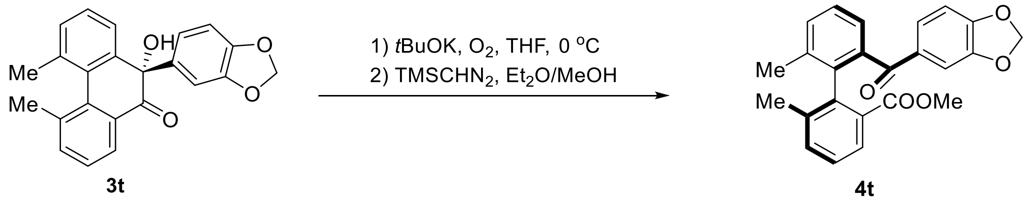

The reaction of **3t** (71.7 mg, 0.20 mmol, 1.0 equiv) and *t*BuOK (89.8 mg, 0.80 mmol, 4.0 equiv) in THF (4 mL) at 0 °C for 3 h afforded crude acid, which was used in next step without further purification.

The reaction of the above crude product and $TMSCHN_2$ (2.0 M in hexane, 0.50 mL, 1.0 mmol) in anhydrous Et_2O (1 mL) and MeOH (1 mL) at room temperature afforded **4t** (67.8 mg, 87%, 87% ee). $[\alpha]_D^{20}$ + 10.2 (c 1.00, $CHCl_3$). HPLC conditions: Chiralcel IC, isopropanol/hexane = 5:95, flow: 1.0 mL/min, λ = 254 nm. **^{1}H NMR** (500 MHz, $CDCl_3$) δ 7.72 (d, *J* = 8.0 Hz, 1H), 7.41 (d, *J* = 7.5 Hz, 1H), 7.37 (d, *J* = 7.5 Hz, 1H), 7.34 – 7.29 (m, 1H), 7.26 – 7.23 (m, 2H), 7.22 – 7.20 (m, 1H), 7.19 – 7.14 (m, 1H), 6.72 (d, *J* = 8.0 Hz, 1H), 5.97 (s, 2H), 3.58 (s, 3H), 2.04 (s, 3H), 2.02 (s, 3H). **^{13}C NMR** (126 MHz, $CDCl_3$) δ 195.5, 167.1, 151.5, 147.6, 139.6, 139.0, 138.2, 137.5, 137.1, 133.5, 132.4, 131.3, 129.4, 127.6, 127.2, 127.2, 126.1, 125.9, 109.4, 107.2, 101.7, 51.7, 20.2, 19.8. HRMS (ESI) calcd for $C_{24}H_{20}O_5Na$ $[M+Na]^+$ 411.1208, found 411.1204.

Compound **4u** was prepared following the **Typical Procedure F**

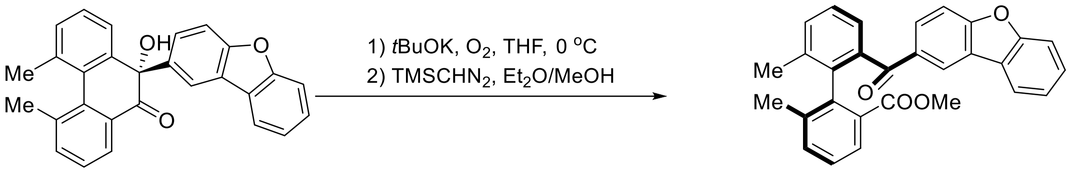

The reaction of **3u** (80.9 mg, 0.20 mmol, 1.0 equiv) and *t*BuOK (89.8 mg, 0.80 mmol, 4.0 equiv) in THF (4 mL) at 0 °C for 3 h afforded crude acid, which was used in next step without further purification.

The reaction of the above crude product and $TMSCHN_2$ (2.0 M in hexane, 0.50 mL, 1.0 mmol) in anhydrous Et_2O (1 mL) and MeOH (1 mL) at room temperature afforded **4u** (77.1 mg, 89%, 90% ee). $[\alpha]_D^{20}$ + 81.1 (c 1.00, $CHCl_3$). HPLC conditions: Chiralcel AD, isopropanol/hexane = 10:90, flow: 1.0 mL/min, λ = 254 nm. **^{1}H NMR** (500 MHz, $CDCl_3$) δ 8.31 (d, *J* = 1.5 Hz, 1H), 7.91 (d, *J* = 7.5 Hz, 1H), 7.86 – 7.80 (m, 1H), 7.69 (d, *J* = 7.5 Hz, 1H), 7.57 (d, *J* = 8.5 Hz, 1H), 7.51 – 7.48 (m, 2H), 7.48 – 7.45 (m, 1H), 7.41 – 7.37 (m, 2H), 7.37 – 7.33 (m, 2H), 7.25 – 7.20 (m, 1H), 3.52 (s, 3H), 2.09 (s, 3H), 2.06 (s, 3H). **^{13}C NMR** (126 MHz, $CDCl_3$) δ 196.8, 167.1, 158.7, 156.8, 139.7, 139.2, 138.4, 137.7, 137.2, 133.6, 133.0, 131.6, 129.8, 129.3, 127.8, 127.7, 127.2, 126.3, 126.1, 124.0, 123.74, 123.68, 123.3, 120.9, 111.8, 111.0, 51.7, 20.3, 19.9. HRMS (ESI) calcd for $C_{29}H_{22}O_4Na$ $[M+Na]^+$ 457.1416, found 457.1414.

Compound **4v** was prepared following the **Typical Procedure F**

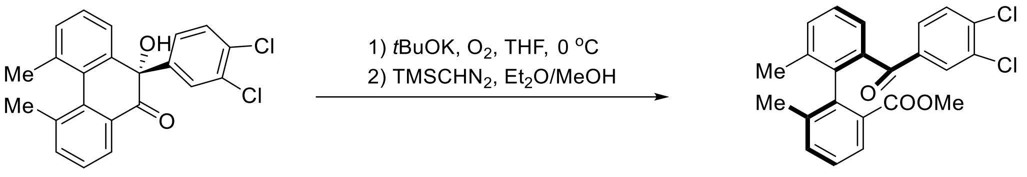

The reaction of **3v** (76.7 mg, 0.20 mmol, 1.0 equiv) and *t*BuOK (89.8 mg, 0.80 mmol, 4.0 equiv) in THF (4 mL) at 0 °C for 3 h afforded crude acid, which was used in next step without further purification.

The reaction of the above crude product and $TMSCHN_2$ (2.0 M in hexane, 0.50 mL, 1.0 mmol) in anhydrous Et_2O (1 mL) and MeOH (1 mL) at room temperature afforded **4v** (48.9 mg, 59%, 95% ee). $[\alpha]_D^{20}$ +36.1 (c 1.00, $CHCl_3$). HPLC conditions: Chiralcel ID, isopropanol/hexane = 1:99, flow: 1.0 mL/min, λ = 254 nm. **^{1}H NMR** (500 MHz, $CDCl_3$) δ 7.75 (d, *J* = 8.0 Hz, 1H), 7.63 (s, 1H), 7.55 (d, *J* = 8.0 Hz, 1H), 7.50 – 7.45 (m, 2H), 7.43 – 7.40 (m, 1H), 7.40 – 7.36 (m, 1H), 7.33 – 7.30 (m, 1H), 7.30 – 7.27 (m, 2H), 3.63 (s, 3H), 2.05 (s, 3H), 2.04 (s, 3H). **^{13}C NMR** (126 MHz, $CDCl_3$) δ 196.2, 167.1, 139.52, 139.48, 138.2, 137.2, 136.8, 134.2, 133.6, 132.5, 132.1, 130.0, 129.3, 128.1, 127.8, 127.3, 126.4, 126.3, 51.8, 20.2, 19.9. HRMS (ESI) calcd for $C_{23}H_{18}Cl_2O_3Na$ $[M+Na]^+$ 435.0531, found 435.0533.

Compound **4x** was prepared following the **Typical Procedure F**

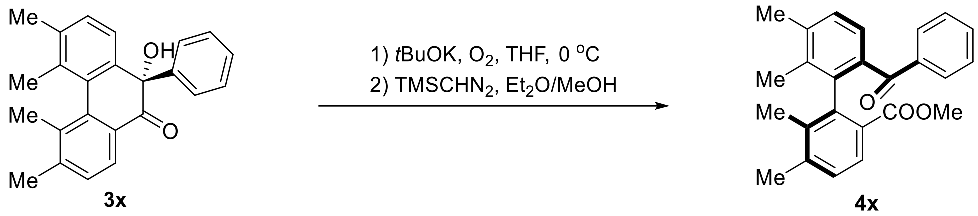

The reaction of **3x** (68.5 mg, 0.20 mmol, 1.0 equiv) and *t*BuOK (89.8 mg, 0.80 mmol, 4.0 equiv) in THF (4 mL) at 0 °C for 3 h afforded crude acid, which was used in next step without further purification.

The reaction of the above crude product and $TMSCHN_2$ (2.0 M in hexane, 0.50 mL, 1.0 mmol) in anhydrous Et_2O (1 mL) and MeOH (1 mL) at room temperature afforded **4x** (67.0 mg, 90%, 87% ee). $[\alpha]_D^{20}$ + 12.7 (c 1.25, $CHCl_3$). HPLC conditions: Chiralcel AD, isopropanol/hexane = 5:95, flow: 1.0 mL/min, λ = 254 nm. **^{1}H NMR** (500 MHz, $CDCl_3$) δ 7.66 – 7.64 (m, 2H), 7.64 – 7.62 (m, 1H), 7.49 – 7.44 (m, 1H), 7.36 – 7.30 (m, 2H), 7.23 – 7.17 (m, 2H), 7.12 (d, *J* = 8.0 Hz, 1H), 3.58 (s, 3H), 2.42 (s, 3H), 2.32 (s, 3H), 1.92 (s, 6H). **^{13}C NMR** (126 MHz, $CDCl_3$) δ 197.6, 167.2, 141.1, 140.3, 140.0, 139.2, 138.2, 136.7, 135.7, 135.4, 132.2, 130.0, 128.6, 127.7, 127.4, 127.34, 127.30, 126.4, 51.6, 21.0, 20.9, 16.5, 16.1. HRMS (ESI) calcd for $C_{25}H_{24}O_3Na$ $[M+Na]^+$ 395.1623, found 395.1614.

Compound **4y** was prepared following the **Typical Procedure F**

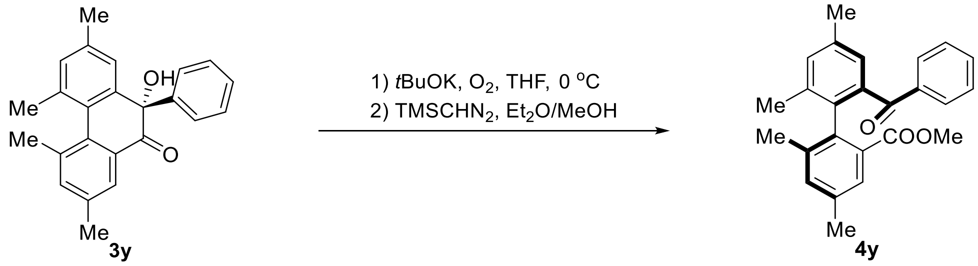

The reaction of **3y** (68.5 mg, 0.20 mmol, 1.0 equiv) and *t*BuOK (89.8 mg, 0.80 mmol, 4.0 equiv) in THF (4 mL) at 0 °C for 3 h afforded crude acid, which was used in next step without further purification.

The reaction of the above crude product and $TMSCHN_2$ (2.0 M in hexane, 0.50 mL, 1.0 mmol) in anhydrous Et_2O (1 mL) and MeOH (1 mL) at room temperature afforded **4y** (70.2 mg, 94%, 93% ee). $[\alpha]_D^{20}$ + 7.15 (c 1.00, $CHCl_3$). HPLC conditions: Chiralcel IC, isopropanol/hexane = 3:97, flow: 1.0 mL/min, λ = 254 nm. **^{1}H NMR** (400 MHz, $CDCl_3$) δ 7.69 – 7.64 (m, 2H), 7.51 (s, 1H), 7.49 – 7.43 (m, 1H), 7.37 – 7.30 (m, 2H), 7.25 (s, 1H), 7.18 (s, 1H), 7.05 (s, 1H), 3.58 (s, 3H), 2.37 (s, 3H), 2.30 (s, 3H), 2.00 (s, 3H), 1.99 (s, 3H). **^{13}C NMR** (101 MHz, $CDCl_3$) δ 197.6, 167.3, 138.1, 137.9, 137.4, 137.1, 136.9, 136.53, 136.49, 135.5, 134.4, 132.5, 132.4, 130.2, 129.4, 128.1, 127.8, 126.9, 51.6, 21.1, 20.9, 20.2, 19.8. HRMS (ESI) calcd for $C_{25}H_{24}O_3Na$ $[M+Na]^+$ 395.1623, found 395.1618.

Compound **4z** was prepared following the **Typical Procedure F**

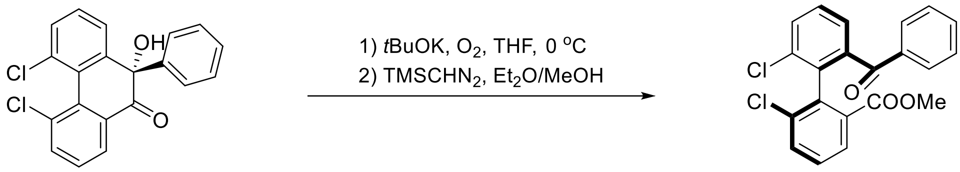

3z **4z**

The reaction of **3z** (71.0 mg, 0.20 mmol, 1.0 equiv) and *t*BuOK (89.8 mg, 0.80 mmol, 4.0 equiv) purification.

The reaction of the above crude product and $TMSCHN_2$ (2.0 M in hexane, 0.50 mL, 1.0 mmol) in anhydrous Et_2O (1 mL) and MeOH (1 mL) at room temperature afforded **4z** (30.8 mg, 40%, 92% ee). $[\alpha]_D^{20}$ – 26.3 (c 0.90, $CHCl_3$). HPLC conditions: Chiralcel IC, isopropanol/hexane = 5:95, flow: 1.0 mL/min, λ = 254 nm. **^{1}H NMR** (500 MHz, $CDCl_3$) δ 7.95 (d, *J* = 8.0 Hz, 1H), 7.73 – 7.69 (m, 2H), 7.69 – 7.65 (m, 1H), 7.56 (d, *J* = 8.0 Hz, 1H), 7.54 – 7.50 (m, 1H), 7.43 – 7.40 (m, 2H), 7.40 – 7.36 (m, 2H), 7.34 (d, *J* = 7.9 Hz, 1H), 3.68 (s, 3H). **^{13}C NMR** (126 MHz, $CDCl_3$) δ 195.6, 165.7, 139.0, 137.5, 137.2, 135.2, 134.9, 133.1, 133.0, 132.1, 131.4, 130.3, 129.0, 128.7, 128.1, 128.0, 127.8, 52.3. HRMS (ESI) calcd for $C_{21}H_{14}Cl_2O_3Na$ $[M+Na]^+$ 407.0218, found 407.0215.

Compound **4aa** was prepared following the **Typical Procedure F**

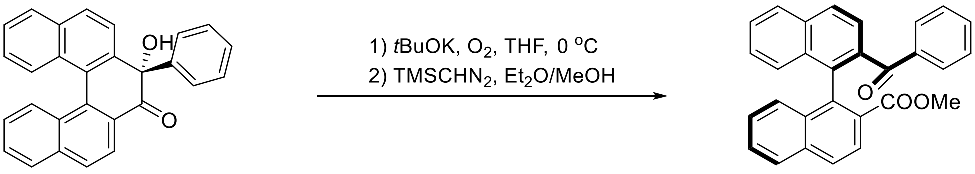

3aa **4aa**

The reaction of **3aa** (77.3 mg, 0.20 mmol, 1.0 equiv) and *t*BuOK (89.8 mg, 0.80 mmol, 4.0 equiv) in THF (4 mL) at 0 °C for 3 h afforded crude acid, which was used in next step without further purification.

The reaction of the above crude product and $TMSCHN_2$ (2.0 M in hexane, 0.50 mL, 1.0 mmol) in anhydrous Et_2O (1 mL) and MeOH (1 mL) at room temperature afforded **4aa** (45.0 mg, 54%, 94% ee). $[\alpha]_D^{20}$ + 21.8 (c 0.67, $CHCl_3$). HPLC conditions: Chiralcel AD-H, isopropanol/hexane = 5:95, flow: 1.0 mL/min, λ = 254 nm. **^{1}H NMR** (500 MHz, $CDCl_3$) δ 8.03 – 8.01 (m, 1H), 8.00 – 7.96 (m, 2H), 7.86 (d, *J* = 8.5 Hz, 1H), 7.84 (d, *J* = 8.0 Hz, 1H), 7.64 (d, *J* = 8.5 Hz, 1H), 7.57 – 7.54 (m, 2H), 7.54 – 7.51 (m, 1H), 7.50 – 7.45 (m, 1H), 7.39 – 7.33 (m, 1H), 7.32 – 7.28 (m, 1H), 7.27 – 7.24 (m, 2H), 7.23 – 7.17 (m, 3H), 3.51 (s, 3H). **^{13}C NMR** (126 MHz, $CDCl_3$) δ 197.6, 167.0, 138.4, 137.7, 137.4, 135.7, 134.6, 133.8, 133.4, 133.1, 132.4, 129.8, 128.31, 128.25, 128.2, 128.1, 127.84, 127.75, 127.6, 127.2, 127.1, 126.9, 126.6, 125.7, 125.5, 51.9. HRMS (ESI) calcd for $C_{29}H_{20}O_3Na$ $[M+Na]^+$ 439.1310, found 439.1311.

Compound **5aa** was prepared following the **Typical Procedure F**

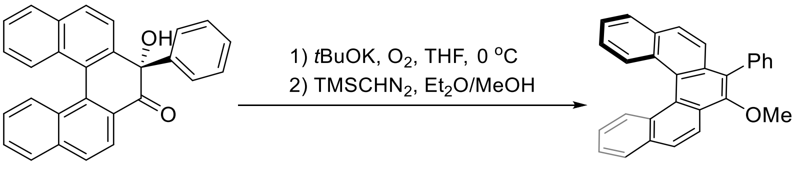

3aa **8aa**

The reaction of **3aa** (77.3 mg, 0.20 mmol, 1.0 equiv) and *t*BuOK (89.8 mg, 0.80 mmol, 4.0 equiv) in THF (4 mL) at 0 °C for 3 h afforded crude acid, which was used in next step without further purification.

The reaction of the above crude product and $TMSCHN_2$ (2.0 M in hexane, 0.50 mL, 1.0 mmol) in anhydrous Et_2O (1 mL) and MeOH (1 mL) at room temperature afforded **4aa** (45.0 mg, 54%) and **5aa** (27.7 mg, 36%). Analytic data of **8aa** : **^{1}H NMR** (500 MHz, $CDCl_3$) δ 8.44 (d, *J* = 8.5 Hz, 1H), 8.42 (d, *J* = 8.5 Hz, 1H), 8.29 (d, *J* = 9.0 Hz, 1H), 8.03 – 7.95 (m, 2H), 7.91 (d, *J* = 8.0 Hz, 1H), 7.79 (d, *J* = 9.0 Hz, 1H), 7.63 (d, *J* = 9.0 Hz, 1H), 7.58 – 7.54 (m, 2H), 7.54 – 7.51 (m, 2H), 7.51 – 7.47 (m, 3H), 7.29 – 7.27 (m, 1H), 7.26 – 7.24 (m, 1H), 3.63 (s, 3H). **^{13}C NMR** (126 MHz, $CDCl_3$) δ 152.2, 136.3, 132.5, 131.8, 131.6, 131.4, 130.9, 130.8, 130.7, 129.4, 129.3, 129.2, 128.4, 128.2, 128.0, 127.82, 127.78, 127.6, 127.5, 127.19, 127.18, 126.4, 126.0, 124.55, 124.50, 124.3, 124.0, 120.3, 61.6. HRMS (ESI) calcd for $C_{29}H_{21}O$ $[M+H]^+$ 385.1592, found 385.1591.

Procedure for the synthesis of dicarboxylate 6a and byproduct 6a'

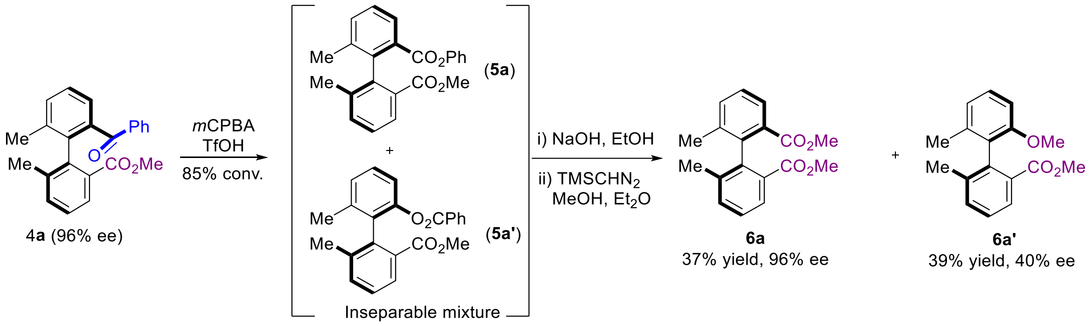

Under nitrogen atmosphere, a solution of **4a** (62.8 mg, 0.20 mmol, 1.0 equiv) in DCE (3 mL) was added *m*CPBA (60% in mineral oil, 32.0 mg, 0.80 mmol, 10.0 equiv) and TfOH (30.2 mg, 0.20 mmol, 1.0 equiv) sequentially at 0 °C, then the mixture was stirred at room temperature for 48 h, The reaction mixture was then poured into $Na_2O_3S_2$ (10 mL), extracted with CH_2Cl_2 for three times. The combined organic layer was washed with brine and dried over anhydrous Na_2SO_4. After filtration the solvent was concentrated in vacuum and purified by flash column chromatography (PE/EtOAc = 10:1) on silica gel to afford crude esters which was used in next step without further purification.

A mixture of above esters and ethanol (2 mL) was added NaOH (80 mg, 2 mmol, 10 equiv), then refluxed at 65 °C for 10 h. The completion of the reaction was monitored by TLC. The reaction mixture was cooled to r.t and 1M HCl (2.5 mL) was added. The mixture was diluted with H_2O and extracted with DCM for three times. The combined organic layer was washed with brine, dried over anhydrous Na_2SO_4, filtered and concentrated to afford crude acid, which was used in next step without further purification.

Under nitrogen atmosphere, to a mixture of the above crude product in anhydrous Et_2O (1 mL) and MeOH (1 mL) was added $TMSCHN_2$ (2.0 M in hexane, 0.50 mL, 1.0 mmol) slowly at room temperature and stirred for 3h. The solvent was removed and the residue was purified by flash chromatography on silica gel (PE/EtOAc = 15:1) to afford **6a** (22.1 mg, 37%, 96% ee) and **6a'** (21.1 mg, 39%, 40% ee).

6a: $[\alpha]_D^{20}$ – 8.70 (c 1.00, $CHCl_3$). HPLC conditions: Chiralcel OD-H, isopropanol/hexane = 2:98, flow: 1.0 mL/min, λ = 254 nm. **^{1}H NMR** (500 MHz, $CDCl_3$) δ 7.86 (dd, *J* = 8.0, 1.4 Hz, 1H), 7.43 (d, *J* = 7.6 Hz, 1H), 7.33 (t, *J* = 7.8 Hz, 1H), 3.58 (s, 3H), 1.91 (s, 3H). **^{13}C NMR** (126 MHz, $CDCl_3$)

δ 167.5, 141.2, 136.5, 133.5, 129.2, 127.7, 126.9, 51.7, 20.0. HRMS (ESI) calcd for $C_{18}H_{18}O_4Na$ $[M+Na]^+$ 321.1097, found 321.1110.

6a': $[\alpha]_D^{20}$ – 3.10 (c 1.00, $CHCl_3$) are also obtained. HPLC conditions: Chiralcel IC, isopropanol/hexane = 2:98, flow: 1.0 mL/min, λ = 254 nm. **^{1}H NMR** (500 MHz, $CDCl_3$) δ 7.81 (dd, *J* = 8.0, 1.5 Hz, 1H), 7.45 (d, *J* = 7.5 Hz, 1H), 7.35 – 7.30 (m, 1H), 7.26 – 7.22 (m, 1H), 6.89 (d, *J* = 7.5 Hz, 1H), 6.81 (d, *J* = 8.0 Hz, 1H), 3.68 (s, 3H), 3.59 (s, 3H), 1.99 (s, 3H), 1.89 (s, 3H). **^{13}C NMR** (126 MHz, $CDCl_3$) δ 167.9, 156.2, 138.3, 137.7, 136.7, 133.4, 130.8, 128.7, 127.8, 127.5, 126.9, 122.2, 107.9, 55.7, 51.7, 19.8, 19.6. HRMS (ESI) calcd for $C_{17}H_{18}O_3Na$ $[M+Na]^+$ 293.1148, found 293.1161.

Procedure for the synthesis of M1

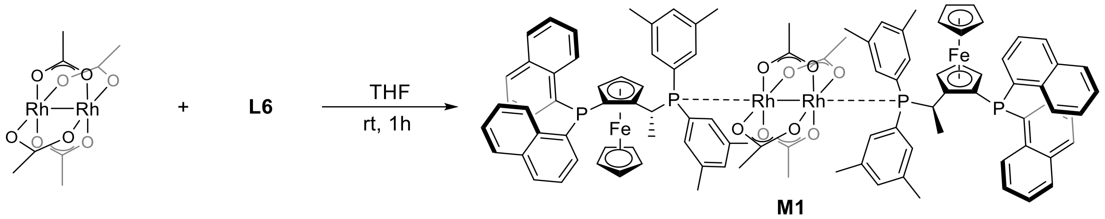

Under nitrogen atmosphere, a mixture of $Rh_2(OAc)_4$ (44.2 mg, 0.1 mmol, 1.0 equiv) and L6 (147.7 mg, 0.2 mmol, 2.0 equiv) in anhydrous THF (1.5 mL) was stirred at room temperature for 1 h. Then the mixture was added MeOH (15 mL) and stirred for 10 min. Then the residue was washed with MeOH and dried under vacuum to afford **M1** (172.7 mg, 90% yield) as an orange solid. **^{1}H NMR** (600 MHz, C_6D_6) δ 10.05 – 9.92 (m, 2H), 8.48 (d, *J* = 8.8 Hz, 2H), 8.03 – 7.93 (m, 4H), 7.71 – 7.65 (m, 4H), 7.63 (d, *J* = 8.2 Hz, 2H), 7.61 – 7.52 (m, 8H), 7.50 (d, *J* = 8.2 Hz, 2H), 7.33 – 7.27 (m, 4H), 7.21 – 7.17 (m, 2H), 7.05 – 7.00 (m, 2H), 6.95 – 6.91 (m, 2H), 6.90 – 6.85 (m, 2H), 6.80 (s, 2H), 6.67 (s, 2H), 5.50 (s, 2H), 5.08 (s, 2H), 4.31 (s, 2H), 4.23 (s, 2H), 3.64 (s, 10H), 2.70 (s, 6H), 2.10 (s, 12H), 1.96 (s, 12H), 1.66 (s, 12H). **^{13}C NMR** (151 MHz, C_6D_6) δ 192.0, 137.6, 137.52, 137.47, 137.1, 136.9, 136.1, 135.5, 134.8, 134.6, 134.2, 134.04(d, *J* = 5.7 Hz), 133.77(d, *J* = 3.8 Hz), 133.6, 132.9, 131.5, 130.9, 130.7, 129.6, 129.3, 128.7, 128.3, 127.1, 126.9, 126.81, 126.77, 126.6, 126.3, 126.0, 125.8, 125.7, 125.6, 101.54(d, *J* = 29.6 Hz), 75.12(d, *J* = 8.8 Hz), 73.6, 71.2, 69.6, 69.2, 32.84(d, *J* = 13.0 Hz), 24.0, 23.6, 21.6, 21.4. **^{31}P NMR** (243 MHz, C_6D_6) δ -10.1, -49.9.

Procedure for the synthesis of M6

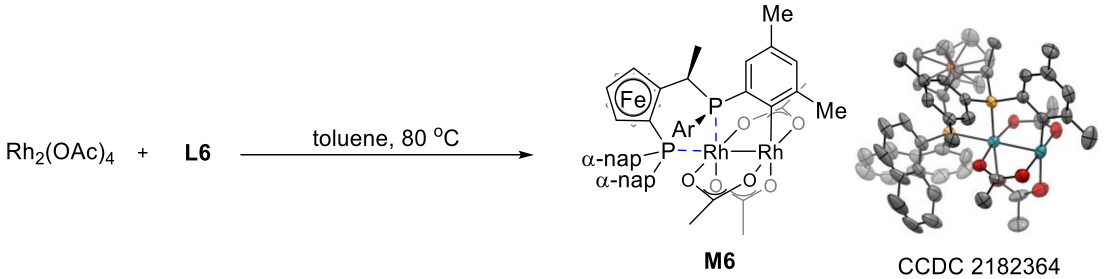

Under nitrogen atmosphere, a mixture of $Rh_2(OAc)_4$ (88.4 mg, 0.2 mmol, 1.0 equiv) and L6 (147.7 mg, 0.2 mmol, 1.0 equiv) in anhydrous toluene (1.5 mL) was stirred at 80 °C for 10 h. The solvent was removed and the residue was purified by flash chromatography on silica gel (PE/EtOAc = 5:1) to afford **M6** (153.2 mg, 68% yield). **^{1}H NMR** (500 MHz, C_6D_6) δ 9.65 (ddd, *J* = 17.5, 7.0,

2.0 Hz, 1H), 8.86 (d, J = 8.5 Hz, 1H), 8.23 (d, J = 9.0 Hz, 1H), 7.82 – 7.71 (m, 2H), 7.53 (d, J = 8.0 Hz, 1H), 7.40 – 7.30 (m, 2H), 6.97 – 6.85 (m, 4H), 6.83 – 6.76 (m, 4H), 6.64 – 6.57 (m, 2H), 6.55 (s, 1H), 5.03 (s, 1H), 4.69 (s, 1H), 4.48 (dq, J = 15.0, 7.0 Hz, 1H), 4.20 – 4.13 (m, 1H), 3.56 (s, 5H), 3.22 (s, 3H), 2.52 (s, 3H), 2.03 (s, 3H), 2.02 – 1.96 (m, 3H), 1.77 (s, 9H), 0.62 (s, 3H). **^{13}C NMR** (126 MHz, C_6D_6) δ 188.6, 180.88 (d, J = 2.9 Hz), 170.02 (t, J = 30.4 Hz), 146.7, 146.62 (d, J = 3.4 Hz), 146.48 (d, J = 3.2 Hz), 146.2, 139.3, 139.1, 136.7, 136.6, 135.5, 135.4, 134.16 (d, J = 3.2 Hz, 1H), 134.03, 133.97, 132.8, 132.7, 132.3, 132.19 (d, J = 2.4 Hz), 132.1, 131.93 (d, J = 7.4 Hz), 131.39 (d, J = 2.8 Hz), 130.9, 130.6, 130.5, 129.70 (d, J = 2.9 Hz), 129.6, 129.4, 129.29 (d, J = 3.2 Hz, 1H), 129.12 (d, J = 4.0 Hz), 129.1, 128.95 (dd, J = 5.8, 3.5 Hz), 128.5, 128.3, 128.1, 127.9, 127.3, 126.4, 126.03 (d, J = 2.8 Hz), 125.8, 125.6, 125.5, 125.3, 125.0, 124.1, 124.09 (d, J = 8.3 Hz), 95.63 (d, J = 23.7 Hz), 74.31 (d, J = 2.9 Hz), 73.06 (d, J = 5.5 Hz), 72.77 (d, J = 5.5 Hz), 71.5, 71.1, 70.45 (dd, J = 8.3, 0.5 Hz), 70.22 (d, J = 3.3 Hz), 30.2, 30.0, 24.7, 23.3, 22.86 (d, J = 7.4 Hz), 21.0, 20.8, 15.77 (d, J = 7.6 Hz). **^{31}P NMR** (202 MHz, C_6D_6) δ 20.1 (ddd, J = 126, 23.7, 4.6 Hz), -62.63 (ddd, J =87.0, 30.4, 23.6 Hz). HRMS (ESI) calcd for $C_{54}H_{53}FeO_6P_2Rh$ $[M+H]^+$ 1121.0771, found 1121.0776.

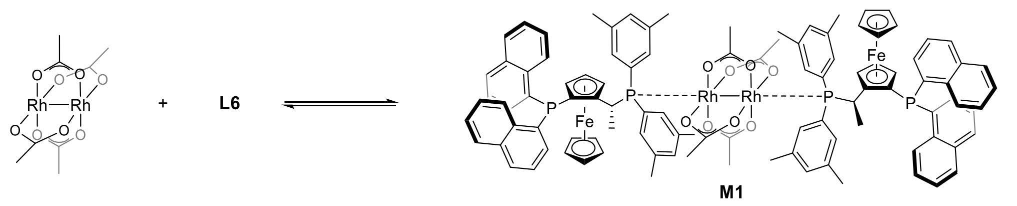

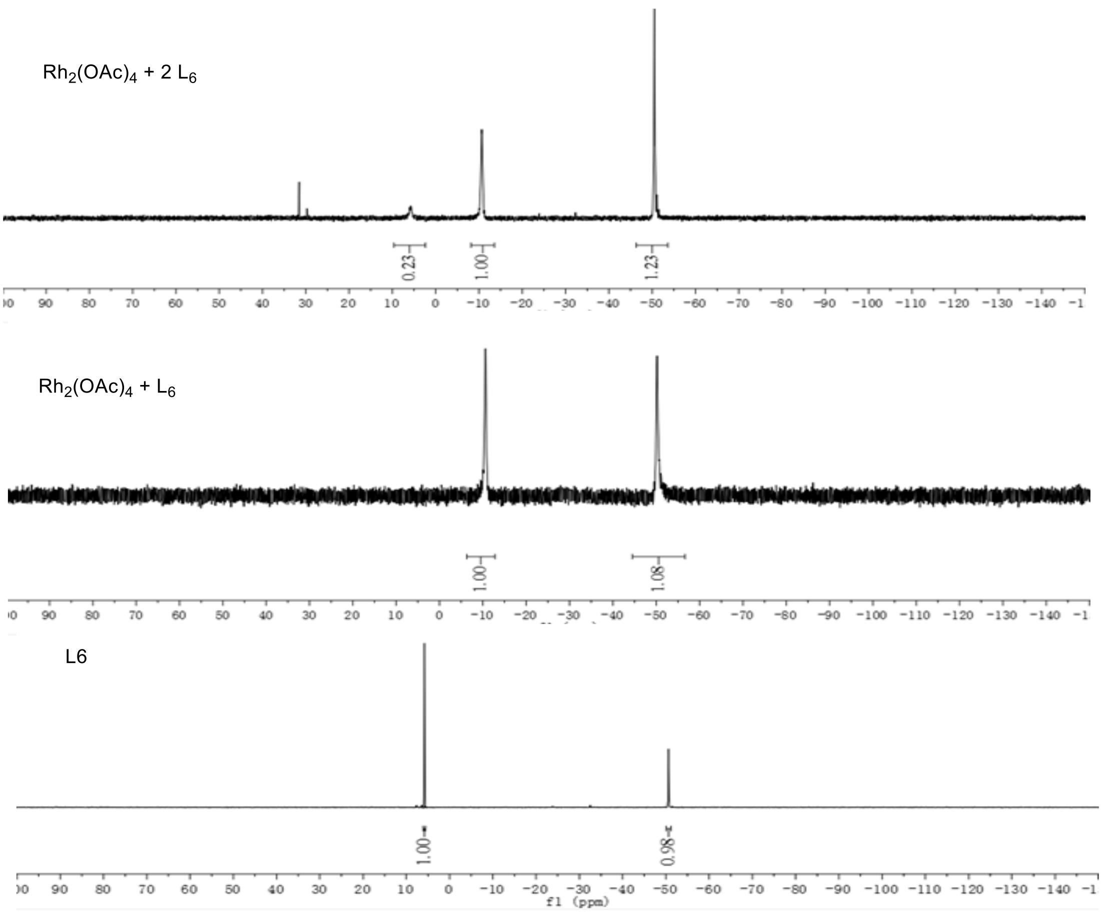

Figure S1. Complexation by ^{31}P NMR Analysis.

Determination of regioselectivity of 3bb and 3cc.

(1) **Determination of regional selectivity of 3bb by X-ray of amide derivative S26**

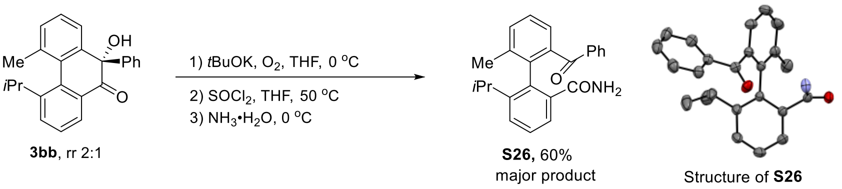

Under oxygen atmosphere, a solution of **3bb** (68.5 mg, 0.20 mmol, 1.0 equiv) in THF (4 mL) was added *t*BuOK (89.8 mg, 0.80 mmol, 4.0 equiv) at 0 °C, then the mixture was stirred at the same temperature for 3 h. The reaction was restored to room temperature, 1M HCl (1 mL) was added and stirred for 10 min. The mixture was diluted with H_2O and extracted with EtOAc for three times. The combined organic layer was washed with brine, dried over anhydrous Na_2SO_4, filtered and concentrated to afford crude acid, which was used in next step without further purification.

Under nitrogen atmosphere, to a mixture of the above crude product in anhydrous THF (1.0 mL) was added thionyl chloride (1.0 mL) at room temperature and stirred for 1 h at 50 °C. The mixture was allowed to cool to room temperature and the solvent was removed. The residue was dissolved in anhydrous THF (1.0 mL) and then cooled to 0 °C and then carefully poured into a cooled ammonium hydroxide solution (37%, 0.5 mL) while stirring. After 30 min, the mixture was extracted with EA for three times. The organic extract was dried and evaporated. purified by flash chromatography on based silica gel with Et_3N (PE/EtOAc = 1:1) to afford **S26** (42.9 mg, 93% yield).**^{1}H NMR** (500 MHz, $CDCl_3$) δ 7.88 (dd, *J* = 8.5, 1.5 Hz, 2H), 7.79 (s, 1H), 7.63 – 7.58 (m, 1H), 7.49 (dd, *J* = 7.5, 1.5 Hz, 1H), 7.48 – 7.46 (m, 1H), 7.46 – 7.43 (m, 2H), 7.40 – 7.33 (m, 2H), 7.32 – 7.27 (m, 2H), 5.24 (s, 1H), 2.54 (hept, *J* = 7.0 Hz, 1H), 2.05 (s, 3H), 0.93 (d, *J* = 7.0 Hz, 3H), 0.59 (d, *J* = 7.0 Hz, 3H). **^{13}C NMR** (126 MHz, $CDCl_3$) δ 198.8, 172.7, 145.4, 139.3, 138.5, 138.1, 137.5, 136.1, 133.8, 133.42, 133.39, 131.0, 128.44, 128.35, 127.3, 126.5, 126.4, 125.3, 30.0, 24.8, 23.2, 20.4. HRMS (ESI) calcd for $C_{24}H_{23}NO_2Na$ $[M+Na]^+$ 380.1621, found 380.1631.

(2) **Determination of regioselectivity of 3cc by ^{1}H NMR analysis**

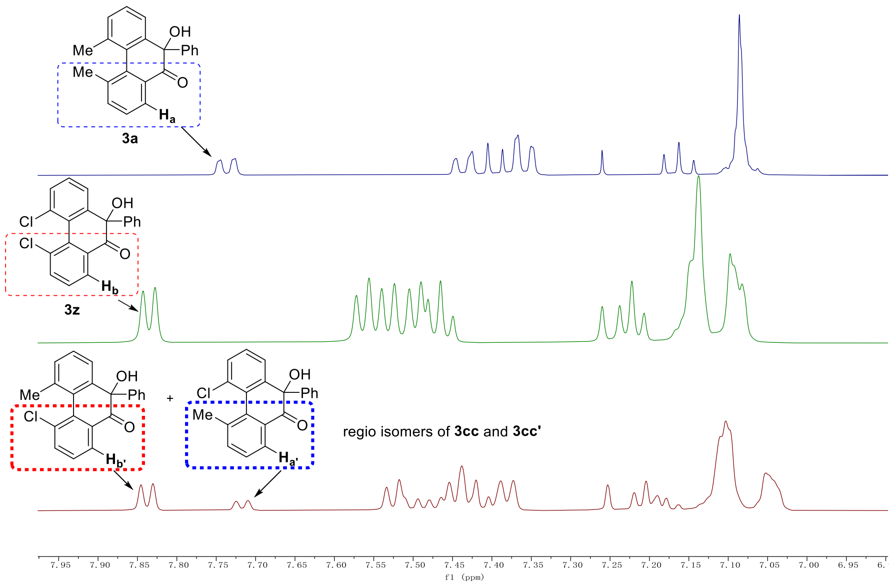

Figure S2. Comparison of ^{1}H NMR of **3a**, **3z** and **3cc**, **3cc'**.

Kinetic Studies

(1) The effect of the concentration of $Rh_2(OAc)_4(L6)_2$.

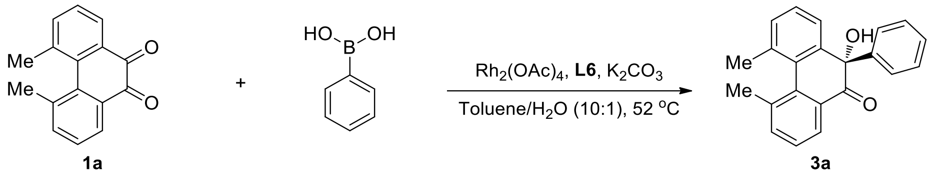

Under nitrogen atmosphere, to a Schlenk tube was sequentially added **1a** (236.1 mg, 1.0 mmol, 1.0 equiv), phenylboronic acid (305.0 mg, 2.5 mmol, 2.5 equiv), $Rh_2(OAc)_4$ (**x** mol%), **L6** (**2x** mol%) and K_2CO_3 (138.2 mg, 1.0 mmol, 1.0 equiv) in toluene (10 mL) and deionized water (1.0 mL) was heated at 52 °C. 0.25 mL of the reaction mixture was taken out every 20 – 40 min and it was passed through a short pad of silica gel. The conversion was calculated by the analysis of crude ^1H NMR of the mixture.

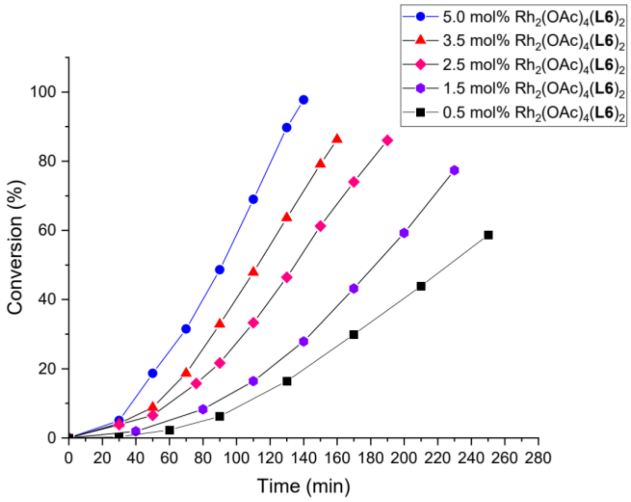

Figure S3. Conversion vs time at different concentrations of $Rh_2(OAc)_4(L6)_2$.

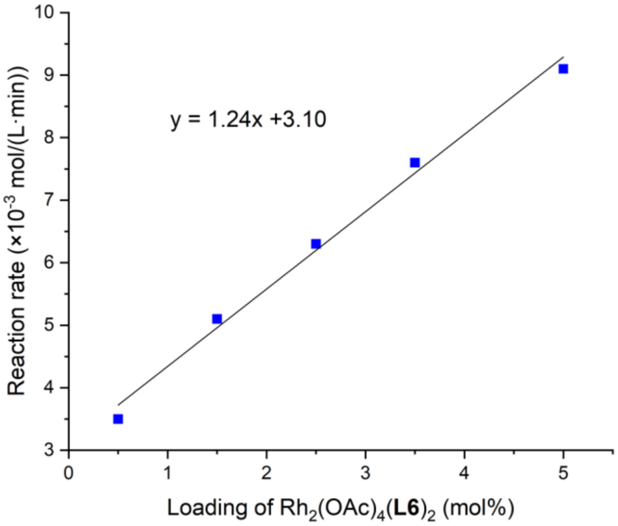

Figure S4. Reaction rate of different concentration of $Rh_2(OAc)_4(L6)_2$.

(2) The effect of the concentration of PhB(OH)₂.

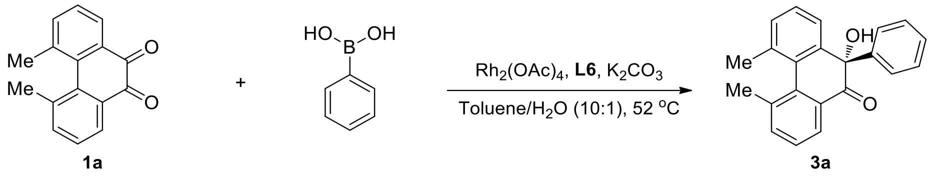

Under nitrogen atmosphere, to a Schlenk tube was sequentially added **1a** (236.1 mg, 1.0 mmol, 1.0 equiv), phenylboronic acid (**x** equiv), $Rh_2(OAc)_4$ (11.0 mg, 2.5 mol%), **L6** (37 mg, 5.0 mol%) and K_2CO_3 (138.2 mg, 1.0 mmol, 1.0 equiv) in toluene (10 mL) and deionized water (1 mL) was heated at 52 °C. 0.25 mL of the reaction mixture was taken out every 20 – 40 min and it was passed through a short pad of silica gel. The conversion was calculated by the analysis of crude ^{1}H NMR of the mixture.

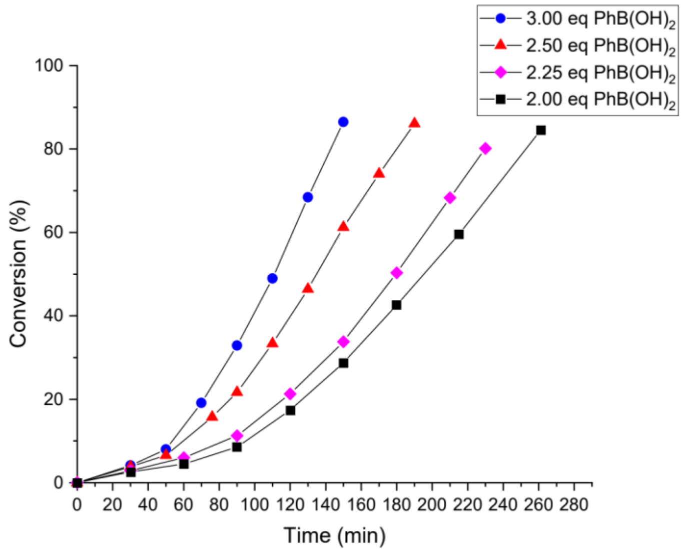

Figure S5. Conversion vs time at different loadings of $PhB(OH)_2$.

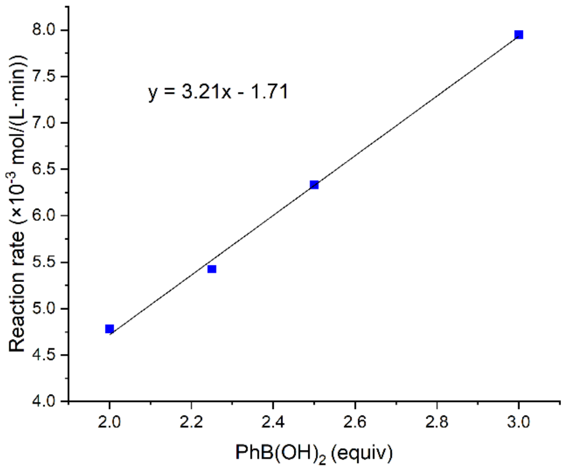

Figure S6. Reaction rate of different concentration of $PhB(OH)_2$.

(3) The effect of the concentration of diketone 1a.

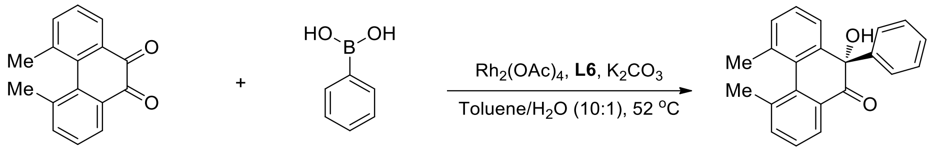

1a **3a**

Under nitrogen atmosphere, to a Schlenk tube was sequentially added **1a** (**x** equiv), phenylboronic acid (305.0 mg, 2.5 mmol, 2.5 equiv), $Rh_2(OAc)_4$ (6.6 mg, 1.5 mol%), **L6** (22.1 mg, 3.0 mol%) and K_2CO_3 (138.2 mg, 1.0 mmol, 1.0 equiv) in toluene (10 mL) and deionized water (1 mL) was heated at 52 °C. 0.25 mL of the reaction mixture was taken out every 20 – 40 min and it was passed through a short pad of silica gel. The conversion was calculated by the analysis of crude ^1H NMR of the mixture.

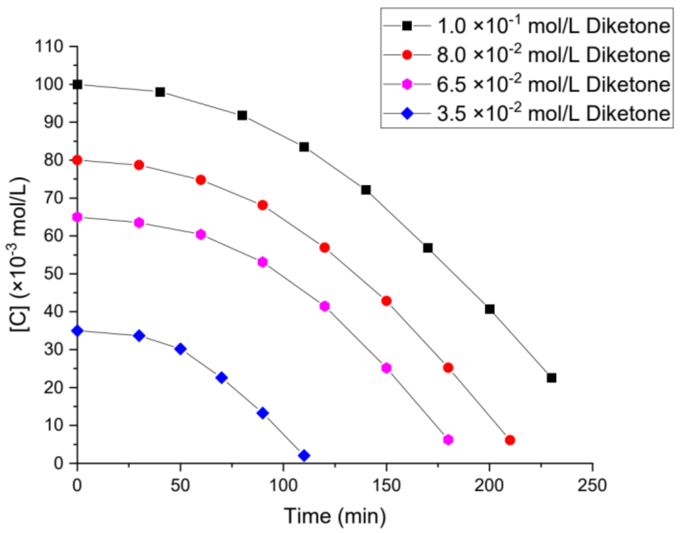

Figure S7. Concentrations of Diketone 1a vs time.

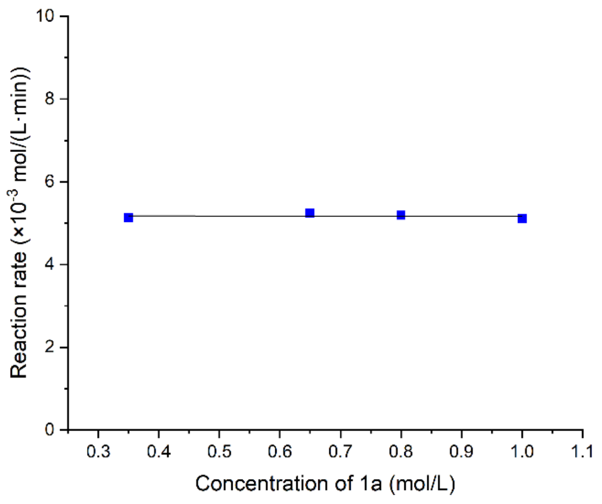

Figure S8. Reaction rate at different concentration of 1a.

(4) Kinetic analysis for $Rh_2(OAc)_4(L6)_2$ and in situ combination of $Rh_2(OAc)_4$ and L6

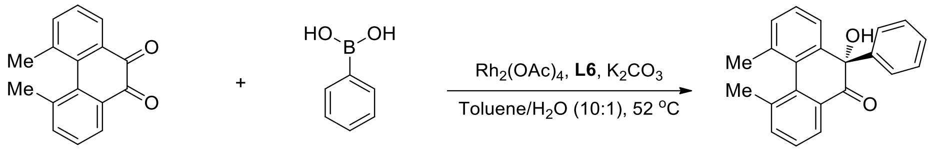

Under nitrogen atmosphere, to a Schlenk tube was sequentially added **1a** (236.1 mg, 1.0 mmol, 1.0 equiv), phenylboronic acid (305.0 mg, 2.5 mmol, 2.5 equiv), $Rh_2(OAc)_4$ (11.0 mg, 2.5 mol%), **L6** (37 mg, 5.0 mol%) and K_2CO_3 (138.2 mg, 1.0 mmol, 1.0 equiv) in toluene (10 mL) and deionized water (1 mL) was heated at 52 °C. 0.25 mL of the reaction mixture was taken out every 20 – 40 min and it was passed through a short pad of silica gel. The conversion was calculated by the analysis of crude ^{1}H NMR of the mixture.

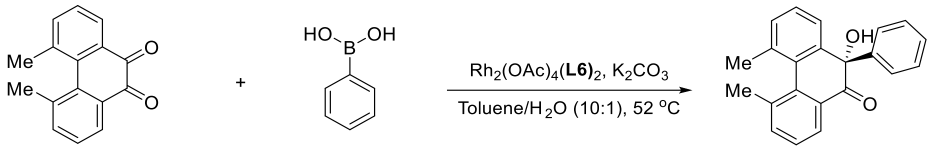

Under nitrogen atmosphere, to a Schleck tube was added $Rh_2(OAc)_4$ (11.0 mg, 2.5 mol%), **L6** (37 mg, 5.0 mol%) and toluene (5 mL) at room temperature and was stirred for 30 minutes. The solution was transfer via cannula carefully to another Schlenk tube charged with **1a** (236.1 mg, 1.0 mmol, 1.0 equiv), phenylboronic acid (305.0 mg, 2.5 mmol, 2.5 equiv), K_2CO_3 (138.2 mg, 1.0 mmol, 1.0 equiv) in toluene (5 mL) and deionized water (1 mL). The tube was stirred at 52 °C. 0.25 mL of the reaction mixture was taken out every 20 – 40 min and it was passed through a short pad of silica gel. The conversion was calculated by the analysis of crude ^{1}H NMR of the mixture.

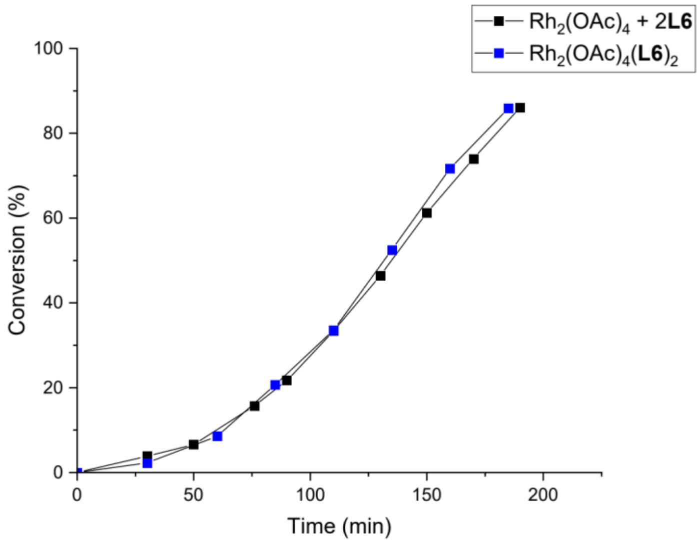

Figure S9. Conversion of 1a vs time.

Non-linear effect

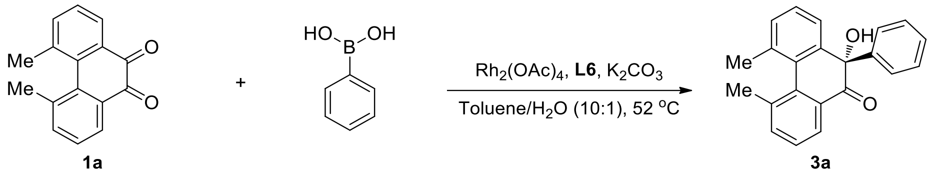

Under nitrogen atmosphere, to a Schlenk tube was sequentially added **1a** (94.5 mg, 0.40 mmol, 1.0 equiv), phenylboronic acid (122.0 mg, 1.0 mmol, 2.5 equiv), $Rh_2(OAc)_4$ (4.4 mg, 2.5 mol%), **L6** (14.8 mg, 5.0 mol%) and K_2CO_3 (55.3 mg, 0.40 mmol, 1.0 equiv) in toluene (3 mL) and deionized water (0.3 mL) was heated at 60 °C for 12 h. After being cooled to room temperature, the solvent was removed and the residue was purified by flash chromatography on silica gel (PE/EtOAc = 10:1) to afford the desired product **3a.** The ee of **3a** was detected by the analysis of HPLC. HPLC conditions: Chiralcel AD-H, isopropanol/hexane = 10:90, flow: 1.0 mL/min, λ = 254 nm.

Table S1 The ee of product 3a of different ee of L6

Entry	1	2	3	4	5	6	7
ee% of **L6**	-100	-50.0	0.0	32.4	50.0	73.0	100
ee% of **3a**	-96	-46	0	29	47	71	96

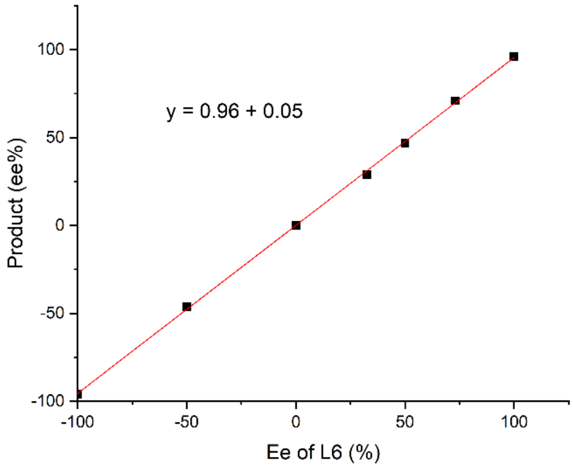

Figure S10. The ee of 3a at different ee of L6

The Procedure for Rh-catalyzed asymmetric addition of arylboronic acids to diketones using M6

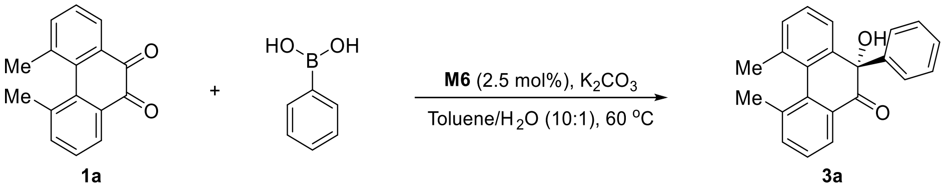

Under nitrogen atmosphere, to a Schlenk tube was sequentially added **1a** (47.2 mg, 0.20 mmol, 1.0 equiv), phenylboronic acid (73.2 mg, 0.60 mmol, 3.0 equiv), **M6** (5.6 mg, 2.5 mol%) and K_2CO_3 (27.6 mg, 0.20 mmol, 1.0 equiv) in toluene (2 mL) and deionized water (0.2 mL) was heated at 60 °C for 12 h. After being cooled to room temperature, the solvent was removed and the residue was purified by flash chromatography on silica gel (PE/EtOAc = 100:10) to afford **3a** (20.1 mg, 32%, 70% ee).

Catalyst recycling experiments

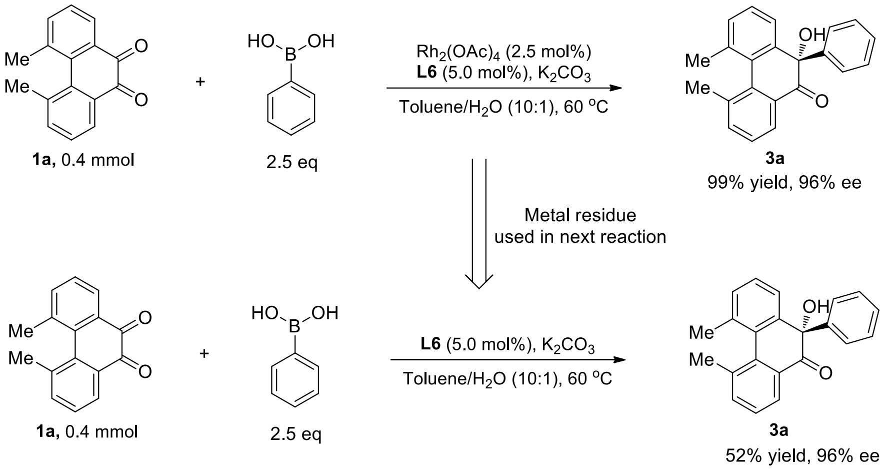

Under nitrogen atmosphere, to a Schlenk tube was sequentially added **1a** (94.5 mg, 0.40 mmol, 1.0 equiv), phenylboronic acid (122.0 mg, 1.0 mmol, 2.5 equiv), $Rh_2(OAc)_4$ (4.4 mg, 2.5 mol%), **L6** (14.8 mg, 5.0 mol%) and K_2CO_3 (55.3 mg, 0.40 mmol, 1.0 equiv) in toluene (3 mL) and deionized water (0.3 mL) was heated at 60 °C for 12 h. After being cooled to room temperature, the solvent was removed and the residue was purified by flash chromatography on silica gel to afford **3a** (124.5 mg, 99%, 96% ee) and the *metal residue,* which was used in the second reaction**.**

Under nitrogen atmosphere, the *metal residue* was added to **1a** (94.5 mg, 0.40 mmol, 1.0 equiv), phenylboronic acid (122.0 mg, 1.0 mmol, 2.5 equiv), **L6** (14.8 mg, 5.0 mol%) and K_2CO_3 (55.3 mg, 0.40 mmol, 1.0 equiv) in toluene (3 mL) and deionized water (0.3 mL). The reaction was stirred at 60 oC for 12 h to afford **3a** (65.4 mg, 52%, 96% ee) via the same workup.

ESI-MS Test

For ESI-MS analysis, the standard reaction mixture solution (1 μL) was taken through a syringe after the initiation period and dissolved in MeCN (1 mL).

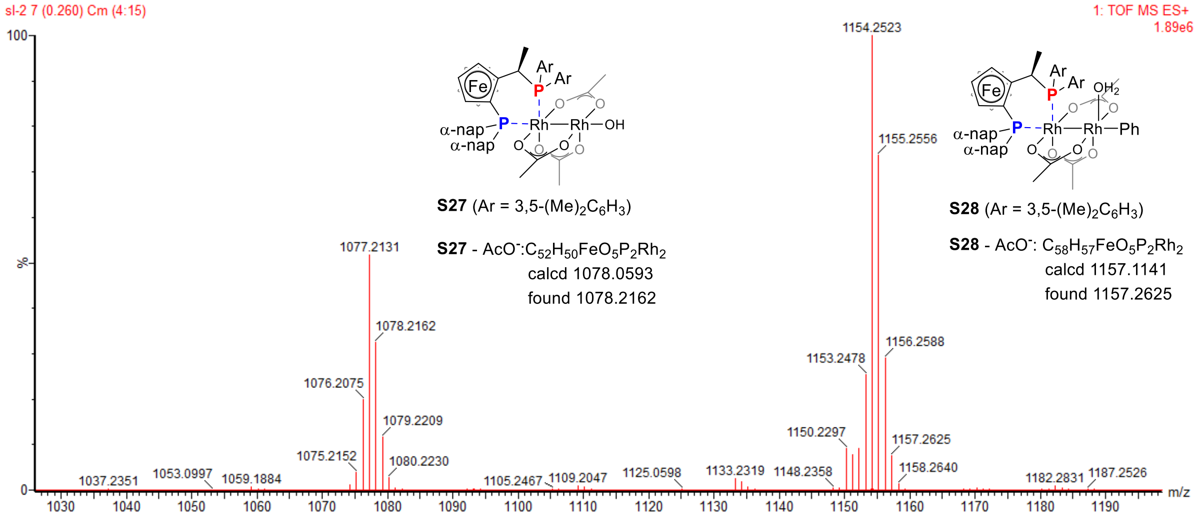

Figure S11. ESI-MS test of reaction mixture solution

Compare ^{1}H and ^{31}P NMR of M1, M6 and the metal residue of the catalytic reaction

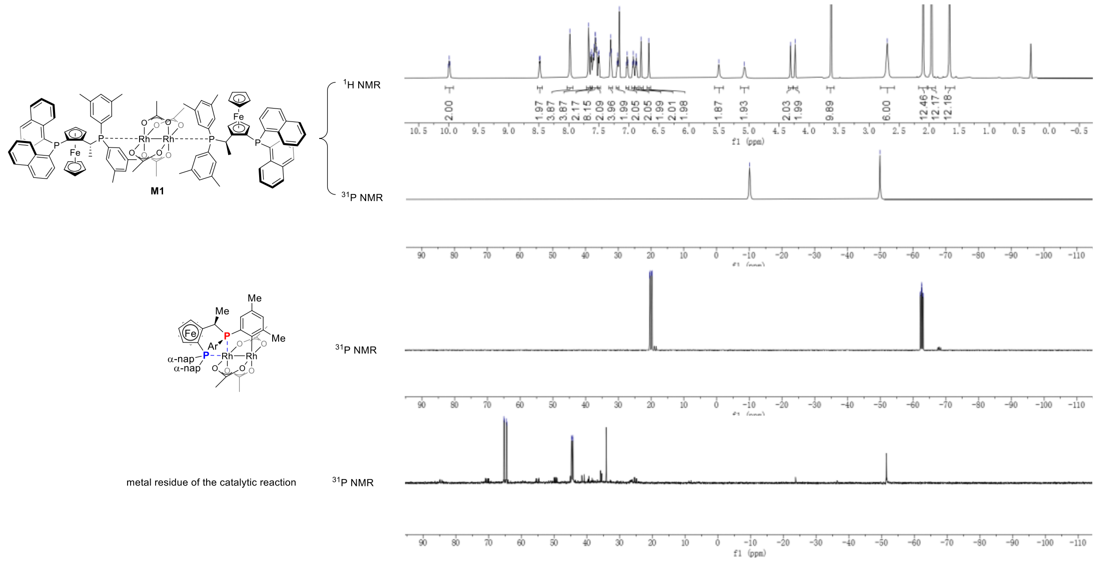

^{31}P NMR of the metal residue of the catalytic reaction with phenylboronic acid and 4-fluorobenzeneboronic acid.

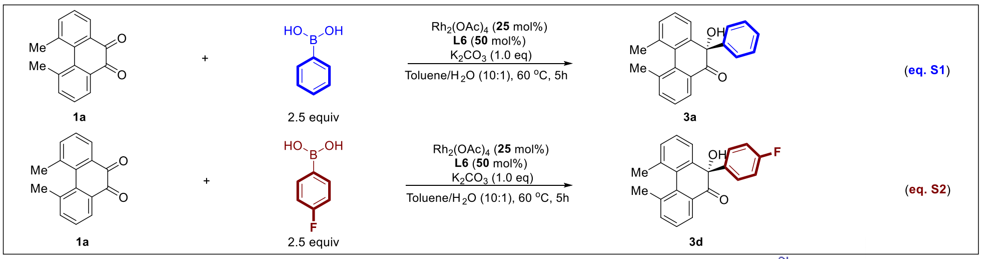

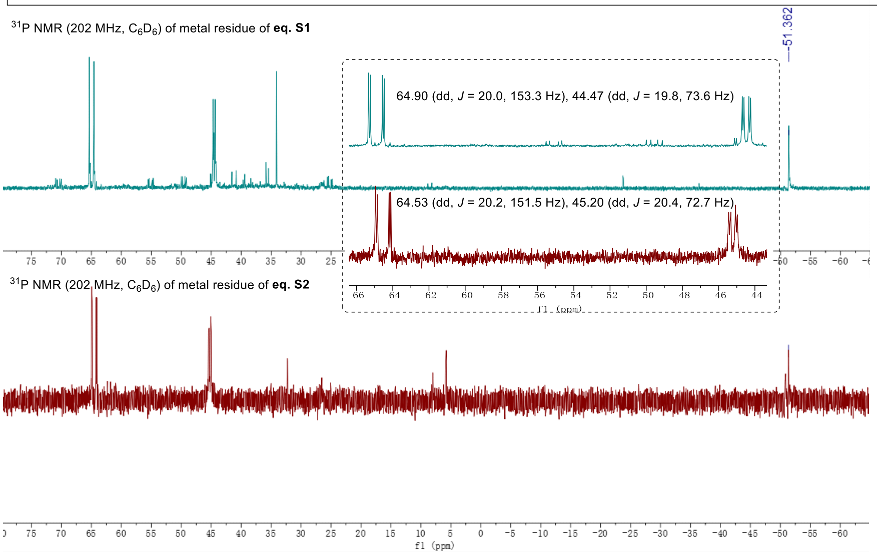

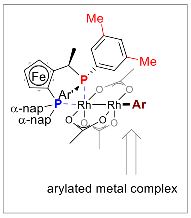

potential reactive intermediate

UV-Vis analysis

For UV-vis analysis, the compounds were dissolved in DCM or DCE to test UV-visible spectroscopy.

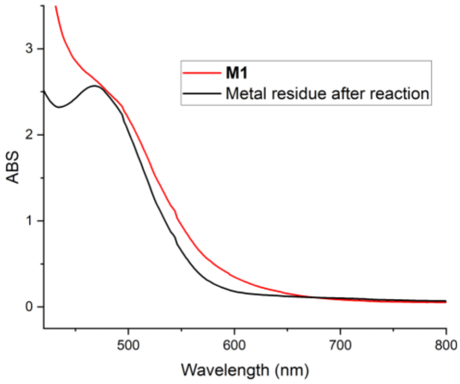

M1:

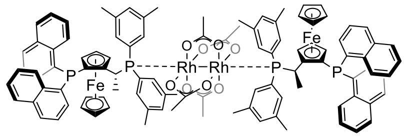

The metal residue of the reaction:
The standard reaction using $Rh_2(OAC)_4$ (25 mol%) and **L6** (50 mol%) was purified by flash chromatography on silica gel to afford the metal residue.

Figure S12. Absorption spectra in $(CH_2Cl)_2$ (2×10^{-3} mol L^{-1})

DFT Calculations

Computational Methods:

B3LYP-D3BJ/6-311G(d,p)

All of the DFT calculations were performed with Gaussian 16 software packages.[12] The optimization calculations and single-point energy calculations were employed at B3LYP level of theory[13] at 298.15 K with the D3 version of Grimme's dispersion (with Becke-Johnson damping).[14] The 6-311G(d,p) basis sets[15] were employed for the C, H, O atoms. Vibrational frequency analysis was calculated at the same level of theory to verify whether each optimized structure is an energy minimum and to evaluate its zero-point vibrational energy. All of the product structures were fully optimized without any symmetric restrictions.

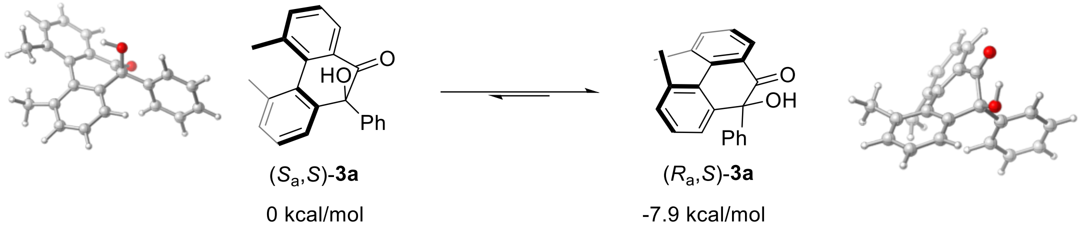

Figure S13. Energy difference between (*S*$_a$, *S*)-3a and (*R*$_a$, *S*)-3a

Table S2. Cartesian coordinates for optimized geometries

（*S*$_a$, *S*） -3a ：

C	3.81415500	-1.76648000	0.30743600
C	3.17202800	-2.83244300	-0.30833800
C	1.83984000	-2.69768000	-0.68513800
C	1.19997900	-1.47902400	-0.50132600
C	1.88934000	-0.34478700	-0.01656300
C	3.19389800	-0.52089000	0.48403900
H	4.82297800	-1.89775000	0.68502000
H	3.68730300	-3.77638900	-0.44002500
H	1.27020900	-3.52806000	-1.08337700
C	1.15357600	0.94989300	-0.04207700
C	1.75201600	2.16821900	-0.42366300
C	-0.23672100	0.92737700	0.22905500
C	1.00116900	3.34616700	-0.32588400
C	-0.95216800	2.11406300	0.32308000
C	-0.32199200	3.33215100	0.08413500
H	1.46831500	4.28431800	-0.60670900
H	-2.00769600	2.08169300	0.55511500
H	-0.87964800	4.25775800	0.16469200
C	-0.28113700	-1.40898700	-0.65438800
C	-0.92850300	-0.42722500	0.37459300
O	-0.90820200	-2.12964300	-1.39230600
O	-0.66056700	-1.00696200	1.66600500
H	0.26773900	-0.85253100	1.86950900

C	-2.42924500	-0.34995100	0.20148600
C	-2.97306600	-0.03555200	-1.04725100
C	-3.28320700	-0.55571900	1.28266800
C	-4.34846200	0.07628500	-1.20816500
H	-2.31968100	0.12143800	-1.89573600
C	-4.66339900	-0.44402900	1.12019400
H	-2.85956500	-0.80278200	2.24550500
C	-5.20009400	-0.12727200	-0.12304300
H	-4.75720700	0.31758500	-2.18260200
H	-5.31726800	-0.60676200	1.96942000
H	-6.27328600	-0.04192700	-0.24945900
C	3.92347000	0.52137800	1.29769600
H	4.77466800	0.94414500	0.75846300
H	3.26949300	1.34025100	1.59366500
H	4.31619100	0.05714500	2.20590000
C	3.12266700	2.26699800	-1.05180700
H	3.85477800	2.71911200	-0.37781200
H	3.50369000	1.29611900	-1.36447100
H	3.06427700	2.90452200	-1.93713100

（*R*$_a$, *S*）-3a ：

C	-0.82610200	3.34187400	-0.01442800
C	-0.04613300	3.47200500	1.12662200
C	0.38681700	2.32632700	1.78559000
C	-0.02883600	1.08239600	1.32755200
C	-0.94819700	0.94746400	0.26114200
C	-1.27251200	2.09602000	-0.48021100
H	-1.08660400	4.22780200	-0.58445000
H	0.27070600	4.45200000	1.46234500
H	1.07919100	2.37259200	2.61689300
C	-1.46671000	-0.42203500	-0.00641200
C	-2.81624800	-0.70176900	-0.30595700
C	-0.57605300	-1.50247400	0.17698200
C	-3.18244600	-2.02588100	-0.57749800
C	-0.96613500	-2.80677800	-0.08346000
C	-2.26872500	-3.06584400	-0.50073100
H	-4.21866900	-2.23781100	-0.81934600
H	-0.25422200	-3.60474200	0.07526500
H	-2.58035500	-4.08178700	-0.71360800
C	0.65095000	-0.13213900	1.81501000
C	0.80997600	-1.23611400	0.74522000
O	1.15202200	-0.27565600	2.91255200
O	1.34235300	-2.38613700	1.36492700
H	1.48989400	-2.13118300	2.29068300

C	1.81077200	-0.70582800	-0.29557100
C	1.44379900	-0.37366300	-1.59803200
C	3.14263700	-0.54921800	0.09671000
C	2.39295700	0.11079000	-2.49575800
H	0.41880100	-0.50301000	-1.91931300
C	4.08897000	-0.06115900	-0.79648900
H	3.43314100	-0.82118700	1.10375100
C	3.71617100	0.27263800	-2.09763100
H	2.09562000	0.35719200	-3.50866600
H	5.11857000	0.05601700	-0.47854000
H	4.45368600	0.65119000	-2.79582600
C	-1.97253500	2.04956900	-1.81661200
H	-2.05802900	1.03244300	-2.19558500
H	-2.97478400	2.48322300	-1.77535900
H	-1.39648100	2.63141000	-2.54065500
C	-3.92031800	0.32695500	-0.23387900
H	-4.77820200	-0.10218400	0.28863200
H	-3.61336700	1.22168800	0.30593000
H	-4.26648700	0.63188200	-1.22494200

Table S3. Thermal correction of Gibbs free energy (TCG, hartree) , single-point energies (E, hartree) and E + Thermal Free Energy Correction (G, hartree)

Compounds	TCG	E	G
(S_a, S) **-3a**	0.290818	-999.976589	-999.685771
(R_a, S) **-3a**	0.29145	-999.989849	-999.698399

Free radical capture experiment

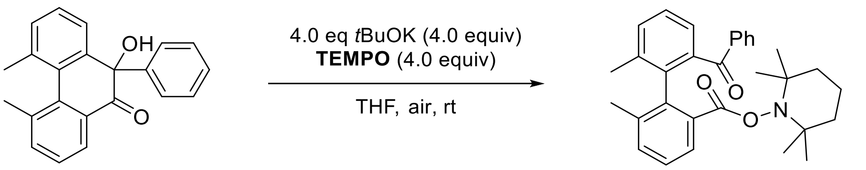

3a **9aa**

Under air atmosphere, a solution of **3a** (31.4 mg, 0.10 mmol, 1.0 equiv) in THF (2 mL) was added *t*BuOK (44.9 mg, 0.40 mmol, 4.0 equiv) and **TEMPO** (62.5 mg, 0.40 mmol, 4.0 equiv) at room temperature, then the mixture was stirred at the same temperature for 30 min. 1 mL of the reaction mixture was taken out and it was passed through a short pad of silica gel. **9aa** was detected by the analysis of HRMS. HRMS (ESI) calcd for $C_{31}H_{36}NO_3$ $[M+H]^+$ 470.2695, found 470.2702. HRMS (ESI) calcd for $C_{31}H_{35}NO_3Na$ $[M+Na]^+$ 492.2515, found 492.2516.

Single Crystal Data

Table S4 Crystal data and structure refinement for 3aa.

Identification code	**3aa**
Empirical formula	$C_{28}H_{18}O_2$
Formula weight	386.42
Temperature/K	150.00(10)
Crystal system	orthorhombic
Space group	$P2_12_12_1$
a/Å	9.0016(3)
b/Å	10.0384(3)
c/Å	20.9374(7)
α/°	90
β/°	90
γ/°	90
Volume/Å^3	1891.94(11)
Z	4
ρ_{calc}g/cm^3	1.357
μ/mm^{-1}	0.663
F(000)	808.0
Crystal size/mm^3	0.13 × 0.11 × 0.09
Radiation	Cu Kα (λ = 1.54184)
2Θ range for data collection/°	8.446 to 147.722
Index ranges	$-10 \le h \le 9$, $-8 \le k \le 12$, $-25 \le l \le 25$
Reflections collected	4662
Independent reflections	3205 [R_{int} = 0.0262, R_{sigma} = 0.0411]
Data/restraints/parameters	3205/0/272
Goodness-of-fit on F^2	1.037
Final R indexes [I>=2σ (I)]	R_1 = 0.0421, wR_2 = 0.1067
Final R indexes [all data]	R_1 = 0.0436, wR_2 = 0.1091
Largest diff. peak/hole / e Å^{-3}	0.18/-0.20
Flack parameter	-0.1(2)

Table S5 Crystal data and structure refinement for 4b.

Identification code	**4b**
Empirical formula	C24H22O3
Formula weight	358.41
Temperature/K	149.99(10)
Crystal system	monoclinic
Space group	P21

a/Å	14.7701(3)
b/Å	7.8414(2)
c/Å	16.9314(3)
α/°	90
β/°	96.701(2)
γ/°	90
Volume/Å3	1947.57(7)
Z	4
ρcalcg/cm3	1.222
μ/mm 1	0.633
F(000)	760.0
Crystal size/mm3	0.12 × 0.11 × 0.1
Radiation	Cu Kα (λ = 1.54184)
2Θ range for data collection/°	5.256 to 148.092
Index ranges	-16 ≤ h ≤ 18, -9 ≤ k ≤ 9, -21 ≤ l ≤ 20
Reflections collected	18150
Independent reflections	7435 [Rint = 0.0282, Rsigma = 0.0316]
Data/restraints/parameters	7435/1/495
Goodness-of-fit on F2	1.095
Final R indexes [I>=2σ (I)]	R1 = 0.0451, wR2 = 0.1165
Final R indexes [all data]	R1 = 0.0462, wR2 = 0.1176
Largest diff. peak/hole / e $Å^{-3}$	0.34/-0.26
Flack/Hooft parameter	0.04(8)/0.06(7)

Table S6 Crystal data and structure refinement for 8aa.

Identification code	**8aa**
Empirical formula	$C_{29}H_{20}O$
Formula weight	384.45
Temperature/K	149.98(10)
Crystal system	monoclinic
Space group	$P2_1/n$
a/Å	9.1753(5)
b/Å	11.3531(8)
c/Å	18.8990(12)
α/°	90
β/°	92.501(5)
γ/°	90
Volume/$Å^3$	1966.8(2)
Z	4

ρ_{calc}g/cm^3	1.298
μ/mm^{-1}	0.077
F(000)	808.0
Crystal size/mm^3	0.13 × 0.11 × 0.09
Radiation	Mo Kα (λ = 0.71073)
2Θ range for data collection/°	4.186 to 50
Index ranges	-10 ≤ h ≤ 10, -9 ≤ k ≤ 13, -19 ≤ l ≤ 22
Reflections collected	9189
Independent reflections	3463 [R_{int} = 0.0303, R_{sigma} = 0.0401]
Data/restraints/parameters	3463/0/272
Goodness-of-fit on F^2	1.059
Final R indexes [I>=2σ (I)]	R_1 = 0.0498, wR_2 = 0.1123
Final R indexes [all data]	R_1 = 0.0651, wR_2 = 0.1214
Largest diff. peak/hole / e Å^{-3}	0.26/-0.18

Table S7 Crystal data and structure refinement for M6.

Identification code	**M6**
Empirical formula	$C_{54.33}H_{52.67}Cl_{0.67}FeO_6P_2Rh_2$
Formula weight	1148.87
Temperature/K	199.98(10)
Crystal system	monoclinic
Space group	$P2_1$
a/Å	16.1289(2)
b/Å	16.2210(3)
c/Å	29.2550(5)
α/°	90
β/°	94.3756(15)
γ/°	90
Volume/Å^3	7631.6(2)
Z	6
ρ_{calc}g/cm^3	1.500
μ/mm^{-1}	8.748
F(000)	3504.0
Crystal size/mm^3	0.14 × 0.12 × 0.11
Radiation	Cu Kα (λ = 1.54184)
2Θ range for data collection/°	5.496 to 143.414
Index ranges	-19 ≤ h ≤ 19, -19 ≤ k ≤ 19, -34 ≤ l ≤ 35
Reflections collected	50787
Independent reflections	23292 [R_{int} = 0.0670, R_{sigma} = 0.0874]

Data/restraints/parameters	23292/66/1807
Goodness-of-fit on F^2	1.024
Final R indexes [I>=2σ (I)]	R_1 = 0.0586, wR_2 = 0.1387
Final R indexes [all data]	R_1 = 0.0711, wR_2 = 0.1466
Largest diff. peak/hole / e $Å^{-3}$	1.65/-0.74
Flack parameter	-0.009(5)

Table S8 Crystal data and structure refinement for S26.

Identification code	**S26**
Empirical formula	$C_{24}H_{23}NO_2$
Formula weight	357.43
Temperature/K	150.00(10)
Crystal system	monoclinic
Space group	$P2_1$
a/Å	12.2340(2)
b/Å	13.7077(2)
c/Å	12.2567(2)
α/°	90
β/°	95.0441(16)
γ/°	90
Volume/$Å^3$	2047.48(6)
Z	4
ρ_{calc}g/cm^3	1.160
μ/mm^{-1}	0.577
F(000)	760.0
Crystal size/mm^3	0.13 × 0.11 × 0.09
Radiation	Cu Kα (λ = 1.54184)
2Θ range for data collection/°	7.24 to 147.246
Index ranges	$-14 \leq h \leq 14$, $-16 \leq k \leq 12$, $-15 \leq l \leq 15$
Reflections collected	13164
Independent reflections	6246 [R_{int} = 0.0174, R_{sigma} = 0.0203]
Data/restraints/parameters	6246/1/493
Goodness-of-fit on F^2	1.074
Final R indexes [I>=2σ (I)]	R_1 = 0.0331, wR_2 = 0.0828
Final R indexes [all data]	R_1 = 0.0335, wR_2 = 0.0833
Largest diff. peak/hole / e $Å^{-3}$	0.13/-0.18
Flack/Hooft parameter	-0.03(8)/-0.04(7)

References:

1. Kun Zhao, Longhui Duan, Shibo Xu, Julong Jiang, Yao Fu, and Zhenhua Gu. *Chem*. **2018**, 4, 599–612.
2. Jian-Feng Wen, Wei Hong, Ke Yuan, Thomas C. W. Mak, and Henry N. C. Wong. *J. Org. Chem.* **2003**, 68, 8918–8931.
3. Rainer Fritsch, Erwin Hartmann, Doris Andert, and Albrecht Mannschreck. *Chemische Berichte*. **1992**, 125, 849 – 856.
4. Cheng-Shein Shu, Ming-Kuo Yeh, Fa-Ching Chen, and Tong-Ing Ho. *Synthesis.* **1987**, 09, 795–797.
5. Haas, Diana; Hammann, Jeffrey M.; Moyeux, Alban; Cahiez, Gérard; Knochel, and Paul. *Synlett.* **2015**, 26, 1515–1519.
6. Bin Wu, and Naohiko Yoshikai. *Angew. Chem. Int. Ed*. **2015**, *54*, 8736–8739.
7. Xiaoping Xue, and Zhenhua Gu. *Org. Lett.* **2019**, 21, 3942–3945.
8. Kai Zhu, Zongqiang Song, Yi Wang, and Fengzhi Zhang. *Org. Lett.* **2020**, 22, 9356-9359.
9. Jie Ke, Bing Zu, Yonghong Guo, Yingzi Li, and Chuan He. *Org. Lett.* **2021**, 23, 329–333.
10. Gaston R. Schaller, Filip Topić, Yoshio Okamoto, Jun Shen, Kari Rissanen, and Rainer Herges. *Nature Chem*. **2014**, 6, 608–613.
11. Oliver Niemeier, Dieter Enders. *Synlett*, **2004**, 12, 2111–2114.
12. M. J. Frisch, G. W. Trucks, H. B. Schlegel, G. E. Scuseria, M. A. Robb, J. R. Cheeseman, G. Scalmani, V. Barone, G. A. Petersson, H. Nakatsuji, X. Li, M. Caricato, A. V. Marenich, J. Bloino, B. G. Janesko, R. Gomperts, B. Mennucci, H. P. Hratchian, J. V. Ortiz, A. F. Izmaylov, J. L. Sonnenberg, D. Williams-Young, F. Ding, F. Lipparini, F. Egidi, J. Goings, B. Peng, A. Petrone, T. Henderson, D. Ranasinghe, V. G. Zakrzewski, J. Gao, N. Rega, G. Zheng, W. Liang, M. Hada, M. Ehara, K. Toyota, R. Fukuda, J. Hasegawa, M. Ishida, T. Nakajima, Y. Honda, O. Kitao, H. Nakai, T. Vreven, K. Throssell, J. A. Montgomery, Jr., J. E. Peralta, F. Ogliaro, M. J. Bearpark, J. J. Heyd, E. N. Brothers, K. N. Kudin, V. N. Staroverov, T. A. Keith, R. Kobayashi, J. Normand, K. Raghavachari, A. P. Rendell, J. C. Burant, S. S. Iyengar, J. Tomasi, M. Cossi, J. M. Millam, M. Klene, C. Adamo, R. Cammi, J. W. Ochterski, R. L. Martin, K. Morokuma, O. Farkas, J. B. Foresman, and D. J. Fox. *Gaussian 16, RevisionC.03*, Gaussian, Inc., Wallingford CT, **2016**.
13. (a) B. Miehlich, A. Savin, H. Stoll, and H. Preuss. *Chem. Phys. Lett.* **1989**, 157, 200–206. (b) C. Lee, W. Yang, and R. G. Parr. Phys. Rev.**1988**, 37, 785–789.
14. (a) S. Grimme, J. Antony, S. Ehrlich, and H. Krieg. *J. Chem. Phys.* **2010**, 132, 154104. (b) S. Grimme, S. Ehrlich, and L. Goerigk. *J. Comp. Chem.* **2011**, 32, 1456–1465.
15. (a) J. A. Pople, R. Krishnan, H. B. Schlegel, and J. S. Binkley. *Int. J. Quantum Chem.* **1978**, 14, 545–560. (b) R. Krishnan, J. S. Binkley, R. Seeger, and J. A. Pople. *J. Chem. Phys.* **1980**, 72, 650.

Copies of NMR Spectroscopies

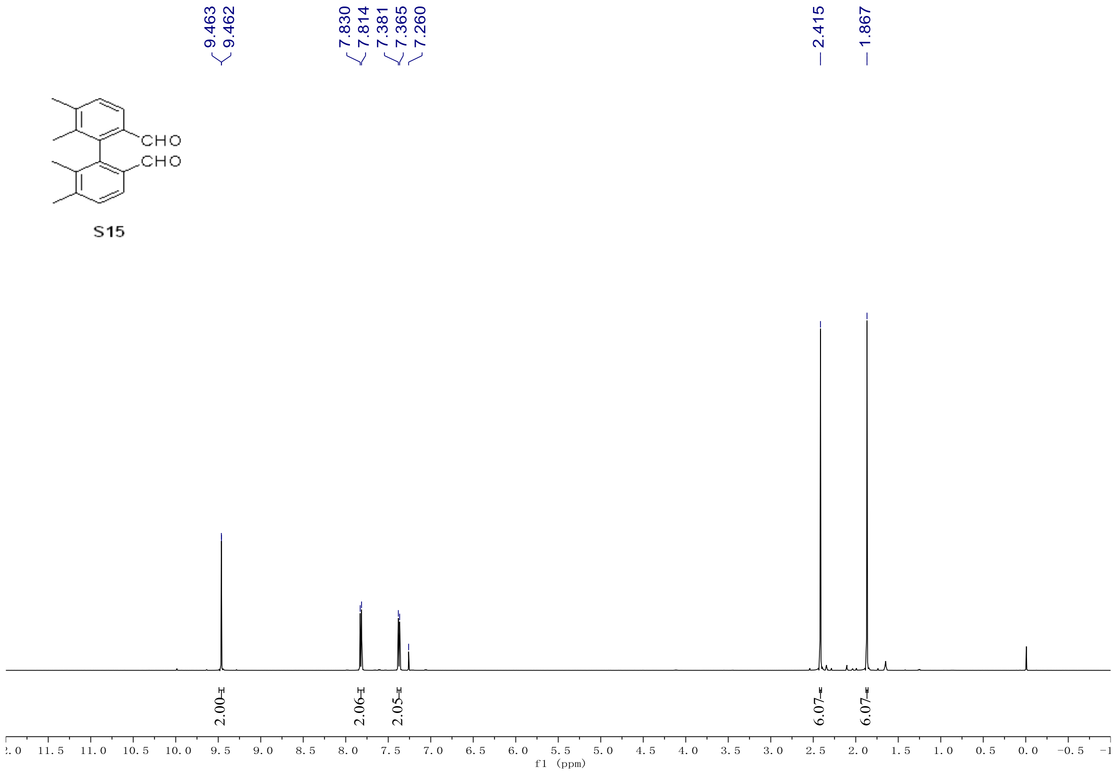

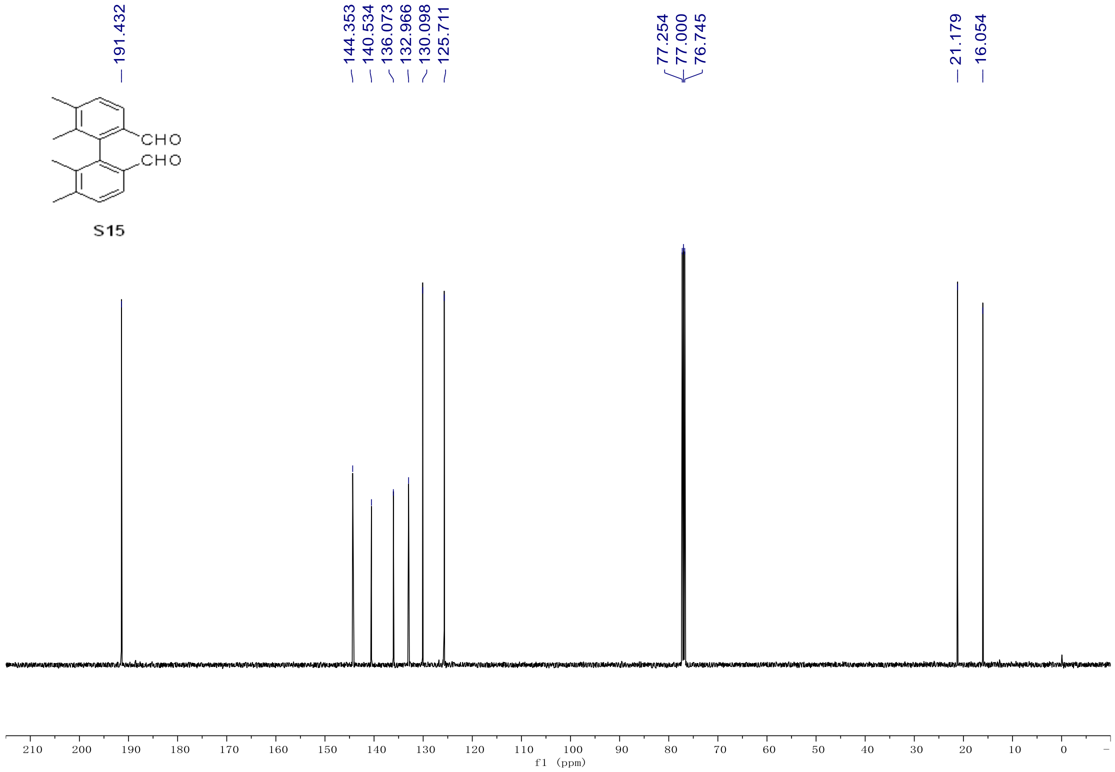

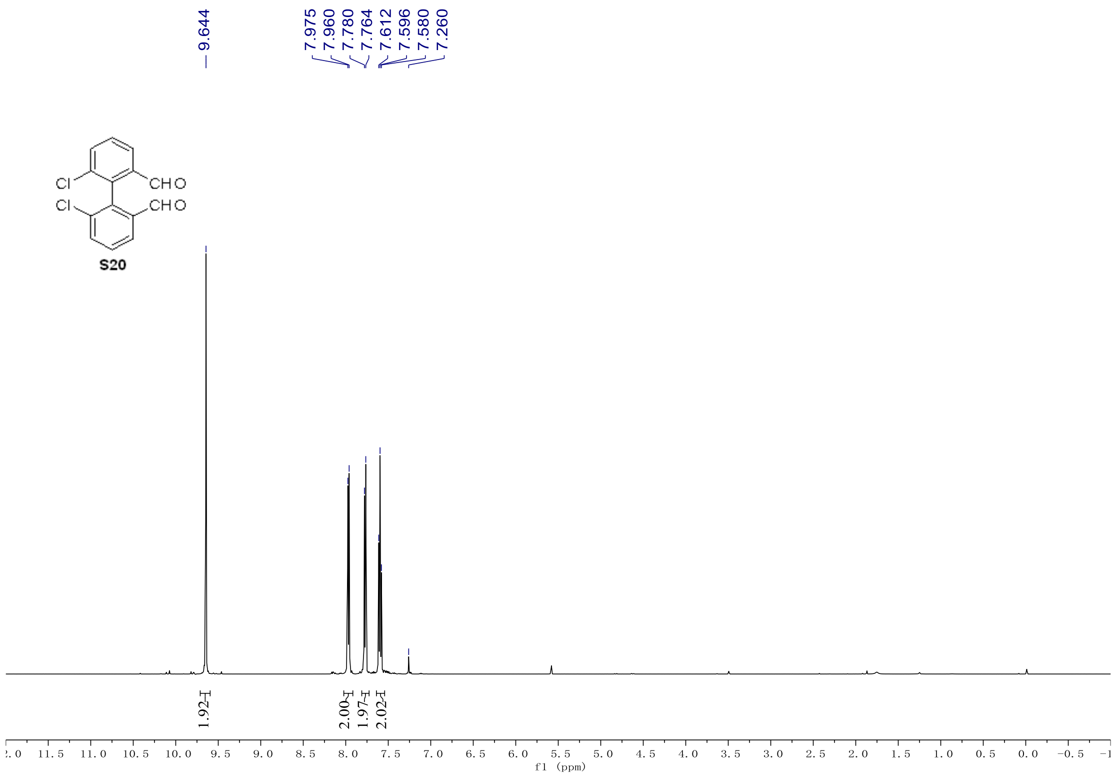
9.644
7.975
7.960
7.780
7.764
7.612
7.596
7.580
7.260
Cl
CHO
Cl
CHO
S20
1.92
2.00
1.97
2.02
f1 (ppm)

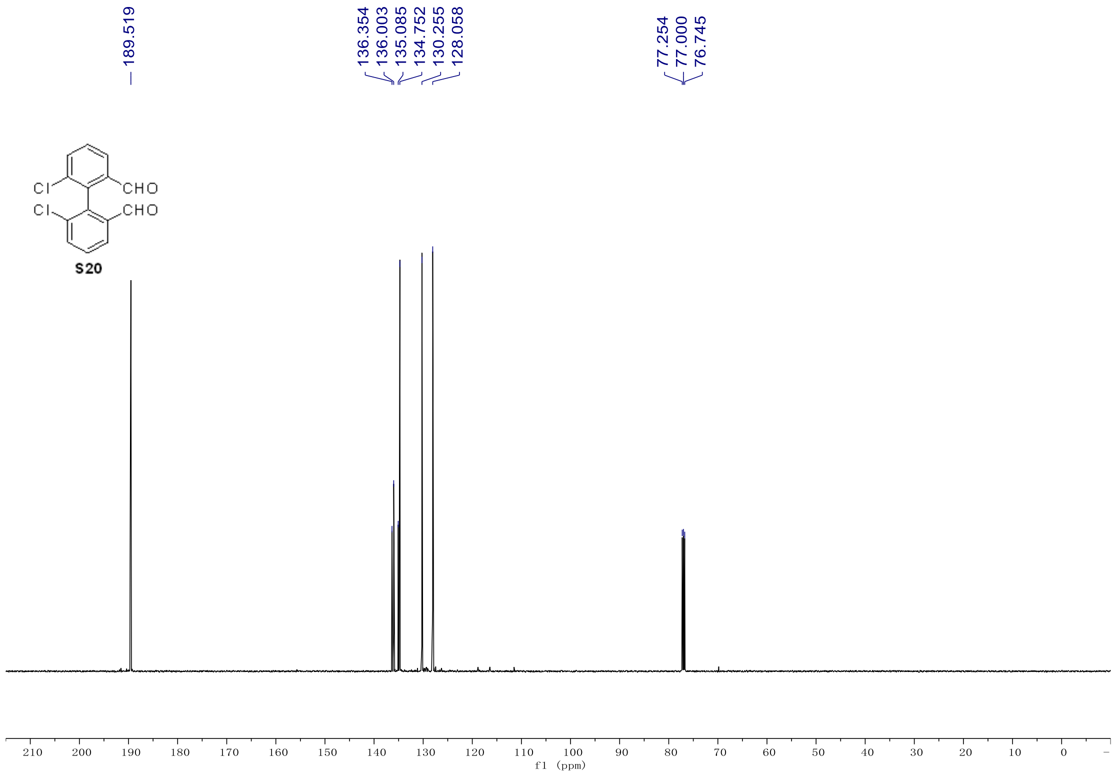
189.519
136.354
136.003
135.085
134.752
130.255
128.058
77.254
77.000
76.745
Cl
CHO
Cl
CHO
S20
f1 (ppm)

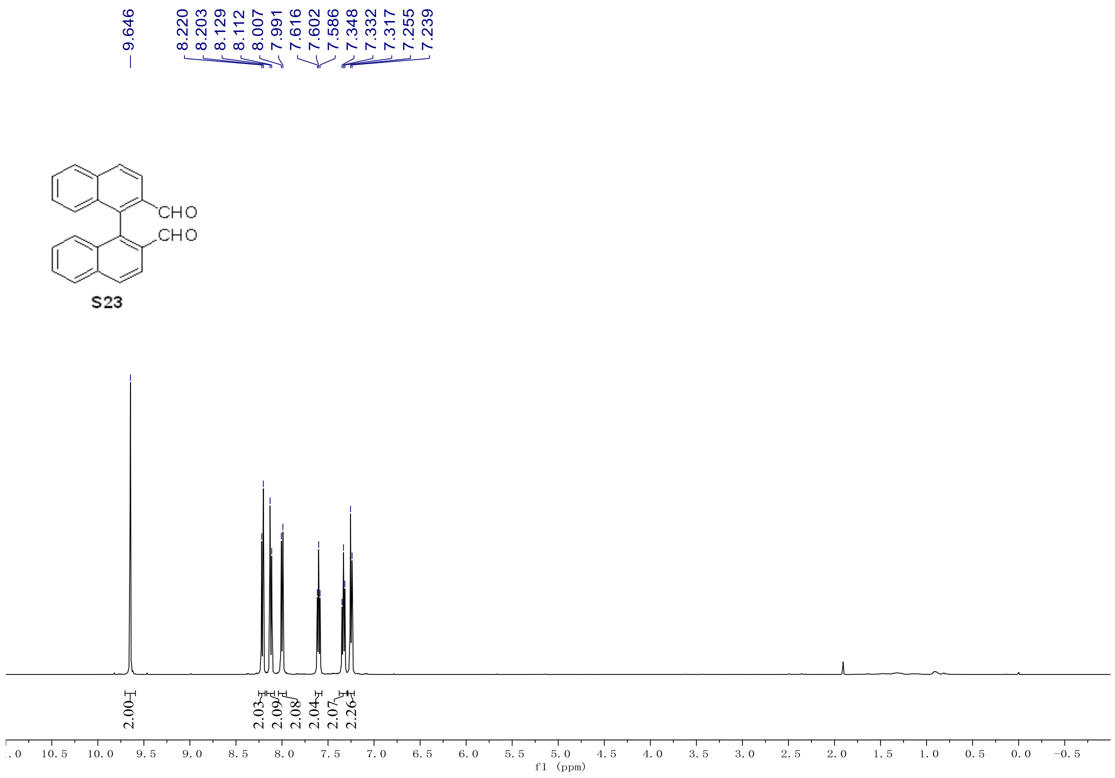
9.646
8.220
8.203
8.129
8.112
8.007
7.991
7.616
7.602
7.586
7.348
7.332
7.317
7.255
7.239
CHO
CHO
S23
2.00
2.03
2.09
2.08
2.04
2.07
2.26
f1 (ppm)

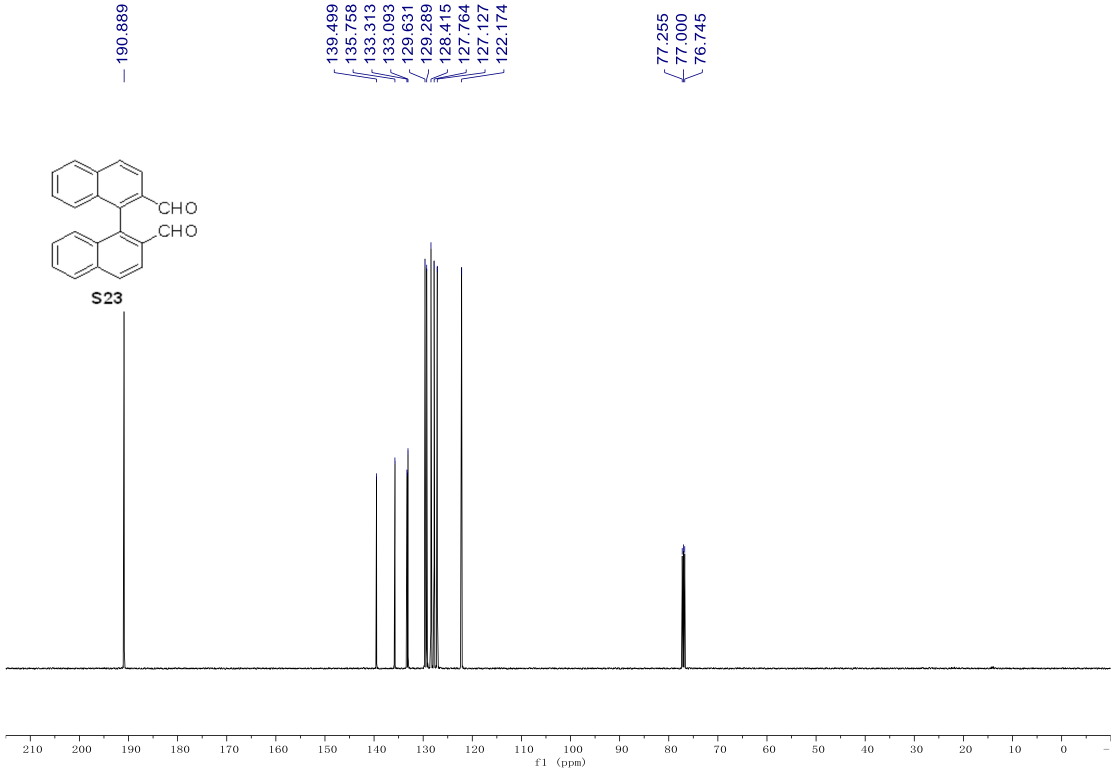
190.889
139.499
135.758
133.313
133.093
129.631
129.289
128.415
127.764
127.127
122.174
77.255
77.000
76.745
CHO
CHO
S23
f1 (ppm)

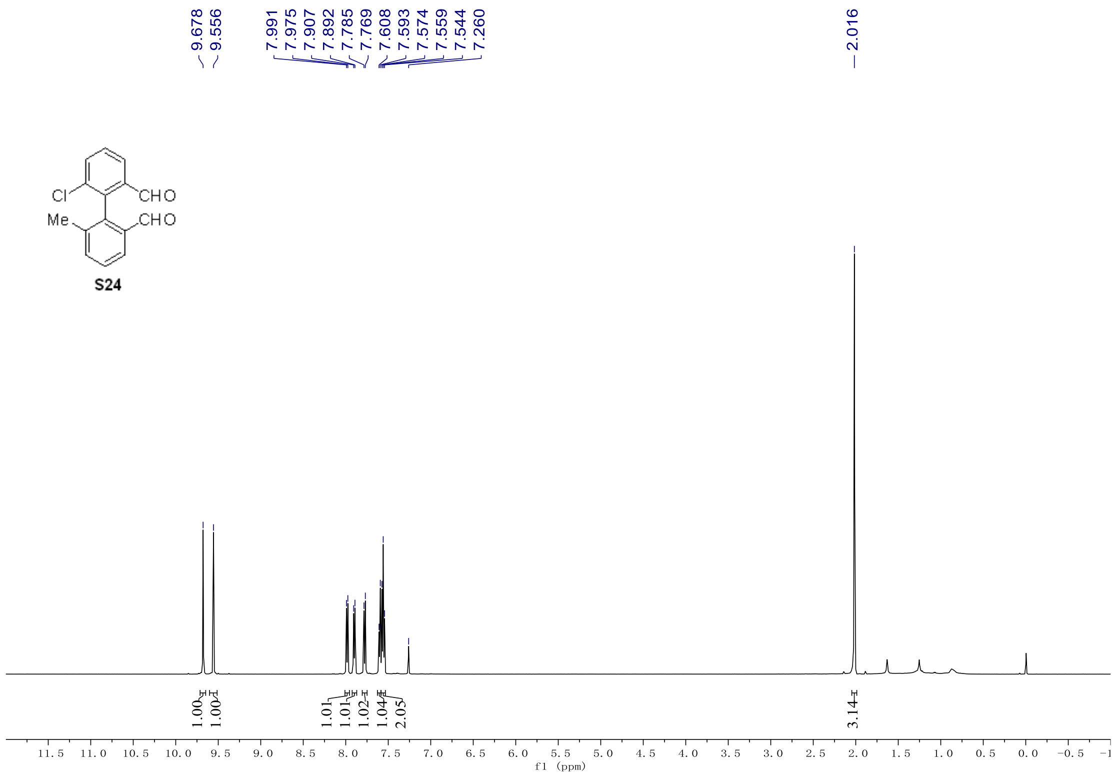
9.678
9.556
7.991
7.975
7.907
7.892
7.785
7.769
7.608
7.593
7.574
7.559
7.544
7.260
2.016
Cl
Me
CHO
CHO
S24
1.00
1.00
1.01
1.01
1.02
1.04
2.05
3.14
f1 (ppm)

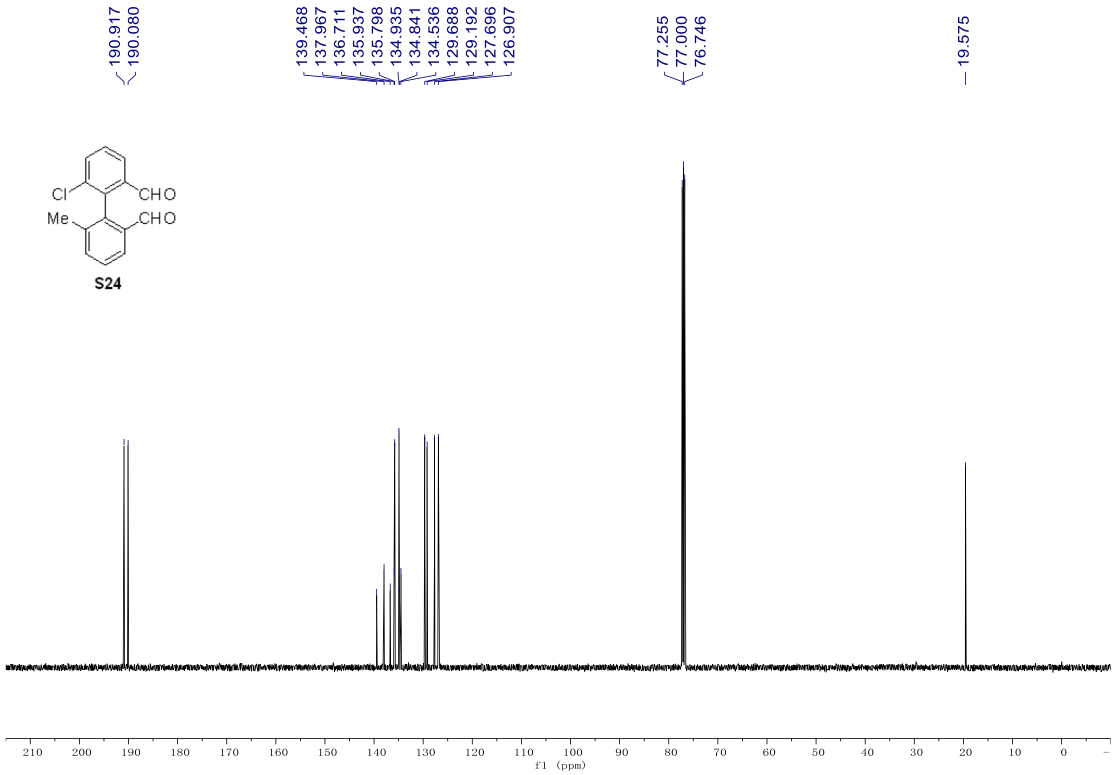
190.917
190.080
139.468
137.967
136.711
135.937
135.798
134.935
134.841
134.536
129.688
129.192
127.696
126.907
77.255
77.000
76.746
19.575
Cl
Me
CHO
CHO
S24
f1 (ppm)

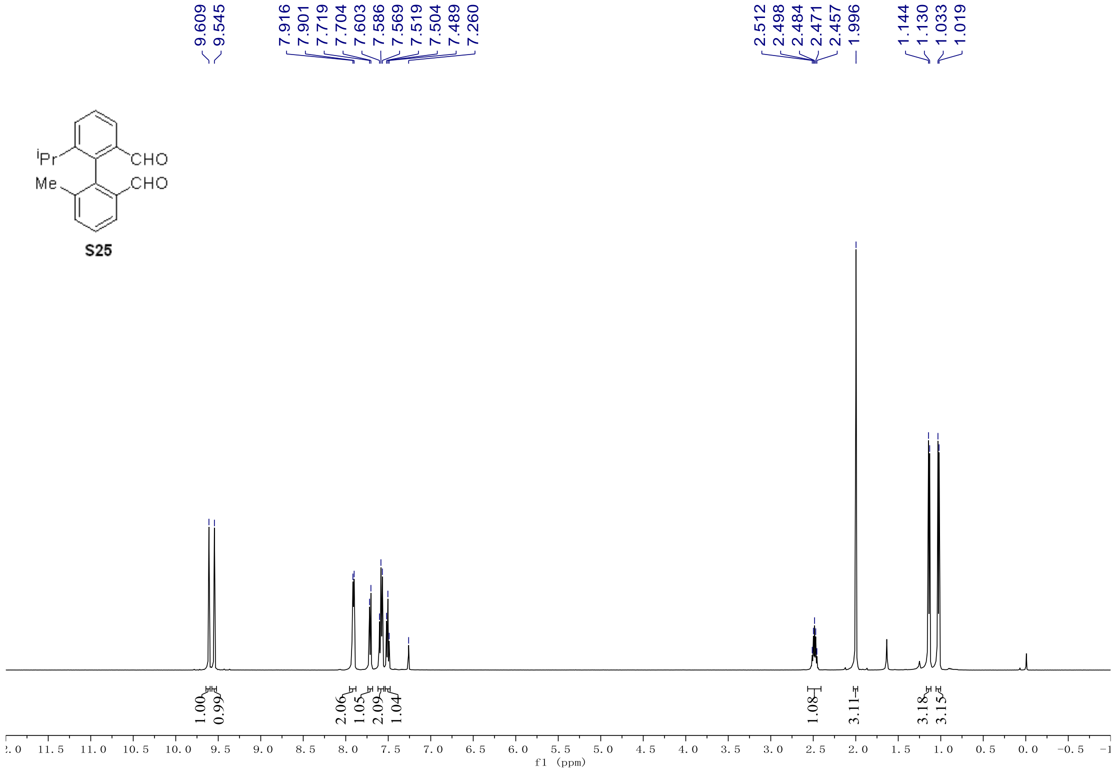
9.609
9.545
7.916
7.901
7.719
7.704
7.603
7.586
7.569
7.519
7.504
7.489
7.260
2.512
2.498
2.484
2.471
2.457
1.996
1.144
1.130
1.033
1.019
iPr
Me
CHO
CHO
S25
1.00
0.99
2.06
1.05
2.09
1.04
1.08
3.11
3.18
3.15
f1 (ppm)

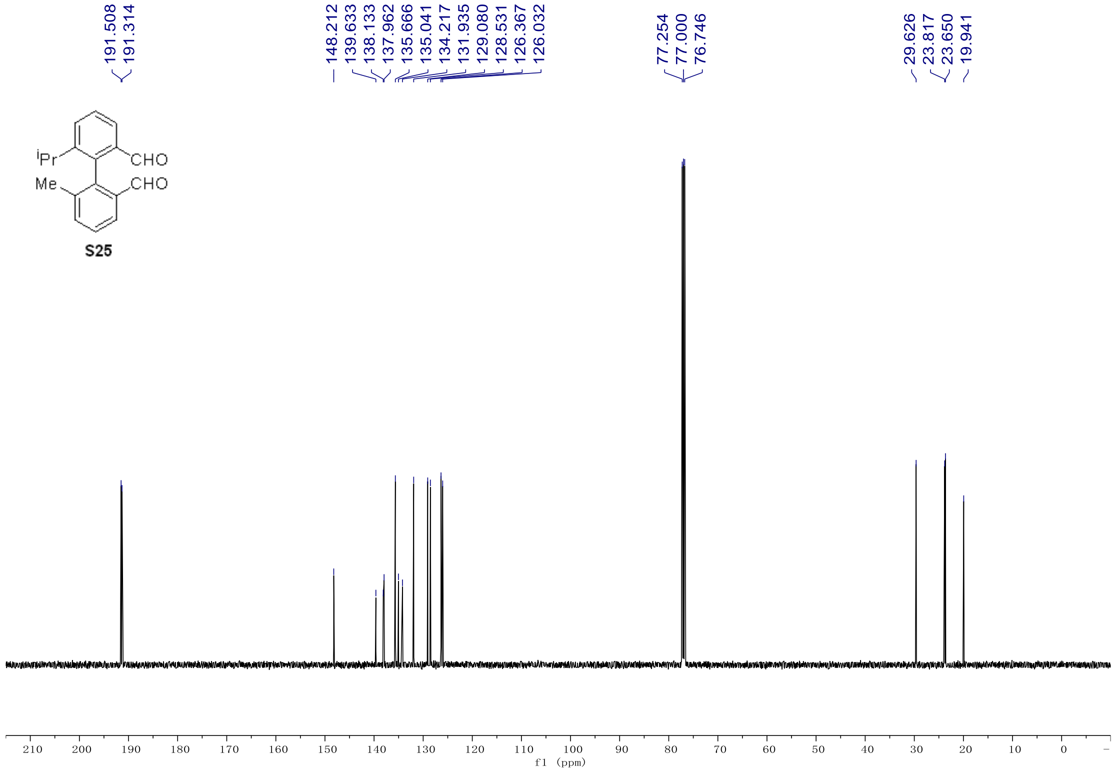
191.508
191.314
148.212
139.633
138.133
137.962
135.666
135.041
134.217
131.935
129.080
128.531
126.367
126.032
77.254
77.000
76.746
29.626
23.817
23.650
19.941
iPr
Me
CHO
CHO
S25
f1 (ppm)

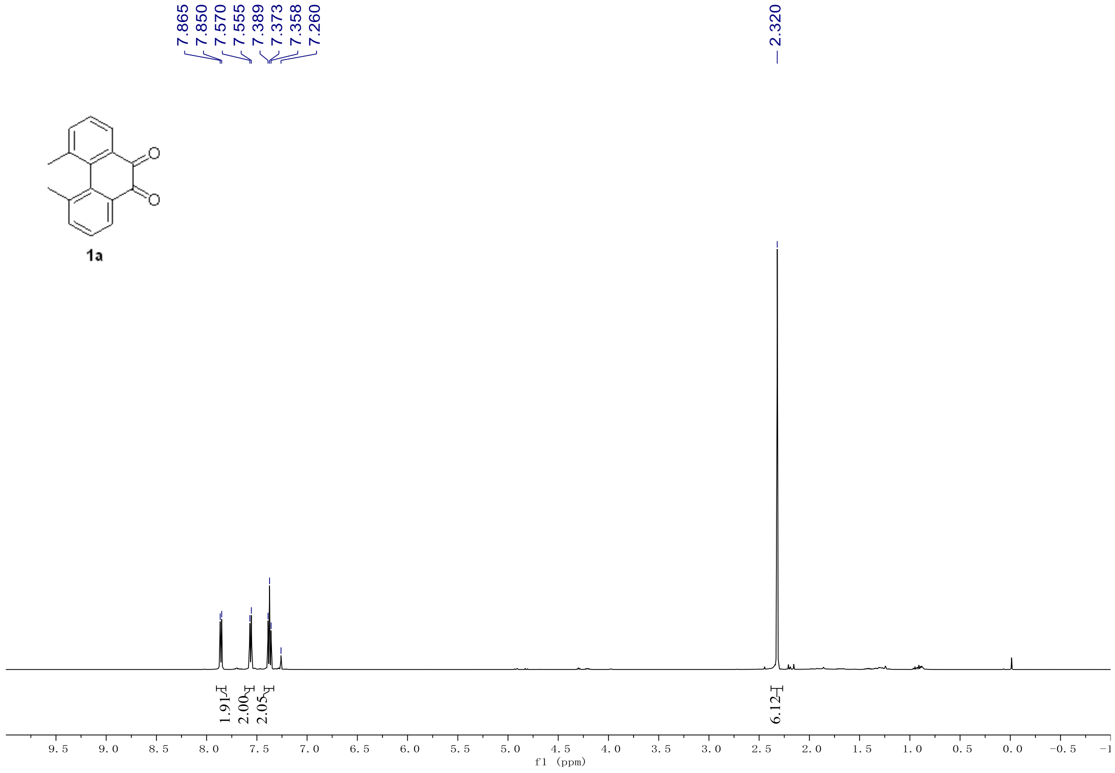
7.865
7.850
7.570
7.555
7.389
7.373
7.358
7.260
2.320
1a
1.91
2.00
2.05
6.12
9.5 9.0 8.5 8.0 7.5 7.0 6.5 6.0 5.5 5.0 4.5 4.0 3.5 3.0 2.5 2.0 1.5 1.0 0.5 0.0 -0.5 -1
f1 (ppm)

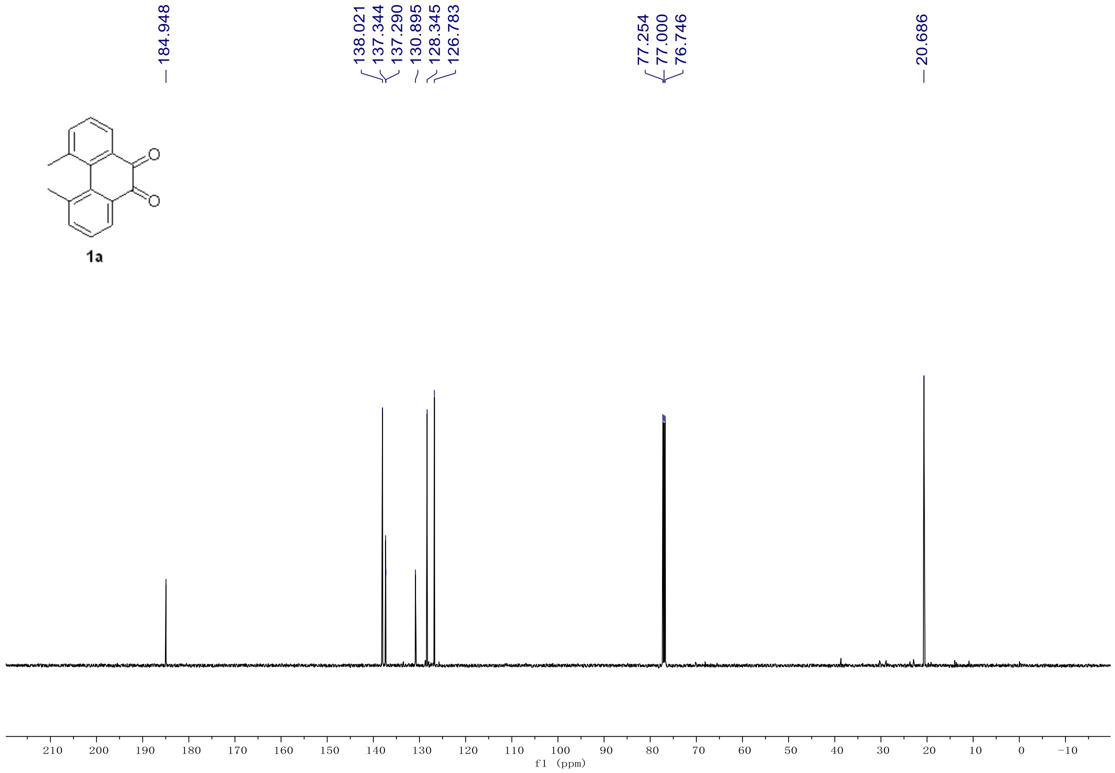
184.948
138.021
137.344
137.290
130.895
128.345
126.783
77.254
77.000
76.746
20.686
1a
210 200 190 180 170 160 150 140 130 120 110 100 90 80 70 60 50 40 30 20 10 0 -10
f1 (ppm)

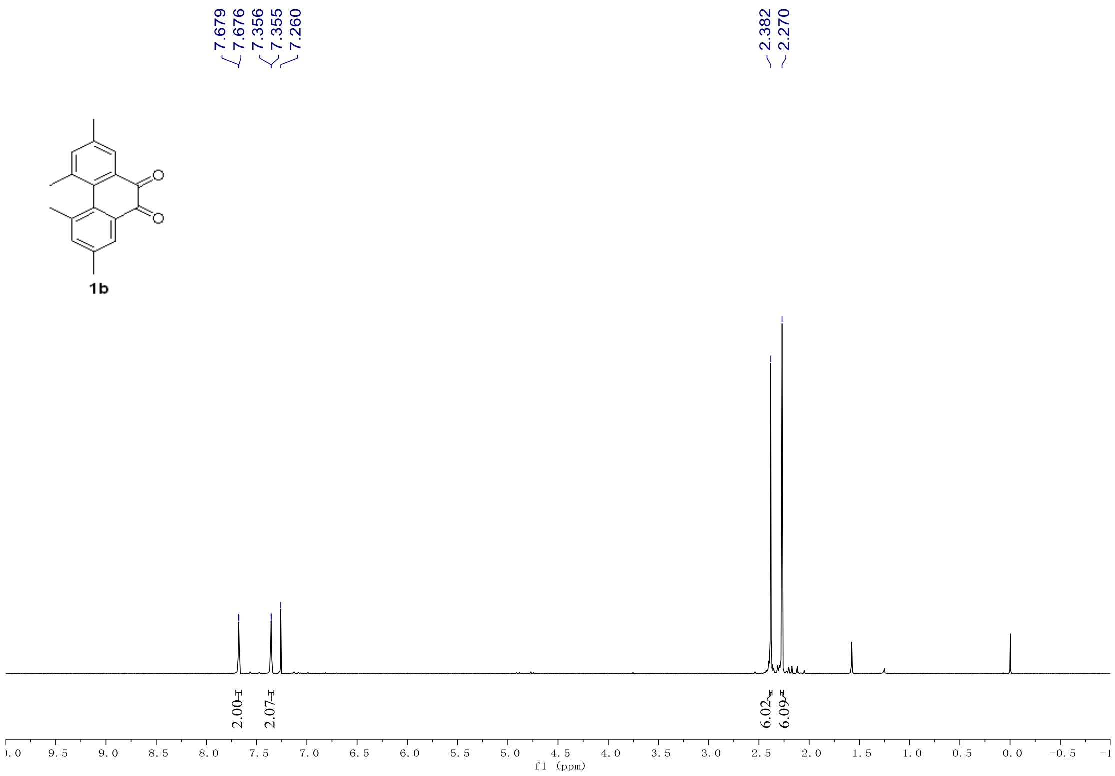
7.679
7.676
7.356
7.355
7.260
2.382
2.270
1b
2.00
2.07
6.02
6.09
f1 (ppm)

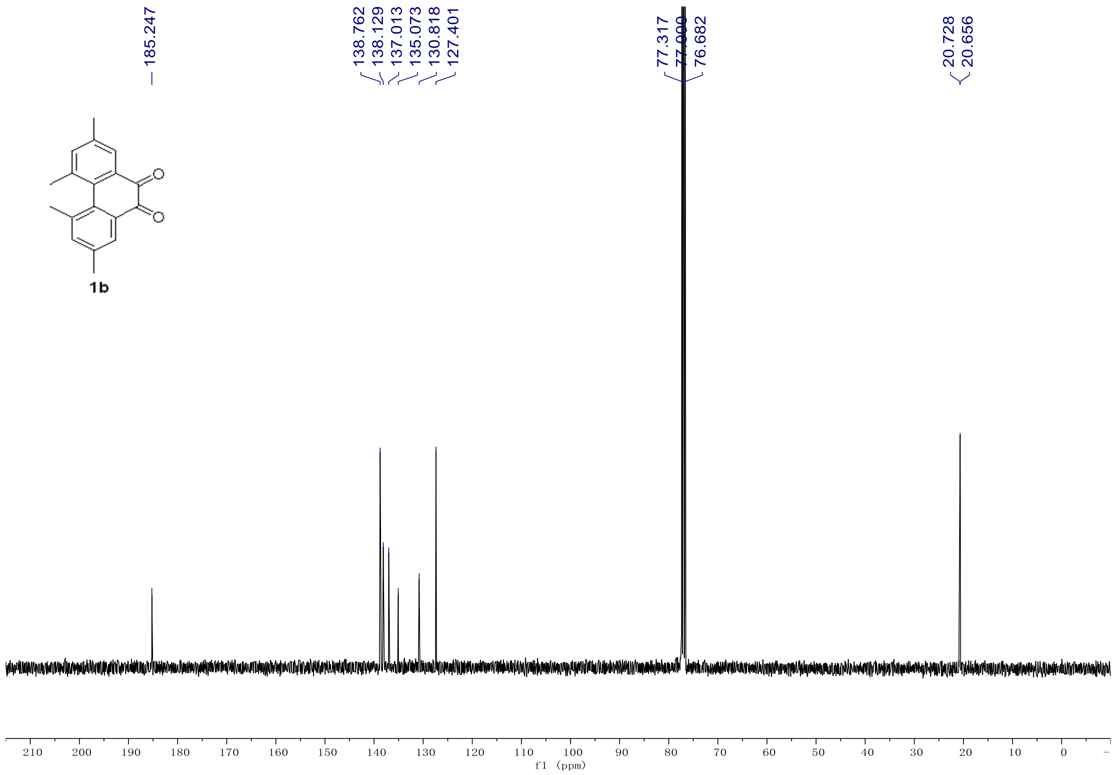
185.247
138.762
138.129
137.013
135.073
130.818
127.401
77.317
76.682
20.728
20.656
1b
f1 (ppm)

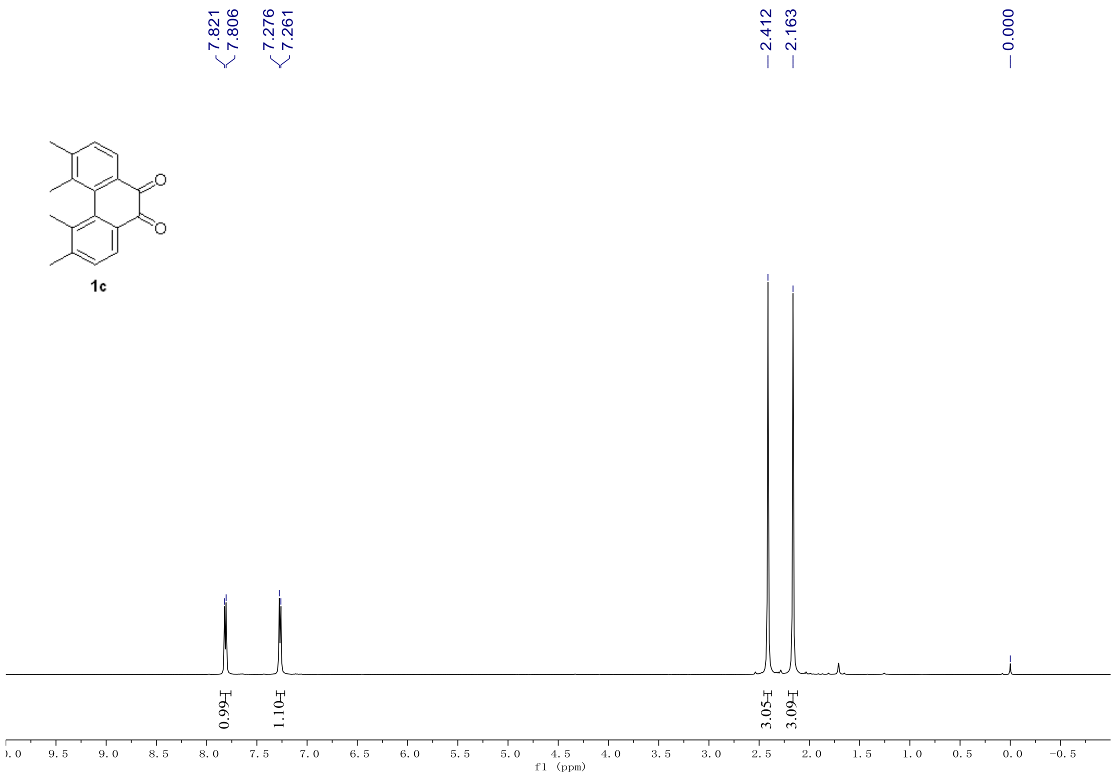
7.821
7.806
7.276
7.261
2.412
2.163
0.000
1c
0.99
1.10
3.05
3.09
f1 (ppm)

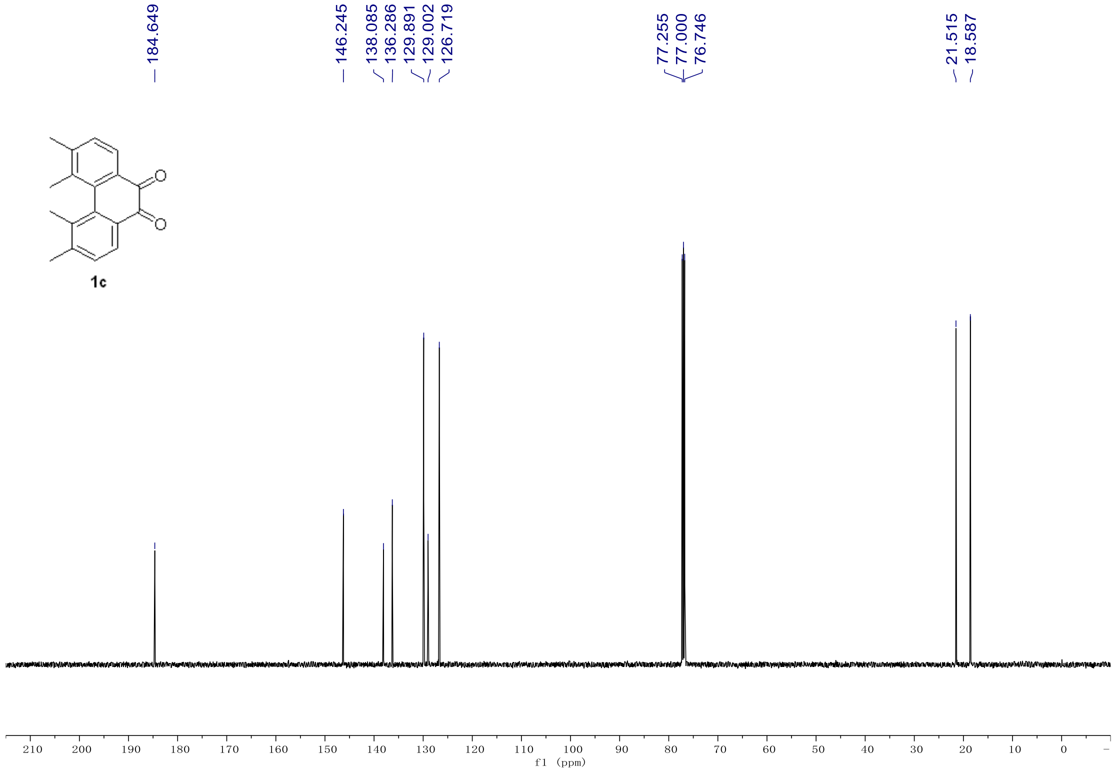
184.649
146.245
138.085
136.286
129.891
129.002
126.719
77.255
77.000
76.746
21.515
18.587
1c
f1 (ppm)

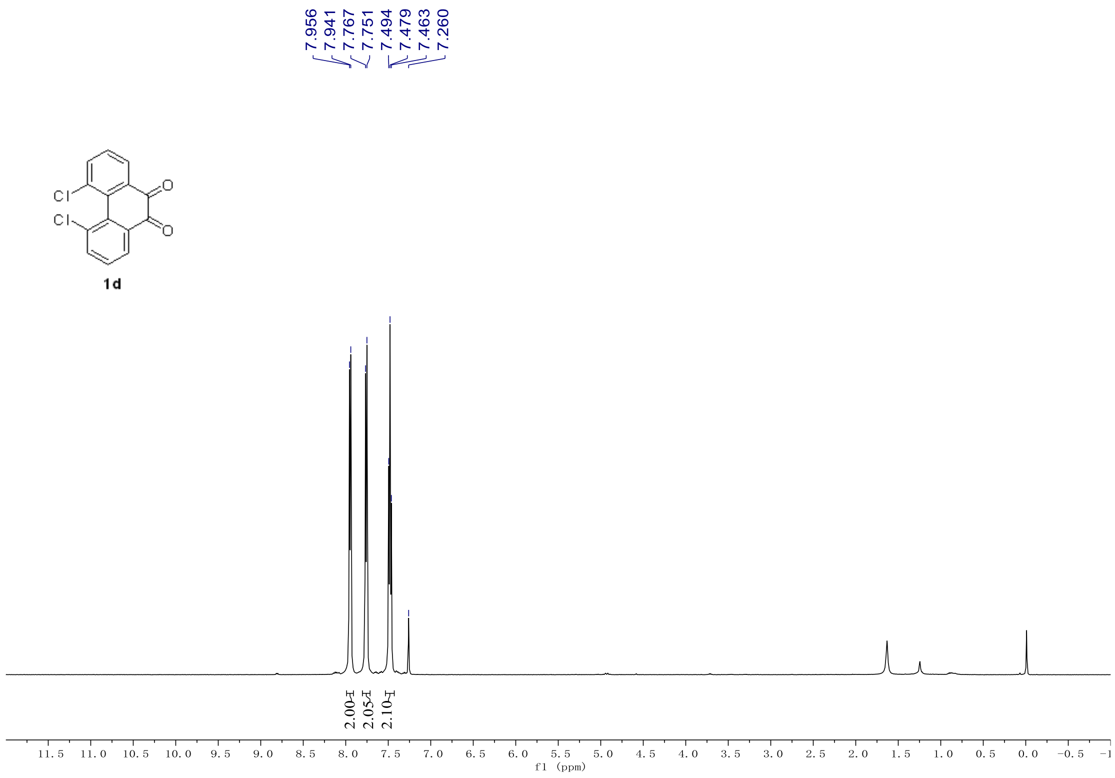

7.956
7.941
7.767
7.751
7.494
7.479
7.463
7.260
Cl
Cl
O
O
1d
2.00
2.05
2.10
11.5 11.0 10.5 10.0 9.5 9.0 8.5 8.0 7.5 7.0 6.5 6.0 5.5 5.0 4.5 4.0 3.5 3.0 2.5 2.0 1.5 1.0 0.5 0.0 -0.5 -1
f1 (ppm)

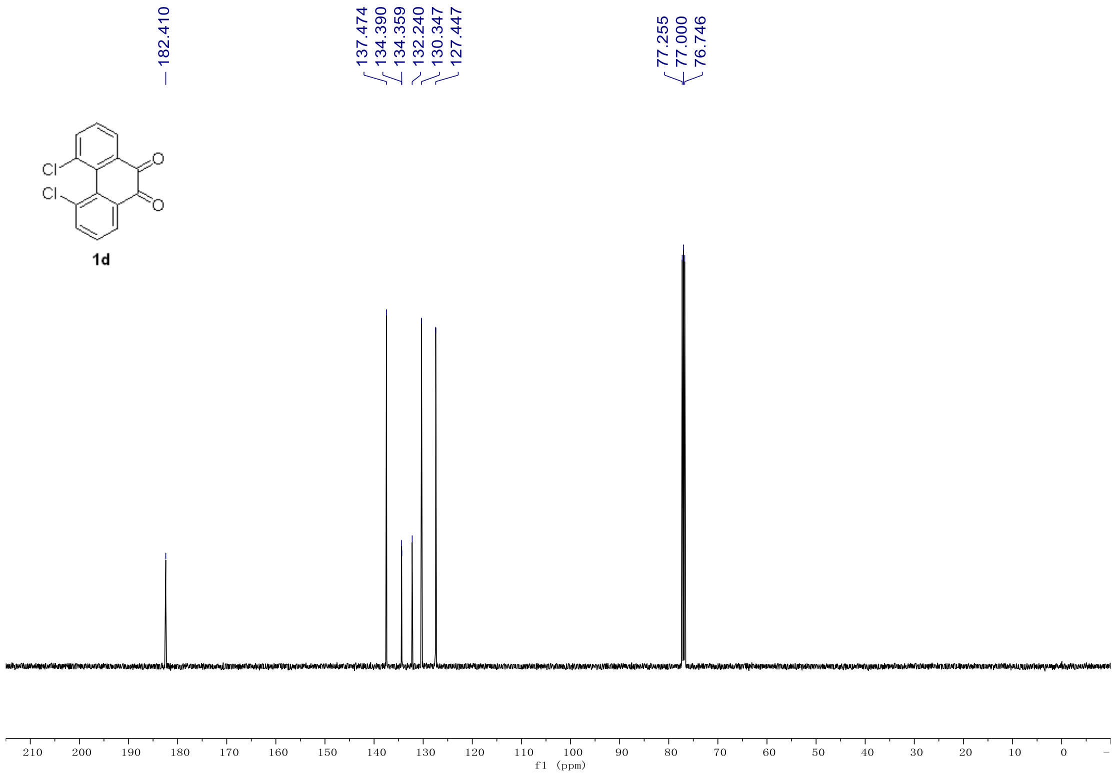

182.410
137.474
134.390
134.359
132.240
130.347
127.447
77.255
77.000
76.746
Cl
Cl
O
O
1d
210 200 190 180 170 160 150 140 130 120 110 100 90 80 70 60 50 40 30 20 10 0
f1 (ppm)

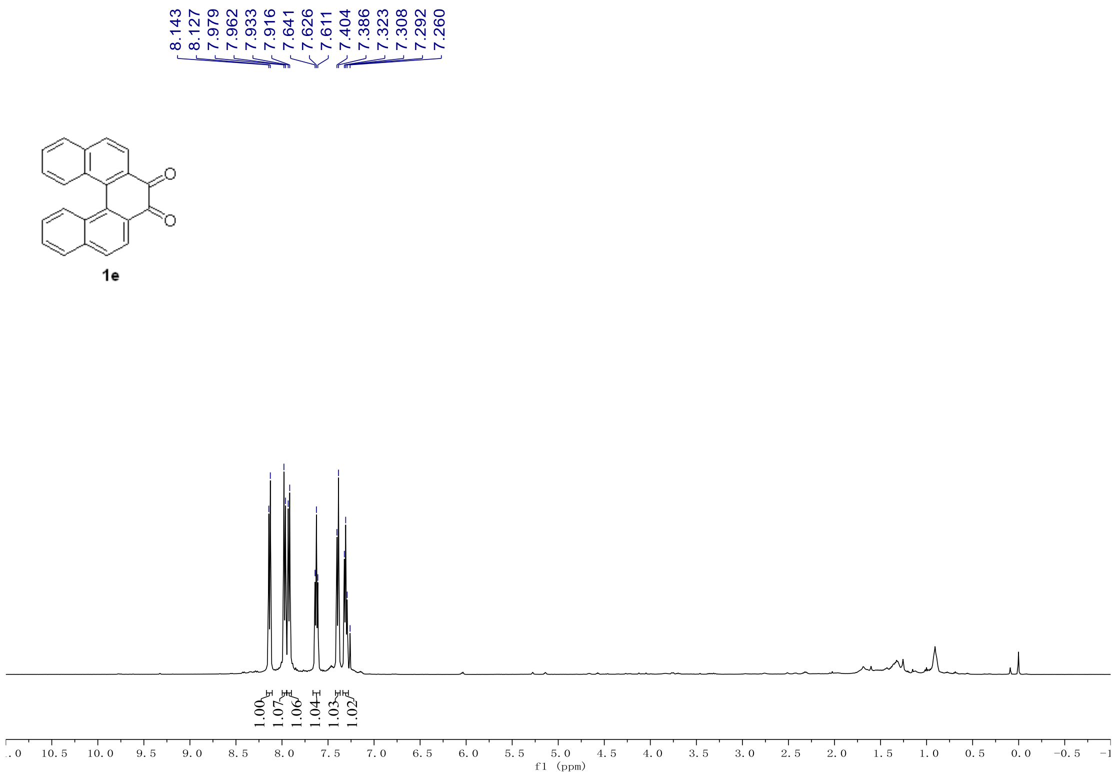
8.143
8.127
7.979
7.962
7.933
7.916
7.641
7.626
7.611
7.404
7.386
7.323
7.308
7.292
7.260
1e
1.00
1.07
1.06
1.04
1.03
1.02
f1 (ppm)

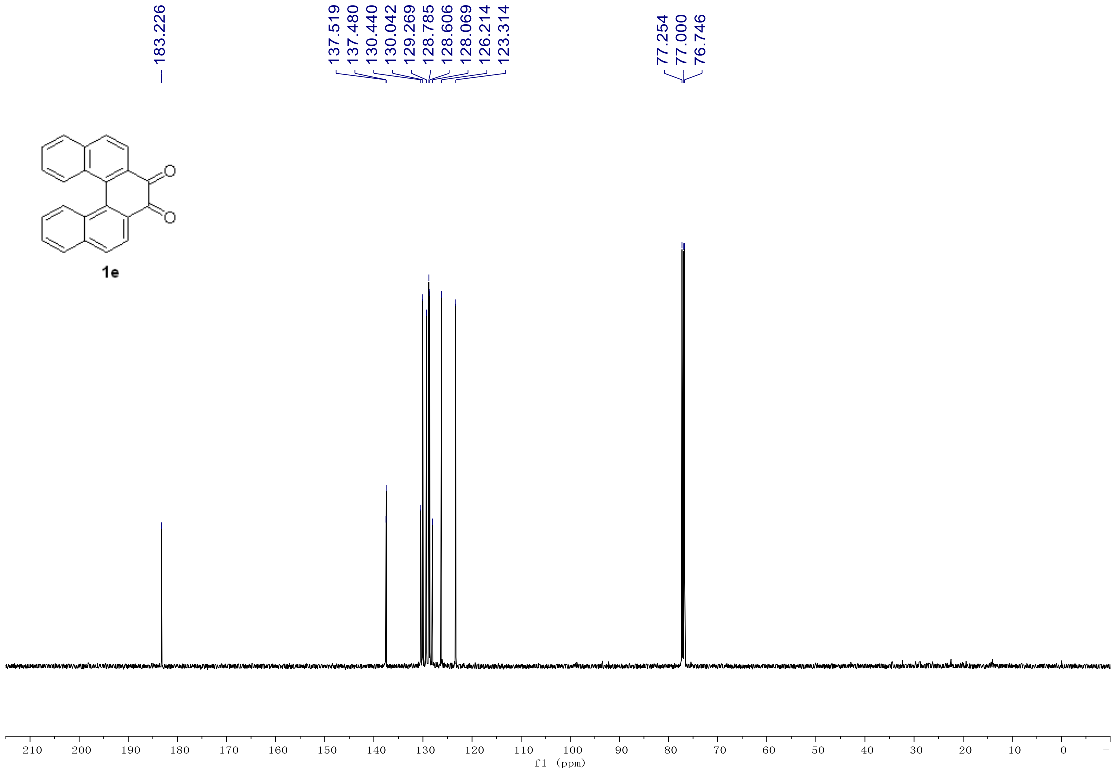
183.226
137.519
137.480
130.440
130.042
129.269
128.785
128.606
128.069
126.214
123.314
77.254
77.000
76.746
1e
f1 (ppm)

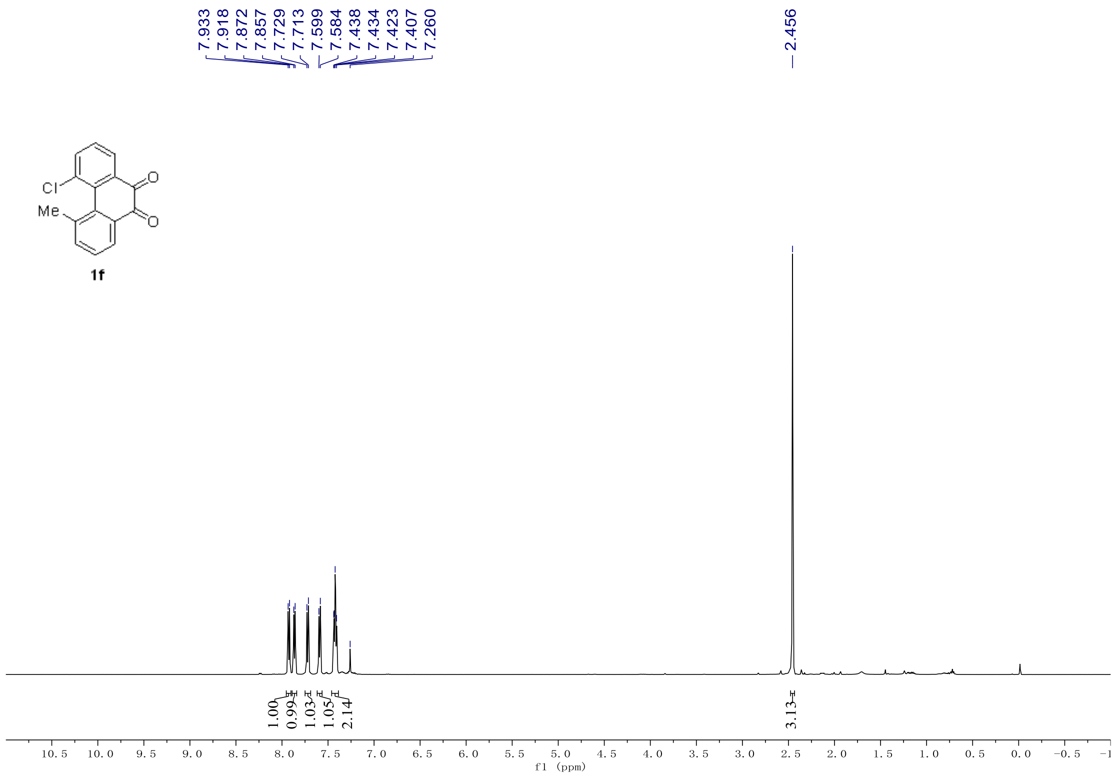
7.933
7.918
7.872
7.857
7.729
7.713
7.599
7.584
7.438
7.434
7.423
7.407
7.260
2.456
Cl
Me
O
O
1f
1.00
0.99
1.03
1.05
2.14
3.13
f1 (ppm)

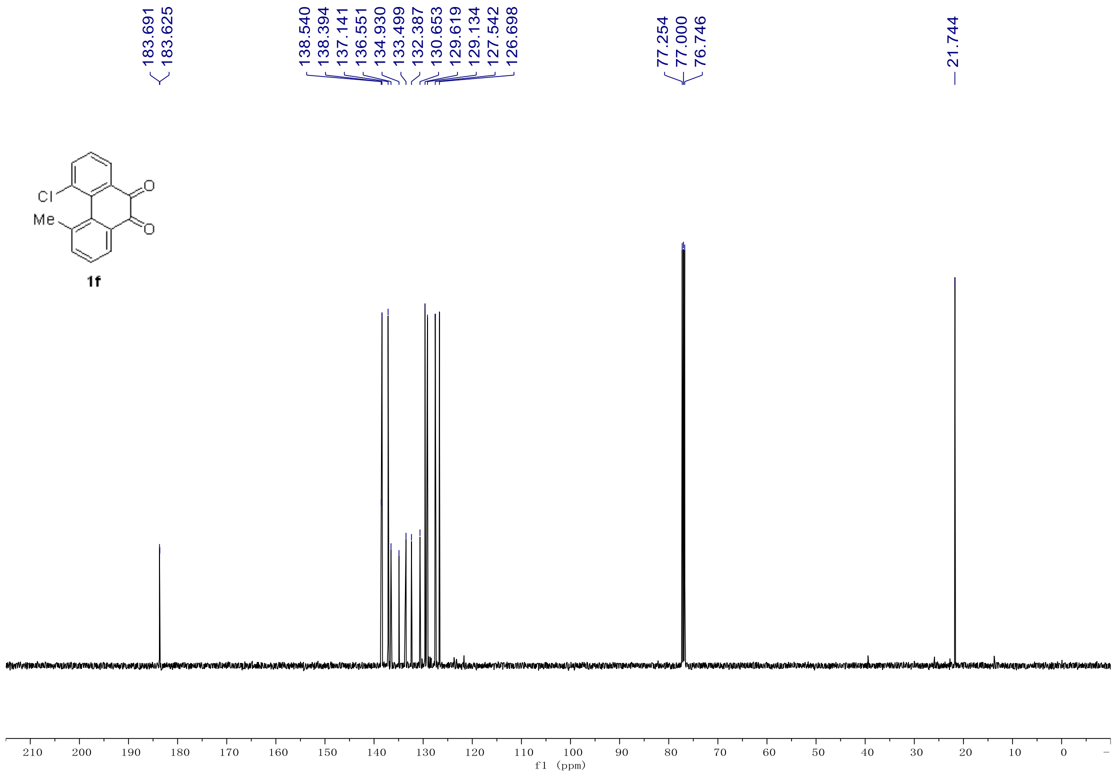
183.691
183.625
138.540
138.394
137.141
136.551
134.930
133.499
132.387
130.653
129.619
129.134
127.542
126.698
77.254
77.000
76.746
21.744
Cl
Me
O
O
1f
f1 (ppm)

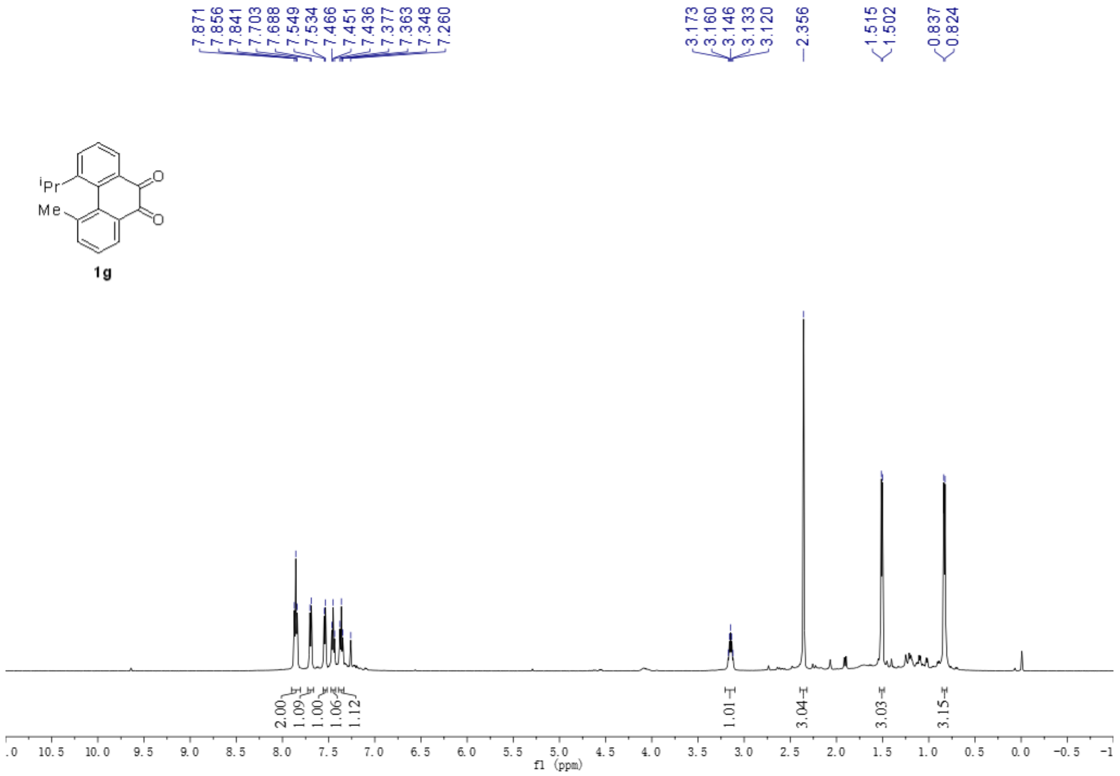
7.871
7.856
7.841
7.703
7.688
7.549
7.534
7.466
7.451
7.436
7.377
7.363
7.348
7.260
3.173
3.160
3.146
3.133
3.120
2.356
1.515
1.502
0.837
0.824
iPr
Me
O
O
1g
2.00
1.09
1.00
1.06
1.12
1.01
3.04
3.03
3.15
f1 (ppm)

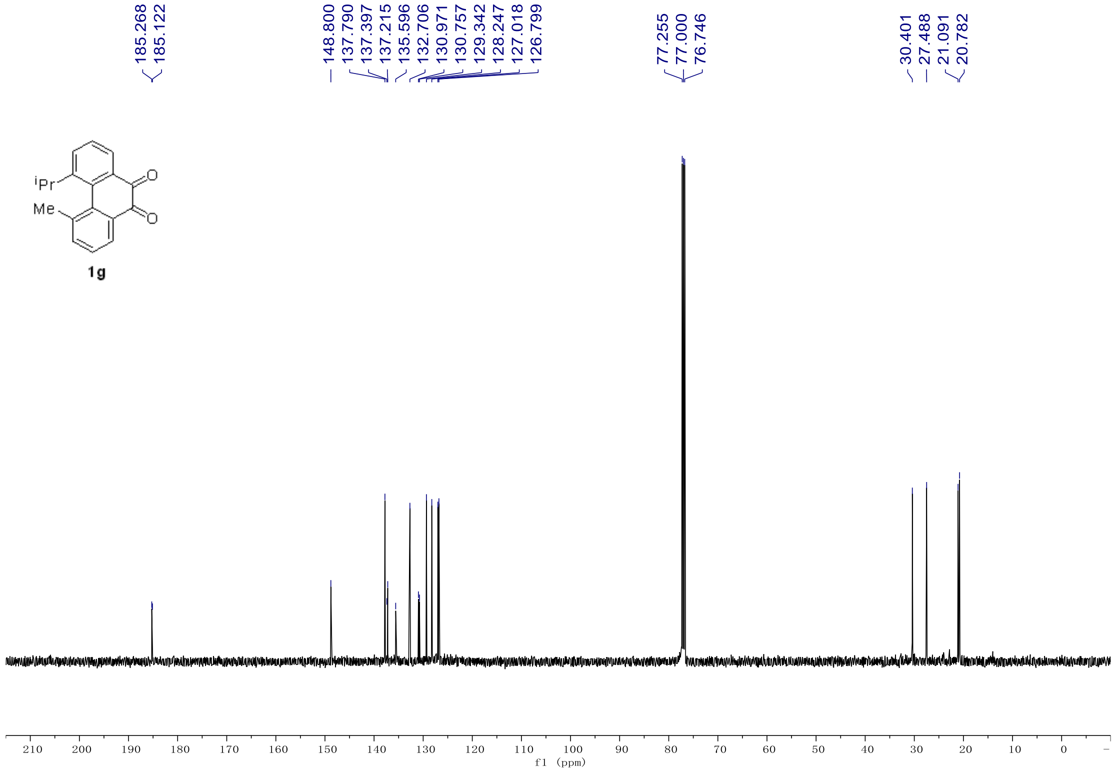
185.268
185.122
148.800
137.790
137.397
137.215
135.596
132.706
130.971
130.757
129.342
128.247
127.018
126.799
77.255
77.000
76.746
30.401
27.488
21.091
20.782
iPr
Me
O
O
1g
f1 (ppm)

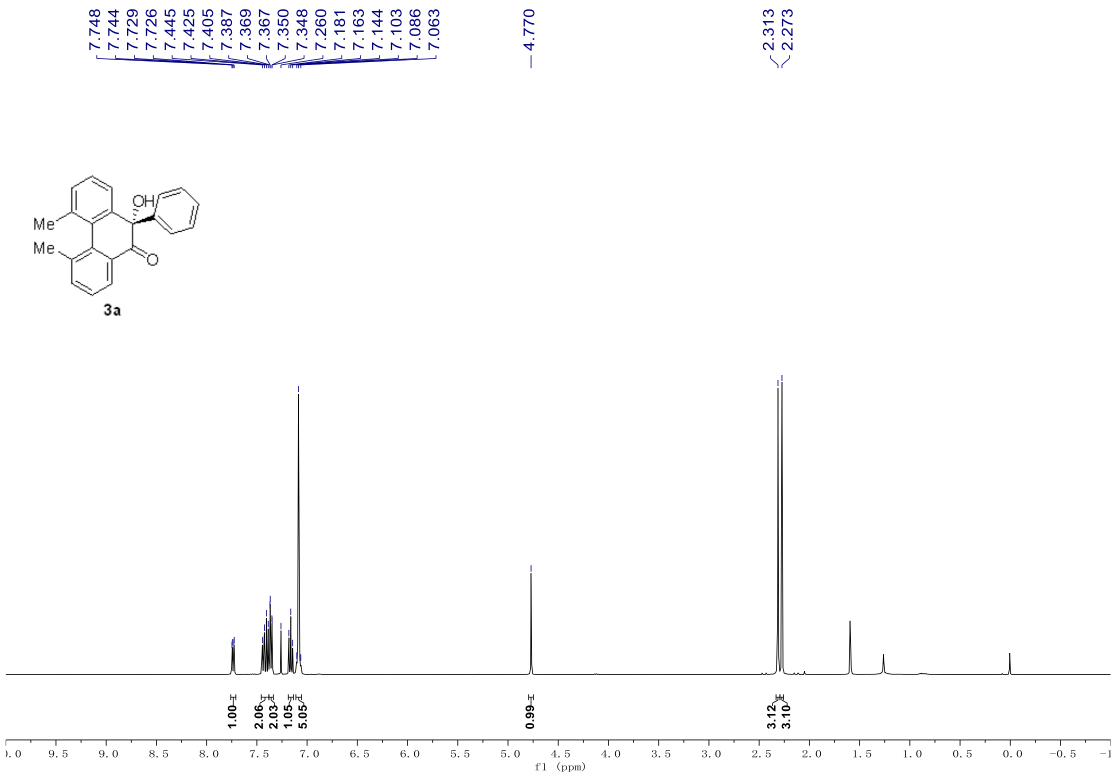
7.748
7.744
7.729
7.726
7.445
7.425
7.405
7.387
7.369
7.367
7.350
7.348
7.260
7.181
7.163
7.144
7.103
7.086
7.063
4.770
2.313
2.273
Me
Me
OH
O
3a
1.00
2.06
2.03
1.05
5.05
0.99
3.12
3.10
f1 (ppm)

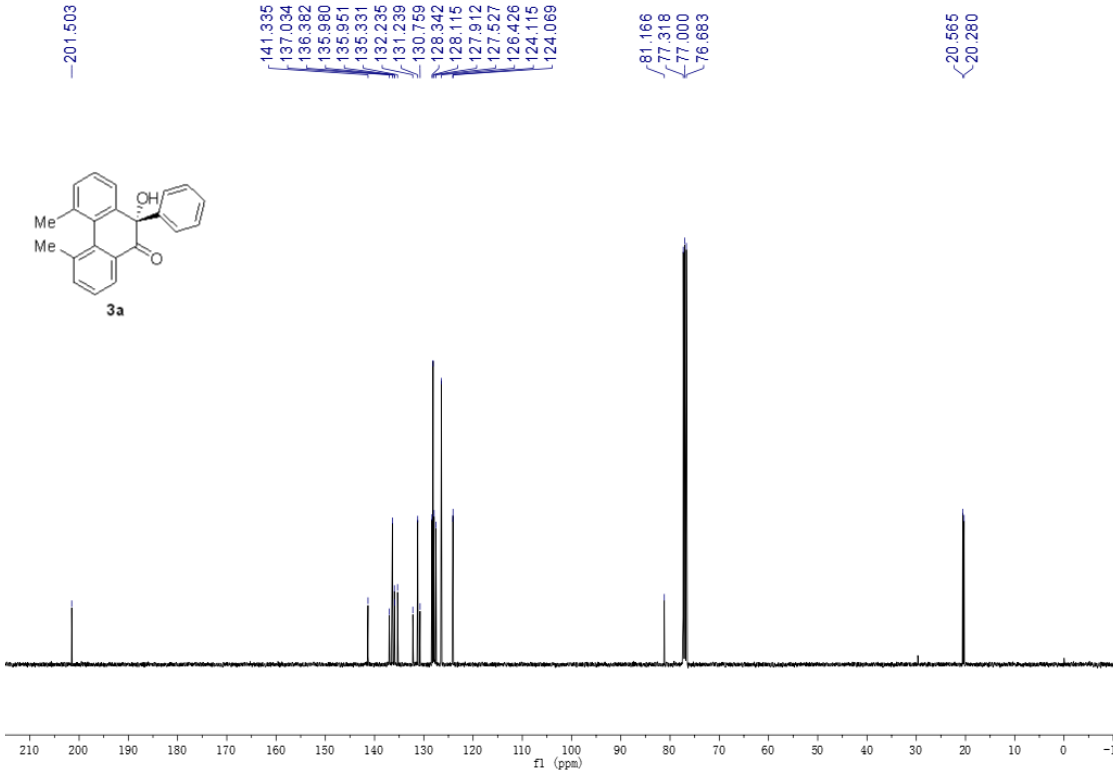
201.503
141.335
137.034
136.382
135.980
135.951
135.331
132.235
131.239
130.759
128.342
128.115
127.912
127.527
126.426
124.115
124.069
81.166
77.318
77.000
76.683
20.565
20.280
Me
Me
OH
O
3a
f1 (ppm)

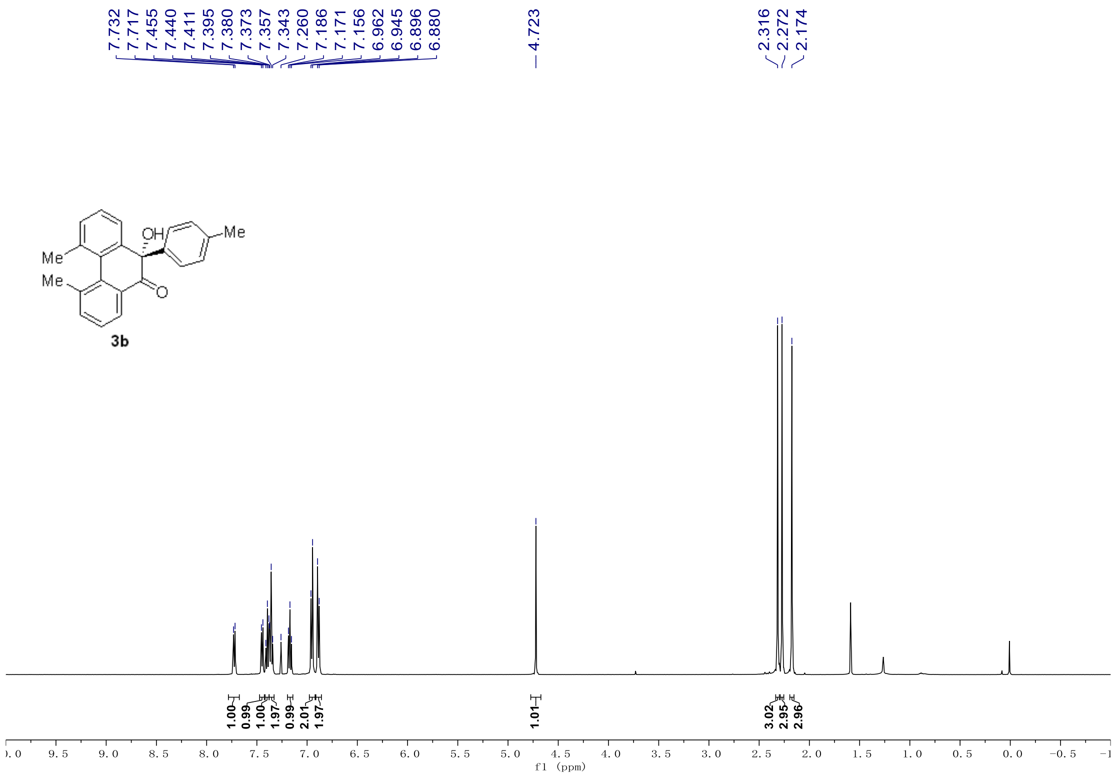
7.732
7.717
7.455
7.440
7.411
7.395
7.380
7.373
7.357
7.343
7.260
7.186
7.171
7.156
6.962
6.945
6.896
6.880
4.723
2.316
2.272
2.174
OH
Me
Me
Me
3b
1.00
0.99
1.00
1.97
0.99
2.01
1.97
1.01
3.02
2.95
2.96
f1 (ppm)

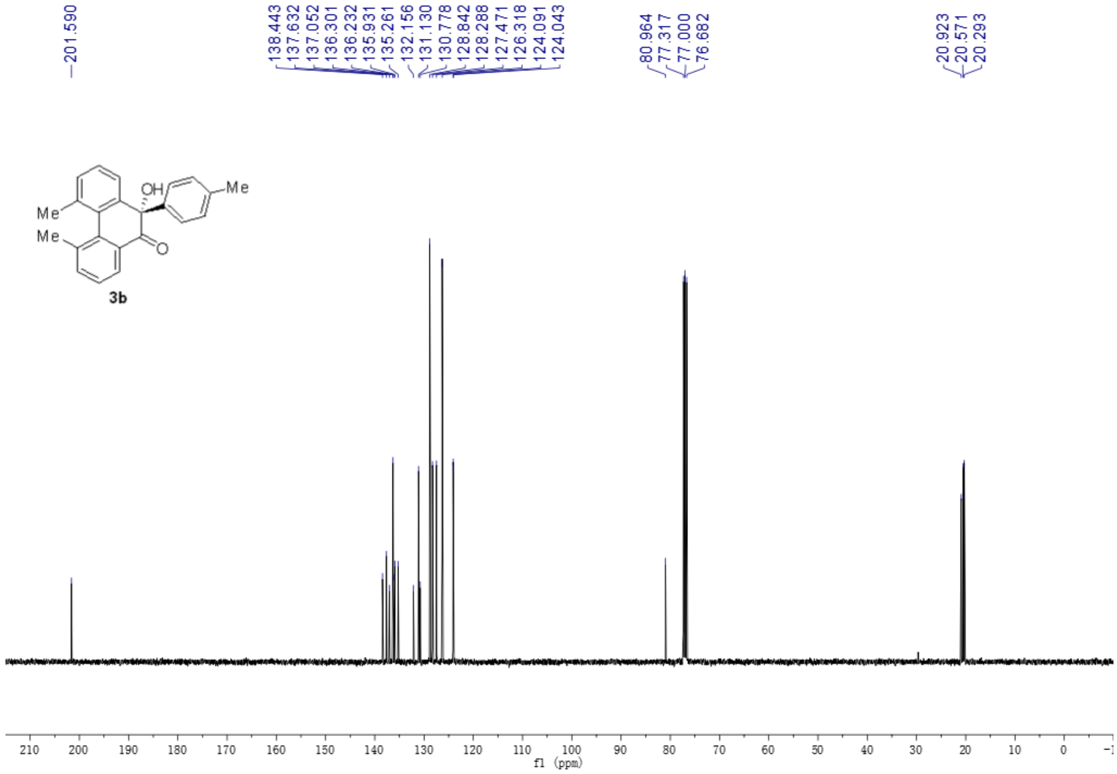
201.590
138.443
137.632
137.052
136.301
136.232
135.931
135.261
132.156
131.130
130.778
128.842
128.288
127.471
126.318
124.091
124.043
80.964
77.317
77.000
76.682
20.923
20.571
20.293
OH
Me
Me
Me
3b
f1 (ppm)

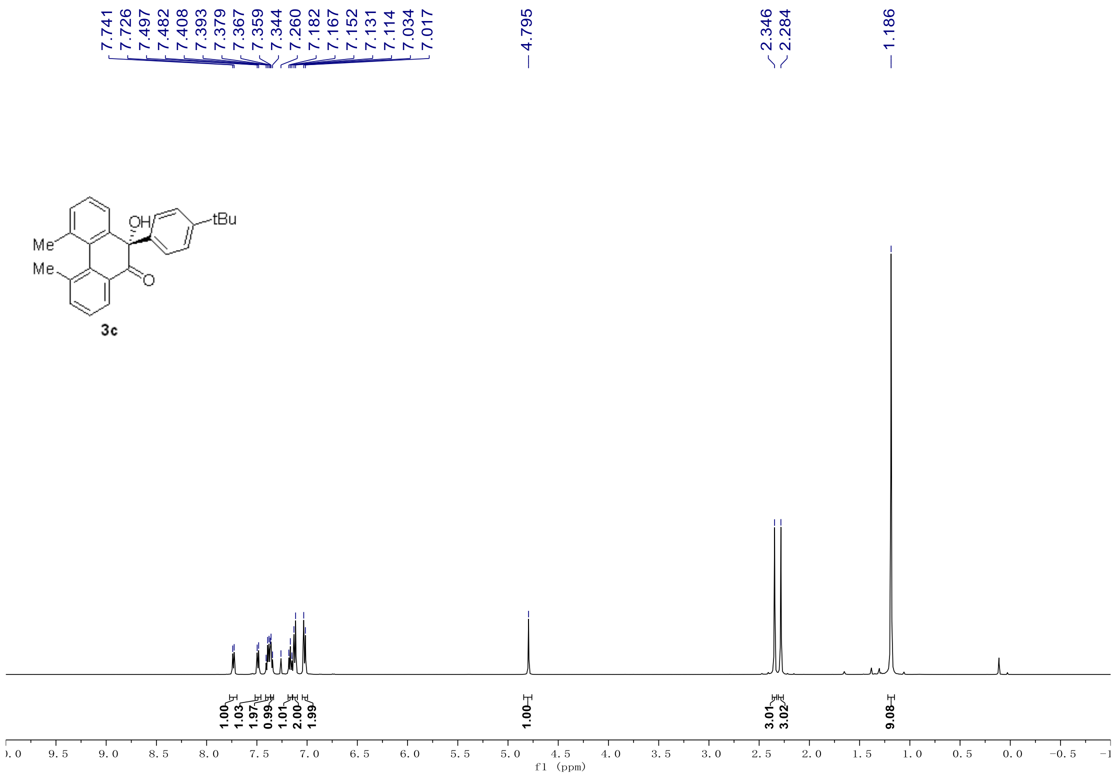
7.741
7.726
7.497
7.482
7.408
7.393
7.379
7.367
7.359
7.344
7.260
7.182
7.167
7.152
7.131
7.114
7.034
7.017
4.795
2.346
2.284
1.186
OH
tBu
Me
Me
O
3c
1.00
1.03
1.97
0.99
1.01
2.00
1.99
1.00
3.01
3.02
9.08
f1 (ppm)

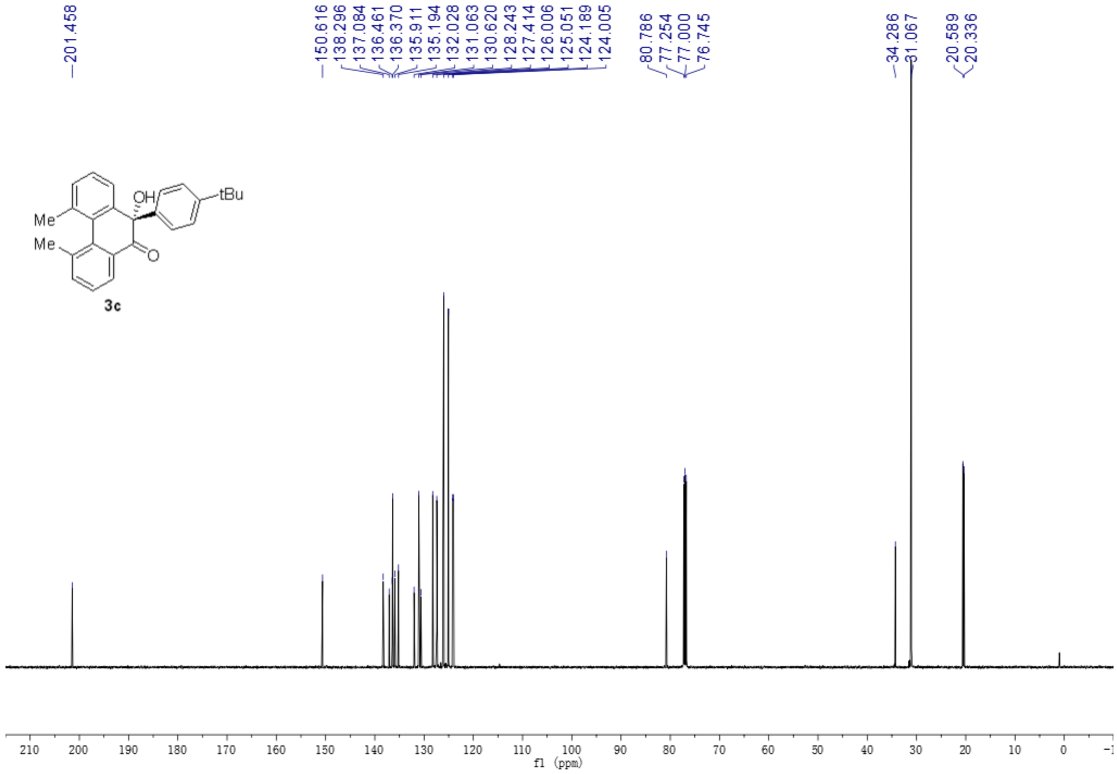
201.458
150.616
138.296
137.084
136.461
136.370
135.911
135.194
132.028
131.063
130.620
128.243
127.414
126.006
125.051
124.189
124.005
80.786
77.254
77.000
76.745
34.286
31.067
20.589
20.336
OH
tBu
Me
Me
O
3c
f1 (ppm)

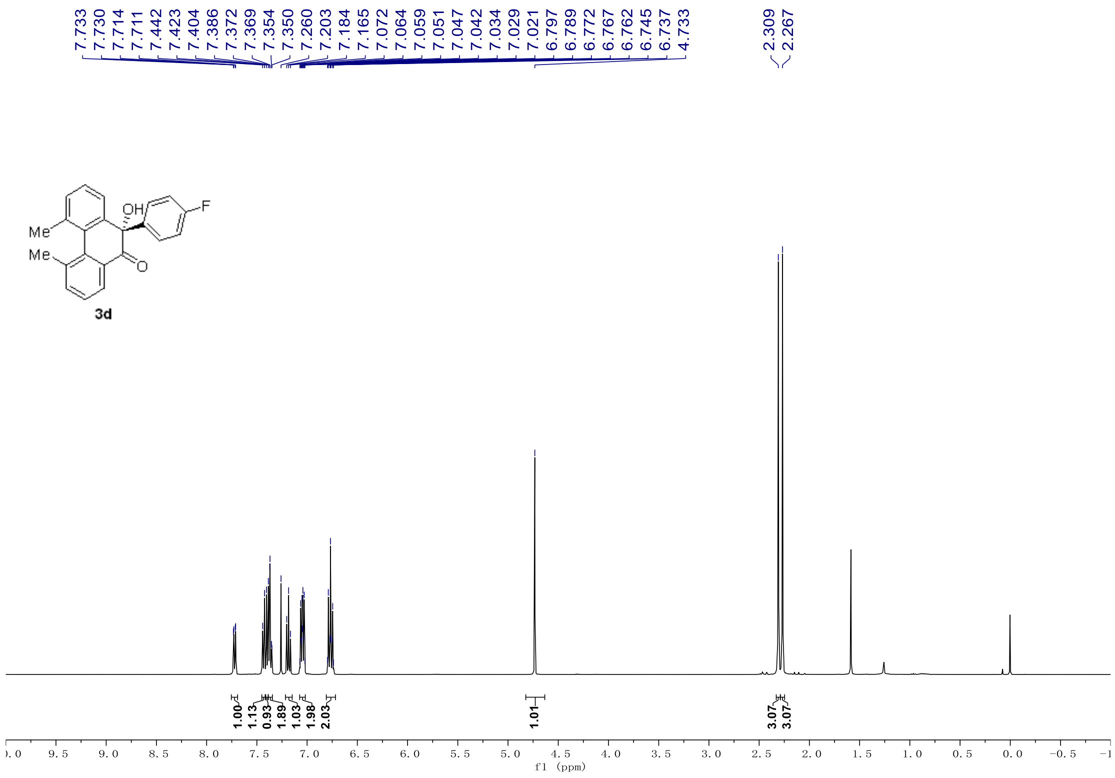

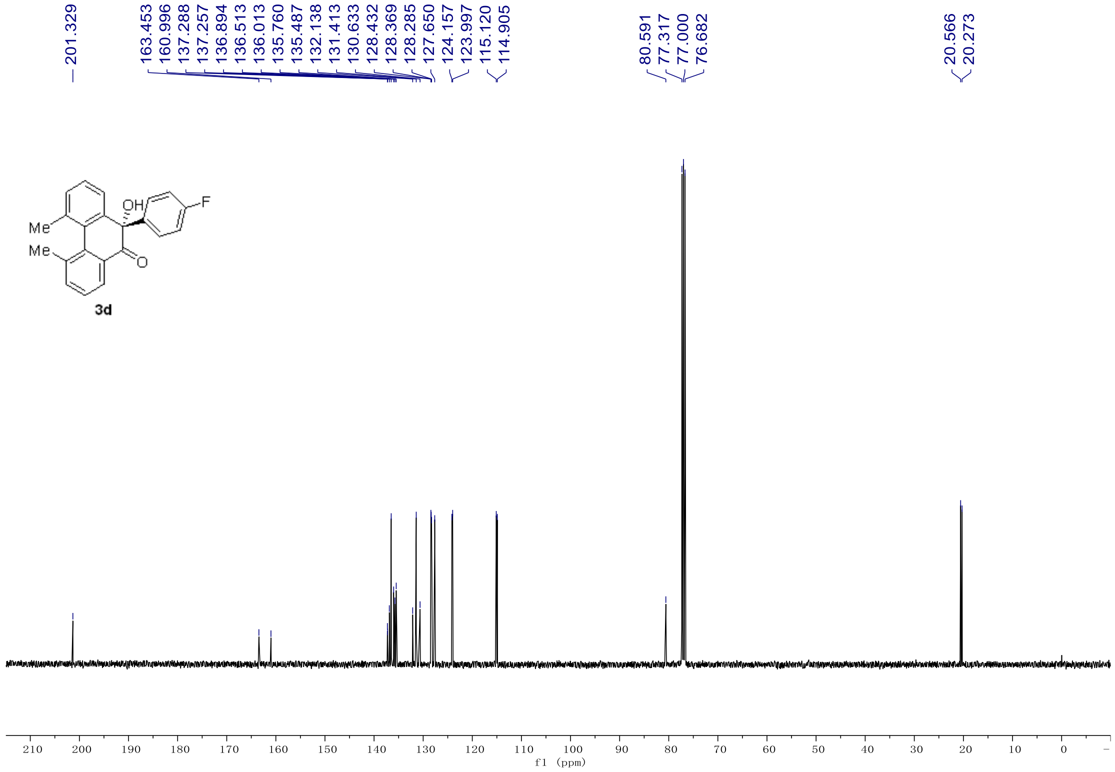

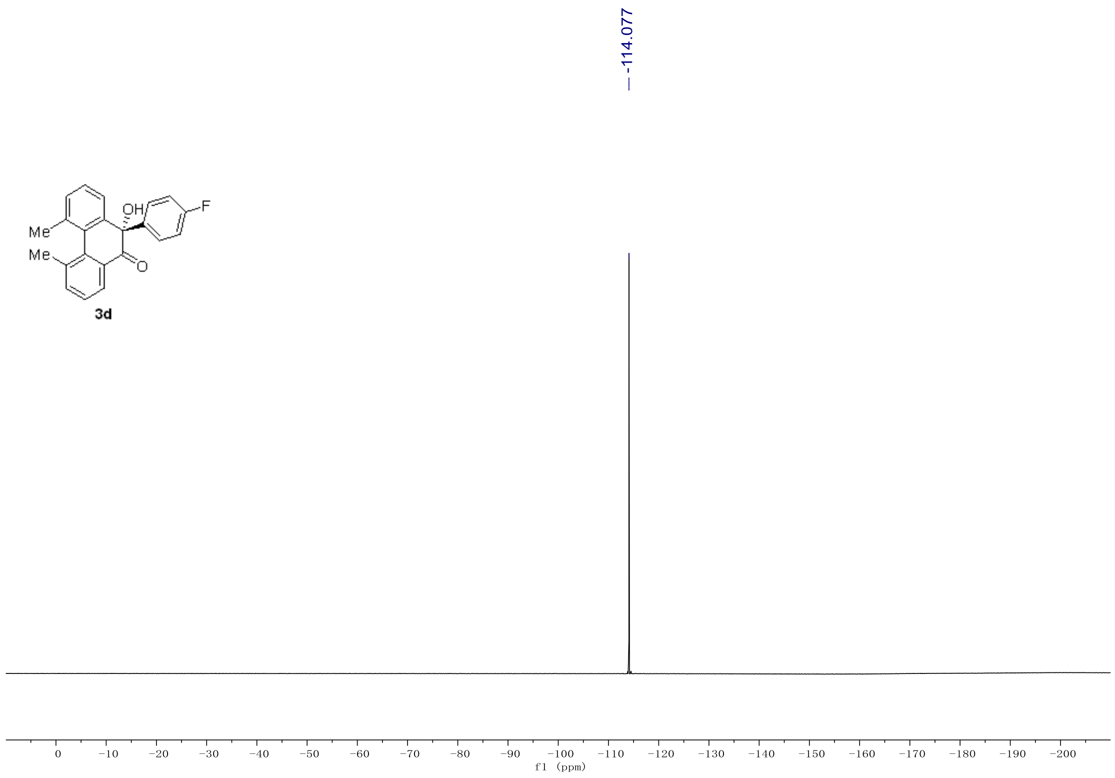
-114.077
OH
F
Me
Me
O
3d
f1 (ppm)

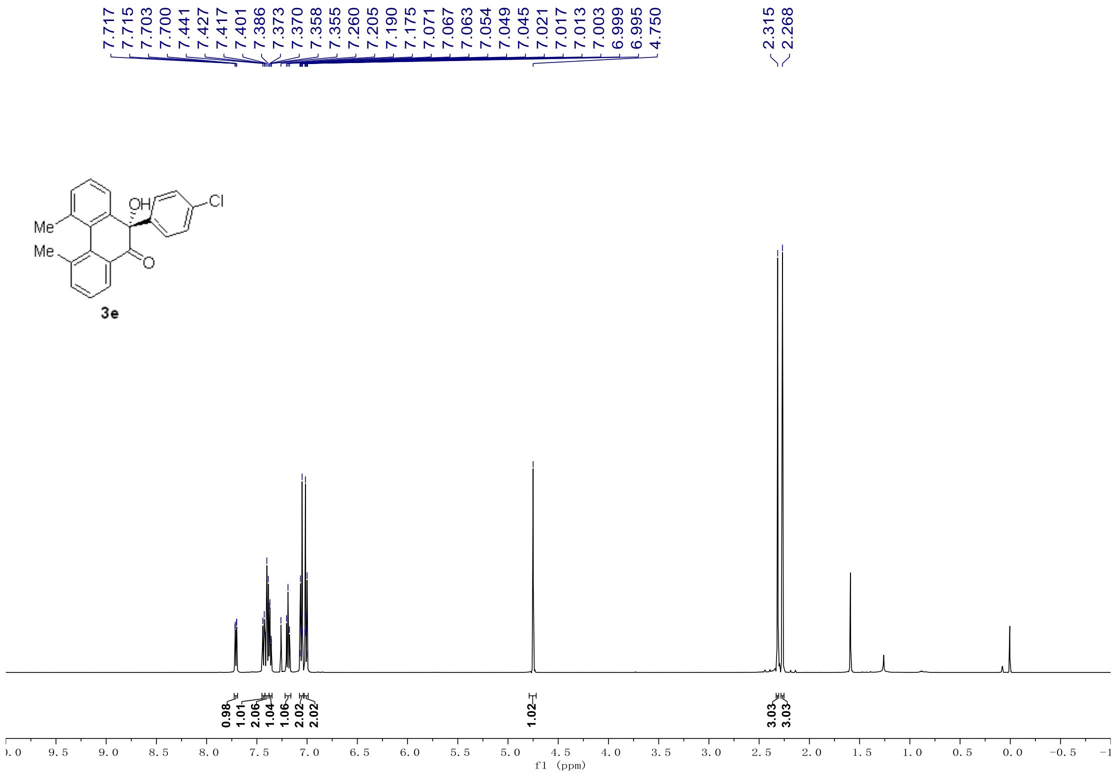
7.717
7.715
7.703
7.700
7.441
7.427
7.417
7.401
7.386
7.373
7.370
7.358
7.355
7.260
7.205
7.190
7.175
7.071
7.067
7.063
7.054
7.049
7.045
7.021
7.017
7.013
7.003
6.999
6.995
4.750
2.315
2.268
OH
Cl
Me
Me
O
3e
0.98
1.01
2.06
1.04
1.06
2.02
2.02
1.02
3.03
3.03
f1 (ppm)

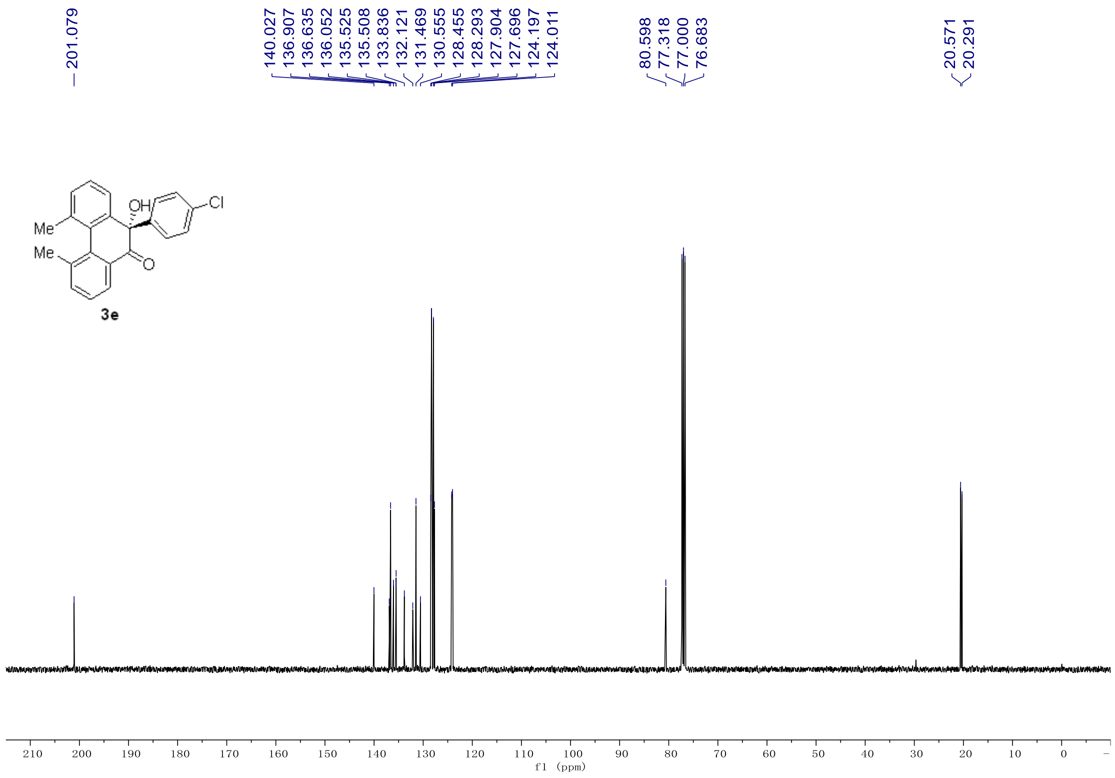
201.079
140.027
136.907
136.635
136.052
135.525
135.508
133.836
132.121
131.469
130.555
128.455
128.293
127.904
127.696
124.197
124.011
80.598
77.318
77.000
76.683
20.571
20.291
OH
Cl
Me
Me
O
3e
210 200 190 180 170 160 150 140 130 120 110 100 90 80 70 60 50 40 30 20 10 0
f1 (ppm)

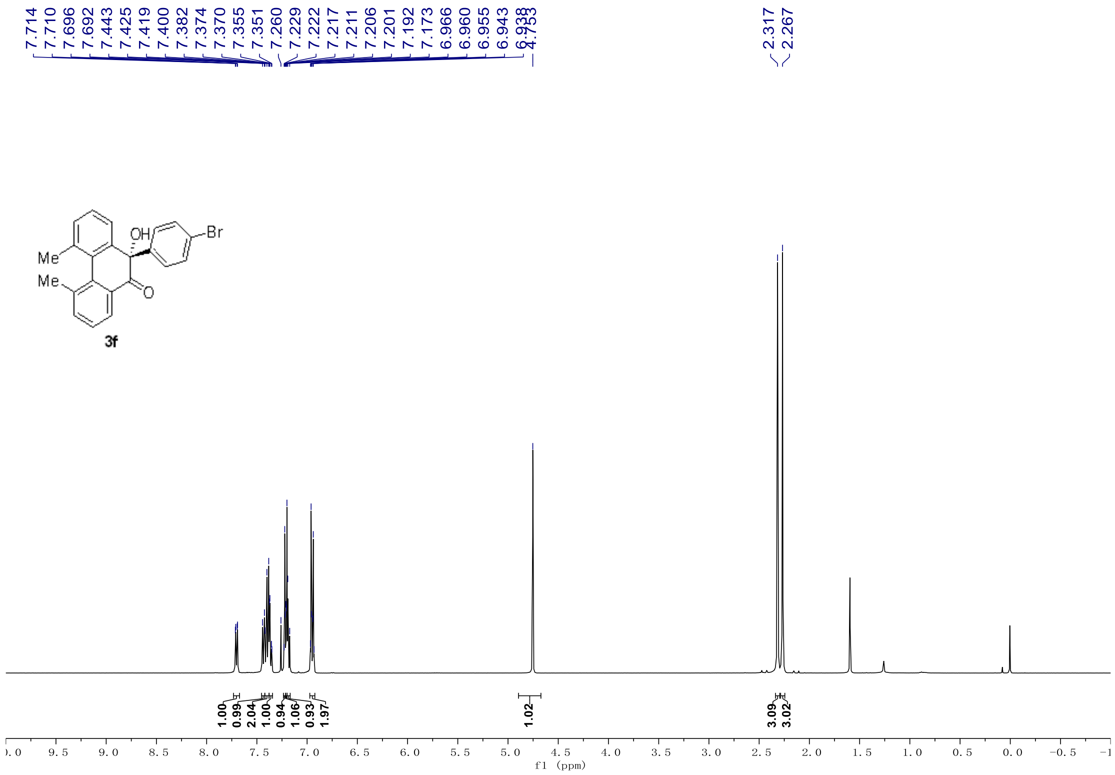
7.714
7.710
7.696
7.692
7.443
7.425
7.419
7.400
7.382
7.374
7.370
7.355
7.351
7.260
7.229
7.222
7.217
7.211
7.206
7.201
7.192
7.173
6.966
6.960
6.955
6.943
6.938
4.753
2.317
2.267
OH
Br
Me
Me
O
3f
1.00
0.99
2.04
1.00
0.94
1.06
0.93
1.97
1.02
3.09
3.02
9.5 9.0 8.5 8.0 7.5 7.0 6.5 6.0 5.5 5.0 4.5 4.0 3.5 3.0 2.5 2.0 1.5 1.0 0.5 0.0 -0.5
f1 (ppm)

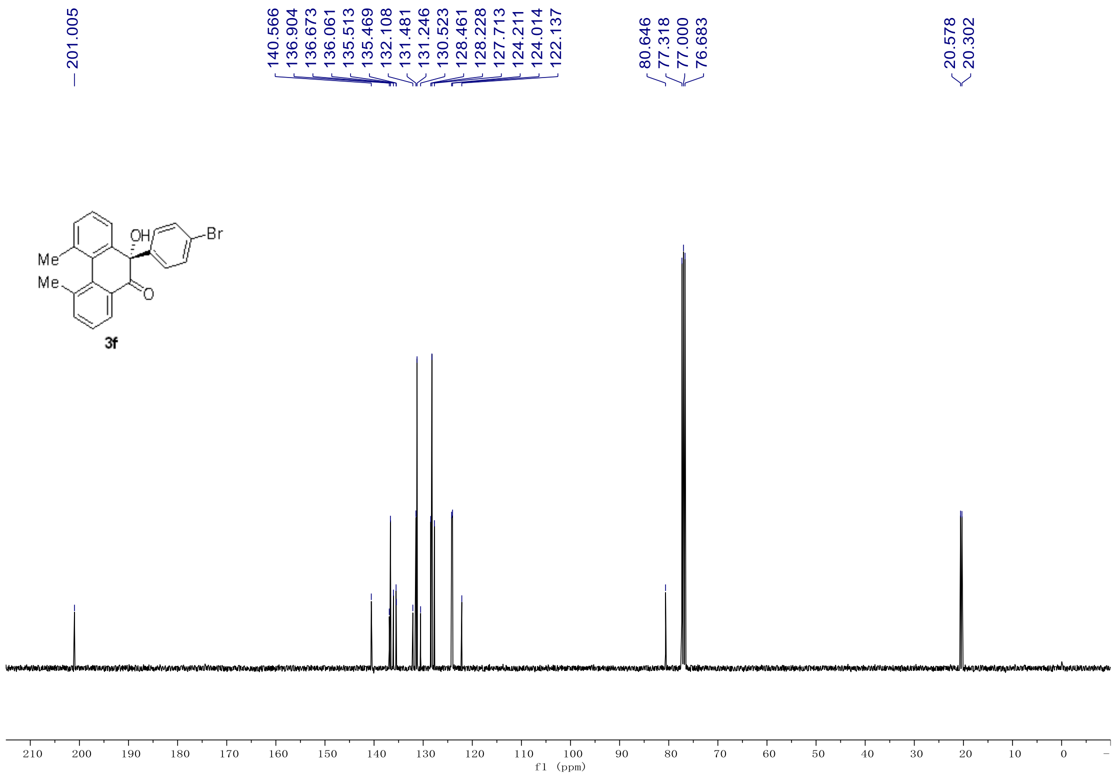
201.005
140.566
136.904
136.673
136.061
135.513
135.469
132.108
131.481
131.246
130.523
128.461
128.228
127.713
124.211
124.014
122.137
80.646
77.318
77.000
76.683
20.578
20.302
OH
Br
Me
Me
O
3f
f1 (ppm)

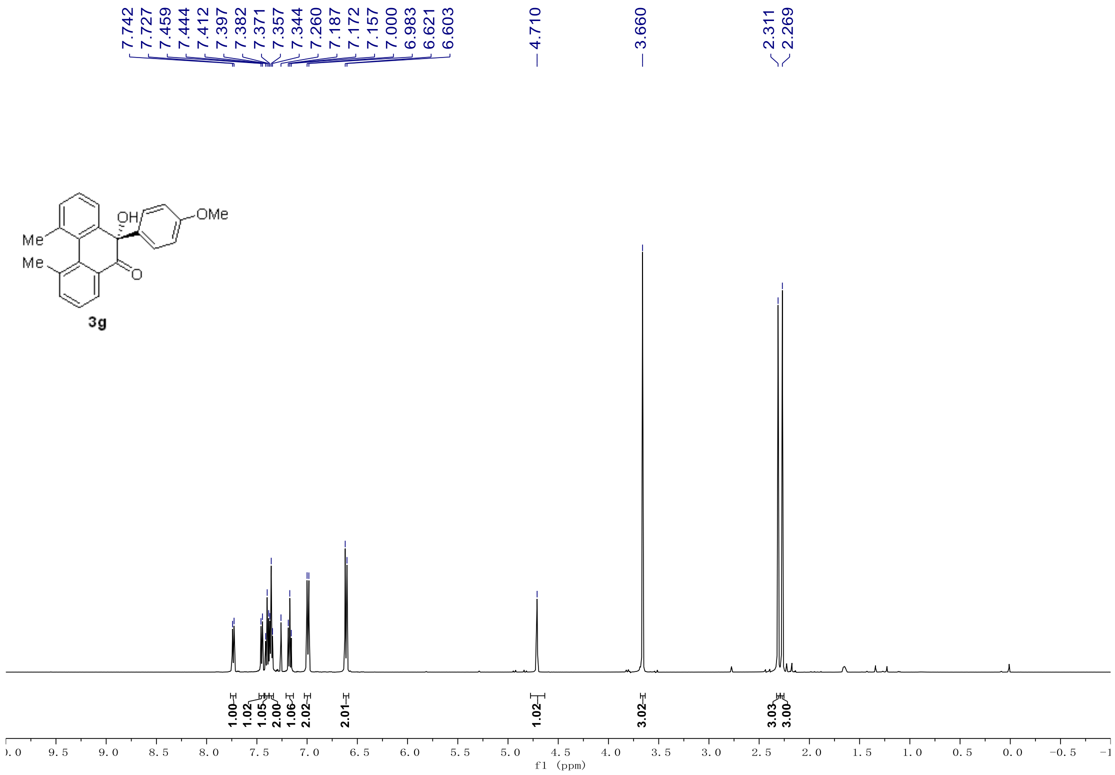
7.742
7.727
7.459
7.444
7.412
7.397
7.382
7.371
7.357
7.344
7.260
7.187
7.172
7.157
7.000
6.983
6.621
6.603
4.710
3.660
2.311
2.269
OH
OMe
Me
Me
O
3g
1.00
1.02
1.05
2.00
1.06
2.02
2.01
1.02
3.02
3.03
3.00
f1 (ppm)

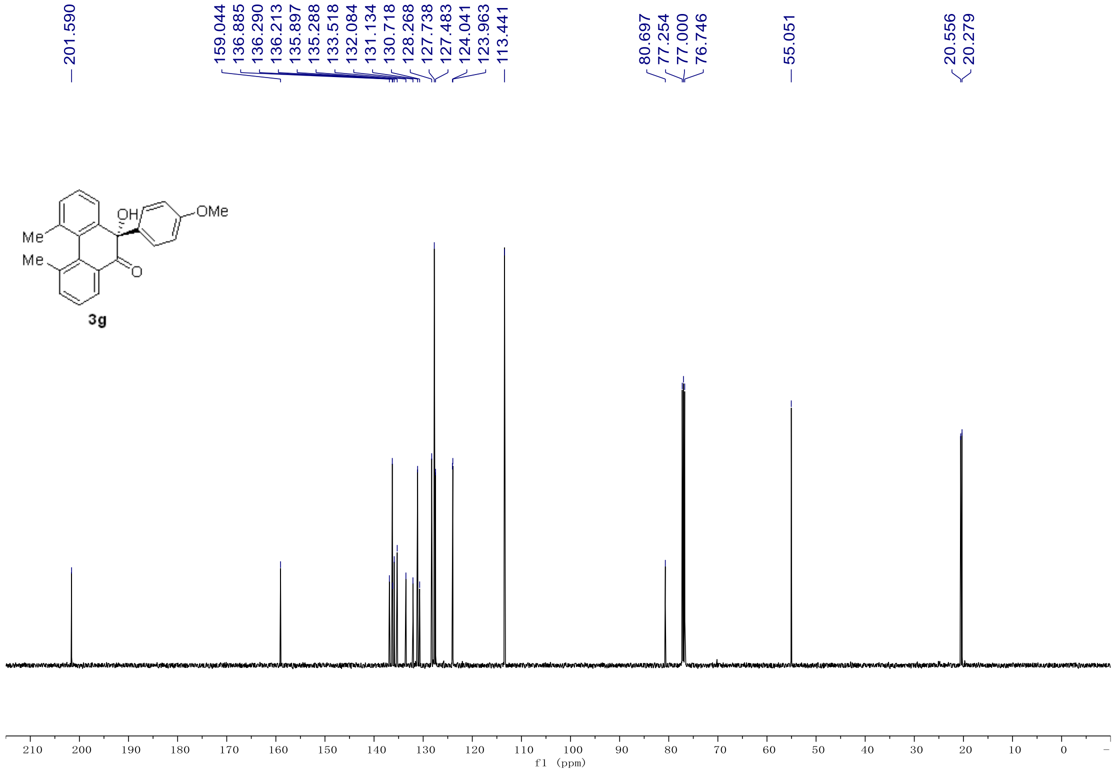
201.590
159.044
136.885
136.290
136.213
135.897
135.288
133.518
132.084
131.134
130.718
128.268
127.738
127.483
124.041
123.963
113.441
80.697
77.254
77.000
76.746
55.051
20.556
20.279
OH
OMe
Me
Me
O
3g
210 200 190 180 170 160 150 140 130 120 110 100 90 80 70 60 50 40 30 20 10 0
f1 (ppm)

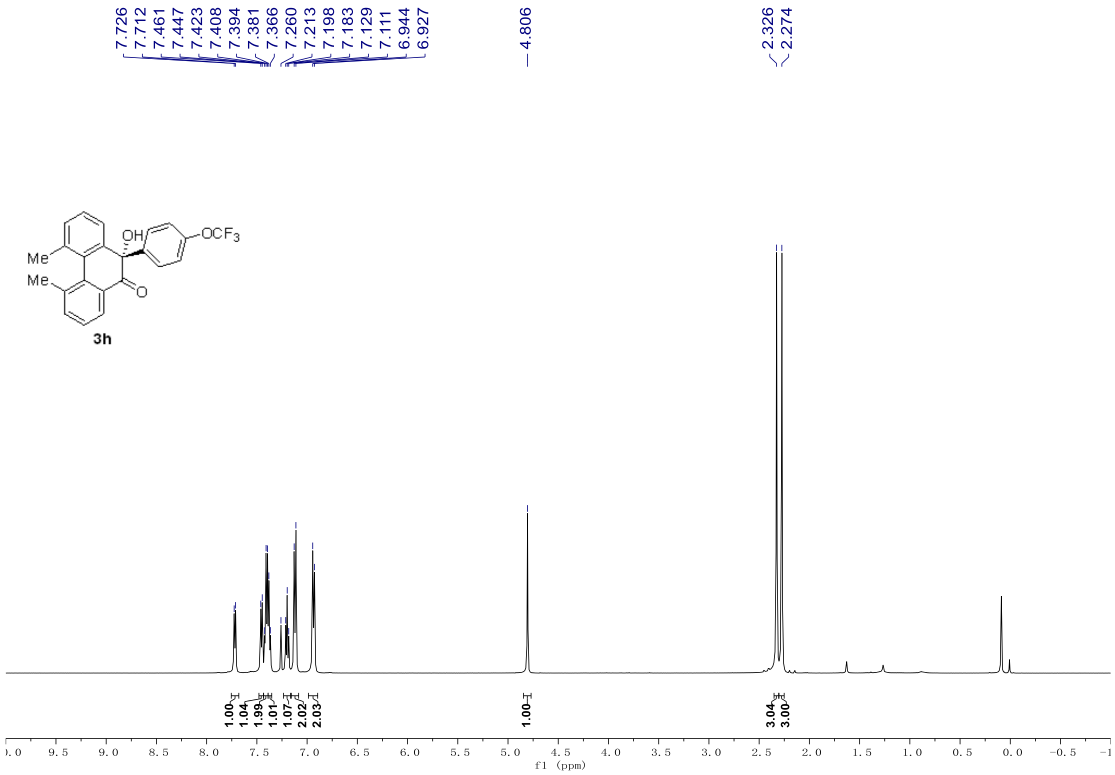
7.726
7.712
7.461
7.447
7.423
7.408
7.394
7.381
7.366
7.260
7.213
7.198
7.183
7.129
7.111
6.944
6.927
4.806
2.326
2.274
OH
OCF3
Me
Me
O
3h
1.00
1.04
1.99
1.01
1.07
2.02
2.03
1.00
3.04
3.00
9.5 9.0 8.5 8.0 7.5 7.0 6.5 6.0 5.5 5.0 4.5 4.0 3.5 3.0 2.5 2.0 1.5 1.0 0.5 0.0 −0.5
f1 (ppm)

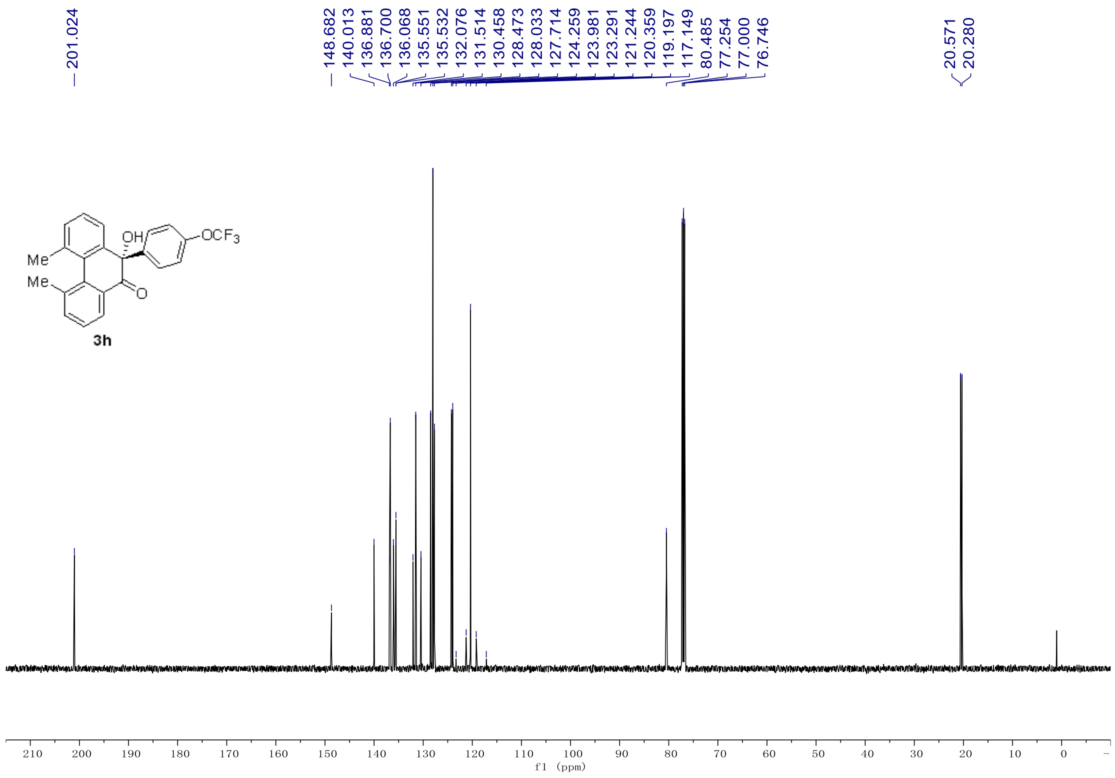
201.024
148.682
140.013
136.881
136.700
136.068
135.551
135.532
132.076
131.514
130.458
128.473
128.033
127.714
124.259
123.981
123.291
121.244
120.359
119.197
117.149
80.485
77.254
77.000
76.746
20.571
20.280
Me
Me
OH
OCF3
O
3h
f1 (ppm)

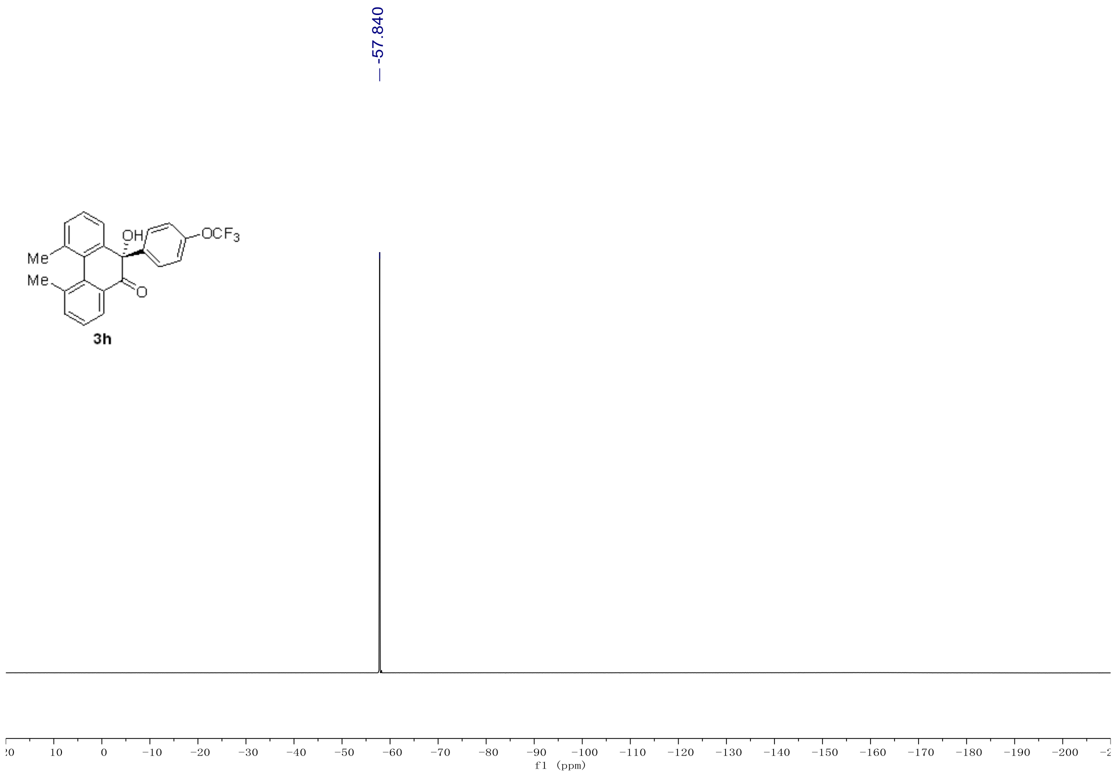
-57.840
Me
Me
OH
OCF3
O
3h
f1 (ppm)

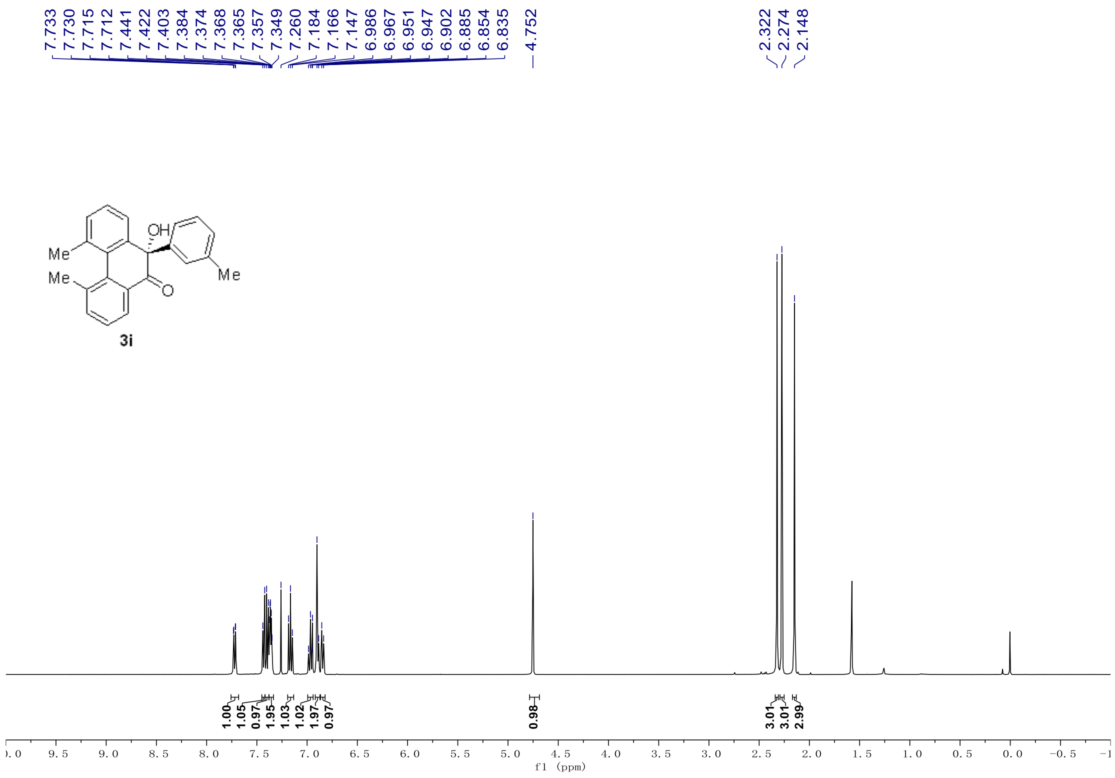
7.733 7.730 7.715 7.712 7.441 7.422 7.403 7.384 7.374 7.368 7.365 7.357 7.349 7.260 7.184 7.166 7.147 6.986 6.967 6.951 6.947 6.902 6.885 6.854 6.835
4.752
2.322 2.274 2.148
Me
Me
OH
Me
3i
1.00 1.05 0.97 1.95 1.03 1.02 1.97 0.97
0.98
3.01 3.01 2.99
f1 (ppm)

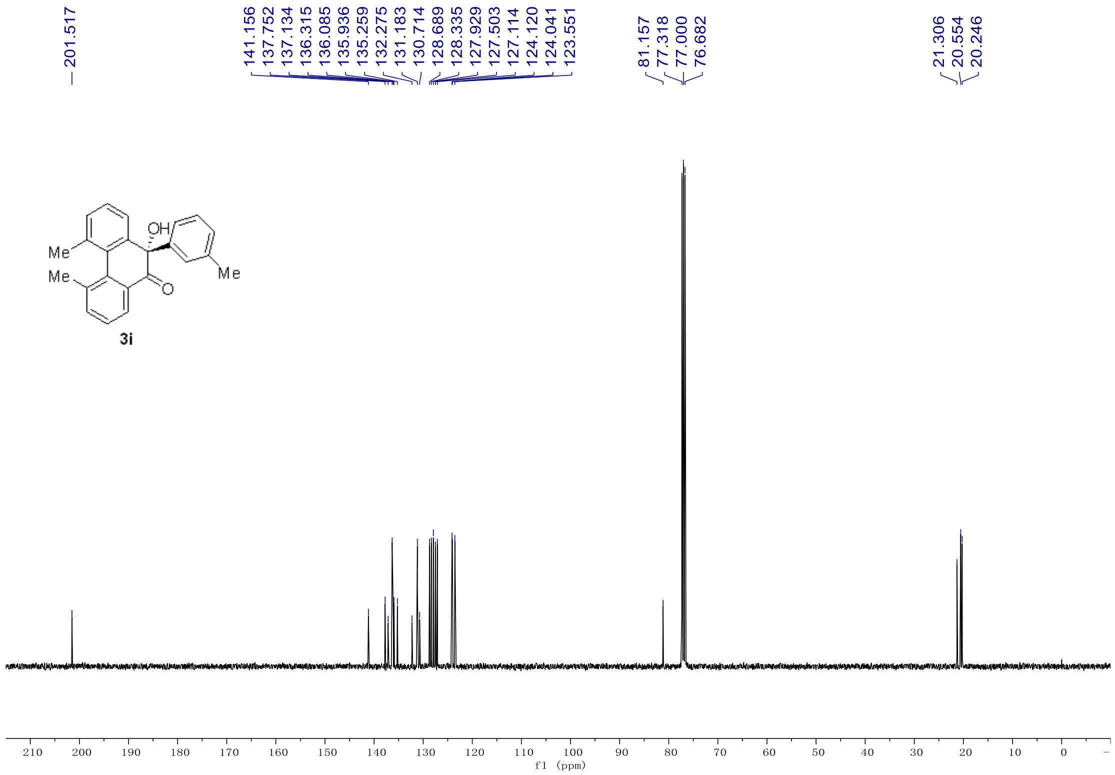
201.517
141.156 137.752 137.134 136.315 136.085 135.936 135.259 132.275 131.183 130.714 128.689 128.335 127.929 127.503 127.114 124.120 124.041 123.551
81.157 77.318 77.000 76.682
21.306 20.554 20.246
Me
Me
OH
Me
3i
f1 (ppm)

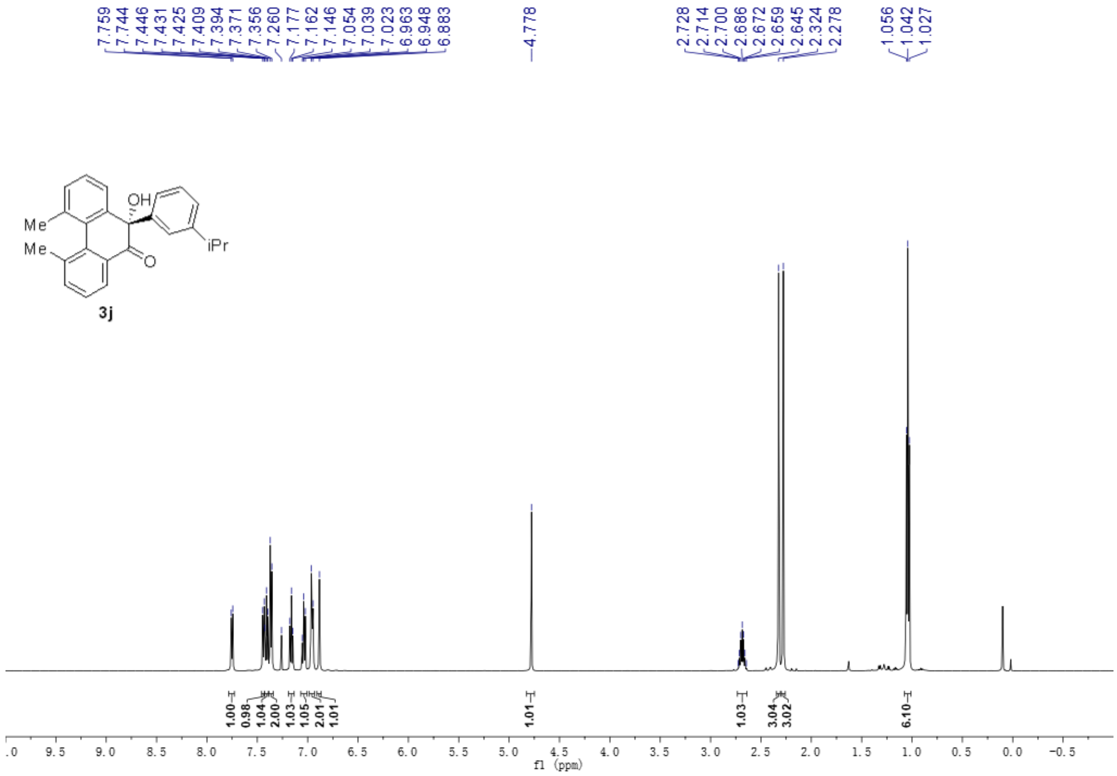
7.759 7.744 7.446 7.431 7.425 7.409 7.394 7.371 7.356 7.260 7.177 7.162 7.146 7.054 7.039 7.023 6.963 6.948 6.883
4.778
2.728 2.714 2.700 2.686 2.672 2.659 2.645 2.324 2.278
1.056 1.042 1.027
Me
Me
OH
iPr
O
3j
1.00 0.98 1.04 2.00 1.03 1.05 2.01 1.01
1.01
1.03
3.04 3.02
6.10
f1 (ppm)

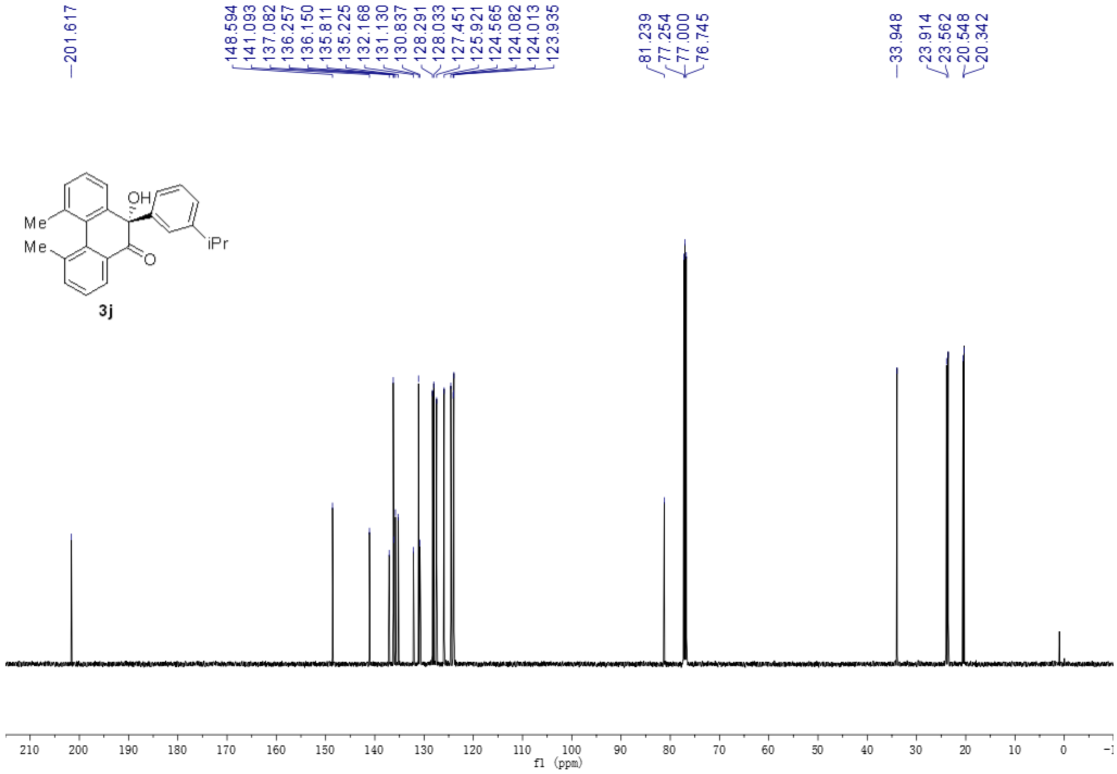
201.617
148.594 141.093 137.082 136.257 136.150 135.811 135.225 132.168 131.130 130.837 128.291 128.033 127.451 125.921 124.565 124.082 124.013 123.935
81.239 77.254 77.000 76.745
33.948 23.914 23.562 20.548 20.342
Me
Me
OH
iPr
O
3j
f1 (ppm)

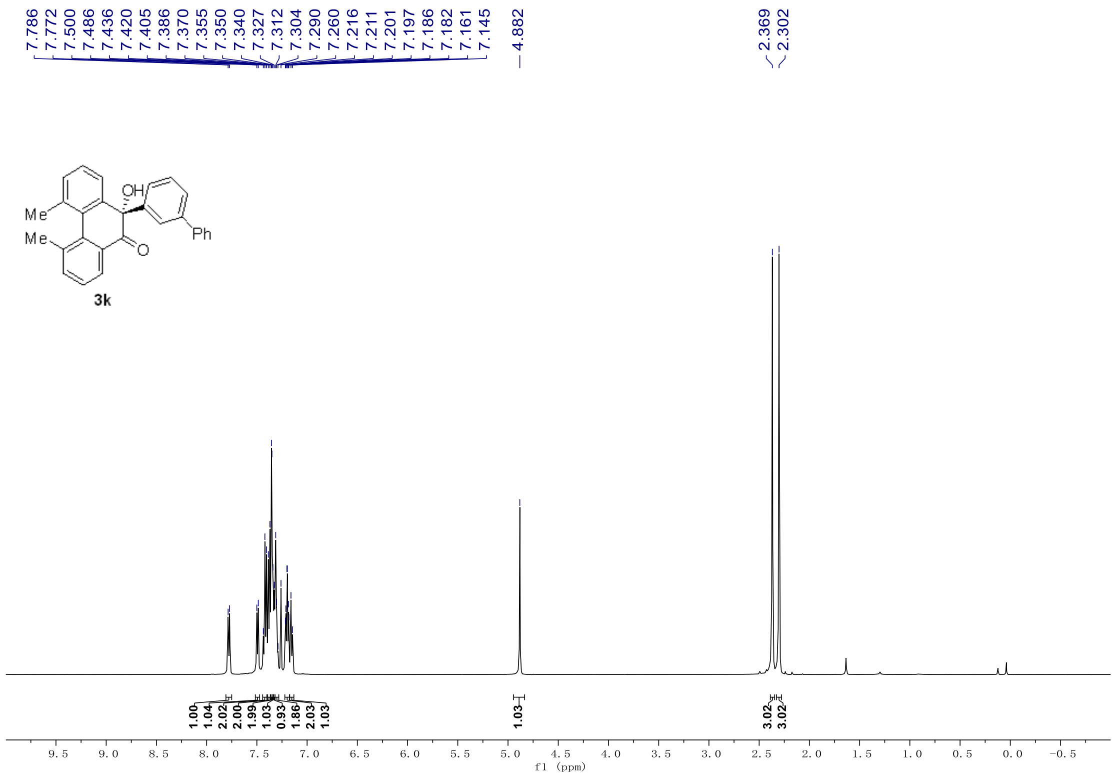

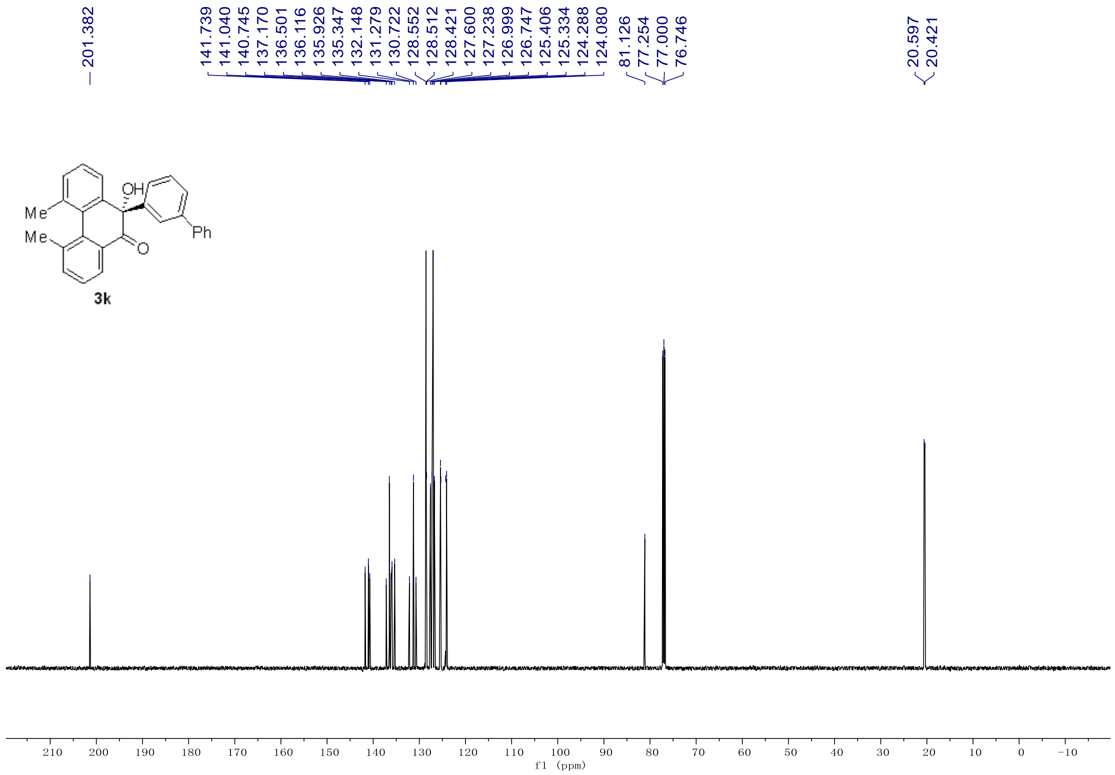

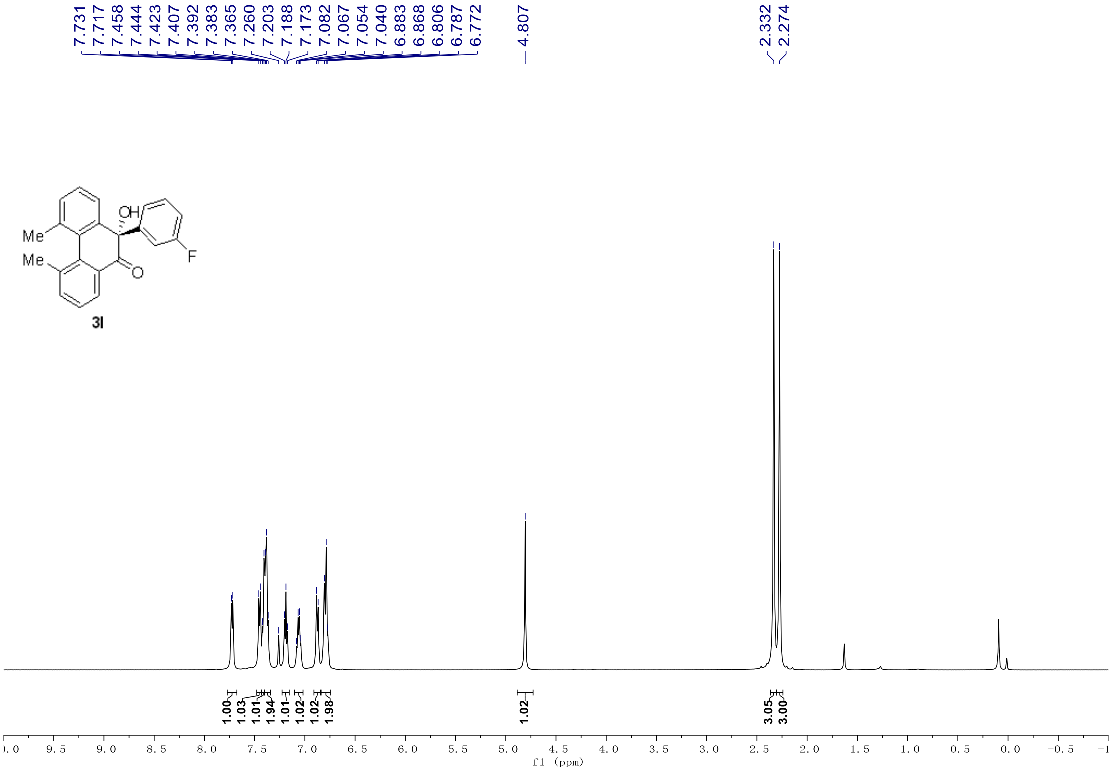
7.731
7.717
7.458
7.444
7.423
7.407
7.392
7.383
7.365
7.260
7.203
7.188
7.173
7.082
7.067
7.054
7.040
6.883
6.868
6.806
6.787
6.772
4.807
2.332
2.274
Me
Me
OH
F
O
3l
1.00
1.03
1.01
1.94
1.01
1.02
1.02
1.98
1.02
3.05
3.00
f1 (ppm)

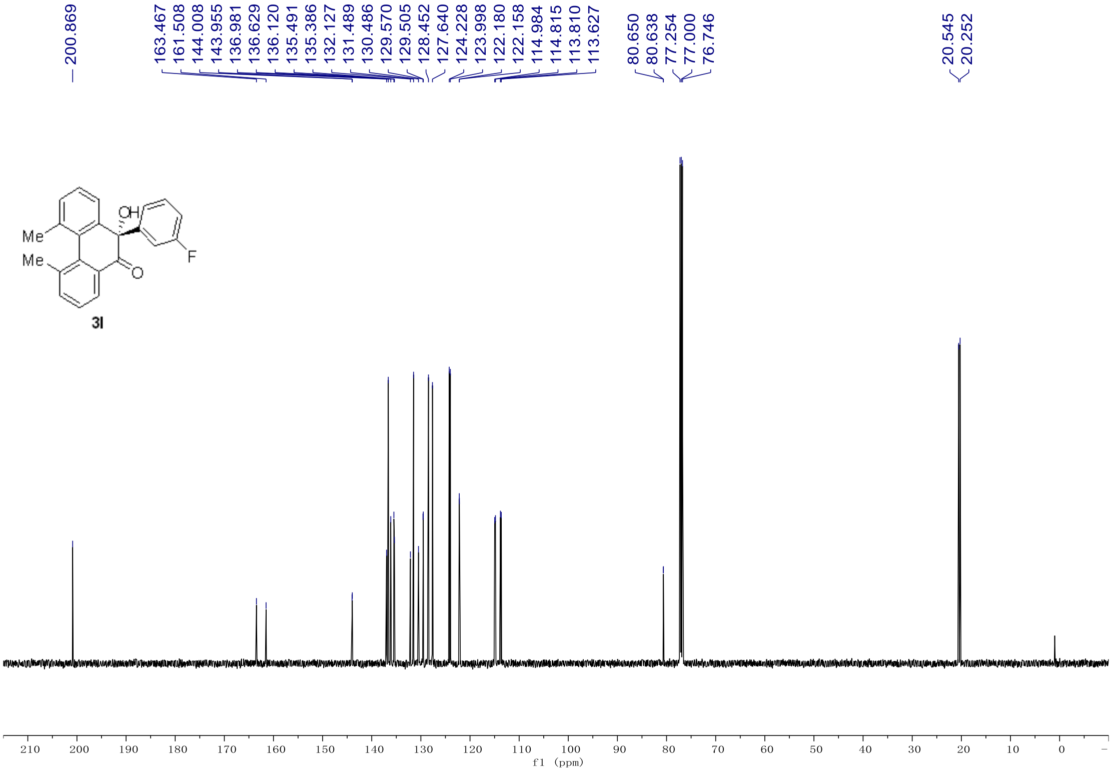
200.869
163.467
161.508
144.008
143.955
136.981
136.629
136.120
135.491
135.386
132.127
131.489
130.486
129.570
129.505
128.452
127.640
124.228
123.998
122.180
122.158
114.984
114.815
113.810
113.627
80.650
80.638
77.254
77.000
76.746
20.545
20.252
Me
Me
OH
F
O
3l
f1 (ppm)

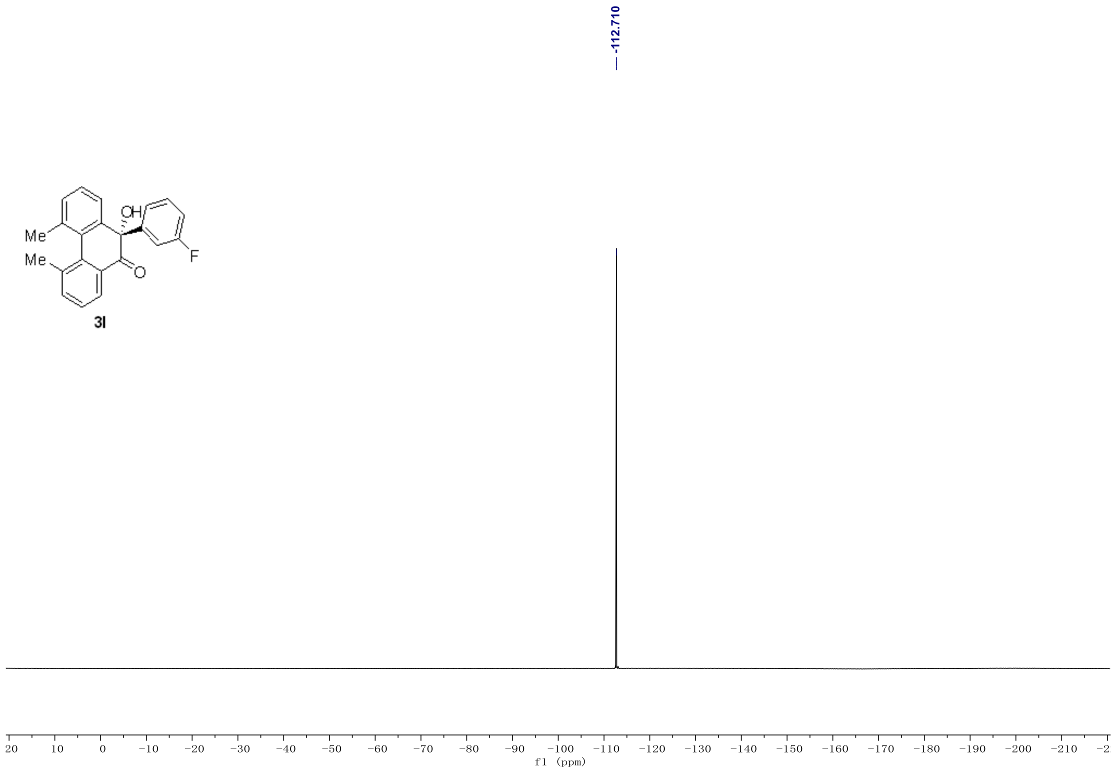
-112.710
Me
Me
OH
F
O
3l
20 10 0 −10 −20 −30 −40 −50 −60 −70 −80 −90 −100 −110 −120 −130 −140 −150 −160 −170 −180 −190 −200 −210
f1 (ppm)

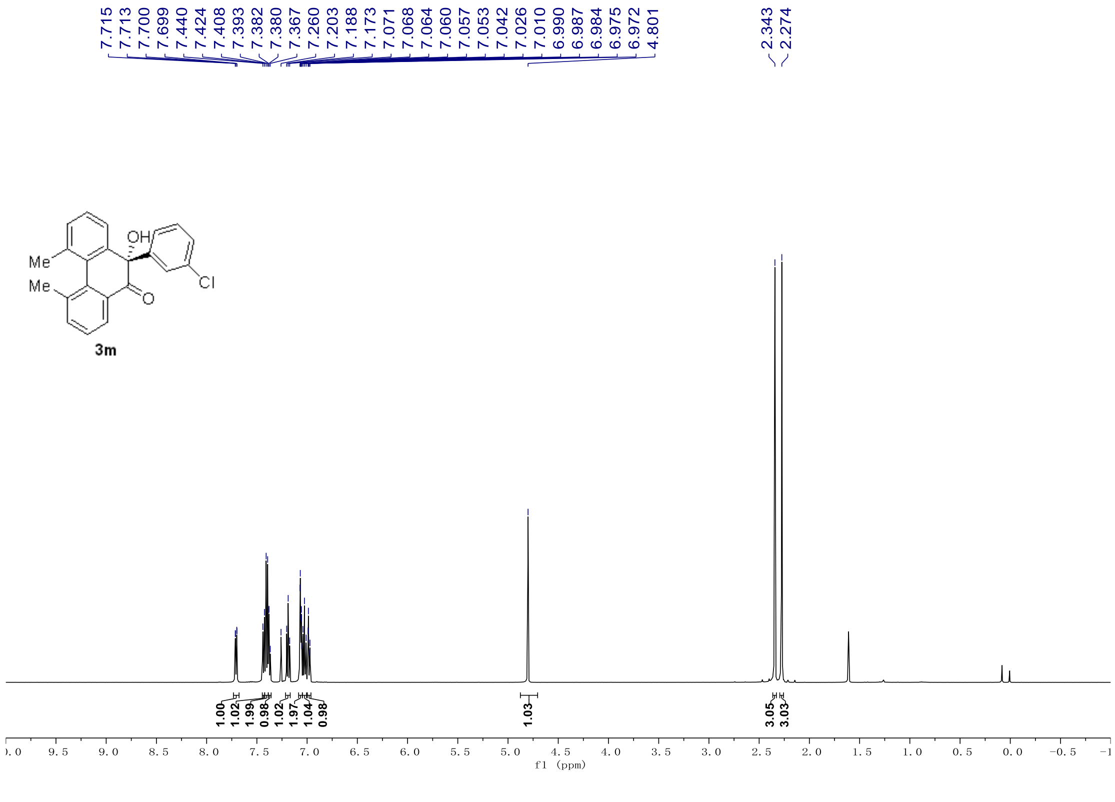
7.715
7.713
7.700
7.699
7.440
7.424
7.408
7.393
7.382
7.380
7.367
7.260
7.203
7.188
7.173
7.071
7.068
7.064
7.060
7.057
7.053
7.042
7.026
7.010
6.990
6.987
6.984
6.975
6.972
4.801
2.343
2.274
Me
Me
OH
Cl
O
3m
1.00
1.02
1.99
0.98
1.02
1.97
1.04
0.98
1.03
3.05
3.03
9.5 9.0 8.5 8.0 7.5 7.0 6.5 6.0 5.5 5.0 4.5 4.0 3.5 3.0 2.5 2.0 1.5 1.0 0.5 0.0 −0.5
f1 (ppm)

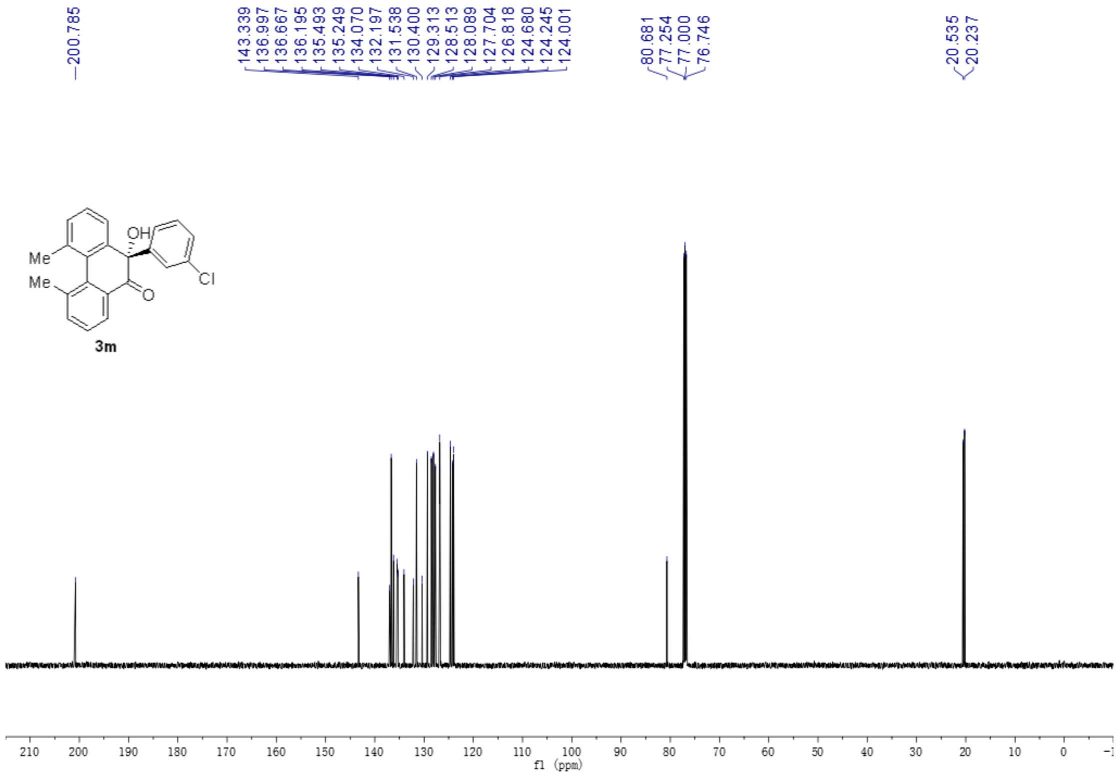
200.785
143.339
136.997
136.667
136.195
135.493
135.249
134.070
132.197
131.538
130.400
129.313
128.513
128.089
127.704
126.818
124.680
124.245
124.001
80.681
77.254
77.000
76.746
20.535
20.237
Me
Me
OH
Cl
O
3m
f1 (ppm)

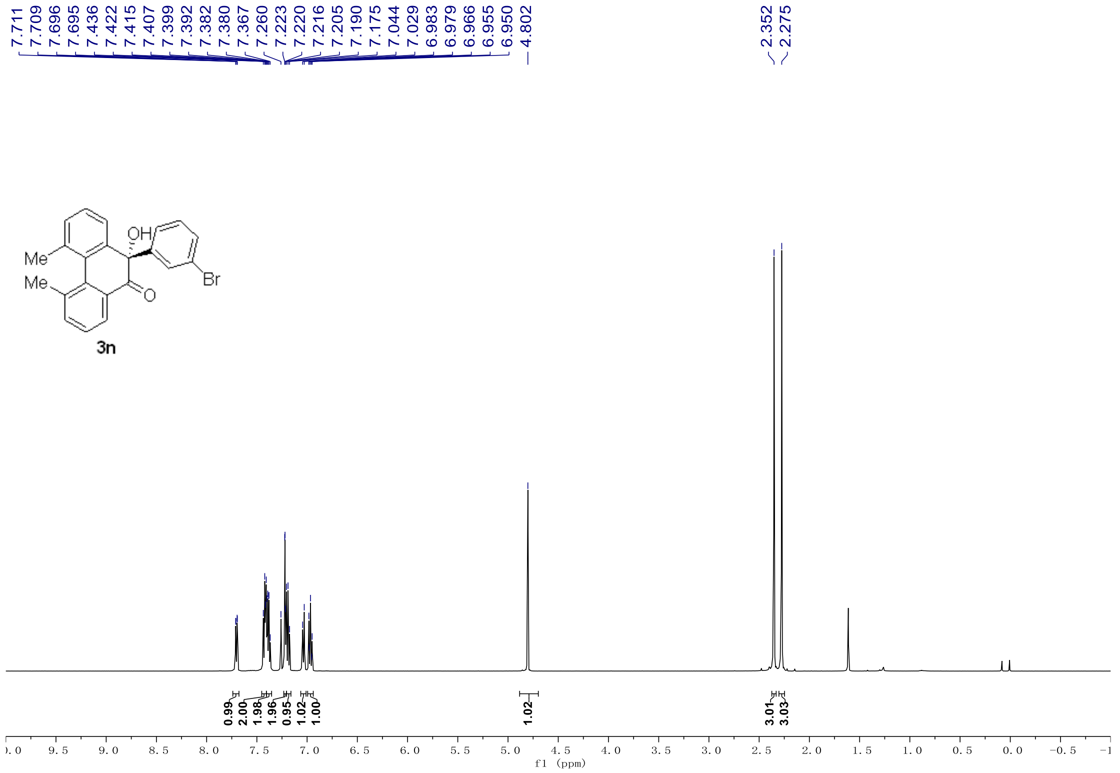
7.711
7.709
7.696
7.695
7.436
7.422
7.415
7.407
7.399
7.392
7.382
7.380
7.367
7.260
7.223
7.220
7.216
7.205
7.190
7.175
7.044
7.029
6.983
6.979
6.966
6.955
6.950
4.802
2.352
2.275
Me
Me
OH
Br
O
3n
0.99
2.00
1.98
1.96
0.95
1.02
1.00
1.02
3.01
3.03
f1 (ppm)

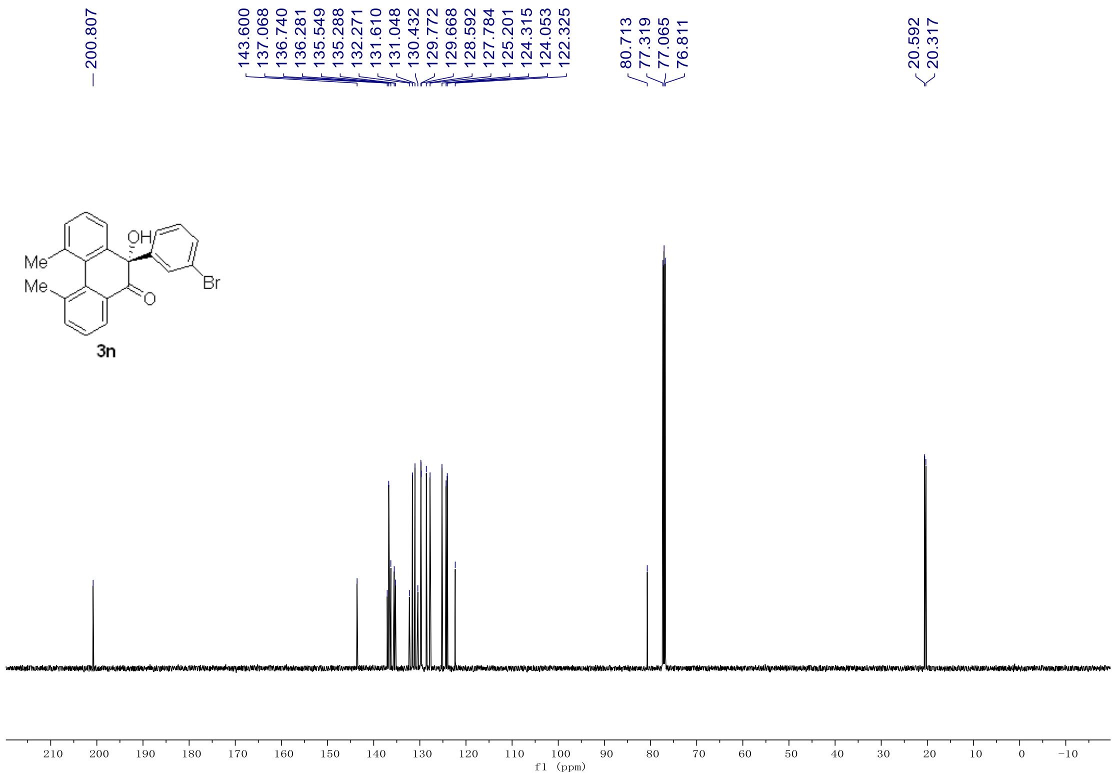
200.807
143.600
137.068
136.740
136.281
135.549
135.288
132.271
131.610
131.048
130.432
129.772
129.668
128.592
127.784
125.201
124.315
124.053
122.325
80.713
77.319
77.065
76.811
20.592
20.317
OH
Me
Me
Br
O
3n
f1 (ppm)

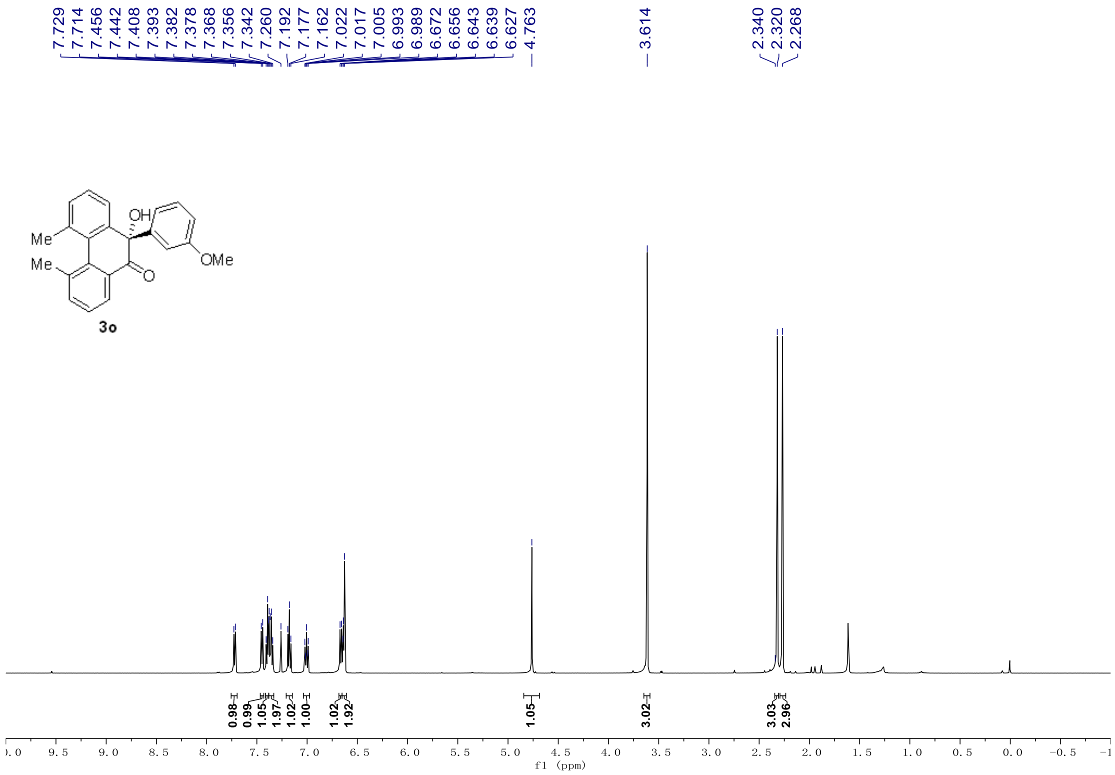
7.729
7.714
7.456
7.442
7.408
7.393
7.382
7.378
7.368
7.356
7.342
7.260
7.192
7.177
7.162
7.022
7.017
7.005
6.993
6.989
6.672
6.656
6.643
6.639
6.627
4.763
3.614
2.340
2.320
2.268
OH
Me
Me
OMe
O
3o
0.98
0.99
1.05
1.97
1.02
1.00
1.02
1.92
1.05
3.02
3.03
2.96
f1 (ppm)

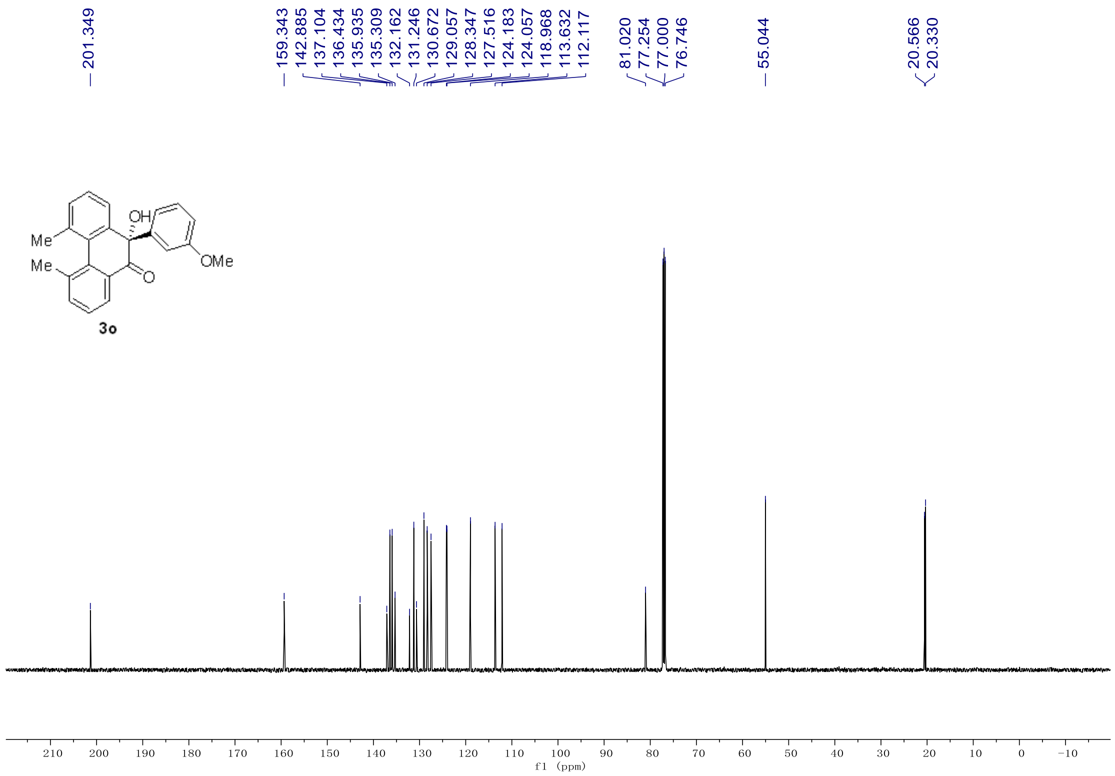

201.349
159.343
142.885
137.104
136.434
135.935
135.309
132.162
131.246
130.672
129.057
128.347
127.516
124.183
124.057
118.968
113.632
112.117
81.020
77.254
77.000
76.746
55.044
20.566
20.330
Me
Me
OH
OMe
O
3o
210 200 190 180 170 160 150 140 130 120 110 100 90 80 70 60 50 40 30 20 10 0 -10
f1 (ppm)

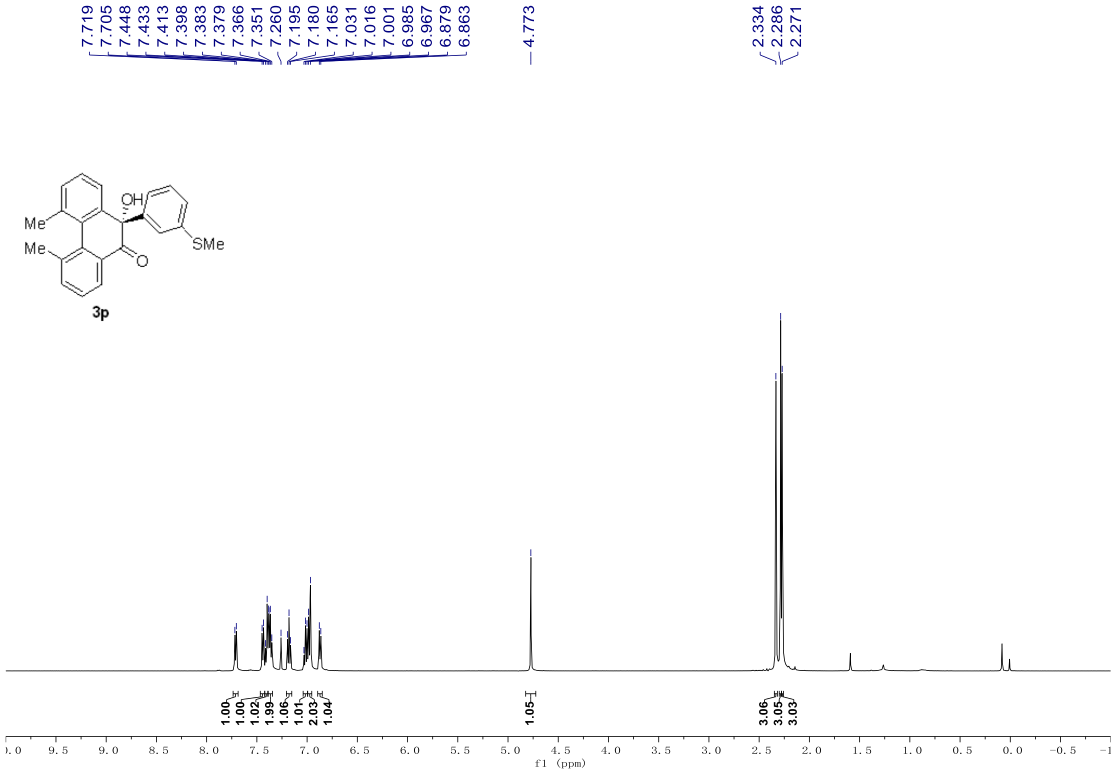

7.719
7.705
7.448
7.433
7.413
7.398
7.383
7.379
7.366
7.351
7.260
7.195
7.180
7.165
7.031
7.016
7.001
6.985
6.967
6.879
6.863
4.773
2.334
2.286
2.271
Me
Me
OH
SMe
O
3p
1.00
1.00
1.02
1.99
1.06
1.01
2.03
1.04
1.05
3.06
3.05
3.03
9.5 9.0 8.5 8.0 7.5 7.0 6.5 6.0 5.5 5.0 4.5 4.0 3.5 3.0 2.5 2.0 1.5 1.0 0.5 0.0 -0.5
f1 (ppm)

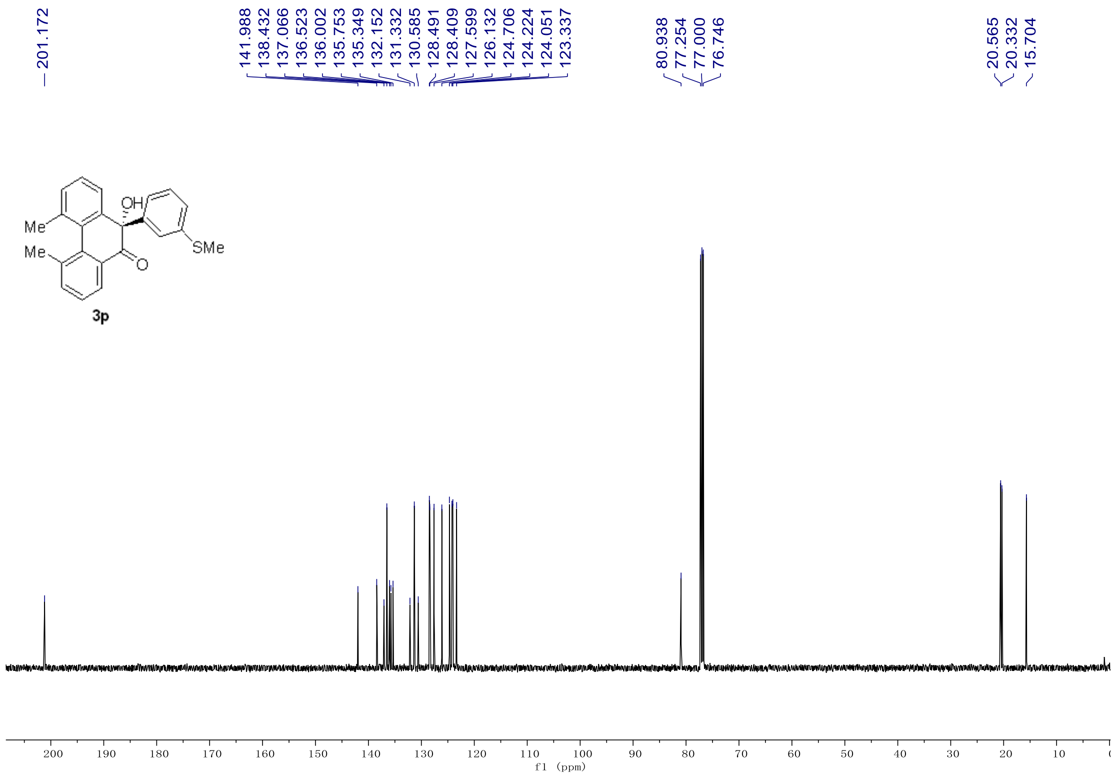
201.172
141.988 138.432 137.066 136.523 136.002 135.753 135.349 132.152 131.332 130.585 128.491 128.409 127.599 126.132 124.706 124.224 124.051 123.337
80.938 77.254 77.000 76.746
20.565 20.332 15.704
Me
Me
OH
SMe
O
3p
f1 (ppm)

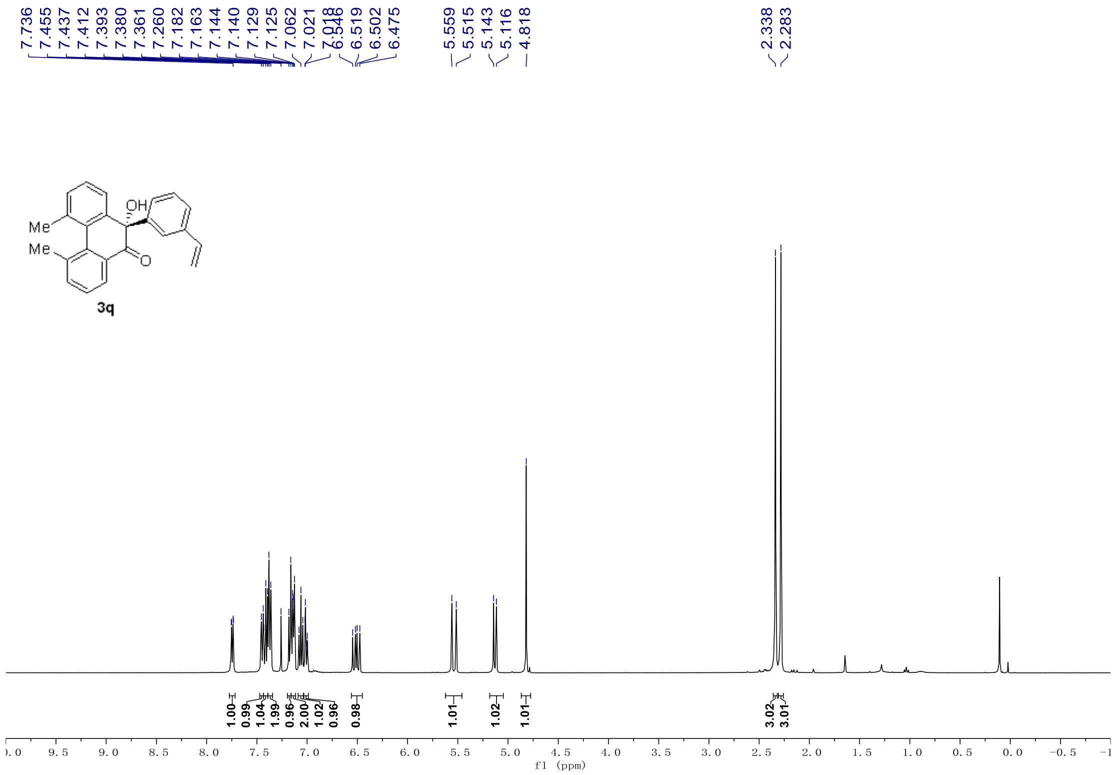
7.736 7.455 7.437 7.412 7.393 7.380 7.361 7.260 7.182 7.163 7.144 7.140 7.129 7.125 7.062 7.021 7.018 6.546 6.519 6.502 6.475
5.559 5.515 5.143 5.116 4.818
2.338 2.283
Me
Me
OH
O
3q
1.00 0.99 1.04 1.99 0.96 2.00 1.02 0.96 0.98 1.01 1.02 1.01 3.02 3.01
f1 (ppm)

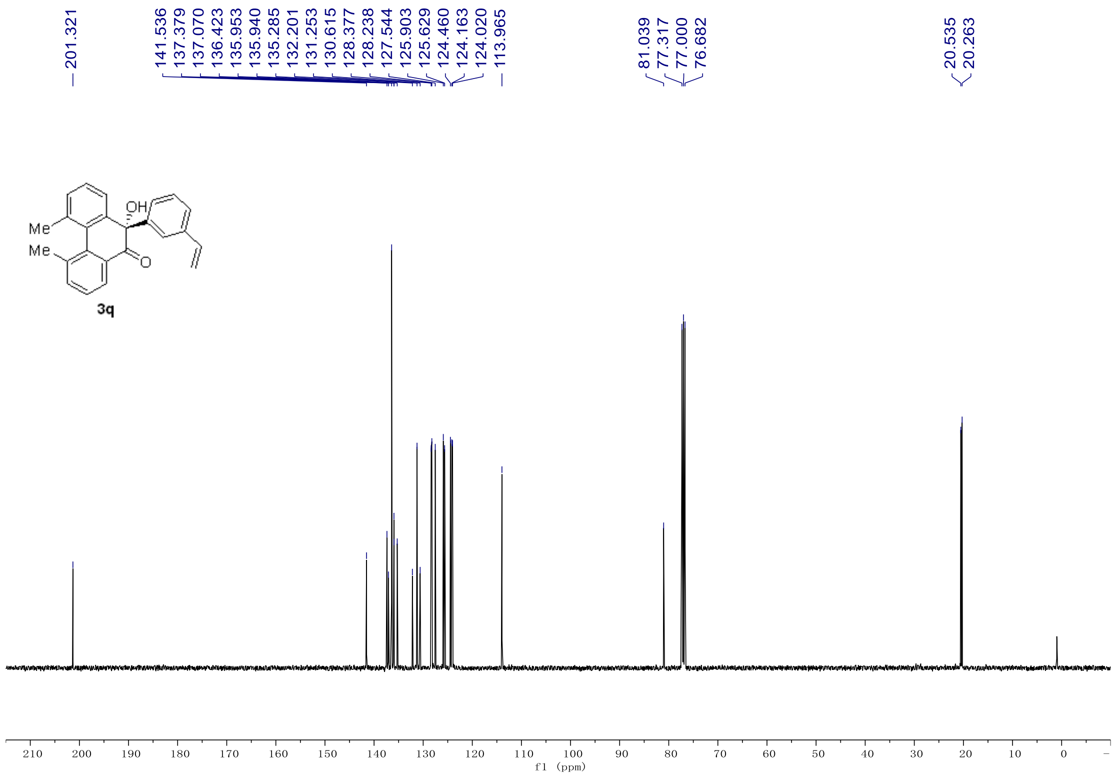
201.321
141.536
137.379
137.070
136.423
135.953
135.940
135.285
132.201
131.253
130.615
128.377
128.238
127.544
125.903
125.629
124.460
124.163
124.020
113.965
81.039
77.317
77.000
76.682
20.535
20.263
OH
Me
Me
O
3q
f1 (ppm)

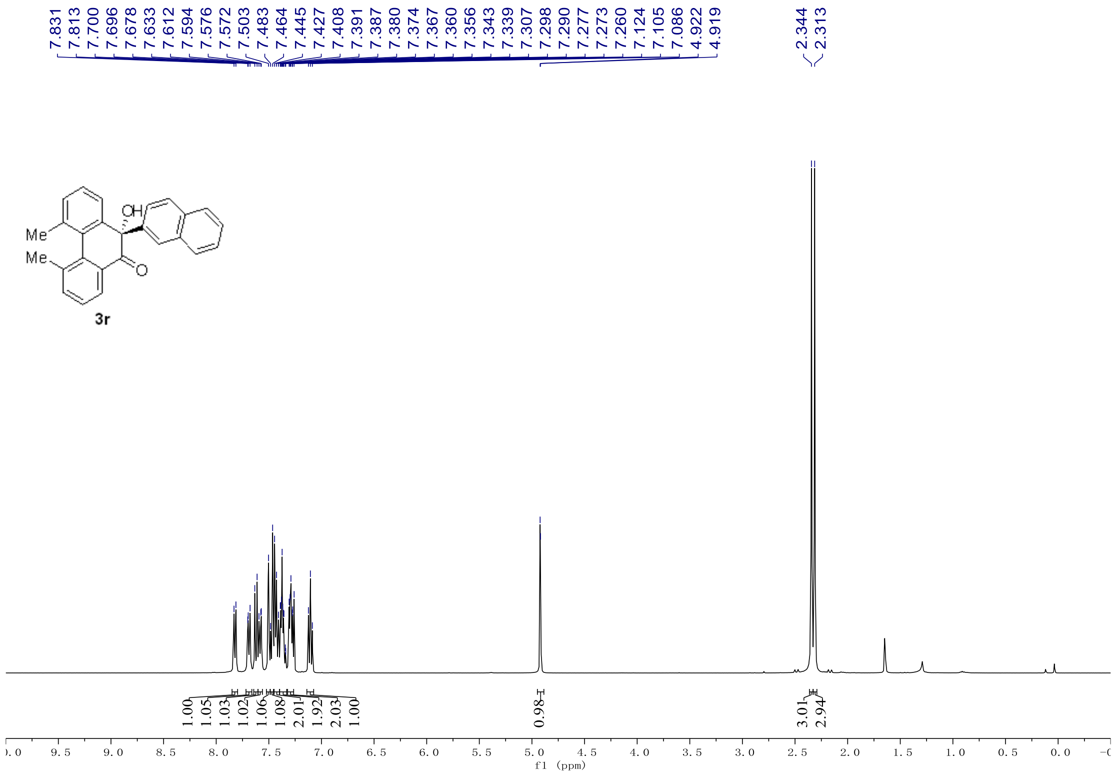
7.831
7.813
7.700
7.696
7.678
7.633
7.612
7.594
7.576
7.572
7.503
7.483
7.464
7.445
7.427
7.408
7.391
7.387
7.380
7.374
7.367
7.360
7.356
7.343
7.339
7.307
7.298
7.290
7.277
7.273
7.260
7.124
7.105
7.086
4.922
4.919
2.344
2.313
OH
Me
Me
O
3r
1.00
1.05
1.03
1.02
1.06
1.08
2.01
1.92
2.03
1.00
0.98
3.01
2.94
f1 (ppm)

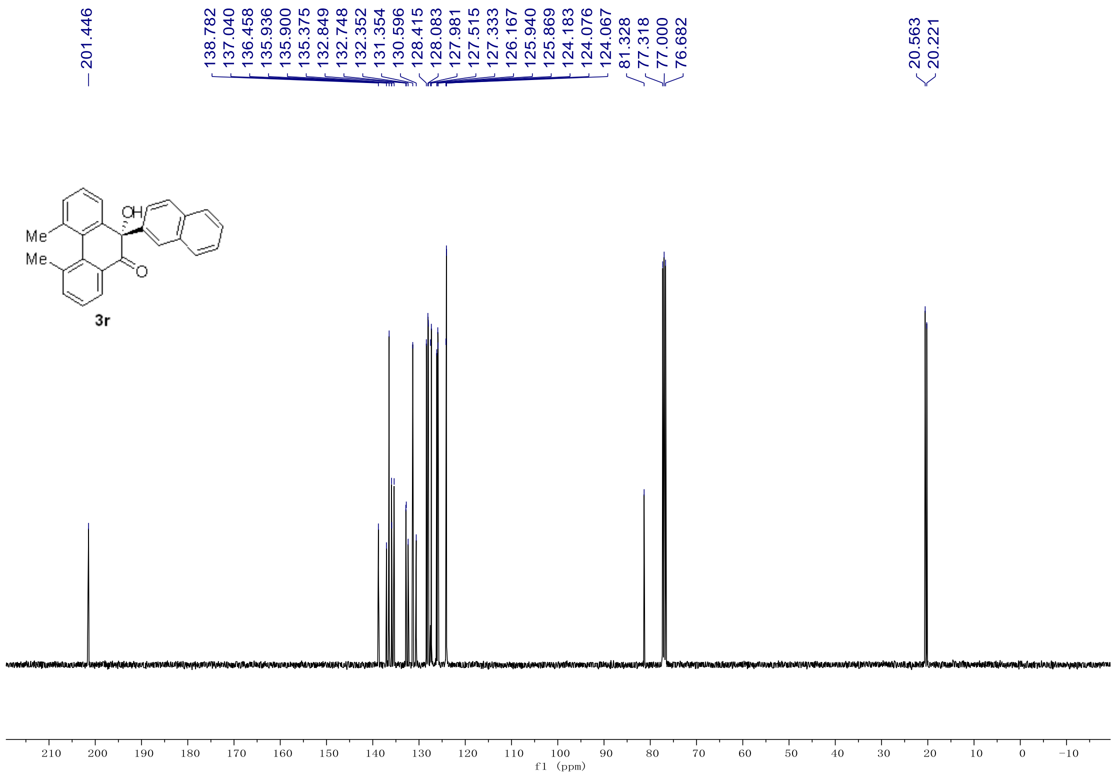
201.446
138.782
137.040
136.458
135.936
135.900
135.375
132.849
132.748
132.352
131.354
130.596
128.415
128.083
127.981
127.515
127.333
126.167
125.940
125.869
124.183
124.076
124.067
81.328
77.318
77.000
76.682
20.563
20.221
Me
Me
OH
O
3r
210 200 190 180 170 160 150 140 130 120 110 100 90 80 70 60 50 40 30 20 10 0 -10
f1 (ppm)

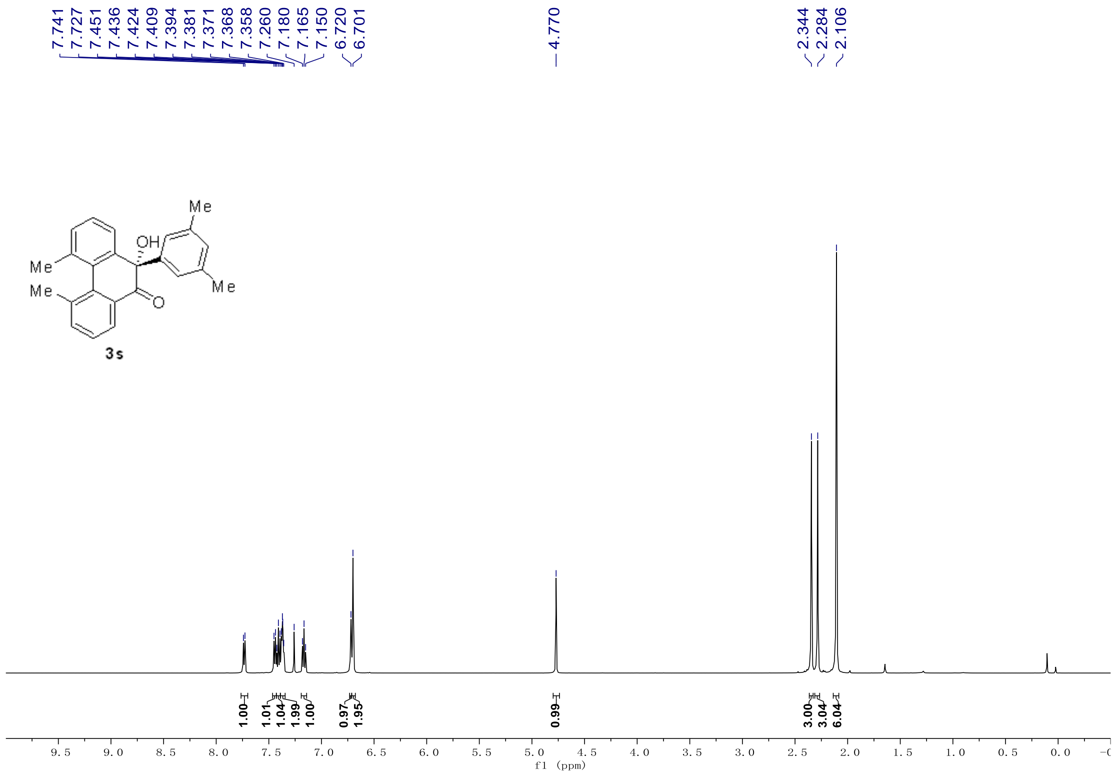
7.741
7.727
7.451
7.436
7.424
7.409
7.394
7.381
7.371
7.368
7.358
7.260
7.180
7.165
7.150
6.720
6.701
4.770
2.344
2.284
2.106
Me
Me
Me
Me
OH
O
3s
1.00
1.01
1.04
1.99
1.00
0.97
1.95
0.99
3.00
3.04
6.04
9.5 9.0 8.5 8.0 7.5 7.0 6.5 6.0 5.5 5.0 4.5 4.0 3.5 3.0 2.5 2.0 1.5 1.0 0.5 0.0
f1 (ppm)

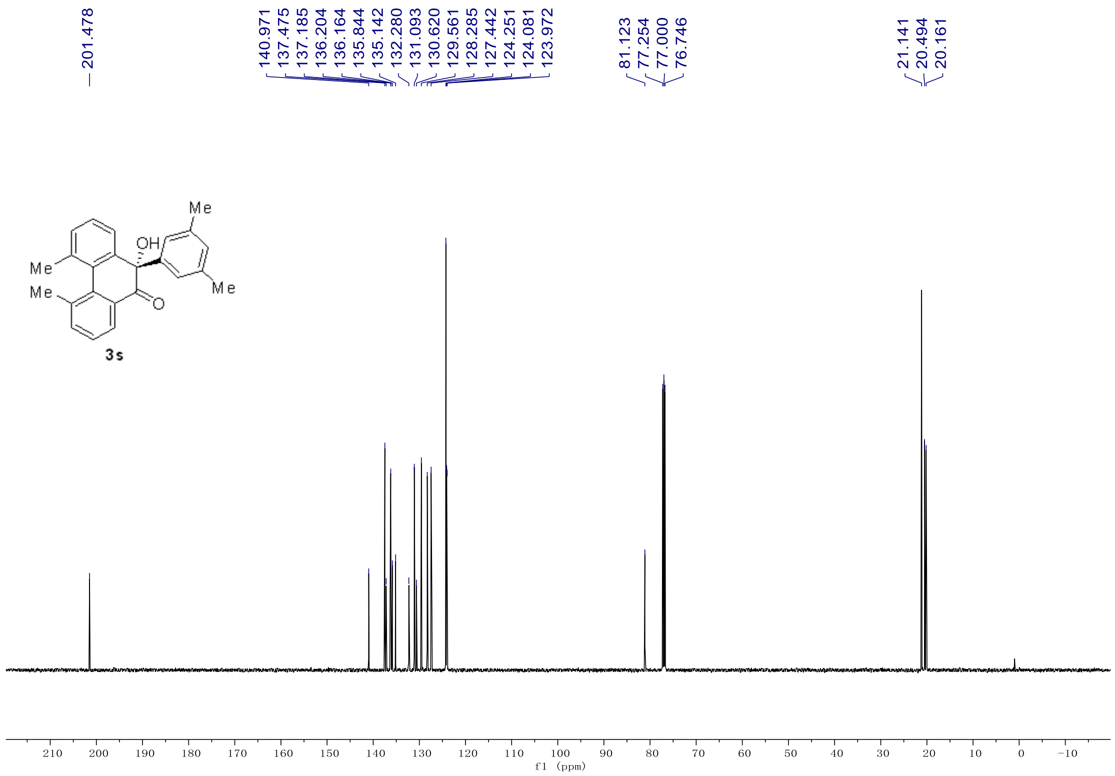
201.478
140.971
137.475
137.185
136.204
136.164
135.844
135.142
132.280
131.093
130.620
129.561
128.285
127.442
124.251
124.081
123.972
81.123
77.254
77.000
76.746
21.141
20.494
20.161
OH
Me
Me
Me
Me
3s
f1 (ppm)

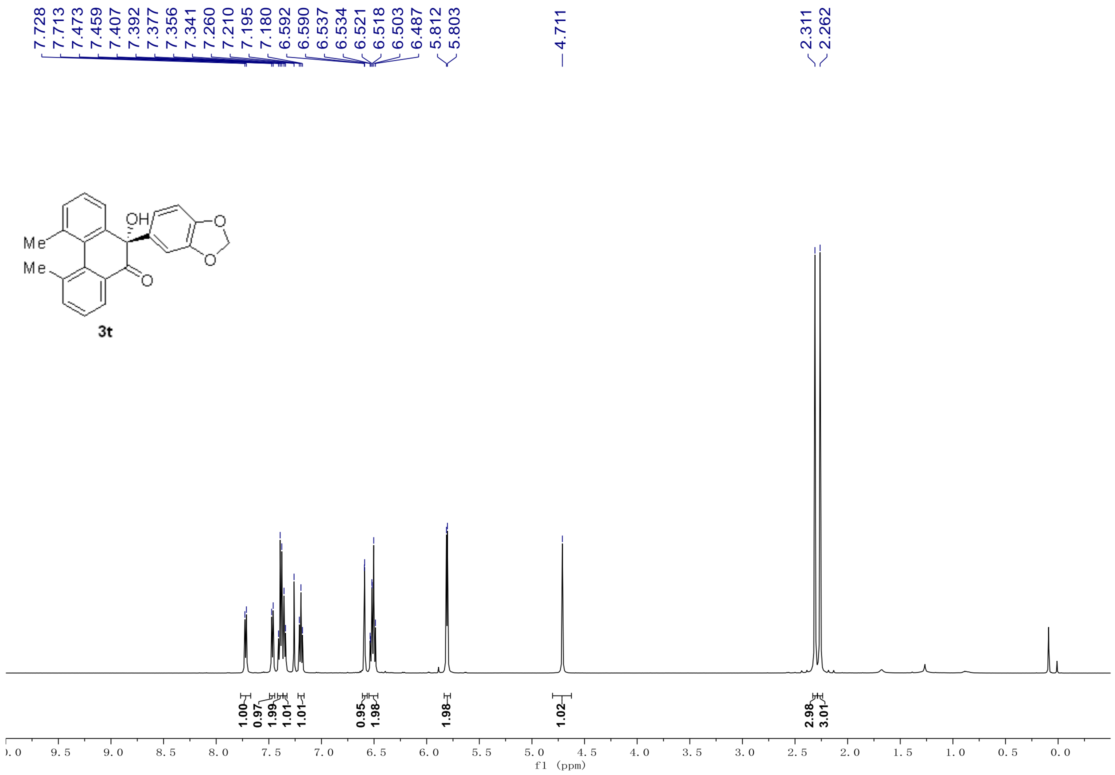
7.728
7.713
7.473
7.459
7.407
7.392
7.377
7.356
7.341
7.260
7.210
7.195
7.180
6.592
6.590
6.537
6.534
6.521
6.518
6.503
6.487
5.812
5.803
4.711
2.311
2.262
OH
Me
Me
3t
1.00
0.97
1.99
1.01
1.01
0.95
1.98
1.98
1.02
2.98
3.01
f1 (ppm)

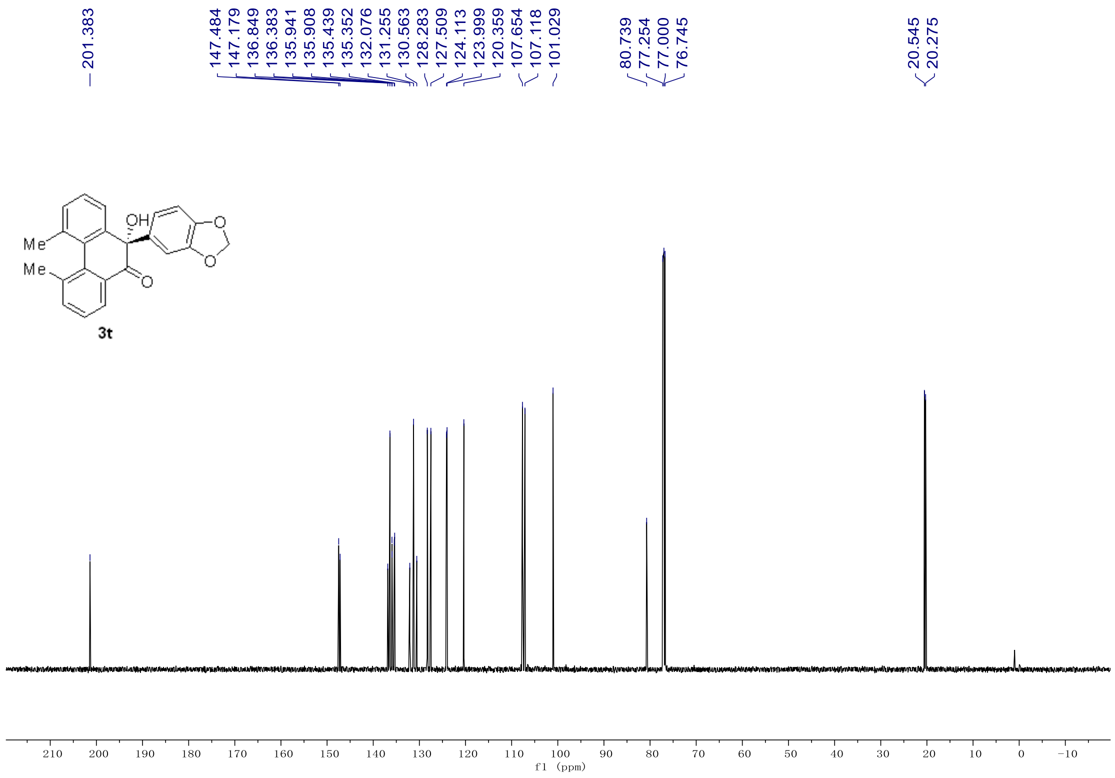
201.383
147.484
147.179
136.849
136.383
135.941
135.908
135.439
135.352
132.076
131.255
130.563
128.283
127.509
124.113
123.999
120.359
107.654
107.118
101.029
80.739
77.254
77.000
76.745
20.545
20.275
OH
Me
Me
O
3t
f1 (ppm)

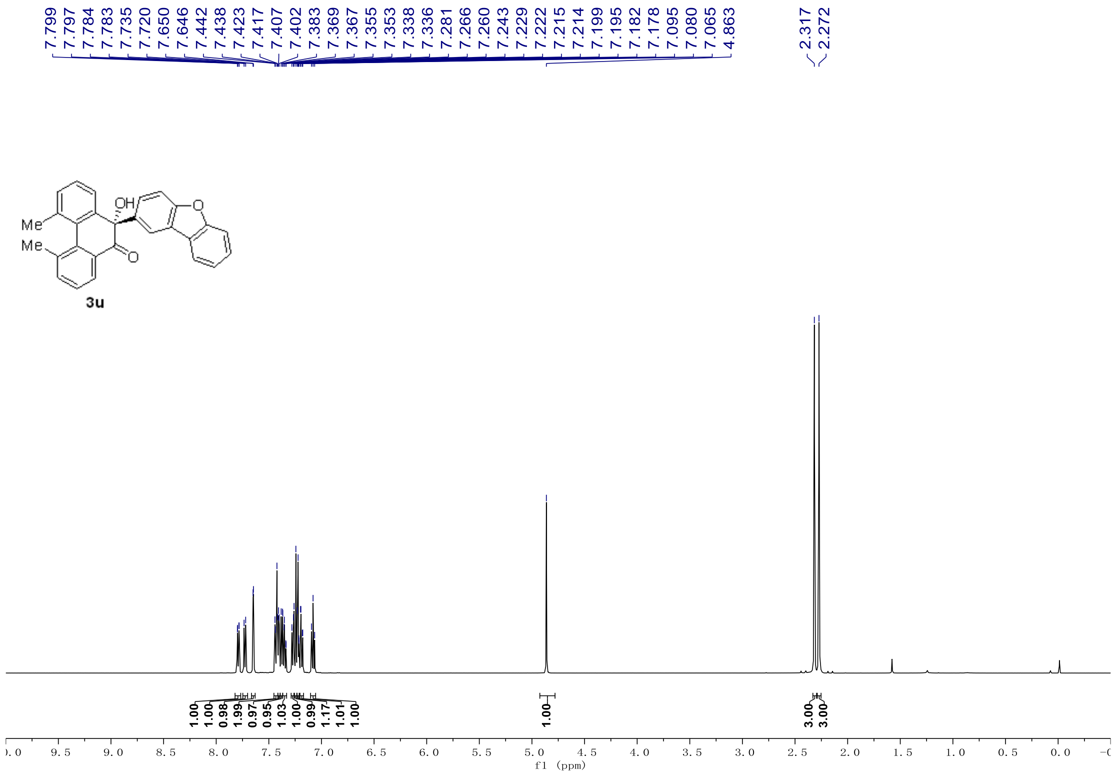
7.799
7.797
7.784
7.783
7.735
7.720
7.650
7.646
7.442
7.438
7.423
7.417
7.407
7.402
7.383
7.369
7.367
7.355
7.353
7.338
7.336
7.281
7.266
7.260
7.243
7.229
7.222
7.215
7.214
7.199
7.195
7.182
7.178
7.095
7.080
7.065
4.863
2.317
2.272
OH
Me
Me
O
3u
1.00
1.00
0.98
1.99
0.97
0.95
1.03
1.00
0.99
1.17
1.01
1.00
1.00
3.00
3.00
f1 (ppm)

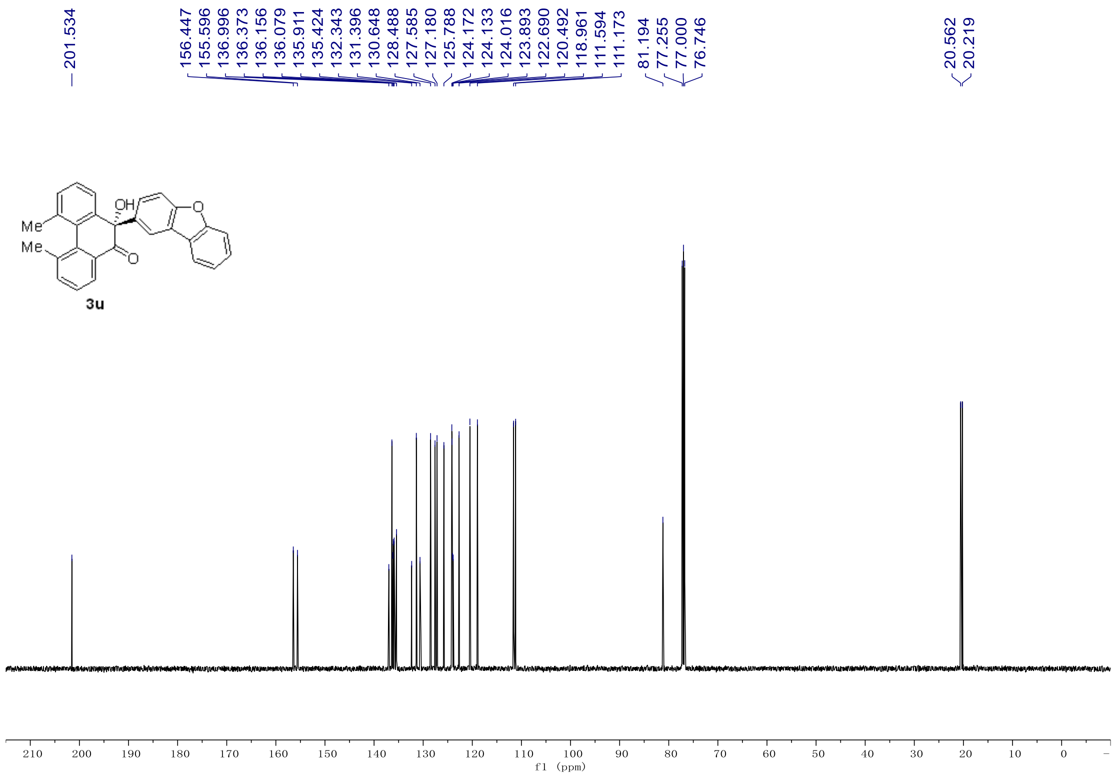
201.534
156.447
155.596
136.996
136.373
136.156
136.079
135.911
135.424
132.343
131.396
130.648
128.488
127.585
127.180
125.788
124.172
124.133
124.016
123.893
122.690
120.492
118.961
111.594
111.173
81.194
77.255
77.000
76.746
20.562
20.219
OH
Me
Me
O
O
3u
f1 (ppm)

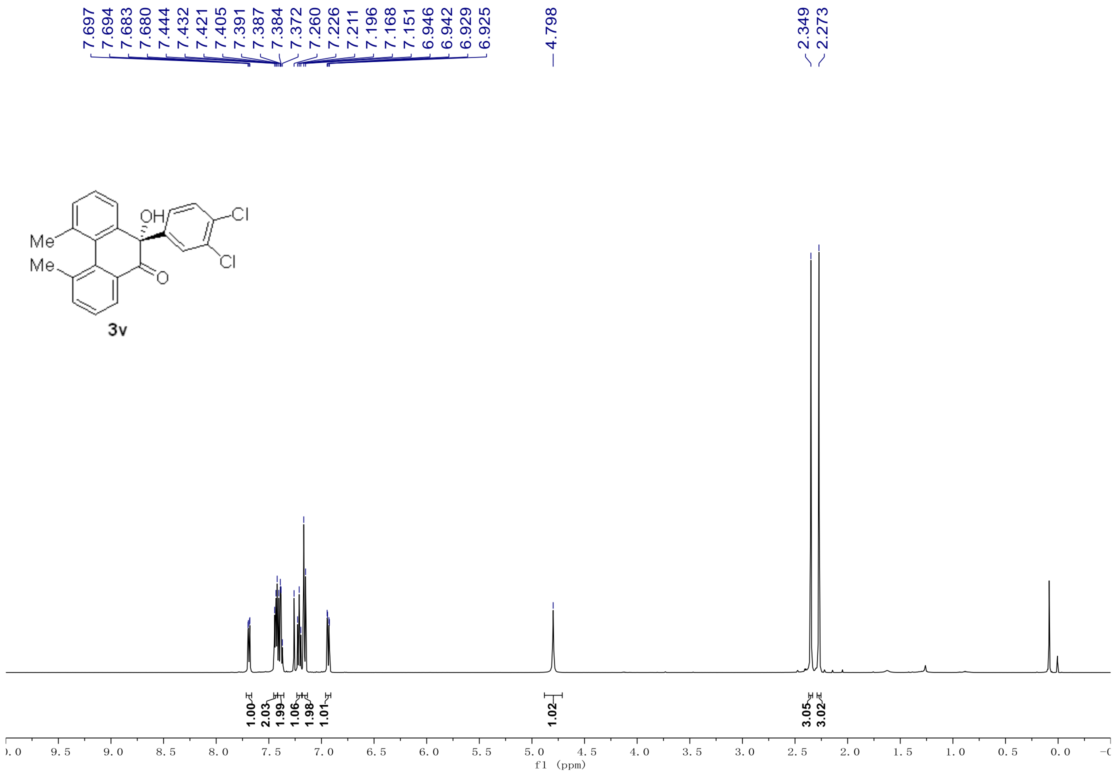
7.697
7.694
7.683
7.680
7.444
7.432
7.421
7.405
7.391
7.387
7.384
7.372
7.260
7.226
7.211
7.196
7.168
7.151
6.946
6.942
6.929
6.925
4.798
2.349
2.273
OH
Cl
Cl
Me
Me
O
3v
1.00
2.03
1.99
1.06
1.98
1.01
1.02
3.05
3.02
f1 (ppm)

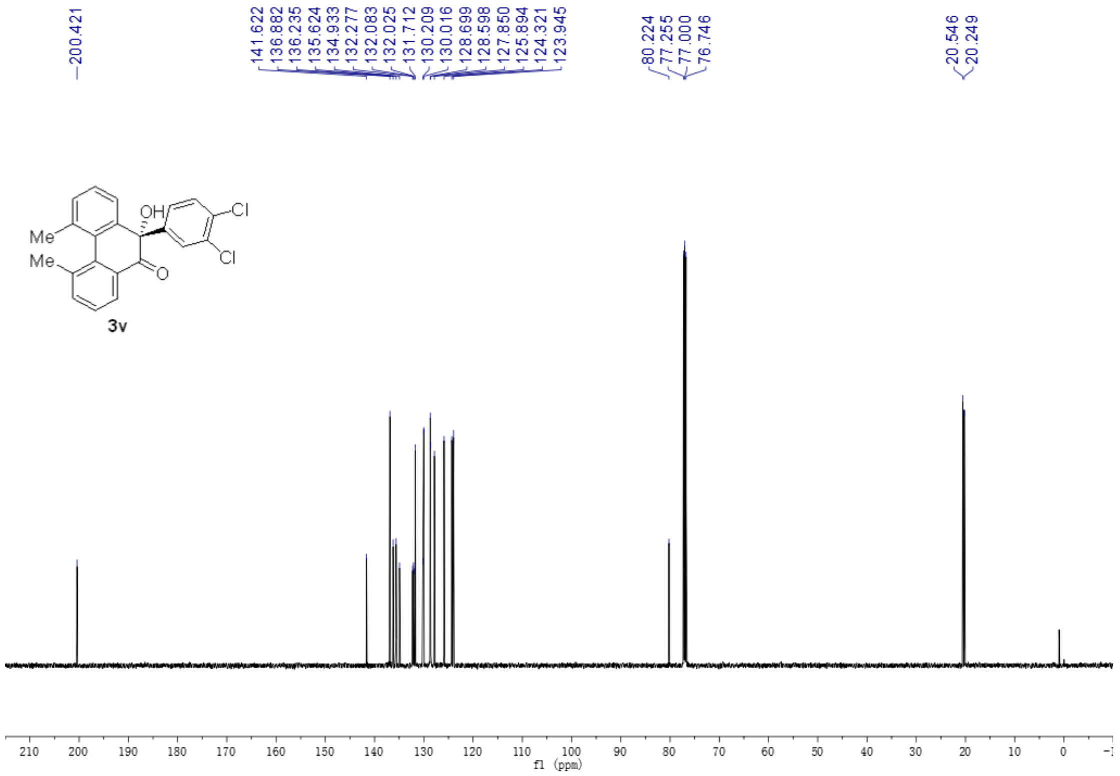

200.421
141.622
136.882
136.235
135.624
134.933
132.277
132.083
132.025
131.712
130.209
130.016
128.699
128.598
127.850
125.894
124.321
123.945
80.224
77.255
77.000
76.746
20.546
20.249
Me
Me
OH
Cl
Cl
O
3v
210 200 190 180 170 160 150 140 130 120 110 100 90 80 70 60 50 40 30 20 10 0
f1 (ppm)

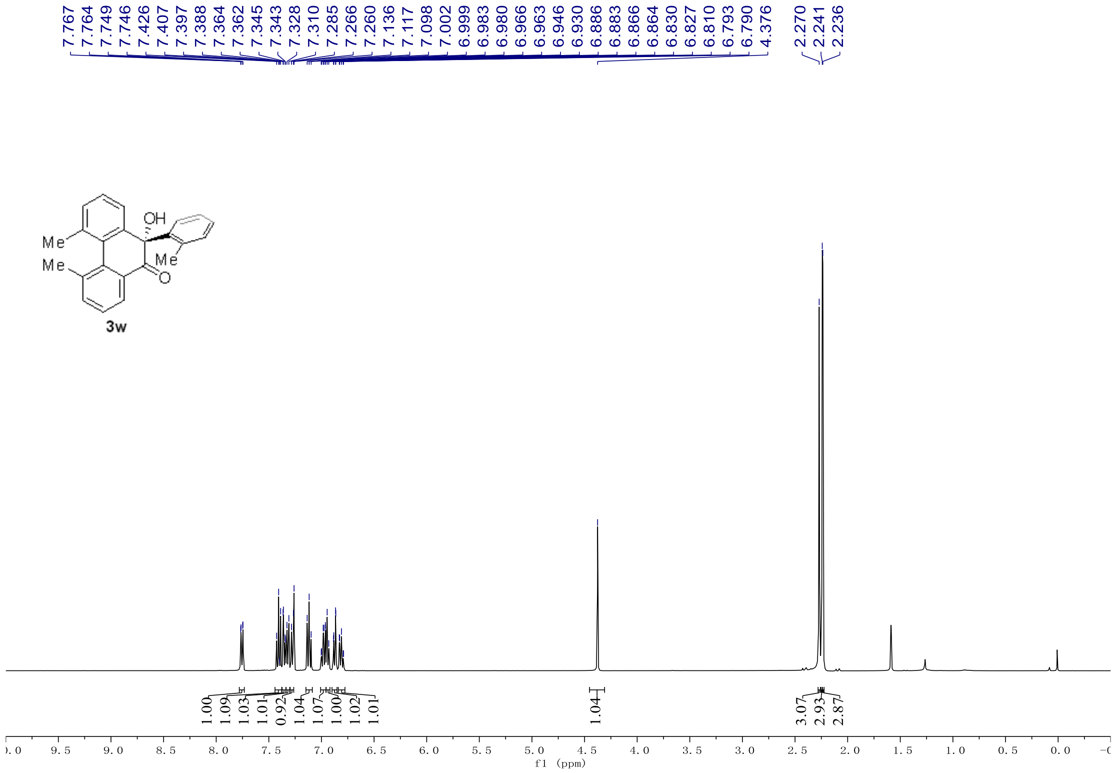

7.767
7.764
7.749
7.746
7.426
7.407
7.397
7.388
7.364
7.362
7.345
7.343
7.328
7.310
7.285
7.266
7.260
7.136
7.117
7.098
7.002
6.999
6.983
6.980
6.966
6.963
6.946
6.930
6.886
6.883
6.866
6.864
6.830
6.827
6.810
6.793
6.790
4.376
2.270
2.241
2.236
Me
Me
OH
Me
O
3w
1.00
1.09
1.03
1.01
0.92
1.04
1.07
1.00
1.02
1.01
1.04
3.07
2.93
2.87
9.5 9.0 8.5 8.0 7.5 7.0 6.5 6.0 5.5 5.0 4.5 4.0 3.5 3.0 2.5 2.0 1.5 1.0 0.5 0.0
f1 (ppm)

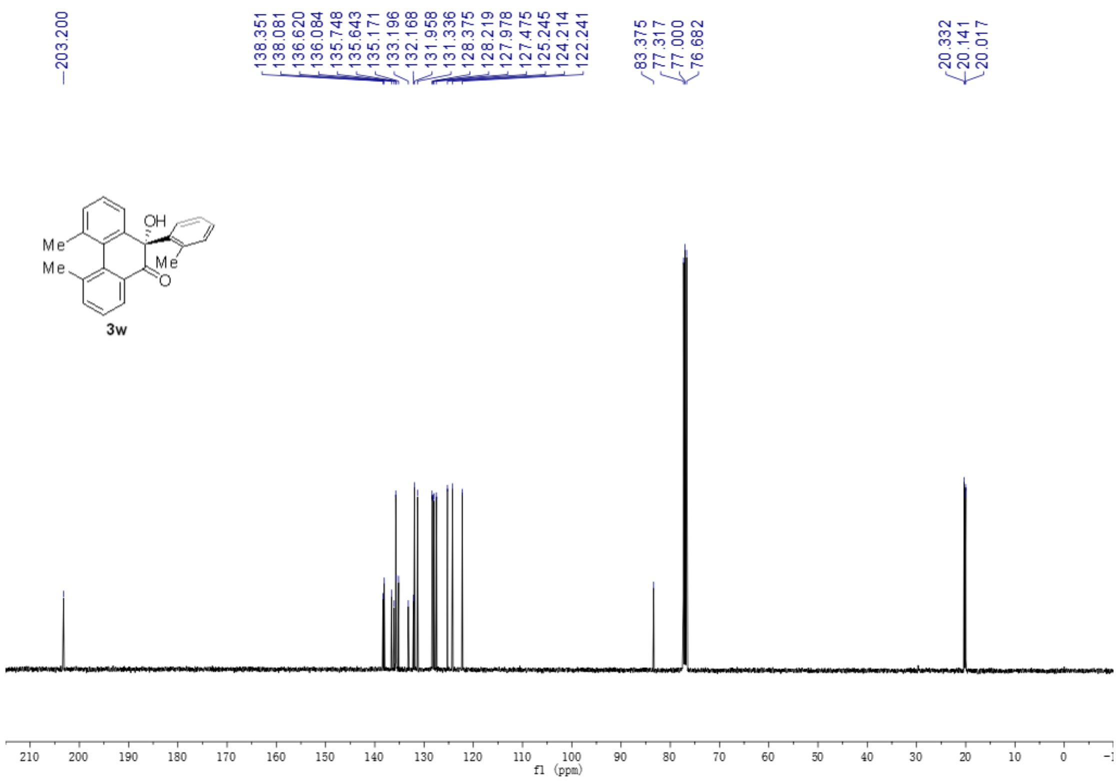
203.200
138.351
138.081
136.620
136.084
135.748
135.643
135.171
133.196
132.168
131.958
131.336
128.375
128.219
127.978
127.475
125.245
124.214
122.241
83.375
77.317
77.000
76.682
20.332
20.141
20.017
OH
Me
Me
Me
O
3w
f1 (ppm)

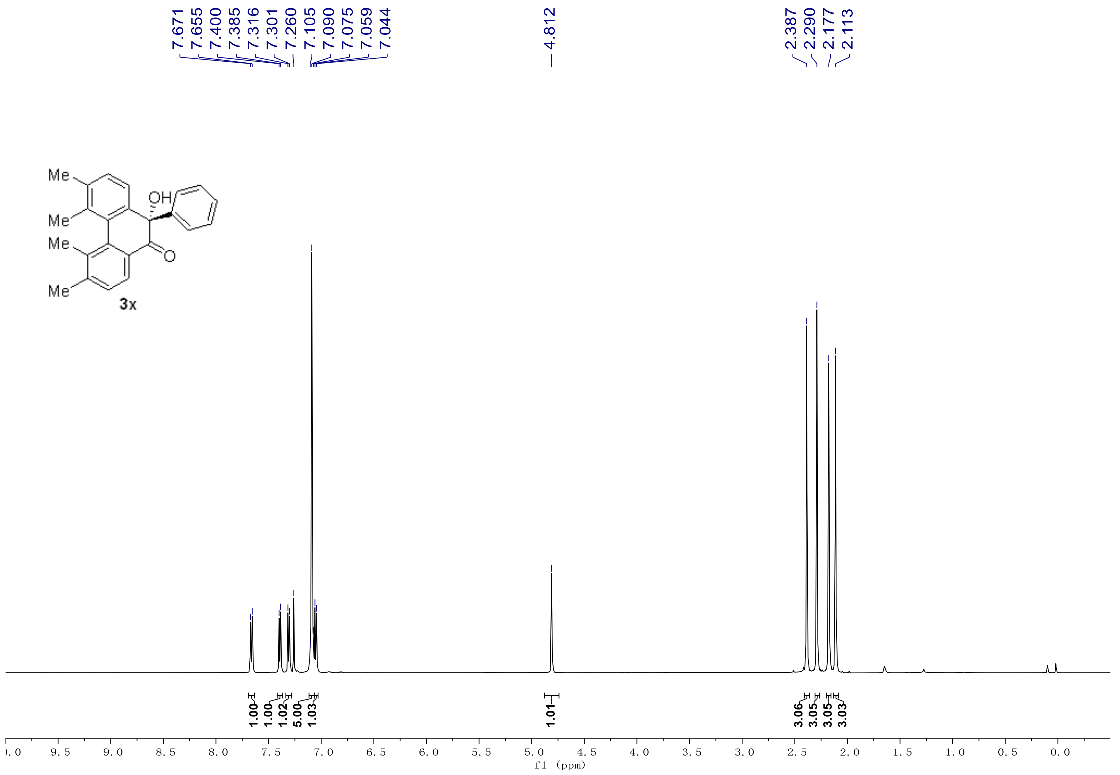
7.671
7.655
7.400
7.385
7.316
7.301
7.260
7.105
7.090
7.075
7.059
7.044
4.812
2.387
2.290
2.177
2.113
Me
Me
Me
Me
OH
O
3x
1.00
1.00
1.02
5.00
1.03
1.01
3.06
3.05
3.05
3.03
f1 (ppm)

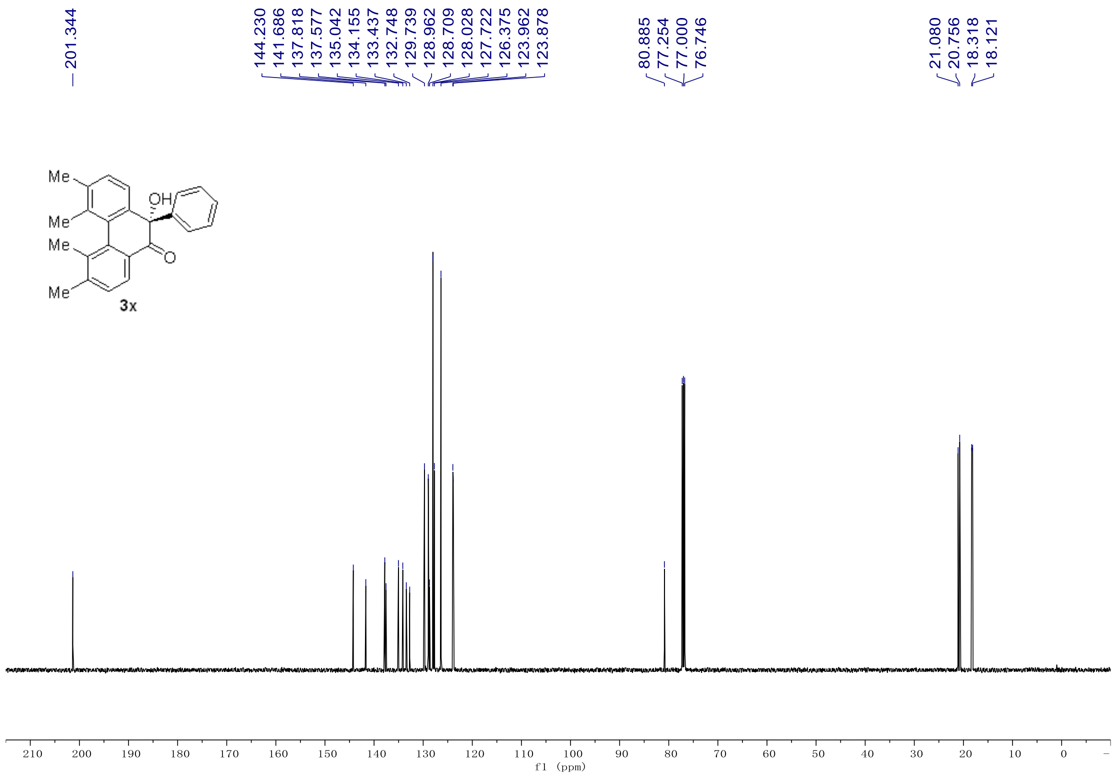
201.344
144.230
141.686
137.818
137.577
135.042
134.155
133.437
132.748
129.739
128.962
128.709
128.028
127.722
126.375
123.962
123.878
80.885
77.254
77.000
76.746
21.080
20.756
18.318
18.121
Me
Me
Me
Me
OH
O
3x
f1 (ppm)

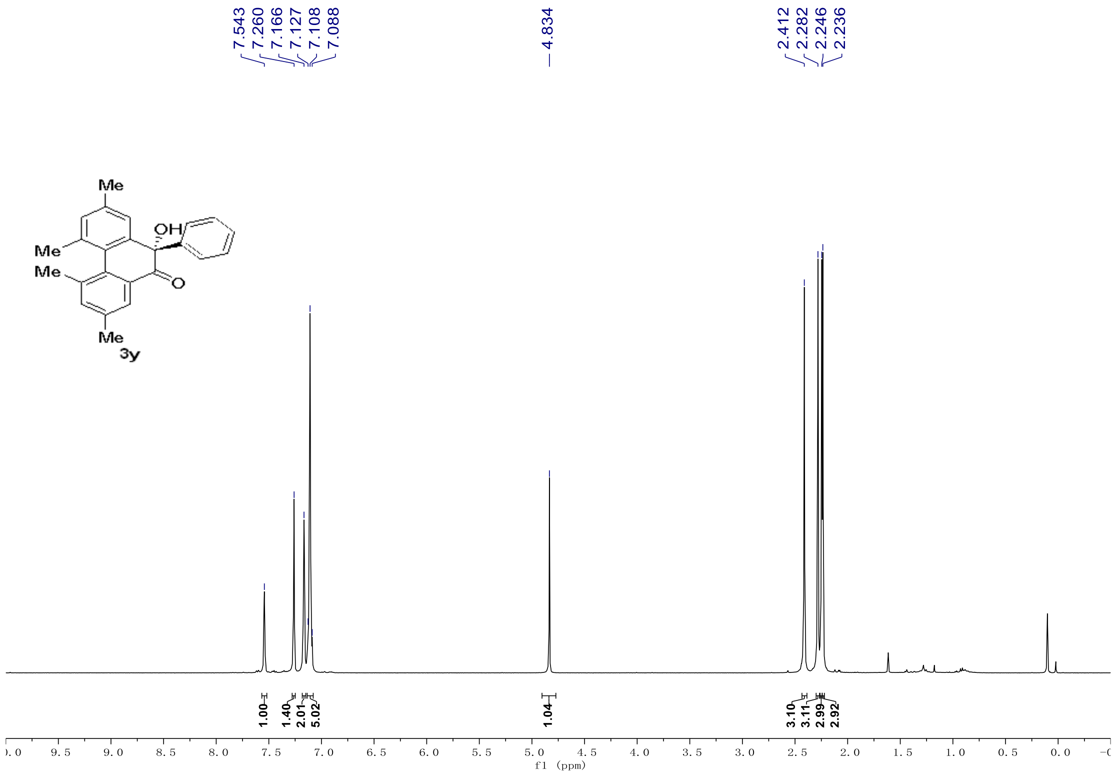
7.543
7.260
7.166
7.127
7.108
7.088
4.834
2.412
2.282
2.246
2.236
Me
Me
Me
Me
OH
O
3y
1.00
1.40
2.01
5.02
1.04
3.10
3.11
2.99
2.92
f1 (ppm)

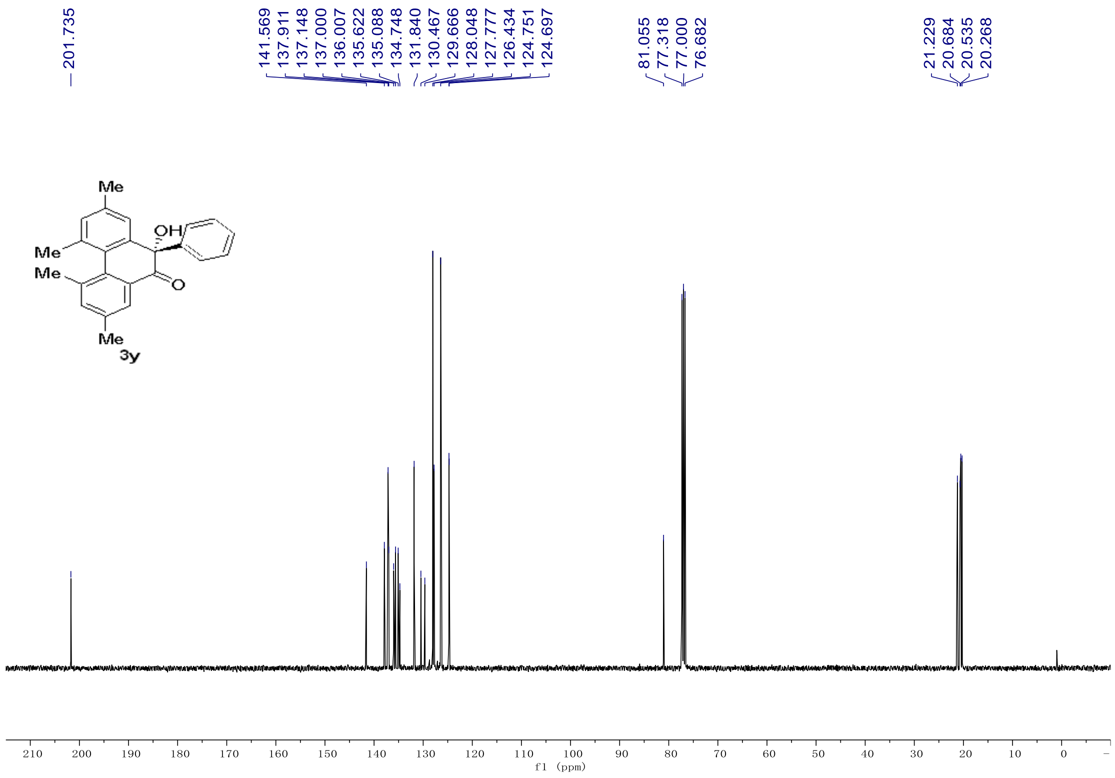
201.735
141.569
137.911
137.148
137.000
136.007
135.622
135.088
134.748
131.840
130.467
129.666
128.048
127.777
126.434
124.751
124.697
81.055
77.318
77.000
76.682
21.229
20.684
20.535
20.268
Me
OH
Me
Me
O
Me
3y
210 200 190 180 170 160 150 140 130 120 110 100 90 80 70 60 50 40 30 20 10 0
f1 (ppm)

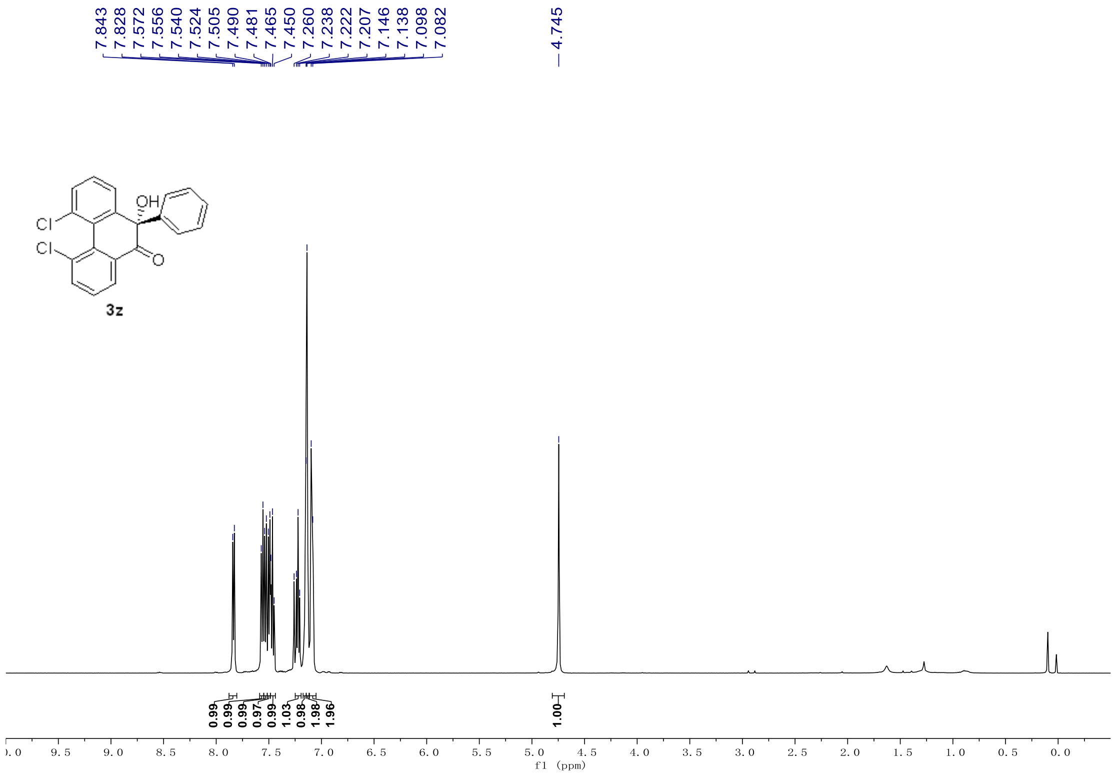
7.843
7.828
7.572
7.556
7.540
7.524
7.505
7.490
7.481
7.465
7.450
7.260
7.238
7.222
7.207
7.146
7.138
7.098
7.082
4.745
Cl
OH
Cl
O
3z
0.99
0.99
0.99
0.97
0.99
1.03
0.98
1.98
1.96
1.00
9.5 9.0 8.5 8.0 7.5 7.0 6.5 6.0 5.5 5.0 4.5 4.0 3.5 3.0 2.5 2.0 1.5 1.0 0.5 0.0
f1 (ppm)

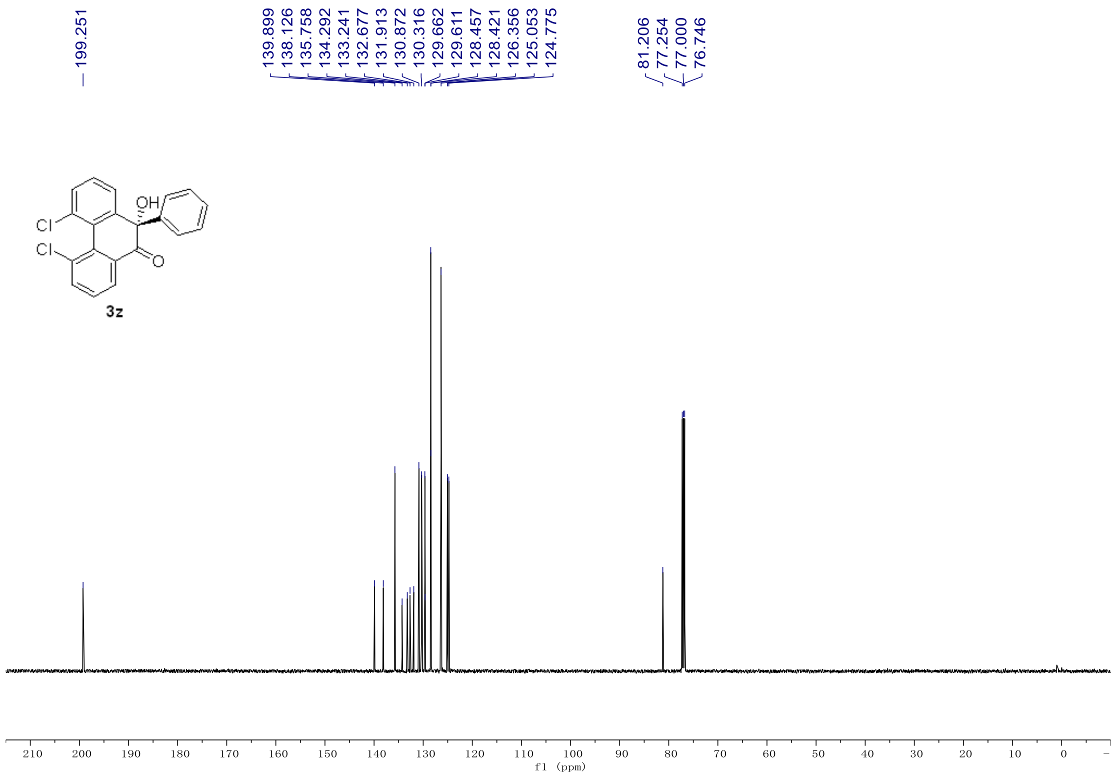
199.251
139.899
138.126
135.758
134.292
133.241
132.677
131.913
130.872
130.316
129.662
129.611
128.457
128.421
126.356
125.053
124.775
81.206
77.254
77.000
76.746
OH
Cl
Cl
O
3z
210 200 190 180 170 160 150 140 130 120 110 100 90 80 70 60 50 40 30 20 10 0
f1 (ppm)

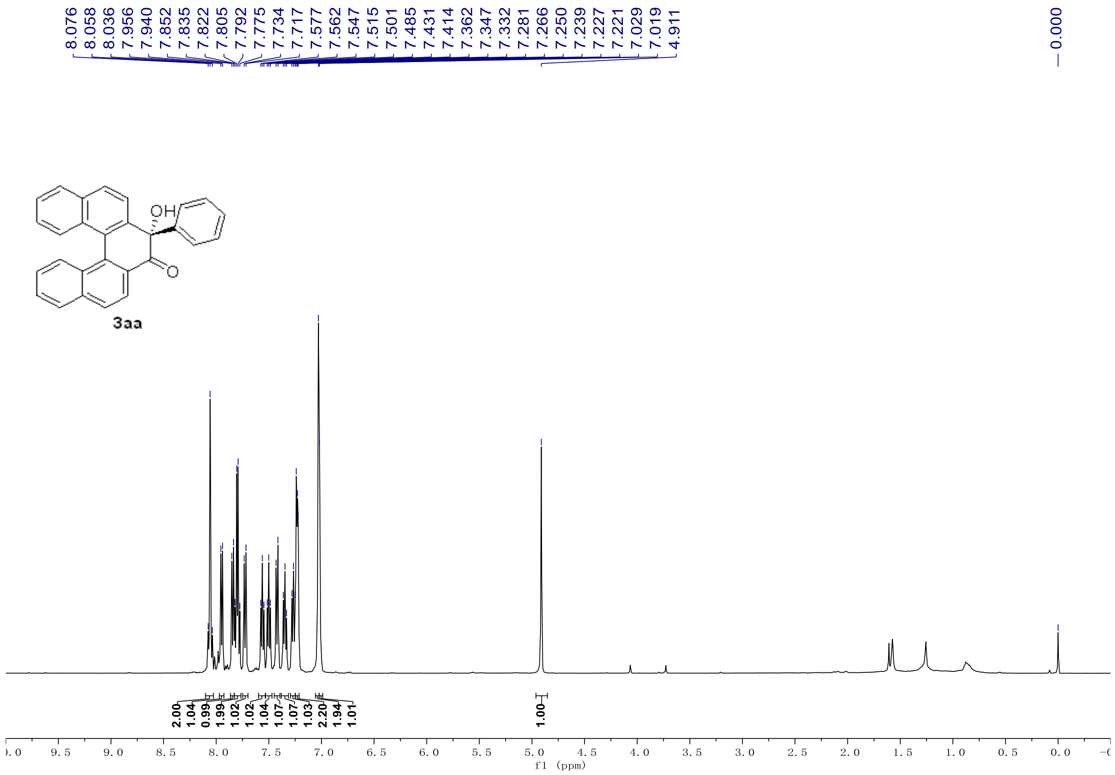
8.076
8.058
8.036
7.956
7.940
7.852
7.835
7.822
7.805
7.792
7.775
7.734
7.717
7.577
7.562
7.547
7.515
7.501
7.485
7.431
7.414
7.362
7.347
7.332
7.281
7.266
7.250
7.239
7.227
7.221
7.029
7.019
4.911
0.000
OH
O
3aa
2.00
1.04
0.99
1.99
1.02
1.02
1.04
1.07
1.07
1.03
2.20
1.94
1.01
1.00
9.5 9.0 8.5 8.0 7.5 7.0 6.5 6.0 5.5 5.0 4.5 4.0 3.5 3.0 2.5 2.0 1.5 1.0 0.5 0.0
f1 (ppm)

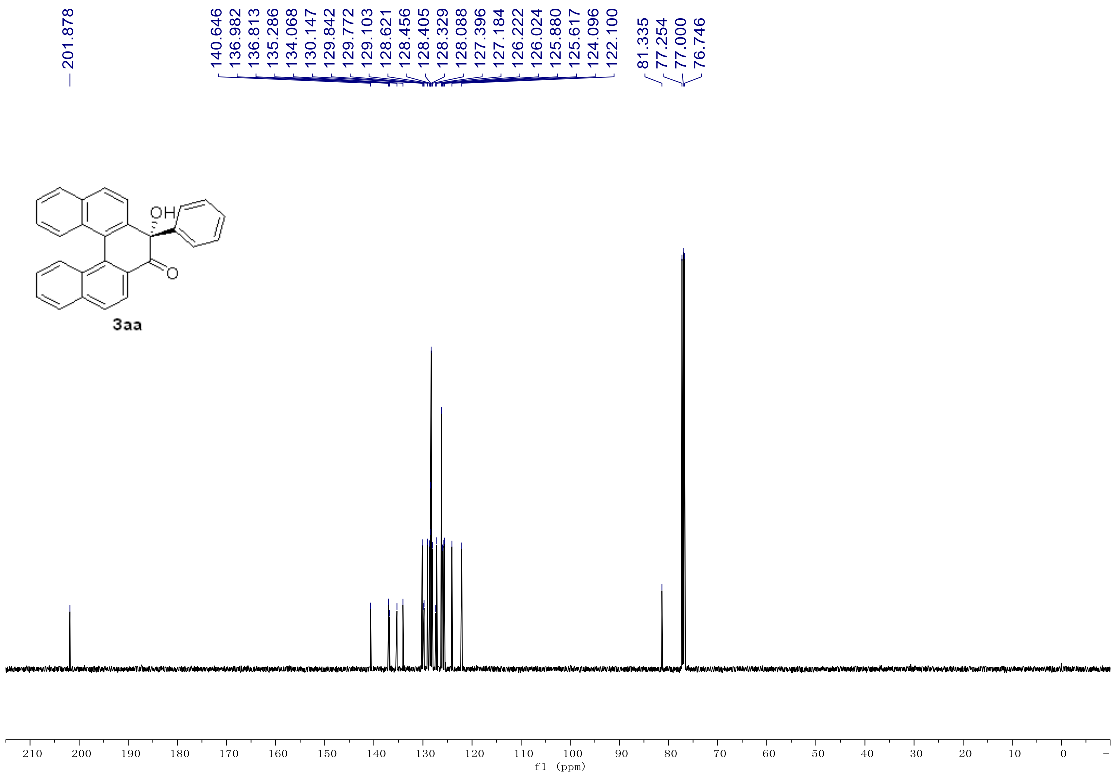
201.878
140.646
136.982
136.813
135.286
134.068
130.147
129.842
129.772
129.103
128.621
128.456
128.405
128.329
128.088
127.396
127.184
126.222
126.024
125.880
125.617
124.096
122.100
81.335
77.254
77.000
76.746
OH
O
3aa
210 200 190 180 170 160 150 140 130 120 110 100 90 80 70 60 50 40 30 20 10 0
f1 (ppm)

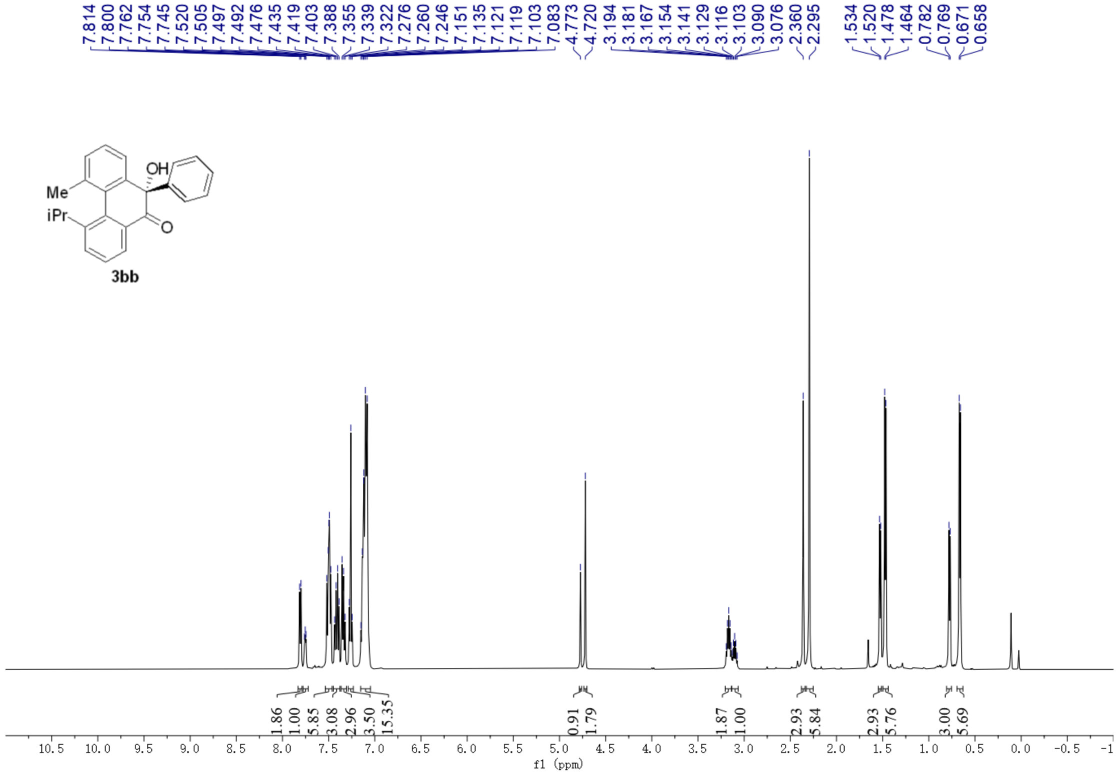
7.814
7.800
7.762
7.754
7.745
7.520
7.505
7.497
7.492
7.476
7.435
7.419
7.403
7.388
7.355
7.339
7.322
7.276
7.260
7.246
7.151
7.135
7.121
7.119
7.103
7.083
4.773
4.720
3.194
3.181
3.167
3.154
3.141
3.129
3.116
3.103
3.090
3.076
2.360
2.295
1.534
1.520
1.478
1.464
0.782
0.769
0.671
0.658
Me
iPr
OH
O
3bb
1.86
1.00
5.85
3.08
2.96
3.50
15.35
0.91
1.79
1.87
1.00
2.93
5.84
2.93
5.76
3.00
5.69
10.5 10.0 9.5 9.0 8.5 8.0 7.5 7.0 6.5 6.0 5.5 5.0 4.5 4.0 3.5 3.0 2.5 2.0 1.5 1.0 0.5 0.0 -0.5 -1
f1 (ppm)

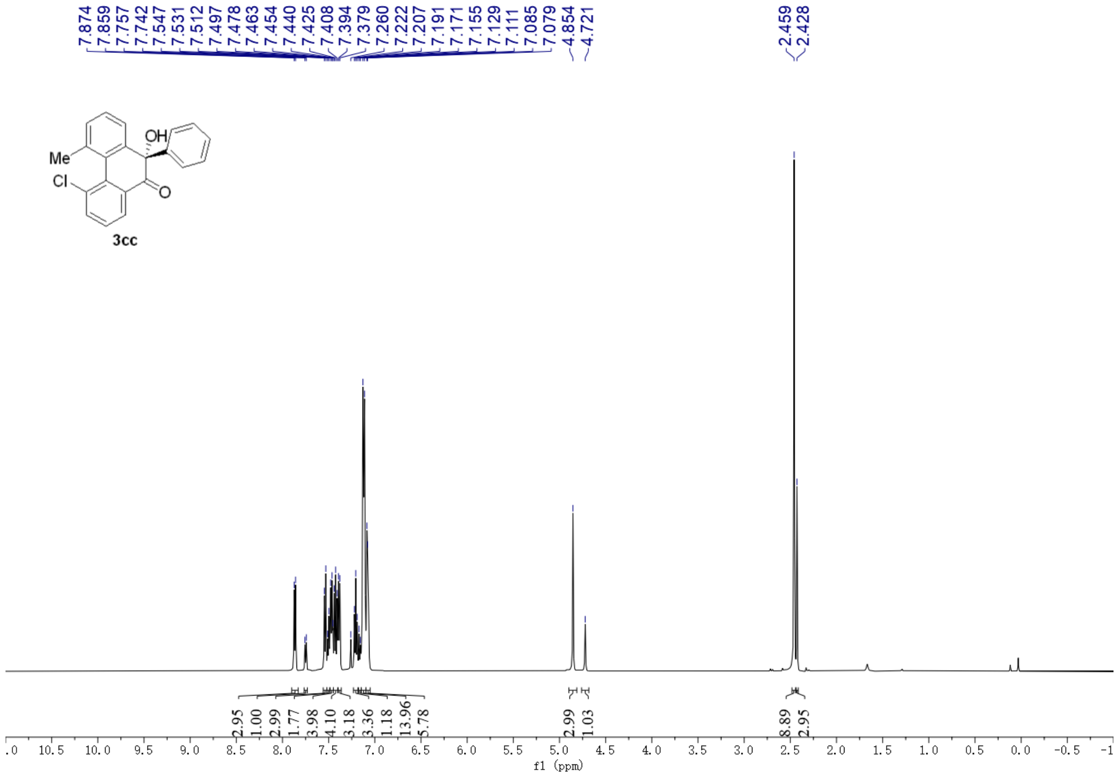

3cc

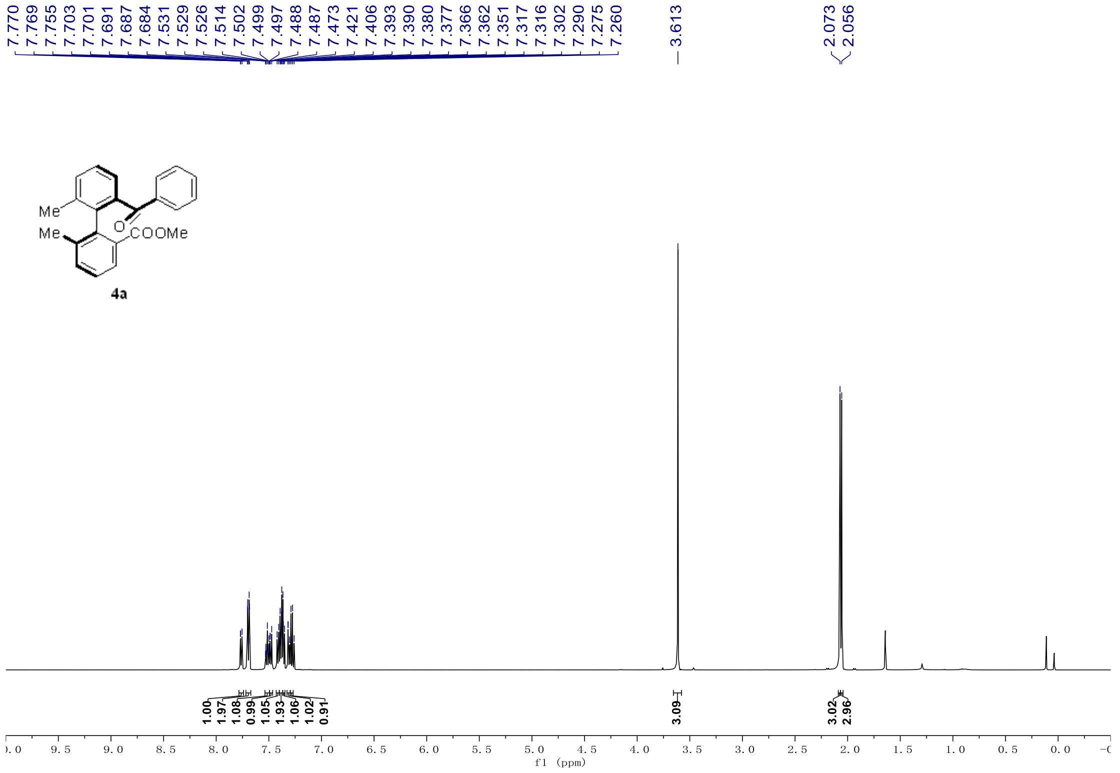

4a

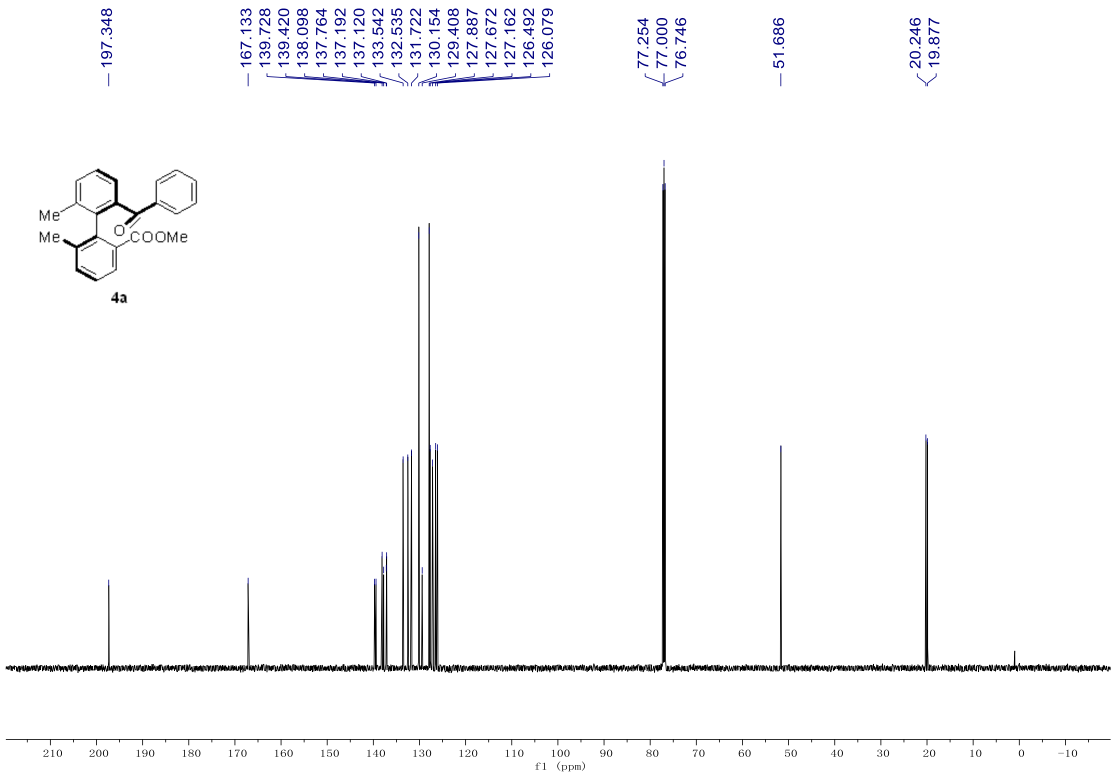
197.348
167.133
139.728
139.420
138.098
137.764
137.192
137.120
133.542
132.535
131.722
130.154
129.408
127.887
127.672
127.162
126.492
126.079
77.254
77.000
76.746
51.686
20.246
19.877
Me
Me
O
COOMe
4a
f1 (ppm)

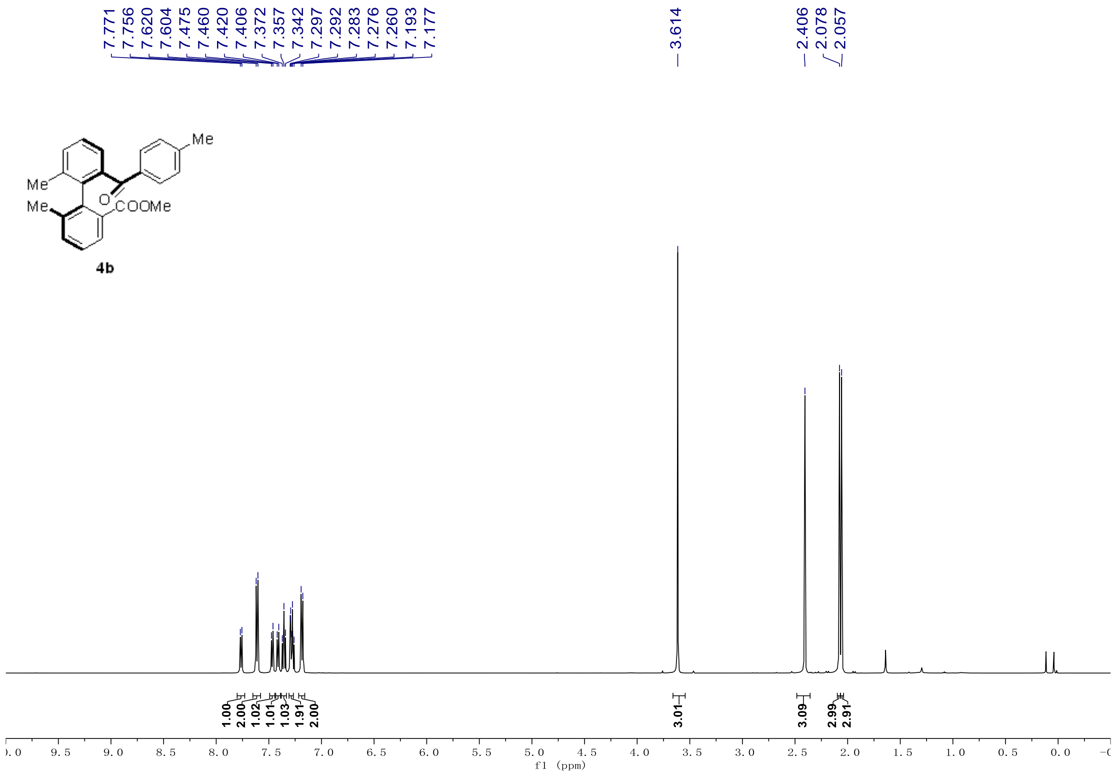
7.771
7.756
7.620
7.604
7.475
7.460
7.420
7.406
7.372
7.357
7.342
7.297
7.292
7.283
7.276
7.260
7.193
7.177
3.614
2.406
2.078
2.057
Me
Me
Me
O
COOMe
4b
1.00
2.00
1.02
1.01
1.03
1.91
2.00
3.01
3.09
2.99
2.91
f1 (ppm)

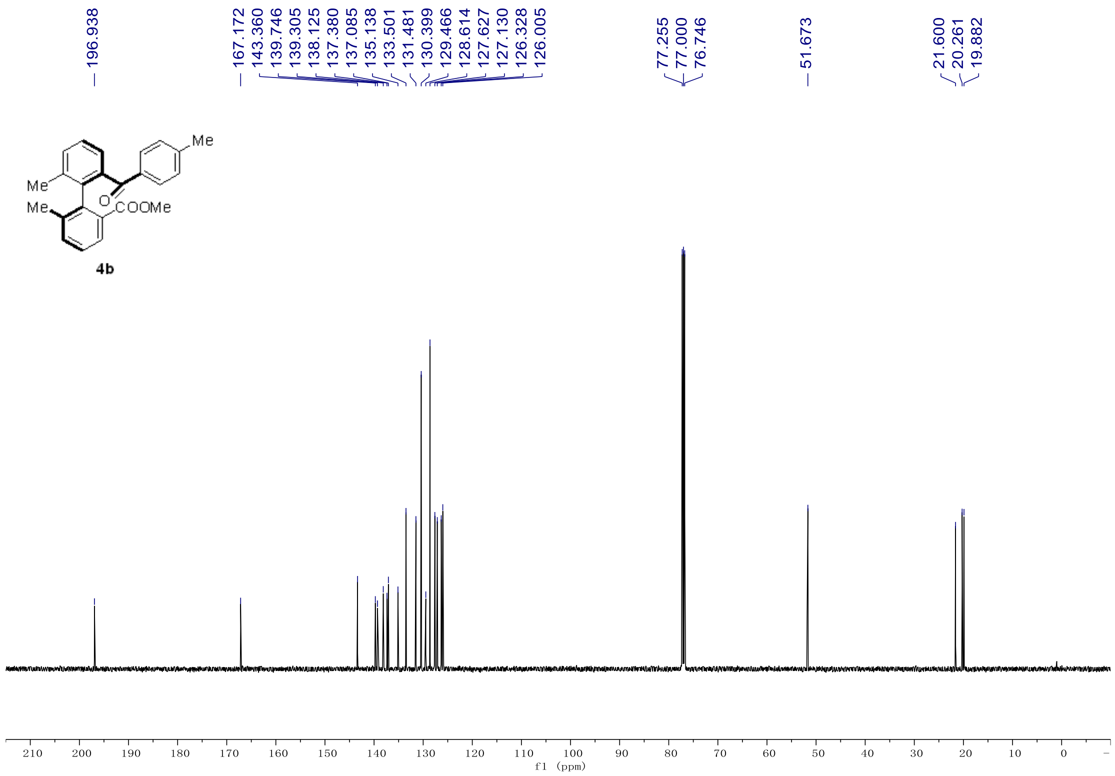
196.938
167.172
143.360
139.746
139.305
138.125
137.380
137.085
135.138
133.501
131.481
130.399
129.466
128.614
127.627
127.130
126.328
126.005
77.255
77.000
76.746
51.673
21.600
20.261
19.882
Me
Me
Me
O
COOMe
4b
210 200 190 180 170 160 150 140 130 120 110 100 90 80 70 60 50 40 30 20 10 0
f1 (ppm)

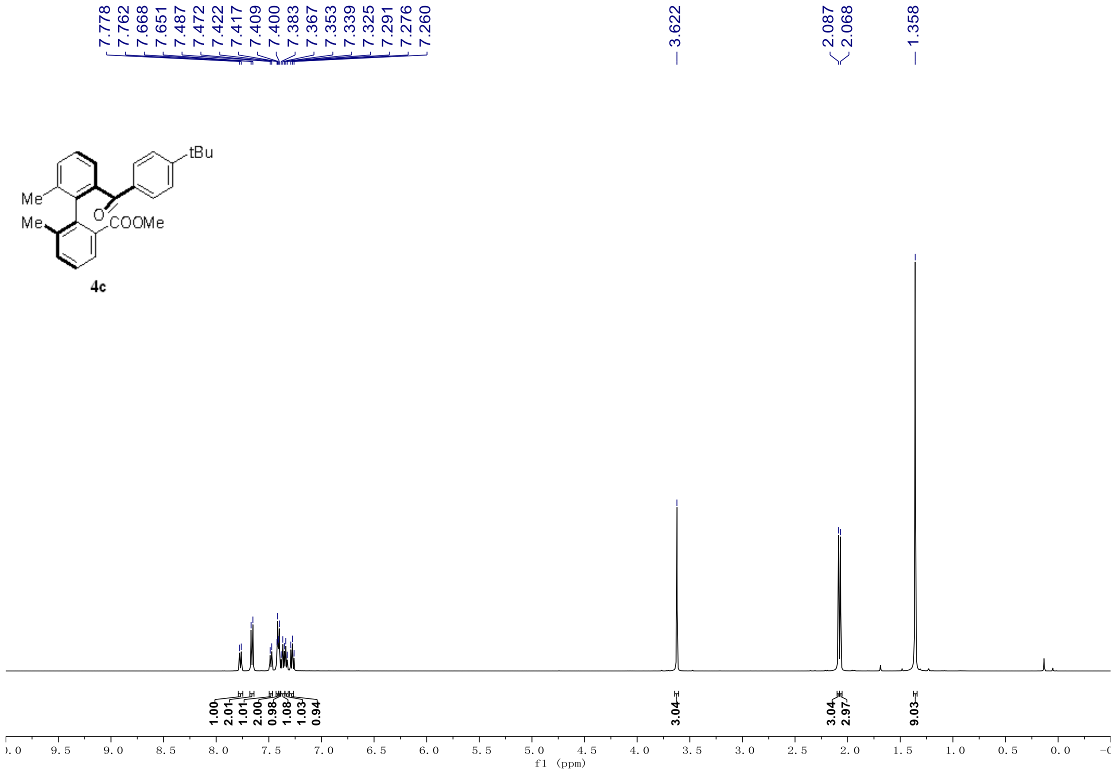
7.778
7.762
7.668
7.651
7.487
7.472
7.422
7.417
7.409
7.400
7.383
7.367
7.353
7.339
7.325
7.291
7.276
7.260
3.622
2.087
2.068
1.358
tBu
Me
Me
O
COOMe
4c
1.00
2.01
1.01
2.00
0.98
1.08
1.03
0.94
3.04
3.04
2.97
9.03
9.5 9.0 8.5 8.0 7.5 7.0 6.5 6.0 5.5 5.0 4.5 4.0 3.5 3.0 2.5 2.0 1.5 1.0 0.5 0.0
f1 (ppm)

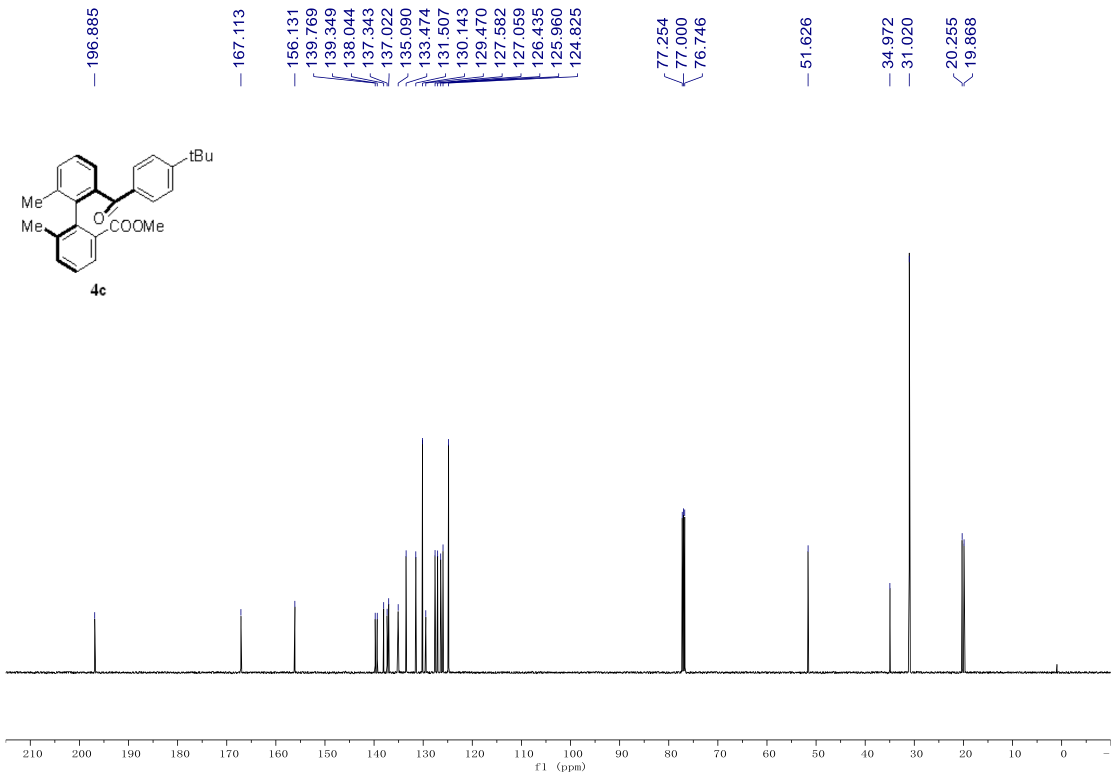
196.885
167.113
156.131
139.769
139.349
138.044
137.343
137.022
135.090
133.474
131.507
130.143
129.470
127.582
127.059
126.435
125.960
124.825
77.254
77.000
76.746
51.626
34.972
31.020
20.255
19.868
tBu
Me
Me
O
COOMe
4c
f1 (ppm)

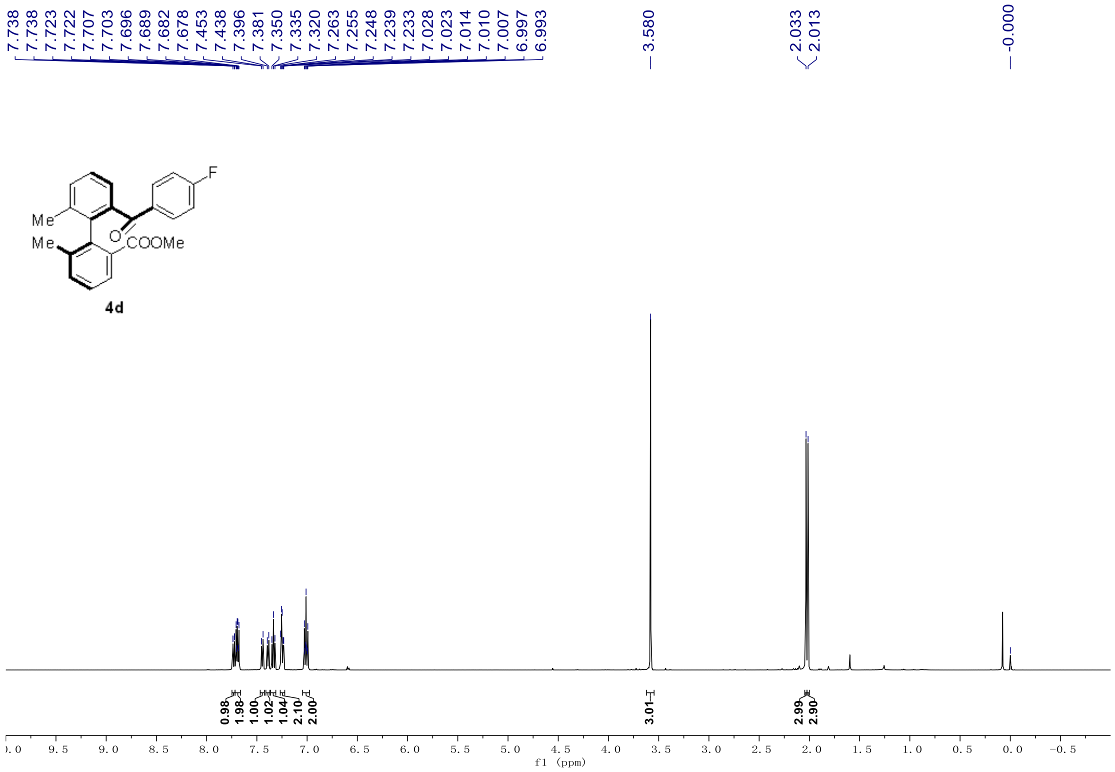
7.738
7.738
7.723
7.722
7.707
7.703
7.696
7.689
7.682
7.678
7.453
7.438
7.396
7.381
7.350
7.335
7.320
7.263
7.255
7.248
7.239
7.233
7.028
7.023
7.014
7.010
7.007
6.997
6.993
3.580
2.033
2.013
-0.000
F
Me
Me
O
COOMe
4d
0.98
1.98
1.00
1.02
1.04
2.10
2.00
3.01
2.99
2.90
f1 (ppm)

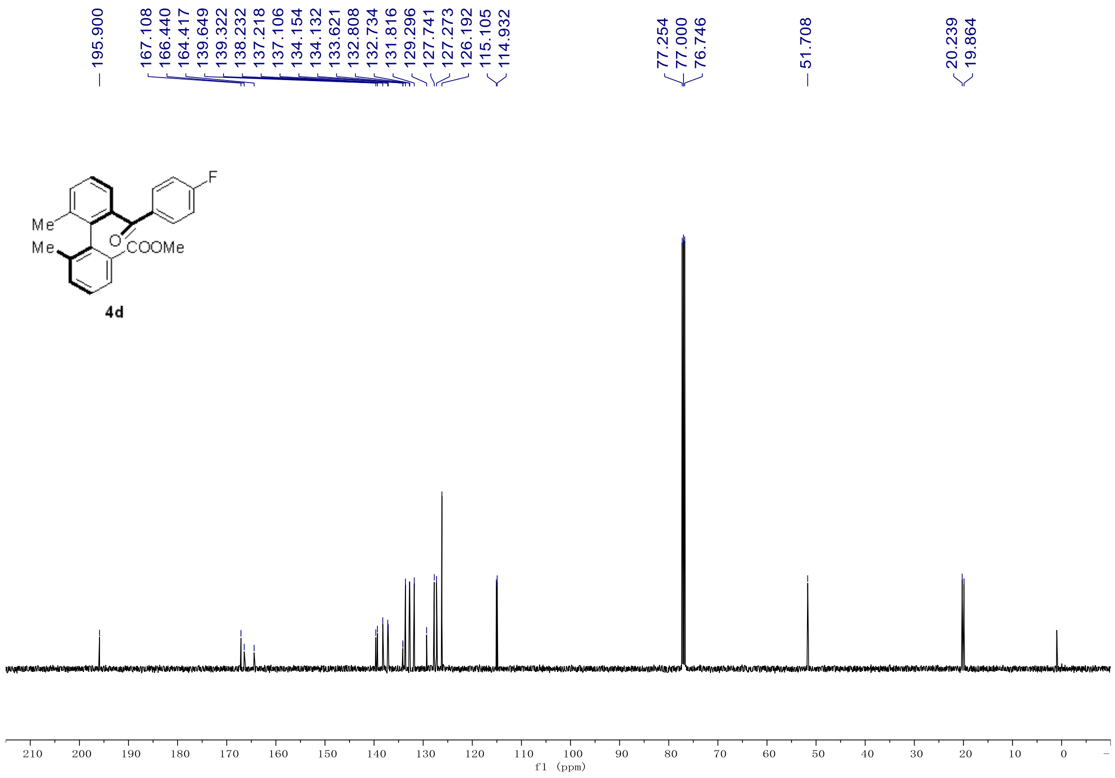
195.900
167.108
166.440
164.417
139.649
139.322
138.232
137.218
137.106
134.154
134.132
133.621
132.808
132.734
131.816
129.296
127.741
127.273
126.192
115.105
114.932
77.254
77.000
76.746
51.708
20.239
19.864
Me
Me
COOMe
F
O
4d
f1 (ppm)

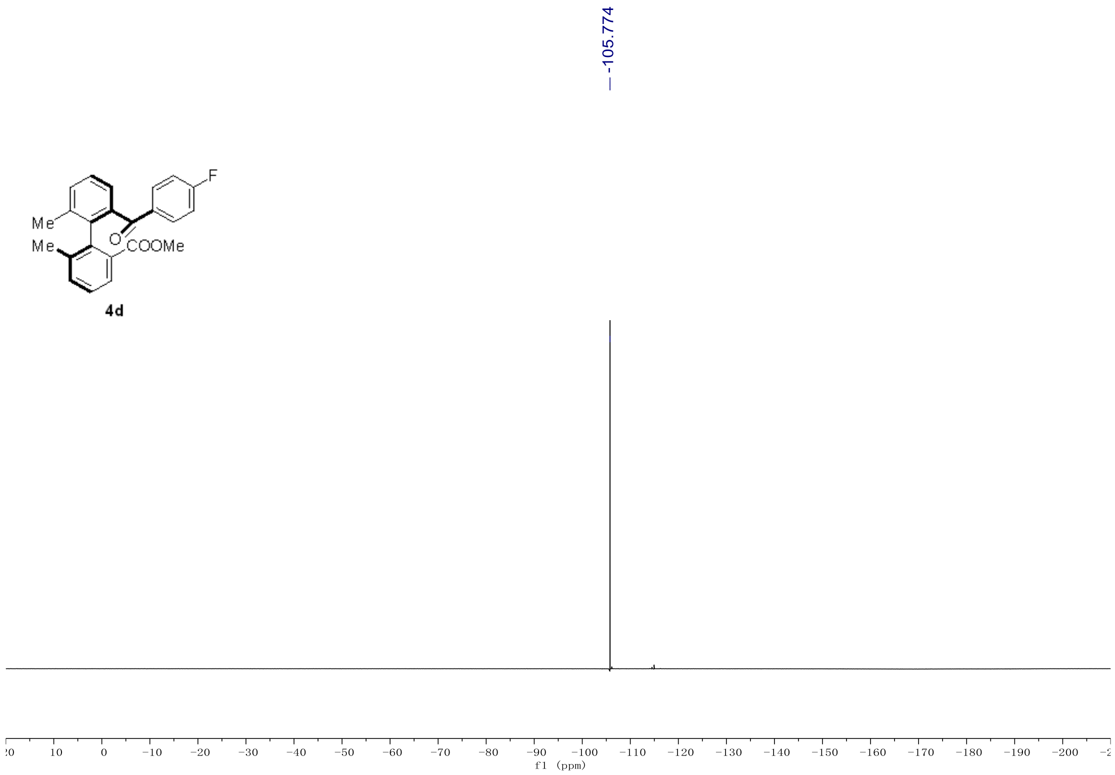
-105.774
Me
Me
COOMe
F
O
4d
f1 (ppm)

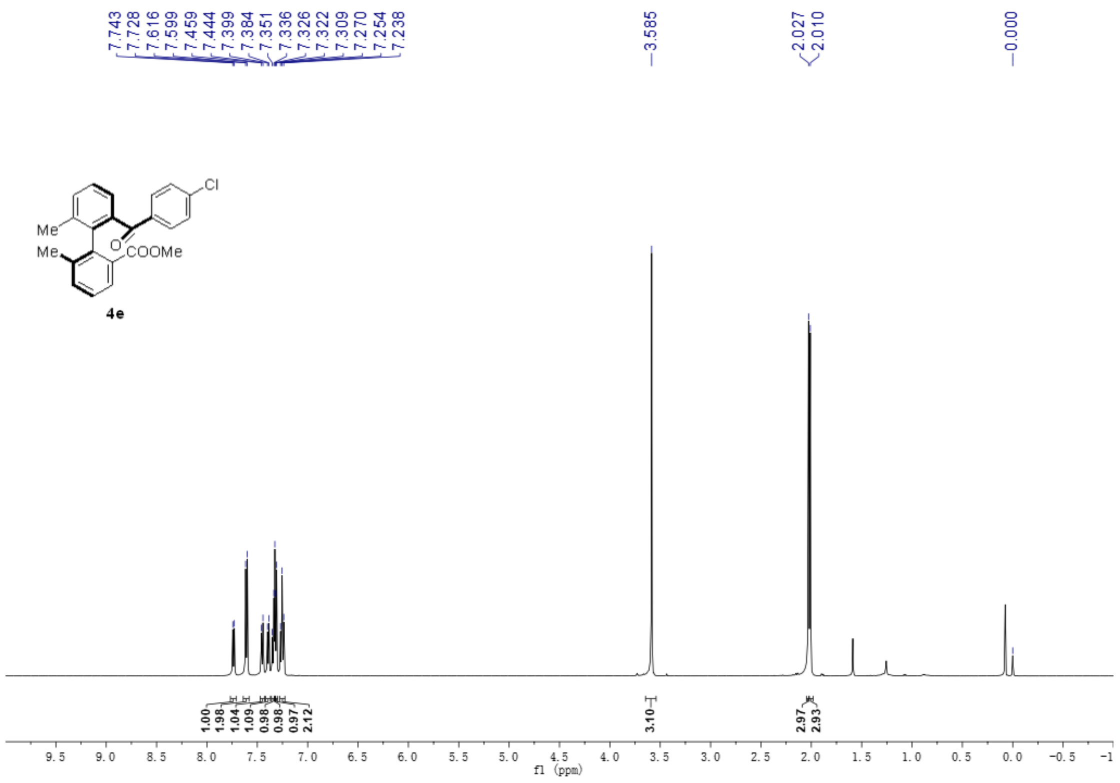
7.743
7.728
7.616
7.599
7.459
7.444
7.399
7.384
7.351
7.336
7.326
7.322
7.309
7.270
7.254
7.238
3.585
2.027
2.010
0.000
Cl
Me
Me
O
COOMe
4e
1.00
1.98
1.04
1.09
0.98
0.98
0.97
2.12
3.10
2.97
2.93
9.5 9.0 8.5 8.0 7.5 7.0 6.5 6.0 5.5 5.0 4.5 4.0 3.5 3.0 2.5 2.0 1.5 1.0 0.5 0.0 -0.5 -1
f1 (ppm)

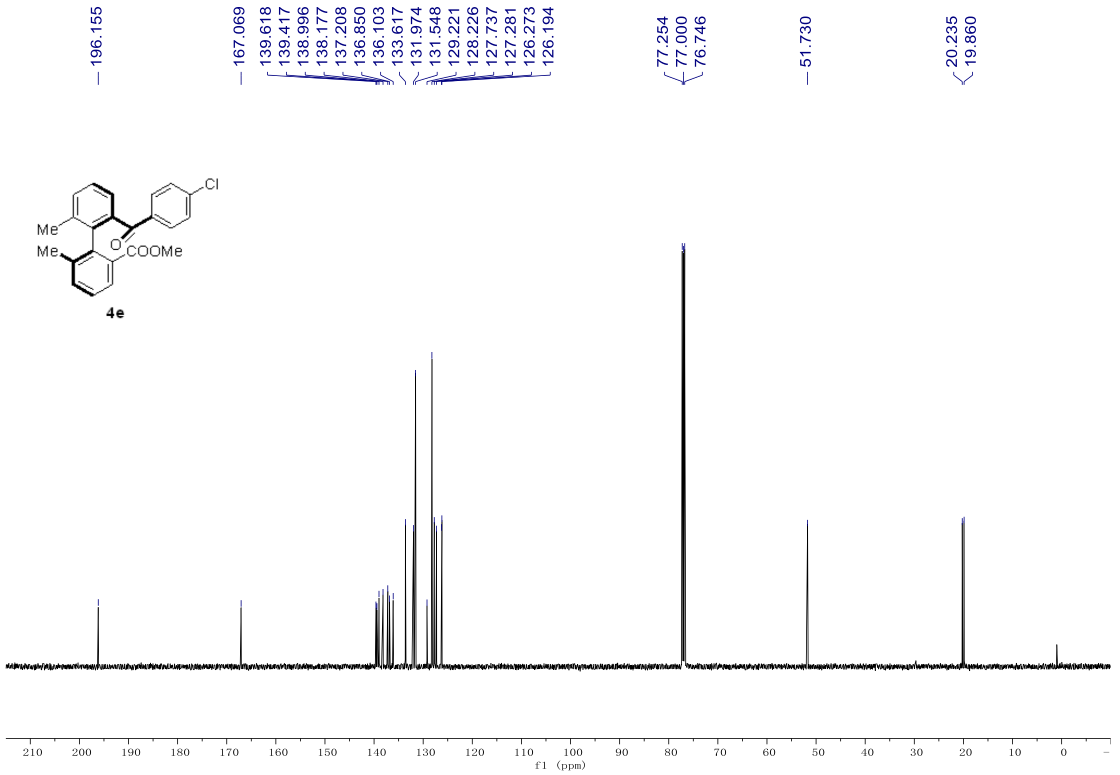
196.155
167.069
139.618
139.417
138.996
138.177
137.208
136.850
136.103
133.617
131.974
131.548
129.221
128.226
127.737
127.281
126.273
126.194
77.254
77.000
76.746
51.730
20.235
19.860
Cl
Me
Me
O
COOMe
4e
210 200 190 180 170 160 150 140 130 120 110 100 90 80 70 60 50 40 30 20 10 0
f1 (ppm)

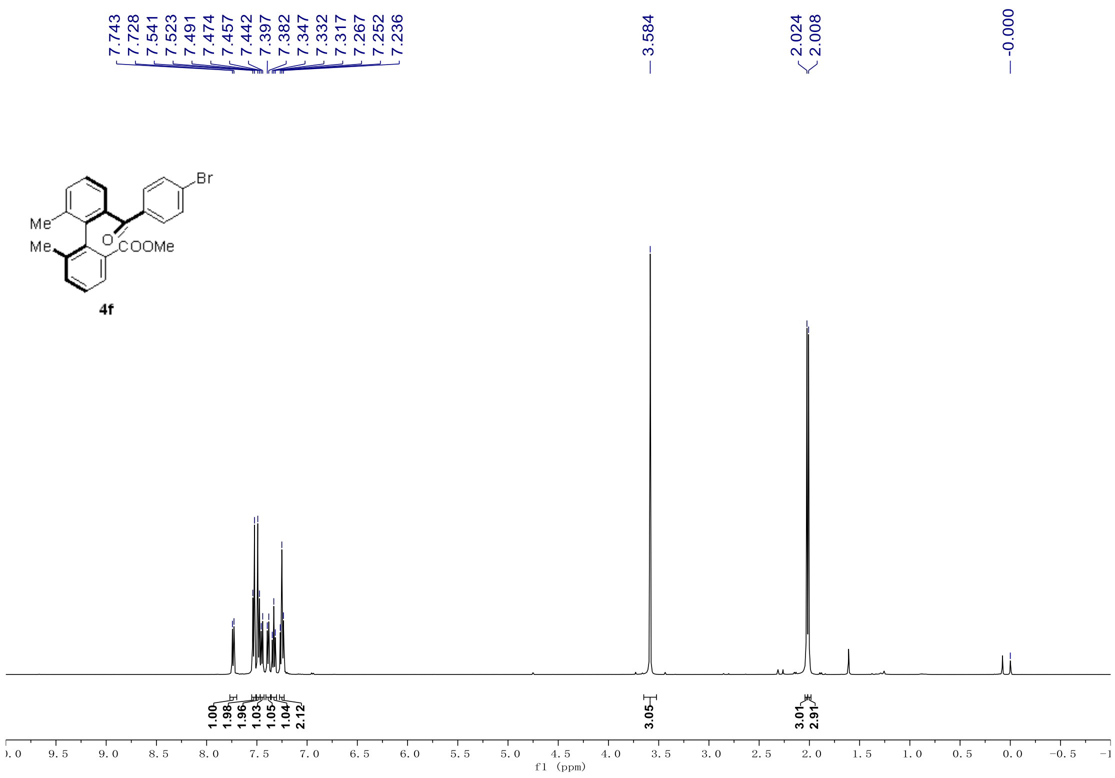
7.743
7.728
7.541
7.523
7.491
7.474
7.457
7.442
7.397
7.382
7.347
7.332
7.317
7.267
7.252
7.236
3.584
2.024
2.008
-0.000
Br
Me
Me
O
COOMe
4f
1.00
1.98
1.96
1.03
1.05
1.04
2.12
3.05
3.01
2.91
f1 (ppm)

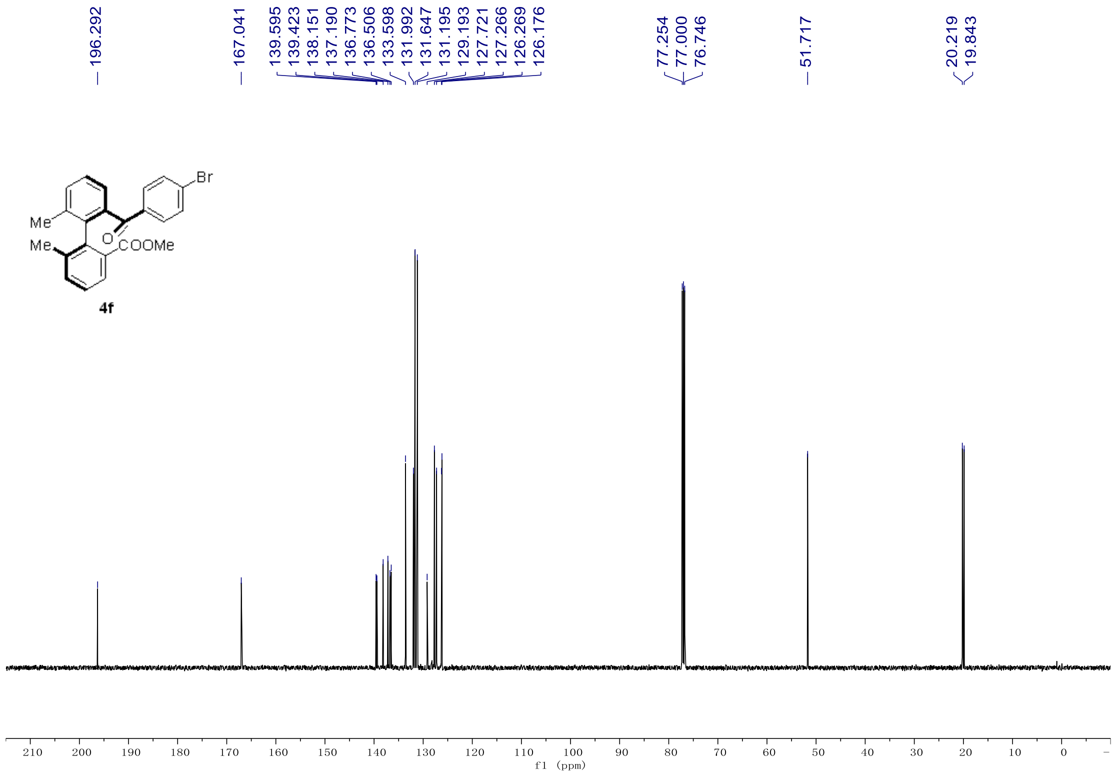
196.292
167.041
139.595
139.423
138.151
137.190
136.773
136.506
133.598
131.992
131.647
131.195
129.193
127.721
127.266
126.269
126.176
77.254
77.000
76.746
51.717
20.219
19.843
Br
Me
Me
O
COOMe
4f
f1 (ppm)

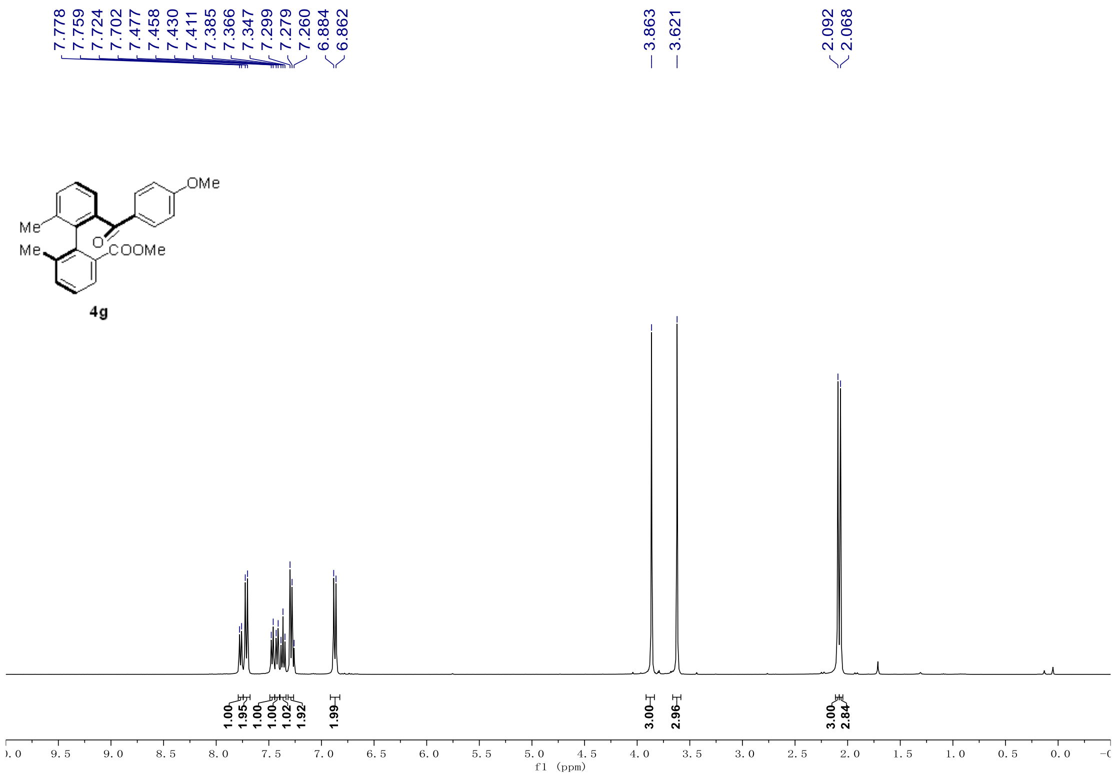

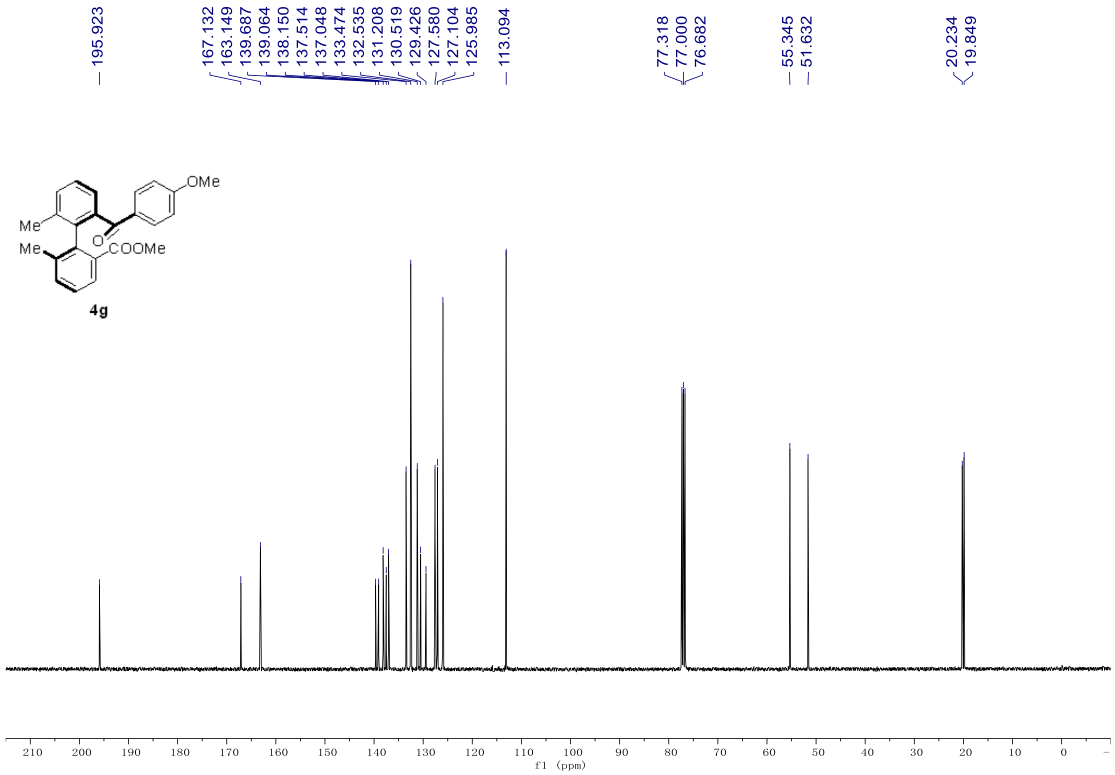

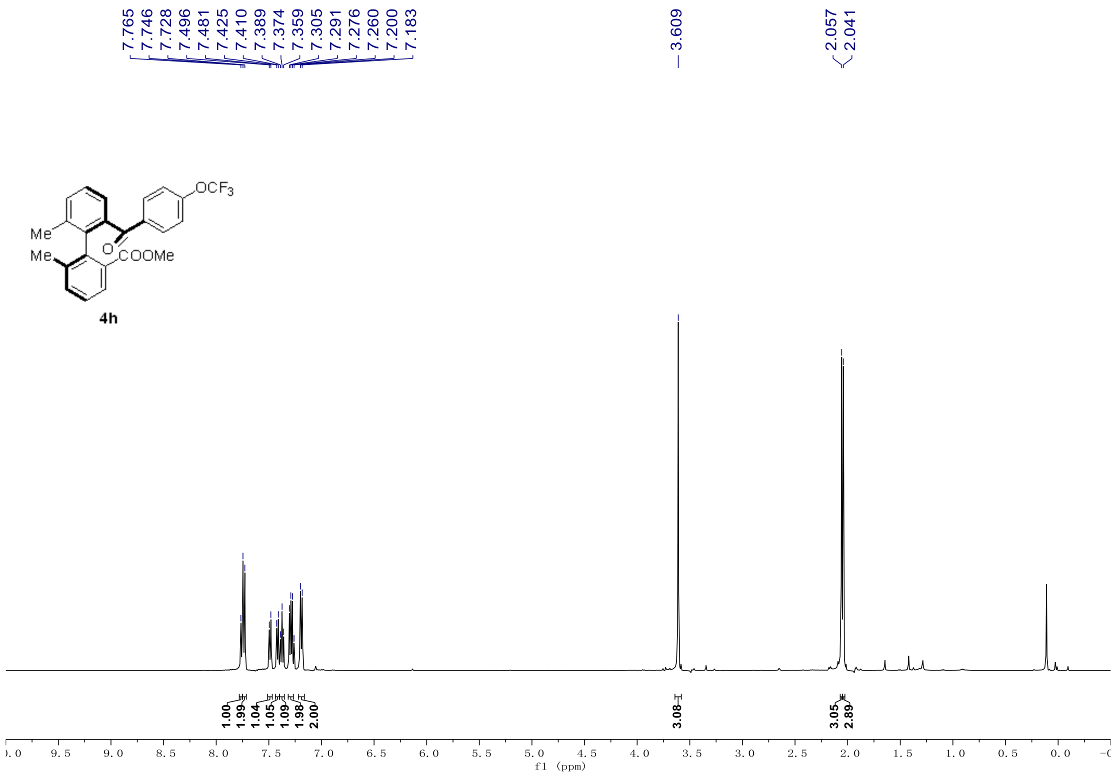
7.765
7.746
7.728
7.496
7.481
7.425
7.410
7.389
7.374
7.359
7.305
7.291
7.276
7.260
7.200
7.183
3.609
2.057
2.041
OCF3
Me
Me
O
COOMe
4h
1.00
1.99
1.04
1.05
1.09
1.98
2.00
3.08
3.05
2.89
f1 (ppm)

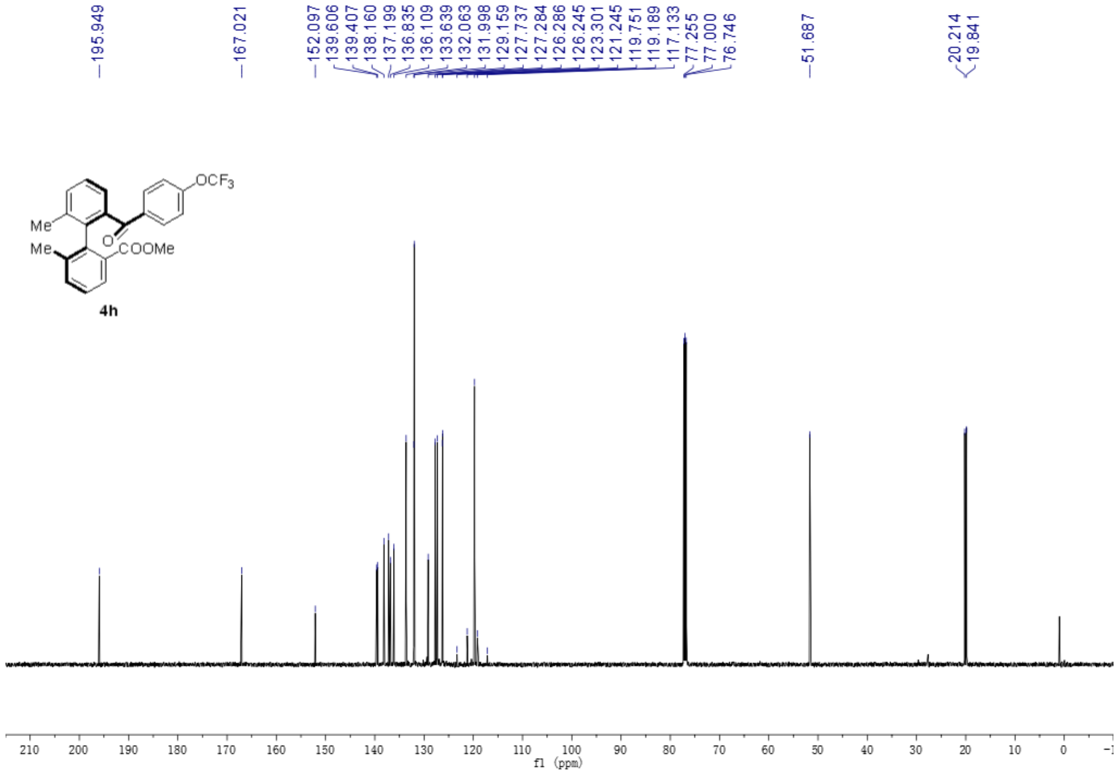
195.949
167.021
152.097
139.606
139.407
138.160
137.199
136.835
136.109
133.639
132.063
131.998
129.159
127.737
127.284
126.286
126.245
123.301
121.245
119.751
119.189
117.133
77.255
77.000
76.746
51.687
20.214
19.841
OCF3
Me
Me
O
COOMe
4h
f1 (ppm)

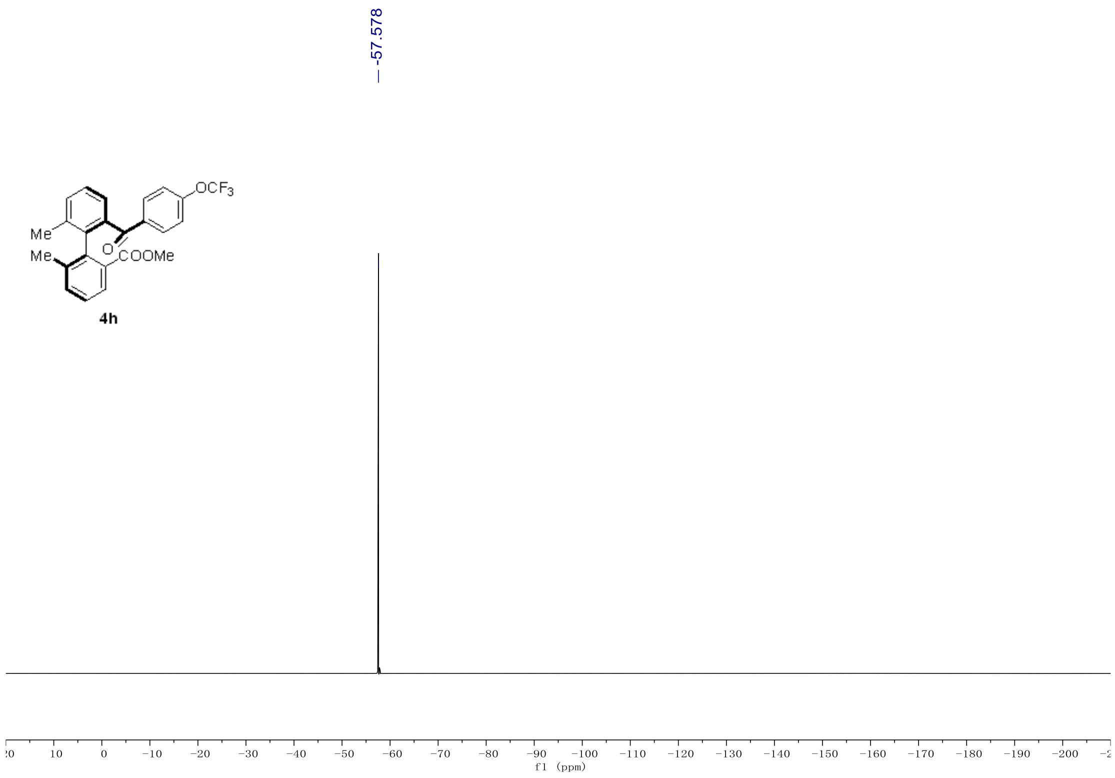
-57.578
OCF3
Me
Me
O
COOMe
4h
f1 (ppm)

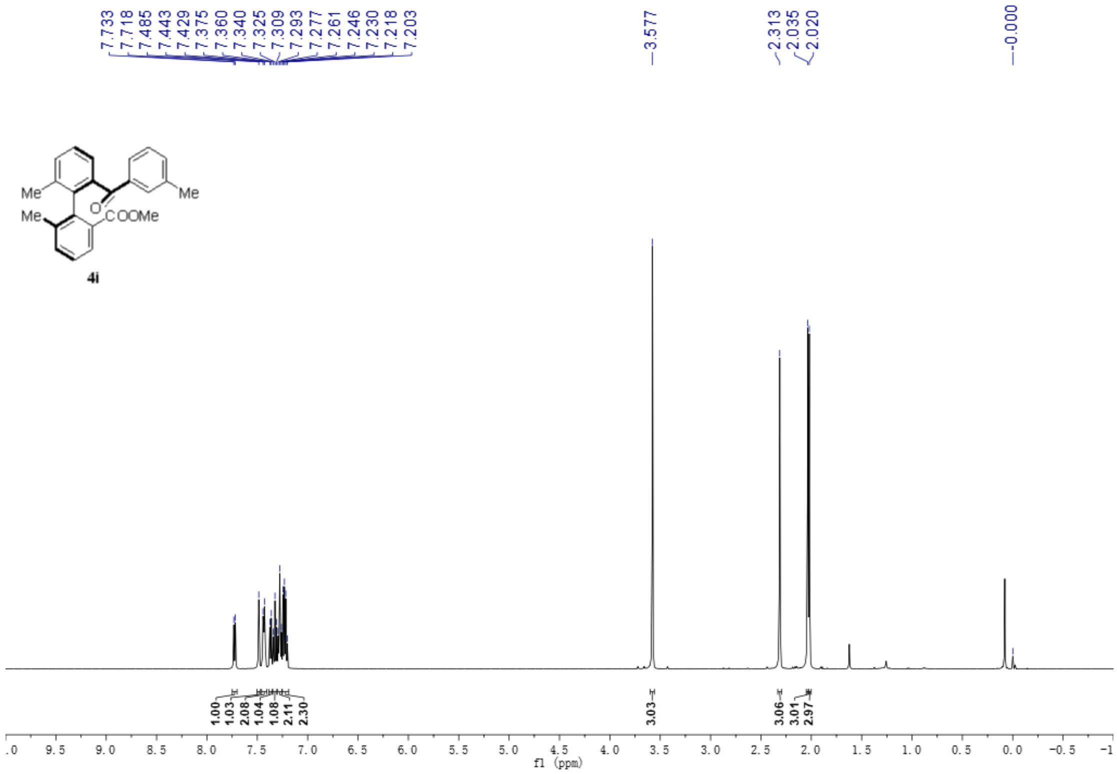
7.733
7.718
7.485
7.443
7.429
7.375
7.360
7.340
7.325
7.309
7.293
7.277
7.261
7.246
7.230
7.218
7.203
3.577
2.313
2.035
2.020
0.000
Me
Me
Me
O
COOMe
4i
1.00
1.03
2.08
1.04
1.08
2.11
2.30
3.03
3.06
3.01
2.97
f1 (ppm)

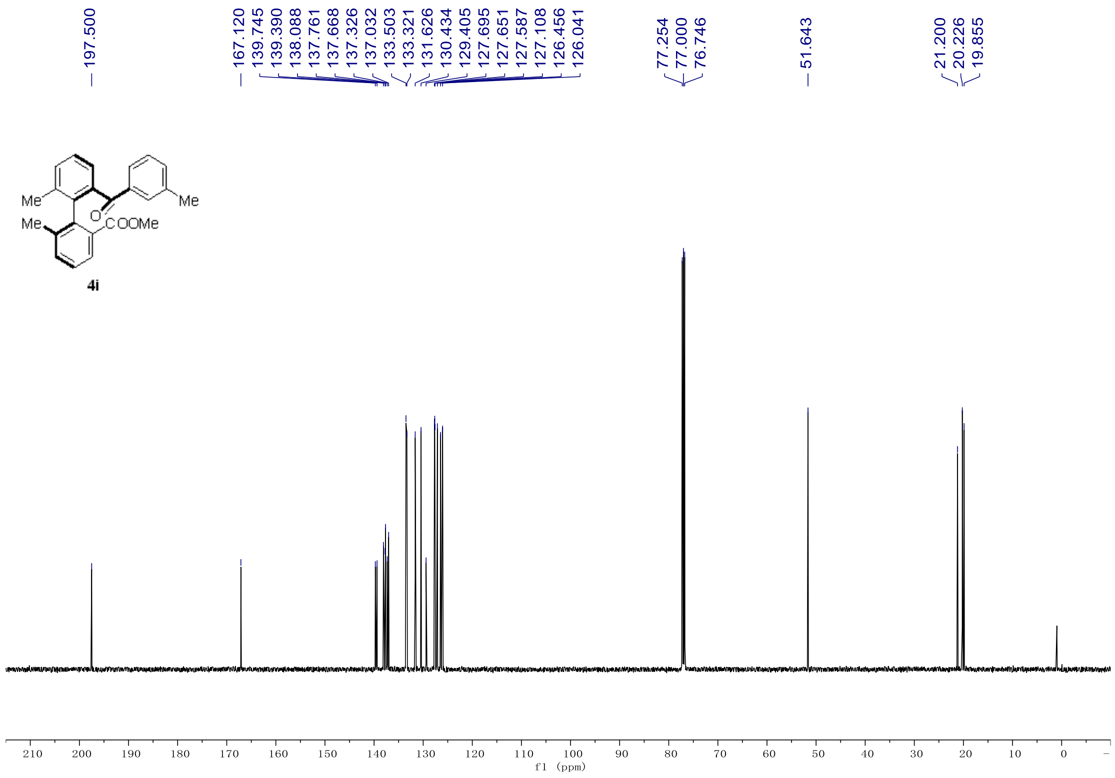
197.500
167.120
139.745
139.390
138.088
137.761
137.668
137.326
137.032
133.503
133.321
131.626
130.434
129.405
127.695
127.651
127.587
127.108
126.456
126.041
77.254
77.000
76.746
51.643
21.200
20.226
19.855
Me
Me
O
COOMe
Me
4i
f1 (ppm)

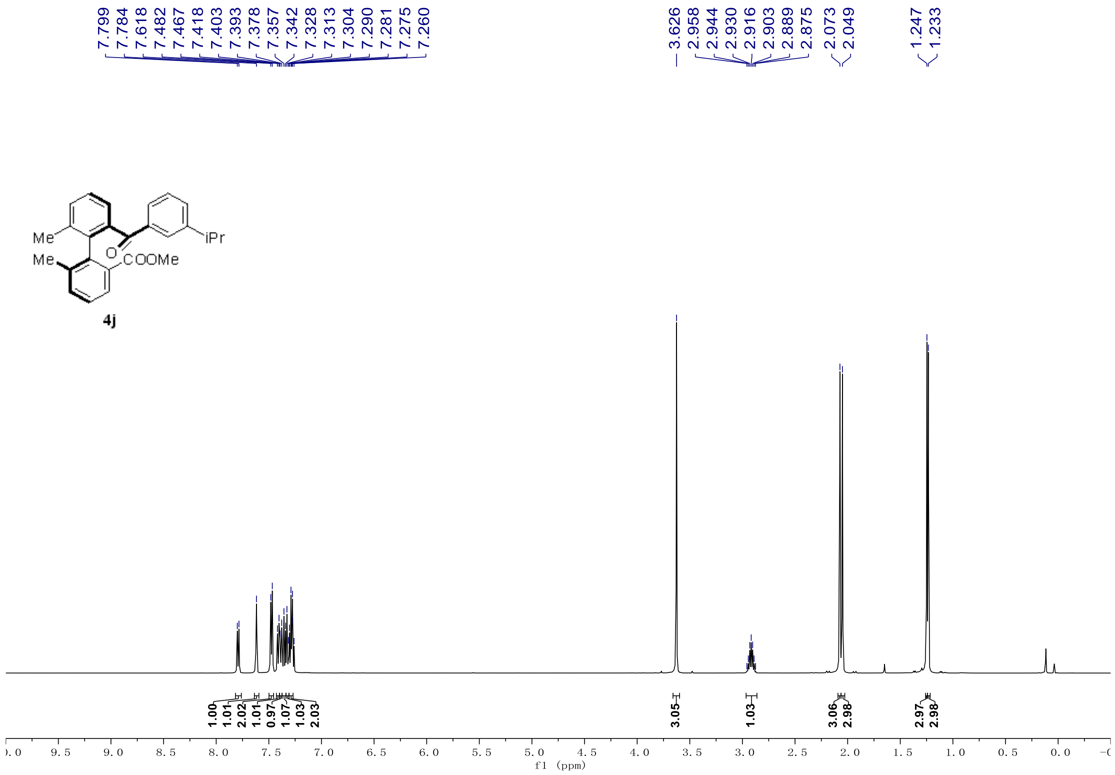
7.799
7.784
7.618
7.482
7.467
7.418
7.403
7.393
7.378
7.357
7.342
7.328
7.313
7.304
7.290
7.281
7.275
7.260
3.626
2.958
2.944
2.930
2.916
2.903
2.889
2.875
2.073
2.049
1.247
1.233
Me
Me
O
COOMe
iPr
4j
1.00
1.01
2.02
1.01
0.97
1.07
1.03
2.03
3.05
1.03
3.06
2.98
2.97
2.98
f1 (ppm)

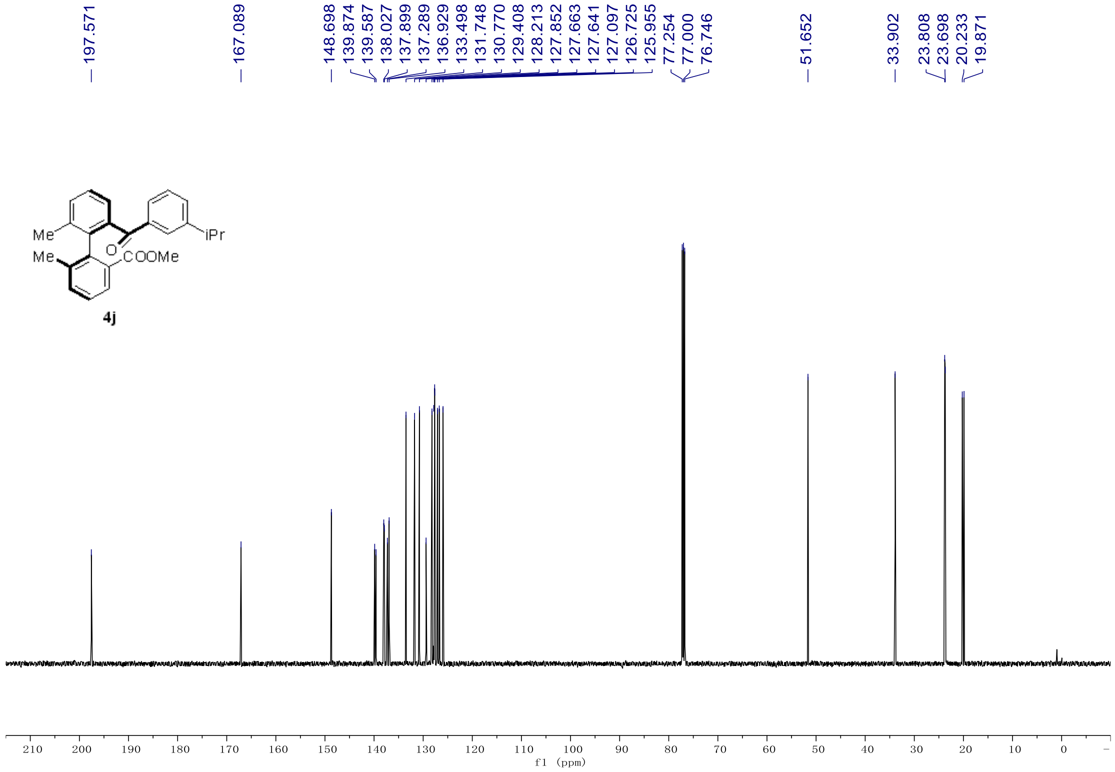
197.571
167.089
148.698
139.874
139.587
138.027
137.899
137.289
136.929
133.498
131.748
130.770
129.408
128.213
127.852
127.663
127.641
127.097
126.725
125.955
77.254
77.000
76.746
51.652
33.902
23.808
23.698
20.233
19.871
Me
Me
O
COOMe
iPr
4j
210 200 190 180 170 160 150 140 130 120 110 100 90 80 70 60 50 40 30 20 10 0
f1 (ppm)

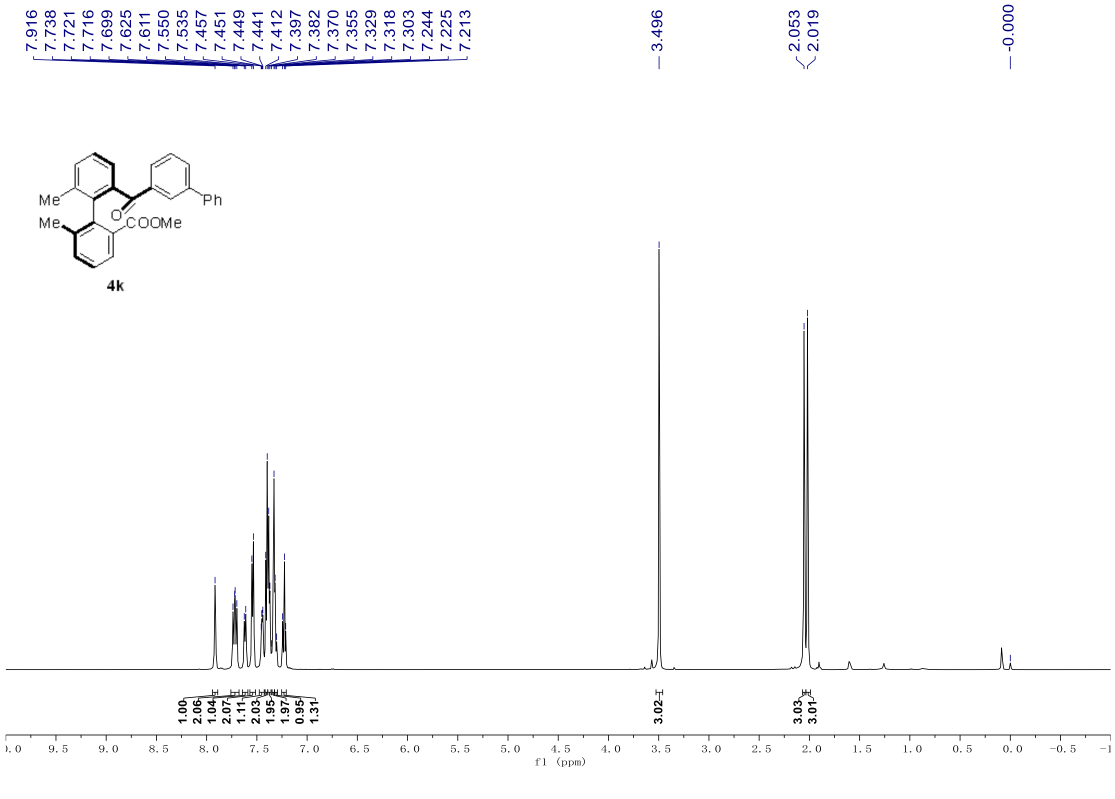
7.916
7.738
7.721
7.716
7.699
7.625
7.611
7.550
7.535
7.457
7.451
7.449
7.441
7.412
7.397
7.382
7.370
7.355
7.329
7.318
7.303
7.244
7.225
7.213
3.496
2.053
2.019
-0.000
Me
Me
O
COOMe
Ph
4k
1.00
2.06
1.04
2.07
1.11
2.03
1.95
1.97
0.95
1.31
3.02
3.03
3.01
9.5 9.0 8.5 8.0 7.5 7.0 6.5 6.0 5.5 5.0 4.5 4.0 3.5 3.0 2.5 2.0 1.5 1.0 0.5 0.0 -0.5
f1 (ppm)

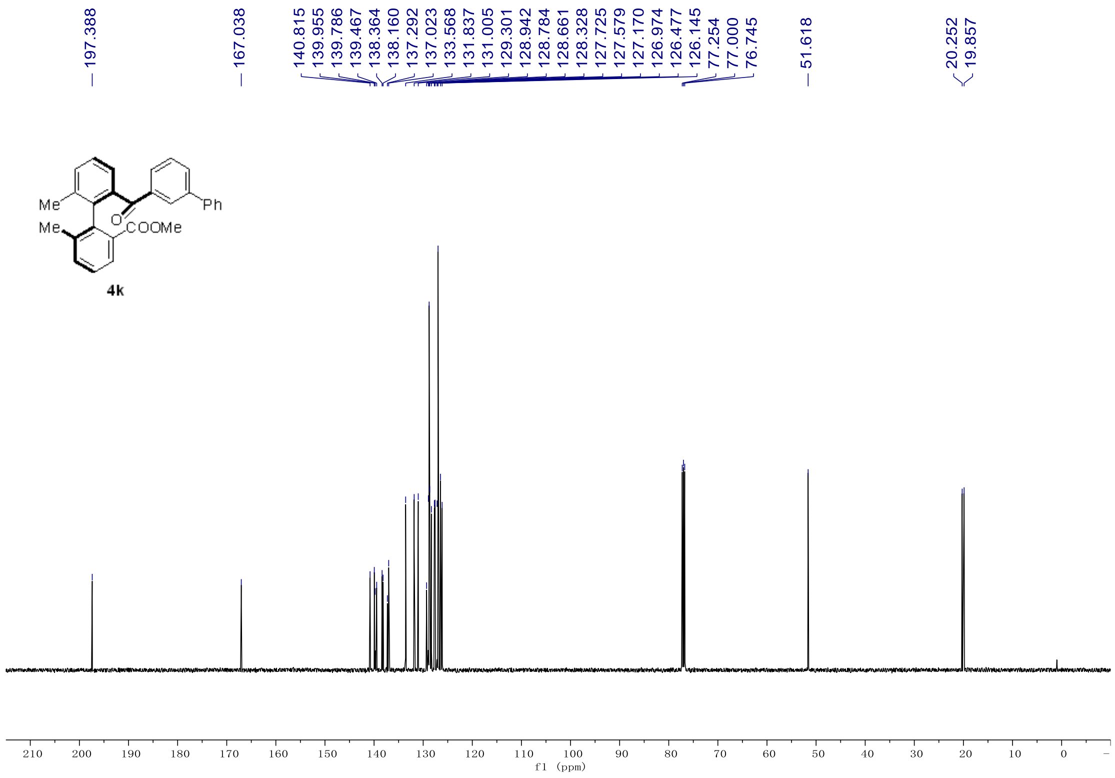
197.388
167.038
140.815
139.955
139.786
139.467
138.364
138.160
137.292
137.023
133.568
131.837
131.005
129.301
128.942
128.784
128.661
128.328
127.725
127.579
127.170
126.974
126.477
126.145
77.254
77.000
76.745
51.618
20.252
19.857
Me
Me
O
COOMe
Ph
4k
f1 (ppm)

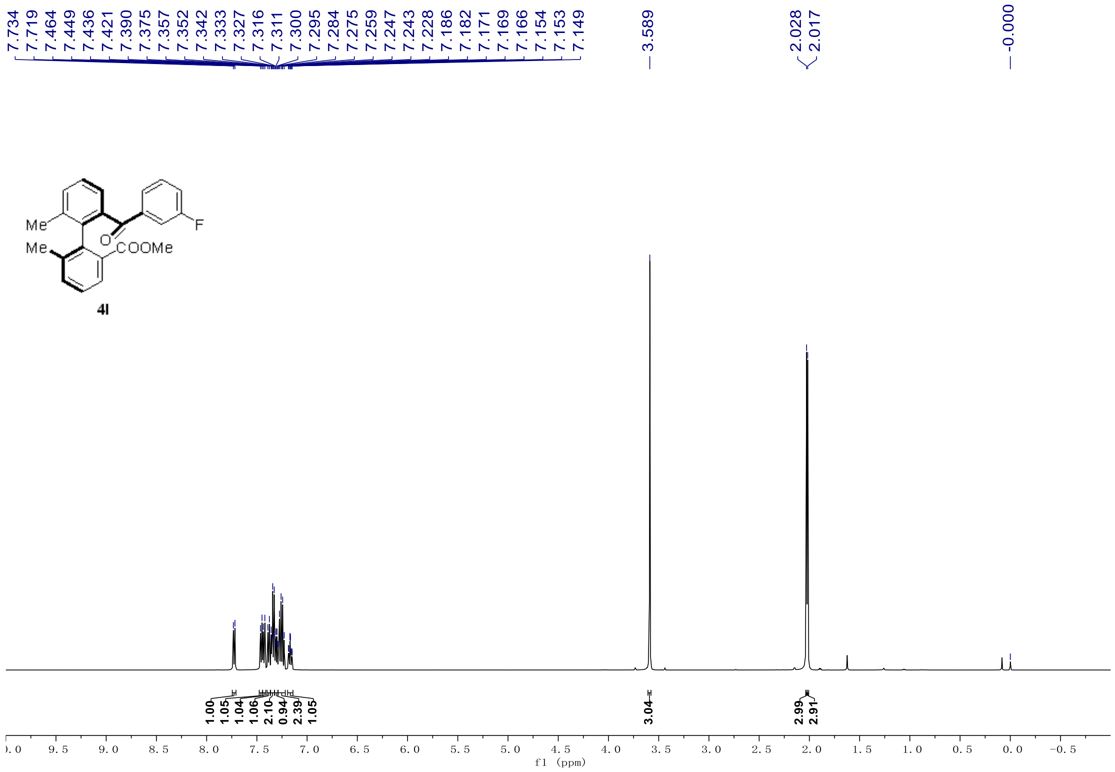
7.734
7.719
7.464
7.449
7.436
7.421
7.390
7.375
7.357
7.352
7.342
7.333
7.327
7.316
7.311
7.300
7.295
7.284
7.275
7.259
7.247
7.243
7.228
7.186
7.182
7.171
7.169
7.166
7.154
7.153
7.149
3.589
2.028
2.017
-0.000
Me
Me
O
COOMe
F
4l
1.00
1.05
1.04
1.06
2.10
0.94
2.39
1.05
3.04
2.99
2.91
f1 (ppm)

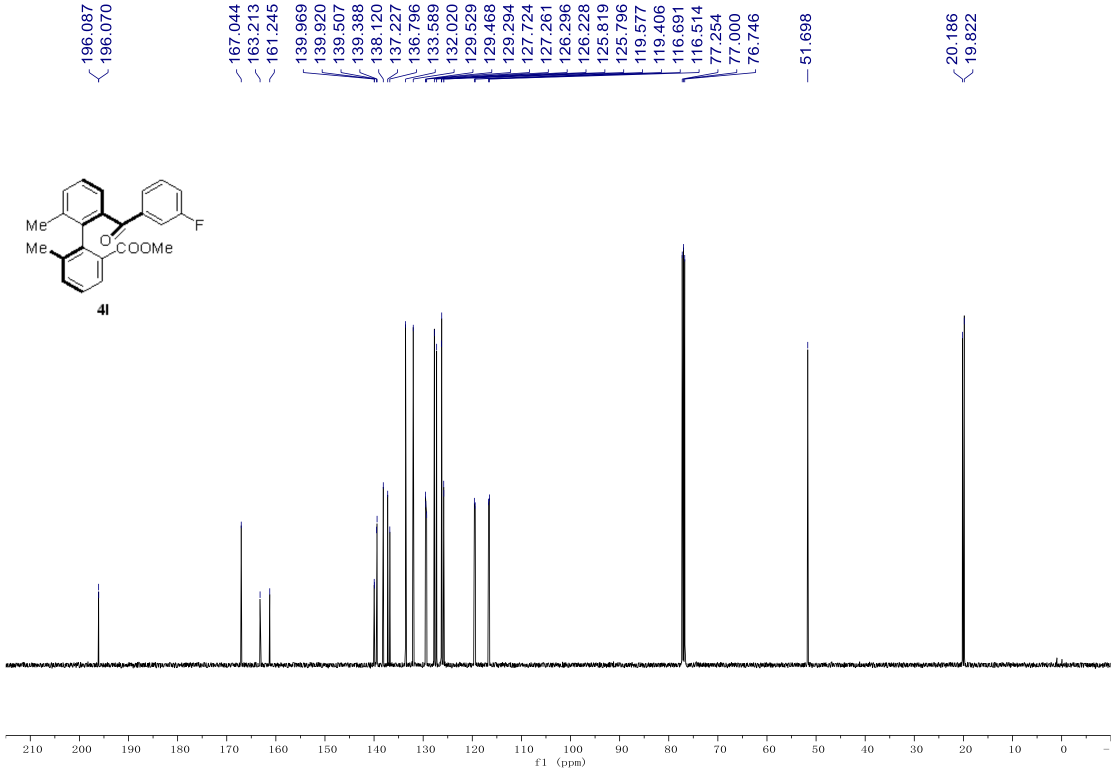
196.087
196.070
167.044
163.213
161.245
139.969
139.920
139.507
139.388
138.120
137.227
136.796
133.589
132.020
129.529
129.468
129.294
127.724
127.261
126.296
126.228
125.819
125.796
119.577
119.406
116.691
116.514
77.254
77.000
76.746
51.698
20.186
19.822
Me
Me
O
COOMe
F
4l
f1 (ppm)

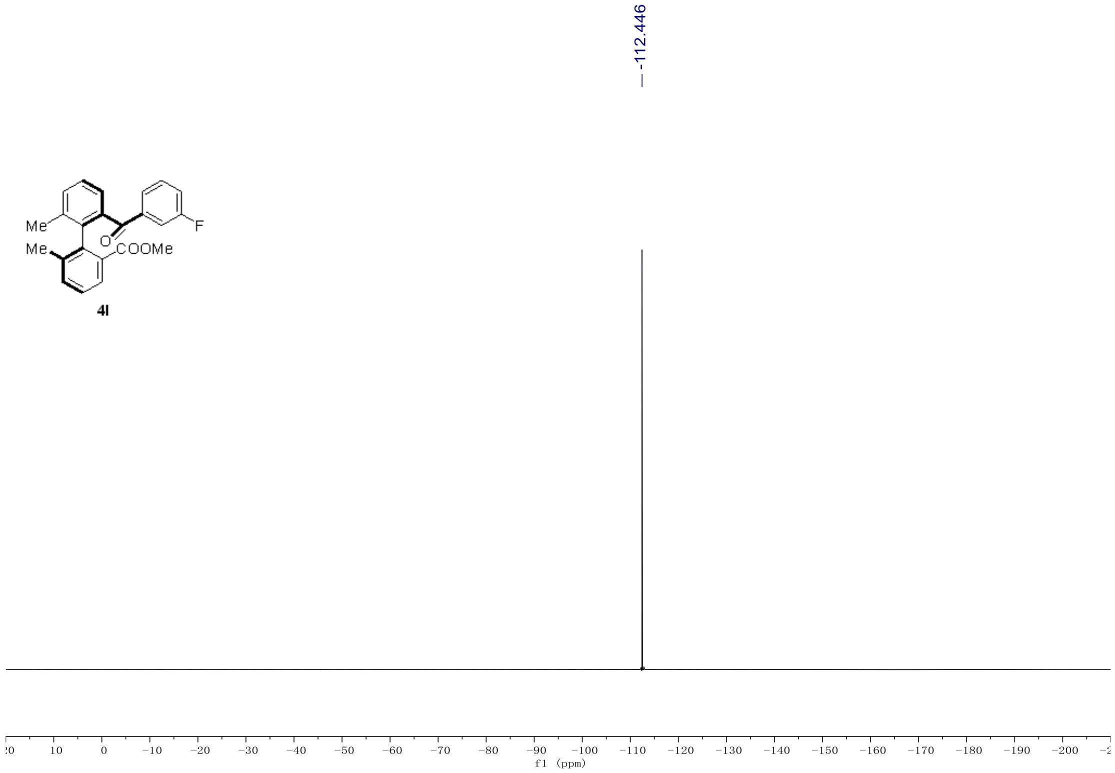
-112.446
Me
Me
O
COOMe
F
4l
f1 (ppm)

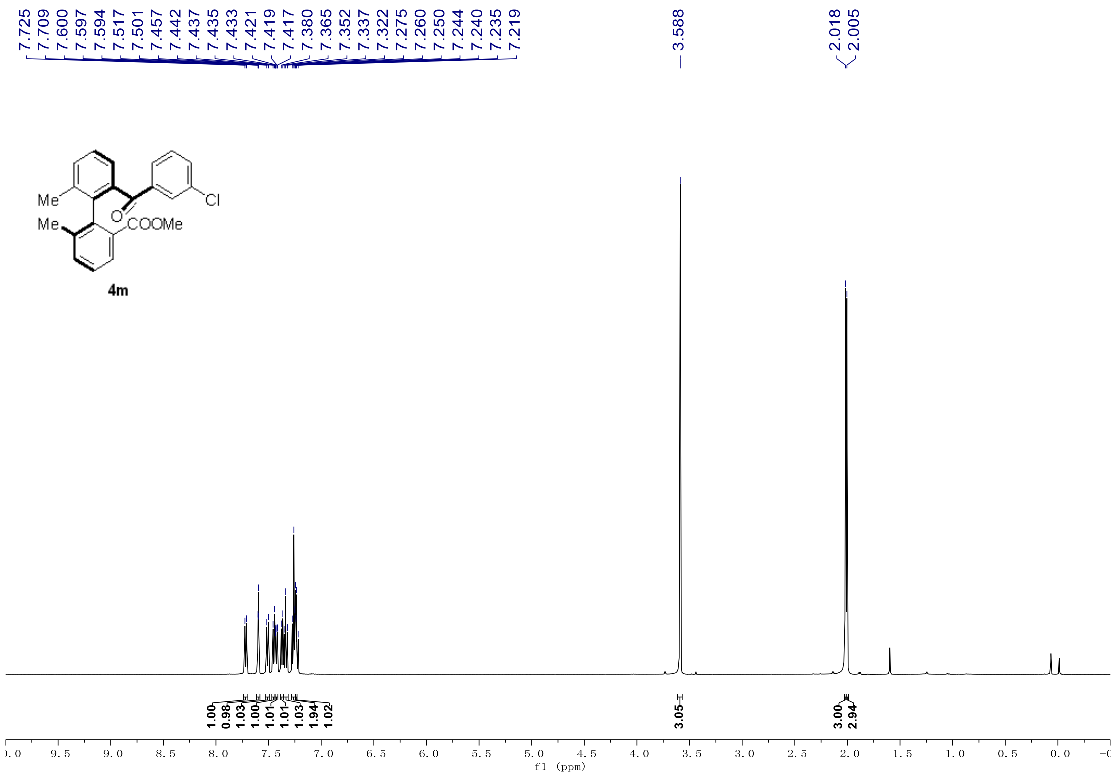

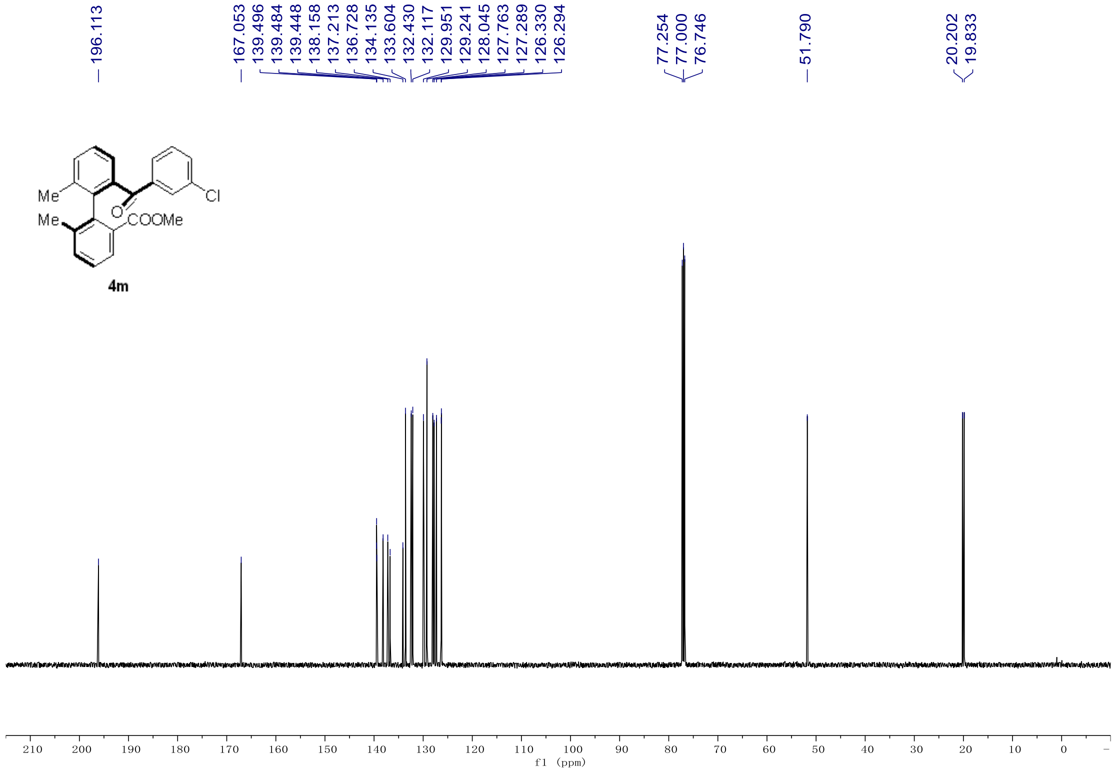

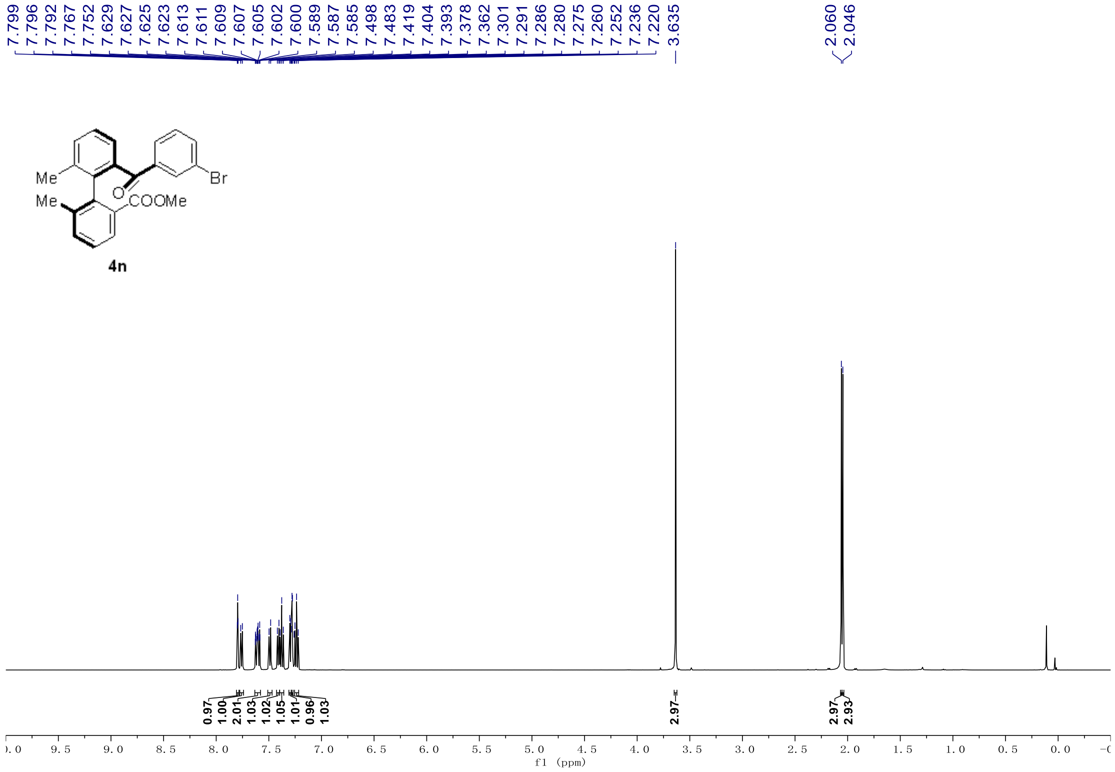
7.799
7.796
7.792
7.767
7.752
7.629
7.627
7.625
7.623
7.613
7.611
7.609
7.607
7.605
7.602
7.600
7.589
7.587
7.585
7.498
7.483
7.419
7.404
7.393
7.378
7.362
7.301
7.291
7.286
7.280
7.275
7.260
7.252
7.236
7.220
3.635
2.060
2.046
Me
Me
O
COOMe
Br
4n
0.97
1.00
2.01
1.03
1.02
1.05
1.01
0.96
1.03
2.97
2.97
2.93
f1 (ppm)

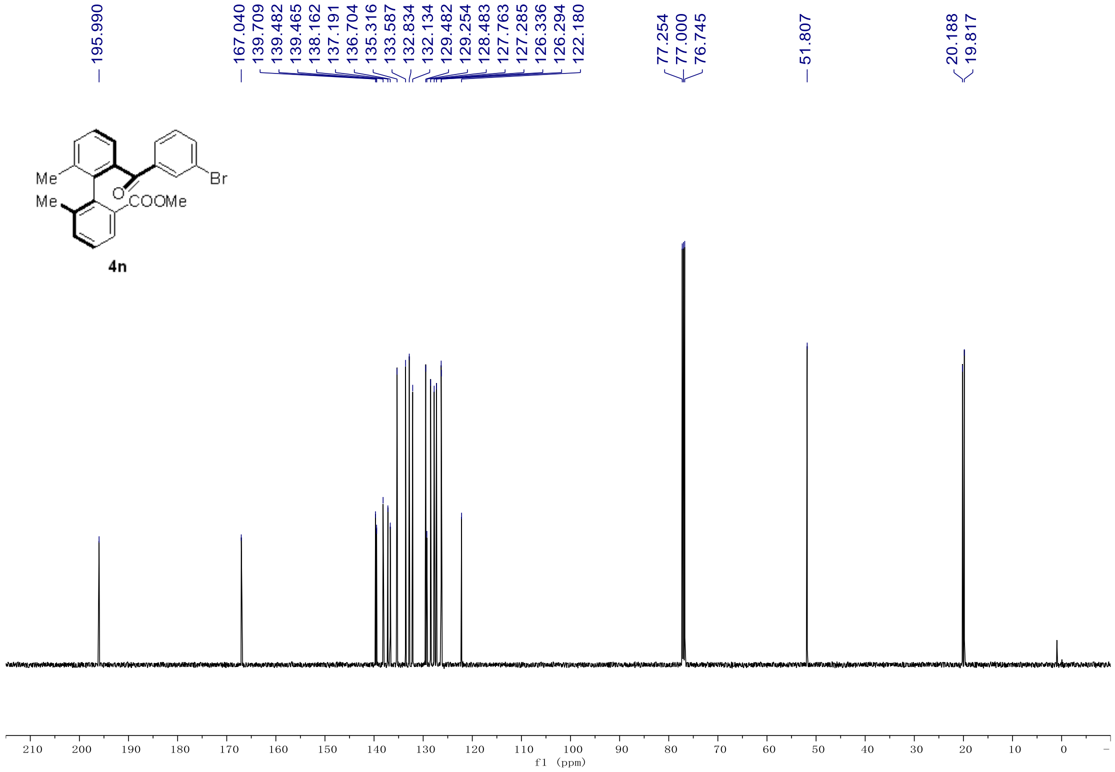
195.990
167.040
139.709
139.482
139.465
138.162
137.191
136.704
135.316
133.587
132.834
132.134
129.482
129.254
128.483
127.763
127.285
126.336
126.294
122.180
77.254
77.000
76.745
51.807
20.188
19.817
Me
Me
O
COOMe
Br
4n
f1 (ppm)

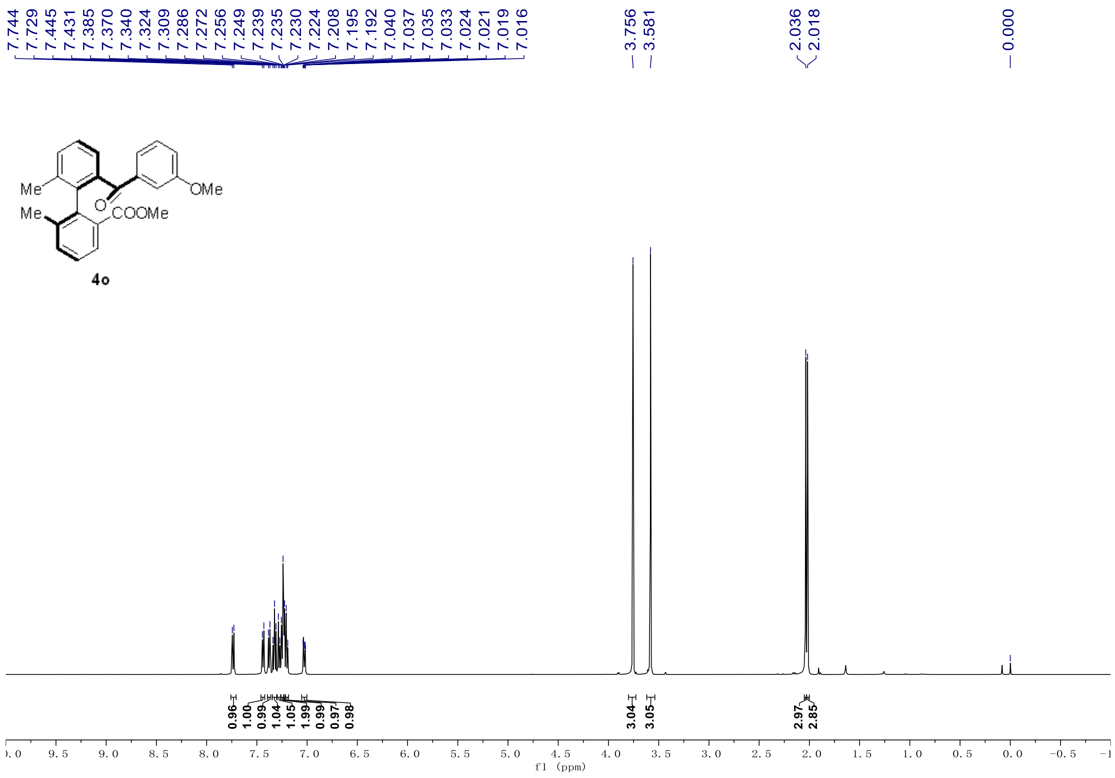

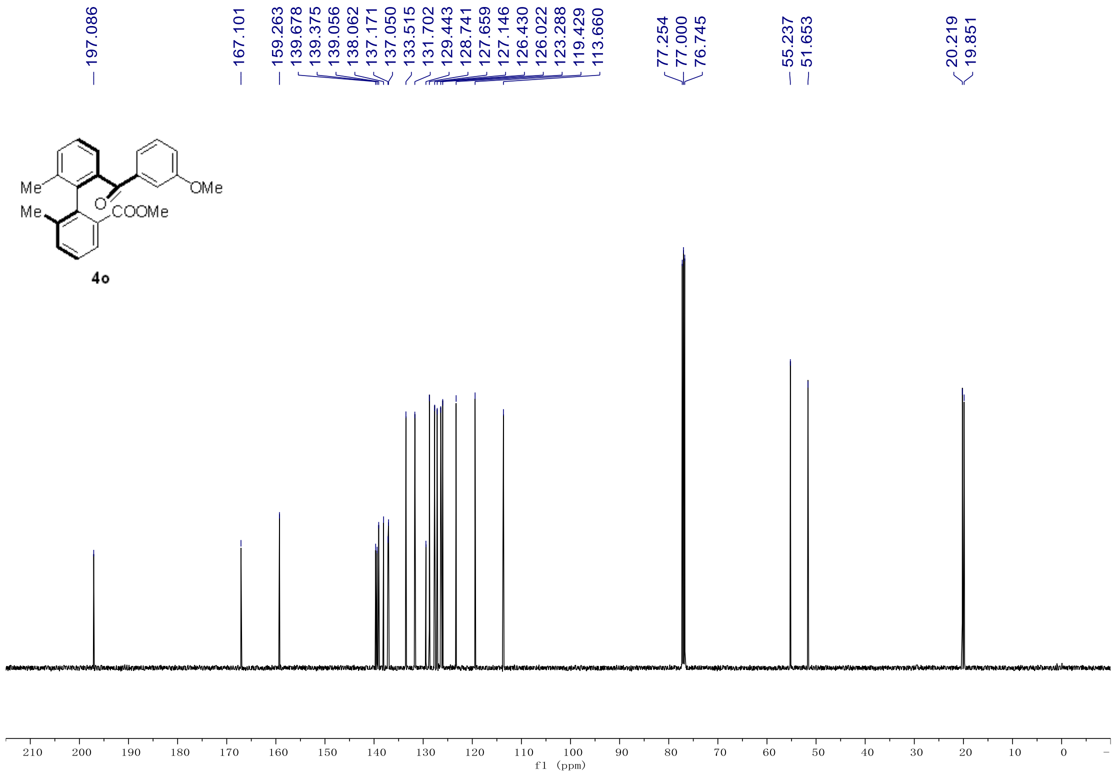

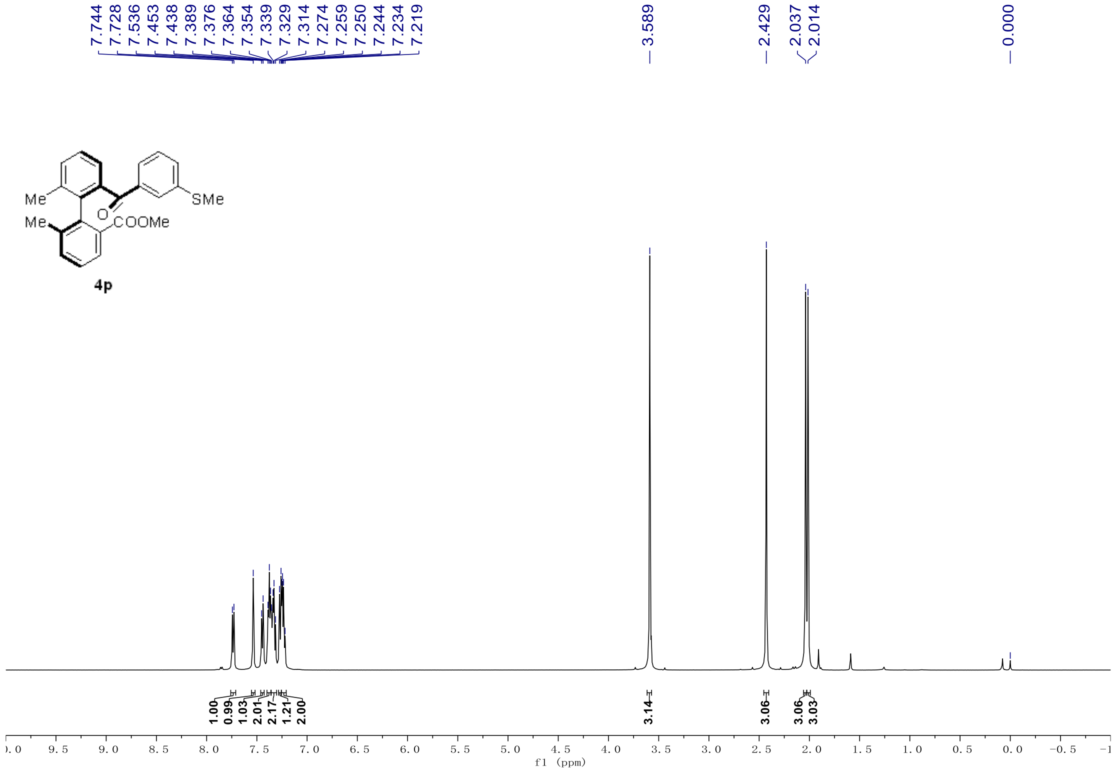
7.744
7.728
7.536
7.453
7.438
7.389
7.376
7.364
7.354
7.339
7.329
7.314
7.274
7.259
7.250
7.244
7.234
7.219
3.589
2.429
2.037
2.014
0.000
Me
Me
SMe
O
COOMe
4p
1.00
0.99
1.03
2.01
2.17
1.21
2.00
3.14
3.06
3.06
3.03
f1 (ppm)

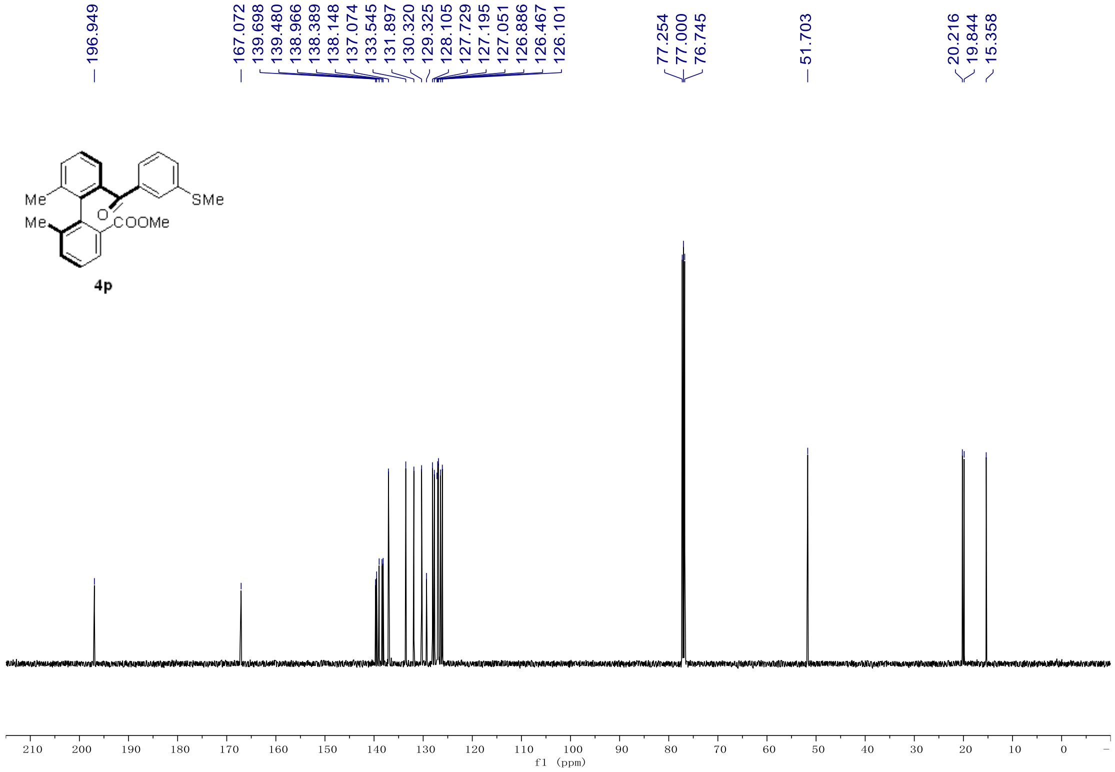
196.949
167.072
139.698
139.480
138.966
138.389
138.148
137.074
133.545
131.897
130.320
129.325
128.105
127.729
127.195
127.051
126.886
126.467
126.101
77.254
77.000
76.745
51.703
20.216
19.844
15.358
Me
Me
SMe
O
COOMe
4p
f1 (ppm)

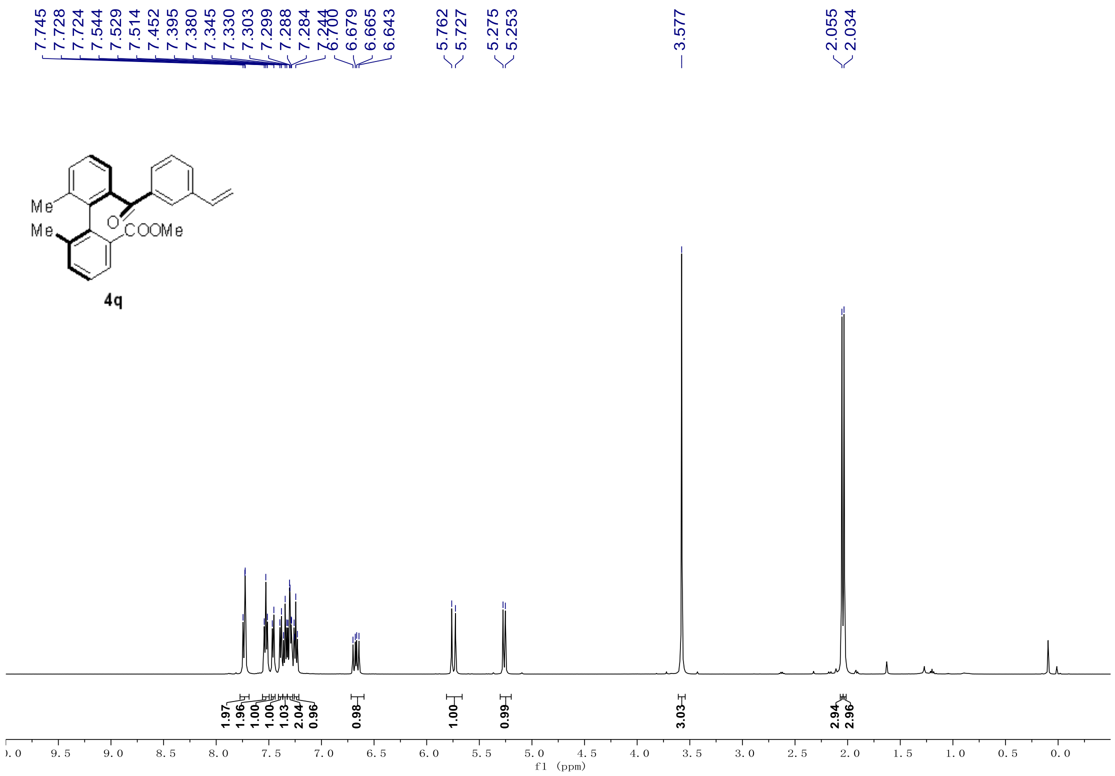
7.745
7.728
7.724
7.544
7.529
7.514
7.452
7.395
7.380
7.345
7.330
7.303
7.299
7.288
7.284
7.244
6.700
6.679
6.665
6.643
5.762
5.727
5.275
5.253
3.577
2.055
2.034
Me
Me
O
COOMe
4q
1.97
1.96
1.00
1.00
1.03
2.04
0.96
0.98
1.00
0.99
3.03
2.94
2.96
f1 (ppm)

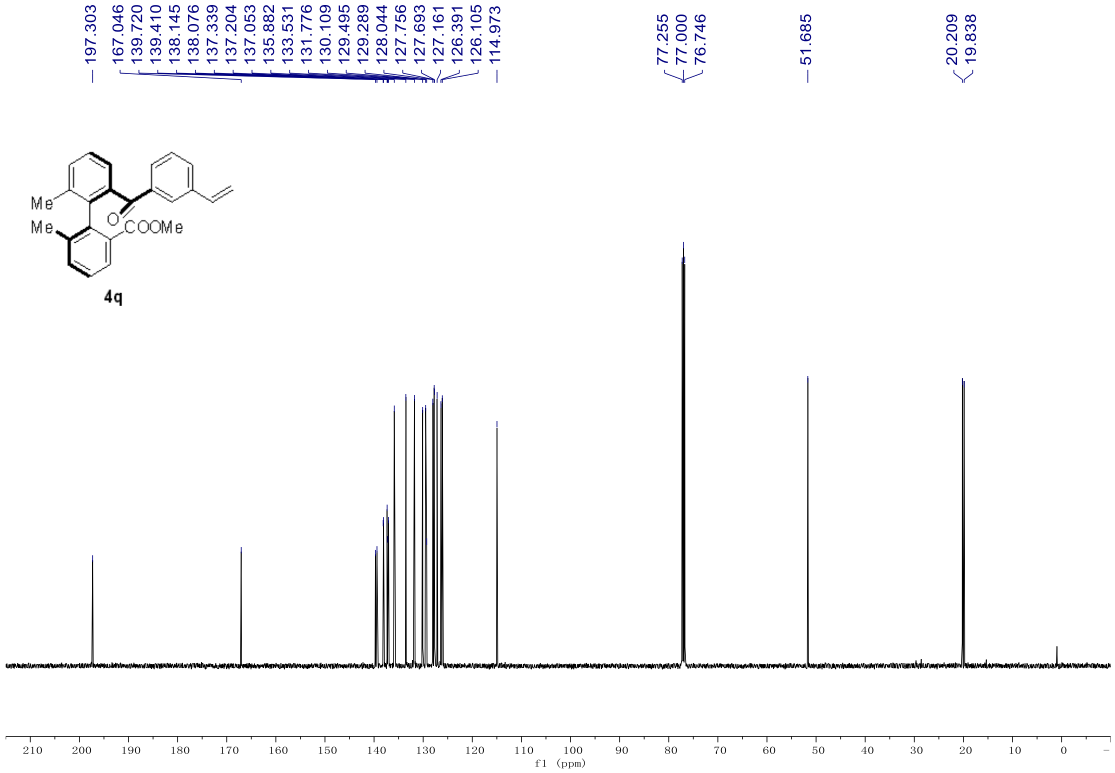
197.303
167.046
139.720
139.410
138.145
138.076
137.339
137.204
137.053
135.882
133.531
131.776
130.109
129.495
129.289
128.044
127.756
127.693
127.161
126.391
126.105
114.973
77.255
77.000
76.746
51.685
20.209
19.838
Me
Me
O
COOMe
4q
f1 (ppm)

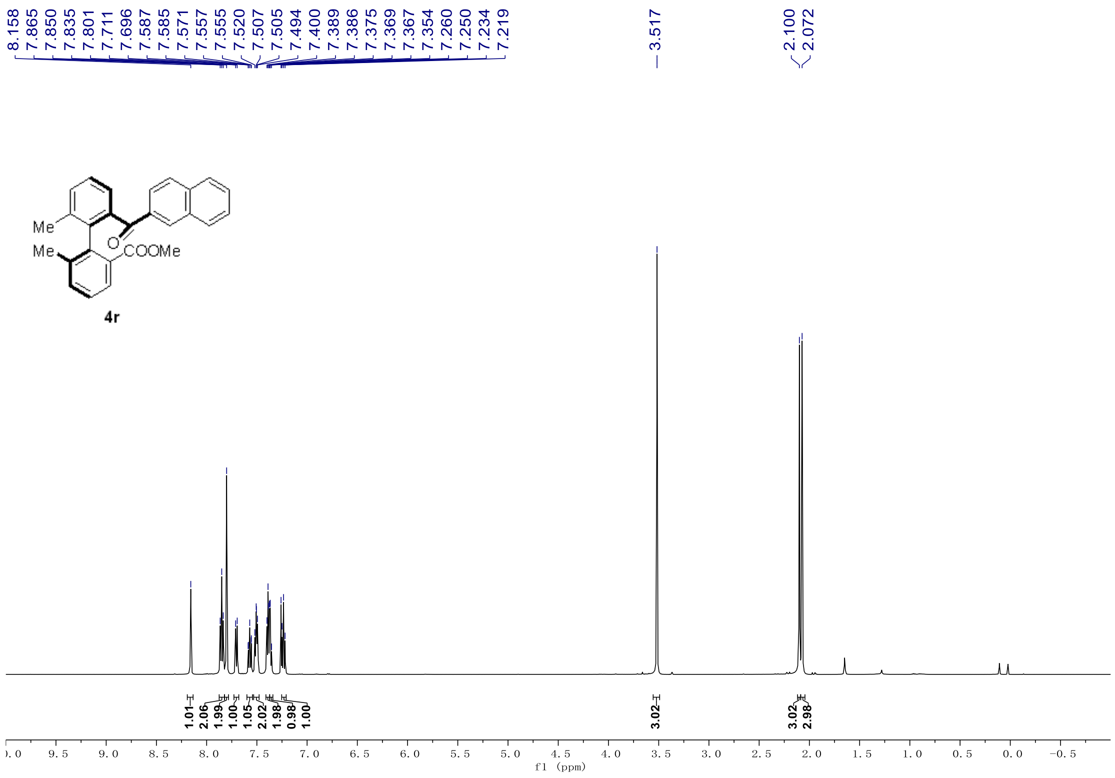
8.158
7.865
7.850
7.835
7.801
7.711
7.696
7.587
7.585
7.571
7.557
7.555
7.520
7.507
7.505
7.494
7.400
7.389
7.386
7.375
7.369
7.367
7.354
7.260
7.250
7.234
7.219
3.517
2.100
2.072
Me
Me
O
COOMe
4r
1.01
2.06
1.99
1.00
1.05
2.02
1.98
0.98
1.00
3.02
3.02
2.98
f1 (ppm)

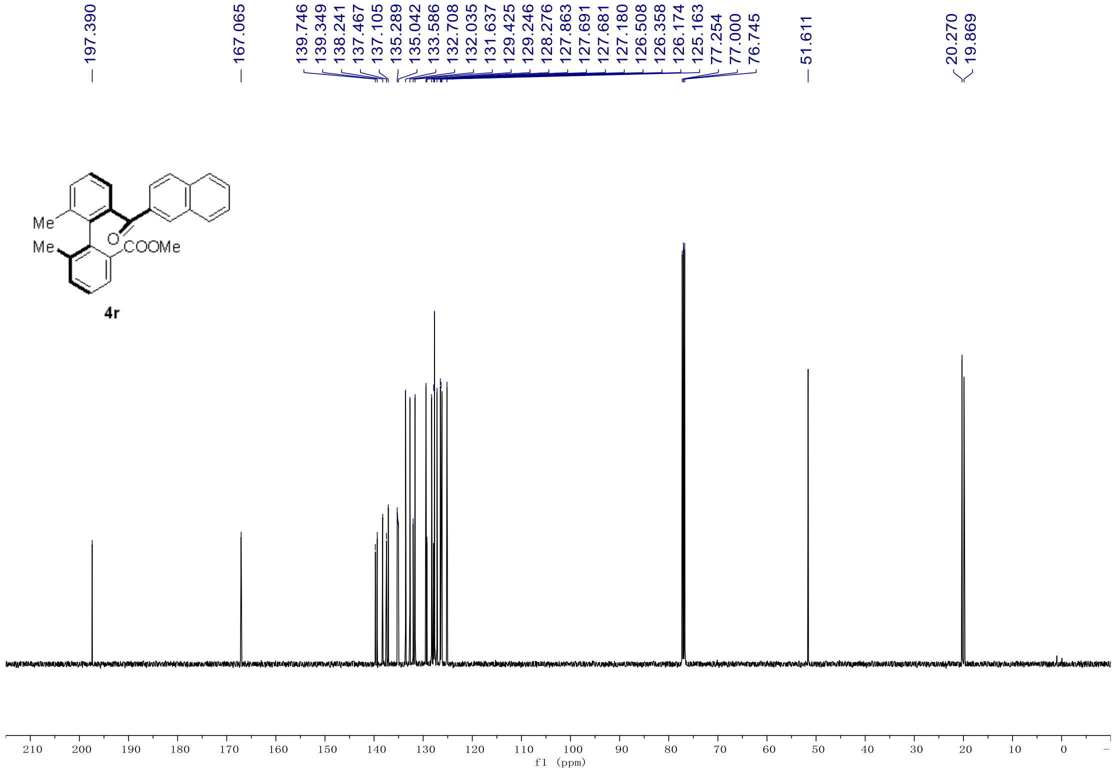
197.390
167.065
139.746
139.349
138.241
137.467
137.105
135.289
135.042
133.586
132.708
132.035
131.637
129.425
129.246
128.276
127.863
127.691
127.681
127.180
126.508
126.358
126.174
125.163
77.254
77.000
76.745
51.611
20.270
19.869
Me
Me
O
COOMe
4r
f1 (ppm)

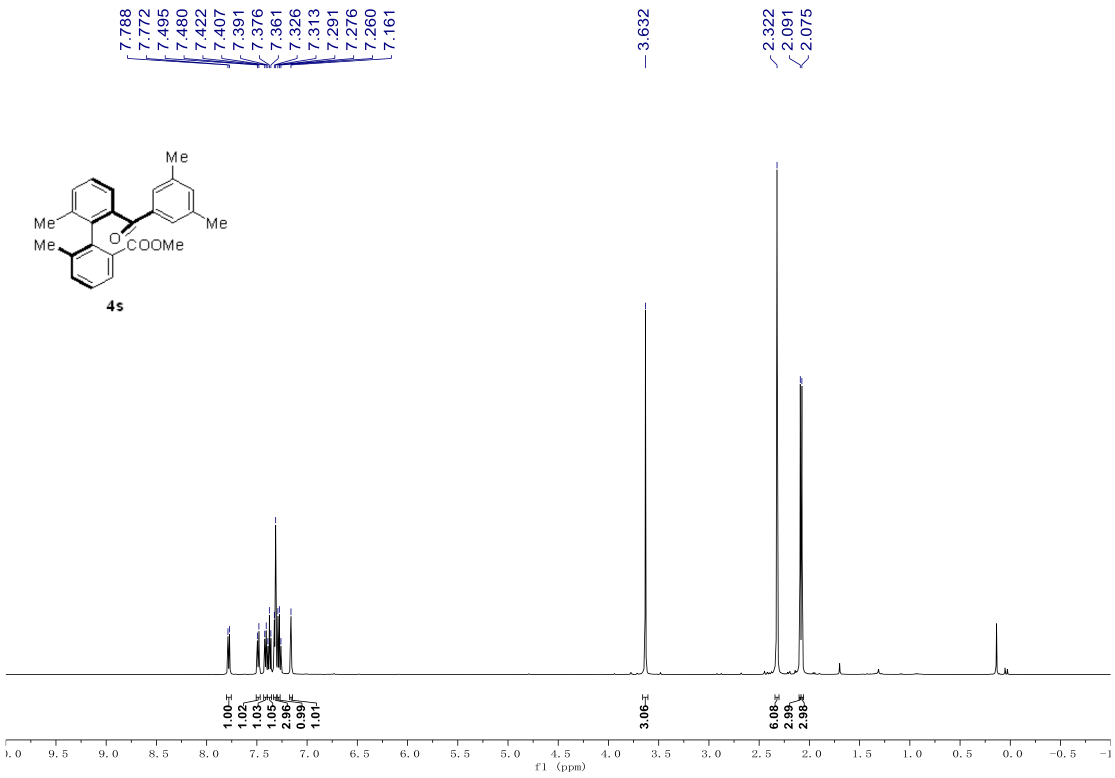
7.788
7.772
7.495
7.480
7.422
7.407
7.391
7.376
7.361
7.326
7.313
7.291
7.276
7.260
7.161
3.632
2.322
2.091
2.075
Me
Me
Me
Me
O
COOMe
4s
1.00
1.02
1.03
1.05
2.96
0.99
1.01
3.06
6.08
2.99
2.98
f1 (ppm)

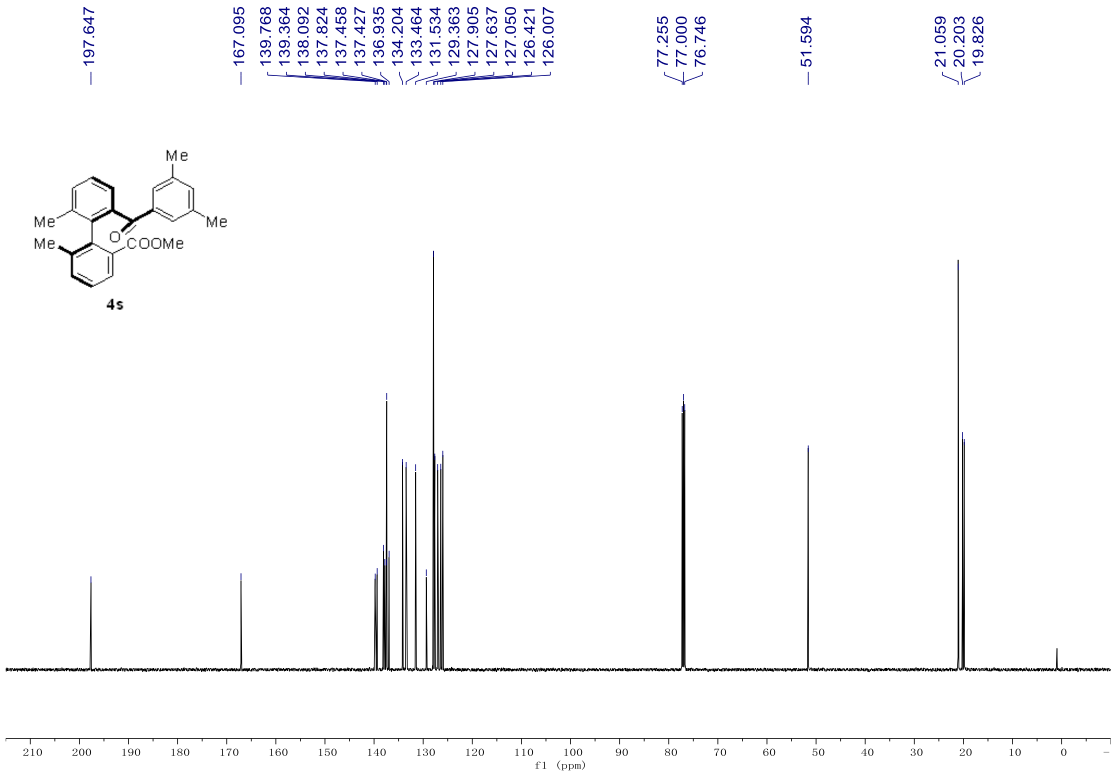
197.647
167.095
139.768
139.364
138.092
137.824
137.458
137.427
136.935
134.204
133.464
131.534
129.363
127.905
127.637
127.050
126.421
126.007
77.255
77.000
76.746
51.594
21.059
20.203
19.826
Me
Me
Me
Me
O
COOMe
4s
f1 (ppm)

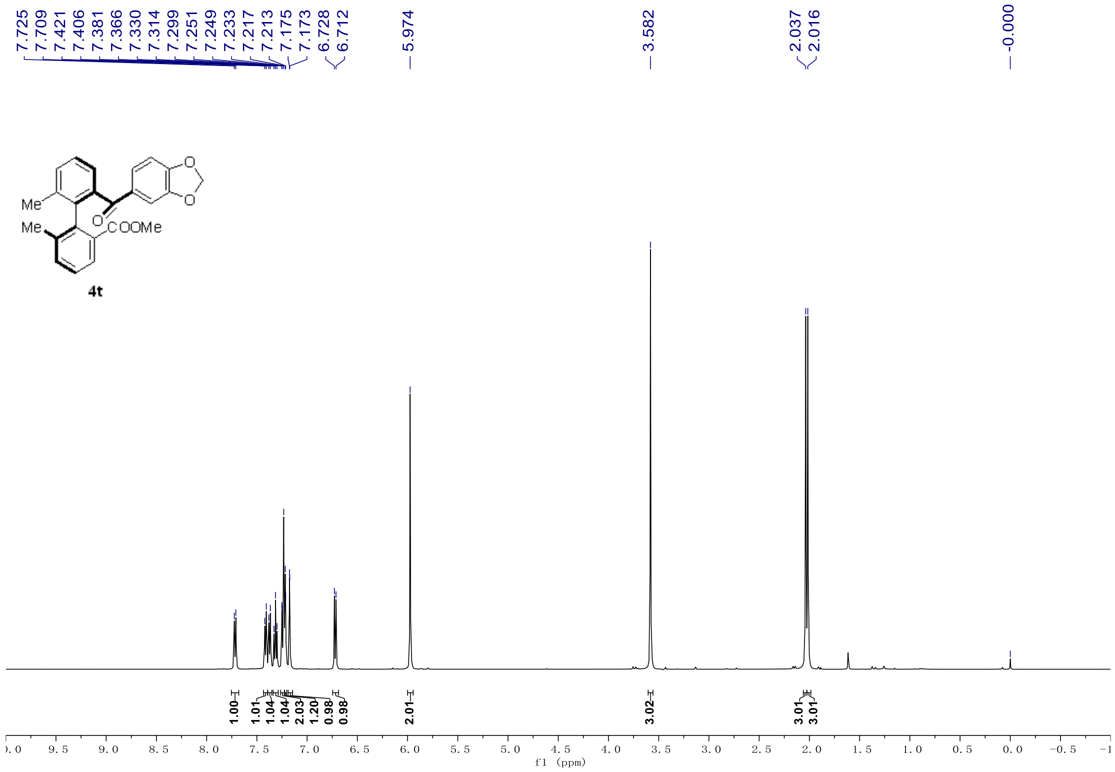
7.725
7.709
7.421
7.406
7.381
7.366
7.330
7.314
7.299
7.251
7.249
7.233
7.217
7.213
7.175
7.173
6.728
6.712
5.974
3.582
2.037
2.016
-0.000
Me
Me
O
COOMe
4t
1.00
1.01
1.04
1.04
2.03
1.20
0.98
0.98
2.01
3.02
3.01
3.01
f1 (ppm)

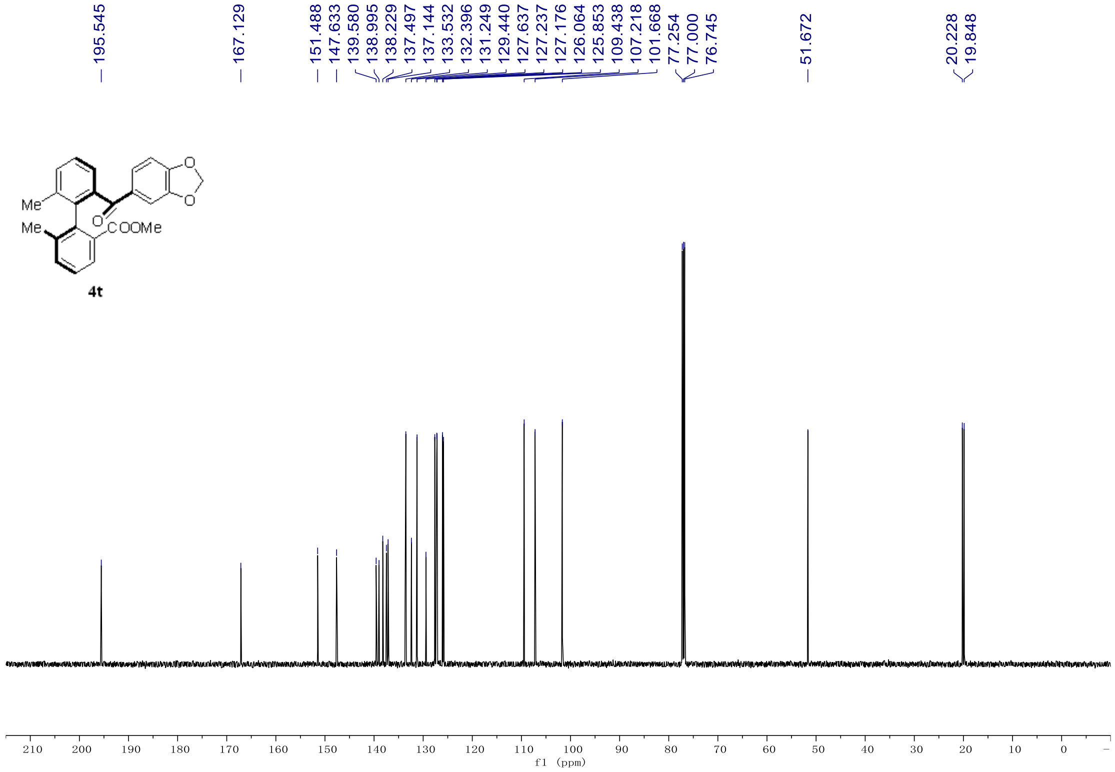
195.545
167.129
151.488
147.633
139.580
138.995
138.229
137.497
137.144
133.532
132.396
131.249
129.440
127.637
127.237
127.176
126.064
125.853
109.438
107.218
101.668
77.254
77.000
76.745
51.672
20.228
19.848
Me
Me
O
COOMe
4t
f1 (ppm)

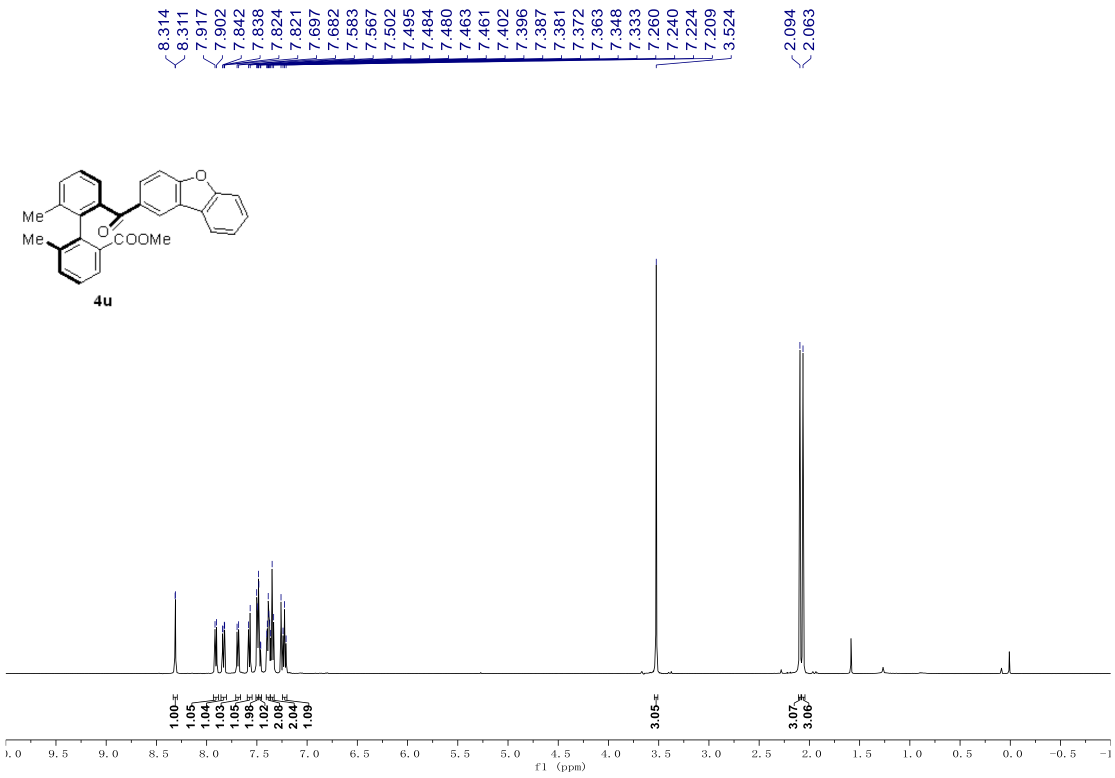
8.314
8.311
7.917
7.902
7.842
7.838
7.824
7.821
7.697
7.682
7.583
7.567
7.502
7.495
7.484
7.480
7.463
7.461
7.402
7.396
7.387
7.381
7.372
7.363
7.348
7.333
7.260
7.240
7.224
7.209
3.524
2.094
2.063
Me
Me
O
COOMe
O
4u
1.00
1.05
1.04
1.03
1.05
1.98
1.02
2.08
2.04
1.09
3.05
3.07
3.06
f1 (ppm)

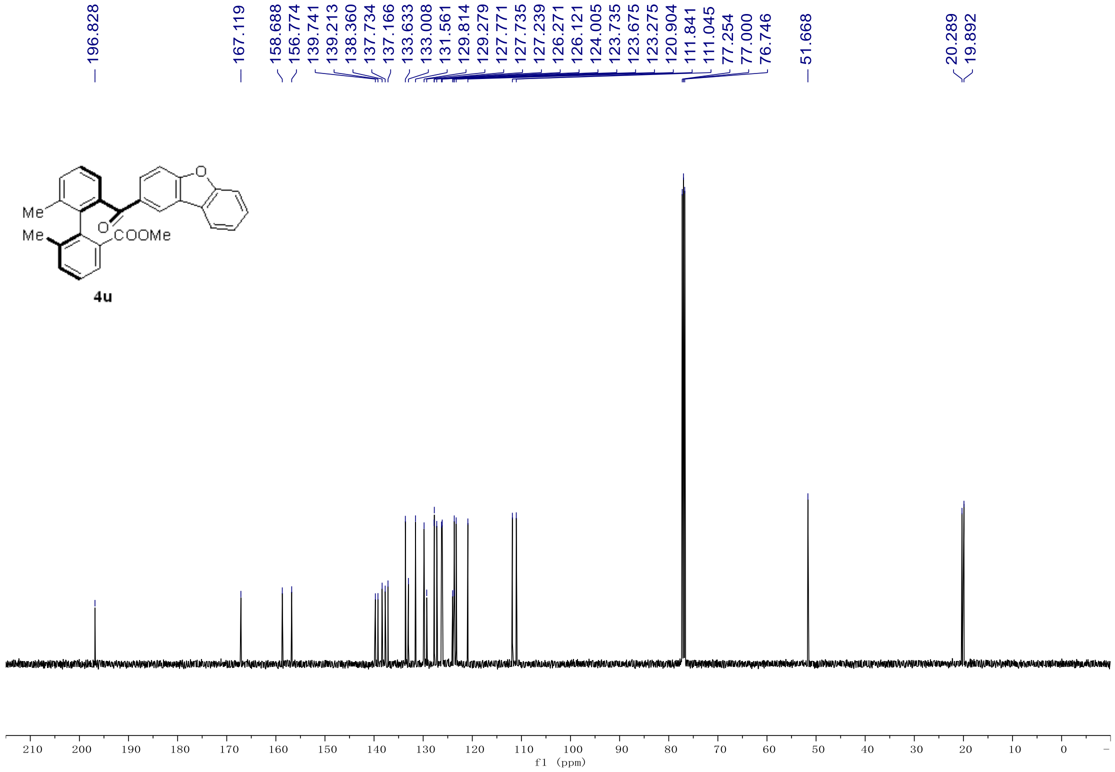
196.828
167.119
158.688
156.774
139.741
139.213
138.360
137.734
137.166
133.633
133.008
131.561
129.814
129.279
127.771
127.735
127.239
126.271
126.121
124.005
123.735
123.675
123.275
120.904
111.841
111.045
77.254
77.000
76.746
51.668
20.289
19.892
Me
Me
O
COOMe
O
4u
f1 (ppm)

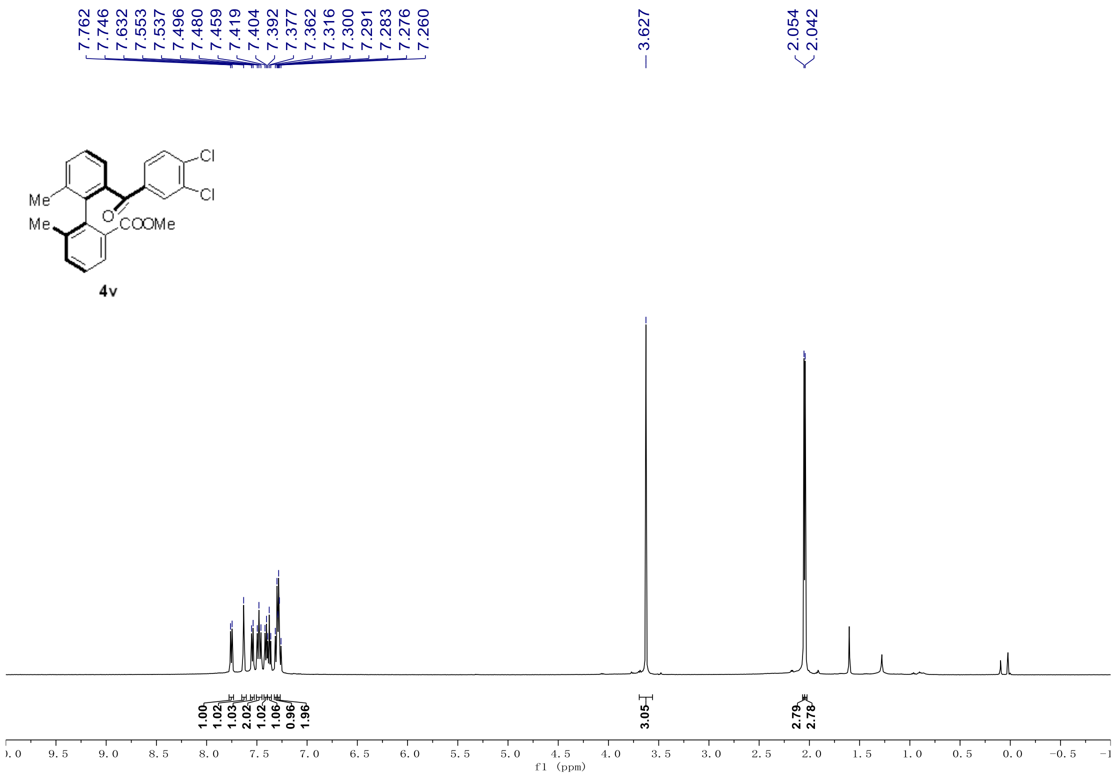

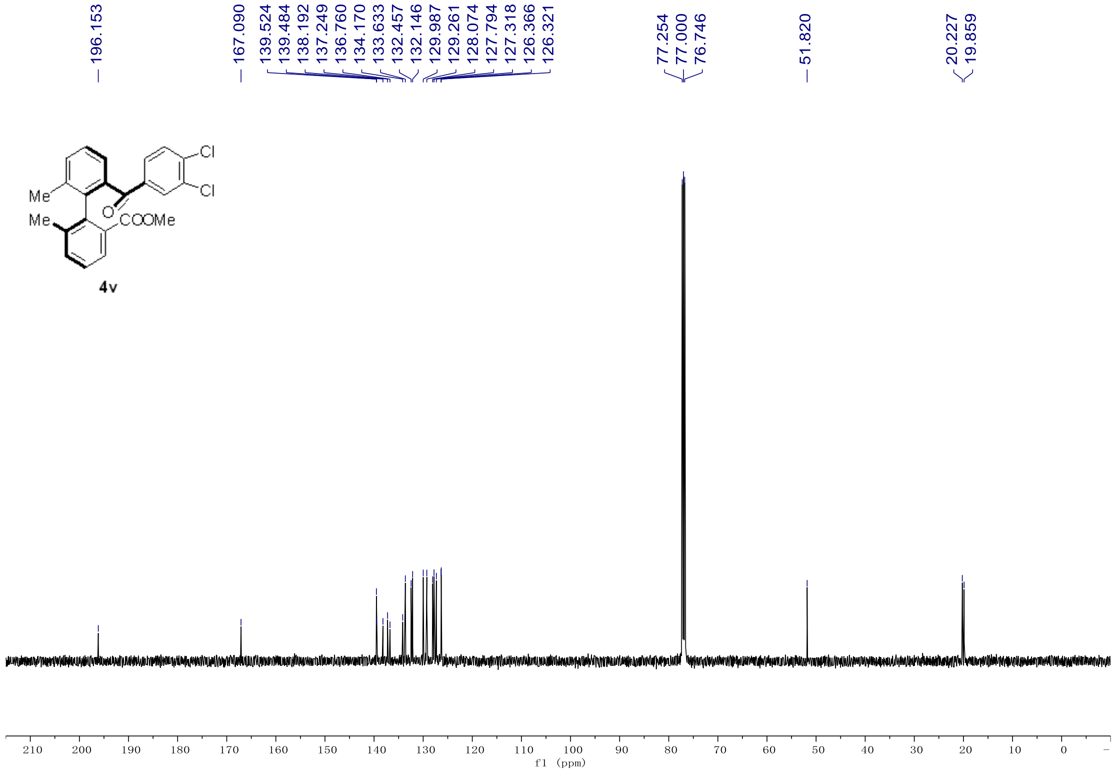

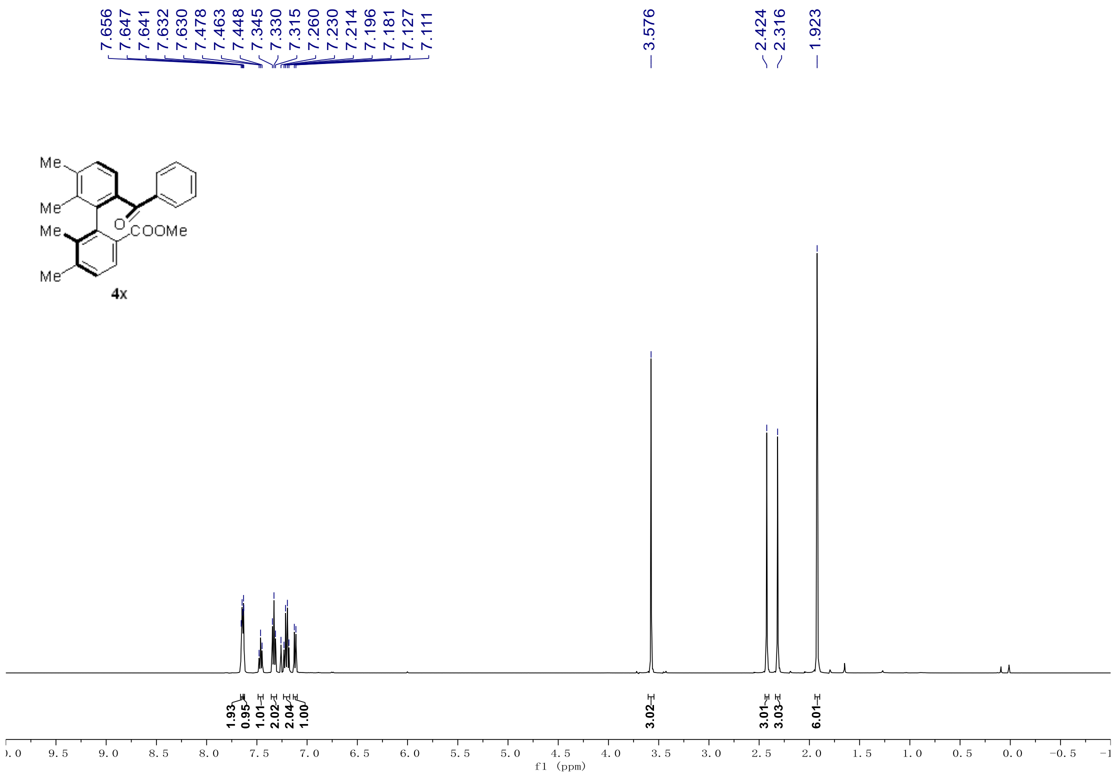
7.656 7.647 7.641 7.632 7.630 7.478 7.463 7.448 7.345 7.330 7.315 7.260 7.230 7.214 7.196 7.181 7.127 7.111
3.576
2.424 2.316
1.923
Me
Me
Me
O
COOMe
Me
4x
1.93 0.95 1.01 2.02 2.04 1.00
3.02
3.01 3.03
6.01
f1 (ppm)

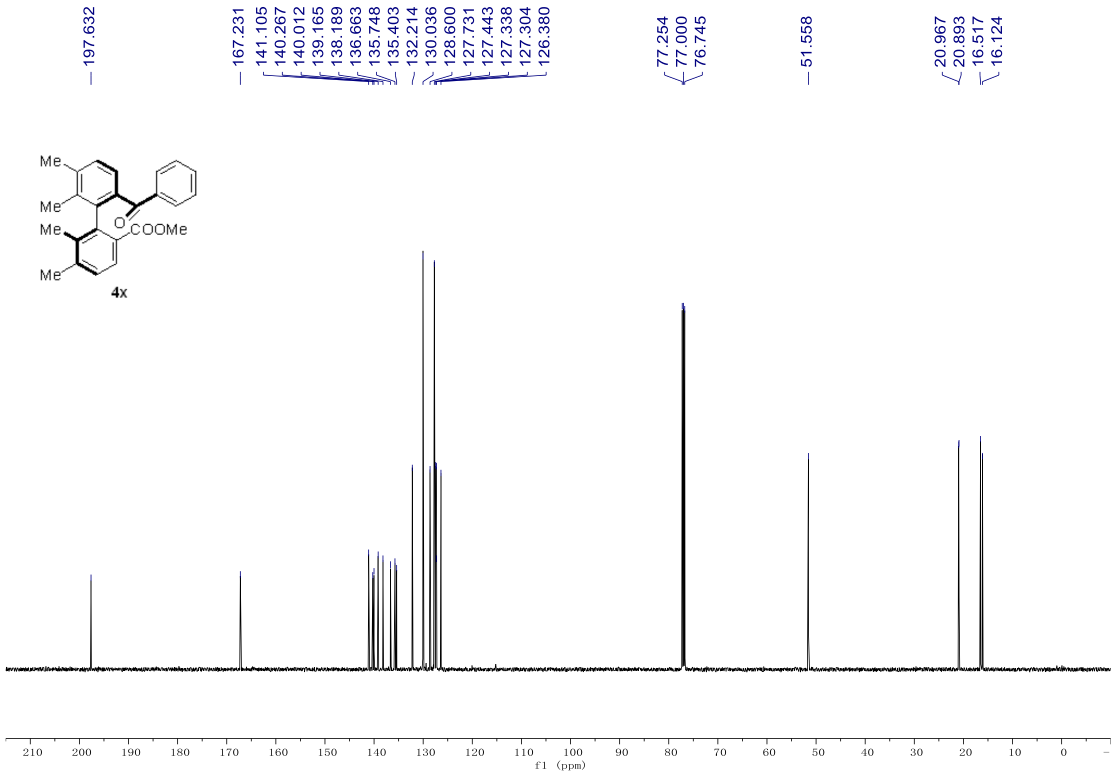
197.632
167.231 141.105 140.267 140.012 139.165 138.189 136.663 135.748 135.403 132.214 130.036 128.600 127.731 127.443 127.338 127.304 126.380
77.254 77.000 76.745
51.558
20.967 20.893 16.517 16.124
Me
Me
Me
O
COOMe
Me
4x
f1 (ppm)

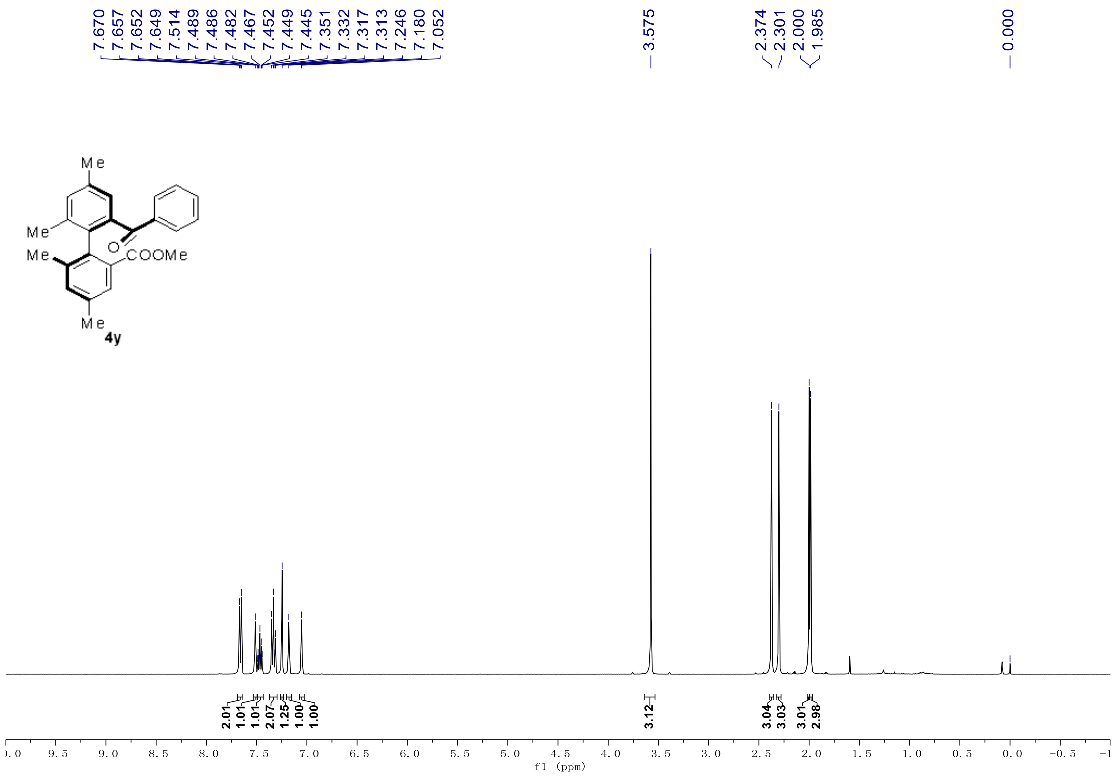
7.670
7.657
7.652
7.649
7.514
7.489
7.486
7.482
7.467
7.452
7.449
7.445
7.351
7.332
7.317
7.313
7.246
7.180
7.052
3.575
2.374
2.301
2.000
1.985
0.000
Me
Me
Me
COOMe
O
Me
4y
2.01
1.01
1.01
2.07
1.25
1.00
1.00
3.12
3.04
3.03
3.01
2.98
f1 (ppm)

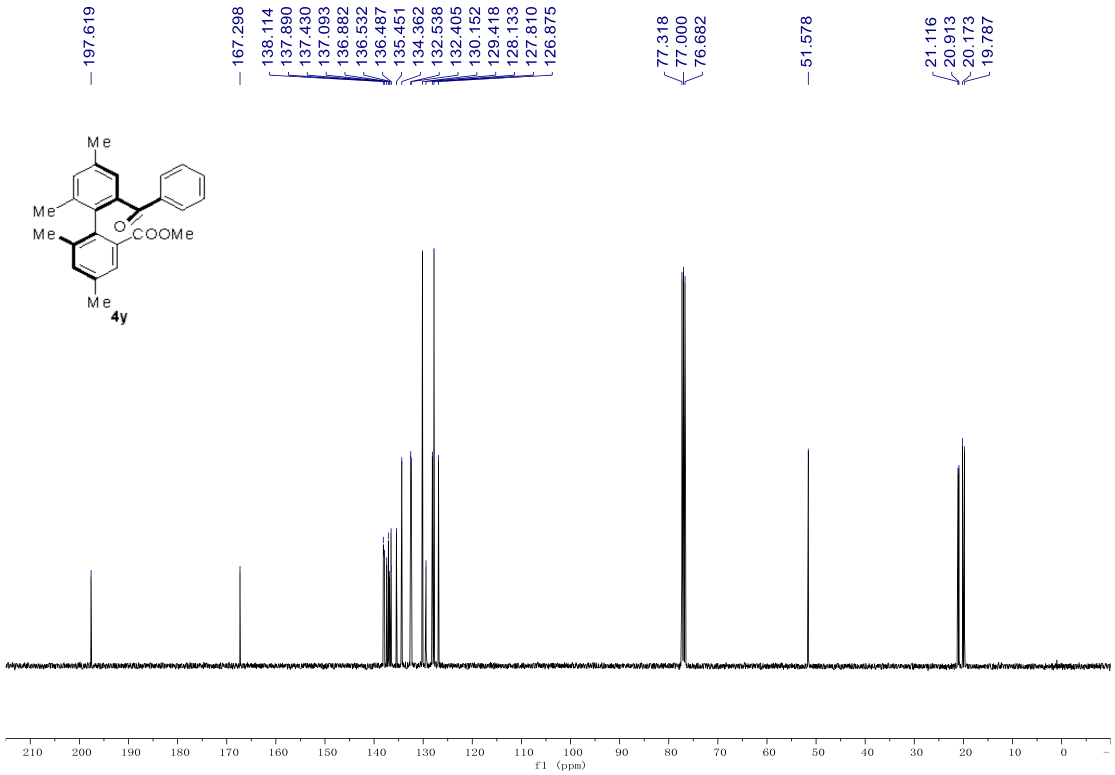
197.619
167.298
138.114
137.890
137.430
137.093
136.882
136.532
136.487
135.451
134.362
132.538
132.405
130.152
129.418
128.133
127.810
126.875
77.318
77.000
76.682
51.578
21.116
20.913
20.173
19.787
Me
Me
Me
COOMe
O
Me
4y
f1 (ppm)

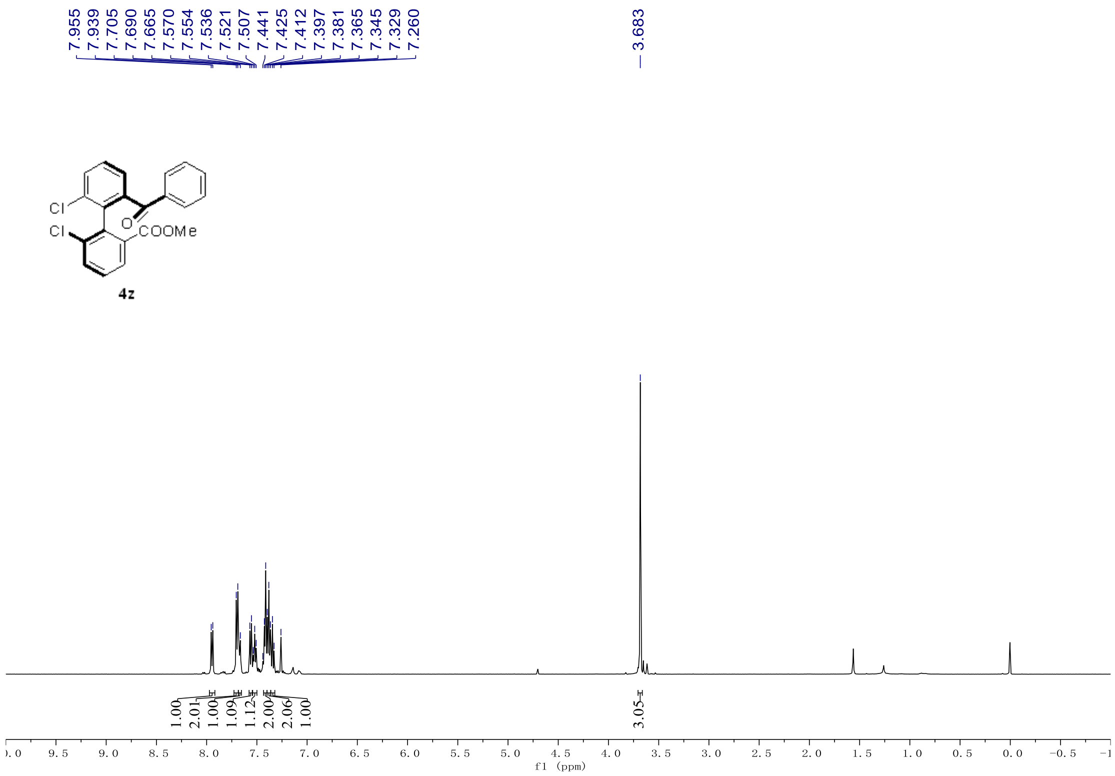
7.955
7.939
7.705
7.690
7.665
7.570
7.554
7.536
7.521
7.507
7.441
7.425
7.412
7.397
7.381
7.365
7.345
7.329
7.260
3.683
Cl
Cl
O
COOMe
4z
1.00
2.01
1.00
1.09
1.12
2.00
2.06
1.00
3.05
f1 (ppm)

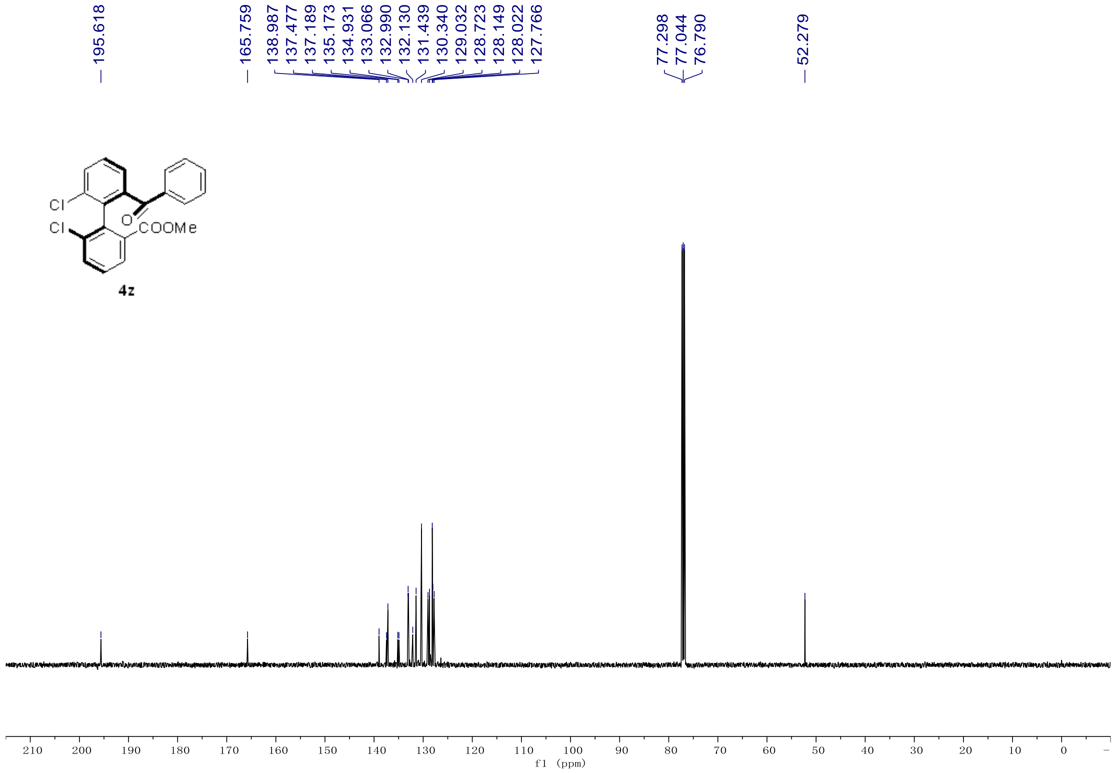
195.618
165.759
138.987
137.477
137.189
135.173
134.931
133.066
132.990
132.130
131.439
130.340
129.032
128.723
128.149
128.022
127.766
77.298
77.044
76.790
52.279
Cl
Cl
O
COOMe
4z
f1 (ppm)

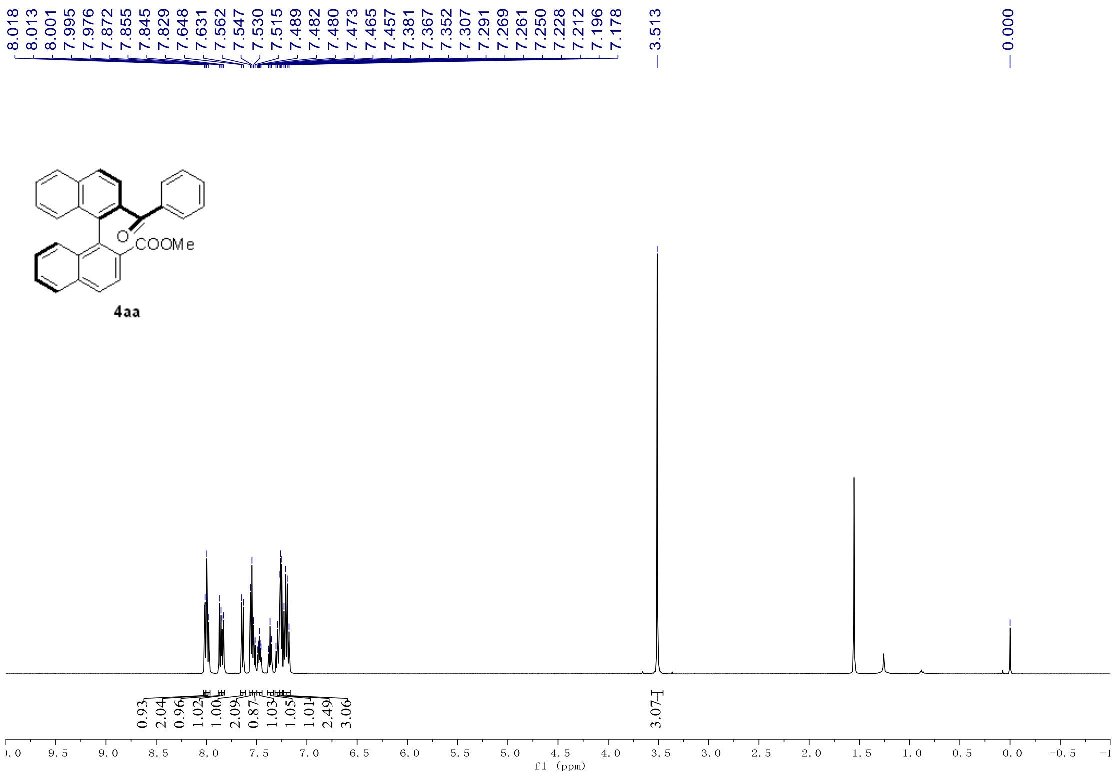
8.018
8.013
8.001
7.995
7.976
7.872
7.855
7.845
7.829
7.648
7.631
7.562
7.547
7.530
7.515
7.489
7.482
7.480
7.473
7.465
7.457
7.381
7.367
7.352
7.307
7.291
7.269
7.261
7.250
7.228
7.212
7.196
7.178
3.513
0.000
O
COOMe
4aa
0.93
2.04
0.96
1.02
1.00
2.09
0.87
1.03
1.05
1.01
2.49
3.06
3.07
f1 (ppm)

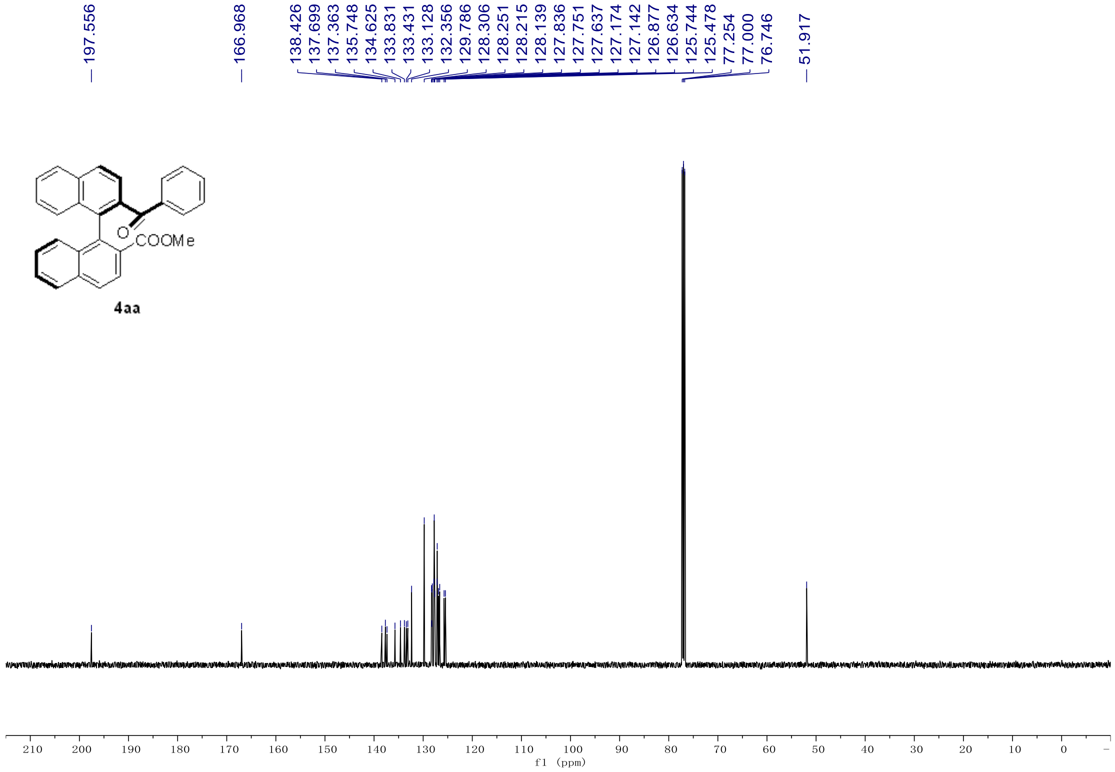
197.556
166.968
138.426
137.699
137.363
135.748
134.625
133.831
133.431
133.128
132.356
129.786
128.306
128.251
128.215
128.139
127.836
127.751
127.637
127.174
127.142
126.877
126.634
125.744
125.478
77.254
77.000
76.746
51.917
O
COOMe
4aa
f1 (ppm)

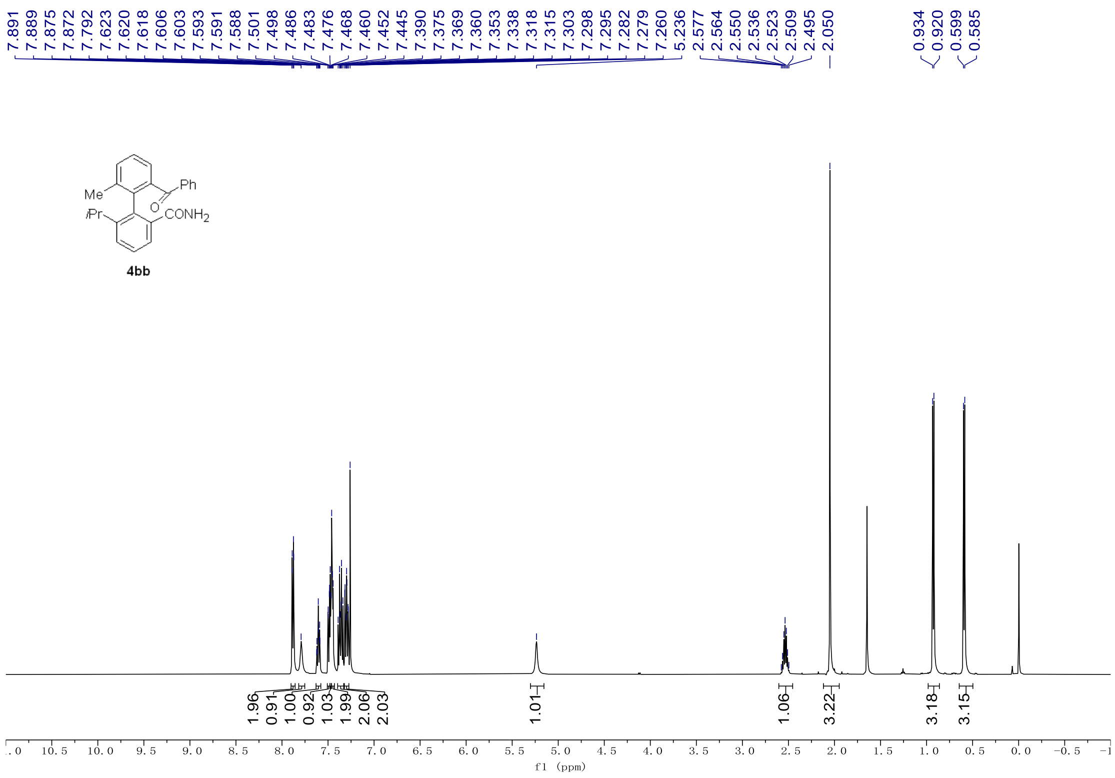
7.891 7.889 7.875 7.872 7.792 7.623 7.620 7.618 7.606 7.603 7.593 7.591 7.588 7.501 7.498 7.486 7.483 7.476 7.468 7.460 7.452 7.445 7.390 7.375 7.369 7.360 7.353 7.338 7.318 7.315 7.303 7.298 7.295 7.282 7.279 7.260 5.236 2.577 2.564 2.550 2.536 2.523 2.509 2.495 2.050 0.934 0.920 0.599 0.585
Me
Ph
O
iPr
CONH2
4bb
1.96 0.91 1.00 0.92 1.03 1.99 2.06 2.03 1.01 1.06 3.22 3.18 3.15
f1 (ppm)

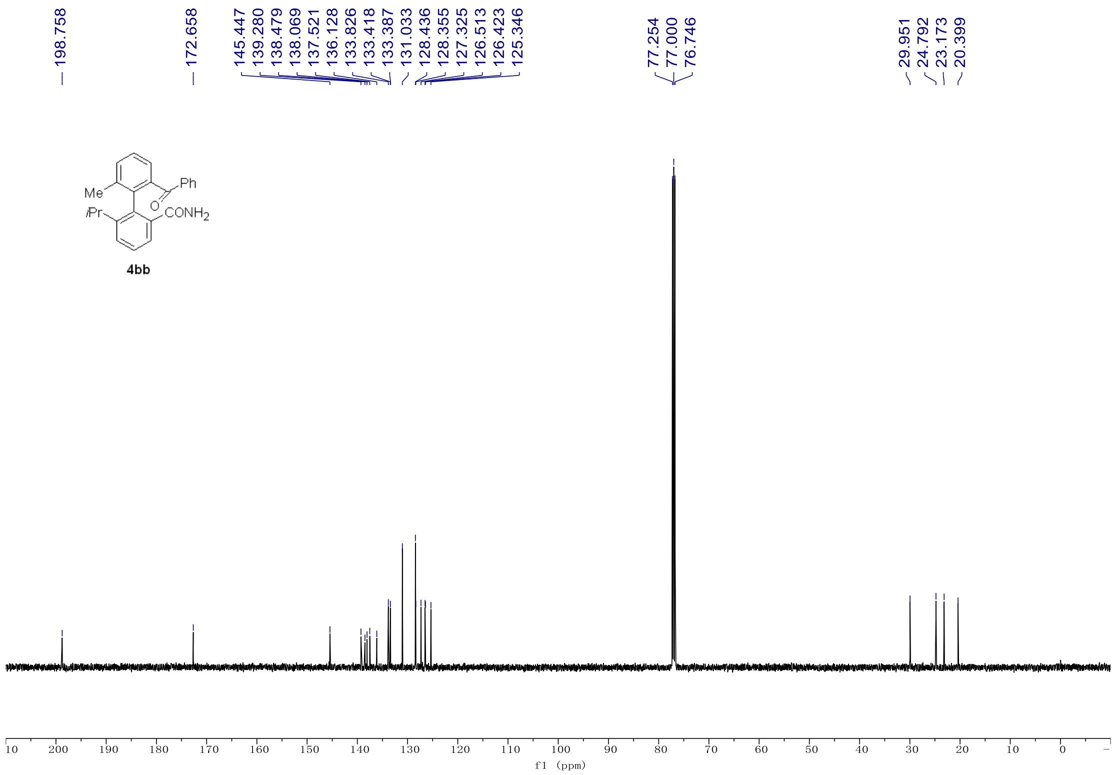
198.758 172.658 145.447 139.280 138.479 138.069 137.521 136.128 133.826 133.418 133.387 131.033 128.436 128.355 127.325 126.513 126.423 125.346 77.254 77.000 76.746 29.951 24.792 23.173 20.399
Me
Ph
O
iPr
CONH2
4bb
f1 (ppm)

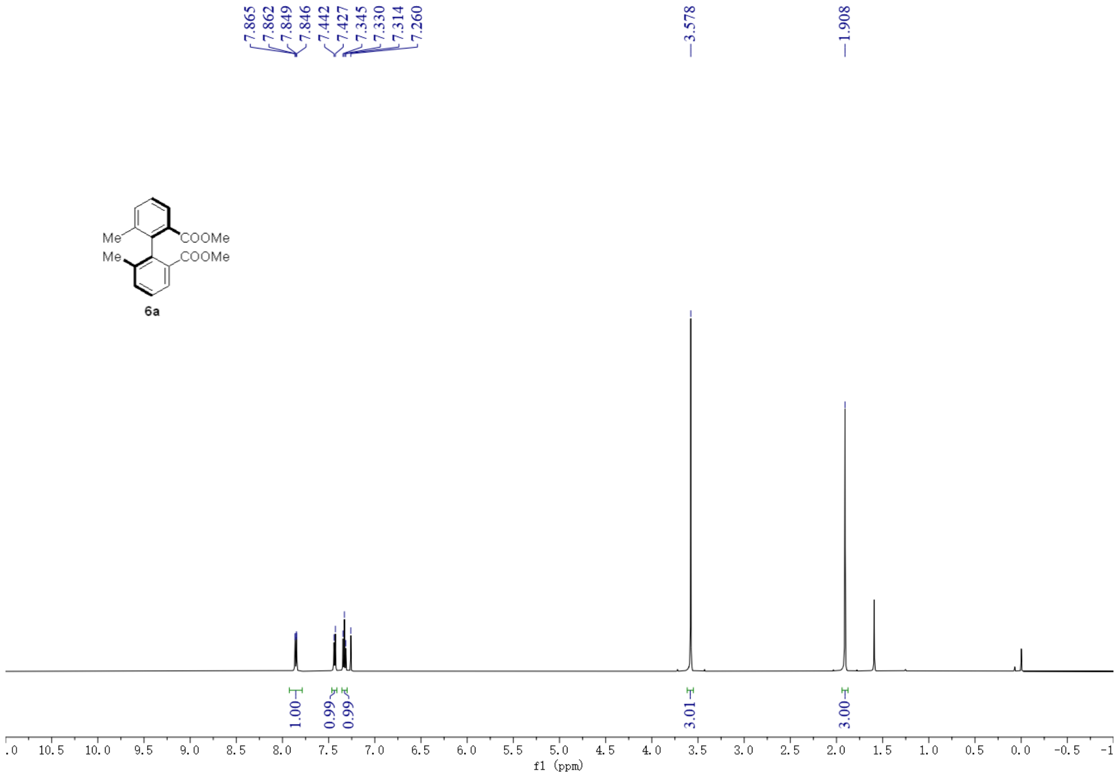
7.865
7.862
7.849
7.846
7.442
7.427
7.345
7.330
7.314
7.260
3.578
1.908
Me
COOMe
Me
COOMe
6a
1.00
0.99
0.99
3.01
3.00
f1 (ppm)

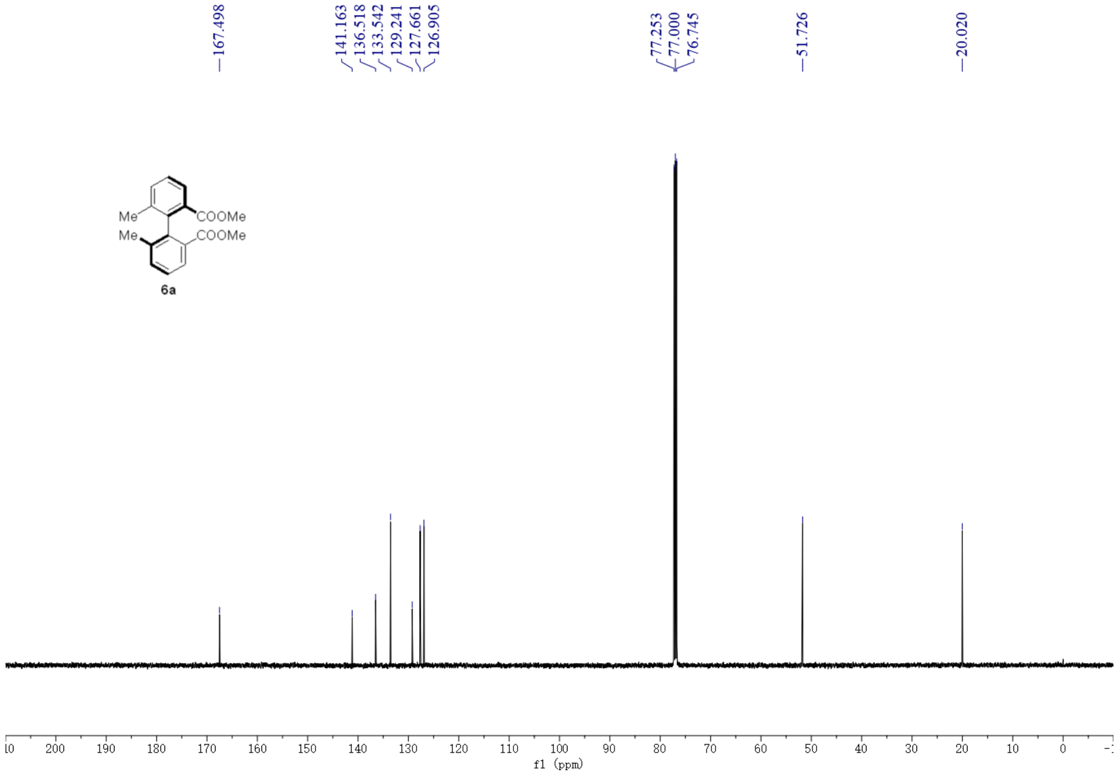
167.498
141.163
136.518
133.542
129.241
127.661
126.905
77.253
77.000
76.745
51.726
20.020
Me
COOMe
Me
COOMe
6a
f1 (ppm)

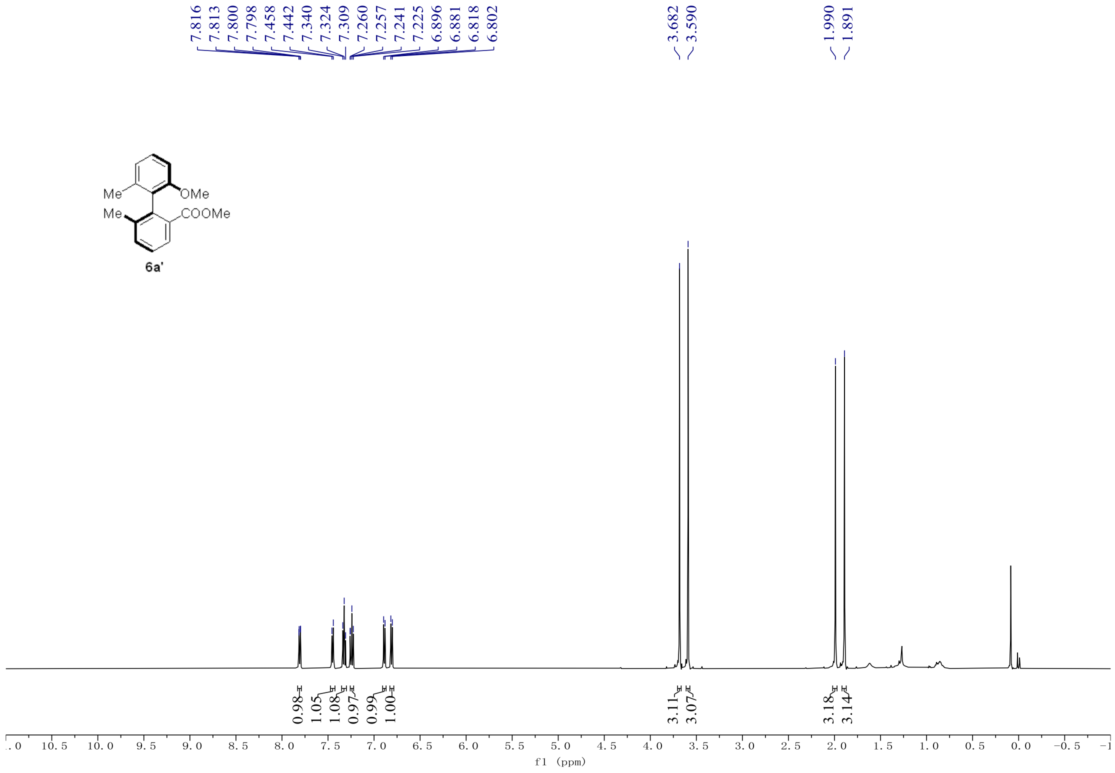
7.816
7.813
7.800
7.798
7.458
7.442
7.340
7.324
7.309
7.260
7.257
7.241
7.225
6.896
6.881
6.818
6.802
3.682
3.590
1.990
1.891
Me
OMe
Me
COOMe
6a'
0.98
1.05
1.08
0.97
0.99
1.00
3.11
3.07
3.18
3.14
f1 (ppm)

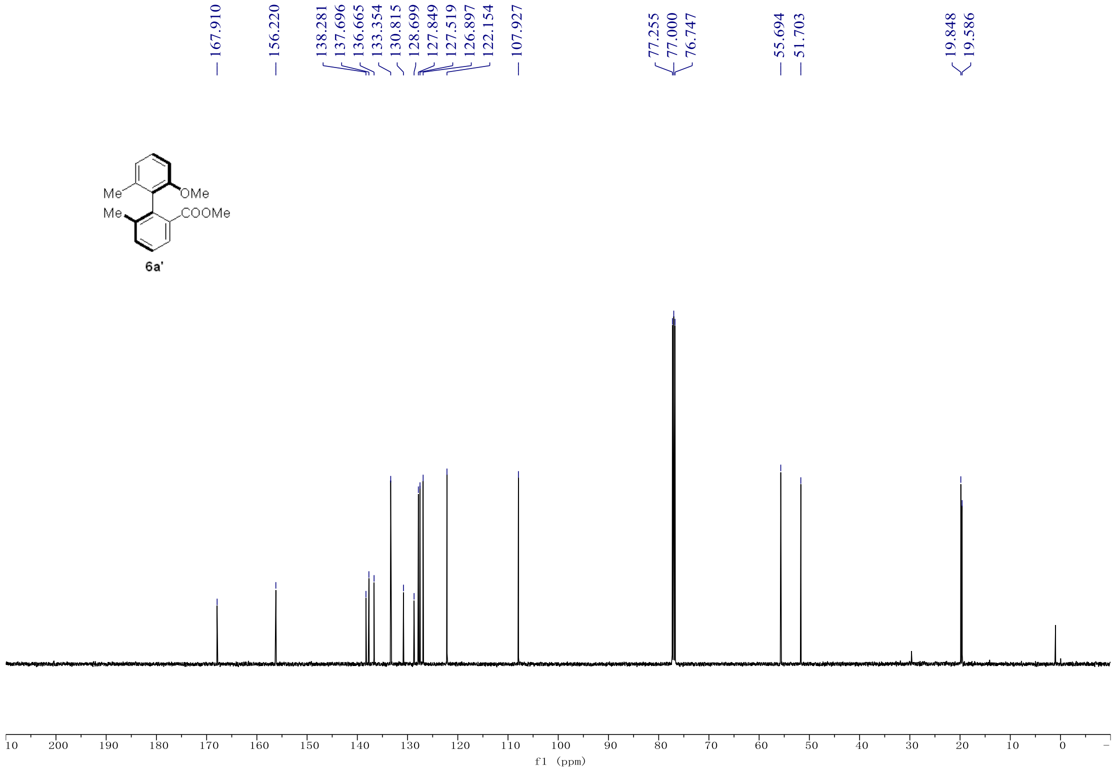
167.910
156.220
138.281
137.696
136.665
133.354
130.815
128.699
127.849
127.519
126.897
122.154
107.927
77.255
77.000
76.747
55.694
51.703
19.848
19.586
Me
OMe
Me
COOMe
6a'
f1 (ppm)

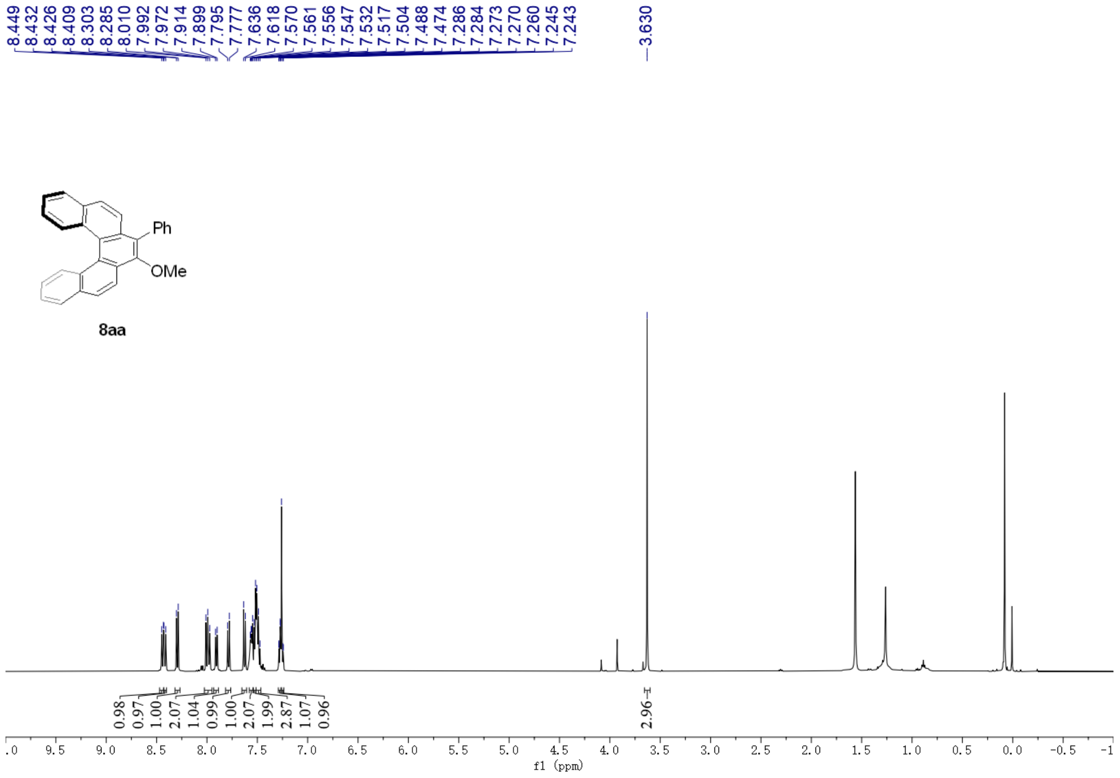
8.449
8.432
8.426
8.409
8.303
8.285
8.010
7.992
7.972
7.914
7.899
7.795
7.777
7.636
7.618
7.570
7.561
7.556
7.547
7.532
7.517
7.504
7.488
7.474
7.286
7.284
7.273
7.270
7.260
7.245
7.243
3.630
Ph
OMe
8aa
0.98
0.97
1.00
2.07
1.04
0.99
1.00
2.07
1.99
2.87
1.07
0.96
2.96
f1 (ppm)

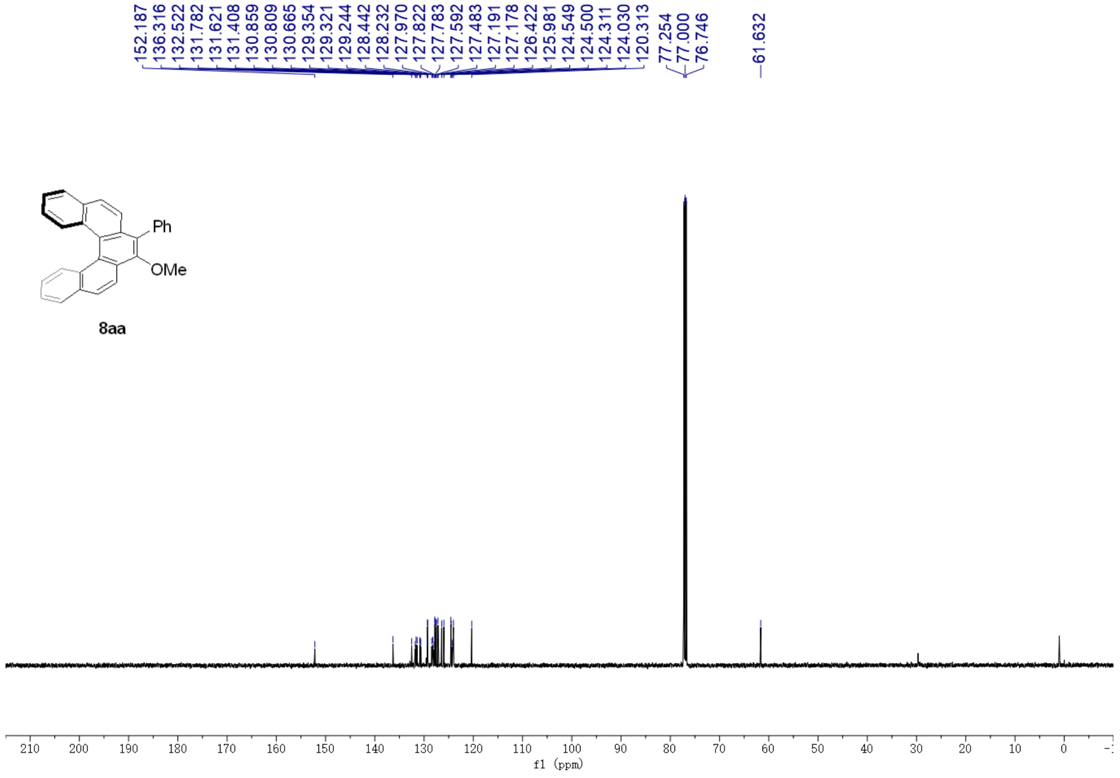
152.187
136.316
132.522
131.782
131.621
131.408
130.859
130.809
130.665
129.354
129.321
129.244
128.442
128.232
127.970
127.822
127.783
127.592
127.483
127.191
127.178
126.422
125.981
124.549
124.500
124.311
124.030
120.313
77.254
77.000
76.746
61.632
Ph
OMe
8aa
f1 (ppm)

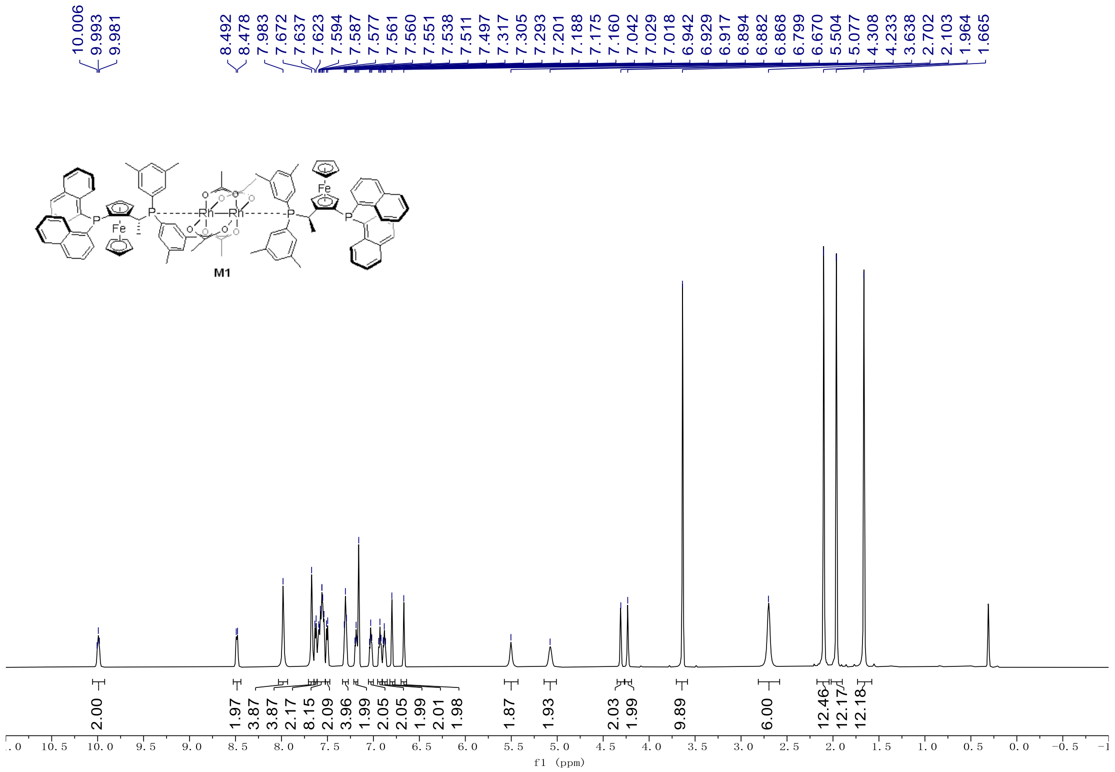
10.006
9.993
9.981
8.492
8.478
7.983
7.672
7.637
7.623
7.594
7.587
7.577
7.561
7.560
7.551
7.538
7.511
7.497
7.317
7.305
7.293
7.201
7.188
7.175
7.160
7.042
7.029
7.018
6.942
6.929
6.917
6.894
6.882
6.868
6.799
6.670
5.504
5.077
4.308
4.233
3.638
2.702
2.103
1.964
1.665
Fe
Rh
P
M1
2.00
1.97
3.87
3.87
2.17
8.15
2.09
3.96
1.99
2.05
2.05
1.99
2.01
1.98
1.87
1.93
2.03
1.99
9.89
6.00
12.46
12.17
12.18
f1 (ppm)

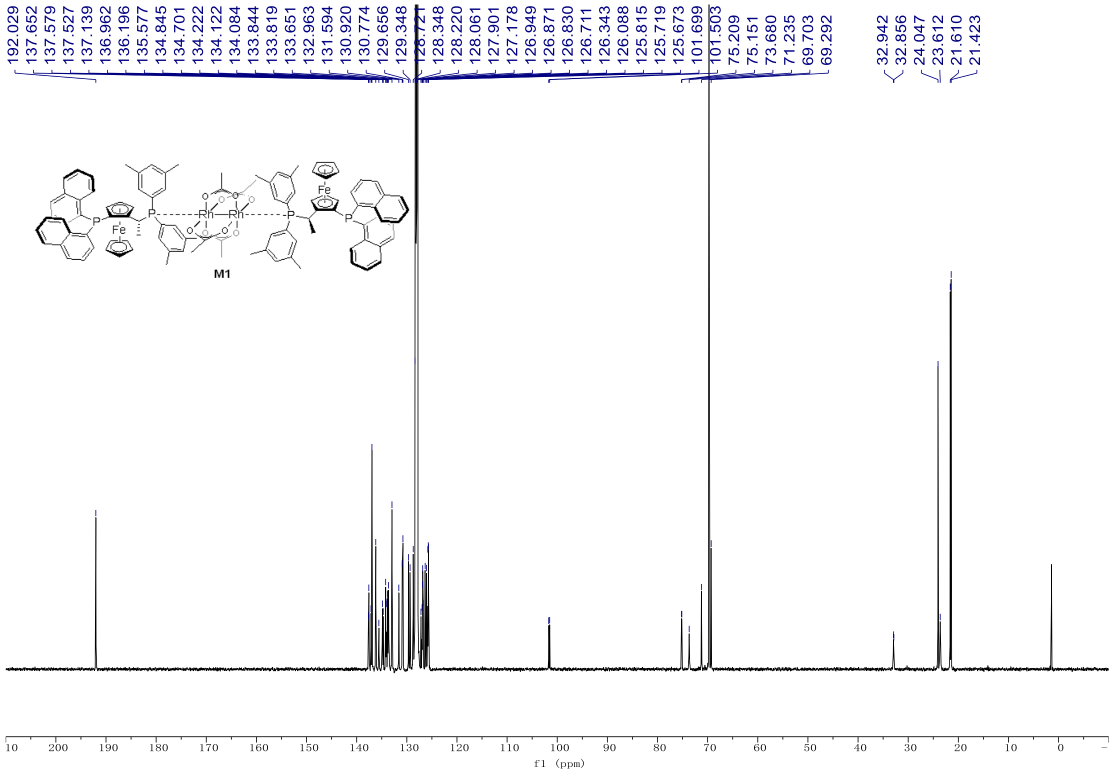
192.029
137.652
137.579
137.527
137.139
136.962
136.196
135.577
134.845
134.701
134.222
134.122
134.084
133.844
133.819
133.651
132.963
131.594
130.920
130.774
129.656
129.348
128.721
128.348
128.220
128.061
127.901
127.178
126.949
126.871
126.830
126.711
126.343
126.088
125.815
125.719
125.673
101.699
101.503
75.209
75.151
73.680
71.235
69.703
69.292
32.942
32.856
24.047
23.612
21.610
21.423
Fe
Rh
P
M1
f1 (ppm)

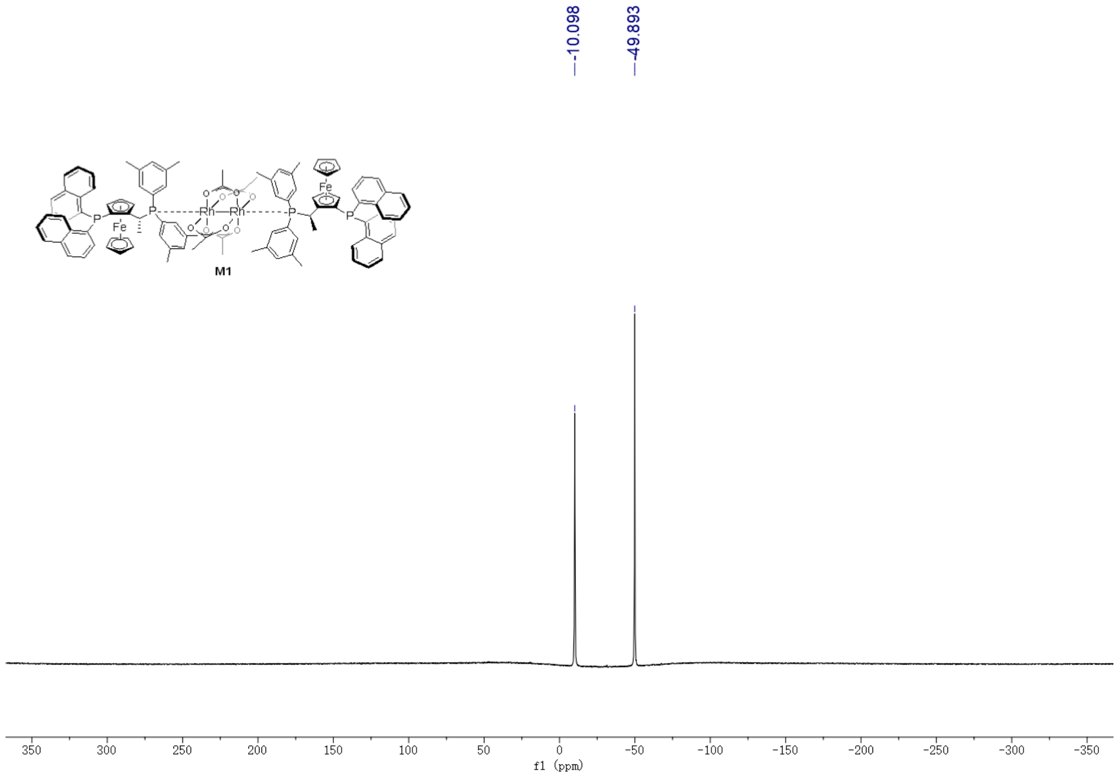
-10.098
-49.893
Fe
Rh
P
M1
f1 (ppm)

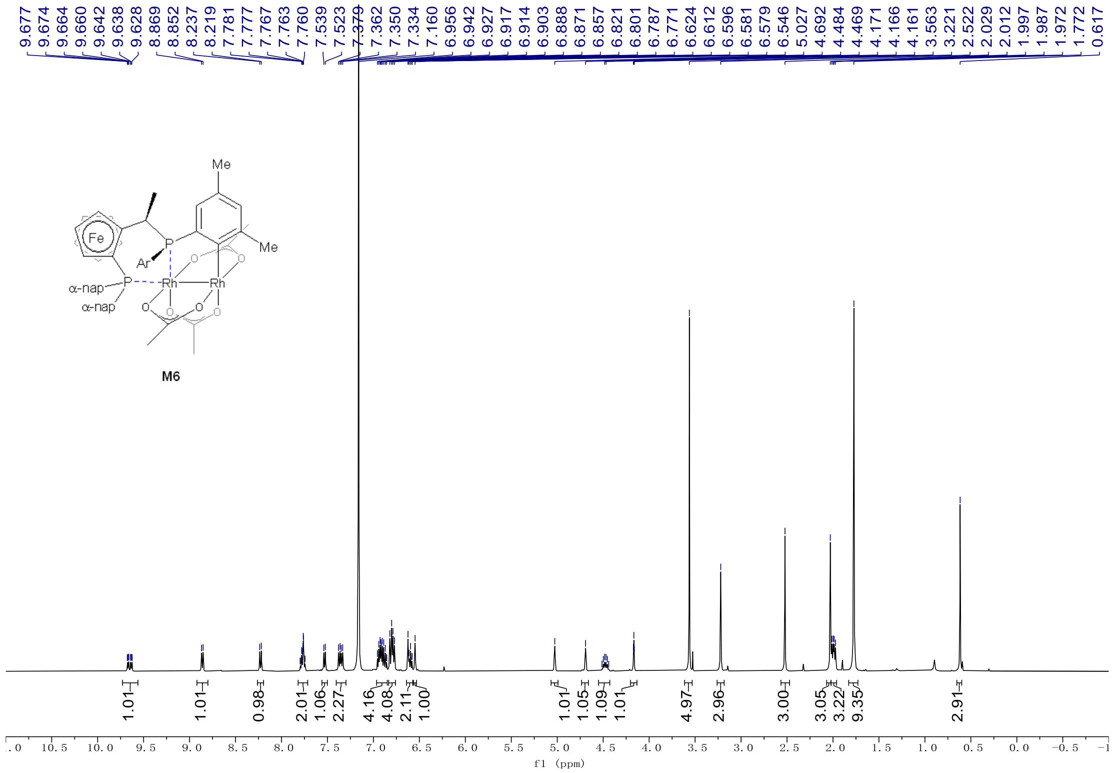
9.677 9.674 9.664 9.660 9.642 9.638 9.628 8.869 8.852 8.237 8.219 7.781 7.777 7.767 7.763 7.760 7.539 7.523 7.379 7.362 7.350 7.334 7.160 6.956 6.942 6.927 6.917 6.914 6.903 6.888 6.871 6.857 6.821 6.801 6.787 6.771 6.624 6.612 6.596 6.581 6.579 6.546 5.027 4.692 4.484 4.469 4.171 4.166 4.161 3.563 3.221 2.522 2.029 2.012 1.997 1.987 1.972 1.772 0.617
Me
Me
Fe
Ar
P
α-nap
α-nap
Rh
M6
1.01 1.01 0.98 2.01 1.06 2.27 4.16 4.08 2.11 1.00 1.01 1.05 1.09 1.01 4.97 2.96 3.00 3.05 3.22 9.35 2.91
f1 (ppm)

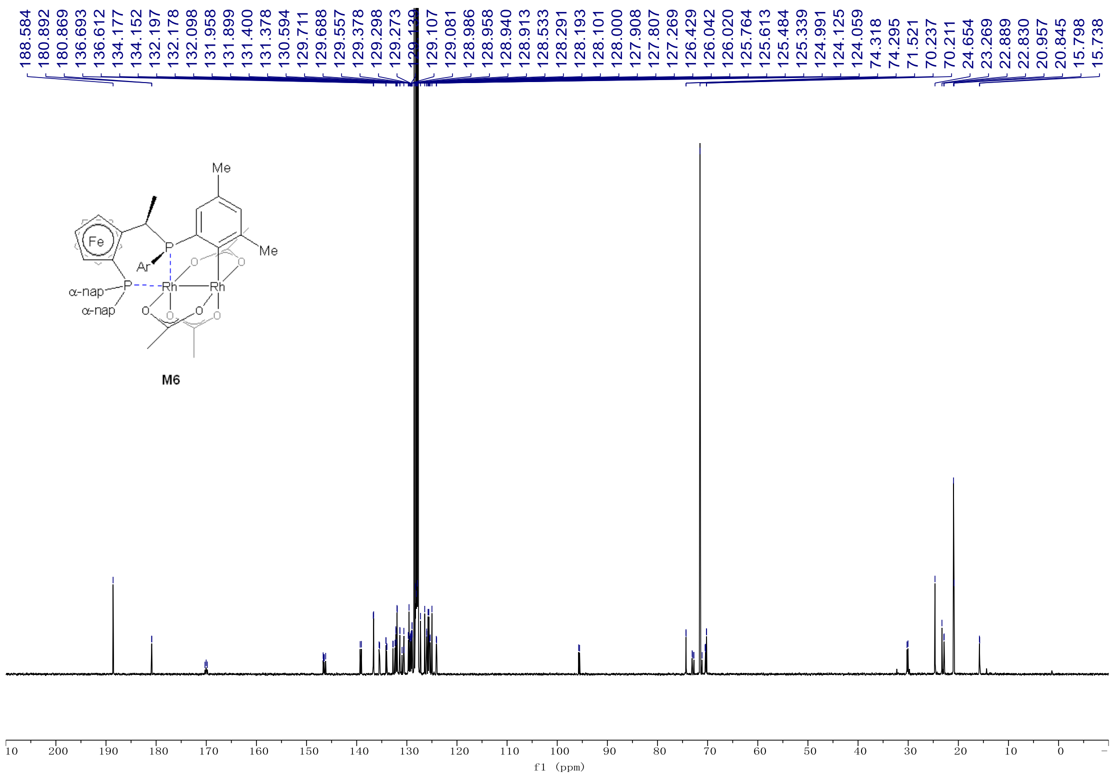
188.584
180.892
180.869
136.693
136.612
134.177
134.152
132.197
132.178
132.098
131.958
131.899
131.400
131.378
130.594
129.711
129.688
129.557
129.378
129.298
129.273
129.107
129.081
128.986
128.958
128.940
128.913
128.533
128.291
128.193
128.101
128.000
127.908
127.807
127.269
126.429
126.042
126.020
125.764
125.613
125.484
125.339
124.991
124.125
124.059
74.318
74.295
71.521
70.237
70.211
24.654
23.269
22.889
22.830
20.957
20.845
15.798
15.738
Me
Fe
Me
Ar
P
α-nap
α-nap
P
Rh
Rh
O
M6
f1 (ppm)

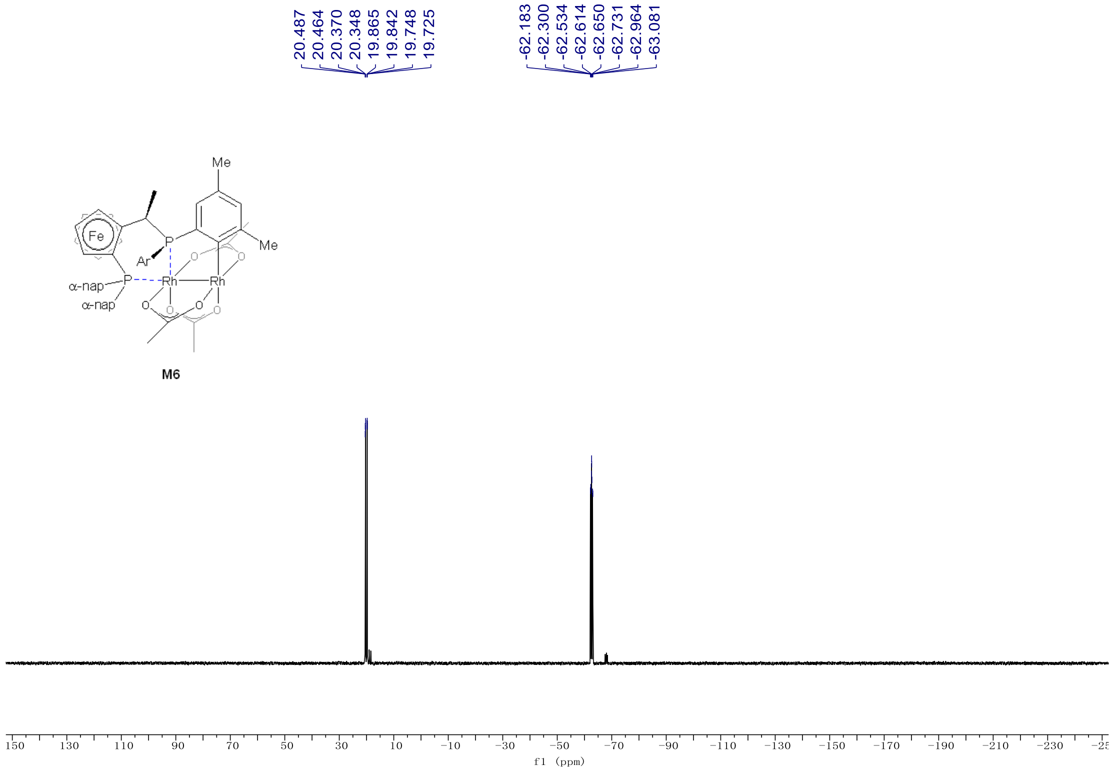
20.487
20.464
20.370
20.348
19.865
19.842
19.748
19.725
-62.183
-62.300
-62.534
-62.614
-62.650
-62.731
-62.964
-63.081
Me
Fe
Me
Ar
P
α-nap
α-nap
P
Rh
Rh
O
M6
f1 (ppm)

Copies of HPLC Traces

<Chromatogram>

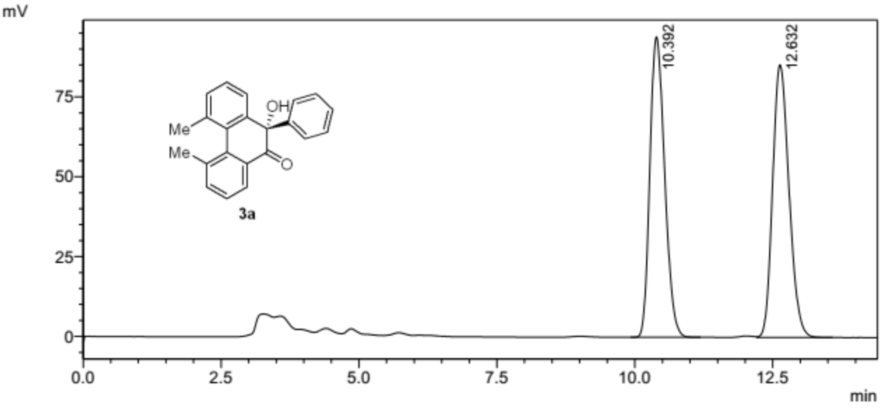

<Peak Table>

Chiralcel AD-H, isopropanol/hexane = 10:90, flow: 1.0 mL/min, λ = 254 nm.

Peak#	Ret. Time	Area	Height	Conc.	Unit	Mark	Name
1	10.392	1754130	94046	49.825			
2	12.632	1766461	85386	50.175			
Total		3520591	179432				

<Chromatogram>

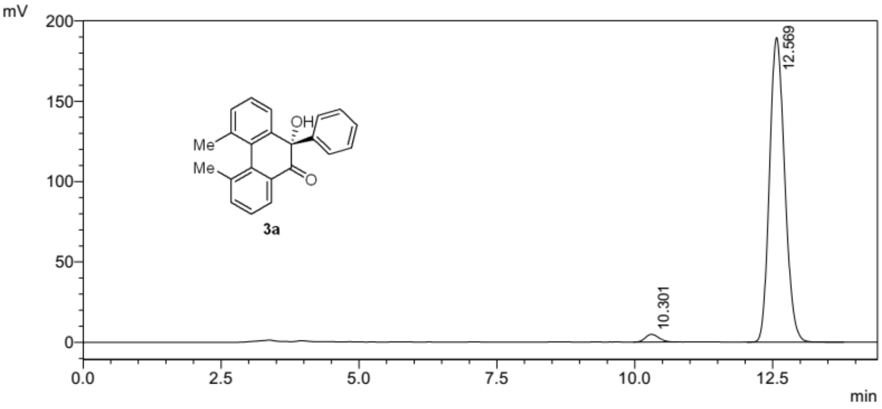

<Peak Table>

Chiralcel AD-H, isopropanol/hexane = 10:90, flow: 1.0 mL/min, λ = 254 nm.

Peak#	Ret. Time	Area	Height	Conc.	Unit	Mark	Name
1	10.301	81579	4781	2.198			
2	12.569	3629758	189368	97.802			
Total		3711337	194149				

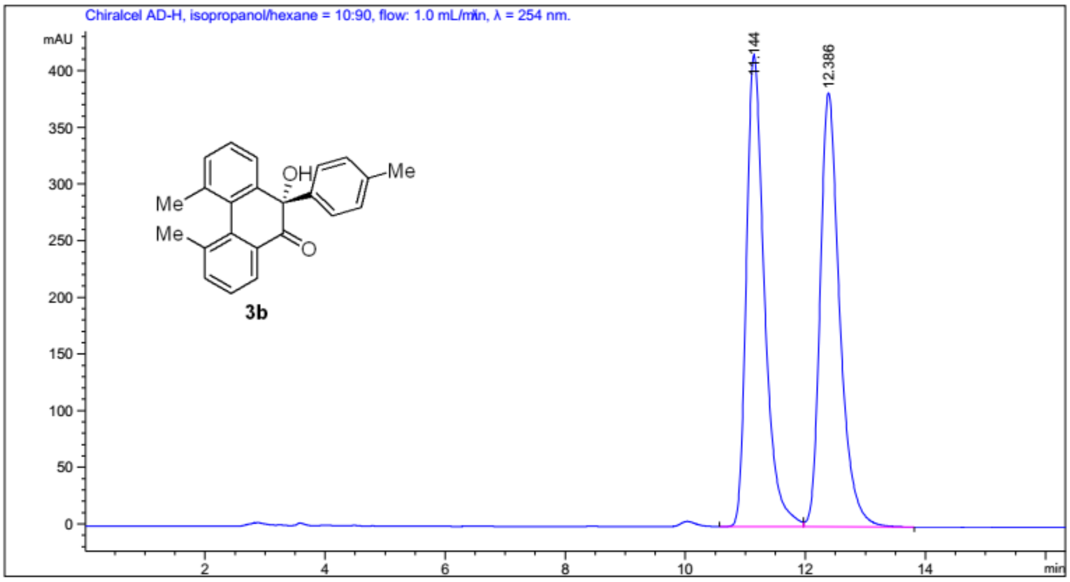

Signal 1: DAD1 A, Sig=254,4 Ref=off

Peak #	RetTime [min]	Type	Width [min]	Area [mAU*s]	Height [mAU]	Area %
1	11.144	BV	0.3153	8692.07227	416.79050	50.1292
2	12.386	VB	0.3393	8647.26074	383.10651	49.8708

Totals : 1.73393e4 799.89700

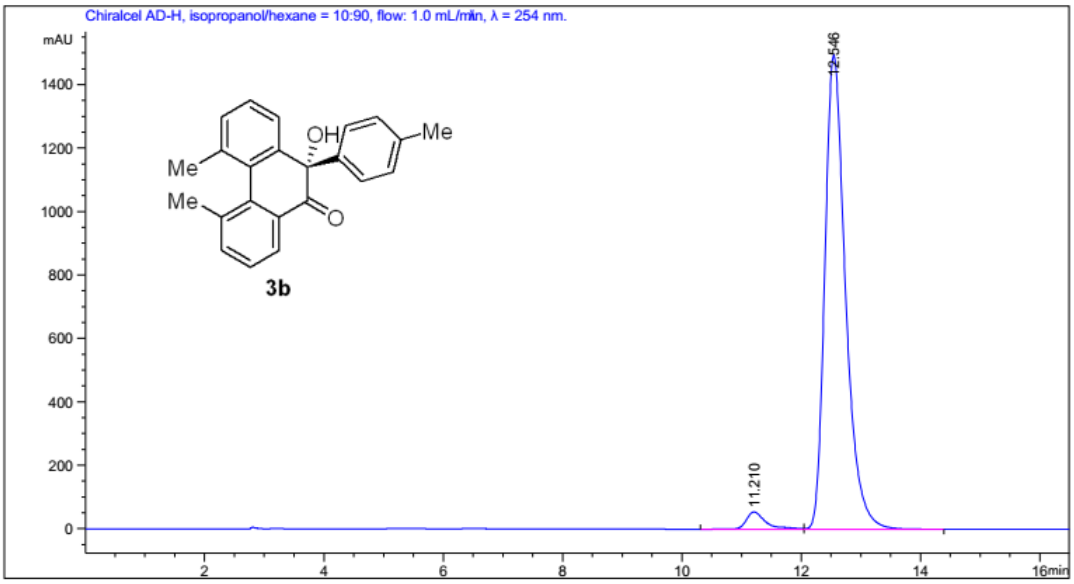

Peak #	RetTime [min]	Type	Width [min]	Area [mAU*s]	Height [mAU]	Area %
1	11.210	BV	0.3383	1241.89368	54.41093	3.3432
2	12.546	VB	0.3662	3.59051e4	1494.95483	96.6568

Totals : 3.71470e4 1549.36576

<Chromatogram>

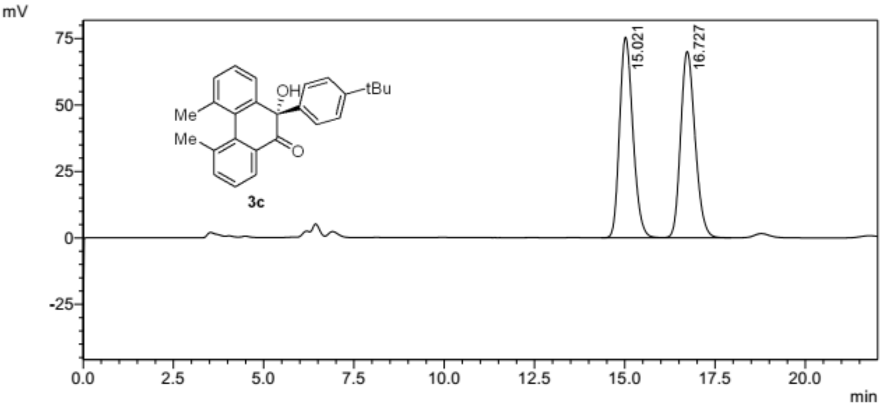

<Peak Table>

Chiralcel ID, isopropanol/hexane = 7:93, flow: 1.0 mL/min, λ = 254 nm.

Peak#	Ret. Time	Area	Height	Conc.	Unit	Mark	Name
1	15.021	1943962	75456	49.849			
2	16.727	1955717	70116	50.151			
Total		3899679	145572				

<Chromatogram>

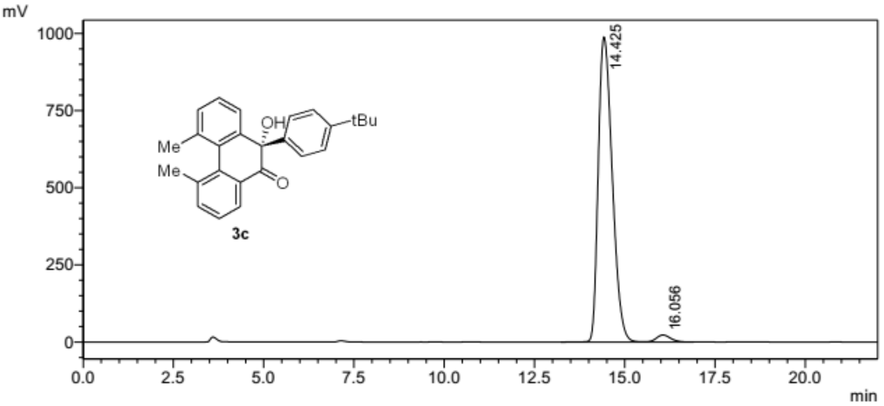

<Peak Table>

Chiralcel ID, isopropanol/hexane = 7:93, flow: 1.0 mL/min, λ = 254 nm.

Peak#	Ret. Time	Area	Height	Conc.	Unit	Mark	Name
1	14.425	26619400	988095	97.677			
2	16.056	632981	22745	2.323			
Total		27252381	1010840				

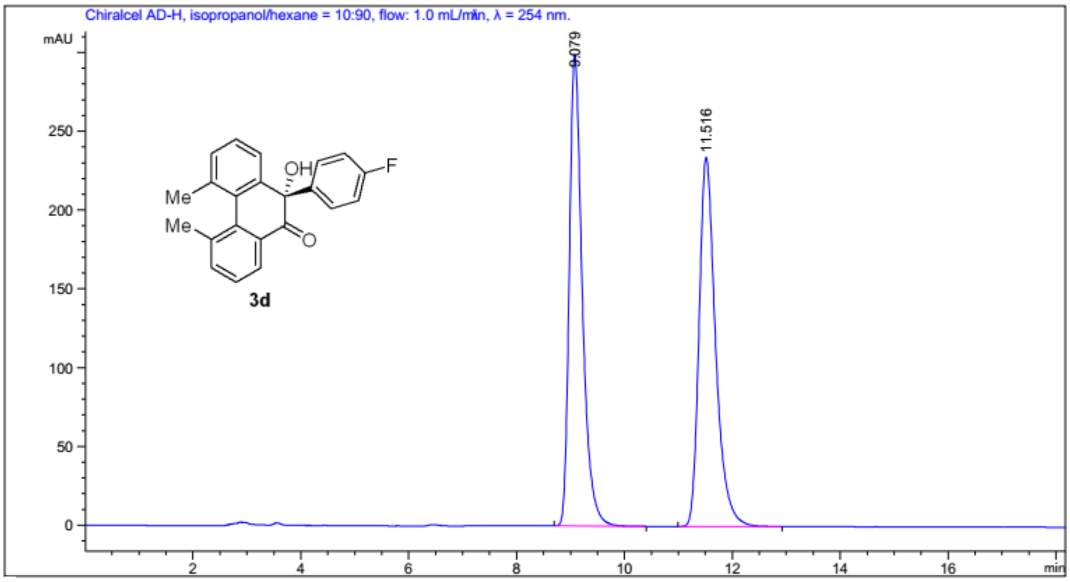

Peak #	RetTime [min]	Type	Width [min]	Area [mAU*s]	Height [mAU]	Area %
1	9.079	BB	0.2525	4982.59424	298.93875	50.0540
2	11.516	BB	0.3216	4971.84619	234.25899	49.9460
Totals :				9954.44043	533.19774	

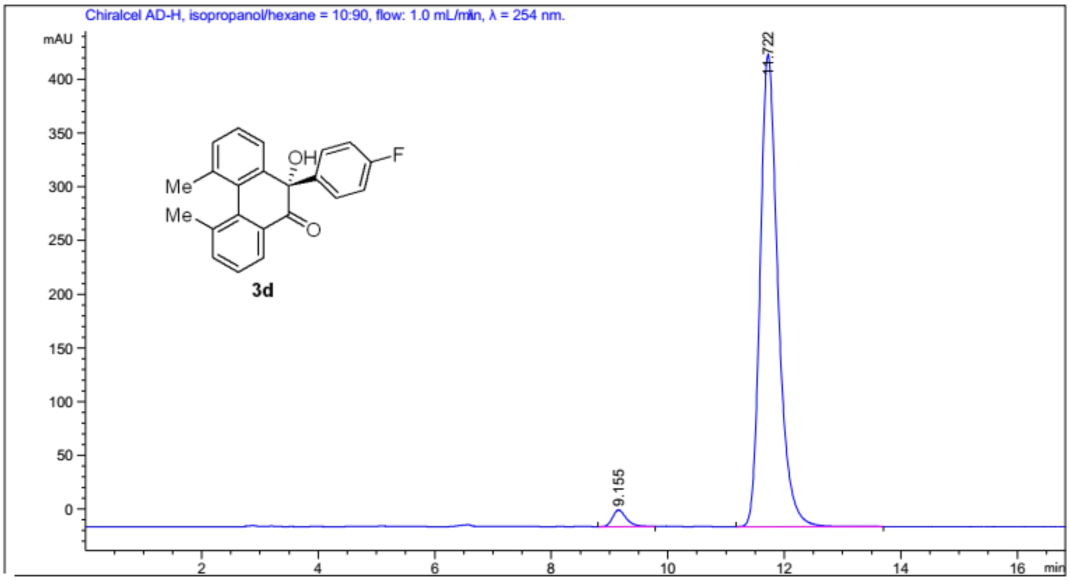

Peak #	RetTime [min]	Type	Width [min]	Area [mAU*s]	Height [mAU]	Area %
1	9.155	BB	0.2475	253.15398	15.42511	2.6390
2	11.722	BB	0.3240	9339.50195	439.39182	97.3610
Totals :				9592.65593	454.81692	

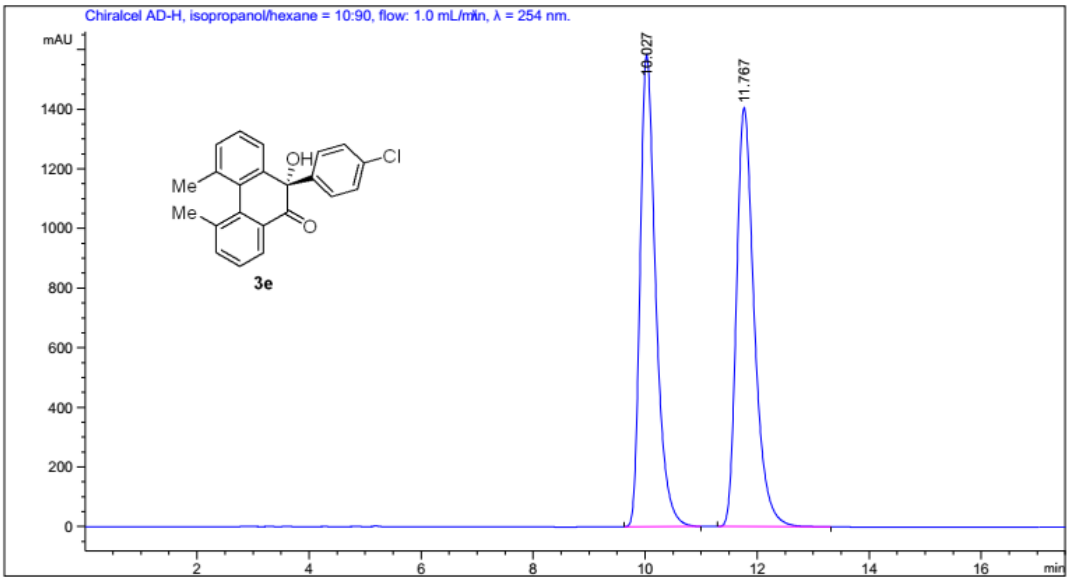

Peak #	RetTime [min]	Type	Width [min]	Area [mAU*s]	Height [mAU]	Area %
1	10.027	BB	0.2935	3.05791e4	1582.33667	49.6486
2	11.767	BB	0.3355	3.10119e4	1405.20996	50.3514

Totals : 6.15911e4 2987.54663

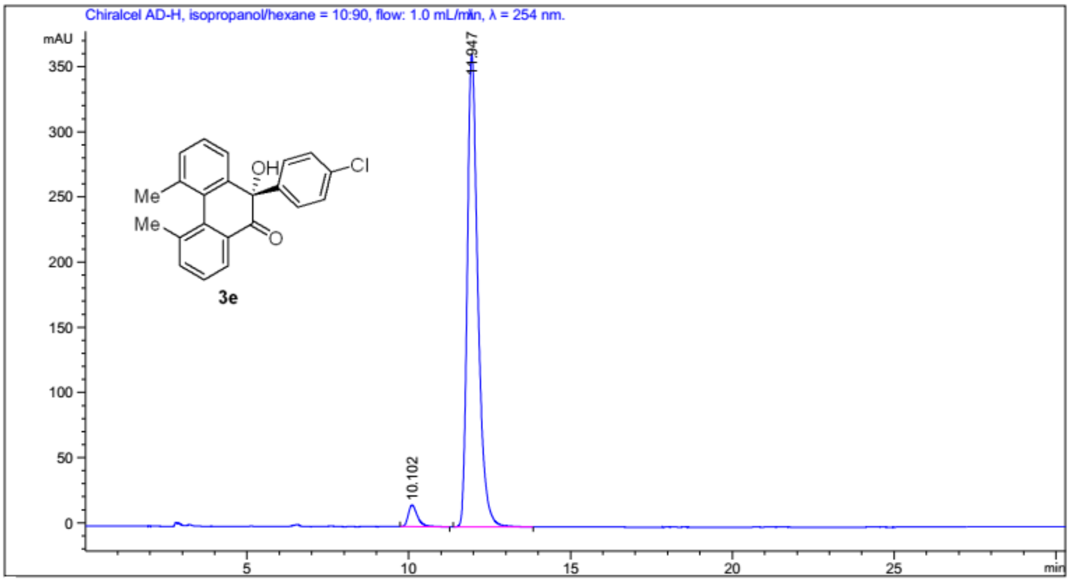

Peak #	RetTime [min]	Type	Width [min]	Area [mAU*s]	Height [mAU]	Area %
1	10.102	BB	0.2941	326.52063	16.69781	3.8023
2	11.947	BB	0.3460	8260.98926	362.35956	96.1977

Totals : 8587.50989 379.05737

<Chromatogram>

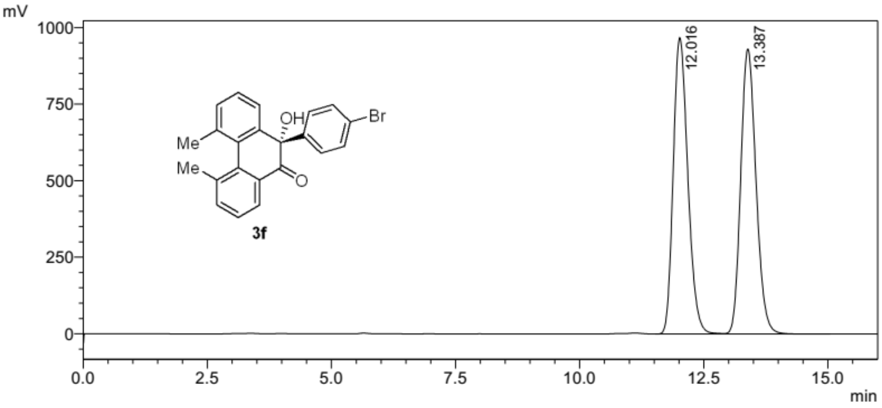

<Peak Table>

Chiralcel AD-H, isopropanol/hexane = 10:90, flow: 1.0 mL/min, λ = 254 nm.

Peak#	Ret. Time	Area	Height	Conc.	Unit	Mark	Name
1	12.016	19788263	967572	49.848			
2	13.387	19908773	930702	50.152			
Total		39697037	1898274				

<Chromatogram>

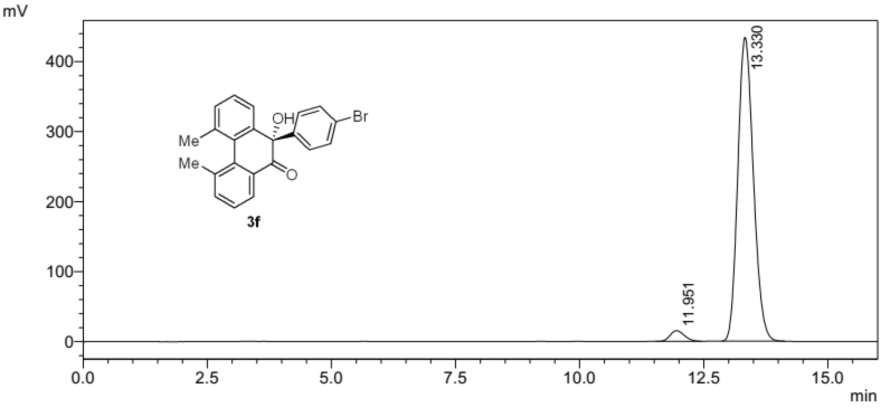

<Peak Table>

Chiralcel AD-H, isopropanol/hexane = 10:90, flow: 1.0 mL/min, λ = 254 nm.

Peak#	Ret. Time	Area	Height	Conc.	Unit	Mark	Name
1	11.951	307723	15285	3.142			
2	13.330	9486462	433723	96.858			
Total		9794185	449008				

<Chromatogram>

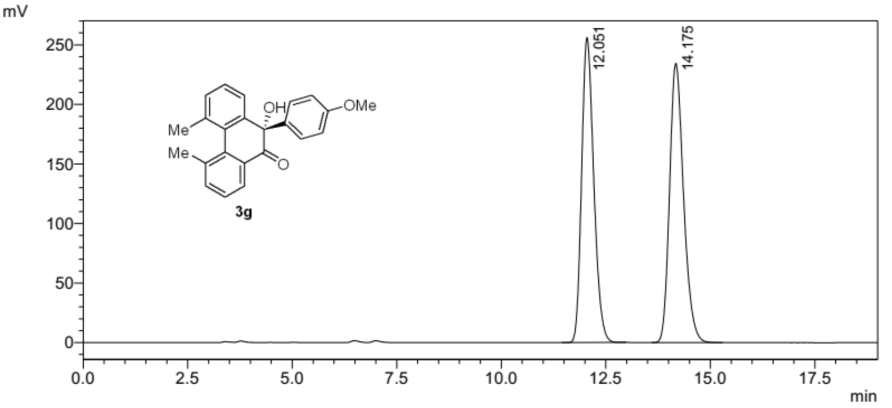

<Peak Table>

Chiralcel AD-H, isopropanol/hexane = 20:80, flow: 1.0 mL/min, λ = 254 nm.

Peak#	Ret. Time	Area	Height	Conc.	Unit	Mark	Name
1	12.051	5162533	255863	48.557			
2	14.175	5469351	234470	51.443			
Total		10631884	490332				

<Chromatogram>

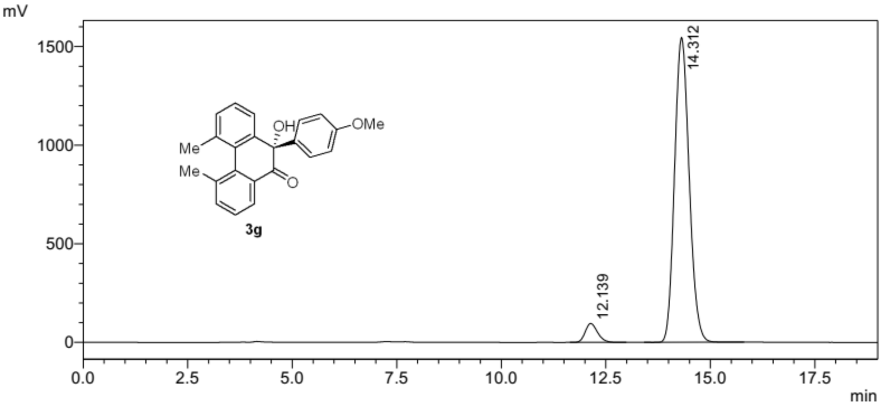

<Peak Table>

Chiralcel AD-H, isopropanol/hexane = 20:80, flow: 1.0 mL/min, λ = 254 nm.

Peak#	Ret. Time	Area	Height	Conc.	Unit	Mark	Name
1	12.139	1965545	95540	4.969			
2	14.312	37591200	1544692	95.031			
Total		39556745	1640232				

<Chromatogram>

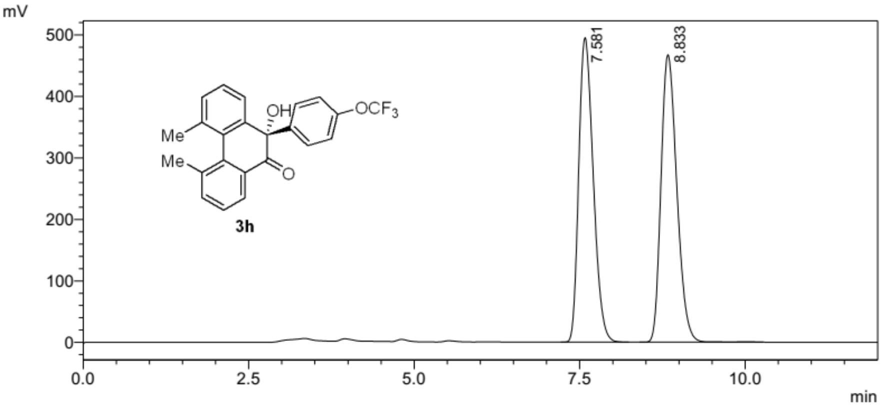

<Peak Table>

Chiralcel AD-H, isopropanol/hexane = 10:90, flow: 1.0 mL/min, λ = 254 nm.

Peak#	Ret. Time	Area	Height	Conc.	Unit	Mark	Name
1	7.581	7533147	494816	49.229			
2	8.833	7769030	467563	50.771			
Total		15302177	962379				

<Chromatogram>

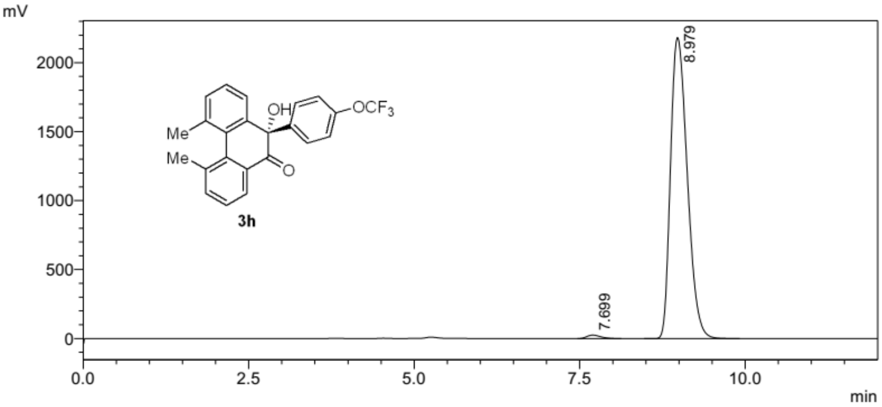

<Peak Table>

Chiralcel AD-H, isopropanol/hexane = 10:90, flow: 1.0 mL/min, λ = 254 nm.

Peak#	Ret. Time	Area	Height	Conc.	Unit	Mark	Name
1	7.699	370746	25507	0.955			
2	8.979	38443389	2181895	99.045			
Total		38814134	2207402				

<Chromatogram>

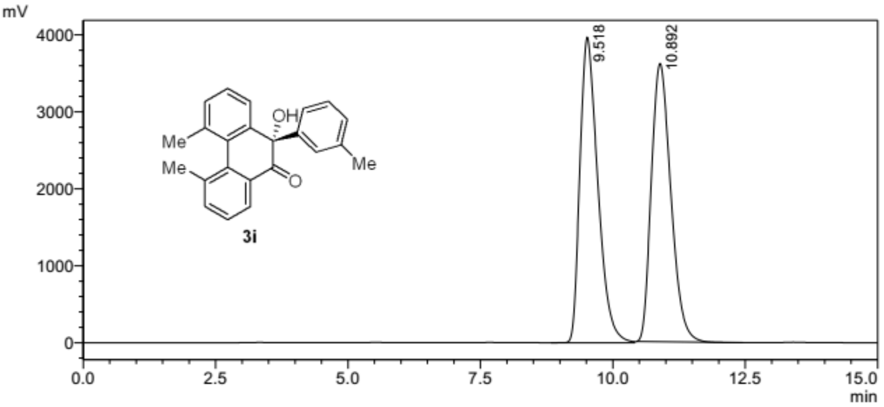

<Peak Table>

Chiralcel AD-H, isopropanol/hexane = 10:90, flow: 1.0 mL/min, λ = 254 nm.

Peak#	Ret. Time	Area	Height	Conc.	Unit	Mark	Name
1	9.518	92962156	3967135	50.132			
2	10.892	92472726	3613311	49.868			
Total		185434881	7580446				

<Chromatogram>

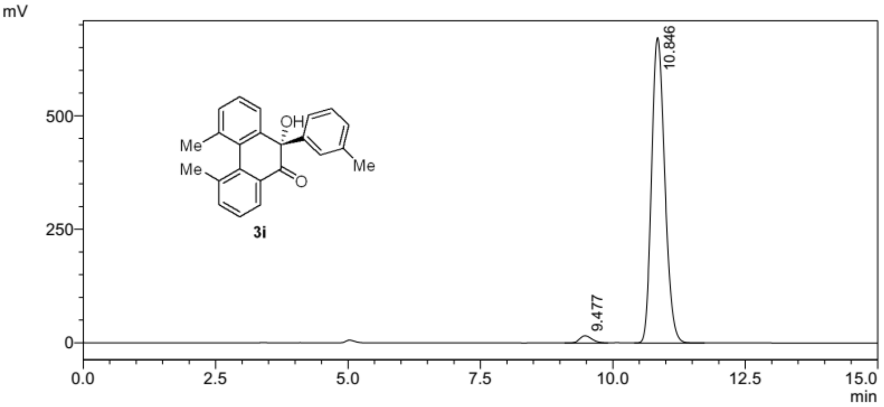

<Peak Table>

Chiralcel AD-H, isopropanol/hexane = 10:90, flow: 1.0 mL/min, λ = 254 nm.

Peak#	Ret. Time	Area	Height	Conc.	Unit	Mark	Name
1	9.477	256485	15774	2.155			
2	10.846	11646852	671832	97.845			
Total		11903337	687605				

<Chromatogram>

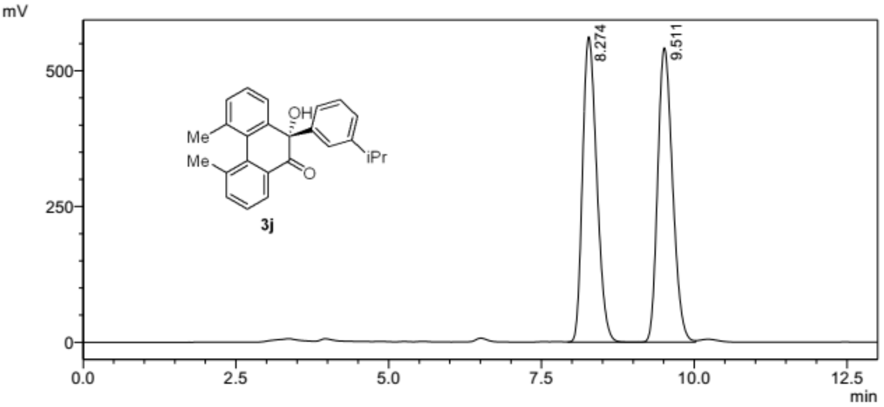

<Peak Table>

Chiralcel AD-H, isopropanol/hexane = 10:90, flow: 1.0 mL/min, λ = 254 nm.

Peak#	Ret. Time	Area	Height	Conc.	Unit	Mark	Name
1	8.274	9102693	562097	49.727			
2	9.511	9202468	542205	50.273			
Total		18305162	1104302				

<Chromatogram>

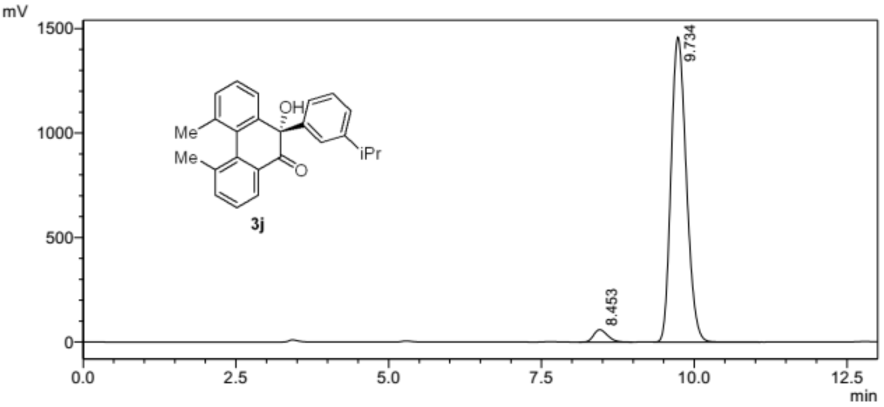

<Peak Table>

Chiralcel AD-H, isopropanol/hexane = 10:90, flow: 1.0 mL/min, λ = 254 nm.

Peak#	Ret. Time	Area	Height	Conc.	Unit	Mark	Name
1	8.453	944028	59266	3.580			
2	9.734	25428911	1459278	96.420			
Total		26372939	1518544				

<Chromatogram>

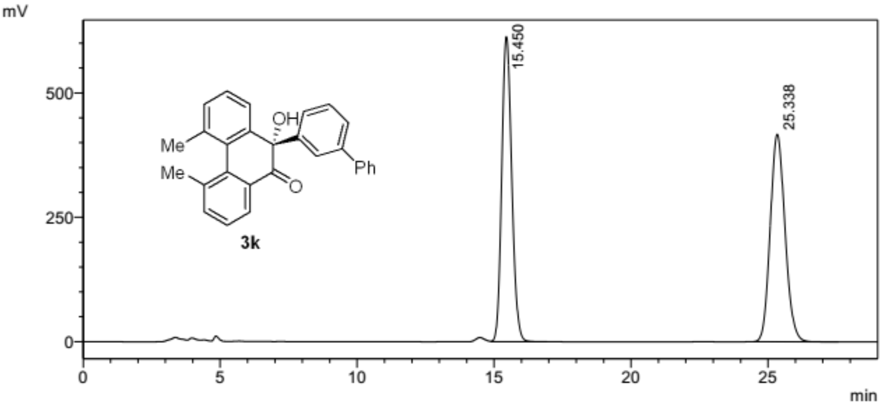

<Peak Table>

Chiralcel AD-H, isopropanol/hexane = 10:90, flow: 1.0 mL/min, λ = 254 nm.

Peak#	Ret. Time	Area	Height	Conc.	Unit	Mark	Name
1	15.450	15560412	612752	49.800			
2	25.338	15685273	417104	50.200			
Total		31245684	1029856				

<Chromatogram>

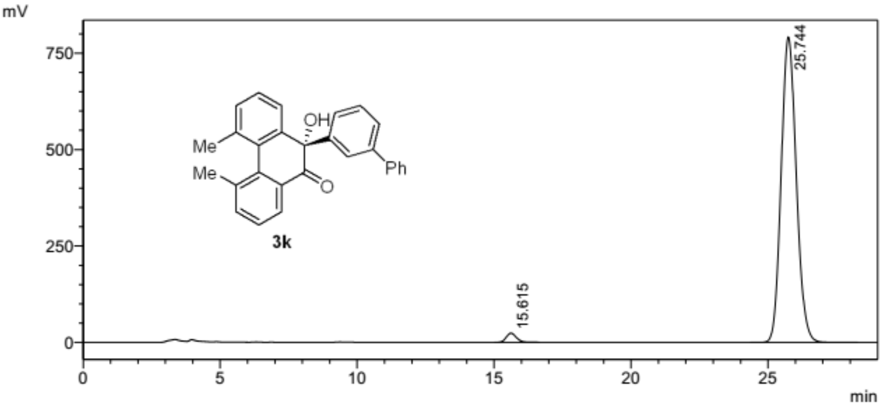

<Peak Table>

Chiralcel AD-H, isopropanol/hexane = 10:90, flow: 1.0 mL/min, λ = 254 nm.

Peak#	Ret. Time	Area	Height	Conc.	Unit	Mark	Name
1	15.615	617625	23827	1.990			
2	25.744	30412308	791355	98.010			
Total		31029933	815182				

<Chromatogram>

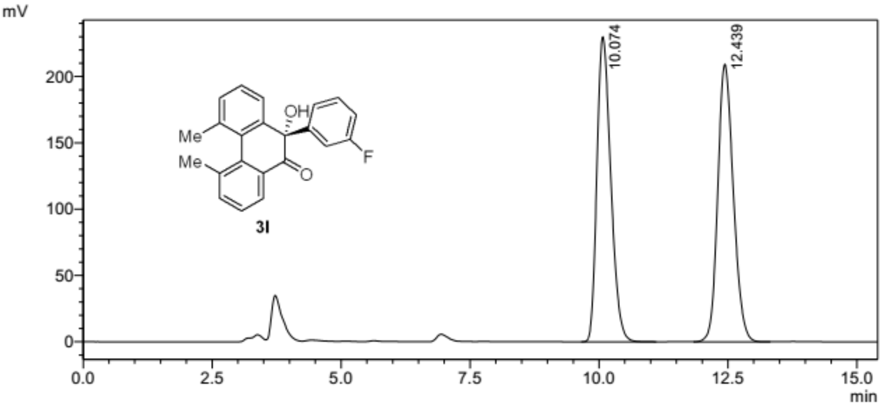

<Peak Table>

Chiralcel AD-H, isopropanol/hexane = 10:90, flow: 1.0 mL/min, λ = 254 nm.

Peak#	Ret. Time	Area	Height	Conc.	Unit	Mark	Name
1	10.074	4298902	229770	49.407			
2	12.439	4402183	209184	50.593			
Total		8701085	438954				

<Chromatogram>

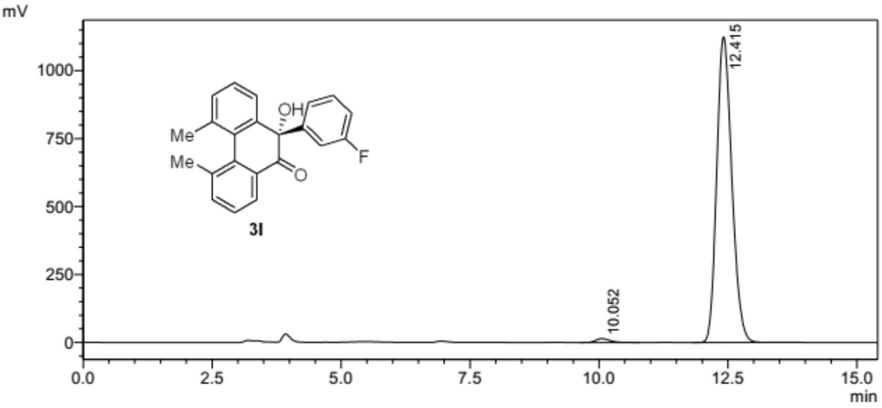

<Peak Table>

Chiralcel AD-H, isopropanol/hexane = 10:90, flow: 1.0 mL/min, λ = 254 nm.

Peak#	Ret. Time	Area	Height	Conc.	Unit	Mark	Name
1	10.052	265587	14154	1.145			
2	12.415	22931541	1123946	98.855			
Total		23197128	1138100				

<Chromatogram>

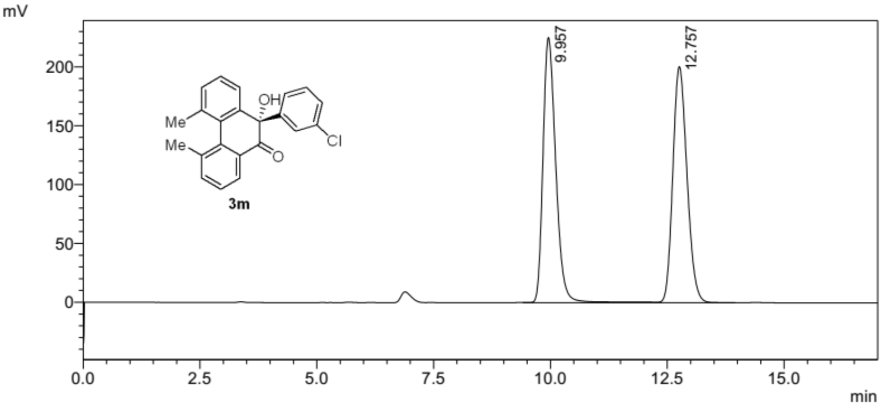

<Peak Table>

Chiralcel AD-H, isopropanol/hexane = 10:90, flow: 1.0 mL/min, λ = 254 nm.

Peak#	Ret. Time	Area	Height	Conc.	Unit	Mark	Name
1	9.957	4298892	225184	50.425			
2	12.757	4226371	200566	49.575			
Total		8525263	425750				

<Chromatogram>

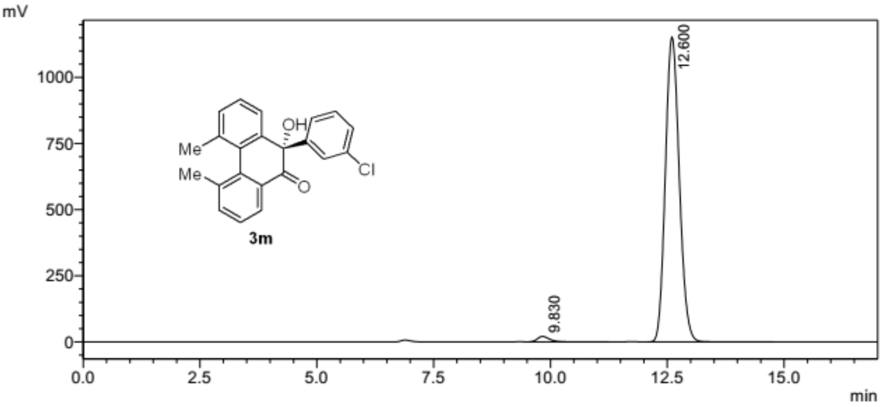

<Peak Table>

Chiralcel AD-H, isopropanol/hexane = 10:90, flow: 1.0 mL/min, λ = 254 nm.

Peak#	Ret. Time	Area	Height	Conc.	Unit	Mark	Name
1	9.830	385557	20797	1.588			
2	12.600	23896241	1152071	98.412			
Total		24281798	1172867				

<Chromatogram>

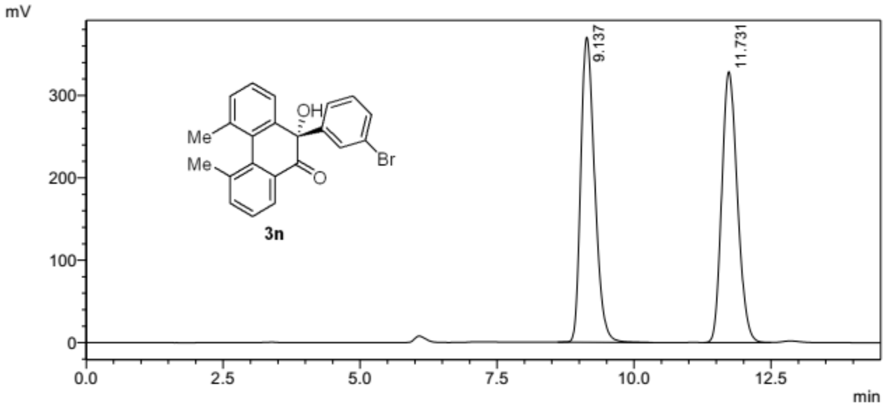

<Peak Table>

Chiralcel AD-H, isopropanol/hexane = 13:87, flow: 1.0 mL/min, λ = 254 nm.

Peak#	Ret. Time	Area	Height	Conc.	Unit	Mark	Name
1	9.137	6543122	370086	50.049			
2	11.731	6530288	328806	49.951			
Total		13073411	698892				

<Chromatogram>

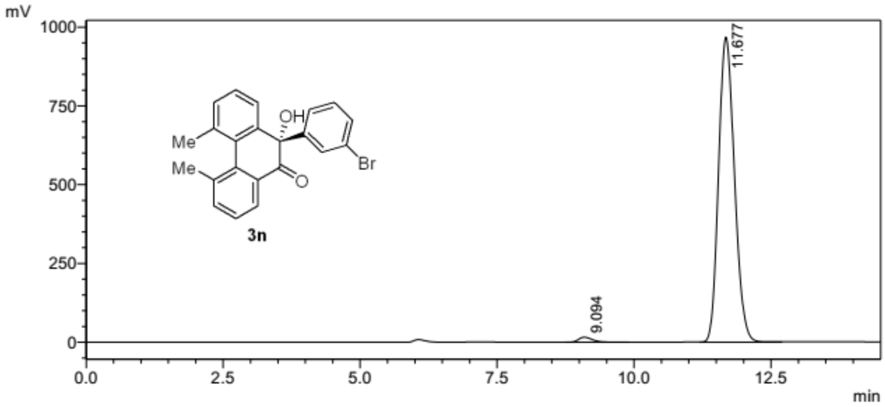

<Peak Table>

Chiralcel AD-H, isopropanol/hexane = 13:87, flow: 1.0 mL/min, λ = 254 nm.

Peak#	Ret. Time	Area	Height	Conc.	Unit	Mark	Name
1	9.094	281904	15993	1.419			
2	11.677	19583820	967460	98.581			
Total		19865723	983453				

<Chromatogram>

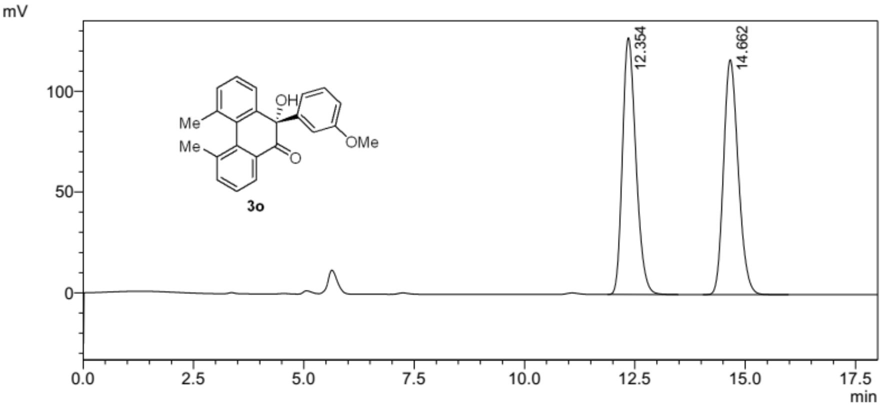

<Peak Table>

Chiralcel AD-H, isopropanol/hexane = 15:85, flow: 1.0 mL/min, λ = 254 nm.

Peak#	Ret. Time	Area	Height	Conc.	Unit	Mark	Name
1	12.354	2704217	127389	50.032			
2	14.662	2700806	116636	49.968			
Total		5405023	244025				

<Chromatogram>

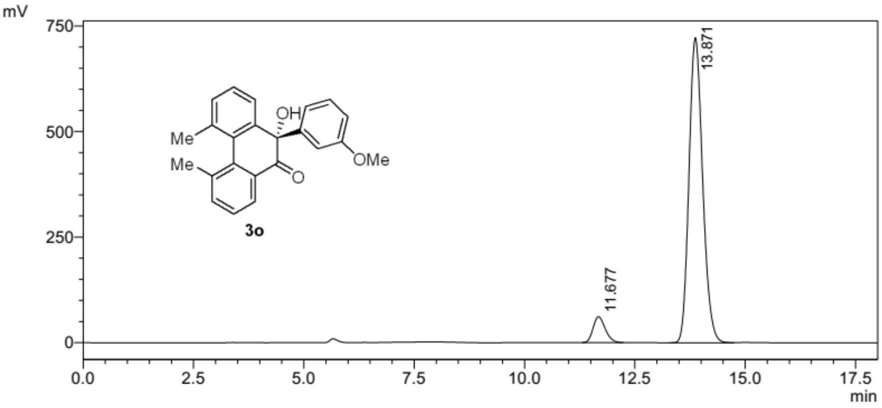

<Peak Table>

Chiralcel AD-H, isopropanol/hexane = 15:85, flow: 1.0 mL/min, λ = 254 nm.

Peak#	Ret. Time	Area	Height	Conc.	Unit	Mark	Name
1	11.677	1177783	61360	7.029			
2	13.871	15577283	721789	92.971			
Total		16755067	783149				

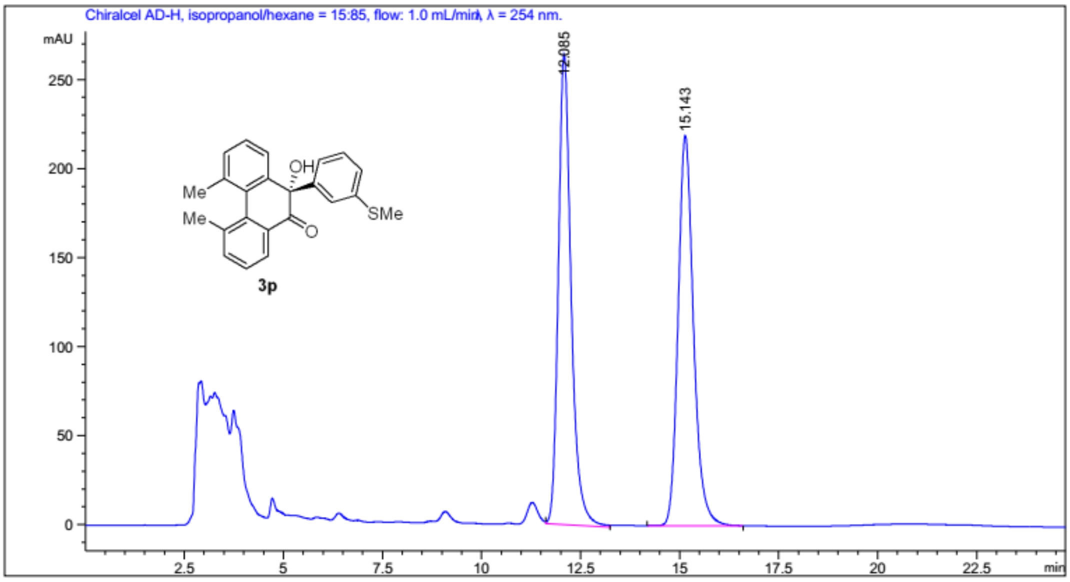

Peak #	RetTime [min]	Type	Width [min]	Area [mAU*s]	Height [mAU]	Area %
1	12.085	MM	0.3739	5922.39990	264.00790	49.9545
2	15.143	BB	0.4140	5933.19873	219.02982	50.0455

Totals : 1.18556e4 483.03772

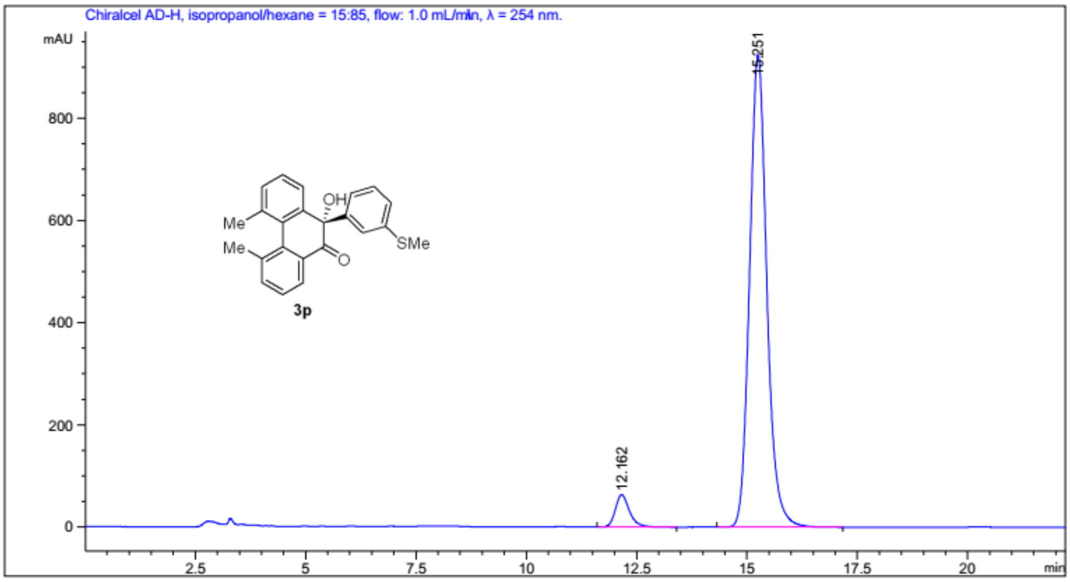

Peak #	RetTime [min]	Type	Width [min]	Area [mAU*s]	Height [mAU]	Area %
1	12.162	BB	0.3374	1413.79675	63.59253	5.3341
2	15.251	BB	0.4166	2.50909e4	924.49713	94.6659

Totals : 2.65047e4 988.08966

<Chromatogram>

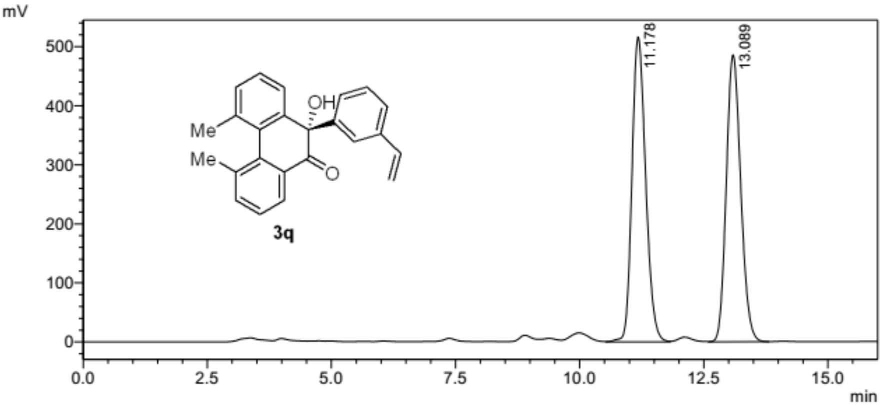

<Peak Table>

Chiralcel AD-H, isopropanol/hexane = 10:90, flow: 1.0 mL/min, λ = 254 nm.

Peak#	Ret. Time	Area	Height	Conc.	Unit	Mark	Name
1	11.178	9914469	515863	50.201			
2	13.089	9835225	485681	49.799			
Total		19749694	1001543				

<Chromatogram>

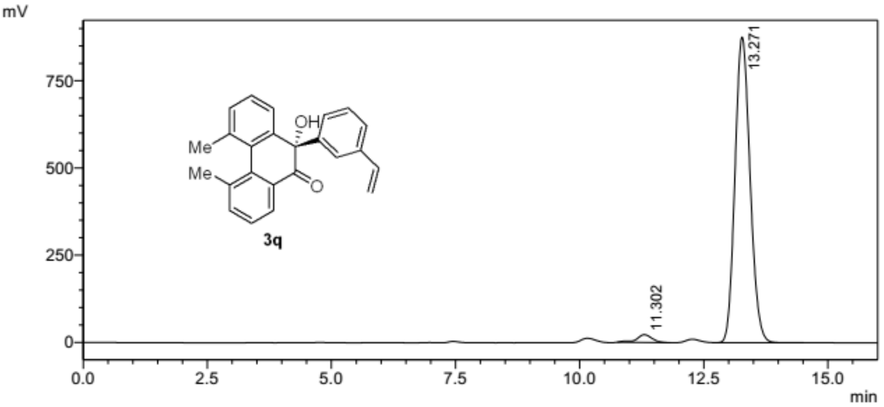

<Peak Table>

Chiralcel AD-H, isopropanol/hexane = 10:90, flow: 1.0 mL/min, λ = 254 nm.

Peak#	Ret. Time	Area	Height	Conc.	Unit	Mark	Name
1	11.302	478961	22264	2.524			
2	13.271	18498606	875257	97.476			
Total		18977568	897521				

<Chromatogram>

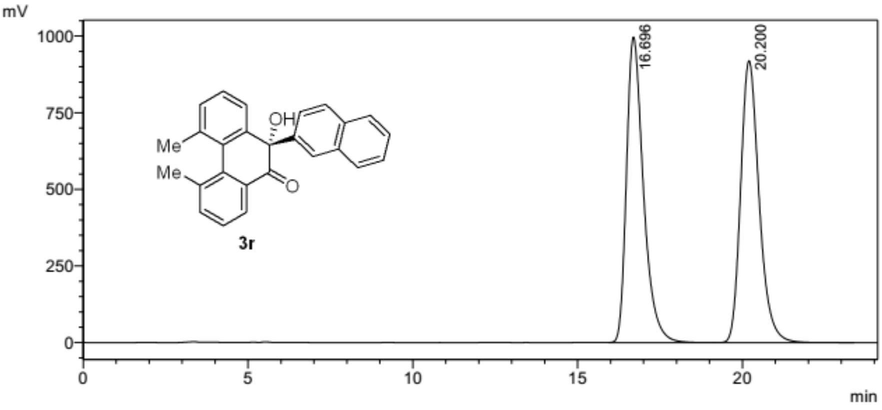

<Peak Table>

Chiralcel AD-H, isopropanol/hexane = 10:90, flow: 1.0 mL/min, λ = 254 nm.

Peak#	Ret. Time	Area	Height	Conc.	Unit	Mark	Name
1	16.696	35663092	996531	49.936			
2	20.200	35754463	919613	50.064			
Total		71417555	1916144				

<Chromatogram>

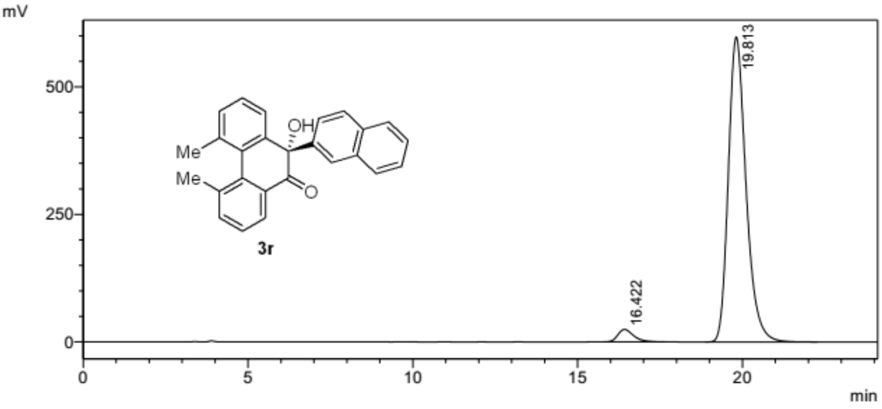

<Peak Table>

Chiralcel AD-H, isopropanol/hexane = 10:90, flow: 1.0 mL/min, λ = 254 nm.

Peak#	Ret. Time	Area	Height	Conc.	Unit	Mark	Name
1	16.422	814529	24458	3.515			
2	19.813	22358657	597909	96.485			
Total		23173186	622367				

<Chromatogram>

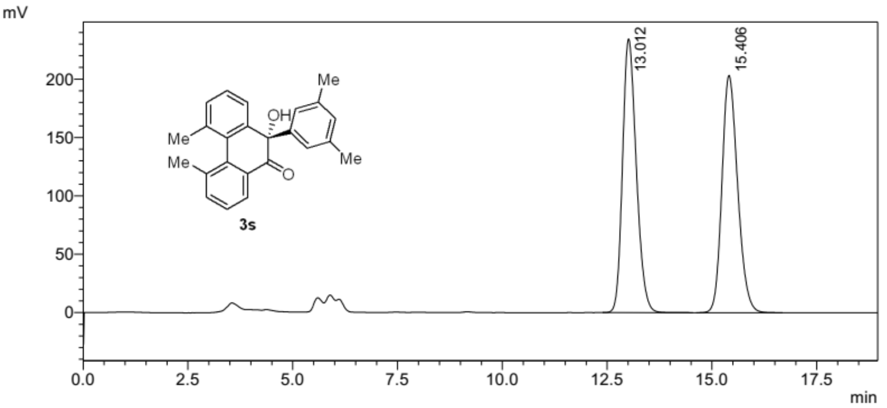

<Peak Table>

Chiralcel ID, isopropanol/hexane = 10:90, flow: 1.0 mL/min, λ = 254 nm.

Peak#	Ret. Time	Area	Height	Conc.	Unit	Mark	Name
1	13.012	5350513	234363	50.235			
2	15.406	5300555	203253	49.765			
Total		10651069	437615				

<Chromatogram>

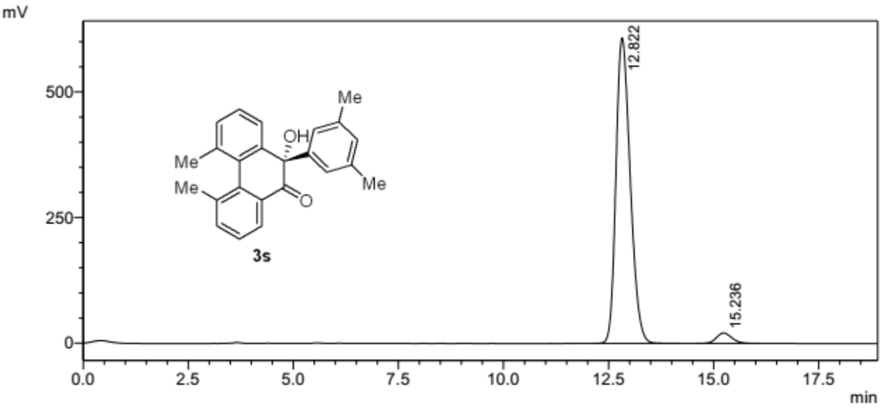

<Peak Table>

Chiralcel ID, isopropanol/hexane = 10:90, flow: 1.0 mL/min, λ = 254 nm.

Peak#	Ret. Time	Area	Height	Conc.	Unit	Mark	Name
1	12.822	14359567	607985	96.409			
2	15.236	534917	20807	3.591			
Total		14894484	628792				

<Chromatogram>

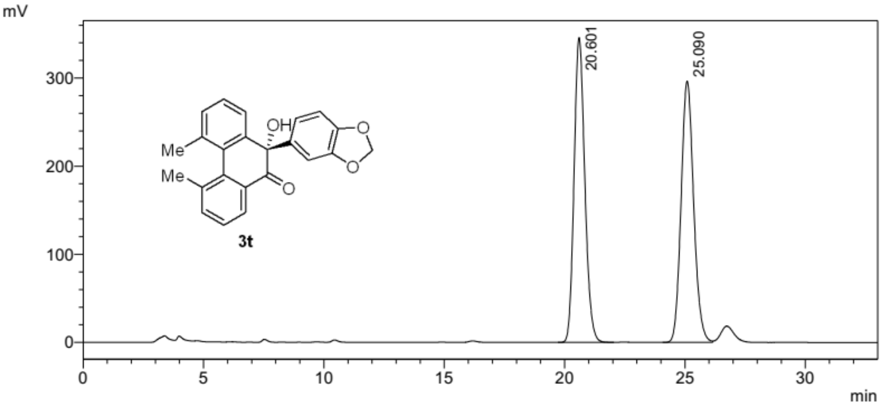

<Peak Table>

Chiralcel AD-H, isopropanol/hexane = 10:90, flow: 1.0 mL/min, λ = 254 nm.

Peak#	Ret. Time	Area	Height	Conc.	Unit	Mark	Name
1	20.601	10745547	345842	49.939			
2	25.090	10771677	296604	50.061			
Total		21517224	642446				

<Chromatogram>

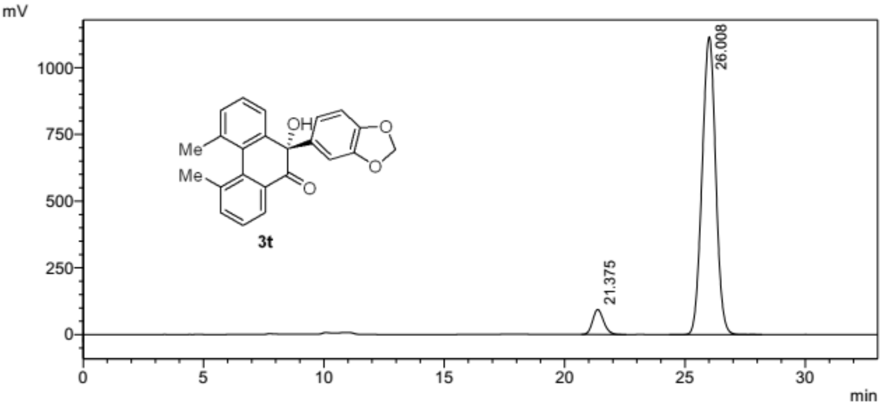

<Peak Table>

Chiralcel AD-H, isopropanol/hexane = 10:90, flow: 1.0 mL/min, λ = 254 nm.

Peak#	Ret. Time	Area	Height	Conc.	Unit	Mark	Name
1	21.375	2970869	93429	6.365			
2	26.008	43706134	1114813	93.635			
Total		46677003	1208242				

<Chromatogram>

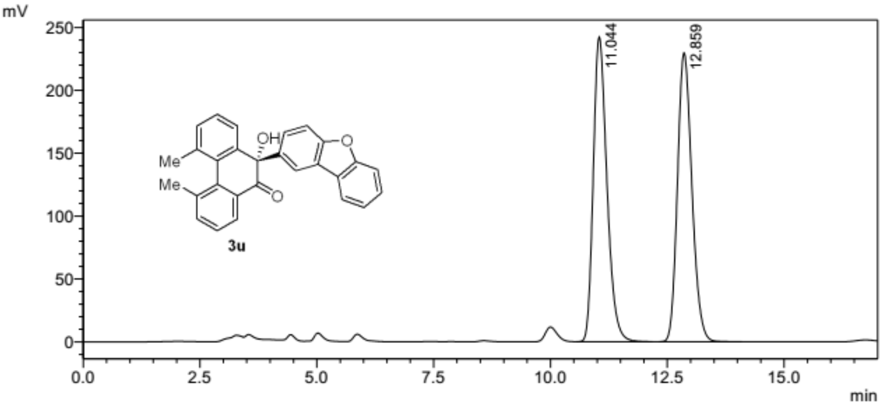

<Peak Table>

Chiralcel AD-H, isopropanol/hexane = 20:80, flow: 1.0 mL/min, λ = 254 nm.

Peak#	Ret. Time	Area	Height	Conc.	Unit	Mark	Name
1	11.044	4927272	242317	49.936			
2	12.859	4939977	229686	50.064			
Total		9867249	472003				

<Chromatogram>

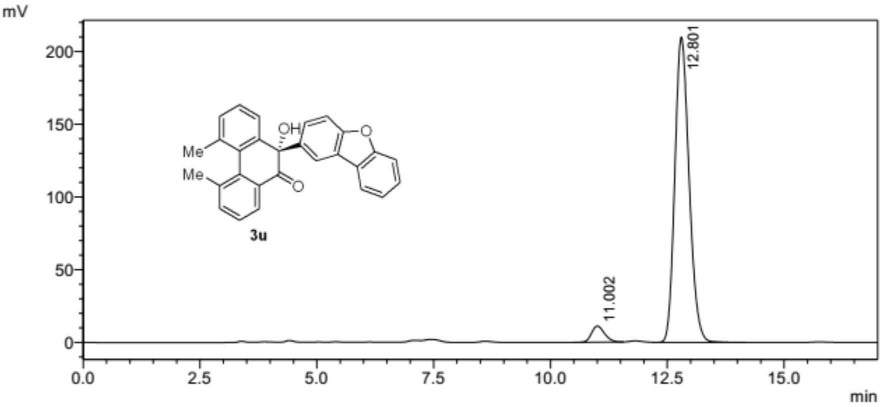

<Peak Table>

Chiralcel AD-H, isopropanol/hexane = 20:80, flow: 1.0 mL/min, λ = 254 nm.

Peak#	Ret. Time	Area	Height	Conc.	Unit	Mark	Name
1	11.002	214294	11084	4.583			
2	12.801	4461861	209682	95.417			
Total		4676155	220766				

<Chromatogram>

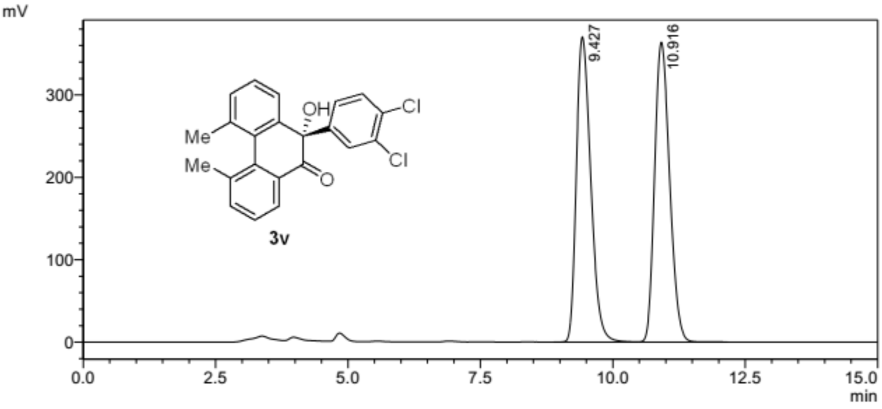

<Peak Table>

Chiralcel AD-H, isopropanol/hexane = 10:90, flow: 1.0 mL/min, λ = 254 nm.

Peak#	Ret. Time	Area	Height	Conc.	Unit	Mark	Name
1	9.427	7011246	370211	49.421			
2	10.916	7175518	363671	50.579			
Total		14186765	733882				

<Chromatogram>

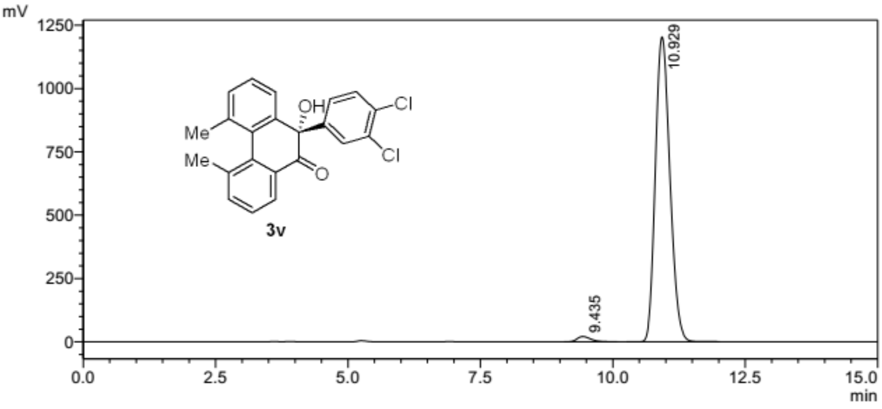

<Peak Table>

Chiralcel AD-H, isopropanol/hexane = 10:90, flow: 1.0 mL/min, λ = 254 nm.

Peak#	Ret. Time	Area	Height	Conc.	Unit	Mark	Name
1	9.435	367589	20674	1.568			
2	10.929	23073075	1202724	98.432			
Total		23440664	1223398				

<Chromatogram>

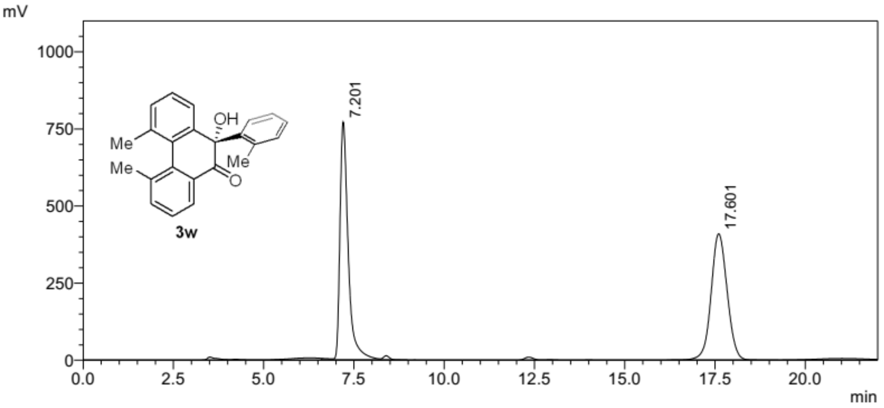

<Peak Table>

Chiralcel IC, isopropanol/hexane = 10:90, flow: 1.0 mL/min, λ = 254 nm.

Peak#	Ret. Time	Area	Height	Conc.	Unit	Mark	Name
1	7.201	12532374	771720	49.755			
2	17.601	12655982	409539	50.245			
Total		25188356	1181260				

<Chromatogram>

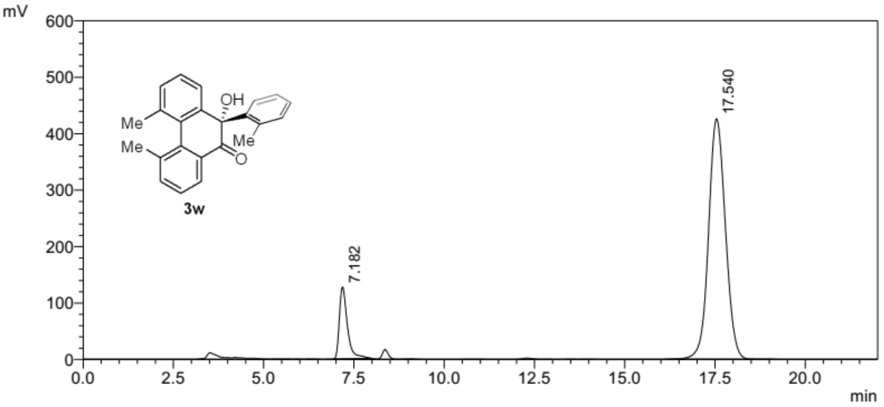

<Peak Table>

Chiralcel IC, isopropanol/hexane = 10:90, flow: 1.0 mL/min, λ = 254 nm.

Peak#	Ret. Time	Area	Height	Conc.	Unit	Mark	Name
1	7.182	1958172	127177	12.402			
2	17.540	13831155	425964	87.598			
Total		15789328	553141				

<Chromatogram>

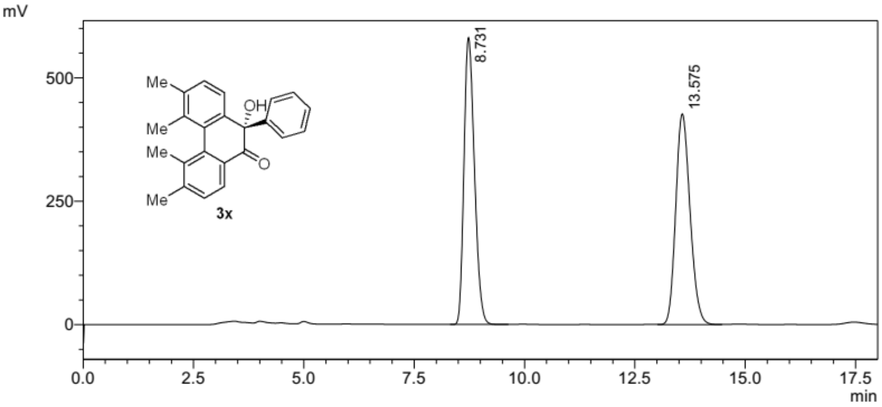

<Peak Table>

Chiralcel AD-H, isopropanol/hexane = 10:90, flow: 1.0 mL/min, λ = 254 nm.

Peak#	Ret. Time	Area	Height	Conc.	Unit	Mark	Name
1	8.731	9594857	580960	49.924			
2	13.575	9624053	426737	50.076			
Total		19218910	1007697				

<Chromatogram>

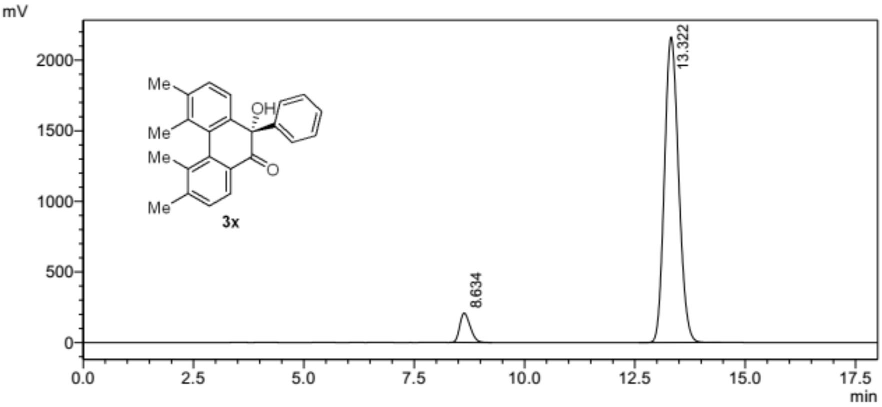

<Peak Table>

Chiralcel AD-H, isopropanol/hexane = 10:90, flow: 1.0 mL/min, λ = 254 nm.

Peak#	Ret. Time	Area	Height	Conc.	Unit	Mark	Name
1	8.634	3408136	207891	6.595			
2	13.322	48267896	2163011	93.405			
Total		51676031	2370902				

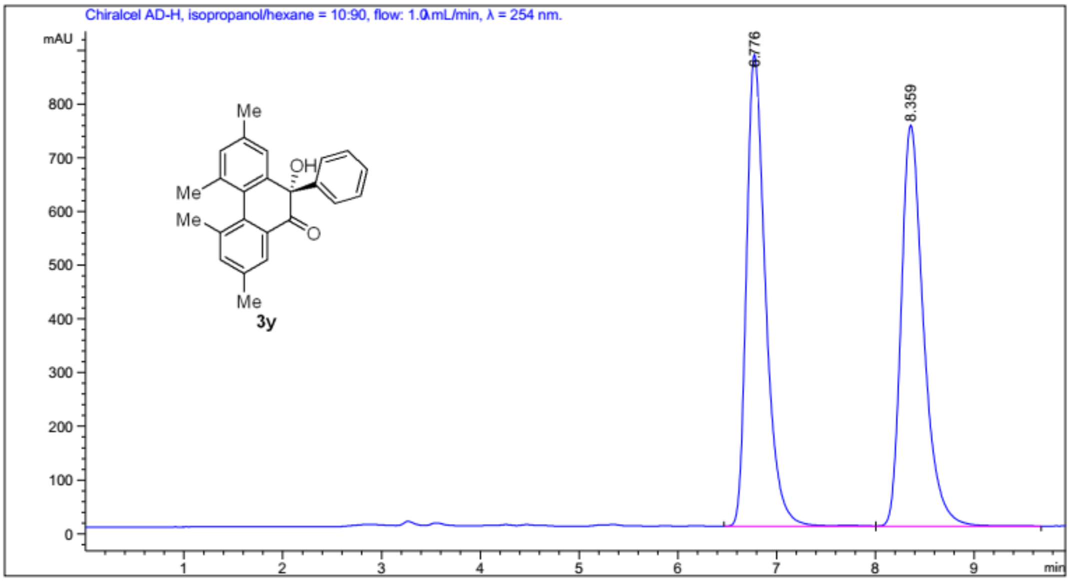

Peak #	RetTime [min]	Type	Width [min]	Area [mAU*s]	Height [mAU]	Area %
1	6.776	BV R	0.2035	1.17378e4	878.93933	50.0634
2	8.359	VB	0.2389	1.17081e4	746.88080	49.9366

Totals : 2.34459e4 1625.82013

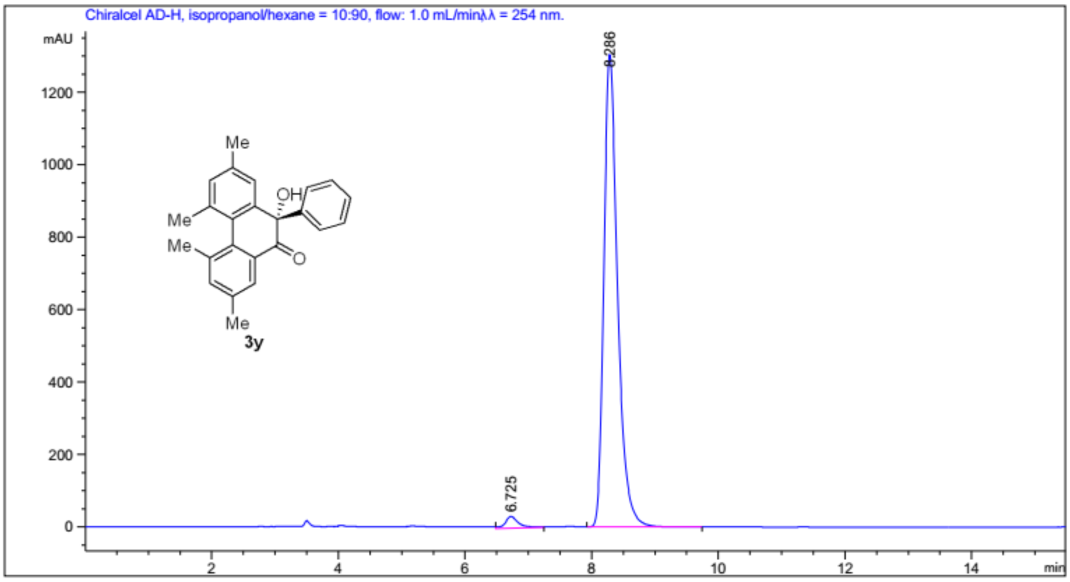

Peak #	RetTime [min]	Type	Width [min]	Area [mAU*s]	Height [mAU]	Area %
1	6.725	MM	0.2390	462.53421	32.25896	2.3027
2	8.286	VB	0.2296	1.96239e4	1303.90710	97.6973

Totals : 2.00864e4 1336.16607

<Chromatogram>

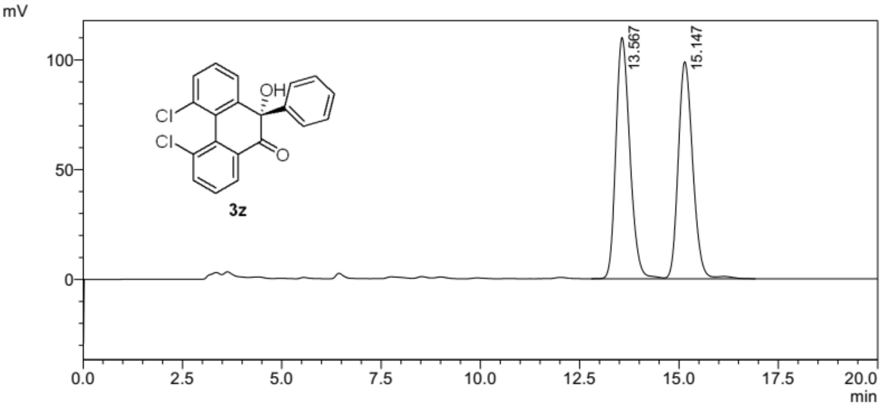

<Peak Table>

Chiralcel AD-H, isopropanol/hexane = 10:90, flow: 1.0 mL/min, λ = 254 nm.

Peak#	Ret. Time	Area	Height	Conc.	Unit	Mark	Name
1	13.567	2691615	109791	51.684			
2	15.147	2516179	98791	48.316			
Total		5207794	208582				

<Chromatogram>

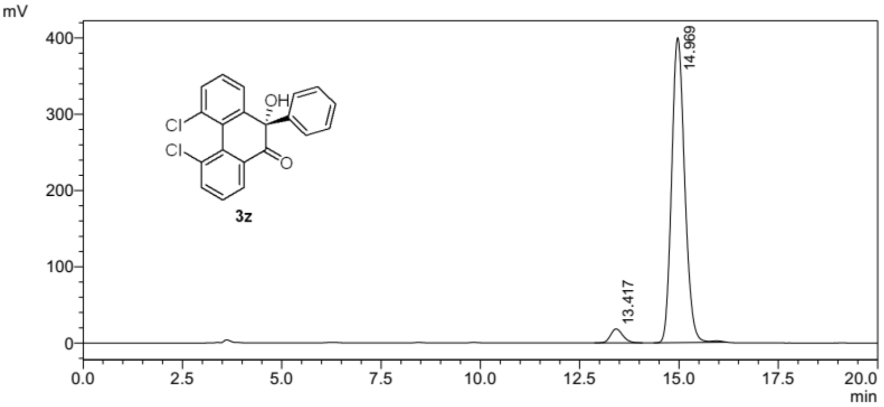

<Peak Table>

Chiralcel AD-H, isopropanol/hexane = 10:90, flow: 1.0 mL/min, λ = 254 nm.

Peak#	Ret. Time	Area	Height	Conc.	Unit	Mark	Name
1	13.417	386207	18182	4.045			
2	14.969	9162406	399801	95.955			
Total		9548612	417983				

<Chromatogram>

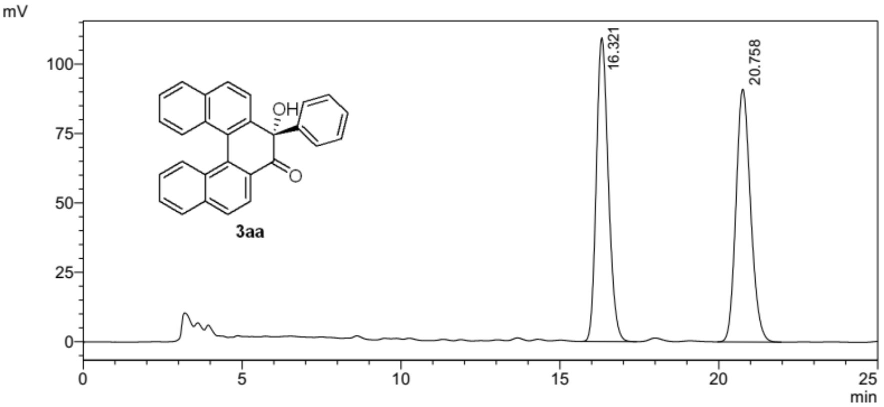

<Peak Table>

Chiralcel AD-H, isopropanol/hexane = 10:90, flow: 1.0 mL/min, λ = 254 nm.

Peak#	Ret. Time	Area	Height	Conc.	Unit	Mark	Name
1	16.321	2876318	109325	49.658			
2	20.758	2915883	91025	50.342			
Total		5792201	200350				

<Chromatogram>

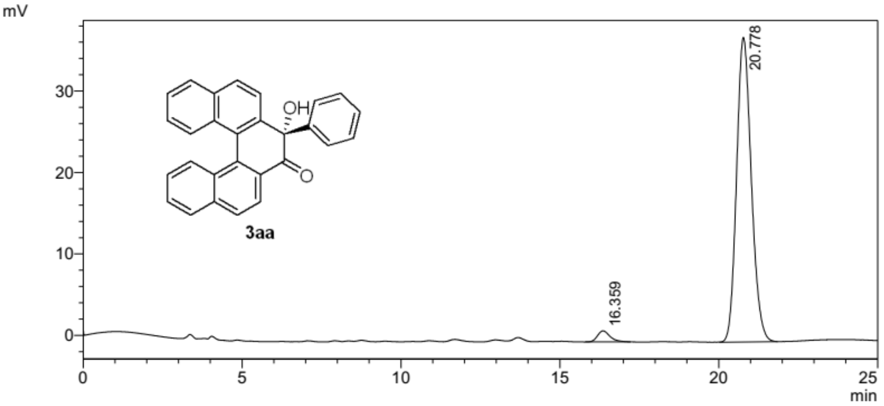

<Peak Table>

Chiralcel AD-H, isopropanol/hexane = 10:90, flow: 1.0 mL/min, λ = 254 nm.

Peak#	Ret. Time	Area	Height	Conc.	Unit	Mark	Name
1	16.359	37417	1362	3.012			
2	20.778	1204913	37384	96.988			
Total		1242329	38746				

<Chromatogram>

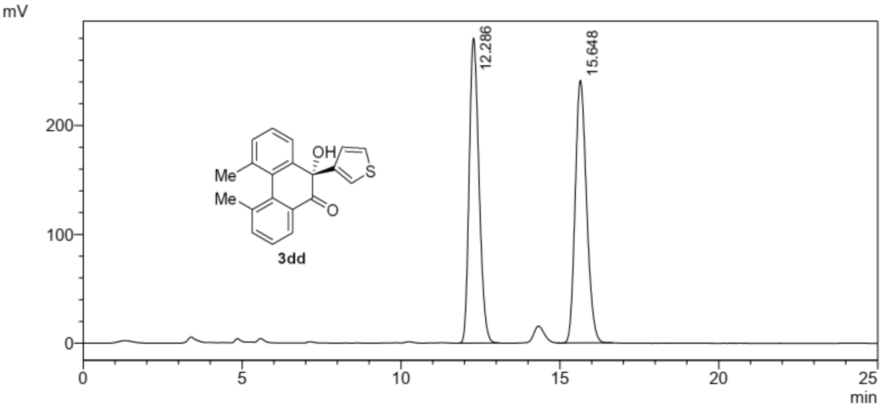

<Peak Table>

Chiralcel AD-H, isopropanol/hexane = 10:90, flow: 1.0 mL/min, λ = 254 nm.

Peak#	Ret. Time	Area	Height	Conc.	Unit	Mark	Name
1	12.286	5996468	280105	50.009			
2	15.648	5994208	241116	49.991			
Total		11990676	521220				

<Chromatogram>

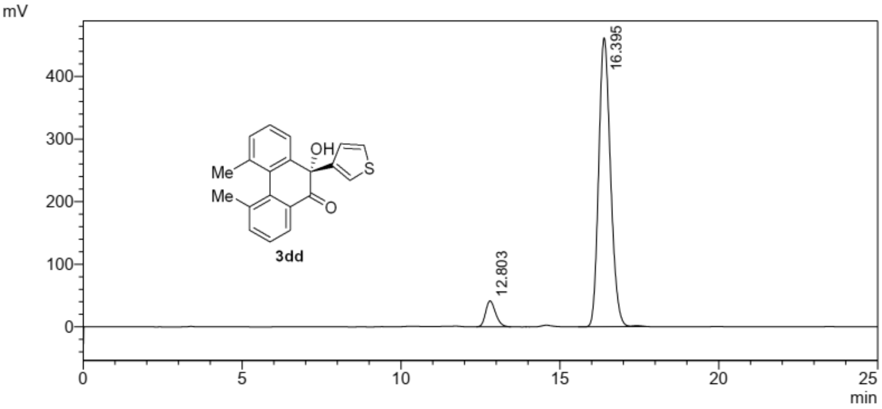

<Peak Table>

Chiralcel AD-H, isopropanol/hexane = 10:90, flow: 1.0 mL/min, λ = 254 nm.

Peak#	Ret. Time	Area	Height	Conc.	Unit	Mark	Name
1	12.803	902255	41355	7.020			
2	16.395	11950825	461196	92.980			
Total		12853081	502552				

<Chromatogram>

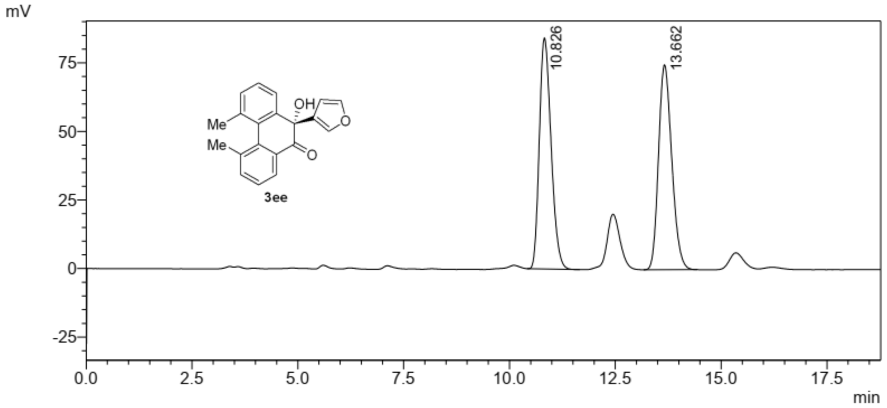

<Peak Table>

Chiralcel AD-H, isopropanol/hexane = 10:90, flow: 1.0 mL/min, λ = 254 nm.

Peak#	Ret. Time	Area	Height	Conc.	Unit	Mark	Name
1	10.826	1627027	84215	49.970			
2	13.662	1628971	74675	50.030			
Total		3255998	158890				

<Chromatogram>

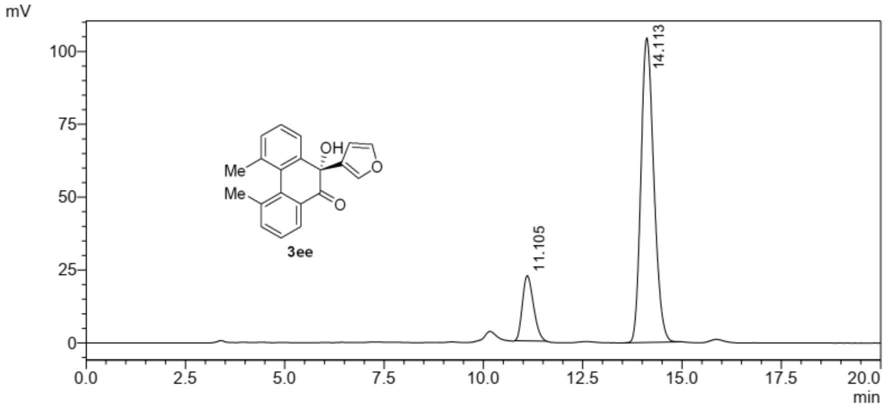

<Peak Table>

Chiralcel AD-H, isopropanol/hexane = 10:90, flow: 1.0 mL/min, λ = 254 nm.

Peak#	Ret. Time	Area	Height	Conc.	Unit	Mark	Name
1	11.105	434324	22383	15.523			
2	14.113	2363704	104451	84.477			
Total		2798028	126834				

<Chromatogram>

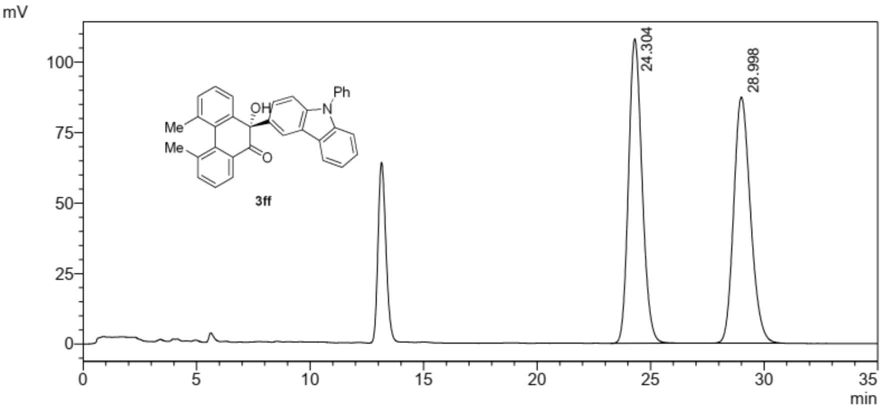

<Peak Table>

Chiralcel AD-H, isopropanol/hexane = 10:90, flow: 1.0 mL/min, λ = 254 nm.

Peak#	Ret. Time	Area	Height	Conc.	Unit	Mark	Name
1	24.304	4472296	107931	49.939			
2	28.998	4483179	87287	50.061			
Total		8955475	195217				

<Chromatogram>

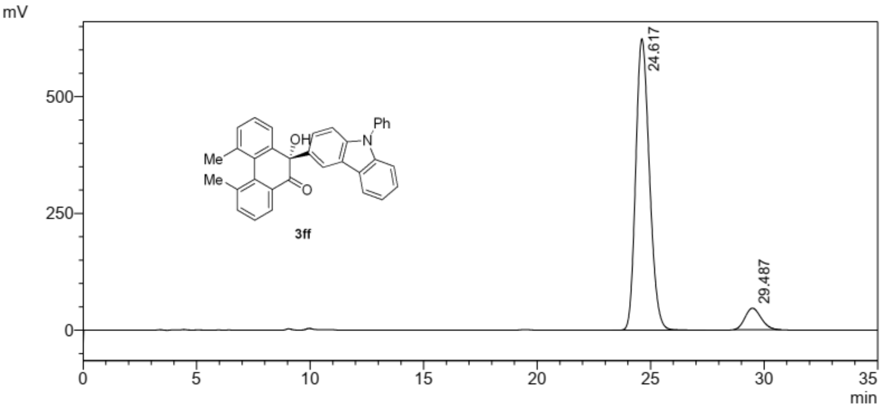

<Peak Table>

Chiralcel AD-H, isopropanol/hexane = 10:90, flow: 1.0 mL/min, λ = 254 nm.

Peak#	Ret. Time	Area	Height	Conc.	Unit	Mark	Name
1	24.617	26147617	623767	91.995			
2	29.487	2275147	46064	8.005			
Total		28422764	669831				

<Chromatogram>

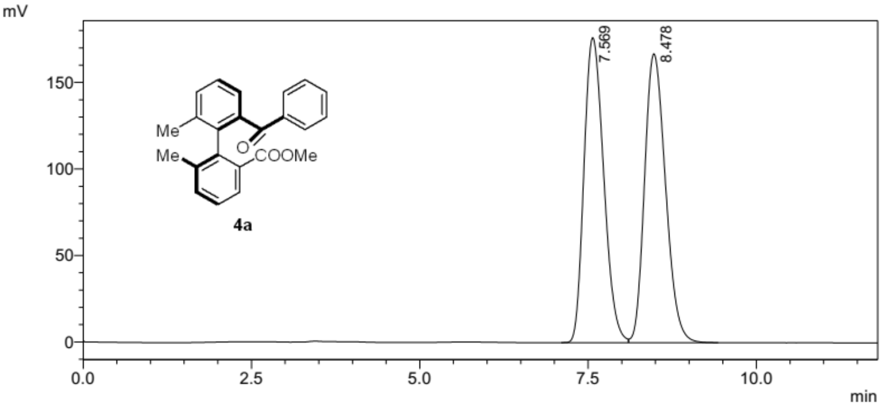

<Peak Table>

Chiralcel AD-H, isopropanol/hexane = 3:97, flow: 1.0 mL/min, λ = 254 nm.

Peak#	Ret. Time	Area	Height	Conc.	Unit	Mark	Name
1	7.569	3589733	176281	49.756			
2	8.478	3624988	166991	50.244			
Total		7214721	343272				

<Chromatogram>

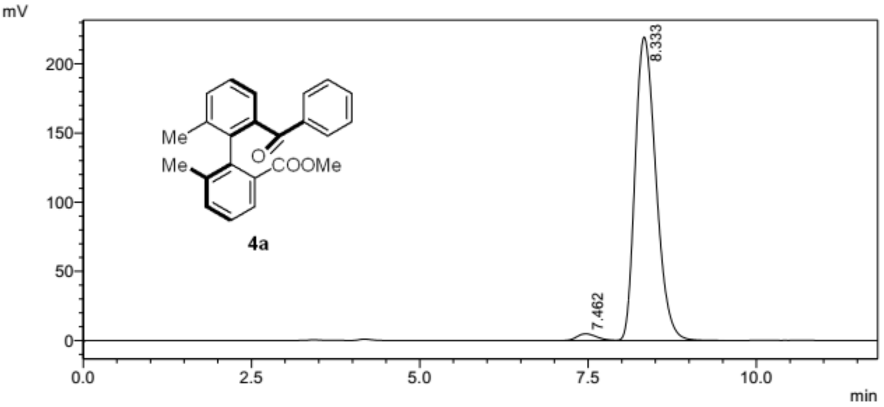

<Peak Table>

Chiralcel AD-H, isopropanol/hexane = 3:97, flow: 1.0 mL/min, λ = 254 nm.

Peak#	Ret. Time	Area	Height	Conc.	Unit	Mark	Name
1	7.462	96690	4825	2.009			
2	8.333	4715557	219372	97.991			
Total		4812247	224197				

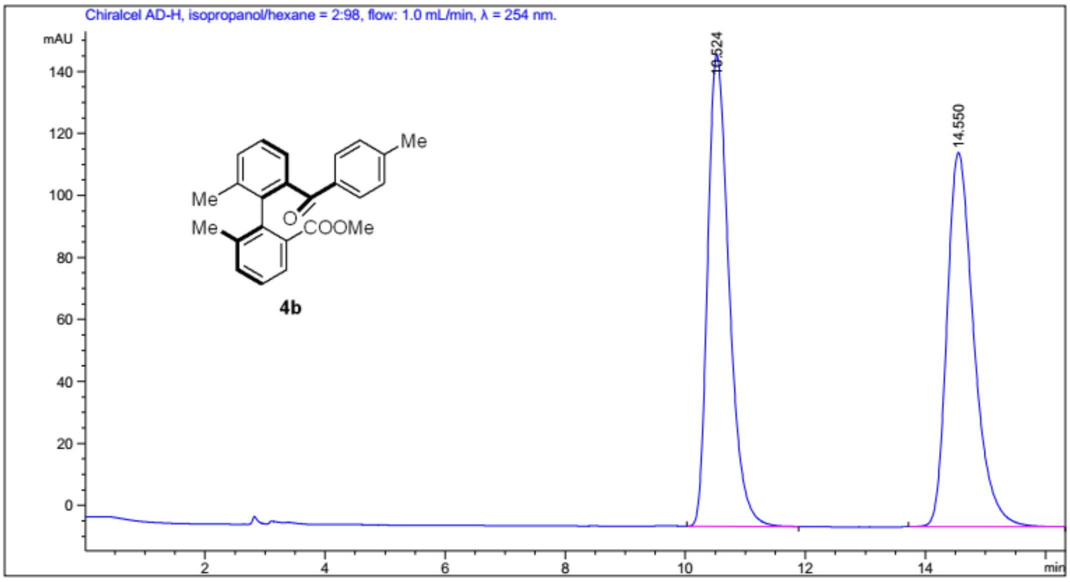

Peak #	RetTime [min]	Type	Width [min]	Area [mAU*s]	Height [mAU]	Area %
1	10.524	BB	0.3821	3758.02661	152.19878	50.0756
2	14.550	BBA	0.4781	3746.68286	120.73072	49.9244

Totals : 7504.70947 272.92950

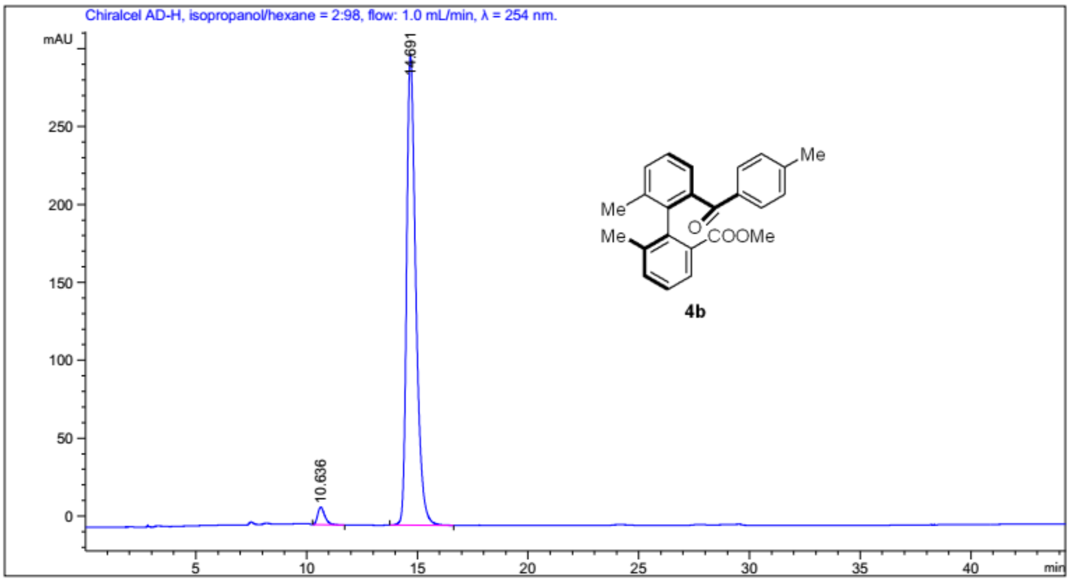

Peak #	RetTime [min]	Type	Width [min]	Area [mAU*s]	Height [mAU]	Area %
1	10.636	BB	0.3511	256.92700	11.30543	2.8647
2	14.691	BB	0.4388	8711.91504	301.77744	97.1353

Totals : 8968.84204 313.08286

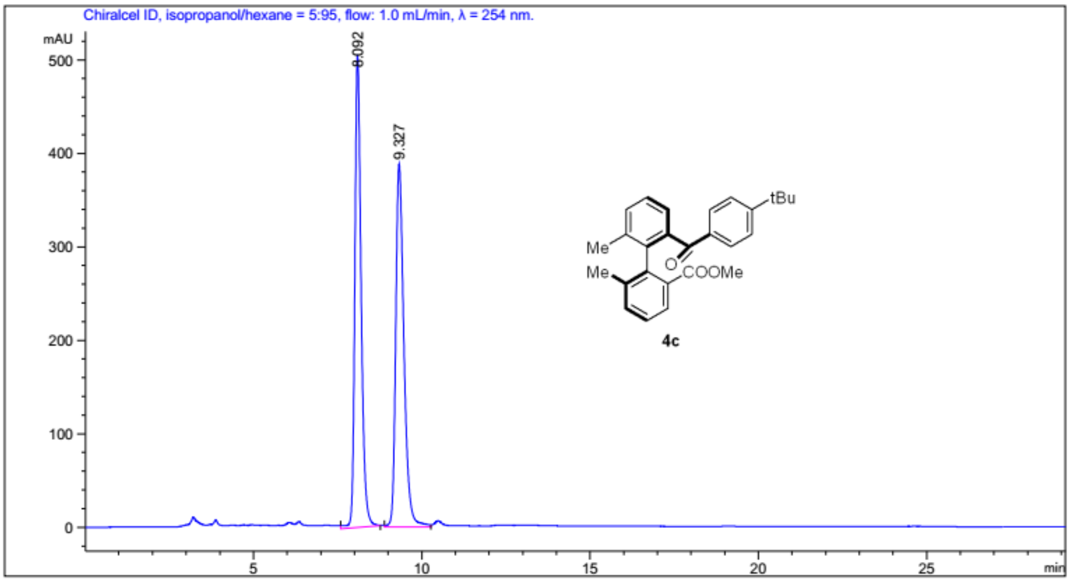

Peak #	RetTime [min]	Type	Width [min]	Area [mAU*s]	Height [mAU]	Area %
1	8.092	MM	0.2171	6587.91260	505.64874	49.8790
2	9.327	MM	0.2839	6619.88281	388.67978	50.1210

Totals : 1.32078e4 894.32852

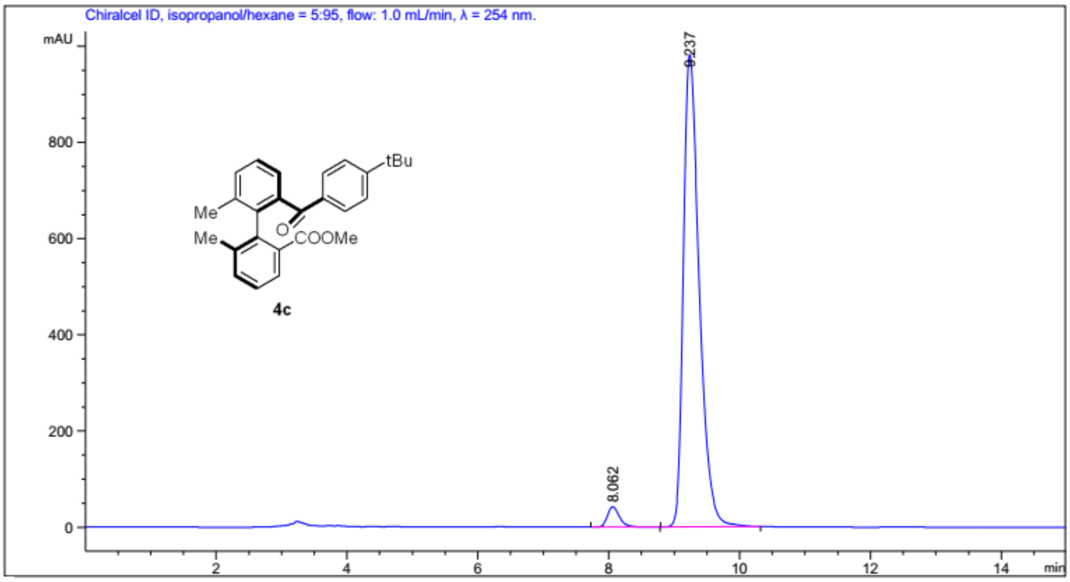

Peak #	RetTime [min]	Type	Width [min]	Area [mAU*s]	Height [mAU]	Area %
1	8.062	BB	0.1926	541.70007	42.52998	3.1764
2	9.237	BB	0.2581	1.65124e4	982.19525	96.8236

Totals : 1.70541e4 1024.72523

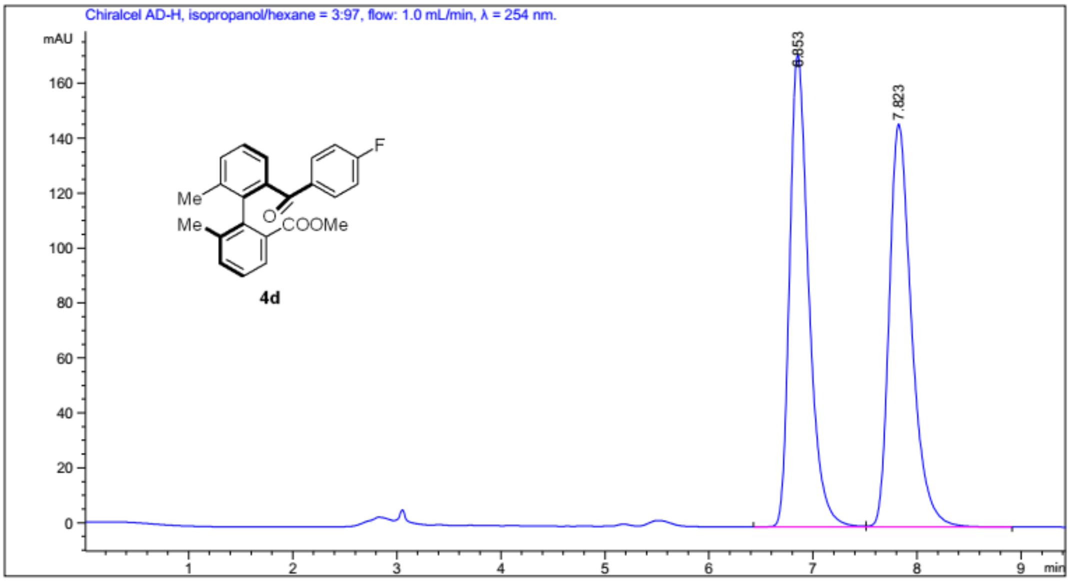

Peak #	RetTime [min]	Type	Width [min]	Area [mAU*s]	Height [mAU]	Area %
1	6.853	BV	0.1952	2199.60059	171.97940	50.0989
2	7.823	VB	0.2263	2190.91650	146.67395	49.9011

Totals : 4390.51709 318.65335

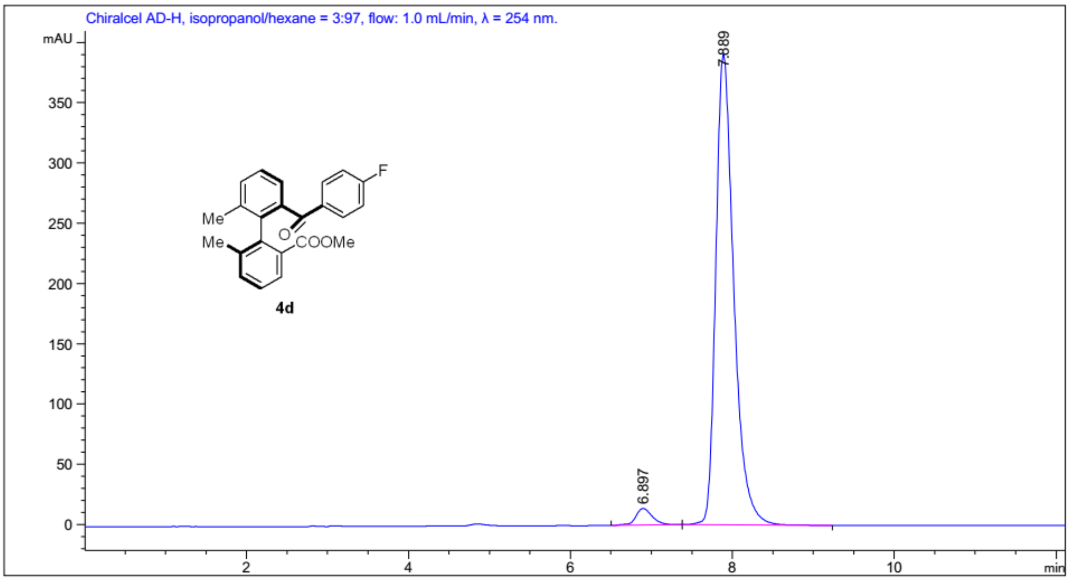

Peak #	RetTime [min]	Type	Width [min]	Area [mAU*s]	Height [mAU]	Area %
1	6.897	BB	0.2181	200.38676	13.90629	3.2185
2	7.889	BB	0.2340	6025.62354	390.46088	96.7815

Totals : 6226.01030 404.36717

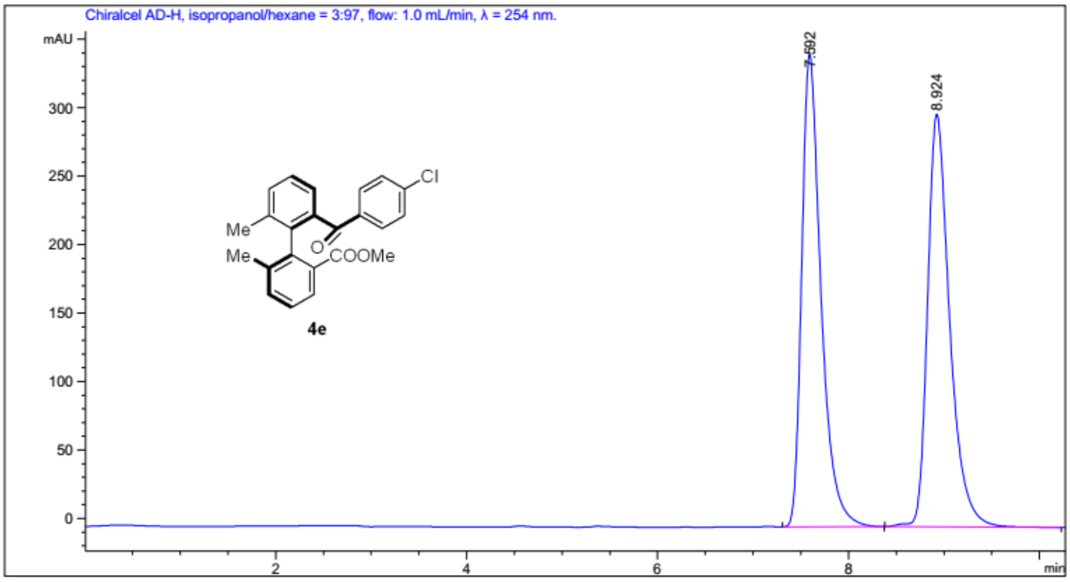

Peak #	RetTime [min]	Type	Width [min]	Area [mAU*s]	Height [mAU]	Area %
1	7.592	BB	0.2118	4843.98584	344.94876	49.9928
2	8.924	BBA	0.2436	4845.37256	301.29544	50.0072

Totals : 9689.35840 646.24420

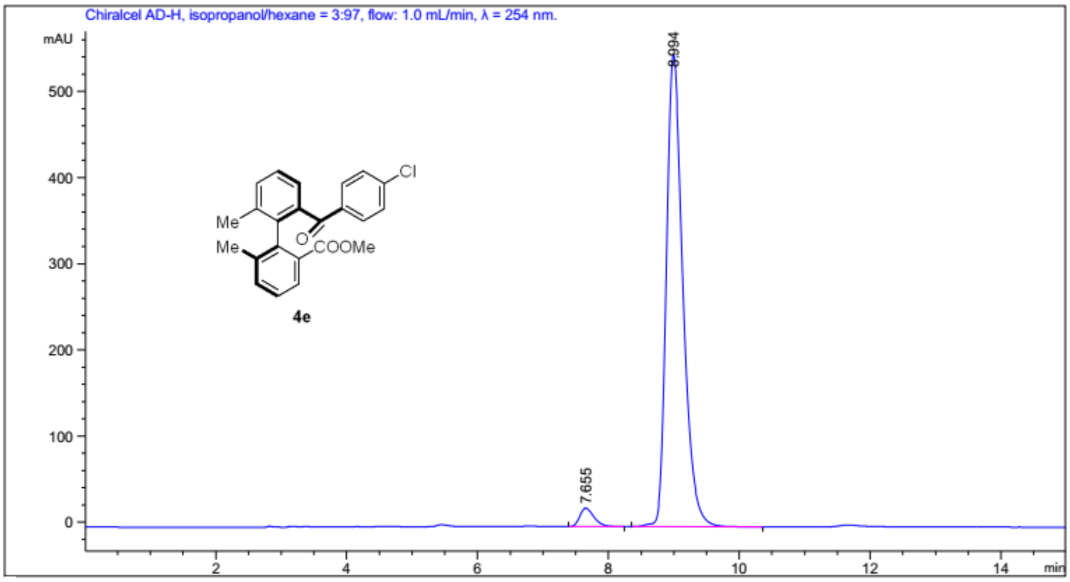

Peak #	RetTime [min]	Type	Width [min]	Area [mAU*s]	Height [mAU]	Area %
1	7.655	MM	0.2527	328.61826	21.67112	3.3103
2	8.994	BB	0.2684	9598.63281	547.88367	96.6897

Totals : 9927.25107 569.55479

<Chromatogram>

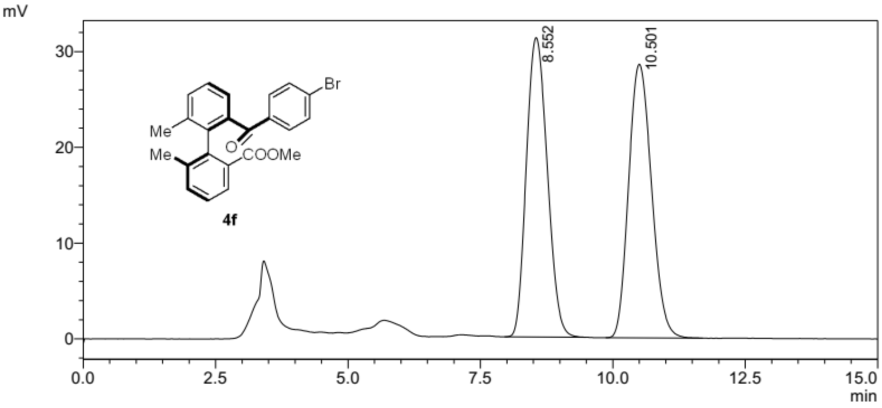

<Peak Table>

Chiralcel AD-H, isopropanol/hexane = 3:97, flow: 1.0 mL/min, λ = 254 nm.

Peak#	Ret. Time	Area	Height	Conc.	Unit	Mark	Name
1	8.552	860754	31256	49.997			
2	10.501	860868	28574	50.003			
Total		1721622	59830				

<Chromatogram>

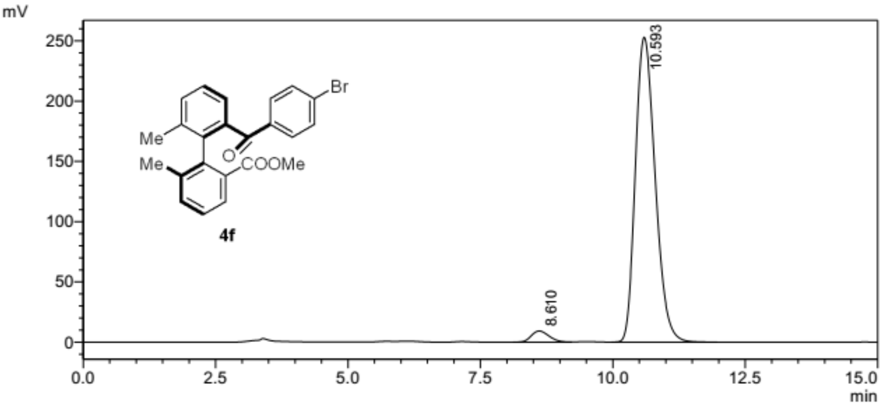

<Peak Table>

Chiralcel AD-H, isopropanol/hexane = 3:97, flow: 1.0 mL/min, λ = 254 nm.

Peak#	Ret. Time	Area	Height	Conc.	Unit	Mark	Name
1	8.610	214493	9208	3.156			
2	10.593	6581071	252863	96.844			
Total		6795564	262071				

<Chromatogram>

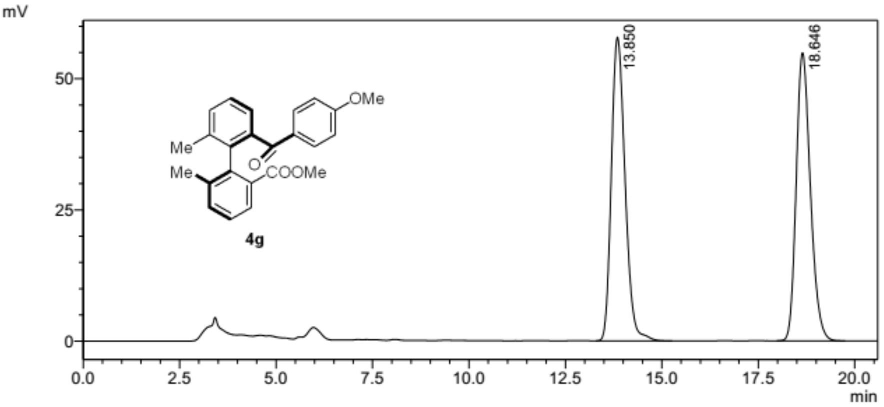

<Peak Table>

Chiralcel AD-H, isopropanol/hexane = 3:97, flow: 1.0 mL/min, λ = 254 nm.

Peak#	Ret. Time	Area	Height	Conc.	Unit	Mark	Name
1	13.850	1429590	57819	50.410			
2	18.646	1406309	54864	49.590			
Total		2835899	112683				

<Chromatogram>

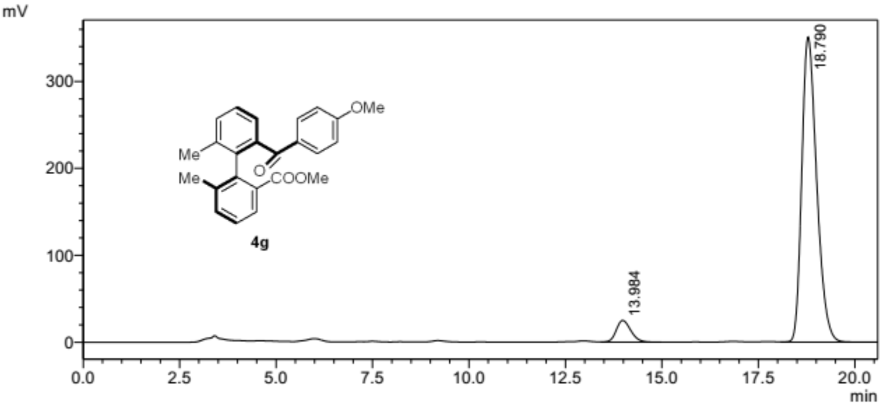

<Peak Table>

Chiralcel AD-H, isopropanol/hexane = 3:97, flow: 1.0 mL/min, λ = 254 nm.

Peak#	Ret. Time	Area	Height	Conc.	Unit	Mark	Name
1	13.984	650128	24910	6.455			
2	18.790	9421351	350939	93.545			
Total		10071479	375849				

<Chromatogram>

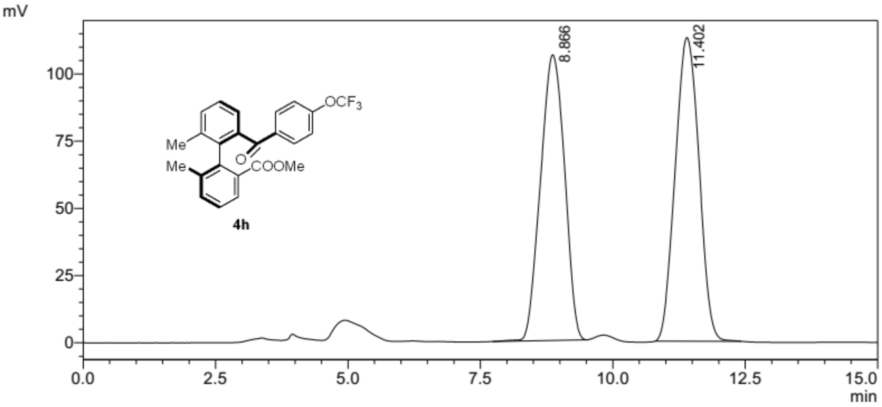

<Peak Table>

Chiralcel AD-H, isopropanol/hexane = 1:99, flow: 1.0 mL/min, λ = 254 nm.

Peak#	Ret. Time	Area	Height	Conc.	Unit	Mark	Name
1	8.866	3357119	106240	48.687			
2	11.402	3538242	112947	51.313			
Total		6895360	219187				

<Chromatogram>

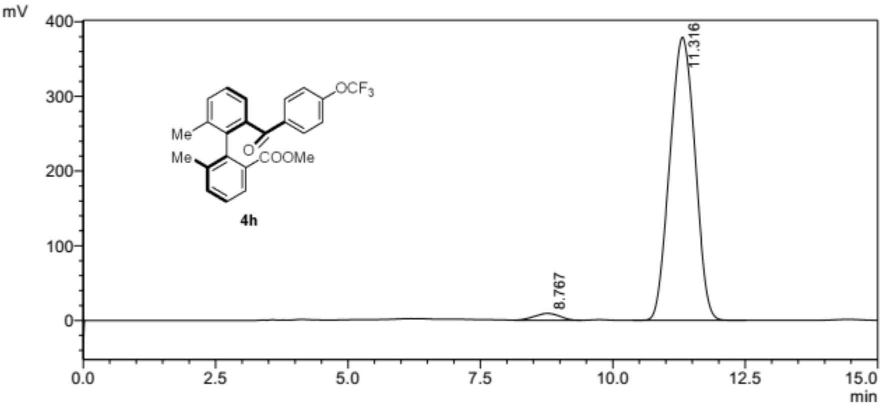

<Peak Table>

Chiralcel AD-H, isopropanol/hexane = 1:99, flow: 1.0 mL/min, λ = 254 nm.

Peak#	Ret. Time	Area	Height	Conc.	Unit	Mark	Name
1	8.767	314730	9325	2.444			
2	11.316	12560621	379039	97.556			
Total		12875350	388364				

<Chromatogram>

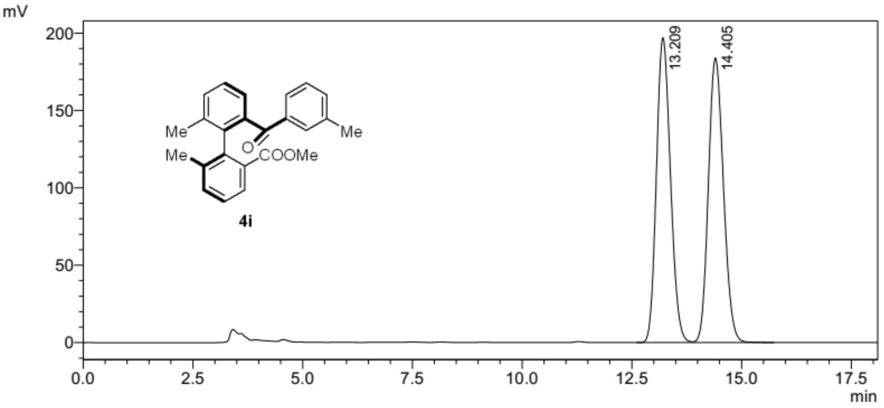

<Peak Table>

Chiralcel IC, isopropanol/hexane = 3:97, flow: 1.0 mL/min, λ = 254 nm.

Peak#	Ret. Time	Area	Height	Conc.	Unit	Mark	Name
1	13.209	4383106	196831	49.938			
2	14.405	4393904	183804	50.062			
Total		8777010	380636				

<Chromatogram>

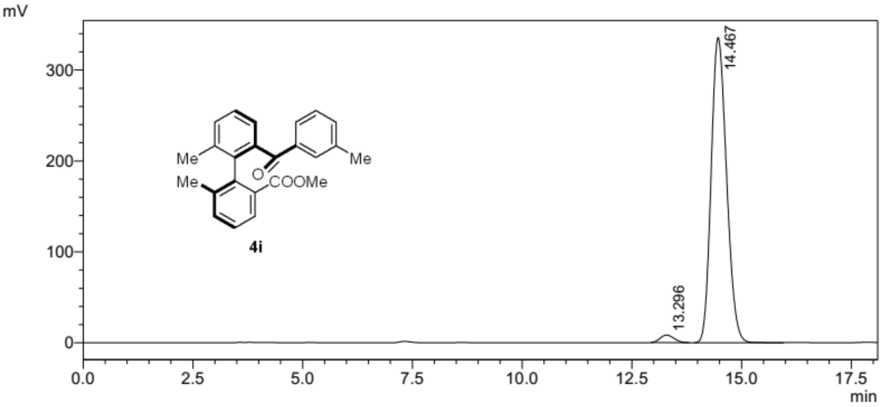

<Peak Table>

Chiralcel IC, isopropanol/hexane = 3:97, flow: 1.0 mL/min, λ = 254 nm.

Peak#	Ret. Time	Area	Height	Conc.	Unit	Mark	Name
1	13.296	182863	8332	2.182			
2	14.467	8195880	335576	97.818			
Total		8378743	343908				

<Chromatogram>

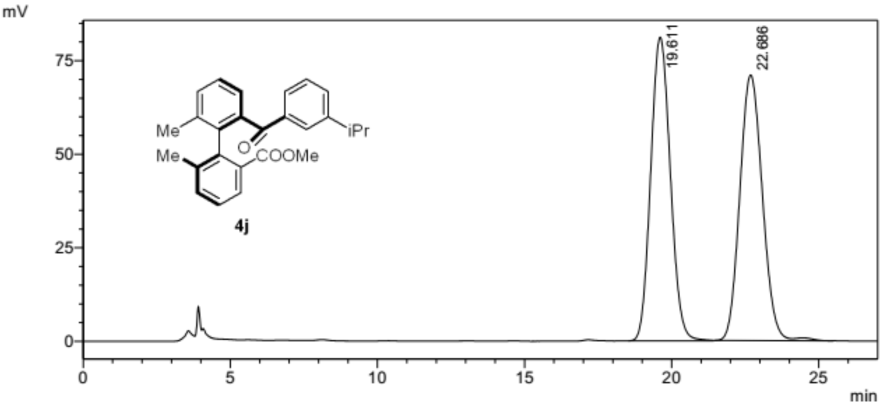

<Peak Table>

Chiralcel IC, isopropanol/hexane = 1:99, flow: 1.0 mL/min, λ = 254 nm.

Peak#	Ret. Time	Area	Height	Conc.	Unit	Mark	Name
1	19.611	3768680	81149	49.884			
2	22.686	3786227	70942	50.116			
Total		7554907	152092				

<Chromatogram>

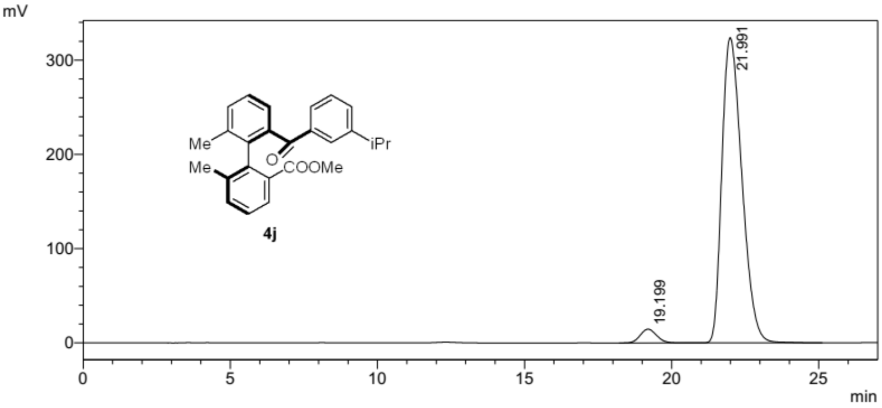

<Peak Table>

Chiralcel IC, isopropanol/hexane = 1:99, flow: 1.0 mL/min, λ = 254 nm.

Peak#	Ret. Time	Area	Height	Conc.	Unit	Mark	Name
1	19.199	568299	14600	3.573			
2	21.991	15335051	323895	96.427			
Total		15903351	338495				

<Chromatogram>

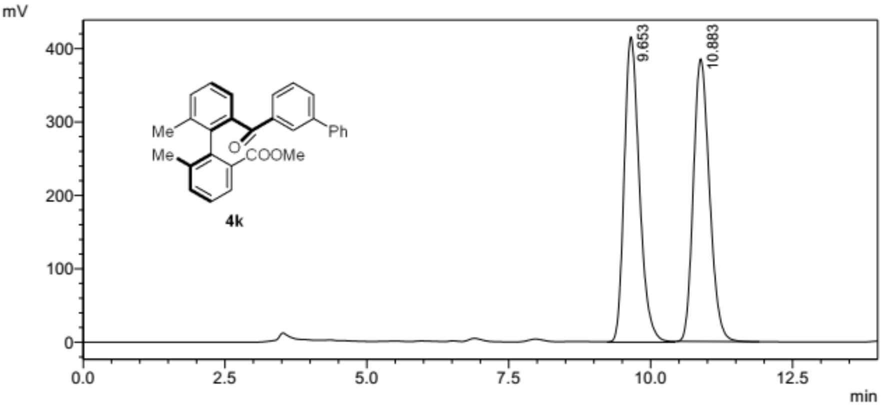

<Peak Table>

Chiralcel IC, isopropanol/hexane = 6:94, flow: 1.0 mL/min, λ = 254 nm.

Peak#	Ret. Time	Area	Height	Conc.	Unit	Mark	Name
1	9.653	7749023	415607	50.384			
2	10.883	7631028	385009	49.616			
Total		15380051	800616				

<Chromatogram>

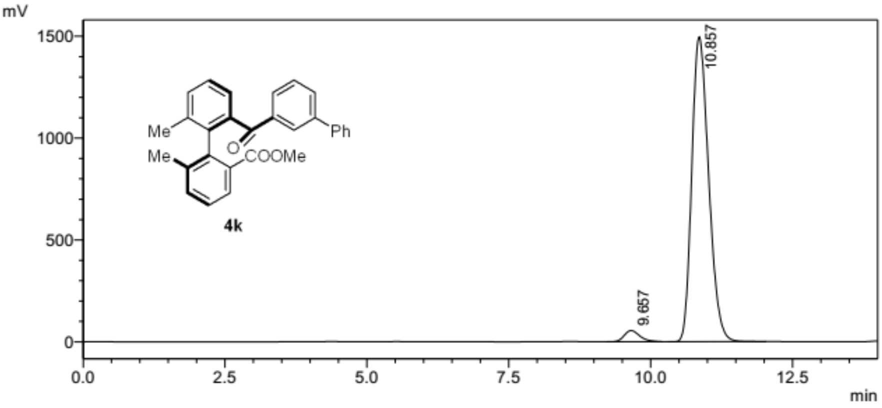

<Peak Table>

Chiralcel IC, isopropanol/hexane = 6:94, flow: 1.0 mL/min, λ = 254 nm.

Peak#	Ret. Time	Area	Height	Conc.	Unit	Mark	Name
1	9.657	1039401	54700	3.240			
2	10.857	31040644	1496297	96.760			
Total		32080045	1550998				

<Chromatogram>

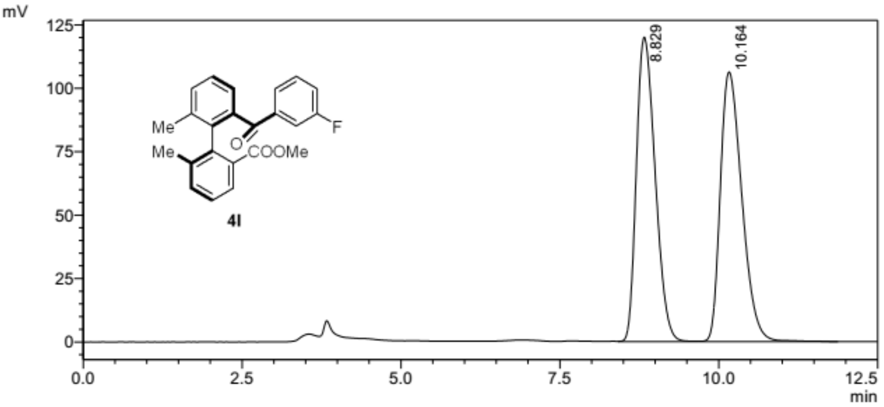

<Peak Table>

Chiralcel ID, isopropanol/hexane = 2:98, flow: 1.0 mL/min, λ = 254 nm.

Peak#	Ret. Time	Area	Height	Conc.	Unit	Mark	Name
1	8.829	2468469	119919	49.599			
2	10.164	2508397	106300	50.401			
Total		4976865	226219				

<Chromatogram>

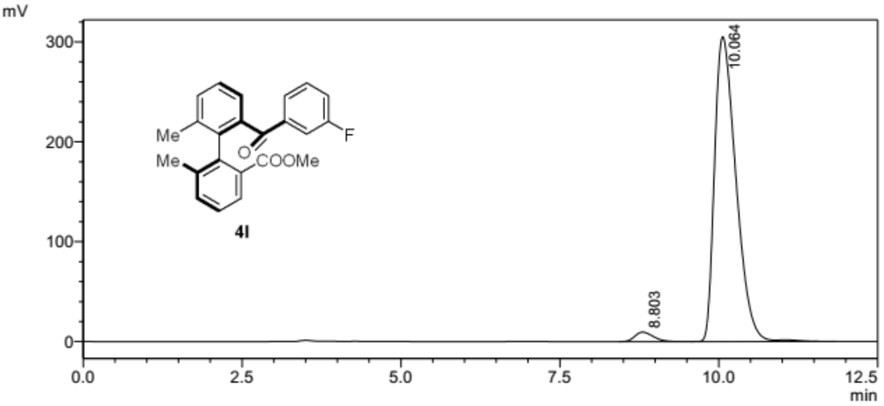

<Peak Table>

Chiralcel ID, isopropanol/hexane = 2:98, flow: 1.0 mL/min, λ = 254 nm.

Peak#	Ret. Time	Area	Height	Conc.	Unit	Mark	Name
1	8.803	190587	9423	2.591			
2	10.064	7165399	305076	97.409			
Total		7355986	314499				

<Chromatogram>

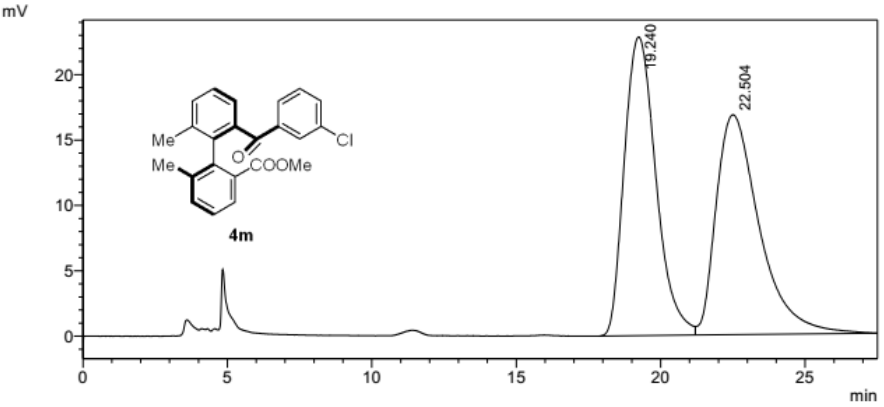

<Peak Table>

Chiralcel ID, isopropanol/hexane = 0.5:99.5, flow: 1.0 mL/min, λ = 254 nm.

Peak#	Ret. Time	Area	Height	Conc.	Unit	Mark	Name
1	19.240	1742368	22844	49.832			
2	22.504	1754090	16824	50.168			
Total		3496458	39668				

<Chromatogram>

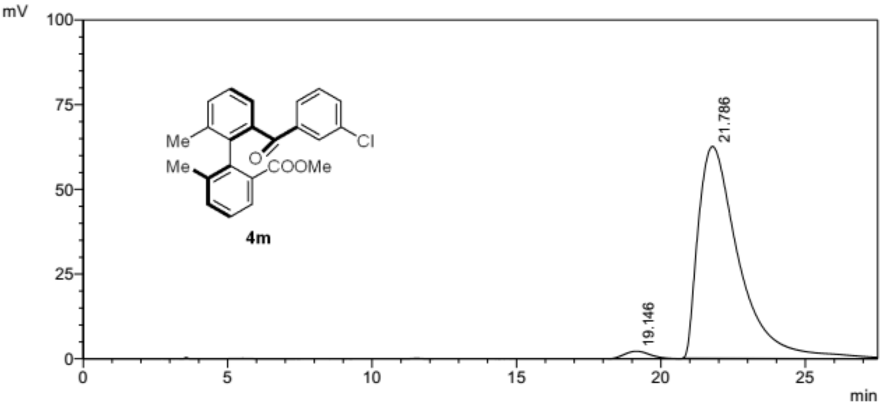

<Peak Table>

Chiralcel ID, isopropanol/hexane = 0.5:99.5, flow: 1.0 mL/min, λ = 254 nm.

Peak#	Ret. Time	Area	Height	Conc.	Unit	Mark	Name
1	19.146	153074	2328	2.429			
2	21.786	6149162	62559	97.571			
Total		6302236	64887				

<Chromatogram>

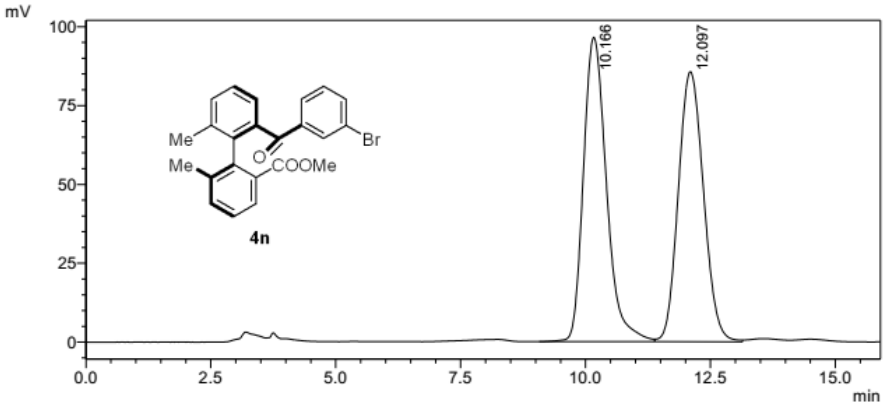

<Peak Table>

Chiralcel OD, isopropanol/hexane = 1:99, flow: 1.0 mL/min, λ = 254 nm.

Peak#	Ret. Time	Area	Height	Conc.	Unit	Mark	Name
1	10.166	3097452	96467	50.778			
2	12.097	3002582	85590	49.222			
Total		6100034	182057				

<Chromatogram>

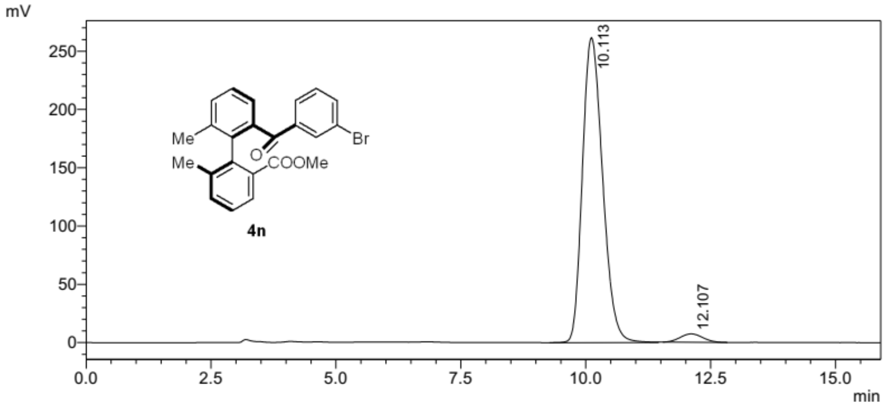

<Peak Table>

Chiralcel OD, isopropanol/hexane = 1:99, flow: 1.0 mL/min, λ = 254 nm.

Peak#	Ret. Time	Area	Height	Conc.	Unit	Mark	Name
1	10.113	7362621	261622	97.033			
2	12.107	225130	7162	2.967			
Total		7587751	268784				

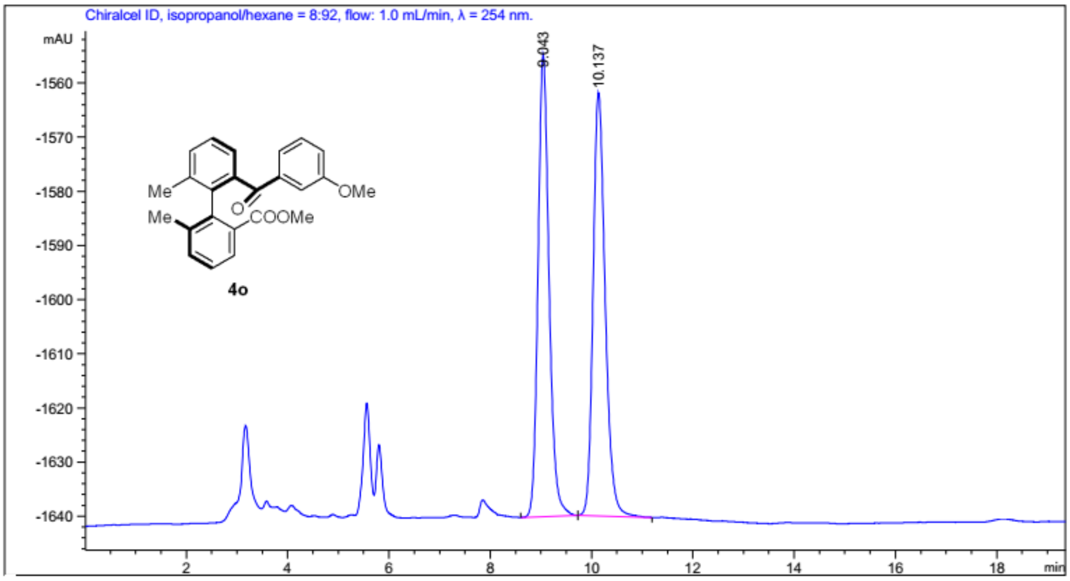

Peak #	RetTime [min]	Type	Width [min]	Area [mAU*s]	Height [mAU]	Area %
1	9.043	BB	0.2301	1288.56226	85.35123	49.9139
2	10.137	BB	0.2511	1293.00940	78.13260	50.0861
Totals :				2581.57166	163.48383	

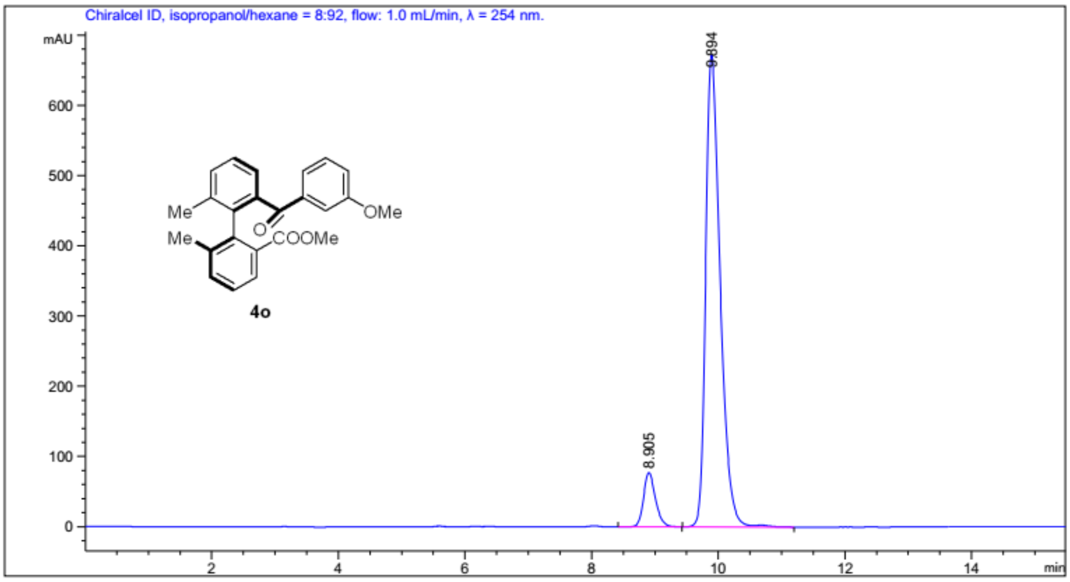

Peak #	RetTime [min]	Type	Width [min]	Area [mAU*s]	Height [mAU]	Area %
1	8.905	BB	0.1988	1012.52521	77.31722	8.6623
2	9.894	BV R	0.2425	1.06763e4	672.42883	91.3377
Totals :				1.16888e4	749.74606	

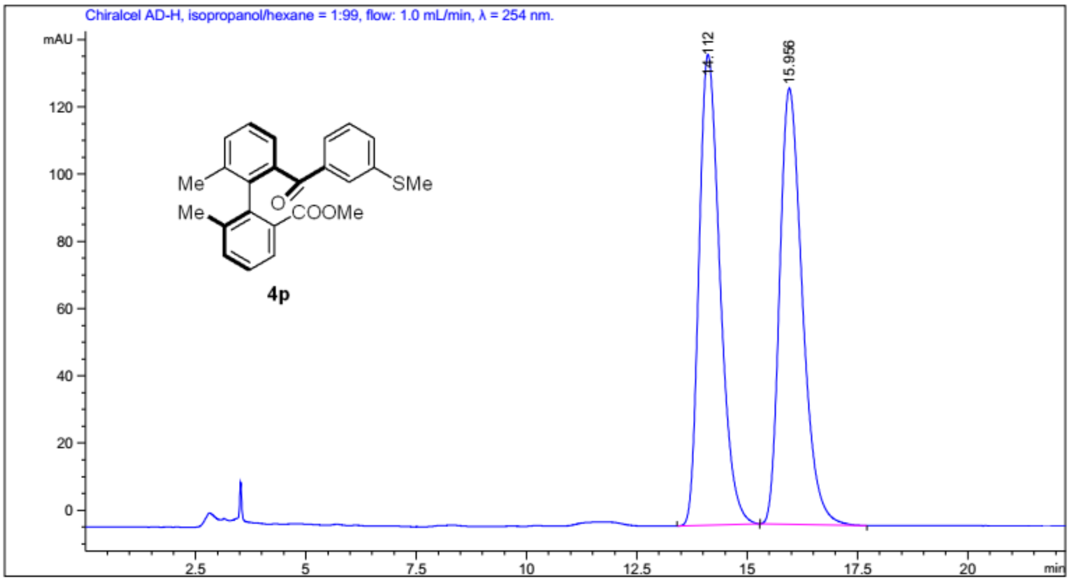

Peak #	RetTime [min]	Type	Width [min]	Area [mAU*s]	Height [mAU]	Area %
1	14.112	BB	0.5127	4617.56641	140.01965	49.9675
2	15.956	BB	0.5538	4623.57422	129.74345	50.0325

Totals : 9241.14063 269.76311

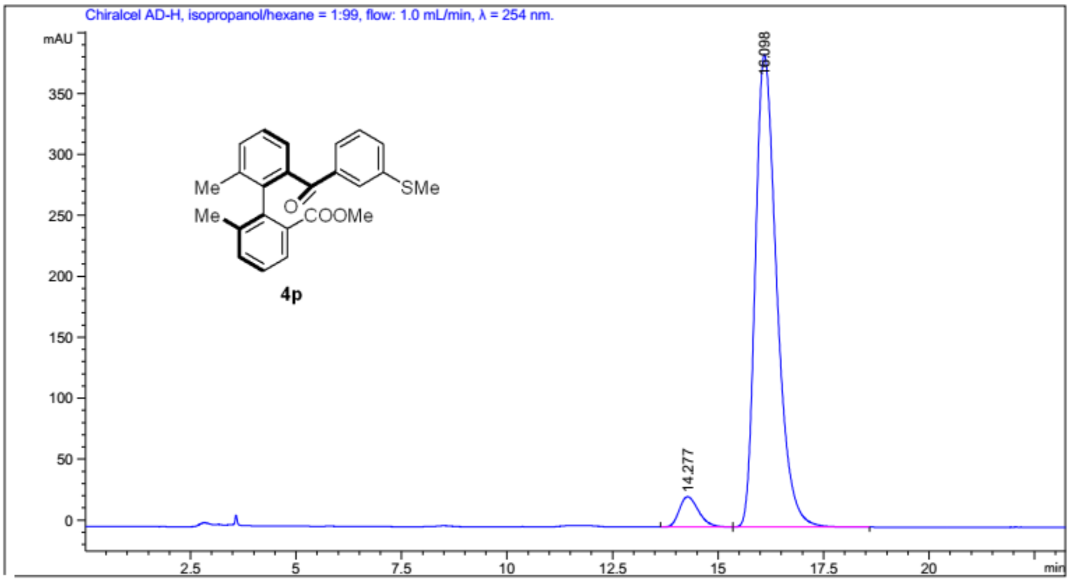

Peak #	RetTime [min]	Type	Width [min]	Area [mAU*s]	Height [mAU]	Area %
1	14.277	BB	0.4948	794.02441	24.97986	5.5045
2	16.098	BB	0.5403	1.36310e4	387.63690	94.4955

Totals : 1.44250e4 412.61676

<Chromatogram>

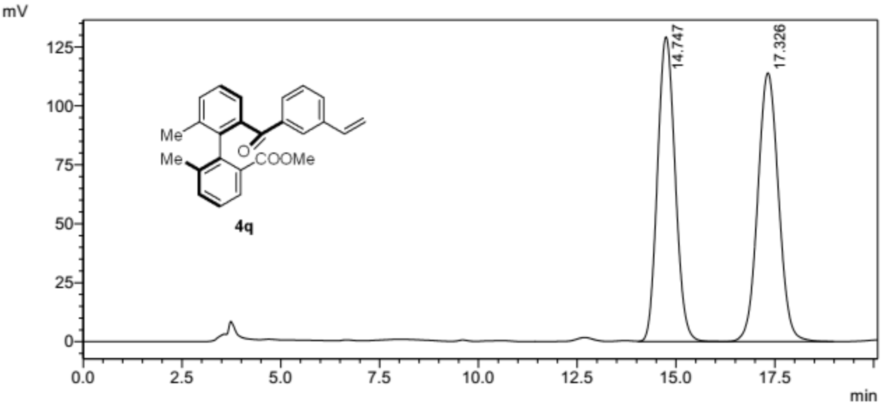

<Peak Table>

Chiralcel IC, isopropanol/hexane = 2:98, flow: 1.0 mL/min, λ = 254 nm.

Peak#	Ret. Time	Area	Height	Conc.	Unit	Mark	Name
1	14.747	4018351	129173	49.110			
2	17.326	4164011	113914	50.890			
Total		8182361	243087				

<Chromatogram>

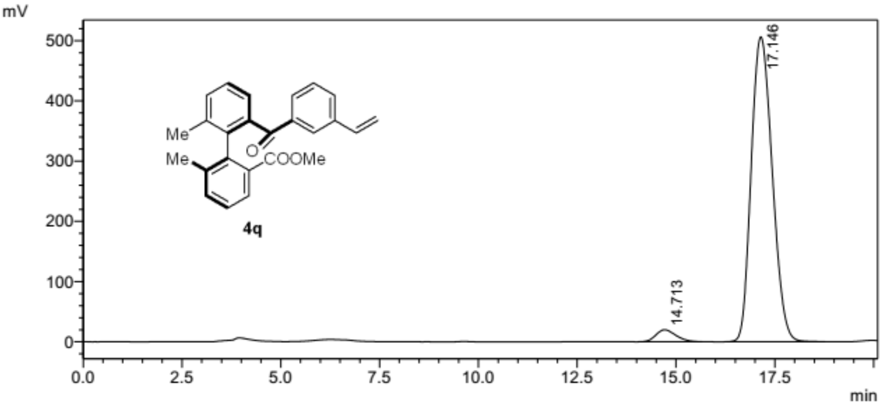

<Peak Table>

Chiralcel IC, isopropanol/hexane = 2:98, flow: 1.0 mL/min, λ = 254 nm.

Peak#	Ret. Time	Area	Height	Conc.	Unit	Mark	Name
1	14.713	697739	19933	3.587			
2	17.146	18756671	506083	96.413			
Total		19454410	526016				

<Chromatogram>

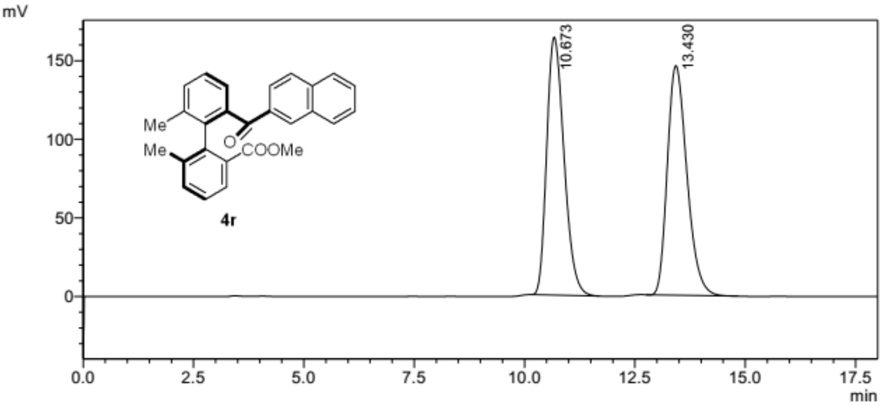

<Peak Table>

Chiralcel AD-H, isopropanol/hexane = 3:97, flow: 1.0 mL/min, λ = 254 nm.

Peak#	Ret. Time	Area	Height	Conc.	Unit	Mark	Name
1	10.673	4489103	164133	49.862			
2	13.430	4513949	146065	50.138			
Total		9003053	310197				

<Chromatogram>

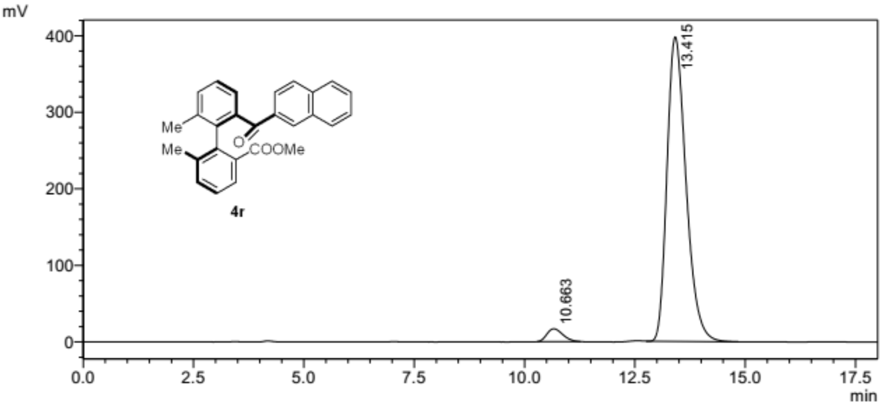

<Peak Table>

Chiralcel AD-H, isopropanol/hexane = 3:97, flow: 1.0 mL/min, λ = 254 nm.

Peak#	Ret. Time	Area	Height	Conc.	Unit	Mark	Name
1	10.663	420072	16779	3.404			
2	13.415	11920739	397475	96.596			
Total		12340811	414254				

<Chromatogram>

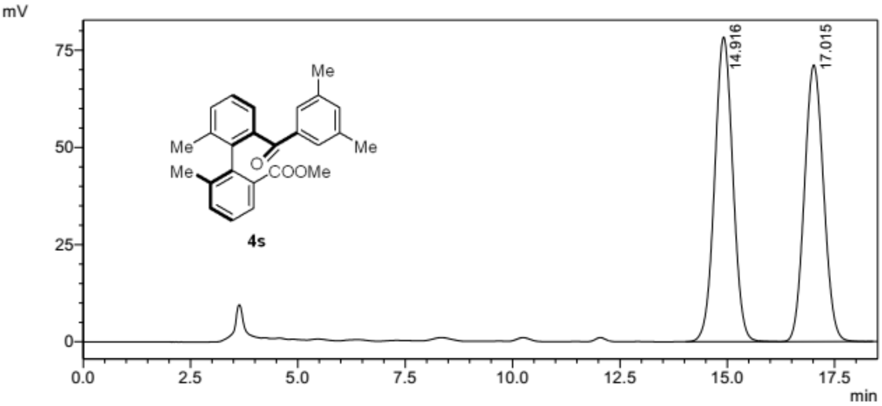

<Peak Table>

Chiralcel IC, isopropanol/hexane = 3:97, flow: 1.0 mL/min, λ = 254 nm.

Peak#	Ret. Time	Area	Height	Conc.	Unit	Mark	Name
1	14.916	2312630	78348	51.173			
2	17.015	2206580	71144	48.827			
Total		4519210	149492				

<Chromatogram>

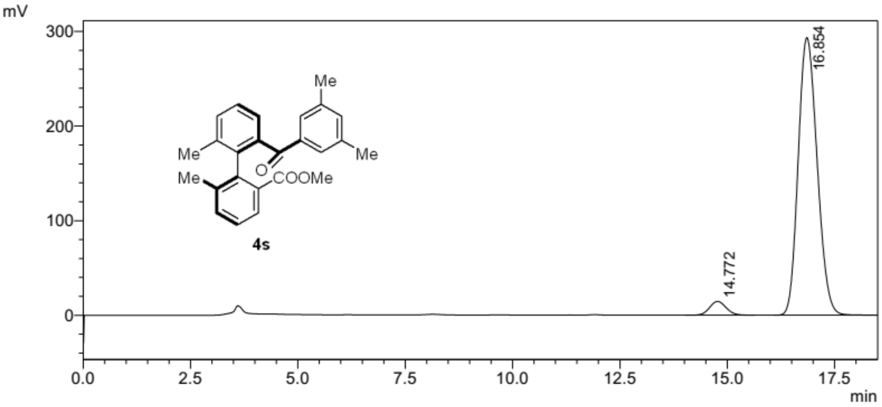

<Peak Table>

Chiralcel IC, isopropanol/hexane = 3:97, flow: 1.0 mL/min, λ = 254 nm.

Peak#	Ret. Time	Area	Height	Conc.	Unit	Mark	Name
1	14.772	386935	14475	4.118			
2	16.854	9009324	293208	95.882			
Total		9396259	307683				

<Chromatogram>

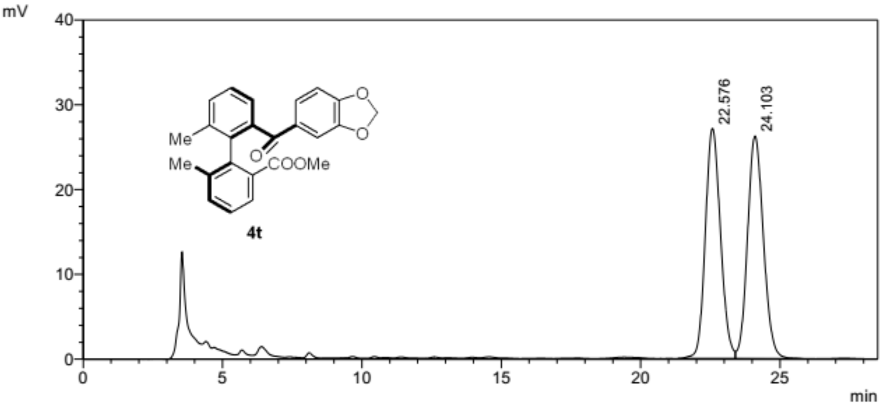

<Peak Table>

Chiralcel IC, isopropanol/hexane = 5:95, flow: 1.0 mL/min, λ = 254 nm.

Peak#	Ret. Time	Area	Height	Conc.	Unit	Mark	Name
1	22.576	1025626	27140	49.658			
2	24.103	1039757	26237	50.342			
Total		2065383	53377				

<Chromatogram>

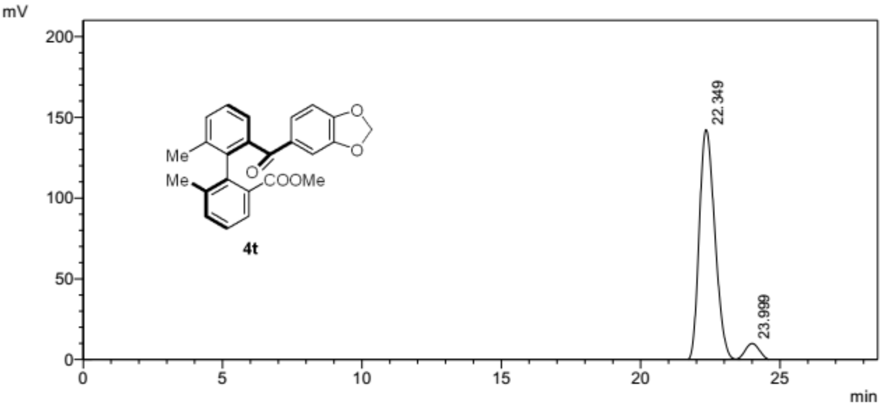

<Peak Table>

Chiralcel IC, isopropanol/hexane = 5:95, flow: 1.0 mL/min, λ = 254 nm.

Peak#	Ret. Time	Area	Height	Conc.	Unit	Mark	Name
1	22.349	5571901	142962	93.723			
2	23.999	373168	10290	6.277			
Total		5945069	153252				

<Chromatogram>

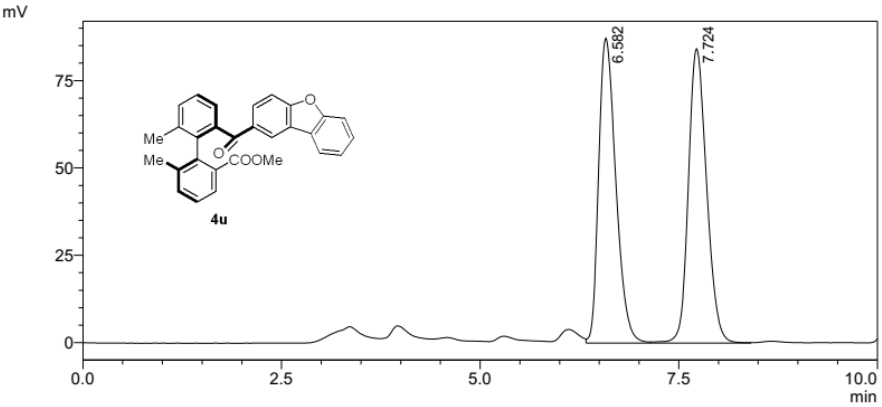

<Peak Table>

Chiralcel AD, isopropanol/hexane = 10:90, flow: 1.0 mL/min, λ = 254 nm.

Peak#	Ret. Time	Area	Height	Conc.	Unit	Mark	Name
1	6.582	1309655	87211	49.338			
2	7.724	1344795	84217	50.662			
Total		2654450	171428				

<Chromatogram>

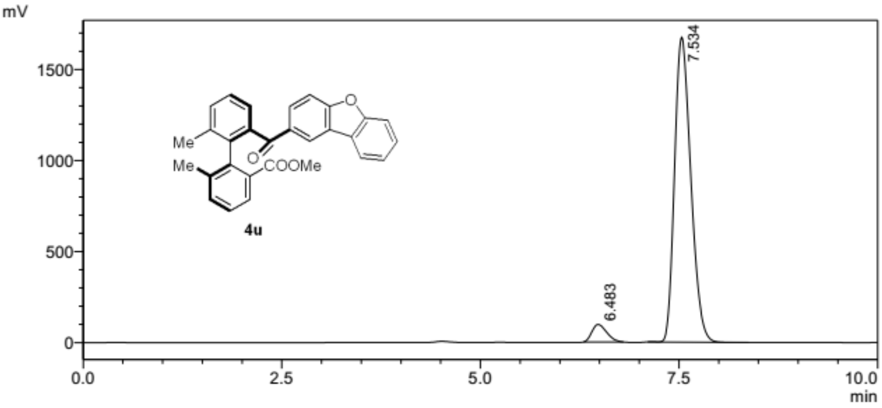

<Peak Table>

Chiralcel AD, isopropanol/hexane = 10:90, flow: 1.0 mL/min, λ = 254 nm.

Peak#	Ret. Time	Area	Height	Conc.	Unit	Mark	Name
1	6.483	1247774	96076	4.968			
2	7.534	23866854	1673890	95.032			
Total		25114628	1769967				

<Chromatogram>

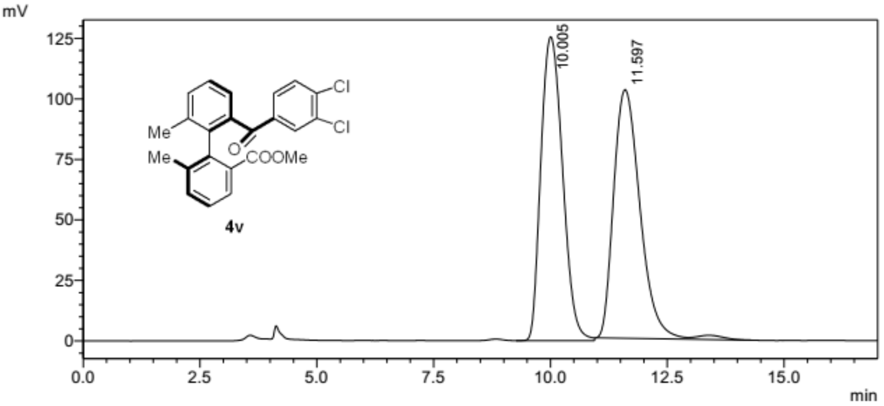

<Peak Table>

Chiralcel ID, isopropanol/hexane = 1:99, flow: 1.0 mL/min, λ = 254 nm.

Peak#	Ret. Time	Area	Height	Conc.	Unit	Mark	Name
1	10.005	4056346	125454	49.852			
2	11.597	4080432	102668	50.148			
Total		8136778	228122				

<Chromatogram>

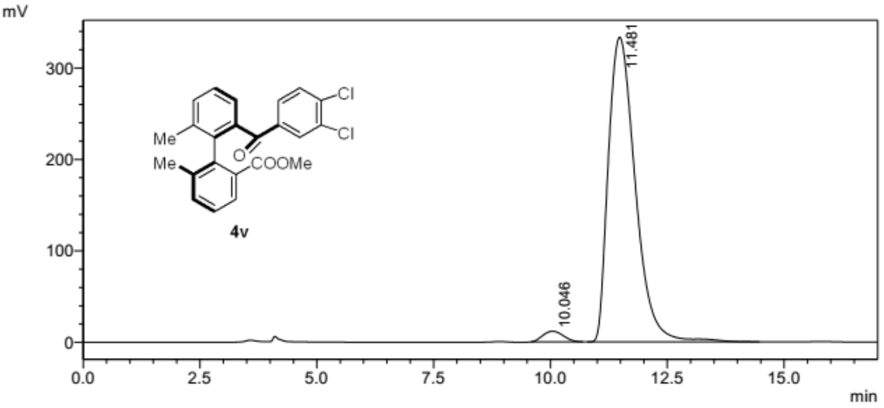

<Peak Table>

Chiralcel ID, isopropanol/hexane = 1:99, flow: 1.0 mL/min, λ = 254 nm.

Peak#	Ret. Time	Area	Height	Conc.	Unit	Mark	Name
1	10.046	355006	11626	2.583			
2	11.481	13391047	333241	97.417			
Total		13746053	344866				

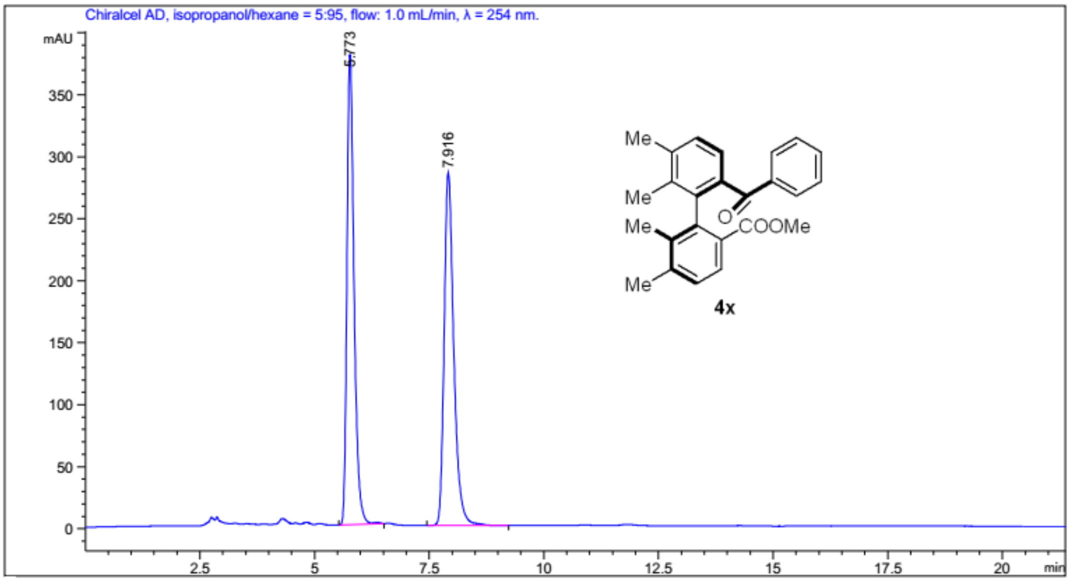

Peak #	RetTime [min]	Type	Width [min]	Area [mAU*s]	Height [mAU]	Area %
1	5.773	BV R	0.1654	4087.43335	379.34399	49.6357
2	7.916	BB	0.2200	4147.42773	284.62881	50.3643

Totals : 8234.86108 663.97281

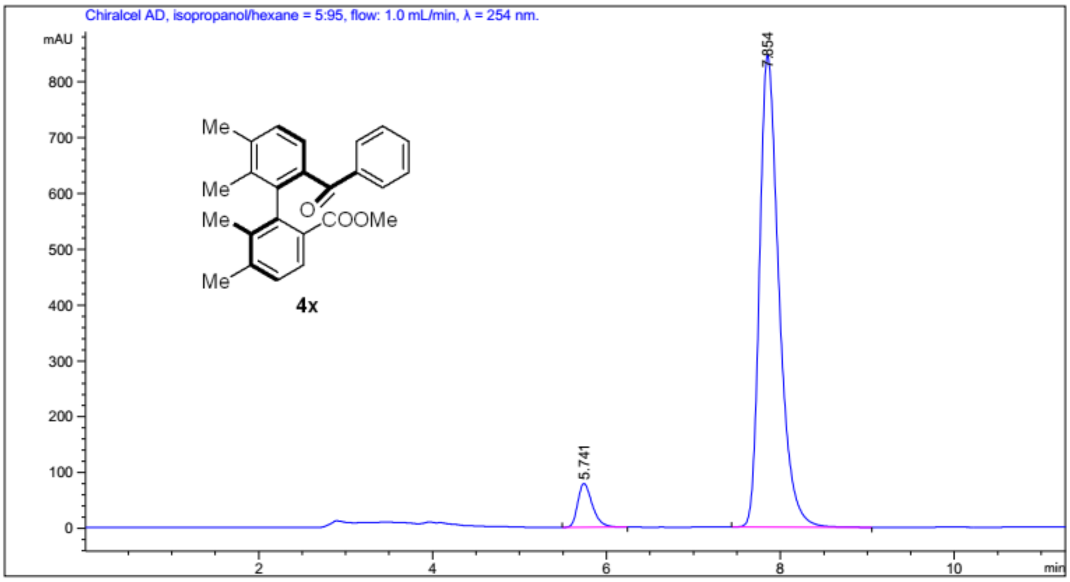

Peak #	RetTime [min]	Type	Width [min]	Area [mAU*s]	Height [mAU]	Area %
1	5.741	BB	0.1778	912.67731	78.41914	6.5127
2	7.854	BB	0.2368	1.31010e4	845.47894	93.4873

Totals : 1.40137e4 923.89808

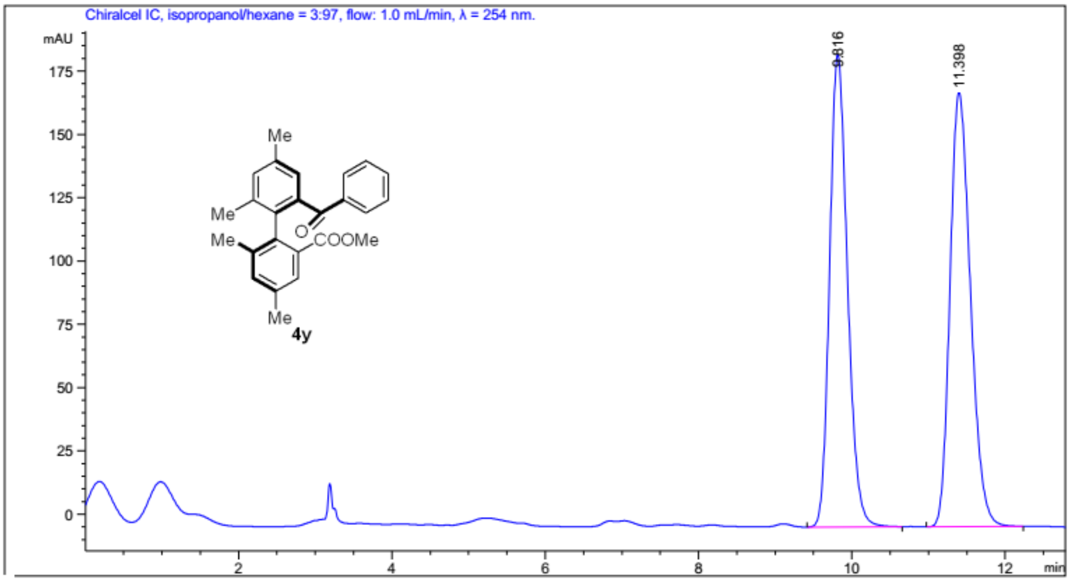

Peak #	RetTime [min]	Type	Width [min]	Area [mAU*s]	Height [mAU]	Area %
1	9.816	BB	0.2482	3008.21729	186.52615	48.5895
2	11.398	BB	0.2885	3182.87231	171.48180	51.4105

Totals : 6191.08960 358.00795

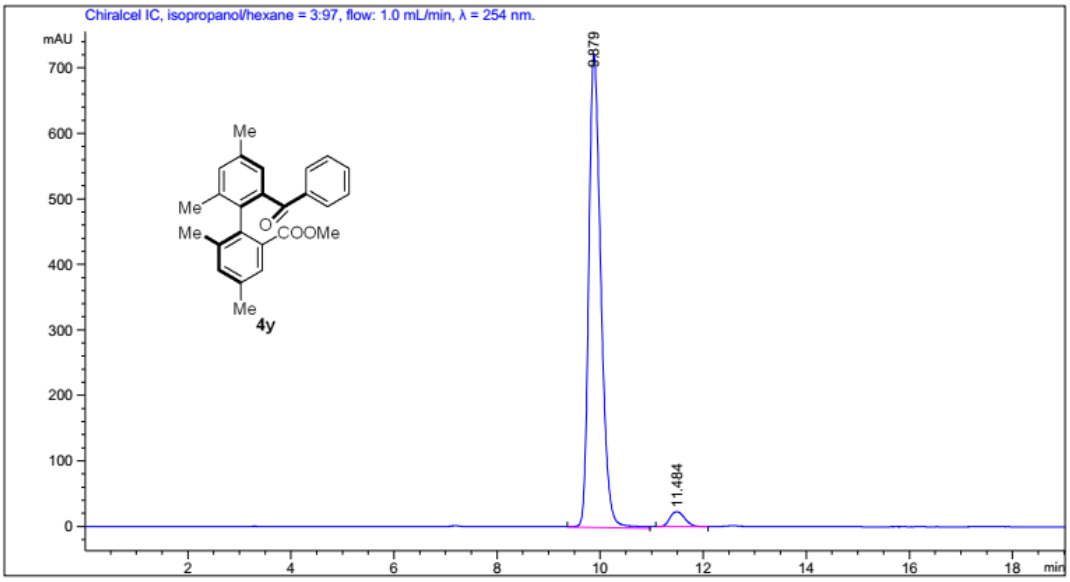

Peak #	RetTime [min]	Type	Width [min]	Area [mAU*s]	Height [mAU]	Area %
1	9.879	MM	0.2715	1.17524e4	721.39233	96.3052
2	11.484	BB	0.3199	450.89429	22.68839	3.6948

Totals : 1.22033e4 744.08072

<Chromatogram>

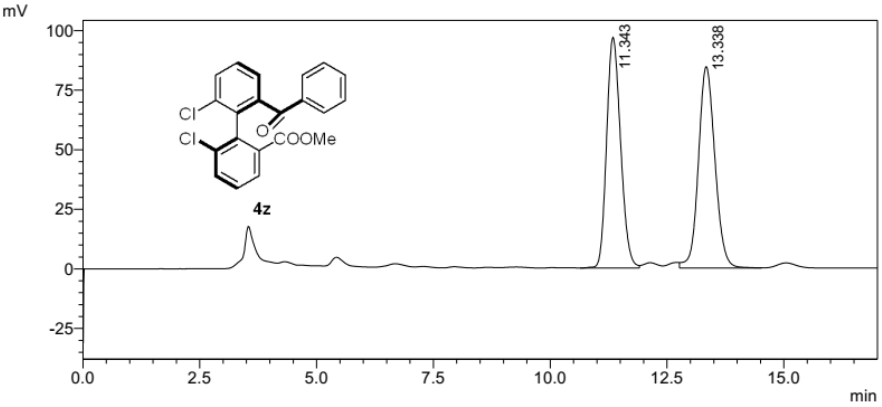

<Peak Table>

Chiralcel IC, isopropanol/hexane = 5:95, flow: 1.0 mL/min, λ = 254 nm.

Peak#	Ret. Time	Area	Height	Conc.	Unit	Mark	Name
1	11.343	2014855	96776	49.158			
2	13.338	2083841	84524	50.842			
Total		4098696	181299				

<Chromatogram>

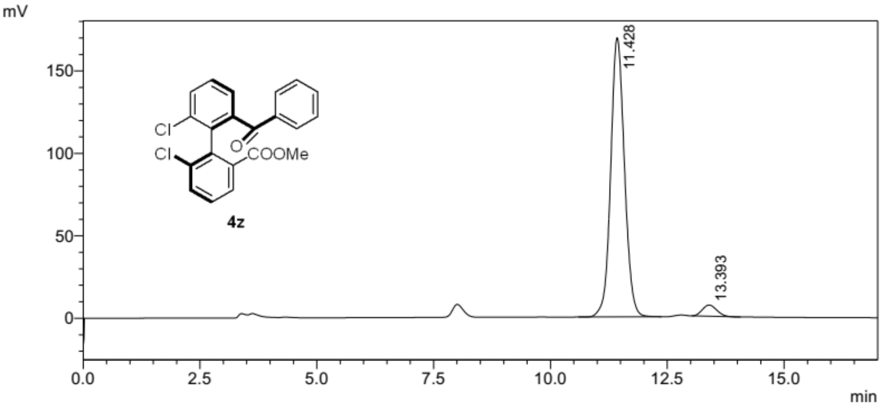

<Peak Table>

Chiralcel IC, isopropanol/hexane = 5:95, flow: 1.0 mL/min, λ = 254 nm.

Peak#	Ret. Time	Area	Height	Conc.	Unit	Mark	Name
1	11.428	3474192	169270	95.930			
2	13.393	147409	6819	4.070			
Total		3621600	176089				

<Chromatogram>

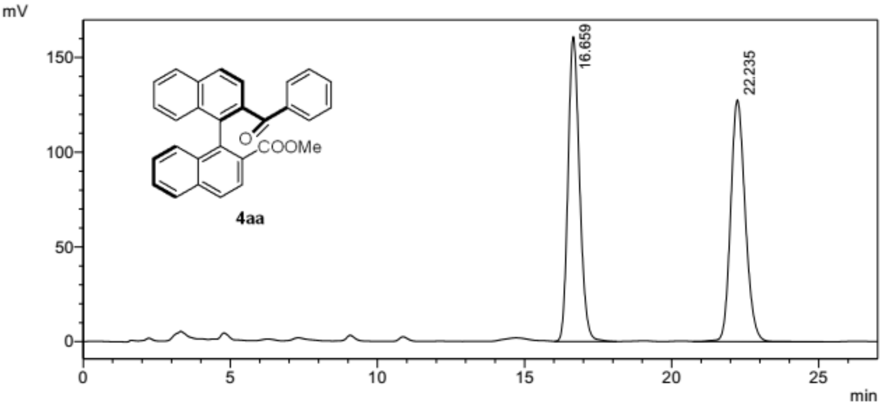

<Peak Table>

Chiralcel AD-H, isopropanol/hexane = 5:95, flow: 1.0 mL/min, λ = 254 nm.

Peak#	Ret. Time	Area	Height	Conc.	Unit	Mark	Name
1	16.659	4364552	160788	49.894			
2	22.235	4383049	127532	50.106			
Total		8747601	288320				

<Chromatogram>

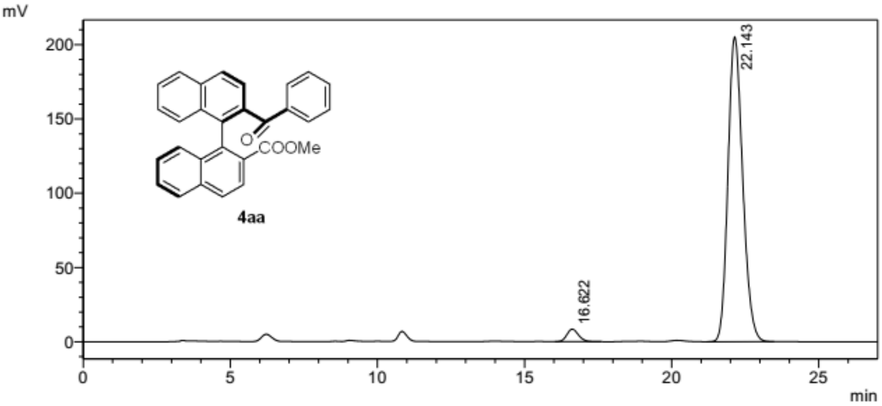

<Peak Table>

Chiralcel AD-H, isopropanol/hexane = 5:95, flow: 1.0 mL/min, λ = 254 nm.

Peak#	Ret. Time	Area	Height	Conc.	Unit	Mark	Name
1	16.622	232293	8423	3.148			
2	22.143	7147266	205125	96.852			
Total		7379559	213548				

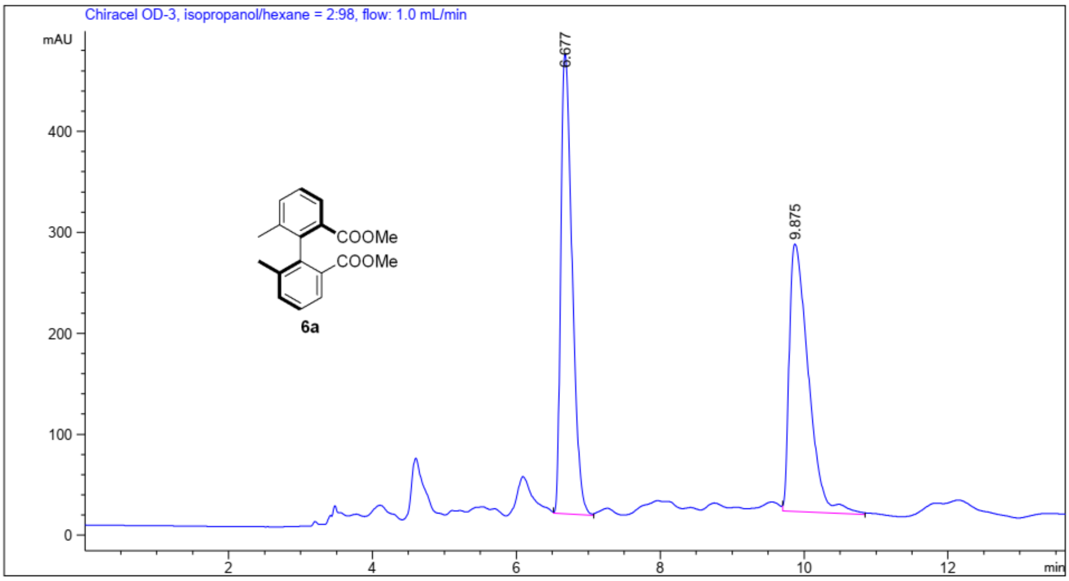

Signal 1: DAD1 A, Sig=254,4 Ref=360,100

Peak #	RetTime [min]	Type	Width [min]	Area [mAU*s]	Height [mAU]	Area %
1	6.677	MM	0.1819	4969.91357	455.35446	50.5217
2	9.875	MM	0.3064	4867.26709	264.71515	49.4783

Totals : 9837.18066 720.06961

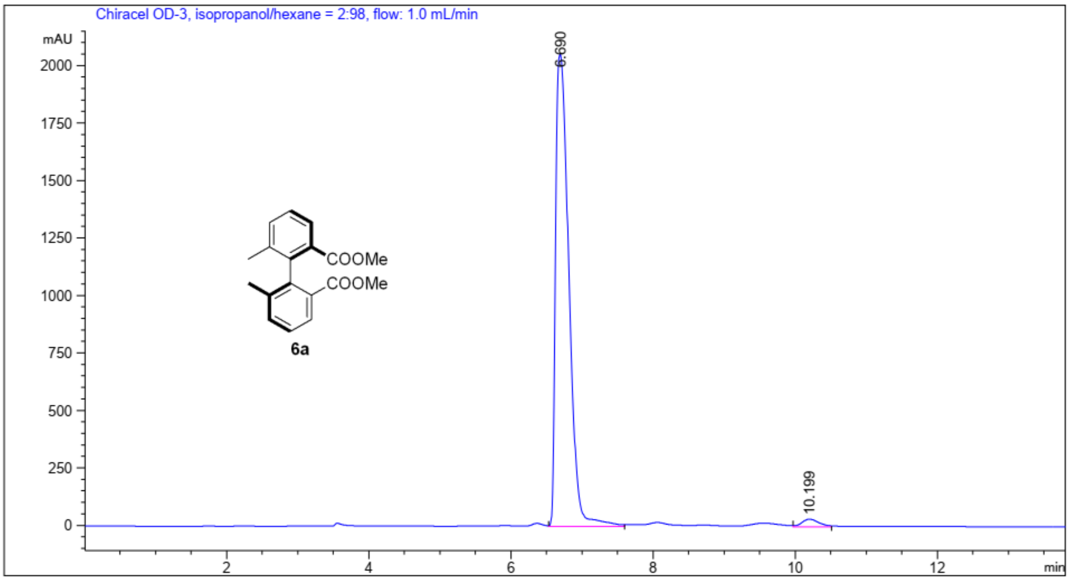

Signal 1: DAD1 A, Sig=254,4 Ref=360,100

Peak #	RetTime [min]	Type	Width [min]	Area [mAU*s]	Height [mAU]	Area %
1	6.690	VV	0.2051	2.66014e4	2053.31543	97.8223
2	10.199	MM	0.2899	592.20563	34.05058	2.1777

Totals : 2.71936e4 2087.36600

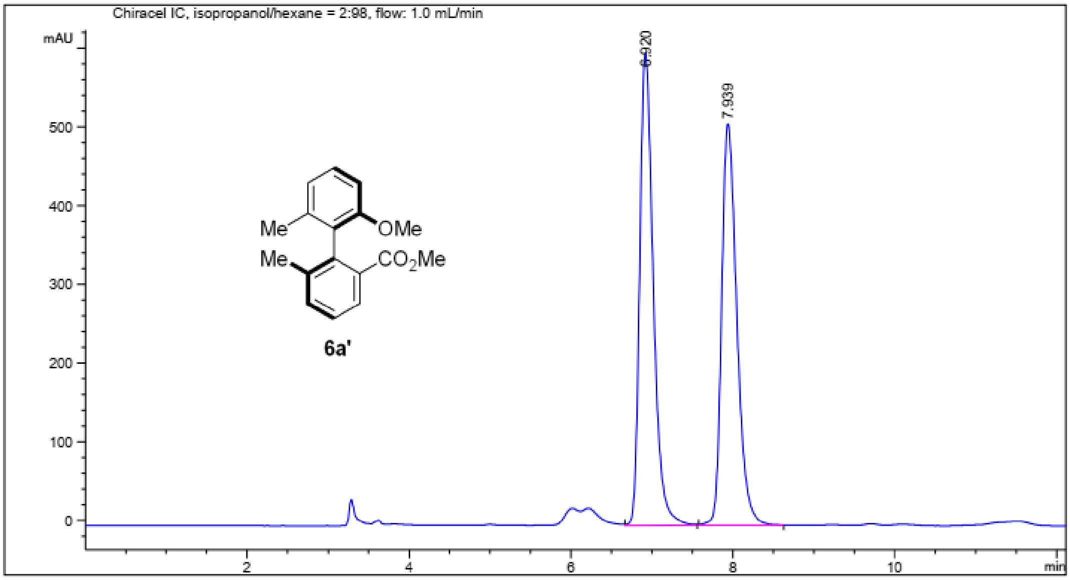

Signal 1: DAD1 A, Sig=254,4 Ref=360,100

Peak #	RetTime [min]	Type	Width [min]	Area [mAU*s]	Height [mAU]	Area %
1	6.920	VB	0.1772	6944.48096	599.41467	51.1887
2	7.939	BB	0.2014	6621.96191	510.23444	48.8113

Totals : 1.35664e4 1109.64911

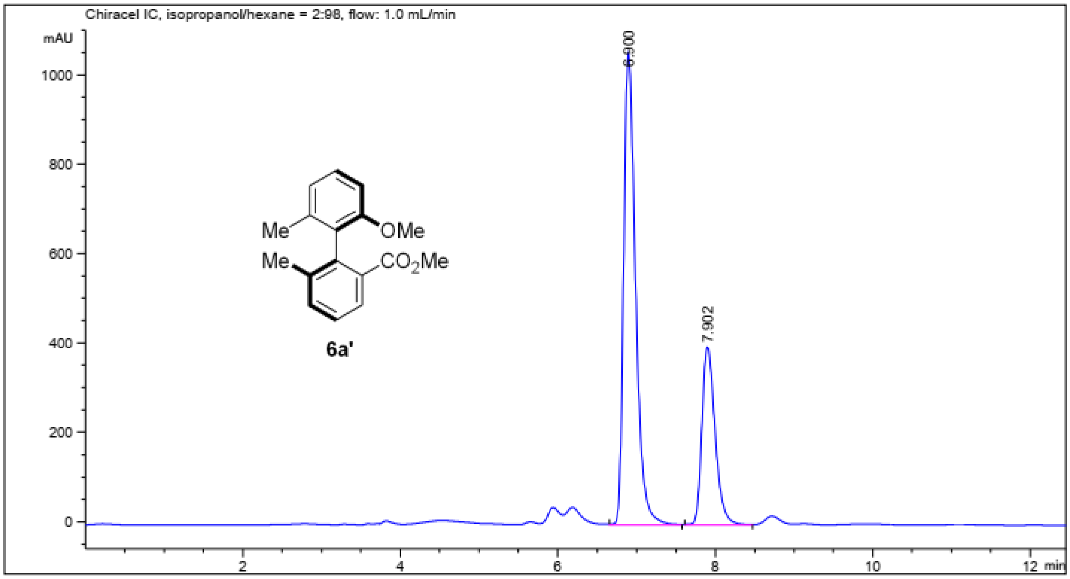

Signal 1: DAD1 A, Sig=254,4 Ref=360,100

Peak #	RetTime [min]	Type	Width [min]	Area [mAU*s]	Height [mAU]	Area %
1	6.900	BB	0.1622	1.10462e4	1055.00159	70.4349
2	7.902	BV	0.1798	4636.64697	398.30685	29.5651

Totals : 1.56828e4 1453.30844
